# Two Separate Clusters of SARS-CoV-2 Delta Variant Infections in a Group of 41 Students Travelling from India: An Illustration of the Need for Rigorous Testing and Quarantine

**DOI:** 10.3390/v14061198

**Published:** 2022-05-31

**Authors:** Jan Van Elslande, Femke Kerckhofs, Lize Cuypers, Elke Wollants, Barney Potter, Anne Vankeerberghen, Lien Cattoir, Astrid Holderbeke, Sylvie Behillil, Sarah Gorissen, Mandy Bloemen, Jef Arnout, Marc Van Ranst, Johan Van Weyenbergh, Piet Maes, Guy Baele, Pieter Vermeersch, Emmanuel André, on behalf of the COG-Belgium Consortium

**Affiliations:** 1 Clinical Department of Laboratory Medicine and National Reference Center for Respiratory Pathogens, University Hospitals Leuven, BE3000 Leuven, Belgium; janvanelslande@gmail.com (J.V.E.); femke.kerckhofs@hotmail.com (F.K.); lize.cuypers@uzleuven.be (L.C.); sarah.gorissen@kuleuven.be (S.G.); marc.vanranst@uzleuven.be (M.V.R.); pieter.vermeersch@uzleuven.be (P.V.); 2 Laboratory of Clinical Bacteriology and Mycology, Department of Microbiology, Immunology and Transplantation, Rega Institute, KU Leuven, BE3000 Leuven, Belgium; johan.vanweyenbergh@kuleuven.be; 3 Laboratory of Clinical and Epidemiological Virology, Department of Microbiology, Immunology and Transplantation, Rega Institute, KU Leuven, BE3000 Leuven, Belgium; elke.wollants@uzleuven.be (E.W.); mandy.bloemen@kuleuven.be (M.B.); piet.maes@kuleuven.be (P.M.); 4 Laboratory of Clinical and Evolutionary Virology, Immunology and Transplantation, Department of Microbiology, Rega Institute, KU Leuven, BE3000 Leuven, Belgium; barney.potter.24@gmail.com (B.P.); guy.baele@kuleuven.be (G.B.); 5 Laboratory of Clinical Microbiology, OLV Hospital Aalst, BE9300 Aalst, Belgium; anne.vankeerberghen@olvz-aalst.be (A.V.); lien.cattoir@olvz-aalst.be (L.C.); astrid.holderbeke@olvz-aalst.be (A.H.); 6 Institut Pasteur, Molecular Genetics of RNA Viruses, Université de Paris, CNRS UMR 3569, FR75000 Paris, France; sylvie.behillil@pasteur.fr; 7 Institut Pasteur, National Reference Center for Respiratory Viruses, FR75000 Paris, France; 8 Biomedical Sciences Group Management, KU Leuven, BE3000 Leuven, Belgium; jef.arnout@kuleuven.be; 9 Department of Cardiovascular Sciences, KU Leuven, BE3000 Leuven, Belgium

**Keywords:** SARS-CoV-2, COVID-19, SARS-CoV-2 variants, Delta variant, communicable disease control, quarantine

## Abstract

We report two clusters of SARS-CoV-2 B.1.617.2 (Delta variant) infections in a group of 41 Indian nursing students who travelled from New Delhi, India, to Belgium via Paris, France. All students tested negative before departure and had a second negative antigen test upon arrival in Paris. Upon arrival in Belgium, the students were quarantined in eight different houses. Four houses remained COVID-free during the 24 days of follow-up, while all 27 residents of the other four houses developed an infection during quarantine, including the four residents who were fully vaccinated and the two residents who were partially vaccinated. Genome sequencing revealed two distinct clusters affecting one and three houses, respectively. In this group of students, vaccination status did not seem to prevent infection nor decrease the viral load. No severe symptoms were reported. Extensive contact tracing and 3 months of nationwide genomic surveillance confirmed that these outbreaks were successfully contained and did not contribute to secondary community transmission in Belgium. These clusters highlight the importance of repeated testing and quarantine measures among travelers coming from countries experiencing a surge of infections, as all infections were detected 6 days or more after arrival.

## 1. Introduction

Since the emergence of severe acute respiratory syndrome coronavirus 2 (SARS-CoV-2) at the end of 2019, over one thousand genetic lineages have arisen as a result of continuous mutations and selective pressure. Several of these variants have been categorized as “variants of concern” (VOCs) because of increased transmissibility and/or immune escape. To mitigate the risk of rapid worldwide spread of VOCs associated with international travel, systematic testing of airline travelers has been proposed as an effective strategy to reduce population-level transmission risk, especially when combined with post-travel quarantine. Testing should be performed regardless of symptoms, as an estimated 30–40% of people infected with SARS-CoV-2 are asymptomatic [1]. Further, quarantine is recommended to compensate the intrinsic limits of pre-travel screening, which can fail to detect the early phase of infection, a phenomenon which will occur more frequently among travelers originating from areas experiencing a surge of infections. A combination of pre-travel testing, quarantine of ≥10 days and RT-PCR testing on quarantine exit is likely the most effective international control measure, outperforming a quarantine of 14 days without testing [2,3]. A recent modelling study by Leung et al. suggested that even with vaccination efficacies of 50–60%, a 5-day quarantine and 2 PCR tests are required to reduce the force of infection of inbound travelers by 92–96%. To increase this number to 99%, a 7-day quarantine would be required [4].

The B.1.167.2 VOC, also named the SARS-CoV-2 “Delta variant”, was first detected in Maharashtra (India) in December 2020. This lineage carries several mutations in the spike (S) protein which are associated with higher transmissibility and partial escape from immunity induced by natural infection or vaccination [5,6,7]. In February and March 2021, the Delta variant started to spread rapidly across India, replacing the Alpha variant as the dominant strain. This viral replacement was associated with a surge in case numbers and an extensive rise in hospitalizations and deaths in India in April and May 2021 [7,8]. The Delta variant quickly spread to other countries around the world, becoming the dominant strain in the UK in June and subsequently in Belgium in July 2021 [9,10]. There are no reports on variant-specific quarantine strategies, although some studies have recommended to use more stringent quarantine strategies for travelers coming from areas with a high prevalence of VOCs [3,4].

In the present article, we describe how a combination of quarantine and repeated testing successfully contained SARS-CoV-2 Delta variant infections within a group of 41 Indian students who travelled to Belgium in April 2021. Using genomic surveillance data, we demonstrated that onward local transmission into the Belgian population was prevented using this strategy.

## 2. Materials and Methods

### 2.1. Diagnostic RT-PCR

RT-PCR for SARS-CoV-2 on nasopharyngeal swabs was performed using a TaqPath COVID-19 CE-IVD RT-PCR assay targeting the genes ORF1ab, spike (S) and nucleocapsid (N) (ThermoFisher Scientific, Waltham, MA, USA) at the clinical laboratory of UZ Leuven, or an in-house RT-PCR method at the clinical laboratory of OLV Aalst. The RT-PCR in Aalst targeted the E-gene (Forward primer 5′-CTTTTTCTTGCTTTCGTGGTATTCT-3′ (300 nM); reverse primer 5′-TACAARACTCACGTTAACAATATTGCA-3′ (300 nM) and TaqMan probe 5′-Cy5-AGCCATCCTTACTGCGCTTCGATTGTG-BHQ2-3′(400 nM)) and the N1 gene (CDC sequences: Forward primer 5′-GACCCCAAAATCAGCGAAAT-3′ (300 nM); reverse primer 5′-TCTGGTTACTGCCAGTTGAATCTG-3′ (300 nM) and TaqMan probe 5′-FAM-ACCCCGCATTACGTTTGGTGGACC-BHQ1-3′ (200 nM)) using the Taqman^®^ fast virus 1-step master mix (ThermoFisher Scientific, Waltham, MA, USA) and temperature profile (Ta = 60 °C).

### 2.2. Whole Genome Sequencing

In short, RNA extraction was performed using Kingfisher, while the genomes were amplified using the ARTIC network V3 primer set. After clean-up of the amplicons, libraries were prepared using the ligation sequencing kit from Oxford Nanopore Technologies following the ARTIC network nCoV-2019 sequencing protocol, version 3 [11]. Sequencing was performed on a GridION platform using MinKNOW’s built-in basecalling, demultiplexing and adapter trimming. Sequencing runs were processed using the ARTIC analysis pipeline and custom scripts. Full-length genome sequences accompanied with metadata were submitted to GISAID (See Appendix A). SARS-CoV-2 lineage assignments were determined using the Pangolin COVID-19 Lineage Assigner as well as Nextclade [12].

### 2.3. Subegenomic RNA (sgRNA)

Subgenomic RNA (sgRNA) is a marker for active viral replication, as it is indicative of transcription of the individual genes to mRNA. An in-house qPCR for sgRNA was developed that targets 236 nucleotides (NT) in the E gene of SARS-CoV-2. Forward SARS-CoV2-E: GAA CTT ATG TAC TCA TTC GTT TCG (NT:26.239–26.262), Reverse SARS-CoV2-E: TCG TTT AGA CCA GAA GAT CAG G (NT:26.475–26.455) and Probe SARS-CoV2-E: FAM-TGC GCT TCG ATT GTG TGC GTA CTG CT-TAMRA. For the detection of sgRNA the qPCR in the E gene was adapted. The forward primer was changed with a different forward primer that was used in the ARTIC V1 panel located in the 5′ leader sequence of the SARS-CoV2 genome (NT:30–54) nCoV-2019_1_LEFT: ACCAACCAACTTTCGATCTCTTGT. RT-qPCR was performed on a QuantStudio 7 (Applied Biosystems). A mix was created with 5 µL Taqman Fast Advanced Master Mix (Applied Biosystems, CAT 4444556), 1 µL Forward primer:nCoV 2019_1_LEFT (20 µM), 1 µL Reverse primer: SARS-CoV2-E (20 µM), 0.5 µL probe: SARS-CoV-E (10 µM) and 7.5 µL of RNAse-free water. Cycling conditions were 5 min at 50 °C, 20 s at 95 °C followed by 45 cycles of 3 s at 95 °C and 30 s at 60 °C.

### 2.4. Phylogenetic Analysis

We employed a pipeline of different software packages to perform a detailed Bayesian phylogeographic analysis of the 24 available genomes out of the 27 reported infections among the traveling students. Initially, we constructed a dedicated Nextstrain build by extending the global Nextstrain build (https://nextstrain.org/ncov/gisaid/global (accessed on 12 July 2021)) with the genomes of 24 infected traveling students, as well as all other available Belgian Delta variant genomes, and the French and Indian Delta variant genomes until the end of April 2021 [13]. We discarded genomes with imprecise collection dates. We used the default settings of the Nextstrain build process to generate a large-scale maximum-likelihood (ML) phylogeny consisting of 13,389 genomes. This phylogeny indicated that the 24 sequenced genomes could be found in two separate clusters within the overall Delta variant clade (see Supplementary Figure S2) with one cluster containing eight genomes and the other containing sixteen (see Supplementary Figure S1).

To study each of these two student clusters in more detail, we performed a Bayesian phylogeographic analysis on the two large clades in the overall phylogeny that comprise our clusters of interest, leading to 777 and 818 Delta variant genomes to be analyzed, respectively. The genomes for these clades were retrieved from GISAID and (re-)aligned using MAFFT v7.475 [14,15]. See supplementary Table S2 for GISAID acknowledgments. We estimated unrooted ML phylogenies for these clades using IQ-TREE v2.1.2 and used these to detect outliers in TempEst, which were subsequently removed from the data sets [16,17]. We used BEAST 1.10 to perform Bayesian phylogeographic reconstruction on both data sets, using countries as the discrete location states [18,19]. We employed the BEAGLE 3.2 high-performance computational library to speed up the required likelihood calculations [20]. We assumed an exponential growth model as the tree-generative prior, an uncorrelated relaxed molecular clock model with underlying lognormal distribution, a general time-reversible substitution model with estimated base frequencies and a discretized gamma distribution to model among-site rate heterogeneity [21,22,23]. We further used an asymmetric location substitution model with Bayesian stochastic search variable selection (BSSVS) to simultaneously determine which migration rates are zero depending on the evidence in the data and to efficiently infer the ancestral locations [19]. We assumed default priors on all parameters as defined in the BEAUti interface to BEAST, but added a calibration prior to each root node corresponding to the inferred age of these nodes in our preceding Nextstrain analysis [18]. These analyses ran for a total of 750 million iterations, with the Markov chains being sampled every 10,000th iteration, in order to reach an effective sample size (ESS) for all relevant parameters of at least 200, as determined by Tracer 1.7 [24]. We used TreeAnnotator to construct maximum clade credibility (MCC) trees for each subtree [18]. Finally, we used the baltic Python library to visualize the clades of interest [25].

### 2.5. Serology

Serum samples were collected on day 15 after arrival. Anti-SARS-CoV-2 antibodies were measured on Abbott Architect (Abbott, Lake Forest Illinois) with the chemiluminescence IgG (anti-nucleocapsid or anti-N) and IgG II Quant (anti-spike or anti-S) assays using the manufacturer’s cut-offs for positivity of 1.4 S/CO and 50 AU/mL, respectively. In addition, we defined a grey zone for IgG anti-N results between 0.80 and 1.4 S/CO.

## 3. Results

### 3.1. Epidemiological Situation

**Figure 1:**
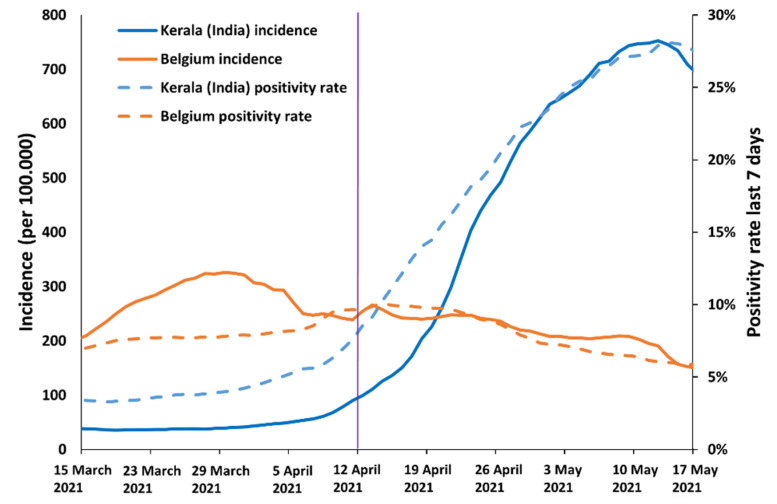
Incidence and 7-day positivity rate in Kerala (India) and Belgium. The date of arrival in Belgium is marked with a vertical purple line.

The first confirmed case of an infection with the SARS-CoV-2 Delta variant in Belgium was detected on 4 April 2021. The first large cluster of infections in Belgium with the Delta variant occurred in a group of Indian nursing students who travelled to Belgium. This group of 41 students departed to Belgium (via New Delhi and Paris) on 11 April 2021 from the state of Kerala (India) at a time of decreasing virus circulation in Belgium and increasing viral circulation in Kerala. Belgian authorities considered India as a high-risk country for travel at that moment because of this upward epidemiological trend in confirmed cases (a metric that is known to lag behind true infection incidence) (Figure 1) and also because of the knowledge that a new (potentially dangerous) SARS-CoV-2 variant was spreading there. The 7-day test positivity rate in Kerala increased from 5.1% on 4 April to 7.6% on 11 April and 14.0% on 18 April with a peak of 28.1% on 13 May (Figure 1) [26,27].

### 3.2. Travel to Belgium

On 11 April, 41 students flew from Kochi (*n* = 33) and Trivandrum (*n* = 8) (both in the state of Kerala, India), via New Delhi (India) to Paris–Charles de Gaulle (CDG) (France). All students had a negative RT-PCR (*n* = 37) or antigen test (*n* = 4) before departure from New Delhi. On 12 April, upon arrival in Paris all students had a negative rapid antigen test. The students subsequently traveled by dedicated coach to their residences in two Belgian cities (Aalst and Leuven). At the date of departure, the Delta variant was responsible for 63.2% and 0.33% of COVID-19 infections in India and Belgium, respectively.

During the flights, the students wore personal protective equipment (face shield and face mask). They wore a face mask while waiting for 3 to 4 h at the airport in New Delhi and 3 to 4 h at the airport in Paris, and during the bus trip to Belgium. Upon arrival they were quarantined in eight different residences. The students were not allowed to leave their house (food and basic necessities were provided); however, they were not required to take protective measures within their respective house. Travel policy in Belgium at the time required that arriving travelers from high-risk countries had a negative PCR or antigen test before departure and were obliged to quarantine for 10 days. Quarantine could only be lifted if the travelers had one or more negative RT-PCR tests, with the last result at least 7 days after arrival. This policy is in line with recommendations from the CDC, which advises a 14-day quarantine without testing or a shortened quarantine of 10 days when RT-PCR testing is performed [28].

These 41 students were joined by two students who departed from Malta (after a negative PCR test) and flew directly to Charleroi, Belgium. After a negative antigen test in Charleroi, these two students continued their journey by taxi to their planned accommodation. The demographic and clinical information of the students is summarized in Table 1.

### 3.3. Reconstructed Time Series of the Infections

**Figure 2:**
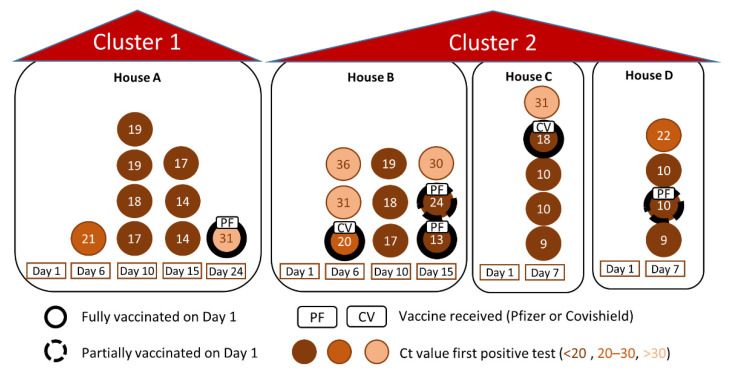
Epicurve showing positive cases per day and per house.

During the first 7 days of quarantine, all students remained asymptomatic, but 13 students nevertheless tested positive at the end of this first week of quarantine (four students on day 6 and nine students on day 7 after arrival). These 13 cases were distributed between five of the eight residences (letters A, B, C, D and G, see Figure 2 and Supplementary Table S1), which housed a total of 32 students.

One student with a low viral load (<10^3^ copies/mL) was immediately transferred from a house where the other students tested negative (house G) to a house where several students had tested positive (house C) in order to protect the other residents of house G from infection. The 14 students in houses A, B, C and D who tested negative during the first testing round remained in their respective residences and eventually all tested positive during follow up. Seven of the 14 students tested positive on day 10, six students became positive on day 15 and one on day 24. By day 24, all 27 students quarantined in the four “positive” houses (A, B, C and D) had developed an infection, while the other 16 students, quarantined in the “negative” houses (letters E, F, G and H), remained negative for the entire follow-up period (See Figure 2 and Supplementary Table S1). Four out of the five students fully vaccinated upon arrival (≥2 weeks after second dose) and two out of three partially vaccinated students (only one dose) developed an infection during follow-up. None of the students required hospital admission. Quarantine ended on day 18 for those residences where nobody tested positive (E, F, G and H; Supplementary Table S1), while the quarantine was extended for another 7 days (25 days total) for the students in the other residences (A, B, C and D). On day 15 and 24, subgenomic RNA (sgRNA), a marker for actively replicating virus, was determined for students who had tested positive during the follow-up. On day 15, 10 out of 11 students tested positive. On day 24, all students tested negative for sgRNA (including the one student who then tested PCR positive for the first time). Quarantine was lifted for all students on day 25, except for the fully vaccinated student who tested positive on day 24. This student was asked to remain in quarantine for another five days until he tested negative with RT-PCR (29 days total). This student was considered to have a low risk of transmission because of three reasons: (1) negative sgRNA (2) pre-existing immunity (both natural infection and vaccine-induced, as indicated by positive anti-nucleocapsid antibodies before his first positive PCR) and (3) a weakly positive PCR result (Ct 31.6, <10^3^ copies/mL). His positive result therefore did not trigger an extended quarantine for all his household members, and his own quarantine was shortened to five days.

### 3.4. Phylogeographic Inference Results

**Figure 3:**
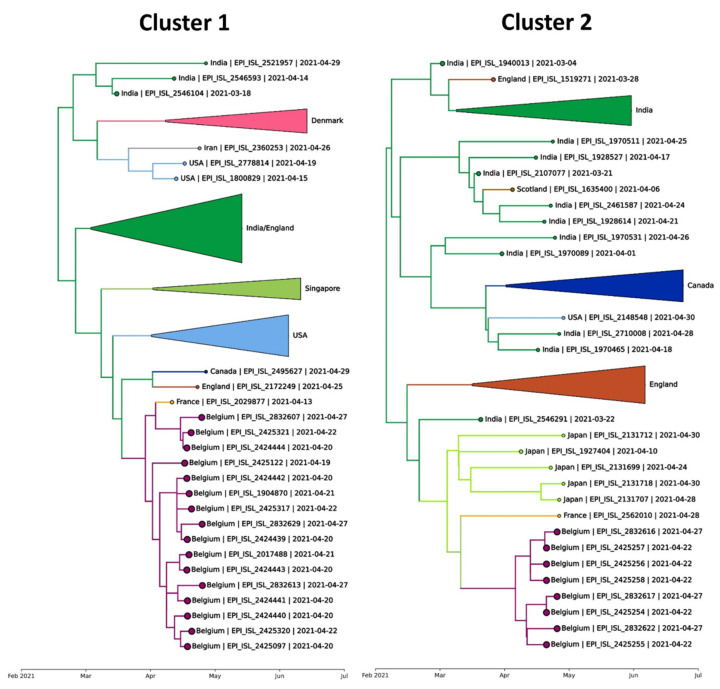
Infections among the Indian students show two separate clusters in the overall phylogeny. A single large-scale phylogeny was constructed in which we detect a first cluster (**left**) with 16 of the Indian students and a second cluster (**right**) with 8 of the Indian students. While both clusters included a French person sampled at Paris Charles de Gaulle airport, based on the available data, the infections among the Indian students are inferred to have originated from an Indian (Cluster 1) and from a Singaporean or Indian (Cluster 2) source.

Two separate clusters of Delta variant strains were identified among the infected students (See Figure 3).

The fact that these two clusters were only distantly related indicate that there were two separate introductions of SARS-CoV-2 in the group (Supplementary Figures S1 and S2). There was no mixing of the two clusters within the different houses, as the students in residence A were infected with cluster 1, and the students in residences B, C and D were infected with cluster 2. Strains from cluster 1 were related to a single genome from France, taken during screening at Paris-CDG after return from India on 13 April 2021, one day after the students spent time there. Strains from cluster 2 were also related to a (different) single genome from France, also taken during screening at Paris-CDG but only on 28 April 2021 (Figure 3).

### 3.5. Contact Tracing

Altogether, contact tracing could identify 22 contacts during and after the bus travel from Paris to Belgium. All tested negative, suggesting that transmission to persons outside the group was prevented. No further genomes related to these clusters have been detected in the ensuing three months of nationwide genomic surveillance (Figure 3), confirming that onward transmission into the community was effectively prevented.

There was no apparent link between the seats on the airplane or bus and the strain with which the students were infected. This suggests that the contamination did not occur while the students were seated in the airplane or bus, and may suggest that these travel-related clusters originated just before or just after the journey, including potentially in crowded testing centers.

## 4. Discussion

We describe how a combination of pre-travel testing, quarantine upon arrival and testing upon quarantine exit contained the infections within a group of Indian travelers and prevented onward local transmission of the SARS-CoV-2 Delta variant, confirming the ‘real world’ effectiveness of this strategy. In this group of students, two distinct clusters emerged despite pre- and post-journey testing. It is still somewhat unclear where and how the students were initially infected. The average incubation time of SARS-CoV-2 is around 6 days [29,30], and probably 4 days for the Delta variant [31], although incubation periods up to 14 and up to 18 days has been reported in <5% and <1% of individuals, respectively [32]. This implies that the students could have been infected in India, on the airplane, in Paris or on the coach to Belgium. The vaccination status did not seem to decrease the risk of infection or viral load. The Delta variant has been reported to partially evade immunity induced by vaccination and natural infection, making breakthrough infections more likely [33]. Despite a lower efficacy against infection by the Delta variant, the vaccines largely retain the ability to protect against severe forms of COVID-19 (hospitalization and death) in immunocompetent hosts [34]. In conclusion, preventing the spread of VOCs associated with international travels requires a holistic approach, which includes repeated testing and quarantine for all travelers regardless of vaccination status, particularly when originating from areas experiencing a surge of infections with a variant of concern.

## Figures and Tables

**Table 1:** Demographics.

	≥1 Positive PCR	Not Infected
Number of individuals	27	16
Median age (years [range])	32.5 (23–39)	34 (29–42)
Male/Female (% men)	4/23 (14.4%)	3/13 (23.1%)
Severity (%)		
Asymptomatic	3 (11.1%)	/
Mild	24 (88.9%)	/
Vaccination status		
Fully vaccinated	4	1
Partially vaccinated	2	1
Unvaccinated	21	14

## Data Availability

The data presented in this study are available on request from the corresponding author.

## Supplementary Materials

The following supporting information can be downloaded at: https://www.mdpi.com/article/10.3390/v14061198/s1, Figure S1: All of the mutations/deletions (compared to Wuhan wild type) that characterize each of the clusters, along with their associated phylogeny, Figure S2: Overall SARS-CoV-2 phylogeny, with the sequences from this manuscript highlighted as maroon points, Table S1: RT-PCR and IgG anti-SARS-CoV-2 results (Day 1 = 12 April 2021 (Day of arrival in Belgium)), Table S2: GISAID acknowledgements, Supplementary document: COG-Belgium consortium members and affiliations.

## Appendix A

**Table A1:** GISAID IDs.

EPI_ISL_2832616	EPI_ISL_2832622	EPI_ISL_2832617	EPI_ISL_2832629
EPI_ISL_2832607	EPI_ISL_2832613	EPI_ISL_1904870	EPI_ISL_2425321
EPI_ISL_2017488	EPI_ISL_2425317	EPI_ISL_2425320	EPI_ISL_2425097
EPI_ISL_2424442	EPI_ISL_2424444	EPI_ISL_2424439	EPI_ISL_2425122
EPI_ISL_2424443	EPI_ISL_2424441	EPI_ISL_2424440	EPI_ISL_2425256
EPI_ISL_2425254	EPI_ISL_2425258	EPI_ISL_2425255	EPI_ISL_2425257
