# Supplementary material for: Two Separate Clusters of SARS-CoV-2 Delta Variant Infections in a Group of 41 Students Travelling from India: An Illustration of the Need for Rigorous Testing and Quarantine
Source: Viruses. 2022 May 31;14(6):1198. doi: 10.3390/v14061198 (PMC9229483; doi:10.3390/v14061198)
Supplement: Supplementary file 1 [file viruses-14-01198-s001.zip › Supplementary_Table_S2.pdf]

We gratefully acknowledge the following Authors from the Originating laboratories responsible for obtaining the specimens, as well as the Submitting laboratories where the genome data were generated and shared via GISAID, on which this research is based.

All Submitters of data may be contacted directly via www.gisaid.org

Authors are sorted alphabetically.

Accession ID	Originating Laboratory	Submitting Laboratory	Authors
EPI_ISL_1167086	"Dr. Andrija Stampar" Teaching Institute of Public Health, Department of Clinical Microbiology	Istituto di Genomica Applicata	Davide Scaglione; Eleonora Paparelli; Fedrica Cattonaro; Gabriele Magris; Irena Jurman; Jasmina Vranes; Michele Morgante; Slobodanka Radovic; Vera Vendramin
EPI_ISL_1394786	"InMedica"	Lithuanian University of Health Sciences Hospital, Department of Genetics and Molecular Medicine	Astra Vitkauskiene; Darius Cereskevicius; Inga Nasvytiene; Mantas Sarauskas; Marius Sukys; Rasa Ugenskiene; Renaldas Jurkevicius; Rima Vainoriene; Zivile Zemeckiene
EPI_ISL_2162162	"National Center of Expertise" CSEC MH RK in Almaty city	Reference laboratory for the control of viral infections	Aidar Usserbayev; Aknur Mutaliyeva; Andrey Komissarov; Artem Fadeev; Azamat Kenessov; Bekzhan Maikotov; Gaukhar Nussupbayeva; Madina Tleubergenova; Maria Pisareva; Nazym Tleumbetova
EPI_ISL_2162147	"National Center of Expertise" CSEC MH RK in West Kazakhstan Region	Reference laboratory for the control of viral infections	Aidar Usserbayev; Aknur Mutaliyeva; Andrey Komissarov; Artem Fadeev; Azamat Kenessov; Bekzhan Maikotov; Gaukhar Nussupbayeva; Madina Tleubergenova; Maria Pisareva; Nazym Tleumbetova
EPI_ISL_1495757	3. Medizinische Abteilung, Hanusch Krankenhaus	Bergthaler laboratory, CeMM Research Center for Molecular Medicine of the Austrian Academy of Sciences	Andreas Bergthaler; Anna Schedl; Bekir Erguner; Benedikt Agerer; Christoph Bock; Fabian Amman; Jan Laine; Lukas Endler; Maelle Le Moing; Martin Senekowitsch; Michael Schuster; Petr Triska; Thomas Penz
EPI_ISL_1940026, EPI_ISL_1940036	A D HALLI	inStem NCBS - INSACOG	Uma Ramakrishnan Dasaradhi Palakodeti Aswin SaiNarain
EPI_ISL_2721281, EPI_ISL_2721283	A.S.L. CN2	Fondazione del Piemonte per l'Oncologia IRCCS	Antonino Sottile; Giorgio Giardina; Paola Marino; Silvia Brossa
EPI_ISL_2721288	A.S.L. TO4	Fondazione del Piemonte per l'Oncologia IRCCS	Antonino Sottile; Giorgio Giardina; Paola Marino; Silvia Brossa
EPI_ISL_498524, EPI_ISL_498534, EPI_ISL_498547, EPI_ISL_735503, EPI_ISL_1170946, EPI_ISL_1170947, EPI_ISL_1208400, EPI_ISL_1208401, EPI_ISL_1208402, EPI_ISL_1807318			
see above	ACT Pathology	Schwessinger Lab	Ashley Jones; Benjamin Schwessinger; Craig Kennedy; Karina Kennedy; Kevin Murray; Megan McDonald; Ming-Dao Chia; Robert Lanfear; Robyn N Hall
EPI_ISL_2379356	AD HALLI	INSACOG-KA, NIMHANS	Ananthapadmanabha Kotambail; Anita S Desai; Anson Kunjumon George; Chetan G K; Chitra Pattabiraman; Darshan Sreenivas; Gautham Arunachal Udupi; Pramada Prasad; V Ravi
EPI_ISL_1940092	AD HALLI	inStem NCBS - INSACOG	Uma Ramakrishnan Dasaradhi Palakodeti Aswin SaiNarain
EPI_ISL_2621212	ADILAB	Universidad Nacional de Colombia - Laboratorio Genómico One Health	Andres F. Cardona-Rios; Carlos Franco-Muñoz; Carolina Muñoz-Arango; Celeny Ortiz; Daniel O. Maldonado-Perez; Diego A. Álvarez-Díaz; Hector Alejandro Ruiz-Moreno; Idabely Betancur Ortiz; Jorge E. Osorio; Juan P. Hernandez-Ortiz; Karl A Ciuoderis; Katherine Laiton-Donato; Laura Silvana Perez; Lina M. Hurtado; Marcela Mercado-Reyes; Maria Angélica Maya; Maria Stella López; Rita Almanza Payares; Sandra Ines Cano; Simón Villegas Velásquez
EPI_ISL_2617039, EPI_ISL_2617048, EPI_ISL_2617056	AFRICA_CDC - Angola (Ship 12)	CERI, Centre for Epidemic Response and Innvoation, Stellenbosch University and KRISP, KZN Research Innovation and Sequencing Platform, UKZN.	Afonso P; David K; Emmanuel SJ; Freitas RH; Giandhari J; Inglês L; Lutucuta S; Miranda J; Morais J; Mufinda M; Naidoo Y; Neto Z; Paulo A Carralero RR Paixão JP; Pereira A; Pillay S; Tegally H; Wilkinson E; de Oliveira T
EPI_ISL_2379322, EPI_ISL_2379323, EPI_ISL_2379324, EPI_ISL_2379325, EPI_ISL_2379326, EPI_ISL_2379327, EPI_ISL_2379328, EPI_ISL_2379329, EPI_ISL_2379330			
see above	AGRAHARA PHC	INSACOG-KA, NIMHANS	Ananthapadmanabha Kotambail; Anita S Desai; Anson Kunjumon George; Chetan G K; Chitra Pattabiraman; Darshan Sreenivas; Gautham Arunachal Udupi; Pramada Prasad; V Ravi
EPI_ISL_1663507, EPI_ISL_1663516, EPI_ISL_1663522, EPI_ISL_1663523	AIIMS, Patna	Institute of Life Sciences - INSACOG	Ajay Parida; Amol M. Kanampalliwar; Arup Ghosh; Atimukta Jha; INSACOG Consortium; Punit Prasad; Rajeeb Swain; Rupesh Dash; Safal Walia; Shifu Aggarwal; Sunil K. Raghav
EPI_ISL_2231590, EPI_ISL_2231597, EPI_ISL_2231598, EPI_ISL_2231599, EPI_ISL_2231601, EPI_ISL_2231605, EPI_ISL_2231607, EPI_ISL_2231608, EPI_ISL_2231609, EPI_ISL_2231611, EPI_ISL_2231612, EPI_ISL_2231614, EPI_ISL_2231615, EPI_ISL_2231616, EPI_ISL_2231627, EPI_ISL_2231628, EPI_ISL_2231632, EPI_ISL_2231633, EPI_ISL_2231634, EPI_ISL_2231635, EPI_ISL_2231636, EPI_ISL_2231639, EPI_ISL_2231640, EPI_ISL_2231641, EPI_ISL_2231642, EPI_ISL_2231645, EPI_ISL_2231650, EPI_ISL_2231651, EPI_ISL_2231652, EPI_ISL_2231654, EPI_ISL_2231656, EPI_ISL_2231659, EPI_ISL_2231660, EPI_ISL_2231665, EPI_ISL_2231666, EPI_ISL_2231667, EPI_ISL_2231669, EPI_ISL_2231672, EPI_ISL_2231677, EPI_ISL_2231678, EPI_ISL_2231680, EPI_ISL_2231681, EPI_ISL_2231684, EPI_ISL_2231685, EPI_ISL_2231687, EPI_ISL_2231690, EPI_ISL_2231691, EPI_ISL_2231695, EPI_ISL_2231696, EPI_ISL_2231697, EPI_ISL_2231701, EPI_ISL_2231702, EPI_ISL_2231703, EPI_ISL_2231706, EPI_ISL_2231707, EPI_ISL_2231711, EPI_ISL_2231712, EPI_ISL_2231713, EPI_ISL_2231716, EPI_ISL_2231721, EPI_ISL_2231726, EPI_ISL_2231728, EPI_ISL_2231730, EPI_ISL_2231732, EPI_ISL_2231738, EPI_ISL_2231739, EPI_ISL_2231746, EPI_ISL_2231747, EPI_ISL_2231748, EPI_ISL_2231750, EPI_ISL_2231752, EPI_ISL_2231754, EPI_ISL_2231756, EPI_ISL_2231757, EPI_ISL_2231759, EPI_ISL_2231761, EPI_ISL_2231767, EPI_ISL_2231769, EPI_ISL_2231772, EPI_ISL_2231773, EPI_ISL_2231777, EPI_ISL_2231779, EPI_ISL_2231782, EPI_ISL_2231784, EPI_ISL_2231794, EPI_ISL_2231797, EPI_ISL_2231798, EPI_ISL_2231799, EPI_ISL_2231808, EPI_ISL_2231809, EPI_ISL_2231810, EPI_ISL_2231812, EPI_ISL_2231821, EPI_ISL_2231822, EPI_ISL_2231823, EPI_ISL_2231830, EPI_ISL_2231831, EPI_ISL_2231832, EPI_ISL_2231841, EPI_ISL_2231848, EPI_ISL_2231849, EPI_ISL_2231850, EPI_ISL_2231853, EPI_ISL_2231854, EPI_ISL_2231855, EPI_ISL_2231864, EPI_ISL_2231866, EPI_ISL_2231867, EPI_ISL_2231869, EPI_ISL_2231872, EPI_ISL_2323024, EPI_ISL_2373211, EPI_ISL_2373242, EPI_ISL_2441111, EPI_ISL_2441212, EPI_ISL_2660722, EPI_ISL_2774724, EPI_ISL_2774725, EPI_ISL_2774792, EPI_ISL_2775381, EPI_ISL_2775382, EPI_ISL_2775383, EPI_ISL_2775385, EPI_ISL_2775386, EPI_ISL_2775387, EPI_ISL_2775389			
see above	AP SSO	CSIR-Centre for Cellular and Molecular Biology-INSACOG	Amareshwar Vodapalli; Ara Sreenivas; Archana Bharadwaj Siva; B Himasri; Blessy B John; Divya Tej Sowpati; Jandhyala Sai Krishna; Karthik Bharadwaj Tallapaka; Lamuk Zaveri; Onkar Kulkarni; Payel Mukherjee; Priya Nurkurthy; Rakesh K Mishra; Sharath Chandra Thota; Shreekant Verma; Sofia Banu; Sumedha Avadhanula; Tulasi Nagabandi; Valli Nagalakshmi Undamatla; Vidhyadhari Methuku; Viswagithe S L
EPI_ISL_682250	AREA DE SALUD ESCAZU (COOPESANA)	Inciensa, Instituto Costarricense de Investigación y Enseñanza en Nutrición y Salud	Adriana Godinez & Melany Calderon; Claudio Soto-Garita; Estela Cordero; Francisco Duarte; Hebleen Porras
EPI_ISL_2502736	AREA DE SALUD GUATUSO	Inciensa, Instituto Costarricense de InvestigaciOn y Enseñanza en NutriciOn y Salud	Adriana Godínez; Claudio Soto-Garita; Estela Cordero; Francisco Duarte; Hebleen Porras; Jose Luis Vargas; Joselyn Prado & Francisco ChacOn-Valverde; Mariela Gutierrez; Melany CalderOn
EPI_ISL_1712388	AREA DE SALUD LOS SANTOS	Inciensa, Instituto Costarricense de Investigación y Enseñanza en Nutrición y Salud	Adriana Godínez; Claudio Soto-Garita; Estela Cordero; Francisco Duarte; Hebleen Porras; Joselyn Prado & Mónica Charpentier-Artavia; José Luis Vargas; Mariela Gutiérrez; Melany Calderón
EPI_ISL_2502732	AREA DE SALUD OREAMUNO-PACAYAS-TIERRA BLANCA	Inciensa, Instituto Costarricense de InvestigaciOn y Enseñanza en NutriciOn y Salud	Adriana Godínez; Claudio Soto-Garita; Estela Cordero; Francisco Duarte; Hebleen Porras; Jose Luis Vargas; Joselyn Prado & Carolina Loría-Acosta; Mariela Gutierrez; Melany CalderOn
EPI_ISL_2502747	AREA DE SALUD PARAISO-CERVANTES	Inciensa, Instituto Costarricense de InvestigaciOn y Enseñanza en NutriciOn y Salud	Adriana Godínez; Claudio Soto-Garita; Estela Cordero; Francisco Duarte; Hebleen Porras; Jose Luis Vargas; Mariela Gutierrez & Joselyn Prado; Melany CalderOn
EPI_ISL_2103393	AREA DE SALUD PAVAS (COOPESALUD)	Inciensa, Instituto Costarricense de Investigación y Enseñanza en Nutrición y Salud	Adriana Godínez; Caterina Guzmán; Claudio Soto-Garita; Estela Cordero; Francisco Duarte; Hebleen Porras; Joselyn Prado; José Luis Vargas; Mariela Gutiérrez; Melany Calderón; Nazareth Ruiz & Mariel López
EPI_ISL_2272993	AREA DE SALUD SANTO DOMINGO	Inciensa, Instituto Costarricense de Investigación y Enseñanza en Nutrición y Salud	Adriana Godínez; Claudio Soto-Garita; Estela Cordero; Francisco Duarte; Hebleen Porras; Joselyn Prado & Margarita Lee-Lui; José Luis Vargas; Mariela Gutiérrez; Melany Calderón
EPI_ISL_2502737, EPI_ISL_2502744	AREA DE SALUD SIQUIRRES	Inciensa, Instituto Costarricense de InvestigaciOn y Enseñanza en NutriciOn y Salud	Adriana Godínez; Claudio Soto-Garita; Estela Cordero; Francisco Duarte; Hebleen Porras; Jose Luis Vargas; Joselyn Prado & Ileana Cháves-Peraza; Joselyn Prado & Karla LOpez; Mariela Gutierrez; Melany CalderOn
EPI_ISL_2272958	AREA DE SALUD SIQUIRRES	Inciensa, Instituto Costarricense de Investigación y Enseñanza en Nutrición y Salud	Adriana Godínez; Claudio Soto-Garita; Estela Cordero; Francisco Duarte; Hebleen Porras; Joselyn Prado & Ileana Chaves-Peraza; José Luis Vargas; Mariela Gutiérrez; Melany Calderón
EPI_ISL_1811240	AS Alajuela Central	Inciensa, Instituto Costarricense de Investigación y Enseñanza en Nutrición y Salud	Pérez-Corrales C & Zuñiga-Carvajal P
EPI_ISL_1811237	AS Santa Barbara	Inciensa, Instituto Costarricense de Investigación y Enseñanza en Nutrición y Salud	Pérez-Corrales C & Aguilar-Monge R
EPI_ISL_2379394	ASHOKPURAM	INSACOG-KA, NIMHANS	Ananthapadmanabha Kotambail; Anita S Desai; Anson Kunjumon George; Chetan G K; Chitra Pattabiraman; Darshan Sreenivas; Gautham Arunachal Udupi; Pramada Prasad; V Ravi
EPI_ISL_1085197, EPI_ISL_1229737, EPI_ISL_2716552	ASL Napoli 1 Centro	AMES Centro Polidiagnostico Strumentale S.r.l.	"Giovanni Savarese; Antonella Di Carlo; Antonio Fico"; Eloisa Evangelista; Luigi D'Amore; Luisa Circelli; Maurizio D'Amora; Maurizio D'Amora Antonio Fico"; Monica Ianniello; Nadia Petrillo; Raffaella Ruggiero; Roberto Sirica
EPI_ISL_2790551	AZ Jan Portaels	Imelda Ziekenhuis	Dagmar Obbels; Hanne Valgaeren; Johan Frans
EPI_ISL_2434148, EPI_ISL_2434150, EPI_ISL_2434159, EPI_ISL_2434163, EPI_ISL_2567003, EPI_ISL_2652162, EPI_ISL_2652247			
see above	AZ Klina	AZ Klina	Carl Vael - Lynsey Berckmans
EPI_ISL_2686393	AZ Sint-Jan Brugge-Oostende AV	AZ Sint Jan	Jorn Hellemans; Laurien Hoornaert; Marie Madeleine Chabert-Consen; Marijke Reynders; Patrick Descheemaeker; Thomas Van Landschoot
EPI_ISL_2709603	AZ Sint-Lucas Gent	AZ Sint-Lucas Gent	Charlotte Verfaillie; Elke Vanlaere; Jos Van Acker; Klara De Rauw
EPI_ISL_1972127, EPI_ISL_1972129	AZ Zeno	AZ SINT-JAN BRUGGE	Jorn Hellemans; Laurien Hoornaert; Marijke Reynders; Patrick Descheemaeker; Thomas Van Landschoot
EPI_ISL_2235305	AZ Zeno Knokke-Blankenberge	AZ SINT-JAN BRUGGE	Jorn Hellemans; Laurien Hoornaert; Marie Madeleine Chabert-Consen; Marijke Reynders; Patrick Descheemaeker; Thomas Van Landschoot
EPI_ISL_2376354, EPI_ISL_2725488, EPI_ISL_2725489, EPI_ISL_2725491, EPI_ISL_2725501, EPI_ISL_2725506, EPI_ISL_2725507, EPI_ISL_2725510, EPI_ISL_2727158, EPI_ISL_2727163, EPI_ISL_2727172, EPI_ISL_2727323, EPI_ISL_2790095, EPI_ISL_2790097, EPI_ISL_2790098, EPI_ISL_2790099, EPI_ISL_2790100, EPI_ISL_2790101, EPI_ISL_2790103, EPI_ISL_2790104, EPI_ISL_2790105, EPI_ISL_2790109, EPI_ISL_2790117, EPI_ISL_2790121, EPI_ISL_2790126, EPI_ISL_2790127, EPI_ISL_2790131			
see above	AZDelta	AZ Delta Medical Laboratories in Roeselare, Belgium	Dieter De Smet; Geert Martens; Merijn Vanhee; on behalf of AZ Delta COVID-19 Genomics core (member of Genomic surveillance of SARS-CoV-2 in Belgium network)
EPI_ISL_1109919, EPI_ISL_1390762	AZDelta	AZDelta	Dieter De Smet; Geert Martens
EPI_ISL_890187	Academic Hospital of Gadjah Mada University (RSA UGM)	Genetics Working Group (Pokja Genetik) Faculty of Medicine, Public Health and Nursing Universitas Gadjah Mada (FK-KMK UGM); Disease Investigation Center Wates Ministry of Agriculture Indonesia; Department of Microbiology FK-KMK UGM; Laboratorium Diagnostik Yayasan Tahija	Afiahayati; Dwi AA Nugrahaningsih; Edwin W. Daniwijaya; Eggi Arguni; Endah Supriyati; Gunadi; Hendra Wibawa; Kristy Iskandar; Ludhang P. Rizki; Marcellus; Mohamad S. Hakim; Nungki Anggorowati; Siswanto; Titik Nuryastuti; Tri Wibawa; Untung Riawan; William Widitjiarso

		World Mosquito Program (WMP) Yogyakarta Center for Tropical Medicine FK-KMK UGM; Integrated Research center FK-KMK UGM; Department of Computer Science and Electronics FMIPA UGM	
EPI_ISL_2360250, EPI_ISL_2360251	Afzalipoor Hospital	National Influenza Center	A Nejati; F Ajaminejad and T Mokhtari Azad; J Yavarian; K Sadeghi; N Ghavvami; NZ Shafiei Jandaghi; V Salimi
EPI_ISL_2162962, EPI_ISL_2164068, EPI_ISL_2165343, EPI_ISL_2170421, EPI_ISL_2170475, EPI_ISL_2170576	Alberta Precision Labs (APL)	Public Health Agency of Canada (PHAC) National Microbiology Laboratory	Buss; Croxen M; Deo A; Dieu P; E; Ferrato C; Gill K; Khan F; Koleva P; Li V; Lloyd C; Lynch T; Ma R; Murphy S; Pabbaraju K; Shokoples S; Thayer J; Tipples G; Whitehouse M; Wong A; Yu C; Zelyas N
EPI_ISL_2272778, EPI_ISL_2272779, EPI_ISL_2358230, EPI_ISL_2422123, EPI_ISL_2545627, EPI_ISL_2545629, EPI_ISL_2626920, EPI_ISL_2714839, EPI_ISL_2714842, EPI_ISL_2714846			
see above	Algemeen Medisch Labo	Labo Klinische Biologie, UZA	Basil Britto Xavier; Christine Lammens; Herman Goossens; Ines Verbesselt; Jasmine Coppens; Kathleen Holemans; Marie Le Mercier; Veerle Matheeussen
EPI_ISL_2100679, EPI_ISL_2100680, EPI_ISL_2100681, EPI_ISL_2100682, EPI_ISL_2100687, EPI_ISL_2100688, EPI_ISL_2100689, EPI_ISL_2100690, EPI_ISL_2100691, EPI_ISL_2100692, EPI_ISL_2100694, EPI_ISL_2100695, EPI_ISL_2100696, EPI_ISL_2100698, EPI_ISL_2100699, EPI_ISL_2105583, EPI_ISL_2105584, EPI_ISL_2105585			
see above	All India Institute of Medical Sciences, Ansari Nagar Delhi	CSIR-Institute of Genomics and Integrative Biology	Animesh Ray; Asangala Kamai; Ashwin Varadarajan; Ayush Goel; Bharathram Uppilli*; Devasish Desai; Manish Kumar; Mohammed Ahmed; Mohammed Faruq; Naveet Wig; Pooja Sharma*; Rajesh Pandey; Sarafaraz Alam; Saruchi Wadhwa; Satish Swain; Sheeba Saifi; Sushma Rajpoot; Umang Arora
EPI_ISL_2426190, EPI_ISL_2426191, EPI_ISL_2426192, EPI_ISL_2426205, EPI_ISL_2426206, EPI_ISL_2426210, EPI_ISL_2426214, EPI_ISL_2426216, EPI_ISL_2426217, EPI_ISL_2426222, EPI_ISL_2426223, EPI_ISL_2426224, EPI_ISL_2426226, EPI_ISL_2426227, EPI_ISL_2426230, EPI_ISL_2426231, EPI_ISL_2426232, EPI_ISL_2426234, EPI_ISL_2426235, EPI_ISL_2426239, EPI_ISL_2426242, EPI_ISL_2426243, EPI_ISL_2426245, EPI_ISL_2426248, EPI_ISL_2426249, EPI_ISL_2426251, EPI_ISL_2426252			
see above	All India Institute of Medical Sciences, Delhi	CSIR Institute of Genomics and Integrative Biology	Abhinav Jain; Afra Shamnath; Anjali Bajaj; Arvinden VR; Bani Jolly; COVID CBNAAT CORE GROUP; Kiran Bala; Mercy Rophina; Mohamed Imran; Mohit Kumar Divakar; Nayer Jamshed; Praveen Aggarwal; Rahul C. Bhoyar; Rama Chaudhry; Randeep Guleria; Ritu Gupta; Sridhar Sivasubbu; Subrata Sinha; Urvashi B Singh; Vigneshwar Senthivel; Vinod Scaria
EPI_ISL_2686110, EPI_ISL_2686137	Amazon Dx, AUV2 Laboratory	Amazon Dx, AUV2 Laboratory	Luke Meredith
EPI_ISL_770008	Area De Salud Corredores	Inciensa, Instituto Costarricense de Investigación y Enseñanza en Nutrición y Salud	Adriana Godínez; Claudio Soto-Garita; Estela Cordero; Francisco Duarte; Hebleen Porras; Melany Calderón & Mariel López
EPI_ISL_512666	Area De Salud La Cruz	Inciensa, Instituto Costarricense de Investigación y Enseñanza en Nutrición y Salud	Adriana Godinez & Melany Calderon; Claudio Soto-Garita; Estela Cordero; Francisco Duarte; Hebleen Porras
EPI_ISL_500697, EPI_ISL_509522, EPI_ISL_527013, EPI_ISL_527014, EPI_ISL_527018, EPI_ISL_1205815, EPI_ISL_1240991			
see above	Area of Virology, Serology and Virology Division (SAViD), New South Wales Health Pathology Randwick	Area of Virology, Serology and Virology Division (SAViD), New South Wales Health Pathology Randwick	Au, J.; Bull, R.; Deveson, I.; Foster, C.; Rawlinson, W.; Ruiz Silva, M.; Van Hal, S.
EPI_ISL_707899, EPI_ISL_1098798, EPI_ISL_1098799, EPI_ISL_1121974, EPI_ISL_1184504, EPI_ISL_1293049, EPI_ISL_1406433, EPI_ISL_1424505, EPI_ISL_1494721, EPI_ISL_1615595, EPI_ISL_1615596, EPI_ISL_1615597, EPI_ISL_1615598, EPI_ISL_1633348, EPI_ISL_1633353, EPI_ISL_1672867, EPI_ISL_1911182, EPI_ISL_1911185, EPI_ISL_1911187, EPI_ISL_1911189, EPI_ISL_1911190, EPI_ISL_1911191, EPI_ISL_2404984, EPI_ISL_2404990			
see above	Area of Virology, Serology and Virology Division (SAViD), New South Wales Health Pathology Randwick	Virology Research Laboratory; Area of Virology, Serology and Virology Division (SAViD), New South Wales Health Pathology Randwick	Au, J.; Bull, R.; Deveson, I.; Foster, C.; Rawlinson, W.; Ruiz Silva, M.; Van Hal, S.; Wong, M.
EPI_ISL_1593888, EPI_ISL_1595718	Armed Forces Institute of Pathology (AFIP), Dhaka Cantonment	Genomic Research Lab, BCSIR	Abu Sayeed Mohammad Mahmud; Barna Goswami; Eshrar Osman; Iffat Jahan; Md. Ahasan Habib; Md. Kamrul Islam; Md. Murshed Hasan Sarkar; Md. Saddam Hossain; Md. Salim Khan; Mohammad Mizanur Rahman; Mohammad Mohi Uddin; Mohammad Samir Uzzaman; Shahina Akter; Susane Giti; Tanjina Akhter Banu
EPI_ISL_2304055, EPI_ISL_2304086, EPI_ISL_2304087, EPI_ISL_2304088, EPI_ISL_2304089, EPI_ISL_2304092, EPI_ISL_2304093, EPI_ISL_2304094, EPI_ISL_2304095, EPI_ISL_2304096, EPI_ISL_2304097, EPI_ISL_2304098, EPI_ISL_2304100, EPI_ISL_2304101, EPI_ISL_2304108, EPI_ISL_2304109, EPI_ISL_2304110, EPI_ISL_2304112, EPI_ISL_2304113, EPI_ISL_2304114, EPI_ISL_2304116, EPI_ISL_2304117, EPI_ISL_2304118, EPI_ISL_2304120, EPI_ISL_2304121, EPI_ISL_2304124, EPI_ISL_2304125, EPI_ISL_2304126, EPI_ISL_2304128			
see above	Armed Forces Medical College Pune	National Centre For Cell Science – INSACOG	Ajay Pillai; Dhiraj Paul; INSACOG Consortium team; Manoj Kumar Bhat; Mitali Inamdar; Mohak P Gujare; Shivang P. Bhanushali; Yogesh Shouche
EPI_ISL_1827951	Australian Infectious Disease Research Centre, School of Chemistry and Molecular Biosciences, The University of Queensland	Australian Infectious Disease Research Centre, School of Chemistry and Molecular Biosciences, The University of Queensland	Parry; R.H. and Khromykh, A.
EPI_ISL_583637, EPI_ISL_583662, EPI_ISL_934436, EPI_ISL_2137225, EPI_ISL_2232832, EPI_ISL_2324190, EPI_ISL_2324315, EPI_ISL_2485065, EPI_ISL_2485083, EPI_ISL_2616202, EPI_ISL_2616245, EPI_ISL_2616260, EPI_ISL_2617655, EPI_ISL_2758039, EPI_ISL_2758056			
see above	Austrian Agency for Health and Food Safety (AGES)	Bergthaler laboratory, CeMM Research Center for Molecular Medicine of the Austrian Academy of Sciences	Adi Steinrigl; Alexander Lercher; Alexandra Popa; Andreas Bergthaler; Anna Schedl; Bekir Erguner; Benedikt Agerer; Christian Paar; Christoph Bock; Daniela Schmid; Dorothee von Laer; Elisabeth Puchhammer-Stoeckl; Fabian Amman; Franz Allerberger; Gernot Walder; Gregor Hörmann; Guenter Weiss; Gunther Vogl; Henrique Colaco; Jakob-Wendelin Genger; Jan Laine; Judith Aberle; Kinga Rigler-Hohenwarter; Lukas Endler; Maelle Le Moing; Manfred Nairz; Mark Smyth; Martin Senekowitsch; Matthew Thornton; Michael Schuster; Peter Hufnagl; Peter Obrist; Petr Triska; Rainer Gattringer; Sabine Sussitz-Rack; Stephan Aberle; Thomas Penz; Wegene Borena
EPI_ISL_882639	Azerbaijan National Hematology Center Division of Medical Genetics	Azerbaijan National Hematology Center Division of Medical Genetics	Aghayev Agha Rza; Bayramlı Ramin
EPI_ISL_1840787, EPI_ISL_2080266, EPI_ISL_2080267, EPI_ISL_2080268, EPI_ISL_2080270, EPI_ISL_2372806, EPI_ISL_2550684			
see above	Azienda Sanitaria dell'Alto Adige Laboratorio Aziendale di Microbiologia e Virologia	Istituto di Genomica Applicata	Davide Scaglione; Eleonora Paparelli; Elisa Masi; Elisabetta Giacobazzi; Elisabetta Pagani; Gabriele Magris; Irena Jurman; Irene Bianconi; Michele Morgante; Stefanie Wieser; Vera Vendramin
EPI_ISL_2389632, EPI_ISL_2389634, EPI_ISL_2389636, EPI_ISL_2389638, EPI_ISL_2389640, EPI_ISL_2389641, EPI_ISL_2389645, EPI_ISL_2389647, EPI_ISL_2389649, EPI_ISL_2389651, EPI_ISL_2389653, EPI_ISL_2389655, EPI_ISL_2389657			
see above	B.J. Medical College and Civil Hospital, Ahmedabad	Gujarat Biotechnology Research Centre	Chaitanya Joshi; Dinesh Kumar; Janvi Raval; Madhvi Joshi; Nitesh Shah; Nitin Savaliya; Pranay Shah; Ramesh Pandit; Sonal Sharma; Twinkle Soni; Umang Mishra; Zarna Patel; Zuber Saiyed
EPI_ISL_1969249	BBLK Palembang	National Institute of Health Research and Development	Arie Ardiansyah Nugraha; Hana Apsari Pawestri; Hartanti Dian Ikawati; Kartika Dewi Puspa; Krisna Pangesti; Nelly Puspandari; Subangkit; Triyani Soekarso; Vivi Setiawaty
EPI_ISL_2728297, EPI_ISL_2728300, EPI_ISL_2728302, EPI_ISL_2728303, EPI_ISL_2728304, EPI_ISL_2728305, EPI_ISL_2728306, EPI_ISL_2728307, EPI_ISL_2728465, EPI_ISL_2728466, EPI_ISL_2728469, EPI_ISL_2728472, EPI_ISL_2728473, EPI_ISL_2728475, EPI_ISL_2728476, EPI_ISL_2728477, EPI_ISL_2728479			
see above	BBMP Urban	INSACOG-KA, NIMHANS	Ananthapadmanabha Kotambail; Anita S Desai; Anson Kunjumon George; Chetan G K; Chitra Pattabiraman; Darshan Sreenivas; Ellango Ramasamy; Gautham Arunachal Udupi; Mahesh Kumar.C.S; Sony Sharma; V Ravi
EPI_ISL_1007661	BBMP Urban PHC	Department of Neurovirology, National Institute of Mental Health and Neurosciences (NIMHANS)	Anita S Desai; Anson Kunjumon George; Chitra Pattabiraman; Darshan Sreenivas; Nakka Vijay Kiran Reddy; Pramada Prasad; Risha Rasheed; V Ravi
EPI_ISL_969453, EPI_ISL_2522408, EPI_ISL_2523674, EPI_ISL_2524651, EPI_ISL_2529589, EPI_ISL_2530862, EPI_ISL_2530880, EPI_ISL_2530909, EPI_ISL_2530922, EPI_ISL_2530949, EPI_ISL_2530951, EPI_ISL_2530954, EPI_ISL_2530962, EPI_ISL_2531015, EPI_ISL_2531020, EPI_ISL_2531022, EPI_ISL_2531043, EPI_ISL_2531046, EPI_ISL_2531048, EPI_ISL_2531050, EPI_ISL_2531058, EPI_ISL_2531067, EPI_ISL_2531077, EPI_ISL_2531079, EPI_ISL_2531082, EPI_ISL_2531084, EPI_ISL_2531086, EPI_ISL_2531090, EPI_ISL_2531092, EPI_ISL_2531094, EPI_ISL_2531096, EPI_ISL_2531097, EPI_ISL_2531098, EPI_ISL_2531099, EPI_ISL_2531100, EPI_ISL_2531104, EPI_ISL_2531105, EPI_ISL_2655690, EPI_ISL_2655803, EPI_ISL_2655902, EPI_ISL_2655964, EPI_ISL_2655972, EPI_ISL_2655997, EPI_ISL_2655998, EPI_ISL_2656001, EPI_ISL_2656003, EPI_ISL_2656025, EPI_ISL_2656028, EPI_ISL_2656077, EPI_ISL_2656181, EPI_ISL_2656303, EPI_ISL_2656337, EPI_ISL_2656349, EPI_ISL_2656460, EPI_ISL_2656462, EPI_ISL_2656487, EPI_ISL_2657111, EPI_ISL_2657112, EPI_ISL_2657119, EPI_ISL_2719041, EPI_ISL_2719042, EPI_ISL_2719043, EPI_ISL_2719048, EPI_ISL_2719261, EPI_ISL_2719268, EPI_ISL_2719339, EPI_ISL_2719387, EPI_ISL_2719389, EPI_ISL_2719392, EPI_ISL_2719401, EPI_ISL_2719407, EPI_ISL_2719427, EPI_ISL_2719459, EPI_ISL_2719483, EPI_ISL_2719510, EPI_ISL_2719559, EPI_ISL_2719571, EPI_ISL_2719705, EPI_ISL_2719706, EPI_ISL_2719732, EPI_ISL_2719872, EPI_ISL_2719873, EPI_ISL_2719879, EPI_ISL_2719883, EPI_ISL_2719897, EPI_ISL_2719905, EPI_ISL_2719923, EPI_ISL_2719931, EPI_ISL_2720222, EPI_ISL_2720228, EPI_ISL_2720230, EPI_ISL_2720291, EPI_ISL_2720609, EPI_ISL_2720610, EPI_ISL_2721073, EPI_ISL_2721074, EPI_ISL_2721075, EPI_ISL_2721120, EPI_ISL_2721139, EPI_ISL_2721140, EPI_ISL_2721355, EPI_ISL_2721398			
see above	BCCDC Public Health Laboratory	BCCDC Public Health Laboratory	Ana Pacagnella; Corrinne Ng; Dan Fornika; John Tyson; Kim Macdonald; Kimia Kamelian; Linda Hoang; Loretta Janz; Mel Krajden; Prystajecky Natalie; Robert Azana; Robert Azana Terry Snutch; Shannon Russell
EPI_ISL_1312755, EPI_ISL_2141743	BIOR	Latvian Biomedical Research and Study Centre	Daina Pule; Davids Fridmanis; Elina Dimina; Guntars Zarins; Irena Meistere; Ivars Silamikelis; Janis Klovins; Janis Pjalkovskis; Jurijs Perevoscikovs; Kaspars Megnis; Laila Silamikele; Lauma Freimane; Laura Ansone; Liga Birzniece; Monta Ustinova; Nikita Zrelovs; Uga Dumpis; Una Krumina; Vita Rovite
EPI_ISL_2017765, EPI_ISL_2017766, EPI_ISL_2017767, EPI_ISL_2017768, EPI_ISL_2017769, EPI_ISL_2017770, EPI_ISL_2017771, EPI_ISL_2017772, EPI_ISL_2017773, EPI_ISL_2017774, EPI_ISL_2017775, EPI_ISL_2017776, EPI_ISL_2017777, EPI_ISL_2017778, EPI_ISL_2017779, EPI_ISL_2017780, EPI_ISL_2017781, EPI_ISL_2017783, EPI_ISL_2017784, EPI_ISL_2017785, EPI_ISL_2017786, EPI_ISL_2017787, EPI_ISL_2017788, EPI_ISL_2391488, EPI_ISL_2405127, EPI_ISL_2405128, EPI_ISL_2405129, EPI_ISL_2405130, EPI_ISL_2405131, EPI_ISL_2405132, EPI_ISL_2405133, EPI_ISL_2405134, EPI_ISL_2405135, EPI_ISL_2405136, EPI_ISL_2405137, EPI_ISL_2405138, EPI_ISL_2405139, EPI_ISL_2405140, EPI_ISL_2405141, EPI_ISL_2405142, EPI_ISL_2405144, EPI_ISL_2509938			
see above	BJ Medical College and Civil Hospital, Ahmedabad	Gujarat Biotechnology Research Centre	Chaitanya Joshi; Dinesh Kumar; Janvi Raval; Madhvi Joshi; Nitesh Shah; Nitin Savaliya; Pranay Shah; Pranay Shah; Ramesh Pandit; Sonal Sharma; Twinkle Soni; Umang Mishra; Zarna Patel; Zuber Saiyed
EPI_ISL_2106975	BPOM Gorontalo	National Institute of Health Research and Development	Arie Ardiansyah Nugraha; Hana Apsari Pawestri; Hartanti Dian Ikawati; Kartika Dewi Puspa; Krisna Pangesti; Nelly Puspandari; Subangkit; Triyani Soekarso; Vivi Setiawaty
EPI_ISL_1663247, EPI_ISL_2342606, EPI_ISL_2342617, EPI_ISL_2342621, EPI_ISL_2342625, EPI_ISL_2342636, EPI_ISL_2342647, EPI_ISL_2342651, EPI_ISL_2342659, EPI_ISL_2342661, EPI_ISL_2342669, EPI_ISL_2342670, EPI_ISL_2342685, EPI_ISL_2342692, EPI_ISL_2342694, EPI_ISL_2342697, EPI_ISL_2342698, EPI_ISL_2342700, EPI_ISL_2342702, EPI_ISL_2342707, EPI_ISL_2342708, EPI_ISL_2342711, EPI_ISL_2342715, EPI_ISL_2342810, EPI_ISL_2342834, EPI_ISL_2342857, EPI_ISL_2342863, EPI_ISL_2342864, EPI_ISL_2342866, EPI_ISL_2342867, EPI_ISL_2342868, EPI_ISL_2342869, EPI_ISL_2342870, EPI_ISL_2342871, EPI_ISL_2342873, EPI_ISL_2342875, EPI_ISL_2342876, EPI_ISL_2342877, EPI_ISL_2342878, EPI_ISL_2342879, EPI_ISL_2342880, EPI_ISL_2342881			
see above	BRLSABVM Government Medical College, Rajnandgaon, Chhattisgarh	Institute of Life Sciences - INSACOG	Ajay Parida; Amol M. Kanampalliwar; Arup Ghosh; Atimukta Jha; INSACOG Consortium; Omprakash Shiriwas; Punit Prasad; Rajeeb Swain; Rupesh Dash; Safal Walia; Sana Fatma; Shifu Aggarwal; Sunil K. Raghav
EPI_ISL_1265456	BTKLPP Kelas I Makassar	Eijkman Institute for Molecular Biology, Ministry of Research and Technology/National Agency for Research and Innovation; National Institute of Health Research and Development	Amin Soebandrio; Edison Johar; Frilasita A Yudhaputri; Hana Apsari Pawestri; Hidayat Trimarsanto; Iskandar Adnan; Khin Saw Myint; Lydia V. Panggalo; Safarina G Malik; Slamet; Sukma Oktavianthi; Vivi Setiawaty; Willy Agustine
EPI_ISL_1159375	Bahman Hospital	Laboratory of Molecular Biology and Cancer Immunology,Lebanese University Public Health England	Fadi Abdel Sater; Steven Pullan
EPI_ISL_2558710	Balai Besar Penelitian dan Pengembangan dan Reservoir Penyakit, Salatiga	Genomik Solidaritas Indonesia Laboratorium	Alfin Mohammad Abdillah; Anindya Pradipta Susanto; Annisa Muthiah Sukirman; Anuraj Shankar; Ariel Pradipta; Budi Wiweko; Carissa Sintca Wijaya; Dhahlia Agustina Cahyono; Gracia Felias Enos Korompis; Joko Waluyo; Louisa Markus; Meutia Ayuputeri Kumaheri; Normastuti Adhini Tantyo; Pratiwi Sudarmono; Vania Gavrila Wikasa; Yudistira Aditya Rahmat
EPI_ISL_2047564	Balai Litbangkes Tanah Bumbu	National Institute of Health Research and Development	Arie Ardiansyah Nugraha; Hana Apsari Pawestri; Hartanti Dian Ikawati; Juhairiyah; Kartika Dewi Puspa; Krisna Pangesti; Nelly Puspandari; Subangkit; Syarif Hidayat; Triyani Soekarso; Vivi Setiawaty
EPI_ISL_2373501, EPI_ISL_2373502, EPI_ISL_2373503, EPI_ISL_2373504, EPI_ISL_2373505, EPI_ISL_2373506, EPI_ISL_2373509, EPI_ISL_2373510, EPI_ISL_2373512, EPI_ISL_2373514, EPI_ISL_2373515, EPI_ISL_2373517, EPI_ISL_2373519, EPI_ISL_2373521, EPI_ISL_2373522, EPI_ISL_2373523, EPI_ISL_2373524, EPI_ISL_2373525, EPI_ISL_2373526, EPI_ISL_2373527, EPI_ISL_2373529, EPI_ISL_2373530, EPI_ISL_2373531, EPI_ISL_2373532, EPI_ISL_2373533, EPI_ISL_2373534, EPI_ISL_2373535, EPI_ISL_2373536, EPI_ISL_2373545, EPI_ISL_2373546, EPI_ISL_2373547, EPI_ISL_2373554, EPI_ISL_2373559, EPI_ISL_2373561, EPI_ISL_2373562, EPI_ISL_2373567, EPI_ISL_2373576, EPI_ISL_2373578, EPI_ISL_2373590, EPI_ISL_2373593, EPI_ISL_2373601, EPI_ISL_2373603, EPI_ISL_2400320, EPI_ISL_2400322, EPI_ISL_2400327			
see above	Banaras Hindu University	CSIR-Centre for Cellular and Molecular Biology-INSACOG	; Abhay Kumar Yadav; Abhishek Pathak; Ajay Kumar Yadav; Amareshwar Vodapalli; Ara Sreenivas; Archana Bharadwaj Siva; Ashish; B Himasri; Blessy B John; Chetan Sahni; Debashruti Das; Deepa Devadas; Divya Tej Sowpati; Gunjan Rai; Gyaneshwer Chaubey; Jaya Chakraborty; Karthik Bharadwaj Tallapaka; Lamuk Zaveri; Maneesha Upadhyay; Manpreet Kaur; Nitish Kumar Singh; Payel Mukherjee; Prajjval Pratap Singh; Priyoneel Basu; Rakesh K Mishra; Royana Singh; Saumya Singh; Saurabh Singh; Shani Vishwakarma; Sharath Chandra Thota; Shivam Tiwari; Shivani Mishra; Shreekant Verma; Sofia Banu; Surendra Pratap Mishra; Tribhuwan Mohan Mohapatra; Tulasi Nagabandi; Umesh Choudhary; Valli Nagalakshmi Undamatla; Vijay Kumar Shukla; Viswagithe S L; Yashvant Patel
EPI_ISL_2724287, EPI_ISL_2724288, EPI_ISL_2724289, EPI_ISL_2724290, EPI_ISL_2724291, EPI_ISL_2724296, EPI_ISL_2724297, EPI_ISL_2724299, EPI_ISL_2724300, EPI_ISL_2724301, EPI_ISL_2724302, EPI_ISL_2724303, EPI_ISL_2724312, EPI_ISL_2724313			
see above	Bangalore Baptist Hospital	inStem NCBS – INSACOG	Uma Ramakrishnan Dasaradhi Palakodeti Aswin SaiNarain
EPI_ISL_2343252, EPI_ISL_2501026	Banteay Meanchey Rapid Response Team	Virology Unit, Institut Pasteur du Cambodge	Cecile Troupin; Chau Darapheak; Chin Savuth; Erik A Karlsson; Jurre Y Siegers; Kraing Sidonn; Leakhena Pum; Ly Sovann; Veasna Duong; Yi Sengdoeurn

EPI_ISL_2801894	Banteay Meanchey Regional Laboratory	Virology Unit, Institut Pasteur du Cambodge	Cecile Troupin; Chau Darapheak; Chin Savuth; Erik A Karlsson; Jurre Y Siegers; Kraing Sidonn; Leakhena Pum; Ly Sovann; Veasna Duong; Yi Sengdoeurn
EPI_ISL_2126019	Barts Health NHS Trust	COVID-19 Genomics UK (COG-UK) Consortium	BROAD; Beatrix; CUTINO-MOGUEL; Claire; David; Dola; HARRINGTON; KELE; KULASEGARAN-SHYLINI; Maria-Teresa; OWOYEMI; Raghavendran
EPI_ISL_2406458	Battambang Provincial Laboratory	Virology Unit, Institut Pasteur du Cambodge	Cecile Troupin; Chau Darapheak; Chin Savuth; Erik A Karlsson; Jurre Y Siegers; Kraing Sidonn; Leakhena Pum; Ly Sovann; Veasna Duong; Yi Sengdoeurn
EPI_ISL_1725322	Bayerisches Landesamt fÃ¼r Gesundheit und Lebensmittelsicherheit (LGL)	Robert Koch Institute	
EPI_ISL_2260430, EPI_ISL_2260533, EPI_ISL_2260540, EPI_ISL_2260543, EPI_ISL_2262724, EPI_ISL_2262751, EPI_ISL_2262763			
see above	Bayerisches Landesamt für Gesundheit und Lebensmittelsicherheit (LGL)	Robert Koch Institute	
EPI_ISL_1833923, EPI_ISL_1833946, EPI_ISL_2128517	Bioinformatics and Biostatistics Lab, Advanced Sequencing Facility	COVID-19 Genomics UK (COG-UK) Consortium	Aengus Stewart; Chelsea Sawyer; Harshil Patel; Jerome Nicod; Laura Cubitt; Margaret Crawford
EPI_ISL_430005, EPI_ISL_430013, EPI_ISL_450189, EPI_ISL_635778, EPI_ISL_635779, EPI_ISL_635782, EPI_ISL_730263, EPI_ISL_730526, EPI_ISL_755254, EPI_ISL_878571			
see above	Biolab Diagnostic Laboratories	Andersen lab at Scripps Research	Ahmad Tibi; Amid Abdelnour with SEARCH Alliance San Diego; Issa Abu-Dayyeh; Lama Hussein; Lina Mohammad; Zein Naber
EPI_ISL_1404614, EPI_ISL_1406143, EPI_ISL_1406195, EPI_ISL_1822601, EPI_ISL_1823177, EPI_ISL_1824718, EPI_ISL_2105671, EPI_ISL_2105672, EPI_ISL_2105673, EPI_ISL_2105674, EPI_ISL_2617449, EPI_ISL_2617452, EPI_ISL_2617453, EPI_ISL_2617454, EPI_ISL_2617455, EPI_ISL_2617459			
see above	Biolab Diagnostic Laboratories	Biolab Diagnostic Laboratories	Ahmad Tibi; Amid Abdelnour; Badia Saddedin; Eiad Atwa; Issa Abu-Dayyeh; Lama Hussein; Shaima Ali; Shayma Ali
EPI_ISL_1524334, EPI_ISL_1524342, EPI_ISL_1524343, EPI_ISL_1524362, EPI_ISL_1524367, EPI_ISL_1524368, EPI_ISL_1524370, EPI_ISL_2467913			
see above	Biology Department, College of Science, Al Muthanna University and Public Health Laboratory, Al-Muthanna Health Directorate	Department of Virology, Faculty of Medicine, University of Helsinki, Helsinki, Finland	Alaa Hameed; Ali Jasim; Hussein Alburkat; Murad Munahi; Nihad Al-Rashedi; Olli Vapalahti; Tarja Sironen; Teemu Smura
EPI_ISL_907084	Biology, MCL	Biology, MCL	A.F.; A.N.; Elhoseiny; Gad; M.D.; M.F.; M.G.; Seadawy; Shamel; Zekri
EPI_ISL_1420654	Biomedical Research Foundation of the Academy of Athens (BRFAA)	Greek Genome Center, Biomedical Research Foundation of the Academy of Athens (BRFAA)	Dimitrios Thanos; Emmanouil Athanasiadis; Ioannis Vatsellas; Katerina Zoi; Theodoros Loupis
EPI_ISL_516085	Biomedical Sciences and Public Health, Polytechnic University of Marche	Biomedical Sciences and Public Health, Polytechnic University of Marche	Alessandrini, F.; Bagnarelli, P.; Caucci, S.; Di Sante, L.; Melchionda, F.; Menzo, S.; Onofri, V.; Tagliabracci, A.; Turchi, C.
EPI_ISL_1916570, EPI_ISL_1916696, EPI_ISL_1917032, EPI_ISL_2401459, EPI_ISL_2401565, EPI_ISL_2401612, EPI_ISL_2401729			
see above	BioneXt Lab	Laboratoire national de sante, Microbiology, Microbial Genomics Platform	Anke Wienecke-Baldacchino; Catherine Ragimbeau; Fatu Djabi; Jessica Tapp; Lise Pignon; Raoul Salmon; Tamir Abdelrahman; Thibault Ferrandon
EPI_ISL_1588696	Bioptická laboratoř s.r.o.	Bioptická laboratoř s.r.o.	Martina Putzová; Michaela Říhová; Nikola Bílá; Petr Šteiner; Silva Vondráková; Tomáš Vaněček
EPI_ISL_1970614, EPI_ISL_2227858	Bioptická laboratoř, s.r.o.	Bioptická laboratoř, s.r.o.	Martina Putzová; Michaela Říhová; Nikola Bílá; Petr Šteiner; Silva Vondráková; Tomáš Vaněček
EPI_ISL_2108737, EPI_ISL_2108817, EPI_ISL_2120670, EPI_ISL_2120779, EPI_ISL_2121818, EPI_ISL_2122020, EPI_ISL_2125827, EPI_ISL_2260789, EPI_ISL_2470057, EPI_ISL_2636914, EPI_ISL_2762695			
see above	Bioscientia Labor Wermsdorf	Robert Koch Institute	
EPI_ISL_2109742, EPI_ISL_2126150	Bioscientia MVZ Labor Karlsruhe GmbH	Robert Koch Institute	
EPI_ISL_1970089, EPI_ISL_1970092, EPI_ISL_1970094, EPI_ISL_1970095, EPI_ISL_1970099, EPI_ISL_1970100, EPI_ISL_1970101, EPI_ISL_1970102, EPI_ISL_1970103, EPI_ISL_1970105, EPI_ISL_1970106, EPI_ISL_1970108, EPI_ISL_1970109, EPI_ISL_1970110, EPI_ISL_1970111, EPI_ISL_1970112, EPI_ISL_1970114, EPI_ISL_1970133, EPI_ISL_1970134, EPI_ISL_1970135, EPI_ISL_1970136, EPI_ISL_1970149, EPI_ISL_1970153, EPI_ISL_1970154, EPI_ISL_1970155, EPI_ISL_1970157, EPI_ISL_1970158, EPI_ISL_1970159, EPI_ISL_1970160, EPI_ISL_1970163, EPI_ISL_1970164, EPI_ISL_1970168, EPI_ISL_1970169, EPI_ISL_1970170, EPI_ISL_1970171, EPI_ISL_1970172, EPI_ISL_1970179, EPI_ISL_1970211, EPI_ISL_1970215, EPI_ISL_1970221, EPI_ISL_1970223, EPI_ISL_1970224, EPI_ISL_1970237, EPI_ISL_1970238, EPI_ISL_1970239, EPI_ISL_1970241, EPI_ISL_1970242, EPI_ISL_1970243, EPI_ISL_1970247, EPI_ISL_1970249, EPI_ISL_1970250, EPI_ISL_1970251, EPI_ISL_1970267, EPI_ISL_1970269, EPI_ISL_1970274, EPI_ISL_1970276, EPI_ISL_1970277, EPI_ISL_1970278, EPI_ISL_1970279, EPI_ISL_1970281, EPI_ISL_1970286, EPI_ISL_1970288, EPI_ISL_1970310, EPI_ISL_1970313, EPI_ISL_1970314, EPI_ISL_1970318, EPI_ISL_1970319, EPI_ISL_1970323, EPI_ISL_1970324, EPI_ISL_1970327, EPI_ISL_1970328, EPI_ISL_1970332, EPI_ISL_1970333, EPI_ISL_1970334, EPI_ISL_1970335, EPI_ISL_1970336, EPI_ISL_1970337, EPI_ISL_1970340			
see above	Biotechnology Division, NCDC Delhi	NCDC Delhi, Biotechnology Division	Hema Gogia; Hemlata Lall; Kalaiarasan Ponnusamy; Mahesh S Dhar; Manoj K Singh; Meena Datta; Partha Rakshit; Preeti Madan; Priyanka Singh; Radhakrishnan V. S; Robin Marwal; Sandhya Kabra; Sujeet K Singh; Uma Sharma
EPI_ISL_2360256, EPI_ISL_2360257	Boshehr University of Medical Sciences	National Influenza Center	A Nejati; F Ajaminejad; F Ajaminejad and T Mokhtari Azad; J Yavarian; K Sadeghi; N Ghavvami; N Ghavvami and T Mokhtari Azad; NZ Shafiei Jandaghi; V Salimi; Mostafa Salehi-Vaziri
EPI_ISL_1287760, EPI_ISL_1516828, EPI_ISL_1516836, EPI_ISL_1516855, EPI_ISL_1516862, EPI_ISL_1516877, EPI_ISL_2372279, EPI_ISL_2372295, EPI_ISL_2372297, EPI_ISL_2504073, EPI_ISL_2504074, EPI_ISL_2504086, EPI_ISL_2504089, EPI_ISL_2504090, EPI_ISL_2566206, EPI_ISL_2566207			
see above	Botswana Harvard HIV Reference Laboratory	Botswana Harvard HIV Reference Laboratory	Boitumelo Zuze; Botshelo Radibe; David Lawrence; Dorcas Maruapula; Joseph Makhema; Keoratile Ntshambiwa; Kgomotso Moruisi; Legodile Kooepile; Madisa Mine; Modisa Motswaledi; Mosepele Mosepele; Ontlametse T. Bareng; Pamela Smith-Lawrence; Roger Shapiro; Shahin Lockman; Sikhulile Dorcas Maruapula; Sikhulile Moyo; Sikhulile Wonderful T. Choga; Simani Gaseitsiwe; Thongbotho Mphoyakgosi; Wonderful T. Choga
EPI_ISL_1000999, EPI_ISL_1001001, EPI_ISL_1001002, EPI_ISL_1034756, EPI_ISL_1034760	Bundeswehr Institute of Microbiology	Bundeswehr Institute of Microbiology	Alexandra Rehn; Enrico Georgi; Malena Bestehorn-Willmann; Markus Antwerpen; Mathias Walter; Mike Pillukat; Roman Wölfel; Sabine Zange
EPI_ISL_2131156	Bundeswehrkrankenhaus Berlin	Bundeswehr Institute of Microbiology	Alexandra Rehn; Enrico Georgi; Malena Bestehorn-Willmann; Markus Antwerpen; Mathias Walter; Mike Pillukat; Roman Wölfel; Sabine Zange
EPI_ISL_1085408	C.H du Cotentin	Department of Virology, Henri Mondor University Hospital, Assistance Publique Hôpitaux de Paris, Université Paris-Est Créteil, INSERM U955	Alexandre Soulier; Christophe Rodriguez; Elisabeth Trawinski; Guillaume Gricourt; Jean-Michel Pawlotsky; Melissa N'Debi; Slim Fourati; Vanessa Demontant
EPI_ISL_2612795	C.H.C MontLégia	GIGA Medical Genomics	Bouchra Boujemla; Cécile Meex; Keith Durkin; Maria Artesi; Marie-Pierre Hayette; Nathalie Renotte; Pierrette Melin; Raphaël Boreux; Sébastien Bontems; Vincent Bours
EPI_ISL_2386977, EPI_ISL_2633577, EPI_ISL_2633652	CENTOGENE Frankfurt Laboratory: Niederlassung Industriepark Höchst	Robert Koch Institute	
EPI_ISL_1795170	CENTRO DE SAUDE DE MARACAI	Instituto Butantan / ESALQ-Piracicaba	Antonio Jorge Martins; Bianca Cechetto Carlos. Mendelics: Bibiana Santos; Claudia Renata dos Santos Barros; David Schlesinger. Hemocentro Ribeirão Preto: Simone Kashima; Debora Botequio Moretti. Centro de Genômica Funcional da ESALQ: Luiz Lehmann Coutinho; Dimas Tadeu Covas; Elaine Cristina Marqueze; Elaine Vieira dos Santos; Elisangela Chicaroni Mattos; Erika Freitas; Evandra Strazza Rodrigues; Felipe Allan da Silva da Costa; Flavia Aburjaile; Guilherme Targino Valente; Heidge Fukumasu. USP-Botucatu: Rejane Maria Tommasini Grotto; Instituto Butantan: Alexander Roberto Precioso; Jayme A. Souza-Neto; Jessika Cristina Chagas Lesbon; José Salvatore Leister Patané; João Paulo Kitajima; Luiz Carlos Junior de Alcantara; Maria Carolina Elias; Marta Giovanetti; Patricia Akemi Assato; Rafael dos Santos Bezerra; Raquel de Lello Rocha Campos Cassano. NGS Soluções Genômicas: Pilar Drummond Sampaio Corrêa Mariani. FZEA-USP Pirassununga: Mirele Daiana Poleti; Raul Machado Neto; Ricardo Augusto Brassaloti; Ricardo Haddad; Rodrigo Tocantins Calado.; Sandra Coccuzzo Sampaio; Svetoslav Nanev Slavov; Vagner Fonseca; Vincent Louis Viala
EPI_ISL_2493539	CENTRO DE SAUDE III BORBOREMA	Instituto Butantan	Antonio Jorge Martins; Claudia Renata dos Santos Barros; David Schlesinger; Debora Botequio Moretti; Dimas Tadeu Covas; Elaine Cristina Marqueze; Elaine Vieira Santos; Evandra Strazza Rodrigues; Heidge Fukumasu; Jayme Augusto de Souza-Neto; José Salvatore Leister Patané; Luiz Alcantara; Luiz Lehmann Coutinho; Maria Carolina Elias; Maurício Lacerda Nogueira; Rafael dos Santos Bezerra; Raul Machado Neto; Rejane Maria Tommasini Grotto; Ricardo Haddad; Sandra Coccuzzo Sampaio Vessoni; Simone Kashima; Svetoslav Nanev Slavov; Vincent Louis Viala
EPI_ISL_1795429	CENTRO INTEGRADO DE SAUDE	Instituto Butantan / ESALQ-Piracicaba	Antonio Jorge Martins; Bianca Cechetto Carlos. Mendelics: Bibiana Santos; Claudia Renata dos Santos Barros; David Schlesinger. Hemocentro Ribeirão Preto: Simone Kashima; Debora Botequio Moretti. Centro de Genômica Funcional da ESALQ: Luiz Lehmann Coutinho; Dimas Tadeu Covas; Elaine Cristina Marqueze; Elaine Vieira dos Santos; Elisangela Chicaroni Mattos; Erika Freitas; Evandra Strazza Rodrigues; Felipe Allan da Silva da Costa; Flavia Aburjaile; Guilherme Targino Valente; Heidge Fukumasu. USP-Botucatu: Rejane Maria Tommasini Grotto; Instituto Butantan: Alexander Roberto Precioso; Jayme A. Souza-Neto; Jessika Cristina Chagas Lesbon; José Salvatore Leister Patané; João Paulo Kitajima; Luiz Carlos Junior de Alcantara; Maria Carolina Elias; Marta Giovanetti; Patricia Akemi Assato; Rafael dos Santos Bezerra; Raquel de Lello Rocha Campos Cassano. NGS Soluções Genômicas: Pilar Drummond Sampaio Corrêa Mariani. FZEA-USP Pirassununga: Mirele Daiana Poleti; Raul Machado Neto; Ricardo Augusto Brassaloti; Ricardo Haddad; Rodrigo Tocantins Calado.; Sandra Coccuzzo Sampaio; Svetoslav Nanev Slavov; Vagner Fonseca; Vincent Louis Viala
EPI_ISL_2714776	CENTRUM VOOR MEDISCHE ANALYSE	Labo Klinische Biologie, UZA	Basil Britto Xavier; Christine Lammens; Herman Goossens; Ines Verbesselt; Jasmine Coppens; Kathleen Holemans; Marie Le Mercier; Veerle Matheeussen
EPI_ISL_2226348	CHU POITIERS	CHU Poitiers	Agnes BEBY-DEFAUX; Birama N'DIAYE; Caroline MICHAUD; Magali GARCIA; Manon PRAT; Maxime PICHON; Nicolas LEVEQUE; Valentin BON-BARET
EPI_ISL_2790247, EPI_ISL_2790248	CHWAPI - SITE NOTRE DAME	Institut de Pathologie et Genetique (IPG)	Jérémie Gras; Pascale Hilbert
EPI_ISL_1663304, EPI_ISL_1663307, EPI_ISL_1663308, EPI_ISL_1663312	CIIMS, Bilaspur, Chhattisgarh	Institute of Life Sciences - INSACOG	Ajay Parida; Amol M. Kanampalliwar; Arup Ghosh; Atimukta Jha; INSACOG Consortium; Punit Prasad; Rajeeb Swain; Rupesh Dash; Safal Walia; Shifu Aggarwal; Sunil K. Raghav
EPI_ISL_1303374	CLINICA COLSANITAS CENTRAL DE REFERENCIA	Instituto Nacional de Salud- Dirección de Investigación en Salud Pública	Carlos Franco-Muñoz; Carmen Osorio; Diana Malo; Diego A. Álvarez-Díaz; Diego Andrés Prada; Gerardo Santamaría; Hector Alejandro Ruiz-Moreno; Jhonnatan Reales-González; Juan Camilo Martinez; Julian Naizaque; Katherine Laiton-Donato; Lisseth Pardo; Magdalena Wiesner; Marcela Mercado-Reyes; Maria T. Herrera-Sepúlveda; Marta Lopez Blanco; Martha Lucia Ospina Martinez; Sergio Gomez; Sheryll Corchuelo; Ángela Alarcon Cruz
EPI_ISL_683392	CNR Virus des Infections Respiratoires - France SUD	CNR Virus des Infections Respiratoires - France SUD	Antonin Bal; Bruno Lina; Gregory Destras; Gwendolyne Burfin; Laurence Josset; Martine Valette; Quentin Semanas
EPI_ISL_2484788	COMMUNITY TESTING CENTRE	Hong Kong Department of Health	Alan K.L. Tsang; Dominic N.C. Tsang; Edman T.K. Lam; Gannon C.K. Mak; Ken H.L. Ng; Peter C.W. Yip; Peter K.C. Cheng; Rickjason C.W. Chan
EPI_ISL_2227272	COVID-19 National Reference Laboratory, Pasteur Institute of Iran	Genetics Research Center, University of Social Welfare and Rehabilitation Sciences	Hossein Najmabadi.; Kimia Kahrizi; Mahsa Tavakoli; Marzieh Mohseni; Mohammad Hassan Pouriayevali; Mostafa Salehi-Vaziri; Tahmineh Jalali; Zohreh Fattahi
EPI_ISL_2227271	COVID-19 National Reference Laboratory, Pasteur Institute of Iran.	Genetics Research Center, University of Social Welfare and Rehabilitation Sciences	Hossein Najmabadi.; Kimia Kahrizi; Mahsa Tavakoli; Marzieh Mohseni; Mohammad Hassan Pouriayevali; Mostafa Salehi-Vaziri; Tahmineh Jalali; Zohreh Fattahi
EPI_ISL_2227204, EPI_ISL_2227268, EPI_ISL_2227269	COVID-19 National Reference Laboratoty, Pasteur Institute of Iran	Genetics Research Center, University of Social Welfare and Rehabilitation Sciences	Hossein Najmabadi.; Kimia Kahrizi; Mahsa Tavakoli; Marzieh Mohseni; Mohammad Hassan Pouriayevali; Mostafa Salehi-Vaziri; Tahmineh Jalali; Zohreh Fattahi
EPI_ISL_2227270	COVID-19 National Reference Laboratoty, Pasteur Institute of Iran.	Genetics Research Center, University of Social Welfare and Rehabilitation Sciences	Hossein Najmabadi.; Kimia Kahrizi; Mahsa Tavakoli; Marzieh Mohseni; Mohammad Hassan Pouriayevali; Mostafa Salehi-Vaziri; Tahmineh Jalali; Zohreh Fattahi

EPI_ISL_1942243	CQRC_QUALITY CONTROL CHEMICAL BIOLOGICAL RISK_AOOR Villa Sofia Cervello Palermo	CQRC_QUALITY CONTROL CHEMICAL BIOLOGICAL RISK_AOOR Villa Sofia Cervello Palermo	Bonvissuto, M.; Brunacci, G.; Buffa, V.; Contino, F.; Di Chiara, V.; Di Gaudio, F.; Di Sclafani, A.; Lungari, A.; Orlando, V.; Seidita, G.; Todaro, I.
EPI_ISL_1795242	CS DE URUPES	Instituto Butantan / ESALQ-Piracicaba	Antonio Jorge Martins; Bianca Cechetto Carlos. Mendelics: Bibiana Santos; Claudia Renata dos Santos Barros; David Schlesinger. Hemocentro Ribeirão Preto: Simone Kashima; Debora Botequio Moretti. Centro de Genômica Funcional da ESALQ: Luiz Lehmann Coutinho; Dimas Tadeu Covas; Elaine Cristina Marqueze; Elaine Vieira dos Santos; Elisangela Chicaroni Mattos; Erika Freitas; Evandra Strazza Rodrigues; Felipe Allan da Silva da Costa; Flavia Aburjaile; Guilherme Targino Valente; Heidge Fukumasu. USP-Botucatu: Rejane Maria Tommasini Grotto; Instituto Butantan: Alexander Roberto Precioso; Jayme A. Souza-Neto; Jessika Cristina Chagas Lesbon; José Salvatore Leister Patané; João Paulo Kitajima; Luiz Carlos Junior de Alcantara; Maria Carolina Elias; Marta Giovanetti; Patricia Akemi Assato; Rafael dos Santos Bezerra; Raquel de Lello Rocha Campos Cassano. NGS Soluções Genômicas: Pilar Drummond Sampaio Corrêa Mariani. FZEA-USP Pirassununga: Mirele Daiana Poleti; Raul Machado Neto; Ricardo Augusto Brassaloti; Ricardo Haddad; Rodrigo Tocantins Calado.; Sandra Coccuzzo Sampaio; Svetoslav Nanev Slavov; Vagner Fonseca; Vincent Louis Viala
EPI_ISL_2614645	CSIR Institute of Himalayan Bioresource Technology	CSIR Institute of Himalayan Bioresource Technology	Narendra Tirpude; Ravi Kumar; Vishal Acharya
EPI_ISL_539675	CSIR-Centre for Cellular and Molecular Biology	CSIR-Centre for Cellular and Molecular Biology	Archana Bharadwaj Siva; Dhiviya Vedagiri; Divya Gupta; Divya Tej Sowpati; Karthik Bharadwaj Tallapaka; Kezia J Ann; Krishnan Harinivas Harshan; Lamuk Zaveri; M Soujanya Reddy; Namami Gaur; Nikhil Hajirnis; Onkar Kulkarni; Payel Mukherjee; Pratheusa Maccha; Priya Singh; Purushotham Vodnala; Radhika Khandelwal; Rakesh K Mishra; Roshan Maku Venkata; Sakshi Shambhavi; Santosh Kumar Kuncha; Shagufta Khan; Shemin Mansuri; Sofia Banu; Sonu Uday; Tulasi Nagabandi; Vishal Sah
EPI_ISL_1838398, EPI_ISL_1838426, EPI_ISL_1838463, EPI_ISL_1838549, EPI_ISL_1838603, EPI_ISL_1838604, EPI_ISL_1838606, EPI_ISL_1838607, EPI_ISL_1838608, EPI_ISL_1838609, EPI_ISL_1838611, EPI_ISL_1838612, EPI_ISL_1838613, EPI_ISL_1838614, EPI_ISL_1838638, EPI_ISL_1838642, EPI_ISL_1838645, EPI_ISL_1838646, EPI_ISL_1838647, EPI_ISL_1838648, EPI_ISL_1838649, EPI_ISL_1838650, EPI_ISL_1838651, EPI_ISL_1838656, EPI_ISL_1838659, EPI_ISL_1838660, EPI_ISL_1838671, EPI_ISL_1838672, EPI_ISL_1838673, EPI_ISL_1838676, EPI_ISL_1838693, EPI_ISL_1838694, EPI_ISL_1838712, EPI_ISL_1838721, EPI_ISL_1838787, EPI_ISL_1838805, EPI_ISL_1838806, EPI_ISL_1838809, EPI_ISL_1838811, EPI_ISL_1838812, EPI_ISL_1838814, EPI_ISL_1838828, EPI_ISL_1838843, EPI_ISL_1838867, EPI_ISL_1838914, EPI_ISL_2156833, EPI_ISL_2156836, EPI_ISL_2156837, EPI_ISL_2156840, EPI_ISL_2156843, EPI_ISL_2156844, EPI_ISL_2156845, EPI_ISL_2156846, EPI_ISL_2156849, EPI_ISL_2156850, EPI_ISL_2156851, EPI_ISL_2156852, EPI_ISL_2156856, EPI_ISL_2156857, EPI_ISL_2156858, EPI_ISL_2156859, EPI_ISL_2156860, EPI_ISL_2156862, EPI_ISL_2156863, EPI_ISL_2156864, EPI_ISL_2156865, EPI_ISL_2156866, EPI_ISL_2156867, EPI_ISL_2156868, EPI_ISL_2156869, EPI_ISL_2156870, EPI_ISL_2156874, EPI_ISL_2156875, EPI_ISL_2156876, EPI_ISL_2156877, EPI_ISL_2156878, EPI_ISL_2156879, EPI_ISL_2156880, EPI_ISL_2156882, EPI_ISL_2156884, EPI_ISL_2156885, EPI_ISL_2156886, EPI_ISL_2156887, EPI_ISL_2156888, EPI_ISL_2156891, EPI_ISL_2156892, EPI_ISL_2156893, EPI_ISL_2156894, EPI_ISL_2156895, EPI_ISL_2156898, EPI_ISL_2156899, EPI_ISL_2156900, EPI_ISL_2156901, EPI_ISL_2156902, EPI_ISL_2156905, EPI_ISL_2156906, EPI_ISL_2156907, EPI_ISL_2156908, EPI_ISL_2156913, EPI_ISL_2156914, EPI_ISL_2156939, EPI_ISL_2156940, EPI_ISL_2156941, EPI_ISL_2156942, EPI_ISL_2156943, EPI_ISL_2156944, EPI_ISL_2156946, EPI_ISL_2156947, EPI_ISL_2156948, EPI_ISL_2156949, EPI_ISL_2156950, EPI_ISL_2156958, EPI_ISL_2156964, EPI_ISL_2156968, EPI_ISL_2156969, EPI_ISL_2156984, EPI_ISL_2156990, EPI_ISL_2156991, EPI_ISL_2156994, EPI_ISL_2156995, EPI_ISL_2156996, EPI_ISL_2156997, EPI_ISL_2156999, EPI_ISL_2157000, EPI_ISL_2157001, EPI_ISL_2157010, EPI_ISL_2157017, EPI_ISL_2157018, EPI_ISL_2157036, EPI_ISL_2157037, EPI_ISL_2157038, EPI_ISL_2157039, EPI_ISL_2157044, EPI_ISL_2157046, EPI_ISL_2157047, EPI_ISL_2157050, EPI_ISL_2157051, EPI_ISL_2157053, EPI_ISL_2157054, EPI_ISL_2157057, EPI_ISL_2157058, EPI_ISL_2157060, EPI_ISL_2157063, EPI_ISL_2157065, EPI_ISL_2157066, EPI_ISL_2157067, EPI_ISL_2157068, EPI_ISL_2157076, EPI_ISL_2157077, EPI_ISL_2157078, EPI_ISL_2157079, EPI_ISL_2157080, EPI_ISL_2157081, EPI_ISL_2157082, EPI_ISL_2157085, EPI_ISL_2157092, EPI_ISL_2157093, EPI_ISL_2157096, EPI_ISL_2157099, EPI_ISL_2157100, EPI_ISL_2157102, EPI_ISL_2157103, EPI_ISL_2157104, EPI_ISL_2157105, EPI_ISL_2157106, EPI_ISL_2157107, EPI_ISL_2157111, EPI_ISL_2157112, EPI_ISL_2157113, EPI_ISL_2157114, EPI_ISL_2157115, EPI_ISL_2157118, EPI_ISL_2157120, EPI_ISL_2157121, EPI_ISL_2157123, EPI_ISL_2157124, EPI_ISL_2157125, EPI_ISL_2157129, EPI_ISL_2157130, EPI_ISL_2157154, EPI_ISL_2157155, EPI_ISL_2373243, EPI_ISL_2373246, EPI_ISL_2373247, EPI_ISL_2373248, EPI_ISL_2373249, EPI_ISL_2373251, EPI_ISL_2373252, EPI_ISL_2373253, EPI_ISL_2373254, EPI_ISL_2373256, EPI_ISL_2373257, EPI_ISL_2373262, EPI_ISL_2373263, EPI_ISL_2373264, EPI_ISL_2373265, EPI_ISL_2373266, EPI_ISL_2373268, EPI_ISL_2373269, EPI_ISL_2373271, EPI_ISL_2373272, EPI_ISL_2373273, EPI_ISL_2373274, EPI_ISL_2373275, EPI_ISL_2373276, EPI_ISL_2373277, EPI_ISL_2373278, EPI_ISL_2373279, EPI_ISL_2373280, EPI_ISL_2373281, EPI_ISL_2373282, EPI_ISL_2373286, EPI_ISL_2373287, EPI_ISL_2373288, EPI_ISL_2373289, EPI_ISL_2373290, EPI_ISL_2373291, EPI_ISL_2373292, EPI_ISL_2373293, EPI_ISL_2373294, EPI_ISL_2373295, EPI_ISL_2373296, EPI_ISL_2373297, EPI_ISL_2373298, EPI_ISL_2373299, EPI_ISL_2373300, EPI_ISL_2373301, EPI_ISL_2373302, EPI_ISL_2373303, EPI_ISL_2373304, EPI_ISL_2373305, EPI_ISL_2373306, EPI_ISL_2373341, EPI_ISL_2373342, EPI_ISL_2373343, EPI_ISL_2373349, EPI_ISL_2400319, EPI_ISL_2441325, EPI_ISL_2441676, EPI_ISL_2660993, EPI_ISL_2661022, EPI_ISL_2661218, EPI_ISL_2661228, EPI_ISL_2774836, EPI_ISL_2774839, EPI_ISL_2774840, EPI_ISL_2774841, EPI_ISL_2774844, EPI_ISL_2774846, EPI_ISL_2774847, EPI_ISL_2774858, EPI_ISL_2774863, EPI_ISL_2774864, EPI_ISL_2774868, EPI_ISL_2774870, EPI_ISL_2774872, EPI_ISL_2774873, EPI_ISL_2774877, EPI_ISL_2774878, EPI_ISL_2774881, EPI_ISL_2774882, EPI_ISL_2774884, EPI_ISL_2774891, EPI_ISL_2774893, EPI_ISL_2774897, EPI_ISL_2774898, EPI_ISL_2774899, EPI_ISL_2774901, EPI_ISL_2774902, EPI_ISL_2774903, EPI_ISL_2774905, EPI_ISL_2774909, EPI_ISL_2774913, EPI_ISL_2774915, EPI_ISL_2774984, EPI_ISL_2774996, EPI_ISL_2775054, EPI_ISL_2775112, EPI_ISL_2775114, EPI_ISL_2775116, EPI_ISL_2775117, EPI_ISL_2775119, EPI_ISL_2789351			
see above	CSIR-Centre for Cellular and Molecular Biology	CSIR-Centre for Cellular and Molecular Biology-INSACOG	; Amareshwar Vodapalli; Ara Sreenivas; Archana Bharadwaj Siva; B Himasri; Blessy B John; Divya Tej Sowpati; Jandhyala Sai Krishna; Karthik Bharadwaj Tallapaka; Lamuk Zaveri; Onkar Kulkarni; Payel Mukherjee; Priya Nurkurthy; Rakesh K Mishra; Sharath Chandra Thota; Shreekant Verma; Sofia Banu; Sumedha Avadhanula; Tulasi Nagabandi; Valli Nagalakshmi Undamatla; Vidhyadhari Methuku; Viswagithe S L
EPI_ISL_1916411, EPI_ISL_1916412, EPI_ISL_1916414, EPI_ISL_1916417, EPI_ISL_1916423, EPI_ISL_1916424, EPI_ISL_1916433, EPI_ISL_1916434, EPI_ISL_1916442, EPI_ISL_1916445, EPI_ISL_1916446, EPI_ISL_1916454, EPI_ISL_1916456, EPI_ISL_1916464, EPI_ISL_1916465, EPI_ISL_1916475, EPI_ISL_1916476, EPI_ISL_1916486, EPI_ISL_1916489, EPI_ISL_1970629			
see above	CSIR-National Environmental Engineering Research Institute	CSIR-Centre for Cellular and Molecular Biology - INSACOG	Amareshwar Vodapalli; Ara Sreenivas; B Himasri; Divya Tej Sowpati; Karthik Bharadwaj Tallapaka; Krishna Khairnar; Lamuk Zaveri; Onkar Kulkarni; Rakesh K Mishra; Sharath Chandra Thota; Shreekant Verma; Sofia Banu; Viswagithe S L
EPI_ISL_2441372, EPI_ISL_2441385, EPI_ISL_2441386, EPI_ISL_2441391, EPI_ISL_2441392, EPI_ISL_2441396, EPI_ISL_2441400, EPI_ISL_2441401, EPI_ISL_2441402, EPI_ISL_2441403, EPI_ISL_2441404, EPI_ISL_2441405, EPI_ISL_2441406, EPI_ISL_2441407, EPI_ISL_2441408, EPI_ISL_2441409, EPI_ISL_2441410, EPI_ISL_2441411, EPI_ISL_2441412, EPI_ISL_2441413, EPI_ISL_2441414, EPI_ISL_2441416, EPI_ISL_2441417, EPI_ISL_2441418, EPI_ISL_2441419, EPI_ISL_2441420, EPI_ISL_2441421, EPI_ISL_2441423, EPI_ISL_2441424, EPI_ISL_2441425, EPI_ISL_2441427, EPI_ISL_2441428, EPI_ISL_2441429, EPI_ISL_2441430, EPI_ISL_2441431, EPI_ISL_2441432, EPI_ISL_2441433, EPI_ISL_2441434, EPI_ISL_2441435, EPI_ISL_2441436, EPI_ISL_2441437, EPI_ISL_2441438, EPI_ISL_2441439, EPI_ISL_2441441			
see above	CSIR-National Environmental Engineering Research Institute	CSIR-Centre for Cellular and Molecular Biology-INSACOG	Amareshwar Vodapalli; Ara Sreenivas; Archana Bharadwaj Siva; B Himasri; Divya Tej Sowpati; Karthik Bharadwaj Tallapaka; Krishna Khairnar; Lamuk Zaveri; Onkar Kulkarni; Payel Mukherjee; Priya Nurkurthy; Rakesh K Mishra; Shreekant Verma; Sofia Banu; Sumedha Avadhanula; Tulasi Nagabandi; Valli Nagalakshmi Undamatla; Vidhyadhari Methuku
EPI_ISL_2790246	Cabinet Priv	Institut de Pathologie et Genetique (IPG)	Jérémie Gras; Pascale Hilbert
EPI_ISL_935782	Cadham Provincial laboratory	National Microbiology Laboratory (NML)	Anna Majer; Anneliese Landgraff; CanCOGeN's metadata curation team; Darian Hole; David Alexander; Elsie Grudeski; Gary Van Domselaar; Grace Seo; Jared Bullard; Jennifer Tanner; Kerry Dust; Kirsten Biggar; Madison Chapel; Morag Graham; Natalie Knox; Nathalie Bastien; Paul Van Caeseele; Philip Mabon; Public Health Agency of Canada CanCOGeN team; Rhiannon Huzarewich; Russell Mandes; Shari Tyson; Timothy Booth; Yan Li
EPI_ISL_1096348, EPI_ISL_1098602, EPI_ISL_1098604, EPI_ISL_1532800	Cambodian National Public Health Laboratory, National Institute of Public Health	Virology Unit, Institut Pasteur du Cambodge	Chau Darapheak; Chin Savuth; Erik A Karlsson; Kraing Sidonn; Leakhena Pum; Ly Sovann; Sokhoun Yann; Teyputita Ou; Veasna Duong; Yi Sengdoeurn
EPI_ISL_907094, EPI_ISL_907105	Cancer Biology Department, National Cancer Institute	Cancer Biology Department, National Cancer Institute	A.A.; A.E.; A.N.; Abouelhoda, M.; Ahmed; Bahnassy; Elhosieny; F.W.; Gad; H.K.; Hafez; Hamdy; M.G.; M.M.; M.S.; O.S.; Sedawy; Soliman; Soliman, L.; Zekri
EPI_ISL_1300646, EPI_ISL_1300654	Cantonal Hospital Zenica; Department of Microbiological Diagnostics	Clinical Center, University of Sarajevo; Unit for Clinical Microbiology	Amela Dedeić-Ljubović; Edina Zahirović; Golubinka Boshevska; Irma Salimović-Bešić; Maja Kuzmanovska; Sandra Vegar-Zubović; Sebija Izetbegović; Suzana Arapčić
EPI_ISL_2341788, EPI_ISL_2341806, EPI_ISL_2341923	Capital Hospital, Bhubaneswar	Institute of Life Sciences - INSACOG	Ajay Parida; Amol M. Kanampalliwar; Arup Ghosh; Atimukta Jha; INSACOG Consortium; Omprakash Shiriwas; Punit Prasad; Rajeeb Swain; Rupesh Dash; Safal Walia; Sana Fatma; Shifu Aggarwal; Sunil K. Raghav
EPI_ISL_1663491	Capital hospital, Bhubaneswar	Institute of Life Sciences - INSACOG	Ajay Parida; Amol M. Kanampalliwar; Arup Ghosh; Atimukta Jha; INSACOG Consortium; Punit Prasad; Rajeeb Swain; Rupesh Dash; Safal Walia; Shifu Aggarwal; Sunil K. Raghav
EPI_ISL_2225271	Caribbean Public Health Agency	Carrington Lab, Department of PreClinical Sciences, Faculty of Medical Sciences, The University of the West Indies	Adesh Ramsubhag; Arianne Brown-Jordan; Avery Hinds; Chinna Chinnadurai; Christine V. F. Carrington; Christopher Oura; Gabriel Escobar; Jaya Jayaraman; Jerome Foster; Karla Georges; Marsha Ivey; Naresh Nandram; Narine Singh; Nikita S. D. Sahadeo; Nuno Faria; Oliver Pybus; Rahul Naidu; Rajini Haraksingh; Risha Singh; Sarah Hill; Stanley Giddings; SueMin Nathaniel; Vernie Ramkissoon
EPI_ISL_2227348, EPI_ISL_2227349, EPI_ISL_2232351, EPI_ISL_2232359, EPI_ISL_2380086	Center of Scientific Excellence for Influenza Viruses (CSEIV), National Research Centre	Center of Scientific Excellence for Influenza Viruses (CSEIV), National Research Centre	Ahmed E Kayed; Ahmed El-Taweel; Ahmed Kandeil; Ahmed Mostafa; Amal Naguib; Ghazi Kayali; M K Khalifa; Mahmoud Shehata; Mina Kamel; Mohamed Ahmed Ali; Mohamed El Sayes; Mohamed Hassany; Mokhtar Gomaa; Nancy M. El Guindy; Noura M Abo Shama; Omnia Kutkat; Rabeh El-Shesheny; Richard Webby; Sara Mahmoud; Shymaa S Ahmed; Wael Roshdy; Yassmin Moatasim
EPI_ISL_815257, EPI_ISL_815352	Centogene	Centogene	Krishna Kumar Kandaswamy; Peter Bauer; Vivi Hue-Trang Lieu
EPI_ISL_1843777, EPI_ISL_1843874, EPI_ISL_1843894, EPI_ISL_2123120, EPI_ISL_2123158, EPI_ISL_2123187, EPI_ISL_2123202, EPI_ISL_2123224, EPI_ISL_2123237, EPI_ISL_2123266, EPI_ISL_2123293, EPI_ISL_2123341, EPI_ISL_2123353			
see above	Centogene; Dr. Bauer Laboratoriums GmbH	Robert Koch Institute	
EPI_ISL_794593, EPI_ISL_794594	Central Laboratories, Egyptian Ministry of Health and Population	Central Laboratories, Egyptian Ministry of Health and Population	A.E.; Ali; El Guindy; El Sayes; El Taweel, A.; El-Shesheny, R.; Gomaa, M.; Kamel; Kandeil, A.; Kayali, G.; Kayed; Khalifa; Kutkat, O.; M.A.; M.K.; M.N.; Mahmoud; Mahrous, N.; Moatasim, Y.; Mostafa, A.; N.M.; Naguib, A.; Roshdy; S.H.; Saleh, M.; Shawky, S.; Shehata, M.; W.H.
EPI_ISL_2463881, EPI_ISL_2464010	Central Public Health Lab, National Public Health Organization	Central Public Health Lab, National Public Health Organization	Gregory Spanakos et al; Kyriaki Tryfinopoulou
EPI_ISL_2233780	Central Public Health Laboratory, National Public Health Organization	Greek Genome Center, Biomedical Research Foundation of the Academy of Athens (BRFAA)	Dimitrios Thanos; Emmanouil Athanasiadis; Giannis Vatsellas; Katerina Zoi; Kyriaki Tryfinopoulou; Theodoros Loupis
EPI_ISL_1222766	Central Scientific Research Department, Gomel State Medical University,	Laboratory of Genomics and Bioinformatics of the Forest Research Institute of the NAS of Belarus	A.A.; A.S.; Baranov; E.V.; I.O.; Kiryanov; L.V.; O.V.; O.Y.; Osipkina; P.S. and Mozharovskaya; Padutov; Panteleev; S.V.; Shaforost; Stoma; V.E.; Voropaev; Zyatskov
EPI_ISL_885143	Central public health laboratory	Molecular Diagnostics Department, Central public health laboratory	Dalia, F.; Dler, H.; Dlshad, H.; F.-A.; Fahmi, A.; Furat, S.; Hemdad, A.; Hemn; M. and Idrees, H.; Mohsen, A.; Sharmeen
EPI_ISL_2141845	Centrala Laboratorija	Latvian Biomedical Research and Study Centre	Davids Fridmanis; Elina Dimina; Guntars Zarins; Ivars Silamikelis; Jana Osite; Janis Klovins; Janis Pjalkovskis; Jurijs Perevoscikovs; Kaspars Megnis; Laila Silamikele; Lauma Freimane; Laura Ansone; Liga Birzniece; Marta Priedite; Monta Ustinova; Nikita Zrelovs; Uga Dumpis; Una Krumina; Vita Rovite
EPI_ISL_1760556	Centre Hospitalier Universitaire Mère-Enfant, Fondation Jeanne Ebori (CHUMEFJE)	Centre de recherches médicales de Lambaréné (CERMEL)	Ayola A. Adegnika; Ayong Moure; Bertrand Lell; Bénédicte Ndeboko; Emilio Skarwan; Georgelin Nguema Ondo; Gédéon P. Manouana; Haruka Abe; Jiro Yasuda; Joel Fleury Djoba Siawaya; Rodrigue Bikangui; Rotimi Myrabelle Avome Houechenou; Samira Zoa-Assoumou; Yuri Ushijima
EPI_ISL_1523254	Centre Hospitalier du Nord	Laboratoire national de sante, Microbiology, Microbial Genomics Platform	Anke Wienecke-Baldacchino; Catherine Ragimbeau; Fatiha Boulmerka; Fatu Djabi; Jessica Tapp; Lise Pignon; Raoul Salmon; Tamir Abdelrahman
EPI_ISL_788936	Centre de Recherche et de Formation en Infectiologie Guinée	TransVIHMI, IRD/INSERM/Monpellier University	Abdoul Karim SOUMAH; Abdoulaye TOURE; Ahidjo AYOUBA; Alimou CAMARA; Alpha Kabinet KEITA; Bouna Yatassaye; Christelle BUTEL; Eric DELAPORTE; Jean-louis MONEMOU; Joel KOIVOGUI; Kaba KOUROUMA; Laetitia SERRANO; Mamadou Bhoye KEITA; Mamadou Saliou BAH; Mamadou Saliou SOW; Mandiou DIAKITE; Martine PEETERS; Moriba POVOGUI; Penda Malhado DIALLO; Sakoba KEITA
EPI_ISL_539573, EPI_ISL_539574	Centre de Recherches Medicales de Lambarene (CERMEL)	Department of Emerging Infectious Diseases, Institute of Tropical Medicine, Nagasaki University	Akim A. Adegnika; Bertrand Lell; Haruka Abe; Jiro Yasuda; Rodrigue Bikangui; Yuri Ushijima
EPI_ISL_1913034, EPI_ISL_1913035, EPI_ISL_1913050, EPI_ISL_1913070, EPI_ISL_2036078, EPI_ISL_2036080, EPI_ISL_2036089, EPI_ISL_2036198, EPI_ISL_2036199, EPI_ISL_2036270, EPI_ISL_2036273, EPI_ISL_2097231, EPI_ISL_2156768, EPI_ISL_2156769, EPI_ISL_2156795, EPI_ISL_2156796, EPI_ISL_2156809, EPI_ISL_2156822, EPI_ISL_2156825, EPI_ISL_2424138, EPI_ISL_2424157, EPI_ISL_2424160, EPI_ISL_2434975, EPI_ISL_2434980, EPI_ISL_2442276, EPI_ISL_2442277, EPI_ISL_2442338			
see above	Centre de Recherches Médicales de Lambaréné (CERMEL)	Centre de Recherches Médicales de Lambaréné (CERMEL)	Anicet Mouity Matoumba; Bertrand Lell and Ayola Akim Adegnika; Georgelin Nguema Ondo; Gédéon Prince Manouana; Jean Bernard Lekana-Douki; Joël-Fleury Djoba Siawaya; Michel Ngonga Dikongo; Moustapha Nzamba Maloum; Rodrigue Bikangui; Sam O'neilla Oye Bingono; Samira Zoa Assoumou; Srinivas reddy Pallerla; Steffen Borrmann; Thirumalaisamy P. Velavan
EPI_ISL_2355227	Centre for Clinical Infection and Diagnostics Research and Genomics Innovation Unit, Guy's and St. Thomas' NHS Trust	COVID-19 Genomics UK (COG-UK) Consortium	Ali Raza Awan; Chloe Fisher; Jonathan Edgeworth; Luke Snell; Penny Cliff; Rahul Batra
EPI_ISL_2106967	Centre for DNA Fingerprinting and Diagnostics	CDFD -INSACOG	Ashwin Dalal; Asmita Gupta; Divya Vashisht; Murali Bashyam; Pratyusha Bala; Vinay Donipadi
EPI_ISL_1730910, EPI_ISL_1730922, EPI_ISL_1833278, EPI_ISL_1980210	Centre for Enzyme Innovation, University of Portsmouth / Translational Research Laboratory, Portsmouth Hospitals NHS Trust	COVID-19 Genomics UK (COG-UK) Consortium	Angela Beckett; Christopher Fearn; Kate Cook; Katie Loveson; Salman Goudarzi; Samuel Robson; Scott Elliott; Sharon Glaysher
EPI_ISL_2240664, EPI_ISL_2624974, EPI_ISL_2625061, EPI_ISL_2708320, EPI_ISL_2708331, EPI_ISL_2708385	Centre for Enzyme Innovation, University of Portsmouth / Translational Research Laboratory, Portsmouth Hospitals NHS Trust / Hampshire Hospitals NHS Foundation Trust / Bournemouth University / University Hospitals Dorset NHS Foundation Trust / Brighton and Sussex University Hospitals NHS Trust / Dartford and Gravesham NHS Trust / Isle of Wight NHS Trust / Maidstone and Tunbridge Wells NHS Trust / University Hospital Southampton NHS Foundation Trust	COVID-19 Genomics UK (COG-UK) Consortium	Adhyana Mahanama; Allyson Lloyd; Andrew Butt; Angela Beckett; Anibolina Castigador; Anna Mantzaouratou; Anoop Chauhan; Ben Macklin; Benjamin Cogger; Buddhini Samaraweera; Cassandra Malone; Christopher Fearn; Claire Thomas; Dorian Crudgington; Eleri Wilson-Davies; Emanuela Pelosi; Emily Macnaughton; Helen Umpleby; Helen Wheeler; Jacqui Prieto; Jonnie Seymour; Karen Withell; Kate Cook; Katie Loveson; Kelly Bicknell; Kevin Tucker; Kordo Saeed; Liz Sheridan; Magdalena Barrow; Matthew Harvey; Mohammed Hassan-Ibrahim; Nathan Moore; Nick Cortes; Rachael Jeremiah; Robert Impey; Salman Goudarzi; Samirakhon Raupova; Samuel Robson; Sarah Buchan; Sarah Jeremiah; Sarah Wyllie; Scott Elliott; Sharon Glaysher; Siona Silvieri; Stephen Aplin; Stephen Kidd; Thea Sass

EPI_ISL_408976, EPI_ISL_412975, EPI_ISL_413214	Centre for Infectious Diseases and Microbiology Laboratory Services	NSW Health Pathology - Institute of Clinical Pathology and Medical Research; Westmead Hospital; University of Sydney	Carter I; Chen SC; Eden J-S; Holmes EC; Kok J and Dwyer DE for the 2019-nCoV Study Group; Kok J and Dwyer DE for the 2019-nCoV Study Group*; Maddocks S; O'Sullivan MV; Rahman H; Rockett R; Sadsad R; Sintchenko V
EPI_ISL_837555, EPI_ISL_837560, EPI_ISL_837571, EPI_ISL_837572, EPI_ISL_837573, EPI_ISL_837576, EPI_ISL_837577, EPI_ISL_837578			
see above	Centro Nacional de Enfermedades Tropicales (CENETROP)	Laboratory of Respiratory Viruses and Measles, Oswaldo Cruz Institute, FIOCRUZ	Ana Carolina Mendonca; Anna Carolina Paixao; Cinthia Avila; Fernando Motta; Luciana Appolinario; Marilda Siqueira on behalf of the Fiocruz COVID-19 Genomic Surveillance Network; Paola Resende; Roxana Loayza
EPI_ISL_2274030, EPI_ISL_2274031, EPI_ISL_2274032, EPI_ISL_2274033, EPI_ISL_2274037, EPI_ISL_2274038, EPI_ISL_2274039			
see above	Centro Nacional de Enfermidades Tropicales (CENETROP)	Laboratory of Respiratory Viruses and Measles, Oswaldo Cruz Institute, FIOCRUZ	Alice Sampaio Rocha; Ana Carolina Mendonca; Anna Carolina Paixao; Cinthia Avila; Elisa Cavalcante Pereira; Fernando Motta; Luciana Appolinario; Marilda Siqueira on behalf of the Fiocruz COVID-19 Genomic Surveillance Network; Paola Resende; Renata Serrano Lopes; Roxana Loayza; Taina Venas
EPI_ISL_2339879, EPI_ISL_2339896, EPI_ISL_2339897, EPI_ISL_2339909	Centro de Estudio de Enfermedades Autoinmunes (CREA), Universidad del Rosario, Bogota, Colombia	Centro de Investigaciones en Microbiología y Biotecnología-UR (CIMBIUR), Facultad de Ciencias Naturales, Universidad del Rosario, Bogotá, Colombia	Carolina Ramírez-Santana; Gustavo Salguero; Juan David Ramírez; Juan Esteban Gallo; Juan-Manuel Anaya; Luz H. Patiño; Marina Muñoz; Nathalia Ballesteros; Sergio Castañeda
EPI_ISL_2612391	Centro de Infectologia Charles Mérieux/ Laboratório Rodolphe Mérieux, FUNDHACRE	Bioinformatics Laboratory / LNCC	Alessandra P Lamarca; Alexandra L Gerber; Ana Paula de C Guimarães; Ana Tereza R Vasconcelos; Andreas Stocker; Cirley Maria de Oliveira Lobato; Douglas Terra Machado; Luiz Fellype Alves de Souza; Luiz G P de Almeida; Ronaldo da Silva F Jr
EPI_ISL_2007473	Centro de Investigaciones Básicas y Aplicadas, UNNOBA	Área de Secuenciación del Laboratorio de Virología del Hospital de Niños Dr. Ricardo Gutierrez on behalf of 'Proyecto Argentino Interinstitucional de genomica de SARS-CoV-2' (PAIS Consortium)	Acuña; Alexay; Bioq. Lucía Romano; D; Dr. Rodrigo Hernández del Pin; Dra. Alejandra Brandone y Dra. Natalí Bagnis; Dra. Carolina Cristina; Dra. Fiorella Spinelli; Dra. Gianina Demarchi; Dra. InaSevic; Dra. Laura Alaniz; Dra. Laura Palumbo; Dra. María Gracia Balbi; Dra. Virginia Pasquinelli; Est. Lorenzo Morro; Goya; LE; Lic. Agustina Chimento; Lic. Alejandra Fernández; Lic. Alejandro Moroni; Lic. Angela Barbero y Lic. Laureano Español; Lic. Antonella Icardi; Lic. Chiara Cassarini; Lic. Daiana Vitale; Lic. Micaela Castro; Lic. Nadia Bonadeo; Lic. Paolo Rosales; Lic. Sofia Perrone; Lic. Sofia Valla; Lusso; M; MI; Nabaes Jodar; Natale; S; Tc. Gastón Villafañe; Tc. Natalia Menite; Valinotto; Viegas, M.
EPI_ISL_944637	Centro de Investigaciones en Microbiología y Biotecnología-UR (CIMBIUR), Facultad de Ciencias Naturales, Universidad del Rosario, Bogotá, Colombia	Centro de Investigaciones en Microbiología y Biotecnología-UR (CIMBIUR), Facultad de Ciencias Naturales, Universidad del Rosario, Bogotá, Colombia Instituto Nacional de Salud, Bogotá, Colombia Icahn School of Medicine at Mount Sinai, New York, USA	Adriana van de Guchte; Alberto Paniz-Mondolfi; Ana S. Gonzalez-Reiche; Carolina Hernández; Emilia Mia Sordillo; Hala Alejel Alshammary; Harm van Bakel; Jayeeta Dutta; Juan David Ramírez; Luz Helena Patiño; Marina Muñoz; Matthew M. Hernandez; Nathalia Ballesteros; Sergio Gomez; Viviana Simon; Zenab Khan
EPI_ISL_1396437, EPI_ISL_1396469, EPI_ISL_1396475, EPI_ISL_1396509, EPI_ISL_1396511	Centro de Tecnología en Salud Pública de la Universidad Nacional de Rosario	Laboratorio Mixto de Biotecnología Acuática (LMBA) on behalf of 'Proyecto Argentino Interinstitucional de genomica de SARS-CoV-2' (PAIS Consortium)	Adriana Giri; Agustina Cerri; Ana Cavatorta; Ana Paletta; Diego Chouhy; Elisa Bolatti; Elizabeth Tapia (argenTAG); Federico Remes Lenicov; Flavio Spetale; Gastón Viarengo; Ignacio García Labari; Javier Murillo; Joaquín Ezpeleta; Julian Acosta; Laura Angelone; Leandro Ciappina; María Re; Pablo Casal; Pilar Bulacio; Silvana Spinelli; Silvia Arranz; Sofía Lavista Llanos; Vanina Villanova; Victoria Posner
EPI_ISL_770039	Centrālā Laboratorija	Latvian Biomedical Research and Study Centre	Ivars Silamiķelis; Jana Osīte; Jānis Kloviņš; Jānis Pjalkovskis; Kaspars Megnis; Marta Priedīte; Monta Ustinova; Stella Lapiņa; Uga Dumpis; Vita Rovīte; Ņikita Zrelovs
EPI_ISL_1590741	Centrālā Laboratorija; Eurofins Genomics Europe Sequencing GmbH	Riga East University Hospital-National Microbiology Reference Laboratory; Eurofins Genomics Europe Sequencing GmbH	Arzu Algulieva; Diāna Dusacka; Dārta Pūpola; Ilva Pole; Jana Osīte; Reinis Vangravs; Reinis Zeltmatis; Sergejs Nikisins; Stella Lapiņa; Ģirts Šķenders
EPI_ISL_501922	Centrālā laboratorija	Latvian Biomedical Research and Study Centre	Ivars Silamiķelis; Jana Osīte; Jānis Kloviņš; Kaspars Megnis; Marta Priedīte; Monta Ustinova; Stella Lapiņa; Uga Dumpis; Vita Rovīte; Ņikita Zrelovs
EPI_ISL_2646266	Cerballiance-IDF	Cerba lab	Aude Lessenne; Bénédicte Roquebert; Emmanuel Lecorche; Kader Merah; Laura Verdurme; Patrice Herisson; Sabine Trombert-Paolantoni; Stéphanie Haïm-Boukobza; Thierry Collin
EPI_ISL_2504138, EPI_ISL_2504140, EPI_ISL_2504141, EPI_ISL_2504142, EPI_ISL_2504143	Chantal Biya International Reference Centre (CIRCB)	International Centre for Genetic Engineering and Biotechnology (ICGEB) and ARGO Open Lab for Genome Sequencing	Alessandro Marcello; Alexis Ndjolo; Celine Nkenfou; Collins Chenwi; Danilo Licastro; Desire Takou; Emanuele Orsini; Grace Angong Beloumou; Joseph Fokam; Ndine Fainguem; Simeone Dal Monego
EPI_ISL_753708, EPI_ISL_753720, EPI_ISL_753986	Charité Universitätsmedizin Berlin, Institut für Virologie/Labor Berlin	Charité Universitätsmedizin Berlin, Institut für Virologie	Barbara Mühlemann; Christian Drosten; Julia Schneider; Jörn Beheim-Schwarzbach; Talitha Veith; Terry Jones; Victor M Corman
EPI_ISL_2337257	Chiba Prefectural Institute of Public Health	Pathogen Genomics Center, National Institute of Infectious Diseases	Kentaro Itokawa; Makoto Kuroda; Masanori Hashino; Rina Tanaka; Tsuyoshi Sekizuka
EPI_ISL_2342019, EPI_ISL_2342110, EPI_ISL_2342128, EPI_ISL_2342354	Chief District Medical Office, Cuttack	Institute of Life Sciences - INSACOG	Ajay Parida; Amol M. Kanampalliwar; Arup Ghosh; Atimukta Jha; INSACOG Consortium; Omprakash Shiriwas; Punit Prasad; Rajeeb Swain; Rupesh Dash; Safal Walia; Sana Fatma; Shifu Aggarwal; Sunil K. Raghav
EPI_ISL_700346, EPI_ISL_1752692, EPI_ISL_2445998, EPI_ISL_2692860	Child Health Research Foundation	Child Health Research Foundation	Afroza Akter Tanni; Arif Mohammad Tanmoy; CHRF Bangladesh Genomics Team; Md Hafizur Rahman; Roly Malaker; Samir K Saha; Senjuti Saha; Sharmistha Goswami; Syed Muktadir Al Sium
EPI_ISL_2162123, EPI_ISL_2162124, EPI_ISL_2162196, EPI_ISL_2162209	Children's City Clinical Infectious Diseases Hospital	Reference laboratory for the control of viral infections	Aidar Usserbayev; Aknur Mutaliyeva; Andrey Komissarov; Artem Fadeev; Azamat Kenessov; Bekzhan Maikotov; Gaukhar Nussupbayeva; Madina Tleubergenova; Maria Pisareva; Nazym Tleumbetova
EPI_ISL_1970348, EPI_ISL_1970349	Chongqing International Travel Health Care Center	Chongqing Municipal Center for Disease Control and Prevention	Dong Wang; Haiyan Wen; Hua Ling; Lan Zhou; Mingyue Wang; Rong Rong; Sheng Ye; Shuang Chen; Wenge Tang; Xingdan Luo; Yanqing Peng; Yun Tang; Zhangping Tan; Zhaohui Deng , Shuang Chen; Zhen Yu
EPI_ISL_1970347	Chongqing Yuzhong District Center For Disease Control And Prevention	Chongqing Municipal Center for Disease Control and Prevention	Hua Ling; Jiaqi Li; Mingyue Wang; Rong Rong; Sheng Ye; Shuang Chen; Wenge Tang; Yingbing Zhou; Yun Tang; Yuyue Luo; Zhangping Tan; Zhen Yu
EPI_ISL_1398367	Cianjur Public Health	West Java Health Laboratory; School of Life Sciences and Technology, Institut Teknologi Bandung	Azzania Fibriani; Cut Nur Cinthia Alamanda; Ema Rahmawati; Isak Solihin; Kamila Tania; Karimatu Khoirunnisa; Miftahul Faridl; Rifky Waluyajati Rachman; Rini Robiani; Ryan Bayusantika Ristandi
EPI_ISL_2162172	City Clinical Infectious Diseases Hospital named after I.S. Zhekenova	Reference laboratory for the control of viral infections	Aidar Usserbayev; Aknur Mutaliyeva; Andrey Komissarov; Artem Fadeev; Azamat Kenessov; Bekzhan Maikotov; Gaukhar Nussupbayeva; Madina Tleubergenova; Maria Pisareva; Nazym Tleumbetova
EPI_ISL_1424939	Clalit Health Services Laboratories, Israel	Stern Lab	Stern Lab
EPI_ISL_462461, EPI_ISL_462474	Clinical Center, University of Sarajevo	Charite Universitatsmedizin Berlin, Institute of Virology	Almedina Hadzihasanovic-Moro; Amela Dedeic-Ljubovic; Barbara Muehlemann; Christian Drosten; Irma Salimovic-Besic; Jorn Beheim-Schwarzbach; Julia Schneider; Selma Mutevelic; Suzana Arapcic; Talitha Veith; Terry Jones; Victor M Corman
EPI_ISL_1240073, EPI_ISL_1300645, EPI_ISL_1300647, EPI_ISL_1300650, EPI_ISL_1300659	Clinical Center, University of Sarajevo; Unit for Clinical Microbiology	Clinical Center, University of Sarajevo; Unit for Clinical Microbiology	Amela Dedeić-Ljubović; Edina Zahirović; Golubinka Boshevska; Irma Salimović-Bešić; Maja Kuzmanovska; Sandra Vegar-Zubović; Sebija Izetbegović; Suzana Arapčić
EPI_ISL_2426148, EPI_ISL_2426149, EPI_ISL_2426151, EPI_ISL_2426153, EPI_ISL_2426154, EPI_ISL_2426155, EPI_ISL_2426156, EPI_ISL_2426159, EPI_ISL_2426160, EPI_ISL_2426161, EPI_ISL_2426162, EPI_ISL_2426163, EPI_ISL_2426164, EPI_ISL_2426165, EPI_ISL_2426166, EPI_ISL_2426167, EPI_ISL_2426168, EPI_ISL_2426170, EPI_ISL_2426171, EPI_ISL_2426175, EPI_ISL_2426177, EPI_ISL_2426178, EPI_ISL_2426179, EPI_ISL_2426180, EPI_ISL_2426182, EPI_ISL_2426183, EPI_ISL_2426186, EPI_ISL_2426187			
see above	Clinical Virology Laboratory, Institute of Liver and Biliary Sciences	ILBS - IGIB	Amit Pandey; Anurag Agrawal; Aparna S; Bharathram Uppili; Chhagan Bihari Sharma; Ekta Gupta; Manish Kumar; Mohammed Faruq; Neha Jha; Partha Chattopadhyay; Pooja Sharma; Pratibha Kale; Priti Devi; Priyanka Mehta; Rajesh Pandey; Ranjeet Maurya; Sarfaraz Alam; Sheeba Saifi; Sheetalnath Rooge; Shiv Kumar Sarin
EPI_ISL_2427381, EPI_ISL_2427389, EPI_ISL_2427396, EPI_ISL_2427399, EPI_ISL_2492437, EPI_ISL_2492438, EPI_ISL_2492439, EPI_ISL_2626062, EPI_ISL_2626067, EPI_ISL_2626068, EPI_ISL_2626072, EPI_ISL_2638046, EPI_ISL_2778098			
see above	Clinique Saint-Pierre Ottignies	UCLouvain/IREC/MBLG	Benoit Kabamba Mukadi; Bertrand Bearzatto; Jean Ruelle; Ophélie Simon
EPI_ISL_1935601, EPI_ISL_2348848, EPI_ISL_2348862, EPI_ISL_2427343, EPI_ISL_2427353, EPI_ISL_2492429, EPI_ISL_2492430, EPI_ISL_2492431, EPI_ISL_2492432, EPI_ISL_2492433, EPI_ISL_2492434, EPI_ISL_2492435, EPI_ISL_2492436, EPI_ISL_2510682, EPI_ISL_2626010, EPI_ISL_2626012, EPI_ISL_2626015, EPI_ISL_2626021, EPI_ISL_2626023, EPI_ISL_2626028, EPI_ISL_2626031, EPI_ISL_2626032, EPI_ISL_2626037, EPI_ISL_2626083, EPI_ISL_2626084, EPI_ISL_2626085, EPI_ISL_2626086, EPI_ISL_2626087, EPI_ISL_2626088, EPI_ISL_2626089, EPI_ISL_2626090, EPI_ISL_2626091, EPI_ISL_2626092, EPI_ISL_2626093, EPI_ISL_2626094, EPI_ISL_2626095, EPI_ISL_2626096, EPI_ISL_2778018, EPI_ISL_2778019, EPI_ISL_2778020, EPI_ISL_2778022, EPI_ISL_2778024, EPI_ISL_2778025, EPI_ISL_2778026, EPI_ISL_2778028, EPI_ISL_2778029, EPI_ISL_2778032, EPI_ISL_2778037, EPI_ISL_2778040, EPI_ISL_2778041, EPI_ISL_2778047, EPI_ISL_2778048, EPI_ISL_2778051, EPI_ISL_2778053, EPI_ISL_2778055, EPI_ISL_2778057, EPI_ISL_2778063, EPI_ISL_2778064, EPI_ISL_2778066, EPI_ISL_2778069, EPI_ISL_2778071, EPI_ISL_2778072, EPI_ISL_2778074, EPI_ISL_2778079, EPI_ISL_2778081, EPI_ISL_2778082, EPI_ISL_2778090			
see above	Cliniques universitaires Saint-Luc	UCLouvain/IREC/MBLG	Benoit Kabamba Mukadi; Bertrand Bearzatto; Eleonore Ngyuvula; Jean Ruelle; Nicolas Pinte; Ophélie Simon
EPI_ISL_632262, EPI_ISL_678262, EPI_ISL_678272, EPI_ISL_681299, EPI_ISL_681301, EPI_ISL_681309, EPI_ISL_682303, EPI_ISL_1660421, EPI_ISL_1660425, EPI_ISL_1660426, EPI_ISL_1660427, EPI_ISL_1660429, EPI_ISL_1660430, EPI_ISL_1660432, EPI_ISL_1660439, EPI_ISL_1660442, EPI_ISL_1660445, EPI_ISL_1660447, EPI_ISL_1660448, EPI_ISL_1660450, EPI_ISL_1660456, EPI_ISL_1660458, EPI_ISL_1660459, EPI_ISL_1660463, EPI_ISL_1660464, EPI_ISL_1660467, EPI_ISL_2466673, EPI_ISL_2466677, EPI_ISL_2622083, EPI_ISL_2622084, EPI_ISL_2622087			
see above	Communicable Disease Laboratory, Public Health Directorate	Communicable Disease Laboratory, Public Health Directorate	AlAbbas, Z.; AlHujairi, Z.; AlTaif, Z.; AlWasti, H.; Almoamen, G.; Altaif, Z.; Alwasti, H.; Marhoon, A.
EPI_ISL_510528	Communicable Disease Laboratory, Public Health Directorate	Communicable Disease Laboratory, Public Health Directorate	Al Wasti; H. and AlTaif, Z.
EPI_ISL_2469779, EPI_ISL_2632134	Corona-Testzentrum ifp Institut für Produktqualität GmbH	Robert Koch Institute	
EPI_ISL_1287369, EPI_ISL_2626562, EPI_ISL_2627292	Croatian Institute of Public Health	Croatian Institute of Public Health	Irena Tabain; Ivana Ferenčak
EPI_ISL_904028	DB Diagnosticos do Brasil	Laboratório de Parasitologia Médica - Instituto de Medicina Tropical - Universidade de São Paulo	Brazil-UK Centre for Arbovirus Discovery Diagnosis Genomics and Epidemiology (CADDE) Genomic Network - Instituto de Medicina Tropical
EPI_ISL_1636523	DNA Solution Ltd.	Genomic Research Lab, BCSIR	Abu Sayeed Mohammad Mahmud; Barna Goswami; Eshrar Osman; Iffat Jahan; Kazi Nadim Hasan; Md Firoz Kabir; Md. Abdul Khaleque; Md. Ahasan Habib; Md. Mizanur Rahman; Md. Murshed Hasan Sarkar; Md. Saddam Hossain; Md. Salim Khan; Mohammad Fazle Alam Rabbi; Mohammad Mohi Uddin; Mohammad Samir Uzzaman; Shahina Akter; Sharif Akhteruzzamani; Tanjina Akhter Banu

EPI_ISL_2725072	DNAGYN	LGBio - Laboratório de Genética & Biodiversidade	Alex Honda Bernardes; Amanda Alves de Melo; Aparecido Divino da Cruz; Cintia Pelegrineti Targueta de Azevedo Brito; Daniela de Melo e Silva; Elisangela de Paula Silveira Lacerda; Francyelli Mello Andrade; Juliana Santana de Curcio; Luiz Augusto Pereira; Marc Alexandre Duarte Gigonzac; Mariana Pires de Campos Telles; Ramilla dos Santos Braga; Renata de Oliveira Dias; Rhewter Nunes; Thais Cidália Vieira Gigonzac; Thais Guimarães Castro; Thays Millena Alves Pedroso
EPI_ISL_2728324	DSU HASSAN	INSACOG-KA, NIMHANS	Ananthapadmanabha Kotambail; Anita S Desai; Anson Kunjumon George; Chetan G K; Chitra Pattabiraman; Darshan Sreenivas; Ellango Ramasamy; Gautham Arunachal Udupi; Mahesh Kumar.C.S; Sony Sharma; V Ravi
EPI_ISL_2450343, EPI_ISL_2450344, EPI_ISL_2450345, EPI_ISL_2450346, EPI_ISL_2450347, EPI_ISL_2450348	DSU KOLAR	INSACOG-KA, NIMHANS	Ananthapadmanabha Kotambail; Anita S Desai; Anson Kunjumon George; Chetan G K; Chitra Pattabiraman; Darshan Sreenivas; Gautham Arunachal Udupi; V Ravi
EPI_ISL_2728349, EPI_ISL_2728351, EPI_ISL_2728352, EPI_ISL_2728354, EPI_ISL_2728359, EPI_ISL_2728360, EPI_ISL_2728361, EPI_ISL_2728362, EPI_ISL_2728363, EPI_ISL_2728364, EPI_ISL_2728366, EPI_ISL_2728370, EPI_ISL_2728375, EPI_ISL_2728380, EPI_ISL_2728381, EPI_ISL_2728384, EPI_ISL_2728385, EPI_ISL_2728388, EPI_ISL_2728389, EPI_ISL_2728390, EPI_ISL_2728391, EPI_ISL_2728392, EPI_ISL_2728396, EPI_ISL_2728398, EPI_ISL_2728399, EPI_ISL_2728400, EPI_ISL_2728401, EPI_ISL_2728406, EPI_ISL_2728408, EPI_ISL_2728409, EPI_ISL_2728412, EPI_ISL_2728414, EPI_ISL_2728415, EPI_ISL_2728417, EPI_ISL_2728418, EPI_ISL_2728419, EPI_ISL_2728420, EPI_ISL_2728421, EPI_ISL_2728424, EPI_ISL_2728425			
see above	DSU VIJAYAPURA	INSACOG-KA, NIMHANS	Ananthapadmanabha Kotambail; Anita S Desai; Anson Kunjumon George; Chetan G K; Chitra Pattabiraman; Darshan Sreenivas; Ellango Ramasamy; Gautham Arunachal Udupi; Mahesh Kumar.C.S; Sony Sharma; V Ravi
EPI_ISL_2100635, EPI_ISL_2100636, EPI_ISL_2100637, EPI_ISL_2100638, EPI_ISL_2100639, EPI_ISL_2100640, EPI_ISL_2100641, EPI_ISL_2100642, EPI_ISL_2100643, EPI_ISL_2100644, EPI_ISL_2100645, EPI_ISL_2100646, EPI_ISL_2100647, EPI_ISL_2100648, EPI_ISL_2100649, EPI_ISL_2100650, EPI_ISL_2100651, EPI_ISL_2100652, EPI_ISL_2100653, EPI_ISL_2100654, EPI_ISL_2100655, EPI_ISL_2100656, EPI_ISL_2100657, EPI_ISL_2100658, EPI_ISL_2100659, EPI_ISL_2100660, EPI_ISL_2100661, EPI_ISL_2100662, EPI_ISL_2100663, EPI_ISL_2100664, EPI_ISL_2100665, EPI_ISL_2100666, EPI_ISL_2100667, EPI_ISL_2100668, EPI_ISL_2100669, EPI_ISL_2100670, EPI_ISL_2100671, EPI_ISL_2100672, EPI_ISL_2105576, EPI_ISL_2105577, EPI_ISL_2105578, EPI_ISL_2105579, EPI_ISL_2105580, EPI_ISL_2105581, EPI_ISL_2106198			
see above	Delhi North District	CSIR-Institute of Genomics and Integrative Biology	Animesh Ray; Asangala Kamai; Ashwin Varadarajan; Ayush Goel; Bharathram Uppilli*; Devasish Desai; Manish Kumar; Mohammed Ahmed; Mohammed Faruq; Naveet Wig; Pooja Sharma*; Rajesh Pandey; Sarafaraz Alam; Saruchi Wadhwa; Satish Swain; Sheeba Saifi; Sushma Rajpoot; Umang Arora
EPI_ISL_470876, EPI_ISL_470877, EPI_ISL_481380, EPI_ISL_481483, EPI_ISL_763062, EPI_ISL_1791045, EPI_ISL_1791055, EPI_ISL_1914578, EPI_ISL_1914603, EPI_ISL_1914604, EPI_ISL_1914670, EPI_ISL_1914783, EPI_ISL_1914784, EPI_ISL_1914938, EPI_ISL_1921858, EPI_ISL_1921879, EPI_ISL_2319000, EPI_ISL_2319003			
see above	Department for Virology, Molecular Biology and Genome Research, R. G. Lugar Center for Public Health Research, National Center for Disease Control and Public Health (NCDC) of Georgia.	Department for Virology, Molecular Biology and Genome Research, R. G. Lugar Center for Public Health Research, National Center for Disease Control and Public Health (NCDC) of Georgia.	Adam Kotorashvili; Amiran Gamkrelidze.; Ana Papkiauri; Ann Machablishvili; Anna Kasradze; Davit Tsaguria; Ekaterine Khmaladze; Ekaterine Zangaladze; Ekaterine Zhghenti; Giorgi Gogoladze; Giorgi Tomashvili; Gvantsa Brachveli; Gvantsa Chanturia; Irma Burjanadze; Ketevan Sidamonidze; Khatuna Zakhashvili; Lela Sabadze; Lela Urushadze; Magda Dgebuadze; Maia Alkhazashvili; Mari Gavashelidze; Mariam Zakalashvili; Marine Murtskhvaladze; Meri Pantsulaia; Nato Kotaria; Nino Berishvili; Paata Imnadze; Roena Sukhiashvili; Tamar Jashiashvili; Tata Imnadze; Tea Tevdoradze
EPI_ISL_2412528, EPI_ISL_2413433, EPI_ISL_2431016, EPI_ISL_2666392, EPI_ISL_2667354, EPI_ISL_2784479	Department of Bacteria, Parasites and Fungi, Statens Serum Institut, Copenhagen, Denmark	Statens Serum Institut Bioinformatics and Microbial Genomics	Danish Covid-19 Genome Consortium
EPI_ISL_515094, EPI_ISL_515103	Department of Biochemistry, Cell and Molecular Biology	WACCBIP, University of Ghana	A.K.; Adu, B.; Amenga-Etego; Ampofo, W.; Amuzu; Anang; Arjarquah, A.; Asante, I.; Awandare; Bediako, Y.; Boatemaa, L.; Bonney, E.; Bonney, K.; C.M.; D.S.; Eshun, M.; G.A.; G.B.; J.K.; J.M.; Kotey, E.; Kumordjie, S.; Kyei; L.N.; Magnussen, V.; Morang'a; Mutungi; Ngoi; Quashie, P.; Tei-Maya, F.
EPI_ISL_884856	Department of Biochemistry, Cell and Molecular Biology, West African Centre for Cell Biology of Infectious Pathogens (WACCBIP), University of Ghana	Department of Biochemistry, Cell and Molecular Biology, West African Centre for Cell Biology of Infectious Pathogens (WACCBIP), University of Ghana	A.-K.; A.B.; Abass; Akoriyea; Amenga-Etego; Amoako, E.; Amuzu; Awandare; Bediako, Y.; Boakye; C.M.; D.S.; Diallo; G.A.; J.M.; Kibinge, N.; Kumi-Ansah, F.; L.N.; Magnussen, V.; Mohammed, A.; Morang'a; Ngoi; O.D.; Odoom, T.; Quashie, P.; S.K.; Tapela, K.; Tei-Maya, F.
EPI_ISL_678405, EPI_ISL_2003343, EPI_ISL_2426586, EPI_ISL_2612790, EPI_ISL_2612793, EPI_ISL_2685887, EPI_ISL_2685889, EPI_ISL_2728639			
see above	Department of Clinical Microbiology	GIGA Medical Genomics	Bouchra Boujemla; Claire Gourzonès; Cécile Meex; Keith Durkin; Laurent Gillet; Maria Artesi; Marie-Pierre Hayette; Nadine Cambisano; Nathalie Renotte; Olivier Ek; Pierrette Melin; Raphaël Boreux; Sébastien Bontems; Vincent Bours
EPI_ISL_2025798	Department of Clinical Microbiology and Center for Genomic Medicine, Rigshospitalet, Copenhagen, Denmark	Aalborg University	Danish Covid-19 Genome Consortium
EPI_ISL_1547372, EPI_ISL_1550506	Department of Genetic Engineering and Biotechnology, Shahjalal University of Science and Technology	Genomic Research Lab, BCSIR	Abu Sayeed Mohammad Mahmud; Ajit Ghosh; Barna Goswami; Eshrar Osman; G. M. Nurnabi Azad Jewel; G. M. Nurnabi Azad Jewel; Iffat Jahan; Md. Ahasan Habib; Md. Akkas Ali; Md. Asraful Jahan; Md. Fahmid Hossain Bhuiyan; Md. Hammadul Hoque; Md. Kamrul Islam; Md. Murshed Hasan Sarkar; Md. Nazmul Hasan; Md. Saddam Hossain; Md. Salim Khan; Md. Shamsul Haque Prodhan; Mohammad Mohi Uddin; Mohammad Samir Uzzaman; Shahina Akter; Tanjina Akhter Banu
EPI_ISL_610207, EPI_ISL_2378701, EPI_ISL_2378704	Department of Health Technology and Informatics, The Hong Kong Polytechnic University	Department of Health Technology and Informatics, The Hong Kong Polytechnic University	A.K.-L.; A.Y.-M.; B.H.-S.; B.K.-C.; B.P.-H.; C.T.-M.; Chan; Chau; Chen, H.; Cheng; D.C.; D.H.-K.; D.S.-H.; E.Y.-K.; E.Y.-W.; Fung; G.K.-H.; H.-C.; H.-Y.; H.W.-H.; Ho; J.S.-L.; J.Y.-W.; Jim; K.-T.; K.-Y.; K.K.; K.K.-G.; K.K.-S.; K.K.-W.; K.N.; K.S.-C.; K.S.-S.; L.-K.; Lai; Lam; Lao; Lee; Leung; Lo; Lu; Luk, K.; Luk, S.; Lung; M.-C.; M.C.-Y.; Mok; Ng; Que; R.A.; S.-C.; S.K.-Y.; S.P.; Shum; Siu; T.-L.; T.T.-L.; Tam; To; Tse, H.; V.C.-C.; W.-K.; W.-S.; W.C.; Wong; Wu; Y.W.-M.; Yam; Yau; Yeh; Yip; Yuen
EPI_ISL_1018120, EPI_ISL_1018280, EPI_ISL_1018313, EPI_ISL_1797626, EPI_ISL_2443057	Department of Health Technology and Informatics, The Hong Kong Polytechnic University	Department of Health Technology and Informatics, The Hong Kong Polytechnic University	Alan Ka-Lun Wu; Alex Yat-Man Ho; Barry Kin-Chung Wong; Chloe Toi-Mei Chan; David Ho-Keung Shum; Denise Sze-Hang Wong; Gilman Kit-Hang Siu; Hiu-Yin Lao; Hoi-Ching Jim; Jake Siu-Lun Leung; Kam-Tong Yip; Kenneth Siu-Sing Leung; Kingsley King-Gee Tam; Kitty Sau-Chun Fung; Kristine Luk; Lam-Kwong Lee; Miranda Chong-Yee Yau; Sandy Ka-Yee Chau; Shea Ping Yip; Tak-Lun Que; Timothy Ting-Leung Ng; Wing Cheong Yam; Wing-Hei Lo; Wing-Kin To; Yvette Wai-Man Lai
EPI_ISL_1920595	Department of Hygiene, Epidemiology and Medical Statistics, Medical School, National and Kapodistrian University of Athens	Central Public Health Laboratory, National Public Health Organization	Gkikas Magiorkinis et al
EPI_ISL_1425484, EPI_ISL_1941895, EPI_ISL_1941944	Department of Infectious Diseases, Kobe Institute of Health	Department of Infectious Diseases, Kobe Institute of Health	Kentaro Itokawa; Makoto Kuroda; Masanori Hashino; Noriko Nakanishi; Rina Tanaka; Ryohei Nomoto; Tomotada Iwamoto; Tsuyoshi Sekizuka
EPI_ISL_1209281, EPI_ISL_1209288, EPI_ISL_1495324	Department of Laboratory Medicine, Division of Clinical Virology, University of Medicine, Vienna	Bergthaler laboratory, CeMM Research Center for Molecular Medicine of the Austrian Academy of Sciences	Andreas Bergthaler; Anna Schedl; Bekir Erguner; Benedikt Agerer; Christoph Bock; Fabian Amman; Jan Laine; Lukas Endler; Maelle Le Moing; Martin Senekowitsch; Michael Schuster; Petr Triska; Thomas Penz
EPI_ISL_1678790, EPI_ISL_1678810	Department of Medical Microbiology & Infection prevention, Amsterdam University Medical Centers location AMC	Department of Medical Microbiology & Infection prevention, Amsterdam University Medical Centers location AMC	Fokla Zorgdrager; Janke Schinkel; Marcel Jonges; Matthijs Welkers; Menno de Jong; Robin van Houdt; Sebastien Matamoros; Sjoerd Rebers
EPI_ISL_2547336, EPI_ISL_2547337, EPI_ISL_2547339, EPI_ISL_2547429, EPI_ISL_2547522	Department of Medical Virology, School of Medicine Ahvaz Jundishapur University of Medical sciences	Genetics Research Center, University of Social Welfare and Rehabilitation Sciences	Ahmad Tavakoli; Azarakhsh Azaran; Farid yousefi; Hossein Najmabadi.; Kimia Kahrizi; Maryam Beheshtian; Marzieh Mohseni; Mohammad Farahmand; Mohsen Savaie; Seyed Mohammad Jazayeri; Zohreh Fattahi
EPI_ISL_497856, EPI_ISL_498271, EPI_ISL_1034423, EPI_ISL_1034489, EPI_ISL_1034665, EPI_ISL_1034714, EPI_ISL_2423555			
see above	Department of Microbiology, The University of Hong Kong	Department of Microbiology, The University of Hong Kong	Kelvin K.W. To; Kwok-Yung Yuen
EPI_ISL_1540503, EPI_ISL_1540522	Department of Microbiology, University Hospital Motol	Department of Microbiology, University Hospital Motol	Ales Briksi; Katerina Chuda; Katerina Polackova; Klara Krivankova; Miroslav Zajac; Ondrej Cinek; Pavel Drevinek; Petr Hubacek
EPI_ISL_2536157	Department of Molecular Diagnostic، Emam Ali Educational and Therapeutic Center , Alborz University of Medical Sciences, Karaj, Iran	Genetics Research Center, University of Social Welfare and Rehabilitation Sciences	Alireza Soleimani; Hossein Najmabadi.; Kimia Kahrizi; Maryam Beheshtian; Marzieh Mohseni; Mohammad Foad Abazari; Razieh Dowran; Saber Soltani; Shima Sadeghi; Zakiye Mokhames; Zohreh Fattahi
EPI_ISL_1164628, EPI_ISL_1164659, EPI_ISL_1164677, EPI_ISL_1164702, EPI_ISL_1164736	Department of Molecular Virology, Cyprus Institute of Neurology and Genetics	Department of Molecular Virology, Cyprus Institute of Neurology and Genetics	Anastasis Oulas; Andreas Hadjisavvas; Christina Christodoulou; Christina Tryfonos; Dana Koptides; Denise Alexandrou; George Krashias; George Spyrou; Jan Richter; Maria Loizidou; Mihalis Panayiotidis; Olga Kalakouta; Pavlos Fanis; Stavros Bashiardes
EPI_ISL_1730064, EPI_ISL_1730071, EPI_ISL_1730072, EPI_ISL_1832202, EPI_ISL_1832205, EPI_ISL_1832216, EPI_ISL_1832228, EPI_ISL_1974779, EPI_ISL_2722110, EPI_ISL_2722197, EPI_ISL_2722358, EPI_ISL_2722481, EPI_ISL_2722537, EPI_ISL_2722597, EPI_ISL_2733926			
see above	Department of Pathology, University of Cambridge	COVID-19 Genomics UK (COG-UK) Consortium	Aminu S. Jahun; Ian Goodfellow; Iliana Georgana; Martin D. Curran; Myra Hosmillo; Rhys Izuagbe; Surendra Parmar; William L. Hamilton; Yasmin Chaudhry
EPI_ISL_450209	Department of Virology	Department of Virology	Ackermann, N.; Antwerpen, M.; Bengs, K.; Berger, A.; Boehm, S.; Boehmer; Boender; Buchholz, U.; Cai, W.; Corman; D.V.; Dangel, A.; Drosten, C.; Eberle, U.; Fingerle, V.; Grahl, A.; Haas, W.; Hamouda, O.; Hoch, M.; Hoermansdorfer, S.; Ippisch, S.; Jones; Katz, K.; Konrad, R.; Liebl, B.; M.M.; Marosevic; Muehlemann, B.; Muller, N.; Poertner, K.; Protzer, U.; Reich, A.; Rexroth, U.; Schneider, J.; Sing, A.; T.C.; T.S.; Treis, B.; V.M.; Veith, T.; Walter, M.; Wicklein, B.; Woelfel, R.; Woudenberg, T.; Zapf, A.; Zeitlmann, N.; an der Heiden, M.
EPI_ISL_862065, EPI_ISL_862115, EPI_ISL_902774, EPI_ISL_1595794, EPI_ISL_1841715, EPI_ISL_1841947, EPI_ISL_1842089, EPI_ISL_1842217, EPI_ISL_2258787, EPI_ISL_2604325, EPI_ISL_2604421, EPI_ISL_2604692, EPI_ISL_2608769, EPI_ISL_2608773, EPI_ISL_2608785, EPI_ISL_2608883, EPI_ISL_2608938, EPI_ISL_2609354			
see above	Department of Virology and Immunology, University of Helsinki and Helsinki University Hospital, Huslab Finland	Department of Virology, Faculty of Medicine, University of Helsinki, Helsinki, Finland	Essi Korhonen; Hanna Jarva; Hanna Liimatainen; Hannimari Kallio-Kokko; Harri Kangas; Hussein Alburkat; Jenni Virtanen; Maija Lappalainen; Maija Suvanto; Olli Vapalahti; Pekka Ellonen; Phuoc Truong; Ravi Kant; Sari Hannula; Satu Kurkela; Teemu Smura; fathiah Zakham
EPI_ISL_2254718	Department of Virology, School of Medicine, Shiraz University of Medical Sciences, Shiraz, Iran	Genetics Research Center, University of Social Welfare and Rehabilitation Sciences	Hossein Najmabadi.; Kimia Kahrizi; Marzieh Mohseni; Moattari A; Moghadami M; Zohreh Fattahi
EPI_ISL_1858833, EPI_ISL_1860885, EPI_ISL_1861168, EPI_ISL_1865192, EPI_ISL_1866210, EPI_ISL_1868349, EPI_ISL_1868655, EPI_ISL_1868943, EPI_ISL_1869673, EPI_ISL_1870726, EPI_ISL_1871129, EPI_ISL_1871787, EPI_ISL_1872528, EPI_ISL_1873137, EPI_ISL_1874259, EPI_ISL_1875582, EPI_ISL_1875917, EPI_ISL_1877615, EPI_ISL_1881208, EPI_ISL_1881663, EPI_ISL_1885762, EPI_ISL_1885979, EPI_ISL_1888026, EPI_ISL_1890934, EPI_ISL_1893878, EPI_ISL_1896403, EPI_ISL_2023939, EPI_ISL_2024372, EPI_ISL_2024988, EPI_ISL_2024994, EPI_ISL_2025050, EPI_ISL_2025253, EPI_ISL_2025326, EPI_ISL_2025835, EPI_ISL_2026084, EPI_ISL_2026230, EPI_ISL_2026527, EPI_ISL_2026729, EPI_ISL_2026828, EPI_ISL_2027124, EPI_ISL_2027238, EPI_ISL_2027359, EPI_ISL_2027667, EPI_ISL_2027848, EPI_ISL_2028708, EPI_ISL_2028728, EPI_ISL_2028832, EPI_ISL_2299602			
see above	Department of Virus and Microbiological Special Diagnostics, Statens Serum Institut, Copenhagen, Denmark	Aalborg University	Danish Covid-19 Genome Consortium
EPI_ISL_614573, EPI_ISL_616194, EPI_ISL_616403, EPI_ISL_616601, EPI_ISL_622178,	Department of Virus and Microbiological Special Diagnostics, Statens Serum Institut, Denmark	Albertsen lab, Department of Chemistry and Bioscience, Aalborg University, Denmark	Danish Covid-19 Genome Consortia

EPI_ISL_622600			
EPI_ISL_1662291	Dept. Of Microbiology, Lt.. Baliram Kashyap Memorial Govt. Medical college, Dimrapal, Jagdalpur	Institute of Life Sciences - INSACOG	Ajay Parida; Amol M. Kanampalliwar; Arup Ghosh; Atimukta Jha; INSACOG Consortium; Punit Prasad; Rajeeb Swain; Rupesh Dash; Safal Walia; Shifu Aggarwal; Sunil K. Raghav
EPI_ISL_2614418	Dept. of Laboratory Medicine	Dept. of Laboratory Medicine	Anna Gschaider; Claudia Weber; Fabian Konig; Harald Esterbauer; Oswald Wagner; Petra Jurkowitsch; Robert Strassl; Sabina Plumer
EPI_ISL_774897	Designated Reference Institute for Chemical Measurements (DRICM)	DNA SOLUTION LTD.	Abdul Khaleque; Abu Sufian; Hasan Ul Haider; Jannatun Naima; Kazi Nadim Hasan; MSM Chowdhury; Mala Khan; Mamudul Hasan Razu; Md. Imran Khan; Mizanur Rahman; Mohammad Fazle Alam Rabbi
EPI_ISL_2080488	Diagnofirm Medical Laboratories	Botswana Harvard HIV Reference Laboratory	Boitumelo Zuze; Botshelo Radibe; Dorcas Maruapula; Joseph Makhema; Keoratile Ntshambiwa; Kgomotso Moruisi; Legodile Kooepile; Madisa Mine; Mosepele Mosepele; Pamela Smith-Lawrence; Roger Shapiro; Shahin Lockman; Sikhulile Moyo; Simani Gaseitsiwe; Thongbotho Mphoyakgosi; Wonderful T. Choga
EPI_ISL_1447349, EPI_ISL_1447360	Diagnostic and Research Center of Infectious Diseases, Medical Faculty, Andalas University	Diagnostic and Research Center of Infectious Diseases, Medical Faculty, Andalas University	Andani Eka Putra; Ayu Novita Trisnawati; Dede Rahman Agustian; Desmawati; Dessy Arisanty; Fauzul Azhim; Gestina Aliska; Ikhwan R. Sudji; Juane Plantika Menra; Linosefa; Mutia Lailani; Nia Ayuni Putri; Nita Afriani; SM Rezvi; Sekar Asri Tresnaningtyas; Siskalil Fahma; Syafrizayanti; Syandrez Prima Putra; Yolani Syaputri
EPI_ISL_2115134, EPI_ISL_2115136, EPI_ISL_2115142, EPI_ISL_2115145	Dianovis GmbH Greiz	Robert Koch Institute	
EPI_ISL_2047571	Dinas Kesehatan Kab Blitar	National Institute of Health Research and Development	Arie Ardiansyah Nugraha; Hana Apsari Pawestri; Hartanti Dian Ikawati; Kartika Dewi Puspa; Krisna Pangesti; Nelly Puspandari; Subangkit; Triyani Soekarso; Vivi Setiawaty
EPI_ISL_2233090	Dinkes Samarinda	National Institute of Health Research and Development	Arie Ardiansyah Nugraha; Hana Apsari Pawestri; Hartanti Dian Ikawati; Kartika Dewi Puspa; Krisna Pangesti; Nelly Puspandari; Subangkit; Triyani Soekarso; Vivi Setiawaty
EPI_ISL_2341996, EPI_ISL_2342047, EPI_ISL_2342122, EPI_ISL_2342262, EPI_ISL_2342281, EPI_ISL_2342291	District Head Quarters Hospital, Nayagarh	Institute of Life Sciences - INSACOG	Ajay Parida; Amol M. Kanampalliwar; Arup Ghosh; Atimukta Jha; INSACOG Consortium; Omprakash Shiriwas; Punit Prasad; Rajeeb Swain; Rupesh Dash; Safal Walia; Sana Fatma; Shifu Aggarwal; Sunil K. Raghav
EPI_ISL_2342038, EPI_ISL_2342065, EPI_ISL_2342070, EPI_ISL_2342073, EPI_ISL_2342079, EPI_ISL_2342086, EPI_ISL_2342299, EPI_ISL_2342300, EPI_ISL_2342301, EPI_ISL_2342302, EPI_ISL_2342303, EPI_ISL_2342304, EPI_ISL_2342305, EPI_ISL_2342306			
see above	District Headquarter Hospital, Puri	Institute of Life Sciences - INSACOG	Ajay Parida; Amol M. Kanampalliwar; Arup Ghosh; Atimukta Jha; INSACOG Consortium; Omprakash Shiriwas; Punit Prasad; Rajeeb Swain; Rupesh Dash; Safal Walia; Sana Fatma; Shifu Aggarwal; Sunil K. Raghav
EPI_ISL_2342100, EPI_ISL_2342286, EPI_ISL_2342289	District Headquarters Hospital, Deogarh	Institute of Life Sciences - INSACOG	Ajay Parida; Amol M. Kanampalliwar; Arup Ghosh; Atimukta Jha; INSACOG Consortium; Omprakash Shiriwas; Punit Prasad; Rajeeb Swain; Rupesh Dash; Safal Walia; Sana Fatma; Shifu Aggarwal; Sunil K. Raghav
EPI_ISL_2379462, EPI_ISL_2379464, EPI_ISL_2379465, EPI_ISL_2379467, EPI_ISL_2379468, EPI_ISL_2379469, EPI_ISL_2379470, EPI_ISL_2379471, EPI_ISL_2379472, EPI_ISL_2379473, EPI_ISL_2379476, EPI_ISL_2379477, EPI_ISL_2379478, EPI_ISL_2379479, EPI_ISL_2379480, EPI_ISL_2379481, EPI_ISL_2379486, EPI_ISL_2379488, EPI_ISL_2379491, EPI_ISL_2379493, EPI_ISL_2379494, EPI_ISL_2379496, EPI_ISL_2379497, EPI_ISL_2379498, EPI_ISL_2379499, EPI_ISL_2379500, EPI_ISL_2379501, EPI_ISL_2379502, EPI_ISL_2379504, EPI_ISL_2379505, EPI_ISL_2379506, EPI_ISL_2379507, EPI_ISL_2379508, EPI_ISL_2379509, EPI_ISL_2379513, EPI_ISL_2379514, EPI_ISL_2379515, EPI_ISL_2379518, EPI_ISL_2379519, EPI_ISL_2379520, EPI_ISL_2379522, EPI_ISL_2379523, EPI_ISL_2379526, EPI_ISL_2379527, EPI_ISL_2379528, EPI_ISL_2379529, EPI_ISL_2379530, EPI_ISL_2379532, EPI_ISL_2379533, EPI_ISL_2379536, EPI_ISL_2379537, EPI_ISL_2379538, EPI_ISL_2379539, EPI_ISL_2379540, EPI_ISL_2379541, EPI_ISL_2379543, EPI_ISL_2379544, EPI_ISL_2379545, EPI_ISL_2379550, EPI_ISL_2379558, EPI_ISL_2379559, EPI_ISL_2379560, EPI_ISL_2379561, EPI_ISL_2379562, EPI_ISL_2379564, EPI_ISL_2379565, EPI_ISL_2379566, EPI_ISL_2379568, EPI_ISL_2379569, EPI_ISL_2379570, EPI_ISL_2379571, EPI_ISL_2379572, EPI_ISL_2379573, EPI_ISL_2379575, EPI_ISL_2379576, EPI_ISL_2379577, EPI_ISL_2379578, EPI_ISL_2379580, EPI_ISL_2379581, EPI_ISL_2379582, EPI_ISL_2379583, EPI_ISL_2379584, EPI_ISL_2379585, EPI_ISL_2379586, EPI_ISL_2379587, EPI_ISL_2379594, EPI_ISL_2379595, EPI_ISL_2379596, EPI_ISL_2379598, EPI_ISL_2379599, EPI_ISL_2379600, EPI_ISL_2379601, EPI_ISL_2379602, EPI_ISL_2379603, EPI_ISL_2379604, EPI_ISL_2379606, EPI_ISL_2379607, EPI_ISL_2379608, EPI_ISL_2379610, EPI_ISL_2379611, EPI_ISL_2379612, EPI_ISL_2379613, EPI_ISL_2379615, EPI_ISL_2379616, EPI_ISL_2379617, EPI_ISL_2379618, EPI_ISL_2379619, EPI_ISL_2379620, EPI_ISL_2379621, EPI_ISL_2379622, EPI_ISL_2379623, EPI_ISL_2379624, EPI_ISL_2379625, EPI_ISL_2379627, EPI_ISL_2379628, EPI_ISL_2379629, EPI_ISL_2379632, EPI_ISL_2379633, EPI_ISL_2379634, EPI_ISL_2379638, EPI_ISL_2379639, EPI_ISL_2379640, EPI_ISL_2379641, EPI_ISL_2379642, EPI_ISL_2379645, EPI_ISL_2379646, EPI_ISL_2379647, EPI_ISL_2379648, EPI_ISL_2379649			
see above	Dr S Raju, Director of Public Heath and Preventive Medicine	inStem NCBS - INSACOG	Uma Ramakrishnan Dasaradhi Palakodeti Aswin SaiNarain
EPI_ISL_2521771, EPI_ISL_2521772, EPI_ISL_2521773, EPI_ISL_2521774, EPI_ISL_2521775, EPI_ISL_2521776, EPI_ISL_2521777, EPI_ISL_2521792, EPI_ISL_2521793, EPI_ISL_2521795, EPI_ISL_2521797, EPI_ISL_2521798, EPI_ISL_2521802, EPI_ISL_2521803, EPI_ISL_2521804, EPI_ISL_2521805, EPI_ISL_2521806, EPI_ISL_2521807, EPI_ISL_2521808, EPI_ISL_2521809, EPI_ISL_2521810, EPI_ISL_2521812, EPI_ISL_2521813, EPI_ISL_2521814, EPI_ISL_2521815, EPI_ISL_2521816, EPI_ISL_2521817, EPI_ISL_2521818, EPI_ISL_2521819, EPI_ISL_2521820, EPI_ISL_2521821, EPI_ISL_2521823, EPI_ISL_2521826, EPI_ISL_2521827, EPI_ISL_2521828, EPI_ISL_2521830, EPI_ISL_2521831, EPI_ISL_2521832, EPI_ISL_2521833, EPI_ISL_2521834, EPI_ISL_2521836, EPI_ISL_2521837, EPI_ISL_2521838, EPI_ISL_2521839, EPI_ISL_2521840, EPI_ISL_2521841, EPI_ISL_2521842, EPI_ISL_2521844, EPI_ISL_2521845, EPI_ISL_2521849, EPI_ISL_2521850, EPI_ISL_2521851, EPI_ISL_2521852, EPI_ISL_2521853, EPI_ISL_2521854, EPI_ISL_2521855, EPI_ISL_2521856, EPI_ISL_2521858, EPI_ISL_2521859, EPI_ISL_2521860, EPI_ISL_2521861, EPI_ISL_2521862, EPI_ISL_2521863, EPI_ISL_2521864, EPI_ISL_2521865, EPI_ISL_2521866, EPI_ISL_2521869, EPI_ISL_2521872, EPI_ISL_2521873, EPI_ISL_2521876, EPI_ISL_2521877, EPI_ISL_2521878, EPI_ISL_2521894, EPI_ISL_2521895, EPI_ISL_2521896, EPI_ISL_2521898, EPI_ISL_2521899, EPI_ISL_2521900, EPI_ISL_2521901, EPI_ISL_2521902, EPI_ISL_2521907, EPI_ISL_2521928, EPI_ISL_2521929, EPI_ISL_2521931, EPI_ISL_2521932, EPI_ISL_2521933, EPI_ISL_2521934, EPI_ISL_2521935, EPI_ISL_2521936, EPI_ISL_2521937, EPI_ISL_2521938, EPI_ISL_2521939, EPI_ISL_2521940, EPI_ISL_2521941, EPI_ISL_2521942, EPI_ISL_2521943, EPI_ISL_2521944, EPI_ISL_2521951, EPI_ISL_2521955, EPI_ISL_2521956, EPI_ISL_2521957, EPI_ISL_2521958			
see above	Dr S raju, Director of public heath and preventive medicine	inStem NCBS - INSACOG	Uma Ramakrishnan Dasaradhi Palakodeti Aswin SaiNarain
EPI_ISL_2723777, EPI_ISL_2723778, EPI_ISL_2723779, EPI_ISL_2723780, EPI_ISL_2723782, EPI_ISL_2723787, EPI_ISL_2723788, EPI_ISL_2723790, EPI_ISL_2723791, EPI_ISL_2723792, EPI_ISL_2723800, EPI_ISL_2723801, EPI_ISL_2723804, EPI_ISL_2723805, EPI_ISL_2723806, EPI_ISL_2723807, EPI_ISL_2723809, EPI_ISL_2723810, EPI_ISL_2723812, EPI_ISL_2723813, EPI_ISL_2723830, EPI_ISL_2723831, EPI_ISL_2723834, EPI_ISL_2723835, EPI_ISL_2723836, EPI_ISL_2723837, EPI_ISL_2723838, EPI_ISL_2723843, EPI_ISL_2723845, EPI_ISL_2723847, EPI_ISL_2723848, EPI_ISL_2723849, EPI_ISL_2723850, EPI_ISL_2723851, EPI_ISL_2723852, EPI_ISL_2723853, EPI_ISL_2723857, EPI_ISL_2723858, EPI_ISL_2723862, EPI_ISL_2723863, EPI_ISL_2723864, EPI_ISL_2723865, EPI_ISL_2723866, EPI_ISL_2723870, EPI_ISL_2723871, EPI_ISL_2723872, EPI_ISL_2723874, EPI_ISL_2723875, EPI_ISL_2723876, EPI_ISL_2723878, EPI_ISL_2723879, EPI_ISL_2723880, EPI_ISL_2723881, EPI_ISL_2723882, EPI_ISL_2723883, EPI_ISL_2723884, EPI_ISL_2723885, EPI_ISL_2723886, EPI_ISL_2723887, EPI_ISL_2723888, EPI_ISL_2723889, EPI_ISL_2723890, EPI_ISL_2723891, EPI_ISL_2723892, EPI_ISL_2723893, EPI_ISL_2723894, EPI_ISL_2723895, EPI_ISL_2723896, EPI_ISL_2723897, EPI_ISL_2723898, EPI_ISL_2723900, EPI_ISL_2723901, EPI_ISL_2723902, EPI_ISL_2723903, EPI_ISL_2723904, EPI_ISL_2723905, EPI_ISL_2723906, EPI_ISL_2723911, EPI_ISL_2723912, EPI_ISL_2723916, EPI_ISL_2723917, EPI_ISL_2723918, EPI_ISL_2723919, EPI_ISL_2723920, EPI_ISL_2723922, EPI_ISL_2723923, EPI_ISL_2723924, EPI_ISL_2723925, EPI_ISL_2723926, EPI_ISL_2723927, EPI_ISL_2723928, EPI_ISL_2723929, EPI_ISL_2723930, EPI_ISL_2723931, EPI_ISL_2723932, EPI_ISL_2723933, EPI_ISL_2723934, EPI_ISL_2723938, EPI_ISL_2723940, EPI_ISL_2723941, EPI_ISL_2723942, EPI_ISL_2723943, EPI_ISL_2723944, EPI_ISL_2723945, EPI_ISL_2723946, EPI_ISL_2723950, EPI_ISL_2723951, EPI_ISL_2723961, EPI_ISL_2723962, EPI_ISL_2723963, EPI_ISL_2723964, EPI_ISL_2723966, EPI_ISL_2723967, EPI_ISL_2723968, EPI_ISL_2723969, EPI_ISL_2723970, EPI_ISL_2723971, EPI_ISL_2723972, EPI_ISL_2723973, EPI_ISL_2723974, EPI_ISL_2723975, EPI_ISL_2723976, EPI_ISL_2723977, EPI_ISL_2723978, EPI_ISL_2723979, EPI_ISL_2724011, EPI_ISL_2724012, EPI_ISL_2724013, EPI_ISL_2724014, EPI_ISL_2724015, EPI_ISL_2724016, EPI_ISL_2724017, EPI_ISL_2724018, EPI_ISL_2724019, EPI_ISL_2724020, EPI_ISL_2724021, EPI_ISL_2724023, EPI_ISL_2724034, EPI_ISL_2724041, EPI_ISL_2724042, EPI_ISL_2724043, EPI_ISL_2724044, EPI_ISL_2724045, EPI_ISL_2724046, EPI_ISL_2724047, EPI_ISL_2724048, EPI_ISL_2724049, EPI_ISL_2724050, EPI_ISL_2724051, EPI_ISL_2724052, EPI_ISL_2724054, EPI_ISL_2724057, EPI_ISL_2724063, EPI_ISL_2724080, EPI_ISL_2724081, EPI_ISL_2724082, EPI_ISL_2724083, EPI_ISL_2724084, EPI_ISL_2724085, EPI_ISL_2724087, EPI_ISL_2724088, EPI_ISL_2724089, EPI_ISL_2724090, EPI_ISL_2724094, EPI_ISL_2724095, EPI_ISL_2724096, EPI_ISL_2724097, EPI_ISL_2724098, EPI_ISL_2724099, EPI_ISL_2724100, EPI_ISL_2724101, EPI_ISL_2724102, EPI_ISL_2724103, EPI_ISL_2724104, EPI_ISL_2724105, EPI_ISL_2724106, EPI_ISL_2724107, EPI_ISL_2724108, EPI_ISL_2724109, EPI_ISL_2724110, EPI_ISL_2724112, EPI_ISL_2724113, EPI_ISL_2724114, EPI_ISL_2724115, EPI_ISL_2724116, EPI_ISL_2724117, EPI_ISL_2724118, EPI_ISL_2724119, EPI_ISL_2724120, EPI_ISL_2724121, EPI_ISL_2724122, EPI_ISL_2724123, EPI_ISL_2724124, EPI_ISL_2724125, EPI_ISL_2724127, EPI_ISL_2724128, EPI_ISL_2724129, EPI_ISL_2724130, EPI_ISL_2724132, EPI_ISL_2724133, EPI_ISL_2724134, EPI_ISL_2724135, EPI_ISL_2724136, EPI_ISL_2724137, EPI_ISL_2724144, EPI_ISL_2724145, EPI_ISL_2724146, EPI_ISL_2724147, EPI_ISL_2724148, EPI_ISL_2724149, EPI_ISL_2724150, EPI_ISL_2724151, EPI_ISL_2724152, EPI_ISL_2724153, EPI_ISL_2724154, EPI_ISL_2724155, EPI_ISL_2724157, EPI_ISL_2724158, EPI_ISL_2724159, EPI_ISL_2724160, EPI_ISL_2724161, EPI_ISL_2724162, EPI_ISL_2724163, EPI_ISL_2724164, EPI_ISL_2724165, EPI_ISL_2724166, EPI_ISL_2724167, EPI_ISL_2724195, EPI_ISL_2724197, EPI_ISL_2724198, EPI_ISL_2724203, EPI_ISL_2724205, EPI_ISL_2724206, EPI_ISL_2724207, EPI_ISL_2724210, EPI_ISL_2724211, EPI_ISL_2724212, EPI_ISL_2724213, EPI_ISL_2724214, EPI_ISL_2724215, EPI_ISL_2724216, EPI_ISL_2724240, EPI_ISL_2724243, EPI_ISL_2724244, EPI_ISL_2724247, EPI_ISL_2724249, EPI_ISL_2724251, EPI_ISL_2724252, EPI_ISL_2724253, EPI_ISL_2724254, EPI_ISL_2724255, EPI_ISL_2724256, EPI_ISL_2724257, EPI_ISL_2724258, EPI_ISL_2724259, EPI_ISL_2724260, EPI_ISL_2724267, EPI_ISL_2724268, EPI_ISL_2724269, EPI_ISL_2724270, EPI_ISL_2724271, EPI_ISL_2724273, EPI_ISL_2724277, EPI_ISL_2724278, EPI_ISL_2724280, EPI_ISL_2724281, EPI_ISL_2724283, EPI_ISL_2724286, EPI_ISL_2724327, EPI_ISL_2724328, EPI_ISL_2724329, EPI_ISL_2724330, EPI_ISL_2724331, EPI_ISL_2724348			
see above	Dr S raju, Director of public heath and preventive medicine	inStem NCBS – INSACOG	Uma Ramakrishnan Dasaradhi Palakodeti Aswin SaiNarain
EPI_ISL_547447, EPI_ISL_547452, EPI_ISL_636519, EPI_ISL_636521, EPI_ISL_1013810, EPI_ISL_1014269, EPI_ISL_1014270, EPI_ISL_1014556, EPI_ISL_1014557, EPI_ISL_1014558, EPI_ISL_1014562, EPI_ISL_1014564, EPI_ISL_1014571, EPI_ISL_1014579, EPI_ISL_1014639, EPI_ISL_1035771, EPI_ISL_1035798, EPI_ISL_1165588, EPI_ISL_1165605, EPI_ISL_1232247, EPI_ISL_1232259, EPI_ISL_1232272, EPI_ISL_1232283, EPI_ISL_1232291, EPI_ISL_1232318, EPI_ISL_1232326, EPI_ISL_1232327, EPI_ISL_1232561, EPI_ISL_1232619, EPI_ISL_1232869, EPI_ISL_1232876, EPI_ISL_1232887, EPI_ISL_1288132, EPI_ISL_1288133, EPI_ISL_1288136, EPI_ISL_1288141, EPI_ISL_1288147, EPI_ISL_1289188, EPI_ISL_1289384, EPI_ISL_1289397, EPI_ISL_1369750, EPI_ISL_1369763, EPI_ISL_1369779, EPI_ISL_1370228, EPI_ISL_1370238, EPI_ISL_1370589, EPI_ISL_1370626, EPI_ISL_1370642, EPI_ISL_1371268, EPI_ISL_1456453, EPI_ISL_1456476, EPI_ISL_1457455, EPI_ISL_1457464, EPI_ISL_1521070, EPI_ISL_1521142, EPI_ISL_1521153, EPI_ISL_1521860, EPI_ISL_1521862, EPI_ISL_1521874, EPI_ISL_1596149, EPI_ISL_1596269, EPI_ISL_1596449, EPI_ISL_1704915, EPI_ISL_1704992, EPI_ISL_1705242, EPI_ISL_1705251, EPI_ISL_1705285, EPI_ISL_1705293, EPI_ISL_1792321, EPI_ISL_1792363, EPI_ISL_1792380, EPI_ISL_1792922, EPI_ISL_1961903, EPI_ISL_1961905, EPI_ISL_1962254, EPI_ISL_1962940, EPI_ISL_1962944, EPI_ISL_1962977, EPI_ISL_2093071, EPI_ISL_2093073, EPI_ISL_2093608, EPI_ISL_2093888, EPI_ISL_2218108, EPI_ISL_2219019, EPI_ISL_2219329, EPI_ISL_2220456, EPI_ISL_2302965, EPI_ISL_2302974, EPI_ISL_2303447, EPI_ISL_2405550, EPI_ISL_2405561, EPI_ISL_2405573, EPI_ISL_2405584, EPI_ISL_2405596, EPI_ISL_2405597, EPI_ISL_2405608, EPI_ISL_2405609, EPI_ISL_2405628, EPI_ISL_2406352, EPI_ISL_2476279, EPI_ISL_2610126, EPI_ISL_2610170, EPI_ISL_2610186, EPI_ISL_2610657, EPI_ISL_2610754, EPI_ISL_2610765			
see above	Dutch COVID-19 response team	National Institute for Public Health and the Environment (RIVM)	Adam Meijer; AnneMarie van den Brandt; Annelies Kroneman; Bas van der Veer; Chantal Reusken; Dennis Schmitz; Dirk Eggink; Euníce Then; Florian Zwagemaker; Harry Vennema; James Groot; Jeroen Cremer; Jolienke Hardeman; Karim Hajji; Kim Freriks; Linda van de Nes; Lisa Wijsman; Lynn Aarts; Melissa van Tuil; Robert Kohl; Ryanne Jaarsma; Sanne Bos; Sharon van den Brink; Sjoerd Kuiling; on behalf of the national COVID-19 response team
EPI_ISL_421656	E. Gulbja Laboratorija	Latvian Biomedical Research and Study Centre	Dmitrijs Perminovs; Ivars Silamiķelis; Jānis Kloviņš; Kaspars Megnis; Mikus Gavars; Monta Ustinova; Uga Dumpis; Vita Rovīte; Ņikita Zrelovs
EPI_ISL_961860, EPI_ISL_1312683, EPI_ISL_2141840	E. Gulbja laboratorija	Latvian Biomedical Research and Study Centre	Davids Fridmanis; Davids Fridmanis; Dmitrijs Perminovs; Dmitrijs Perminovs; Elina Dimina; Guntars Zarins; Ivars Silamikelis; Ivars Silamikelis; Janis Klovins; Janis Pjalkovskis; Janis Klovins; Jurijs Perevoscikovs; Kaspars Megnis; Kaspars Megnis; Laila Silamikele; Laila Silamikele; Lauma Freimane; Lauma Freimane; Laura Ansone; Laura Ansone; Liga Birzniece; Liga Birzniece; Mikus Gavars; Mikus Gavars; Monta Ustinova; Monta Ustinova; Nikita Zrelovs; Nikita Zrelovs; Uga Dumpis; Uga Dumpis; Una Krumina; Vita Rovite; Vita Rovite
EPI_ISL_1582978, EPI_ISL_1582980	E.S.E. HOSPITAL SAN JOSE DE MAICAO	Instituto Nacional de Salud- Dirección de Investigación en Salud Pública	Carlos Franco-Muñoz; Carmen Osorio; Diana Malo; Diego A. Álvarez-Díaz; Diego Andrés Prada; Gerardo Santamaría; Hector Alejandro Ruiz-Moreno; Jhonnatan Reales-González; Juan Camilo Martinez; Julian Naizaque; Katherine Laiton-Donato; Lisseth Pardo; Magdalena Wiesner; Marcela Mercado-Reyes; Maria T. Herrera-Sepúlveda; Marta Lopez Blanco; Martha Lucia Ospina Martinez; Paola Rojas; Sergio Gomez; Sheryll Corchuelo; Ángela Alarcon Cruz
EPI_ISL_2477869, EPI_ISL_2478413, EPI_ISL_2478415, EPI_ISL_2478457, EPI_ISL_2478515, EPI_ISL_2478641, EPI_ISL_2478642, EPI_ISL_2478963, EPI_ISL_2478988, EPI_ISL_2479000, EPI_ISL_2479023, EPI_ISL_2479399, EPI_ISL_2479454, EPI_ISL_2479472, EPI_ISL_2479479, EPI_ISL_2479575, EPI_ISL_2479576, EPI_ISL_2479581, EPI_ISL_2479594, EPI_ISL_2479801, EPI_ISL_2479844, EPI_ISL_2479857, EPI_ISL_2479874			
see above	Edmonton Provincial Lab	Public Health Agency of Canada (PHAC) National Microbiology Laboratory	Buss; Croxen M; Deo A; Dieu P; E; Ferrato C; Gill K; Khan F; Koleva P; Li V; Lloyd C; Lynch T; Ma R; Murphy S; Pabbaraju K; Shokoples S; Thayer J; Tipples G; Whitehouse M; Wong A; Yu C; Zelyas N
EPI_ISL_2029260	Ekstralab Tuzla	Alea Genetic Centre	Adis Kandic; Dino Pecar; Enis Kandic; Lana Salihefendic; Nusret Butkovic; Rijad Konjhodzic
EPI_ISL_1398925	Emam Ali Hospital	Razi Vaccine and Serum Research Institute	Akbar Khorasani; Amir Kaffashi; Jiabin Huang; Mohammad Hossein Fallah Mehrabadi; Mohsen Bashashati; Mohsen Lotfi; Morteza Taghizadeh; S Reza Banihashemi; Sayed Hamidreza Mozhgan
EPI_ISL_2241623	Ethiopian Biotechnology Institute (EBTI)	International Centre for Genetic Engineering and Biotechnology (ICGEB) and ARGO Open Lab for Genome Sequencing	Alessandro Marcello; Danilo Licastro; Emanuele Orsini; Getnet Hailu; Hailu Dadi; Kassahun Tesfaye; Keyru Tuki; Kominist Asmamaw; Molalegne Bitew; Simeone Dal Monego; Yakob Gebregziabher Tsegay
EPI_ISL_1845854, EPI_ISL_1846077, EPI_ISL_1846153, EPI_ISL_2110448, EPI_ISL_2110453, EPI_ISL_2110454, EPI_ISL_2110460, EPI_ISL_2110488, EPI_ISL_2110489, EPI_ISL_2110499, EPI_ISL_2110715, EPI_ISL_2110737, EPI_ISL_2123724, EPI_ISL_2123736, EPI_ISL_2123791, EPI_ISL_2123794, EPI_ISL_2123795, EPI_ISL_2123806, EPI_ISL_2259546, EPI_ISL_2259576, EPI_ISL_2259579, EPI_ISL_2259582, EPI_ISL_2678480			
see above	Eurofins LifeCodexx GmbH	Robert Koch Institute	
EPI_ISL_2341690, EPI_ISL_2341694, EPI_ISL_2341850, EPI_ISL_2341931, EPI_ISL_2341932, EPI_ISL_2341934, EPI_ISL_2341935, EPI_ISL_2341936, EPI_ISL_2342011, EPI_ISL_2342024, EPI_ISL_2342026, EPI_ISL_2342035, EPI_ISL_2342052, EPI_ISL_2342056, EPI_ISL_2342080			
see above	FM Medical College, Balasore	Institute of Life Sciences - INSACOG	Ajay Parida; Amol M. Kanampalliwar; Arup Ghosh; Atimukta Jha; INSACOG Consortium; Omprakash Shiriwas; Punit Prasad; Rajeeb Swain; Rupesh Dash; Safal Walia; Sana Fatma; Shifu Aggarwal; Sunil K. Raghav
EPI_ISL_1632497	FUNDACION VALLE DE LILI	Instituto Nacional de Salud- Dirección de Investigación en Salud Pública	Carlos Franco-Muñoz; Carmen Osorio; Diana Malo; Diego A. Álvarez-Díaz; Diego Andrés Prada; Gerardo Santamaría; Hector Alejandro Ruiz-Moreno; Jhonnatan Reales-González; Jorge Rivera; Juan Camilo Martinez; Julian Naizaque; Katherine Laiton-Donato; Lisseth Pardo; Magdalena Wiesner; Marcela Mercado-Reyes; Maria T. Herrera-Sepúlveda; Marta Lopez Blanco; Martha Lucia Ospina Martinez; Paola Rojas; Sergio Gomez; Sheryll Corchuelo; Ángela Alarcon Cruz
EPI_ISL_2466841, EPI_ISL_2467518	Fimlab Laboratories	Fimlab Laboratories	Bruno Luukinen; Leena Huhti; Mauri Keinänen; Minna Paloniemi; Sara Lehtinen; Tapio Seiskari
EPI_ISL_2363868, EPI_ISL_2508263, EPI_ISL_2644898	Fimlab Laboratoriot Oy Tampere	Expert Microbiology, National Institute for Health and Welfare	Carita Savolainen-Kopra; Erika Lindh; Haider al-Hello; Jani Halkilahti; Kirsi Liitsola; Niina Ikonen; Olli Vapalahti; Pekka Ellonen; Phuoc Truong; Päivi Laurila; Ravi Kant; Sari Hannula; Soile Blomqvist; Teemu Smura
EPI_ISL_581487	Fondation Congolaise pour la recherche medicale (FCRM)	NGS Competence Center Tübingen, Institut für Medizinische Mikrobiologie und Hygiene, Universitätsklinikum Tübingen	Angel Angelov
EPI_ISL_912356	Fondation Congolaise pour la recherche medicale (FCRM), Francine Ntoumi	NGS Competence Center Tuebingen, Institut für Medizinische Mikrobiologie und Hygiene, Universitaetsklinikum Tübingen	Angel Angelov
EPI_ISL_2620951	Fondazione IRCCS Ca' Granda Ospedale Maggiore Policlinico	Fondazione IRCCS Ca' Granda Ospedale Maggiore Policlinico	Ferruccio Ceriotti; Sara Uceda Renteria
EPI_ISL_2125018, EPI_ISL_2125024	Fraunhofer-Institut fur Zelltherapie und Immunologie IZI AG Next-Generation Diagnostics	Robert Koch Institute	
EPI_ISL_479976	Fukui Prefectural Institute of Public Health and Environmental Science	Pathogen Genomics Center, National Institute of Infectious Diseases	Hajime Kamiya; Kentaro Itokawa; Makoto Kuroda; Masanori Hashino; Miho Toho; Motoi Suzuki; Rina Tanaka; Tsuyoshi Sekizuka

EPI_ISL_684666	Fukuoka Institute of Health and Environmental Sciences	Pathogen Genomics Center, National Institute of Infectious Diseases	Kentaro Itokawa; Makoto Kuroda; Masanori Hashino; Rina Tanaka; Tsuyoshi Sekizuka
EPI_ISL_1182578	Fundação Ezequiel Dias (FUNED)	Coordenação Geral de Laboratórios de Saúde Pública (CGLAB/DAEVS/SVS/MS)	Vagner Fonseca; et al.
EPI_ISL_2228933	GH A.CHENEVIER-H.MONDOR	Department of Virology, Henri Mondor University Hospital, Assistance Publique Hôpitaux de Paris, Université Paris-Est Créteil, INSERM U955	Alexandre Soulier; Christophe Rodriguez; Elisabeth Trawinski; Guillaume Gricourt; Jean-Michel Pawlotsky; Melissa N'Debi; Slim Fourati; Vanessa Demontant
EPI_ISL_1755093	GH JOFFRE DUPUYTREN	Department of Virology, Henri Mondor University Hospital, Assistance Publique Hôpitaux de Paris, Université Paris-Est Créteil, INSERM U955	Alexandre Soulier; Christophe Rodriguez; Elisabeth Trawinski; Guillaume Gricourt; Jean-Michel Pawlotsky; Melissa N'Debi; Slim Fourati; Vanessa Demontant
EPI_ISL_2544813, EPI_ISL_2544814, EPI_ISL_2544815	GMERS Government Medical College (GMC), Gandhinagar	Gujarat Biotechnology Research Centre	Chaitanya Joshi; Dinesh Kumar; Gaurishankar Shrimali; Janvi Raval; Madhvi Joshi; Nitesh Shah; Nitin Savaliya; Ramesh Pandit; Sonal Sharma; Twinkle Soni; Umang Mishra; Zarna Patel; Zuber Saiyed
EPI_ISL_2405125, EPI_ISL_2405126, EPI_ISL_2509959, EPI_ISL_2544836	GMERS Government Medical College (GMC), Gotri	Gujarat Biotechnology Research Centre	Bithika Duttaroy; Chaitanya Joshi; Dinesh Kumar; Janvi Raval; Madhvi Joshi; Nitesh Shah; Nitin Savaliya; Ramesh Pandit; Sonal Sharma; Twinkle Soni; Umang Mishra; Zarna Patel; Zuber Saiyed
EPI_ISL_2001183, EPI_ISL_2001185, EPI_ISL_2001186, EPI_ISL_2017757, EPI_ISL_2017758, EPI_ISL_2391489, EPI_ISL_2391490, EPI_ISL_2391491, EPI_ISL_2391492, EPI_ISL_2391493, EPI_ISL_2391494, EPI_ISL_2391495, EPI_ISL_2391496, EPI_ISL_2391497, EPI_ISL_2391498, EPI_ISL_2391501, EPI_ISL_2391502, EPI_ISL_2391503			
see above	GMERS, Government Medical College (GMC), Gotri	Gujarat Biotechnology Research Centre	Bithika Duttaroy; Chaitanya Joshi; Dinesh Kumar; Janvi Raval; Madhvi Joshi; Nitesh Shah; Nitin Savaliya; Ramesh Pandit; Sonal Sharma; Twinkle Soni; Umang Mishra; Zarna Patel; Zuber Saiyed
EPI_ISL_2001139, EPI_ISL_2001140, EPI_ISL_2001143	GMERS, Government Medical College, Gandhinagar	Gujarat Biotechnology Research Centre	Chaitanya Joshi; Dinesh Kumar; Gaurishankar Shrimali; Janvi Raval; Madhvi Joshi; Nitesh Shah; Nitin Savaliya; Ramesh Pandit; Sonal Sharma; Twinkle Soni; Umang Mishra; Zarna Patel; Zuber Saiyed
EPI_ISL_2379353	GOVIDRAJ NAGAR	INSACOG-KA, NIMHANS	Ananthapadmanabha Kotambail; Anita S Desai; Anson Kunjumon George; Chetan G K; Chitra Pattabiraman; Darshan Sreenivas; Gautham Arunachal Udupi; Pramada Prasad; V Ravi
EPI_ISL_1940019	GOVIMDRAJ NAGAR	inStem NCBS - INSACOG	Uma Ramakrishnan Dasaradhi Palakodeti Aswin SaiNarain
EPI_ISL_1940072, EPI_ISL_1940074, EPI_ISL_1940075	GOVINDRAJ NAGAR	inStem NCBS - INSACOG	Uma Ramakrishnan Dasaradhi Palakodeti Aswin SaiNarain
EPI_ISL_1940049, EPI_ISL_1940111	GOVINDRAJNAGAR	inStem NCBS - INSACOG	Uma Ramakrishnan Dasaradhi Palakodeti Aswin SaiNarain
EPI_ISL_2029122	General Hospital "Abdulah Nakas"	Alea Genetic Centre	Adis Kandix; Dino Pecar; Enis Kandic; Lana Salihefendic; Rijad Konjhodzic
EPI_ISL_746478, EPI_ISL_746512, EPI_ISL_746571, EPI_ISL_746650, EPI_ISL_746767, EPI_ISL_746808, EPI_ISL_1167704, EPI_ISL_1167741, EPI_ISL_1167796, EPI_ISL_1167812, EPI_ISL_1167852, EPI_ISL_1167886, EPI_ISL_1300514, EPI_ISL_1300516, EPI_ISL_1321450, EPI_ISL_1321504, EPI_ISL_1321515, EPI_ISL_1321520, EPI_ISL_1321523, EPI_ISL_1321528, EPI_ISL_1321589, EPI_ISL_1470422, EPI_ISL_1470435, EPI_ISL_1470440, EPI_ISL_1470452, EPI_ISL_1470550, EPI_ISL_1470554, EPI_ISL_1534583, EPI_ISL_1534591, EPI_ISL_1541005, EPI_ISL_1633496, EPI_ISL_1712335, EPI_ISL_2009200, EPI_ISL_2009256, EPI_ISL_2009258, EPI_ISL_2009577, EPI_ISL_2009655, EPI_ISL_2391055, EPI_ISL_2391219, EPI_ISL_2391318, EPI_ISL_2391473, EPI_ISL_2508582, EPI_ISL_2508589, EPI_ISL_2543503, EPI_ISL_2543535, EPI_ISL_2543590, EPI_ISL_2557257			
see above	Genetica Molecular and Subdepartamento de Virologia ISP Chile	Instituto de Salud Publica de Chile	Andres Castillo; Barbara Parra; Constanza Campano; Gisselle Barra; Jaime Lagos; Javier Tognarelli; Jorge Fernandez; Karen Orostica; Loredana Arata; Patricia Bustos; Rodrigo Fasce; Soledad Ulloa
EPI_ISL_2565984	Genome Analysis Center, Yamanashi Central Hospital	Genome Analysis Center, Yamanashi Central Hospital	Yosuke Hirotsu
EPI_ISL_2348662	Genome Center	Genome Center	A. S. M. Rubayet Ul Alam; Ali Ahsan Setu; Hassan M. Al-Emran; Iqbal Kabir Jahid; M. Anwar Hossain; M. Shaminur Rahman; M. Tanvir Islam; Md. Shazid Hasan; Ovinu Kibria Islam; Prosanto Kumar Das; Shovon Lal Sarkar; Tanay Chakrabarty
EPI_ISL_1942249	Genome Centre	Genome Centre	A. S. M. Rubayet Ul Alam; Ali Ahsan Setu; Hassan M. Al-Emran; Iqbal Kabir Jahid; M. Anwar Hossain; M. Shaminur Rahman; M. Tanvir Islam; Md. Shazid Hasan; Ovinu Kibria Islam; Shovon Lal Sarkar; Tanay Chakrabarty
EPI_ISL_1673680	Genomic Research Lab, BCSIR	Genomic Research Lab, BCSIR	Abu Sayeed Mohammad Mahmud; Barna Goswami; Eshrar Osman; Iffat Jahan; Md. Ahasan Habib; Md. Murshed Hasan Sarkar; Md. Saddam Hossain; Md. Salim Khan; Mohammad Mohi Uddin; Mohammad Samir Uzzaman; Shahina Akter; Tanjina Akhter Banu
EPI_ISL_2566467, EPI_ISL_2566472, EPI_ISL_2566477, EPI_ISL_2566500, EPI_ISL_2566516	Genomics Program, Children Cancer Hospital	Genomics Program, Children Cancer Hospital	Abdelaziz, H.; Abdo, I.; Abouelnaga, S.; Almeldin, A.; Amer, K.; Diab, A.; El-Shaqnqery, H.; El-Zayat, M.; ElHaddad, A.; ElHalafawy, A.; Elnaqeeb, M.; Farawela, H.; Hammad, M.; Hassan, R.; Hassan, W.; Hatem, A.; Hossam, M.; Hussein, S.; Ismail, J.; Jalal, D.; Mansour, T.; Saaid, M.; Said, D.; Salah, H.; Samir, O.; Sayed, A.; Shalaby, L.; Soliman, S.; Yahia, A.
EPI_ISL_2626595, EPI_ISL_2626598	Genomik Solidaritas Indonesia Laboratorium	Genomik Solidaritas Indonesia Laboratorium	Ahmad Zoebad Foeady; Alfin Mohammad Abdillah; Annisa Muthiah Sukirman; Anuraj Shankar; Ariel Pradipta; Carissa Sintca Wijaya; Dhahlia Agustina Cahyono; Gracia Felias Enos Korompis; Meutia Ayuputeri Kumaheri; Normastuti Adhini Tantyo; Vania Gavrila Wikasa
EPI_ISL_2349172, EPI_ISL_2376808	Greek Genome Center, Biomedical Research Foundation of the Academy of Athens (BRFAA)	Greek Genome Center, Biomedical Research Foundation of the Academy of Athens (BRFAA)	Dimitrios Thanos; Emmanouil Athanasiadis; Giannis Vatsellas; Katerina Zoi; Theodoros Loupis
EPI_ISL_2301958, EPI_ISL_2343846, EPI_ISL_2364992, EPI_ISL_2368552, EPI_ISL_2368818, EPI_ISL_2370147, EPI_ISL_2370893, EPI_ISL_2371187, EPI_ISL_2371400, EPI_ISL_2371406			
see above	Greek Genome Center, Biomedical Research Foundation of the Academy of Athens (BRFAA)	Greek Genome Center, Biomedical Research Foundation of the Academy of Athens (BRFAA)	Dimitrios Thanos; Emmanouil Athanasiadis; Giannis Vatsellas; Katerina Zoi; Theodoros Loupis
EPI_ISL_2339831, EPI_ISL_2603169	Grupo de Investigación en Enfermedades Tropicales del Ejército (GINETEJ), Laboratorio de Referencia e Investigación, Dirección de Sanidad Ejército, Bogotá, Colombia	Centro de Investigaciones en Microbiología y Biotecnología-UR (CIMBIUR), Facultad de Ciencias Naturales, Universidad del Rosario, Bogotá, Colombia	Camilo A. Correa-Cárdenas; Carolina Oliveros; Claudia Méndez; Elizabeth K. Márquez; Frank de los Santos Ortiz; Juan David Ramírez; Julie Pérez; Lorena Albarracín; Luz H. Patiño; Maria Clara Duque; Marina Muñoz; María Teresa Alvarado; Nathalia Ballesteros; Sergio Castañeda; Sergio Gutíerrez-Riveros; Yanira Romero; Zulma Cucunubá
EPI_ISL_1273073	Guam Public Health Laboratory	Centers for Disease Control and Prevention Division of Viral Diseases, Pathogen Discovery	Anna Montmayeur; Anna Uehara; Ben L. Rambo-Martin; Clinton R. Paden; Dhwani Batra; Haibin Wang; Jasmine Padilla; Jing Zhang; Justin Lee; Katie Dillon; Krista Queen; Kristen Knipe; Kristine Lacek; Lori Rowe; Mark Burroughs; Matthew Schmerer; Mili Sheth; Peter W. Cook; Rachel Marine; Sam Shepard; Sarah Nobles; Shoshona Le; Suxiang Tong; Yan Li; Ying Tao
EPI_ISL_2017789	Gujarat Biotechnology Research Centre	Gujarat Biotechnology Research Centre	Chaitanya Joshi; Dinesh Kumar; Janvi Raval; Madhvi Joshi; Nitesh Shah; Nitin Savaliya; Ramesh Pandit; Sonal Sharma; Twinkle Soni; Umang Mishra; Zarna Patel; Zuber Saiyed
EPI_ISL_2029113, EPI_ISL_2029114	HEGP - Laboratoire de Virologie	HEGP - Laboratoire de Virologie	David Veyer; Hélène Péré; Julien Puech; Maxime Wack; Nicolas Robillard; Sandrine Imbeaud
EPI_ISL_733077, EPI_ISL_733078, EPI_ISL_733452	HELIX LLC	WHO National Influenza Centre Russian Federation	Andrey Komissarov; Anna Ivanova; Artem Fadeev; Daria Danilenko; Dmitry Bazhenov; Dmitry Lioznov; Elena Nabieva; Georgii Bazykin; Ksenia Safina; Kseniya Komissarova
EPI_ISL_2017306, EPI_ISL_2017375, EPI_ISL_2617596, EPI_ISL_2617599, EPI_ISL_2617601, EPI_ISL_2617602, EPI_ISL_2617617, EPI_ISL_2617619, EPI_ISL_2617621			
see above	HLAGYN - Laboratorio de Imunologia de Transplantes de Goias	HLAGYN - Laboratorio de Imunologia de Transplantes de Goias	Alessandro Leonardo Alvares Magalhaes; Daniel Ferreira de Sousa; Danielle de Paiva Rezende; Erika Lopes Rocha Batista; Fernando Antonio Vinhal dos Santos; Frederico Rodrigues Vinhal; Lucas Carlos Gomes Pereira; Paola Cristina Resende Silva; Raphael Bessa Parmigiane; Sabrina Sara Moreira Duarte
EPI_ISL_2731451, EPI_ISL_2731452	HLAGYN-Laboratório de Imunologia de Transplantes de Goiás	Laboratory of Respiratory Viruses and Measles, Oswaldo Cruz Institute, FIOCRUZ	Alice Sampaio Rocha; Ana Carolina Mendonca; Anna Carolina Paixao; Elisa Cavalcante Pereira; Fernando Motta; Luciana Appolinario; Marilda Siqueira on behalf of the Fiocruz COVID-19 Genomic Surveillance Network; Paola Resende; Renata Serrano Lopes; Taina Venas
EPI_ISL_2611671	HOME QUARANTINE TASKFORCE	Hong Kong Department of Health	Alan K.L. Tsang; Dominic N.C. Tsang; Edman T.K. Lam; Ken H.L. Ng; Peter C.W. Yip; Rickjason C.W. Chan
EPI_ISL_1904989, EPI_ISL_1904992, EPI_ISL_1904994, EPI_ISL_1904998, EPI_ISL_2142418	HOPITAL SAINT ANDRE	CNR Virus des Infections Respiratoires - France SUD	Antonin Bal; Bruno Lina; Gregory Destras; Gwendolyne Burfin; Hadrien Regue; Laurence Josset; Martine Valette; Quentin Semanas
EPI_ISL_2379349, EPI_ISL_2379350	HOSA HALLI	INSACOG-KA, NIMHANS	Ananthapadmanabha Kotambail; Anita S Desai; Anson Kunjumon George; Chetan G K; Chitra Pattabiraman; Darshan Sreenivas; Gautham Arunachal Udupi; Pramada Prasad; V Ravi
EPI_ISL_1940001, EPI_ISL_1940002, EPI_ISL_1940003, EPI_ISL_1940004, EPI_ISL_1940008, EPI_ISL_1940011, EPI_ISL_1940013, EPI_ISL_1940014, EPI_ISL_1940015, EPI_ISL_1940062, EPI_ISL_1940064, EPI_ISL_1940067, EPI_ISL_1940068, EPI_ISL_1940112, EPI_ISL_1940113, EPI_ISL_1940114, EPI_ISL_1940118, EPI_ISL_1940119, EPI_ISL_1940123, EPI_ISL_1940125, EPI_ISL_1940127, EPI_ISL_1940128			
see above	HOSAHALLI	inStem NCBS - INSACOG	Uma Ramakrishnan Dasaradhi Palakodeti Aswin SaiNarain
EPI_ISL_1379434, EPI_ISL_1712400	HOSPITAL CIUDAD NEILY	Inciensa, Instituto Costarricense de Investigación y Enseñanza en Nutrición y Salud	Adriana Godínez; Claudio Soto-Garita; Estela Cordero; Francisco Duarte; Hebleen Porras; Joselyn Prado & Raúl Zeledón-Mayorga; José Luis Vargas; Mariela Gutiérrez; Melany Calderón; Melany Calderón & Mariel López
EPI_ISL_914816	HOSPITAL DE NIÑOS DR. CARLOS SAENZ HERRERA	Inciensa, Instituto Costarricense de Investigación y Enseñanza en Nutrición y Salud	Adriana Godínez; Claudio Soto-Garita; Estela Cordero; Francisco Duarte; Hebleen Porras; Melany Calderón & Cristian Pérez-Corrales
EPI_ISL_1494944	HOSPITAL DEPARTAMENTAL SAN VICENTE DE PAUL	Instituto Nacional de Salud- Dirección de Investigación en Salud Pública	Carlos Franco-Muñoz; Carmen Osorio; Diana Malo; Diego A. Álvarez-Díaz; Diego Andrés Prada; Gerardo Santamaría; Hector Alejandro Ruiz-Moreno; Jhonnatan Reales-González; Juan Camilo Martinez; Julian Naizaque; Katherine Laiton-Donato; Lisseth Pardo; Magdalena Wiesner; Marcela Mercado-Reyes; Maria T. Herrera-Sepúlveda; Marta Lopez Blanco; Martha Lucia Ospina Martinez; Paola Rojas; Sergio Gomez; Sheryll Corchuelo; Ángela Alarcon Cruz
EPI_ISL_2493332	HOSPITAL DO RIM E HIPERTENSAO	Instituto Butantan	Antonio Jorge Martins; Claudia Renata dos Santos Barros; David Schlesinger; Debora Botequio Moretti; Dimas Tadeu Covas; Elaine Cristina Marqueze; Elaine Vieira Santos; Evandra Strazza Rodrigues; Heidge Fukumasu; Jayme Augusto de Souza-Neto; José Salvatore Leister Patané; Luiz Alcantara; Luiz Lehmann Coutinho; Maria Carolina Elias; Maurício Lacerda Nogueira; Rafael dos Santos Bezerra; Raul Machado Neto; Rejane Maria Tommasini Grotto; Ricardo Haddad; Sandra Coccuzzo Sampaio Vessoni; Simone Kashima; Svetoslav Nanev Slavov; Vincent Louis Viala
EPI_ISL_1712403, EPI_ISL_1712405, EPI_ISL_2272982	HOSPITAL DR. ENRIQUE BALTODANO BRICEÑO	Inciensa, Instituto Costarricense de Investigación y Enseñanza en Nutrición y Salud	Adriana Godínez; Claudio Soto-Garita; Estela Cordero; Francisco Duarte; Hebleen Porras; Joselyn Prado & Adriana Bemúdez Espinoza; Joselyn Prado & Adriana Bermúdez-Espinoza; José Luis Vargas; Mariela Gutiérrez; Melany Calderón
EPI_ISL_2502738	HOSPITAL DR. MAX TERAN VALLS	Inciensa, Instituto Costarricense de InvestigaciOn y Enseñanza en NutriciOn y Salud	Adriana Godínez; Claudio Soto-Garita; Estela Cordero; Francisco Duarte; Hebleen Porras; Jose Luis Vargas; Joselyn Prado & María Jose GOmez-Umaña; Mariela Gutierrez; Melany CalderOn
EPI_ISL_2103391	HOSPITAL DR. MAX TERAN VALLS	Inciensa, Instituto Costarricense de Investigación y Enseñanza en	Adriana Godínez; Caterina Guzmán; Claudio Soto-Garita; Estela Cordero; Francisco Duarte; Hebleen Porras; Joselyn Prado; José Luis Vargas; Mariela Gutiérrez; Melany Calderón; Nazareth Ruiz & María José Gómez-Umaña

		Nutrición y Salud	
EPI_ISL_2272990	HOSPITAL DR. RAFAEL A. CALDERON GUARDIA	Inciensa, Instituto Costarricense de Investigación y Enseñanza en Nutrición y Salud	Adriana Godínez; Claudio Soto-Garita; Estela Cordero; Francisco Duarte; Hebleen Porras; Joselyn Prado & Fabian Salas-Flores; José Luis Vargas; Mariela Gutiérrez; Melany Calderón
EPI_ISL_1827529	HOSPITAL DR. WILLIAM ALLEN	Inciensa, Instituto Costarricense de Investigación y Enseñanza en Nutrición y Salud	Adriana Godínez; Claudio Soto-Garita; Estela Cordero; Francisco Duarte; Hebleen Porras; Joselyn Prado & Mónica Charpentier-Artavia; José Luis Vargas; Mariela Gutiérrez; Melany Calderón
EPI_ISL_445345	HOSPITAL DR.HERNAN HENRIQUEZ ARAVENA	Instituto de Salud Publica de Chile	Alejandra Acevedo; Andrés E Castillo; Bárbara Parra; Carolina Tambley; Gabriel Leal; Jaime Lagos; Jorge Fernandez; Loredana Arata; Patricia Bustos; Paz Tapia; Rodrigo Fasce; Winston Andrade
EPI_ISL_2103411	HOSPITAL GOLFITO MANUEL MORA VALVERDE	Inciensa, Instituto Costarricense de Investigación y Enseñanza en Nutrición y Salud	Adriana Godínez; Caterina Guzmán; Claudio Soto-Garita; Estela Cordero; Francisco Duarte; Hebleen Porras; Joselyn Prado; José Luis Vargas; Mariela Gutiérrez; Melany Calderón; Nazareth Ruiz & Lizzeth Blanco
EPI_ISL_2502729	HOSPITAL LA ANEXION	Inciensa, Instituto Costarricense de InvestigaciOn y Enseñanza en NutriciOn y Salud	Adriana Godínez; Claudio Soto-Garita; Estela Cordero; Francisco Duarte; Hebleen Porras; Jose Luis Vargas; Joselyn Prado & Ivanna Krize-Morún; Mariela Gutierrez; Melany CalderOn
EPI_ISL_1712382	HOSPITAL MEXICO	Inciensa, Instituto Costarricense de Investigación y Enseñanza en Nutrición y Salud	Adriana Godínez; Claudio Soto-Garita; Estela Cordero; Francisco Duarte; Hebleen Porras; Joselyn Prado & Teresita Somogyi; José Luis Vargas; Mariela Gutiérrez; Melany Calderón
EPI_ISL_1827516, EPI_ISL_2272961, EPI_ISL_2272964	HOSPITAL MONSEÑOR SANABRIA	Inciensa, Instituto Costarricense de Investigación y Enseñanza en Nutrición y Salud	Adriana Godínez; Claudio Soto-Garita; Estela Cordero; Francisco Duarte; Hebleen Porras; Joselyn Prado & Andrea Moreno-Carvajal; Joselyn Prado & María José Gómez-Umaña; José Luis Vargas; Mariela Gutiérrez; Melany Calderón
EPI_ISL_2502741	HOSPITAL SAN FRANCISCO DE ASIS	Inciensa, Instituto Costarricense de InvestigaciOn y Enseñanza en NutriciOn y Salud	Adriana Godínez; Claudio Soto-Garita; Estela Cordero; Francisco Duarte; Hebleen Porras; Jose Luis Vargas; Joselyn Prado & Adrián Fallas-Mora; Mariela Gutierrez; Melany CalderOn
EPI_ISL_2502762	HOSPITAL SAN JUAN DE DIOS	Inciensa, Instituto Costarricense de InvestigaciOn y Enseñanza en NutriciOn y Salud	Adriana Godínez; Claudio Soto-Garita; Estela Cordero; Francisco Duarte; Hebleen Porras; Jose Luis Vargas; Joselyn Prado & Marco Cháves-Otárola; Mariela Gutierrez; Melany CalderOn
EPI_ISL_2103373	HOSPITAL SAN JUAN DE DIOS	Inciensa, Instituto Costarricense de Investigación y Enseñanza en Nutrición y Salud	Adriana Godinez & Melany Calderon; Claudio Soto-Garita; Estela Cordero; Francisco Duarte; Hebleen Brenes
EPI_ISL_1469578	HOSPITAL SAO FRANCISCO DE ASSIS	Epiclin	Ana Paula Muterle; Carolina Comerlato; Eliana Márcia Da Ros Wendland; Fernando Hayashi Sant'Anna; Janira Prichula; Juliana Comerlato
EPI_ISL_412971	HUS Diagnostiikkakeskus, Hallinto	Department of Virology Faculty of Medicine, Medicum University of Helsinki	Hannimari Kallio-Kokko; Olli Vapalahti; Suvi Kuivanen; Teemu Smura
EPI_ISL_2096776	Hadassah Medical Center Clinical Virology Laboratory, Hadassah Ein Kerem	Hadassah Hebrew University Viral Sequencing Group, Hadassah Hebrew University Medical Center	Dana G. Wolf; Esther Oiknine-Djian; Hadar Golan Berman; Mila Rivkin; Sheera Adar
EPI_ISL_1831284, EPI_ISL_1831287, EPI_ISL_1831292, EPI_ISL_1831294, EPI_ISL_1831295, EPI_ISL_1831297, EPI_ISL_1831310, EPI_ISL_1831312, EPI_ISL_1831331, EPI_ISL_1831333, EPI_ISL_1831369, EPI_ISL_1831372, EPI_ISL_1831377, EPI_ISL_2005350, EPI_ISL_2005415, EPI_ISL_2005455, EPI_ISL_2005490, EPI_ISL_2005494, EPI_ISL_2005497, EPI_ISL_2005527, EPI_ISL_2005569, EPI_ISL_2005587, EPI_ISL_2006541, EPI_ISL_2006543, EPI_ISL_2006547, EPI_ISL_2006550, EPI_ISL_2006553, EPI_ISL_2006556, EPI_ISL_2006559, EPI_ISL_2006572, EPI_ISL_2006577, EPI_ISL_2006582, EPI_ISL_2006586, EPI_ISL_2006590, EPI_ISL_2006592, EPI_ISL_2006594, EPI_ISL_2006600, EPI_ISL_2006604, EPI_ISL_2006606, EPI_ISL_2006607, EPI_ISL_2006609, EPI_ISL_2006612, EPI_ISL_2006613, EPI_ISL_2006614, EPI_ISL_2006616, EPI_ISL_2006624, EPI_ISL_2006625, EPI_ISL_2006640, EPI_ISL_2006642, EPI_ISL_2006648, EPI_ISL_2006659, EPI_ISL_2006661, EPI_ISL_2022557, EPI_ISL_2022558, EPI_ISL_2022561, EPI_ISL_2022570, EPI_ISL_2022575, EPI_ISL_2080368, EPI_ISL_2080396, EPI_ISL_2080404, EPI_ISL_2080419, EPI_ISL_2080424, EPI_ISL_2080433, EPI_ISL_2080440, EPI_ISL_2080441, EPI_ISL_2080446, EPI_ISL_2080451, EPI_ISL_2080459, EPI_ISL_2080462, EPI_ISL_2080465, EPI_ISL_2080470, EPI_ISL_2253257, EPI_ISL_2348111, EPI_ISL_2447285, EPI_ISL_2468036, EPI_ISL_2469258, EPI_ISL_2512057, EPI_ISL_2512197, EPI_ISL_2512233, EPI_ISL_2513891, EPI_ISL_2537494, EPI_ISL_2552888, EPI_ISL_2553953, EPI_ISL_2638995, EPI_ISL_2641360, EPI_ISL_2682216, EPI_ISL_2682537, EPI_ISL_2766042			
see above	Health Services Laboratories	Wellcome Sanger Institute for the COVID-19 Genomics UK (COG-UK) Consortium	Cordelia Langford; David K. Jackson; Dominic Kwiatkowski; Ewan Harrison; Health Services Laboratories and Alex Alderton; Ian Johnston; Jeffrey Barrett; John Sillitoe on behalf of the Wellcome Sanger Institute COVID-19 Surveillance Team; Roberto Amato; Sonia Goncalves
EPI_ISL_2322989, EPI_ISL_2376218	Heilig hart Lier	Imeda Hospital	Dagmar Obbels; Hanne Valgaeren; Johan Frans
EPI_ISL_2293245	Heilig hart Lier	Imelda Ziekenhuis	Dagmar Obbels; Hanne Valgaeren; Johan Frans
EPI_ISL_2617253	Hellenic National Blood Transfusion Center - EKEA	Greek Genome Center, Biomedical Research Foundation of the Academy of Athens (BRFAA)	Dimitrios Thanos; Efthimia Petinaki; Emmanouil Athanasiadis; Giannis Vatsellas; Katerina Zoi; Kostas Stamoulis; Theodoros Loupis
EPI_ISL_2530115, EPI_ISL_2530117, EPI_ISL_2530119, EPI_ISL_2530132, EPI_ISL_2530134, EPI_ISL_2530143, EPI_ISL_2530148, EPI_ISL_2530160, EPI_ISL_2530161, EPI_ISL_2530163, EPI_ISL_2530169, EPI_ISL_2530174, EPI_ISL_2530179, EPI_ISL_2530185, EPI_ISL_2530186, EPI_ISL_2530191, EPI_ISL_2530193, EPI_ISL_2530195, EPI_ISL_2530196, EPI_ISL_2530198, EPI_ISL_2530199, EPI_ISL_2530201, EPI_ISL_2530202, EPI_ISL_2530204, EPI_ISL_2530206, EPI_ISL_2530207, EPI_ISL_2530209, EPI_ISL_2530210, EPI_ISL_2530212, EPI_ISL_2530214, EPI_ISL_2530217, EPI_ISL_2530219, EPI_ISL_2530221, EPI_ISL_2530222, EPI_ISL_2530224, EPI_ISL_2530226, EPI_ISL_2530227, EPI_ISL_2530229, EPI_ISL_2530230, EPI_ISL_2530232, EPI_ISL_2530233, EPI_ISL_2530235, EPI_ISL_2530236, EPI_ISL_2530239, EPI_ISL_2530241, EPI_ISL_2530243, EPI_ISL_2530245, EPI_ISL_2530246, EPI_ISL_2530248, EPI_ISL_2530265, EPI_ISL_2530299, EPI_ISL_2530304, EPI_ISL_2530308, EPI_ISL_2530319, EPI_ISL_2530373, EPI_ISL_2530374, EPI_ISL_2530378, EPI_ISL_2530379, EPI_ISL_2600958			
see above	Hematopathology Laboratory, ACTREC, TMC	Hematopathology Laboratory, ACTREC, TMC	ACTREC; Hematopathology Laboratory
EPI_ISL_1168193, EPI_ISL_1259296	Hopital	National Reference Center for Viruses of Respiratory Infections, Institut Pasteur, Paris	Angela Brisebarre; Camille Capel; Etienne Simon-Lorière; Hermann CéCile; Marion Barbet; Maud Vanpeene; Méline Bizard; Sylvie Behillil; Sylvie van der Werf; Vincent Enouf
EPI_ISL_1381196, EPI_ISL_2029931, EPI_ISL_2188352, EPI_ISL_2188418, EPI_ISL_2374451, EPI_ISL_2462927	Hospital	National Reference Center for Viruses of Respiratory Infections, Institut Pasteur, Paris	Amaury Vaysse; Angela Brisebarre; Bressollette CéLine; Camille Capel; Christophe Malabat; Corinne Maufrais; CéLine Ramanantsoa; Didier Mattera; Emmanuelle Permal; Etienne Simon-Lorière; Frédéric Lemoine; Laura Djamdjian; Louise Lefrançois; Marion Barbet; Maud Vanpeene; Méline Bizard; Nabil Gastli; Raphael Guiheneuf; Sylvie Behillil; Sylvie Van der Werf; Sylvie van der Werf; Vincent Enouf
EPI_ISL_1523274	Hospital Center Emile Mayrisch	Laboratoire national de sante, Microbiology, Microbial Genomics Platform	Anke Wienecke-Baldacchino; Catherine Ragimbeau; Cynthia Oxacelay; Fatu Djabi; Jessica Tapp; Lise Pignon; Raoul Salmon; Tamir Abdelrahman
EPI_ISL_1384137, EPI_ISL_1917450	Hospital Center Luxembourg	Laboratoire national de sante, Microbiology, Microbial Genomics Platform	Anke Wienecke-Baldacchino; Catherine Ragimbeau; Fatu Djabi; Jean-Hugues Francois; Jessica Tapp; Lise Pignon; Michel Kohnen; Raoul Salmon; Tamir Abdelrahman
EPI_ISL_769990	Hospital Dr. Rafael A. Calderon Guardia	Inciensa, Instituto Costarricense de Investigación y Enseñanza en Nutrición y Salud	Adriana Godínez; Claudio Soto-Garita; Estela Cordero; Francisco Duarte; Hebleen Porras; Melany Calderón & Mariel López
EPI_ISL_1527015	Hospital Guápiles	Inciensa, Instituto Costarricense de Investigación y Enseñanza en Nutrición y Salud	Barboza-Arguedas E & Cerdas-Quesada C; Pérez-Corrales C
EPI_ISL_2014355	Hospital Herrera Llerandi	Asociación de Salud Integral/Clínica Familiar Luis Ángel García	Ana S. Gonzalez-Reiche; Claudia Rangel; Danicela Mercado; Eduardo Arathoon; Hilda Ruiz; Luis Aguirre; Luis Rivas; Narda Medina; Oscar Bonilla; Osmar Gamboa.
EPI_ISL_481247	Hospital IESS Babahoyo	Institute of Microbiology, Universidad San Francisco de Quito	Belén Prado-Vivar; Bernardo Gutiérrez; Carla Torres; Francisco Cordova; Gabriel Trueba; Juan José Guadalupe; Killen Briones-Claudette; Killen Briones-Zamora; Michelle Grunauer; Monica Becerra-Wong; Ninfa Henriquez; Patricio Rojas-Silva; Paúl Cárdenas; Sully Márquez; Verónica Barragán
EPI_ISL_413016	Hospital Israelita Albert Einstein	Instituto Adolfo Lutz, Interdisciplinary Procedures Center, Strategic Laboratory	Andrew Rambaut; Claudia Regina Gonçalves; Claudio Tavares Sacchi; Ester Cerdeira Sabino; Fabiana Cristina Pereira dos Santos; Flávia Cristina da Silva Sales; Ingra Morales Claro; Jaqueline Goes de Jesus; Joshua Quick; Maria do Carmo Sampaio Tavares Timenetsky; Nicholas James Loman; Nuno Rodrigues Faria
EPI_ISL_527739	Hospital Mexico [San Jose/San Jose]	Inciensa, Instituto Costarricense de Investigación y Enseñanza en Nutrición y Salud	Adriana Godinez & Melany Calderon; Claudio Soto-Garita; Estela Cordero; Francisco Duarte; Hebleen Porras
EPI_ISL_414016	Hospital São Joaquim Beneficencia Portuguesa	Instituto Adolfo Lutz, Interdiciplinary Procedures Center, Strategic Laboratory	Audrey Cilli; Carlos Henrique Camargo; Claudia Regina Gonçalves; Claudio Tavares Sacchi; Daniela Bernardes Borges da Silva; Ester Cerdeira Sabino; Maria do Carmo Sampaito Tavares Timenetsky; Terezinha Maria de Paiva
EPI_ISL_2233095	Hotel Horison	National Institute of Health Research and Development	Arie Ardiansyah Nugraha; Hana Apsari Pawestri; Hartanti Dian Ikawati; Kartika Dewi Puspa; Krisna Pangesti; Nelly Puspandari; Subangkit; Triyani Soekarso; Vivi Setiawaty
EPI_ISL_2466656	Human Genetic Research Center, Kawsar Biotech Company	Human Genetic Research Center, Kawsar Biotech Company	Abbasalipour Bashash, M.; Azadmanesh, K.; Chamran Hospital, C.; Khosravi; M.A.; National Institute for Medical Research Development, N.; Zeinali, R.; Zeinali, S.
EPI_ISL_2466657, EPI_ISL_2466658	Human Genetic Research Center, Kawsar Biotech Company	Kawsar Biotech Company	Abbasalipour Bashash, M.; Abbasalipour, M.; Azadmanesh, K.; Chamran Hospital, C.; Dabagh, S.; Keshvar, Y.; Khosravi; M.A.; National Institute for Medical Research Development, N.; Sabeghi, S.; Sharifi, Z.; Shirzad, T.; Zeinali, R.; Zeinali, S.
EPI_ISL_526225, EPI_ISL_1041208	Hungarian Defence Forces Military Medical Centre	National Laboratory of Virology, Szentágothai Research Centre	Balázs Somogyi; Bálint Eszenyi; Csaba Pereszlényi; Endre Gábor Tóth; Ferenc Jakab; Gábor Kemenesi; Ágnes Balázs-Nagy
EPI_ISL_2151871	Hôpital Avicenne	Department of Virology, Henri Mondor University Hospital, Assistance Publique Hôpitaux de Paris, Université Paris-Est Créteil, INSERM U955	Alexandre Soulier; Christophe Rodriguez; Elisabeth Trawinski; Guillaume Gricourt; Jean-Michel Pawlotsky; Melissa N'Debi; Slim Fourati; Vanessa Demontant
EPI_ISL_2688682	Hôpital Jacques Monod	Centre Hospitalier Universitaire de Rouen Laboratoire de Virologie	Alice Moisan; Fabienne De Oliveira; Marie Leoz
EPI_ISL_2348923, EPI_ISL_2348924, EPI_ISL_2348925	Hôpitaux Iris Sud Etterbeek-Ixelles	UCLouvain/IREC/MBLG	Benoit Kabamba Mukadi; Jean Ruelle; Nicolas Pinte; Ophélie Simon
EPI_ISL_1477140, EPI_ISL_1477146	Hôpitaux Robert Schuman	Laboratoire national de sante, Microbiology, Microbial Genomics Platform	Alain Hakim; Anke Wienecke-Baldacchino; Catherine Ragimbeau; Fatu Djabi; Jessica Tapp; Lise Pignon; Raoul Salmon; Tamir Abdelrahman
EPI_ISL_2100187, EPI_ISL_2100188, EPI_ISL_2100190, EPI_ISL_2100194, EPI_ISL_2100195, EPI_ISL_2100216, EPI_ISL_2361845, EPI_ISL_2361873			
see above	ICMR-National Institute for Research In Tuberculosis	NIV Influenza	Dr Luke Elizabeth Hanna; Dr Nandhini Palani; Dr Padmapriyadarsini.C; Dr Radha Gopalaswamy; Dr S Siva Kumar; Mr Anbazagan Selvaraj; Mr Ramesh K
EPI_ISL_2189572, EPI_ISL_2189596, EPI_ISL_2189638, EPI_ISL_2189639, EPI_ISL_2189641, EPI_ISL_2189642, EPI_ISL_2189643, EPI_ISL_2189644, EPI_ISL_2189646, EPI_ISL_2189649, EPI_ISL_2189650, EPI_ISL_2189652, EPI_ISL_2189654, EPI_ISL_2189656, EPI_ISL_2189657, EPI_ISL_2189658, EPI_ISL_2189662, EPI_ISL_2189663, EPI_ISL_2189669, EPI_ISL_2189670, EPI_ISL_2189676, EPI_ISL_2189679, EPI_ISL_2189686, EPI_ISL_2189691, EPI_ISL_2189697, EPI_ISL_2189698, EPI_ISL_2189699, EPI_ISL_2189704, EPI_ISL_2189705, EPI_ISL_2189708, EPI_ISL_2189710, EPI_ISL_2189711, EPI_ISL_2189712, EPI_ISL_2189713, EPI_ISL_2189716, EPI_ISL_2189717, EPI_ISL_2189718, EPI_ISL_2189723, EPI_ISL_2189724, EPI_ISL_2189726, EPI_ISL_2189728, EPI_ISL_2189738, EPI_ISL_2189744, EPI_ISL_2189748, EPI_ISL_2189749, EPI_ISL_2189755, EPI_ISL_2189761, EPI_ISL_2189765, EPI_ISL_2189766, EPI_ISL_2189768, EPI_ISL_2189770, EPI_ISL_2189773, EPI_ISL_2189778, EPI_ISL_2189780, EPI_ISL_2189786, EPI_ISL_2189787, EPI_ISL_2189788, EPI_ISL_2189791, EPI_ISL_2189792, EPI_ISL_2189795, EPI_ISL_2189796, EPI_ISL_2189800, EPI_ISL_2189803, EPI_ISL_2189806, EPI_ISL_2189809, EPI_ISL_2189810, EPI_ISL_2189811, EPI_ISL_2189813, EPI_ISL_2189814, EPI_ISL_2189817, EPI_ISL_2189818, EPI_ISL_2189819, EPI_ISL_2189823, EPI_ISL_2189825, EPI_ISL_2189827, EPI_ISL_2189828, EPI_ISL_2189829, EPI_ISL_2189830, EPI_ISL_2189831, EPI_ISL_2189834, EPI_ISL_2189836, EPI_ISL_2189837, EPI_ISL_2189840, EPI_ISL_2189841, EPI_ISL_2189843, EPI_ISL_2189845, EPI_ISL_2189849, EPI_ISL_2189850, EPI_ISL_2189852, EPI_ISL_2189857, EPI_ISL_2189859, EPI_ISL_2189860, EPI_ISL_2189863, EPI_ISL_2555672, EPI_ISL_2555696, EPI_ISL_2555701, EPI_ISL_2555714, EPI_ISL_2555716, EPI_ISL_2555720, EPI_ISL_2555724, EPI_ISL_2555732			
see above	ICMR-National Institute of Virology	NCDC Delhi, Biotechnology Division	A Walimbe; A.Awhale; A.Titkare; B.Apoorva; G.Divekar; H.Kengale; Hema Gogia; Hemlata Lall; K P.Shinde; K.Iyengar; K.Korabu; Kalaiarasan Ponnusamy; M L Choudhary; M.Das; Mahesh S Dhar; Manoj K Singh; Meena Datta; Partha Rakshit; Preeti Madan; Priyanka Singh; R.Verma; Radhakrishnan V. S; Robin Marwal; S Jadhav; S.Bhorekar; S.Jadhav; S.Shelkande; Sandhya Kabra; Sujeet K Singh; T.Sanjeevi; U.Saha; Uma Sharma; V Malik; V.Autude; V.Vipat; Z. Sayyed
EPI_ISL_1703662, EPI_ISL_1703668, EPI_ISL_1703740, EPI_ISL_1703878, EPI_ISL_1704234, EPI_ISL_1704561, EPI_ISL_1704605, EPI_ISL_1704617, EPI_ISL_1704618, EPI_ISL_1704620, EPI_ISL_1704625, EPI_ISL_1704626, EPI_ISL_1704627, EPI_ISL_1704628, EPI_ISL_1704629, EPI_ISL_1704630, EPI_ISL_1704632, EPI_ISL_1704634, EPI_ISL_1704635, EPI_ISL_1704636, EPI_ISL_1704637, EPI_ISL_1704638, EPI_ISL_1704639, EPI_ISL_1704642, EPI_ISL_1841240, EPI_ISL_1841241, EPI_ISL_1841242, EPI_ISL_1841243, EPI_ISL_1841244, EPI_ISL_1841245, EPI_ISL_1841246, EPI_ISL_1841247, EPI_ISL_1841248, EPI_ISL_1841249, EPI_ISL_1841250, EPI_ISL_1841251, EPI_ISL_1841252, EPI_ISL_1841253, EPI_ISL_1841254, EPI_ISL_1841255, EPI_ISL_1841256, EPI_ISL_1841257, EPI_ISL_1841258, EPI_ISL_1841259, EPI_ISL_1841260, EPI_ISL_1841261, EPI_ISL_1841262, EPI_ISL_1841263, EPI_ISL_1841264, EPI_ISL_1841265, EPI_ISL_1841266, EPI_ISL_1841267, EPI_ISL_1841268, EPI_ISL_1841269, EPI_ISL_1841270, EPI_ISL_1841271, EPI_ISL_1841272, EPI_ISL_1841273, EPI_ISL_1841274, EPI_ISL_1841275, EPI_ISL_1841276, EPI_ISL_1841279, EPI_ISL_1841280, EPI_ISL_1841281, EPI_ISL_1841282, EPI_ISL_1841283,			

EPI_ISL_1841284, EPI_ISL_1841285, EPI_ISL_1841293, EPI_ISL_1841317, EPI_ISL_1841318, EPI_ISL_1841355, EPI_ISL_1841356, EPI_ISL_1841357, EPI_ISL_1841365, EPI_ISL_1841366, EPI_ISL_1841367, EPI_ISL_1841370, EPI_ISL_1841371, EPI_ISL_1841372, EPI_ISL_1841373, EPI_ISL_1841374, EPI_ISL_1841375, EPI_ISL_1841382, EPI_ISL_1841383, EPI_ISL_1928407, EPI_ISL_1928417, EPI_ISL_1928439, EPI_ISL_1928457, EPI_ISL_1928462, EPI_ISL_1928477, EPI_ISL_1928484, EPI_ISL_1928506, EPI_ISL_1928507, EPI_ISL_1928509, EPI_ISL_1928512, EPI_ISL_1928513, EPI_ISL_1928515, EPI_ISL_1928520, EPI_ISL_1928522, EPI_ISL_1928525, EPI_ISL_1928527, EPI_ISL_1928530, EPI_ISL_1928531, EPI_ISL_1928534, EPI_ISL_1928536, EPI_ISL_1928537, EPI_ISL_1928539, EPI_ISL_1928540, EPI_ISL_1928541, EPI_ISL_1928543, EPI_ISL_1928544, EPI_ISL_1928547, EPI_ISL_1928549, EPI_ISL_1928550, EPI_ISL_1928552, EPI_ISL_1928553, EPI_ISL_1928555, EPI_ISL_1928556, EPI_ISL_1928558, EPI_ISL_1928559, EPI_ISL_1928561, EPI_ISL_1928562, EPI_ISL_1928565, EPI_ISL_1928566, EPI_ISL_1928568, EPI_ISL_1928569, EPI_ISL_1928571, EPI_ISL_1928572, EPI_ISL_1928573, EPI_ISL_1928575, EPI_ISL_1928576, EPI_ISL_1928578, EPI_ISL_1928579, EPI_ISL_1928581, EPI_ISL_1928583, EPI_ISL_1928584, EPI_ISL_1928586, EPI_ISL_1928588, EPI_ISL_1928590, EPI_ISL_1928592, EPI_ISL_1928593, EPI_ISL_1928595, EPI_ISL_1928596, EPI_ISL_1928598, EPI_ISL_1928599, EPI_ISL_1928601, EPI_ISL_1928602, EPI_ISL_1928603, EPI_ISL_1928605, EPI_ISL_1928607, EPI_ISL_1928608, EPI_ISL_1928610, EPI_ISL_1928611, EPI_ISL_1928613, EPI_ISL_1928614, EPI_ISL_1928615, EPI_ISL_1928617, EPI_ISL_1928618, EPI_ISL_1928620, EPI_ISL_1928622, EPI_ISL_1928623, EPI_ISL_1928624, EPI_ISL_1928626, EPI_ISL_1928627, EPI_ISL_1928629, EPI_ISL_1928631, EPI_ISL_1928632, EPI_ISL_1928633, EPI_ISL_1928635, EPI_ISL_1928636, EPI_ISL_1928638, EPI_ISL_1928639, EPI_ISL_1928641, EPI_ISL_1928642, EPI_ISL_1928645, EPI_ISL_1928647, EPI_ISL_1928648, EPI_ISL_1928650, EPI_ISL_1970422, EPI_ISL_1970427, EPI_ISL_1970429, EPI_ISL_1970431, EPI_ISL_1970433, EPI_ISL_1970434, EPI_ISL_1970435, EPI_ISL_1970437, EPI_ISL_1970438, EPI_ISL_1970439, EPI_ISL_1970440, EPI_ISL_1970441, EPI_ISL_1970442, EPI_ISL_1970443, EPI_ISL_1970444, EPI_ISL_1970445, EPI_ISL_1970447, EPI_ISL_1970448, EPI_ISL_1970449, EPI_ISL_1970450, EPI_ISL_1970451, EPI_ISL_1970452, EPI_ISL_1970453, EPI_ISL_1970454, EPI_ISL_1970455, EPI_ISL_1970456, EPI_ISL_1970457, EPI_ISL_1970458, EPI_ISL_1970459, EPI_ISL_1970461, EPI_ISL_1970462, EPI_ISL_1970463, EPI_ISL_1970464, EPI_ISL_1970465, EPI_ISL_1970466, EPI_ISL_1970467, EPI_ISL_1970468, EPI_ISL_1970469, EPI_ISL_1970470, EPI_ISL_1970471, EPI_ISL_1970472, EPI_ISL_1970473, EPI_ISL_1970474, EPI_ISL_1970475, EPI_ISL_1970476, EPI_ISL_1970477, EPI_ISL_1970478, EPI_ISL_1970479, EPI_ISL_1970480, EPI_ISL_1970481, EPI_ISL_1970482, EPI_ISL_1970484, EPI_ISL_1970485, EPI_ISL_1970486, EPI_ISL_1970488, EPI_ISL_1970489, EPI_ISL_1970491, EPI_ISL_1970492, EPI_ISL_1970493, EPI_ISL_1970494, EPI_ISL_1970495, EPI_ISL_1970496, EPI_ISL_1970497, EPI_ISL_1970499, EPI_ISL_1970500, EPI_ISL_1970501, EPI_ISL_1970502, EPI_ISL_1970503, EPI_ISL_1970504, EPI_ISL_1970505, EPI_ISL_1970506, EPI_ISL_1970507, EPI_ISL_1970508, EPI_ISL_1970509, EPI_ISL_1970510, EPI_ISL_1970511, EPI_ISL_1970512, EPI_ISL_1970513, EPI_ISL_1970514, EPI_ISL_1970515, EPI_ISL_1970517, EPI_ISL_1970518, EPI_ISL_1970519, EPI_ISL_1970520, EPI_ISL_1970522, EPI_ISL_1970523, EPI_ISL_1970524, EPI_ISL_1970525, EPI_ISL_1970526, EPI_ISL_1970527, EPI_ISL_1970529, EPI_ISL_1970530, EPI_ISL_1970531, EPI_ISL_1970534, EPI_ISL_1970535, EPI_ISL_1970536, EPI_ISL_1970538, EPI_ISL_1970540, EPI_ISL_1970541, EPI_ISL_1970542, EPI_ISL_1970543, EPI_ISL_1970544, EPI_ISL_1970545, EPI_ISL_1970546, EPI_ISL_2131509, EPI_ISL_2131510, EPI_ISL_2131514, EPI_ISL_2131538, EPI_ISL_2131540, EPI_ISL_2131541, EPI_ISL_2131542, EPI_ISL_2131543, EPI_ISL_2131544, EPI_ISL_2131546, EPI_ISL_2131555, EPI_ISL_2131556, EPI_ISL_2131557, EPI_ISL_2131558, EPI_ISL_2131559, EPI_ISL_2131560, EPI_ISL_2131561, EPI_ISL_2131562, EPI_ISL_2131564, EPI_ISL_2131565, EPI_ISL_2546013, EPI_ISL_2546023, EPI_ISL_2546034, EPI_ISL_2546053, EPI_ISL_2546063, EPI_ISL_2546068, EPI_ISL_2546078, EPI_ISL_2546085, EPI_ISL_2546089, EPI_ISL_2546099, EPI_ISL_2546102, EPI_ISL_2546104, EPI_ISL_2546106, EPI_ISL_2546107, EPI_ISL_2546122, EPI_ISL_2546136, EPI_ISL_2546145, EPI_ISL_2546154, EPI_ISL_2546156, EPI_ISL_2546157, EPI_ISL_2546158, EPI_ISL_2546163, EPI_ISL_2546164, EPI_ISL_2546187, EPI_ISL_2546228, EPI_ISL_2546230, EPI_ISL_2546233, EPI_ISL_2546275, EPI_ISL_2546276, EPI_ISL_2546278, EPI_ISL_2546279, EPI_ISL_2546281, EPI_ISL_2546289, EPI_ISL_2546291, EPI_ISL_2546294, EPI_ISL_2546295, EPI_ISL_2546297, EPI_ISL_2546298, EPI_ISL_2546304, EPI_ISL_2546308, EPI_ISL_2546321, EPI_ISL_2546327, EPI_ISL_2546330, EPI_ISL_2546336, EPI_ISL_2546337, EPI_ISL_2546349, EPI_ISL_2546350, EPI_ISL_2546356, EPI_ISL_2546357, EPI_ISL_2546359, EPI_ISL_2546360, EPI_ISL_2546362, EPI_ISL_2546364, EPI_ISL_2546365, EPI_ISL_2546366, EPI_ISL_2546367, EPI_ISL_2546368, EPI_ISL_2546374, EPI_ISL_2546375, EPI_ISL_2546381, EPI_ISL_2546387, EPI_ISL_2546388, EPI_ISL_2546389, EPI_ISL_2546396, EPI_ISL_2546397, EPI_ISL_2546401, EPI_ISL_2546403, EPI_ISL_2546411, EPI_ISL_2546413, EPI_ISL_2546414, EPI_ISL_2546416, EPI_ISL_2546417, EPI_ISL_2546418, EPI_ISL_2546419, EPI_ISL_2546422, EPI_ISL_2546425, EPI_ISL_2546431, EPI_ISL_2546432, EPI_ISL_2546434, EPI_ISL_2546435, EPI_ISL_2546437, EPI_ISL_2546438, EPI_ISL_2546443, EPI_ISL_2546444, EPI_ISL_2546447, EPI_ISL_2546487, EPI_ISL_2546489, EPI_ISL_2546490, EPI_ISL_2546493, EPI_ISL_2546495, EPI_ISL_2546496, EPI_ISL_2546497, EPI_ISL_2546499, EPI_ISL_2546500, EPI_ISL_2546501, EPI_ISL_2546502, EPI_ISL_2546504, EPI_ISL_2546505, EPI_ISL_2546506, EPI_ISL_2546508, EPI_ISL_2546509, EPI_ISL_2546510, EPI_ISL_2546511, EPI_ISL_2546512, EPI_ISL_2546514, EPI_ISL_2546515, EPI_ISL_2546516, EPI_ISL_2546518, EPI_ISL_2546520, EPI_ISL_2546522, EPI_ISL_2546523, EPI_ISL_2546524, EPI_ISL_2546526, EPI_ISL_2546527, EPI_ISL_2546528, EPI_ISL_2546529, EPI_ISL_2546530, EPI_ISL_2546532, EPI_ISL_2546534, EPI_ISL_2546536, EPI_ISL_2546537, EPI_ISL_2546539, EPI_ISL_2546540, EPI_ISL_2546541, EPI_ISL_2546542, EPI_ISL_2546543, EPI_ISL_2546544, EPI_ISL_2546547, EPI_ISL_2546548, EPI_ISL_2546549, EPI_ISL_2546550, EPI_ISL_2546552, EPI_ISL_2546553, EPI_ISL_2546555, EPI_ISL_2546558, EPI_ISL_2546559, EPI_ISL_2546562, EPI_ISL_2546563, EPI_ISL_2546564, EPI_ISL_2546565, EPI_ISL_2546566, EPI_ISL_2546567, EPI_ISL_2546568, EPI_ISL_2546569, EPI_ISL_2546570, EPI_ISL_2546572, EPI_ISL_2546576, EPI_ISL_2546577, EPI_ISL_2546578, EPI_ISL_2546580, EPI_ISL_2546581, EPI_ISL_2546582, EPI_ISL_2546583, EPI_ISL_2546584, EPI_ISL_2546585, EPI_ISL_2546586, EPI_ISL_2546587, EPI_ISL_2546589, EPI_ISL_2546591, EPI_ISL_2546592, EPI_ISL_2546593, EPI_ISL_2546594, EPI_ISL_2546595, EPI_ISL_2546596, EPI_ISL_2546597, EPI_ISL_2546598, EPI_ISL_2546599, EPI_ISL_2546600, EPI_ISL_2546601, EPI_ISL_2546602, EPI_ISL_2546603, EPI_ISL_2546604, EPI_ISL_2546605, EPI_ISL_2546606, EPI_ISL_2546607, EPI_ISL_2546609, EPI_ISL_2546610, EPI_ISL_2546611, EPI_ISL_2546612, EPI_ISL_2546613, EPI_ISL_2546614, EPI_ISL_2546615, EPI_ISL_2546616, EPI_ISL_2546617, EPI_ISL_2546619, EPI_ISL_2546620, EPI_ISL_2546622, EPI_ISL_2546623, EPI_ISL_2546624, EPI_ISL_2546625, EPI_ISL_2546627, EPI_ISL_2546628, EPI_ISL_2546630, EPI_ISL_2546631, EPI_ISL_2546632, EPI_ISL_2546633, EPI_ISL_2546634, EPI_ISL_2546635, EPI_ISL_2546636, EPI_ISL_2546638, EPI_ISL_2546639, EPI_ISL_2546640, EPI_ISL_2546641, EPI_ISL_2546642, EPI_ISL_2546644, EPI_ISL_2546645, EPI_ISL_2546646, EPI_ISL_2546647, EPI_ISL_2546648, EPI_ISL_2546649, EPI_ISL_2546650, EPI_ISL_2546652, EPI_ISL_2546653, EPI_ISL_2546654, EPI_ISL_2546655, EPI_ISL_2546656, EPI_ISL_2546657, EPI_ISL_2546658, EPI_ISL_2546659, EPI_ISL_2546660, EPI_ISL_2546661, EPI_ISL_2546682, EPI_ISL_2546683, EPI_ISL_2546684, EPI_ISL_2546685, EPI_ISL_2546686, EPI_ISL_2546687, EPI_ISL_2546698, EPI_ISL_2546699, EPI_ISL_2546700, EPI_ISL_2546702, EPI_ISL_2546703, EPI_ISL_2546704, EPI_ISL_2546705, EPI_ISL_2546707, EPI_ISL_2546708, EPI_ISL_2546709, EPI_ISL_2546829, EPI_ISL_2547074, EPI_ISL_2547076, EPI_ISL_2547078, EPI_ISL_2547136, EPI_ISL_2547153, EPI_ISL_2547166, EPI_ISL_2547167, EPI_ISL_2547212, EPI_ISL_2547262, EPI_ISL_2547277, EPI_ISL_2547282			
see above	ICMR-National Institute of Virology - INSACOG	NIV Influenza	Dr. Varsha Potdar; Dr. Varsha Potdar and NIC Team
EPI_ISL_2621161, EPI_ISL_2621162, EPI_ISL_2621167	ICMT-APARTADO	Universidad Nacional de Colombia - Laboratorio Genómico One Health	Andres F. Cardona-Rios; Carlos Franco-Muñoz; Carolina Muñoz-Arango; Celeny Ortiz; Daniel O. Maldonado-Perez; Diego A. Álvarez-Díaz; Hector Alejandro Ruiz-Moreno; Idabely Betancur Ortiz; Jorge E. Osorio; Juan P. Hernandez-Ortiz; Karl A Ciuoderis; Katherine Laiton-Donato; Laura Silvana Perez; Lina M. Hurtado; Marcela Mercado-Reyes; Maria Angélica Maya; Maria Stella López; Rita Almanza Payares; Sandra Ines Cano; Simón Villegas Velásquez
EPI_ISL_1825677	IHU Méditerranée Infection	IHU Méditerranée Infection	Bernard La Scola et al.
EPI_ISL_1217401, EPI_ISL_2637195, EPI_ISL_2637207	IMD - Labor Oderland	Robert Koch Institute	
EPI_ISL_1282363	IMD - MVZ Labor Greifswald	Robert Koch Institute	
EPI_ISL_1722241	IMD - MVZ Labor Martinsried	Robert Koch Institute	
EPI_ISL_1848402, EPI_ISL_2112834	IMD Labor Frankfurt	Robert Koch Institute	
EPI_ISL_2000617, EPI_ISL_2000619, EPI_ISL_2000620, EPI_ISL_2000621, EPI_ISL_2000622, EPI_ISL_2000623, EPI_ISL_2000624, EPI_ISL_2000625, EPI_ISL_2000626, EPI_ISL_2000627			
see above	INMI Lazzaro Spallanzani IRCCS	INMI Lazzaro Spallanzani IRCCS	A Di Caro; B Bartolini; CEM Gruber; E Giombini; F Messina; F Santini; G Bonfiglio; M Rueca; MR Capobianchi; O Butera
EPI_ISL_410545	INMI Lazzaro Spallanzani IRCCS	Laboratory of Virology, INMI Lazzaro Spallanzani IRCCS	Barbara Bartolini; Cesare E. M. Gruber; Concetta Castilletti; Eleonora Lalle; Emanuela Giombini; Emanuele Nicastri; Fabrizio Carletti; Francesca Colavita; Francesco Messina; Giuseppe Ippolito.; Maria R. Capobianchi; Martina Rueca
EPI_ISL_2503826, EPI_ISL_2503827, EPI_ISL_2503828, EPI_ISL_2503831, EPI_ISL_2503832, EPI_ISL_2503833, EPI_ISL_2503834, EPI_ISL_2503835, EPI_ISL_2503836			
see above	INSACOG-ARUNACHAL PRADESH	National Institute of Biomedical Genomics – INSACOG	Arindam Maitra; Lobsang Jampa; Mika Umpo; Nidhan Kumar Biswas; Saumitra Das; Sreedhar Chinnaswamy; Yompe Kamki
EPI_ISL_2502308, EPI_ISL_2502309, EPI_ISL_2502311, EPI_ISL_2502312, EPI_ISL_2502319, EPI_ISL_2502321, EPI_ISL_2502322, EPI_ISL_2502323, EPI_ISL_2502326, EPI_ISL_2502327, EPI_ISL_2502329, EPI_ISL_2502330, EPI_ISL_2502331, EPI_ISL_2502332, EPI_ISL_2502333, EPI_ISL_2502334, EPI_ISL_2502335, EPI_ISL_2502336, EPI_ISL_2502337, EPI_ISL_2502338, EPI_ISL_2502339, EPI_ISL_2502340, EPI_ISL_2502342, EPI_ISL_2502343, EPI_ISL_2502344, EPI_ISL_2502345, EPI_ISL_2502346, EPI_ISL_2502347, EPI_ISL_2502350, EPI_ISL_2502351, EPI_ISL_2502352, EPI_ISL_2502355, EPI_ISL_2502356, EPI_ISL_2502357, EPI_ISL_2502358, EPI_ISL_2502359, EPI_ISL_2502361, EPI_ISL_2502363, EPI_ISL_2502364, EPI_ISL_2502365, EPI_ISL_2502367, EPI_ISL_2502369			
see above	INSACOG-Assam	National Institute of Biomedical Genomics – INSACOG	Ajanta Sharma; Arindam Maitra; Kailash Chamuah; Lahari Saikia; Nidhan Kumar Biswas; Saumitra Das; Sreedhar Chinnaswamy
EPI_ISL_2547439, EPI_ISL_2547440, EPI_ISL_2547441, EPI_ISL_2547444, EPI_ISL_2547445, EPI_ISL_2547447, EPI_ISL_2547448, EPI_ISL_2547450, EPI_ISL_2547451, EPI_ISL_2547459, EPI_ISL_2547460, EPI_ISL_2547461, EPI_ISL_2547464, EPI_ISL_2547465, EPI_ISL_2547466, EPI_ISL_2547467, EPI_ISL_2547468, EPI_ISL_2547469, EPI_ISL_2547470, EPI_ISL_2547471, EPI_ISL_2547474, EPI_ISL_2547485, EPI_ISL_2547486, EPI_ISL_2547490, EPI_ISL_2547491, EPI_ISL_2547493, EPI_ISL_2547494, EPI_ISL_2547495, EPI_ISL_2547496, EPI_ISL_2547497, EPI_ISL_2547498, EPI_ISL_2547499, EPI_ISL_2547501, EPI_ISL_2547502, EPI_ISL_2547503, EPI_ISL_2547505, EPI_ISL_2547506, EPI_ISL_2547507, EPI_ISL_2547509, EPI_ISL_2547511, EPI_ISL_2547512, EPI_ISL_2547513, EPI_ISL_2547515, EPI_ISL_2547516, EPI_ISL_2547517, EPI_ISL_2547518, EPI_ISL_2547519, EPI_ISL_2547520, EPI_ISL_2547521			
see above	INSACOG-MEGHALAYA	National Institute of Biomedical Genomics – INSACOG	Arindam Maitra; E Marak; E Shadap; Nidhan Kumar Biswas; R. Lyngdoh; Saumitra Das; Sreedhar Chinnaswamy
EPI_ISL_1419092, EPI_ISL_2521601, EPI_ISL_2521605, EPI_ISL_2521606, EPI_ISL_2521609	INSACOG-Mizoram	National Institute of Biomedical Genomics – INSACOG	Arindam Maitra; Gracy Laldinmawii; N Senthil Kumar; Nidhan Kumar Biswas; Saumitra Das; Sreedhar Chinnaswamy; Swagnik Roy
EPI_ISL_2675305, EPI_ISL_2675306, EPI_ISL_2675358, EPI_ISL_2675359, EPI_ISL_2675365, EPI_ISL_2675372, EPI_ISL_2675375, EPI_ISL_2675377, EPI_ISL_2675378, EPI_ISL_2675379, EPI_ISL_2675381, EPI_ISL_2675385, EPI_ISL_2675387, EPI_ISL_2675389, EPI_ISL_2675392			
see above	INSACOG-NAGALAND	National Institute of Biomedical Genomics – INSACOG	Arindam Maitra; Chenole Keppen; Nidhan Kumar Biswas; Saumitra Das; Sreedhar Chinnaswamy; Vinotsole Khamo; Wetetsho Kapfo
EPI_ISL_2151096, EPI_ISL_2151097, EPI_ISL_2151098, EPI_ISL_2151099, EPI_ISL_2151100, EPI_ISL_2151101, EPI_ISL_2151102			
see above	INSACOG-Sikkim	National Institute of Biomedical Genomics – INSACOG	Arindam Maitra; Kaden Zangmu Bhutia; Nidhan Kumar Biswas; Saumitra Das; Sreedhar Chinnaswamy Tshering Ongmu Bhutia; Tara D Sharma
EPI_ISL_2433259, EPI_ISL_2433260, EPI_ISL_2433268, EPI_ISL_2433269, EPI_ISL_2433276	INSACOG-Tripura	National Institute of Biomedical Genomics – INSACOG	Apurba Sarkar; Arindam Maitra; Nidhan Kumar Biswas; Saikat Majumder; Saumitra Das; Sreedhar Chinnaswamy; Tapan Majumdar
EPI_ISL_1419152, EPI_ISL_1419365, EPI_ISL_1419594, EPI_ISL_1589660, EPI_ISL_1589667, EPI_ISL_1589674, EPI_ISL_1589690, EPI_ISL_1589718, EPI_ISL_1589824, EPI_ISL_1589829, EPI_ISL_1589834, EPI_ISL_1589835, EPI_ISL_1589867, EPI_ISL_1589868, EPI_ISL_1589869, EPI_ISL_1589870, EPI_ISL_1589871, EPI_ISL_1589872, EPI_ISL_2400135, EPI_ISL_2400138, EPI_ISL_2400139, EPI_ISL_2400143, EPI_ISL_2400144, EPI_ISL_2400148, EPI_ISL_2400149, EPI_ISL_2400151, EPI_ISL_2400153, EPI_ISL_2400154, EPI_ISL_2400156, EPI_ISL_2400157, EPI_ISL_2400158, EPI_ISL_2400159, EPI_ISL_2400160, EPI_ISL_2400161, EPI_ISL_2400166, EPI_ISL_2400167, EPI_ISL_2400170, EPI_ISL_2400171, EPI_ISL_2400177, EPI_ISL_2400178, EPI_ISL_2400181, EPI_ISL_2400182, EPI_ISL_2400183, EPI_ISL_2400186, EPI_ISL_2400188, EPI_ISL_2400198, EPI_ISL_2400199, EPI_ISL_2400201, EPI_ISL_2400204, EPI_ISL_2400205, EPI_ISL_2400206, EPI_ISL_2400208, EPI_ISL_2400212, EPI_ISL_2400213, EPI_ISL_2400215, EPI_ISL_2400220, EPI_ISL_2400221, EPI_ISL_2400222, EPI_ISL_2400223, EPI_ISL_2400225, EPI_ISL_2433277, EPI_ISL_2433278, EPI_ISL_2433279, EPI_ISL_2503151, EPI_ISL_2503156, EPI_ISL_2503162, EPI_ISL_2503165, EPI_ISL_2503169, EPI_ISL_2503188, EPI_ISL_2503189, EPI_ISL_2503190, EPI_ISL_2503193, EPI_ISL_2503194, EPI_ISL_2503195, EPI_ISL_2503204, EPI_ISL_2503207, EPI_ISL_2503226, EPI_ISL_2503235, EPI_ISL_2503236, EPI_ISL_2503241, EPI_ISL_2503246, EPI_ISL_2503253, EPI_ISL_2503257, EPI_ISL_2503261, EPI_ISL_2503262, EPI_ISL_2503263, EPI_ISL_2503270, EPI_ISL_2503274, EPI_ISL_2503277, EPI_ISL_2503287, EPI_ISL_2503289, EPI_ISL_2503290, EPI_ISL_2503291, EPI_ISL_2503294, EPI_ISL_2503295, EPI_ISL_2503300, EPI_ISL_2503301, EPI_ISL_2503304, EPI_ISL_2503307, EPI_ISL_2503308, EPI_ISL_2503312, EPI_ISL_2503314, EPI_ISL_2503315, EPI_ISL_2503320, EPI_ISL_2503321, EPI_ISL_2503329, EPI_ISL_2503330, EPI_ISL_2503335, EPI_ISL_2503339, EPI_ISL_2503341, EPI_ISL_2503342, EPI_ISL_2503343, EPI_ISL_2503346, EPI_ISL_2503348, EPI_ISL_2503349, EPI_ISL_2503350, EPI_ISL_2503351, EPI_ISL_2503353, EPI_ISL_2503359, EPI_ISL_2503362, EPI_ISL_2503363, EPI_ISL_2503367, EPI_ISL_2503399, EPI_ISL_2503401, EPI_ISL_2503402, EPI_ISL_2503409, EPI_ISL_2503415, EPI_ISL_2503429, EPI_ISL_2503474, EPI_ISL_2503480, EPI_ISL_2503516, EPI_ISL_2503517, EPI_ISL_2503519, EPI_ISL_2503520, EPI_ISL_2503521, EPI_ISL_2503522, EPI_ISL_2503523, EPI_ISL_2503524, EPI_ISL_2503525, EPI_ISL_2503526, EPI_ISL_2503527, EPI_ISL_2503528, EPI_ISL_2503529, EPI_ISL_2503530, EPI_ISL_2503532, EPI_ISL_2503535, EPI_ISL_2503538, EPI_ISL_2503547, EPI_ISL_2503572, EPI_ISL_2503577, EPI_ISL_2503578, EPI_ISL_2503579, EPI_ISL_2503580, EPI_ISL_2503581, EPI_ISL_2503584, EPI_ISL_2503585, EPI_ISL_2503586, EPI_ISL_2503590, EPI_ISL_2503591, EPI_ISL_2503592, EPI_ISL_2503593, EPI_ISL_2503595, EPI_ISL_2503596, EPI_ISL_2503597, EPI_ISL_2503599, EPI_ISL_2503603, EPI_ISL_2503604, EPI_ISL_2503606, EPI_ISL_2503608, EPI_ISL_2503609, EPI_ISL_2503614, EPI_ISL_2503615, EPI_ISL_2503616, EPI_ISL_2503618, EPI_ISL_2503619, EPI_ISL_2503620, EPI_ISL_2503622, EPI_ISL_2503623, EPI_ISL_2503624, EPI_ISL_2503627, EPI_ISL_2503628, EPI_ISL_2503629, EPI_ISL_2503632, EPI_ISL_2503635, EPI_ISL_2503636, EPI_ISL_2503637, EPI_ISL_2503638, EPI_ISL_2503640, EPI_ISL_2503641, EPI_ISL_2503653, EPI_ISL_2503654, EPI_ISL_2503656, EPI_ISL_2503657, EPI_ISL_2503658, EPI_ISL_2503659, EPI_ISL_2503662, EPI_ISL_2503664, EPI_ISL_2503665, EPI_ISL_2503667, EPI_ISL_2503672, EPI_ISL_2503673, EPI_ISL_2503674, EPI_ISL_2503675, EPI_ISL_2503677, EPI_ISL_2503684, EPI_ISL_2503685, EPI_ISL_2503686, EPI_ISL_2503688, EPI_ISL_2503692, EPI_ISL_2503694, EPI_ISL_2503701, EPI_ISL_2503704, EPI_ISL_2503708, EPI_ISL_2503709, EPI_ISL_2503710, EPI_ISL_2503711, EPI_ISL_2503712, EPI_ISL_2503715, EPI_ISL_2503716, EPI_ISL_2503717, EPI_ISL_2503719, EPI_ISL_2503720, EPI_ISL_2503721, EPI_ISL_2503722, EPI_ISL_2503723, EPI_ISL_2503724, EPI_ISL_2503725, EPI_ISL_2503732, EPI_ISL_2503733, EPI_ISL_2503735, EPI_ISL_2503739, EPI_ISL_2503741, EPI_ISL_2503742, EPI_ISL_2503743, EPI_ISL_2503744, EPI_ISL_2503747, EPI_ISL_2503749, EPI_ISL_2503750, EPI_ISL_2503751, EPI_ISL_2503752, EPI_ISL_2503753, EPI_ISL_2503754, EPI_ISL_2503757, EPI_ISL_2503758, EPI_ISL_2503759, EPI_ISL_2503762, EPI_ISL_2503763, EPI_ISL_2503764, EPI_ISL_2503765, EPI_ISL_2503767, EPI_ISL_2503769, EPI_ISL_2503771, EPI_ISL_2503775, EPI_ISL_2503776, EPI_ISL_2503779, EPI_ISL_2503790, EPI_ISL_2503791, EPI_ISL_2503792, EPI_ISL_2503793, EPI_ISL_2503794, EPI_ISL_2503797, EPI_ISL_2503800, EPI_ISL_2503802, EPI_ISL_2503803, EPI_ISL_2503805, EPI_ISL_2503806, EPI_ISL_2503809, EPI_ISL_2503810, EPI_ISL_2503811, EPI_ISL_2503812, EPI_ISL_2503816, EPI_ISL_2503818, EPI_ISL_2503819, EPI_ISL_2503822, EPI_ISL_2548371, EPI_ISL_2548372, EPI_ISL_2548373, EPI_ISL_2548375, EPI_ISL_2548376, EPI_ISL_2548377, EPI_ISL_2548378, EPI_ISL_2548386, EPI_ISL_2548399, EPI_ISL_2548573, EPI_ISL_2548574, EPI_ISL_2548575, EPI_ISL_2548576, EPI_ISL_2548578, EPI_ISL_2548579, EPI_ISL_2548580, EPI_ISL_2548581, EPI_ISL_2548593, EPI_ISL_2548594, EPI_ISL_2548595, EPI_ISL_2548596, EPI_ISL_2548597, EPI_ISL_2548598, EPI_ISL_2548600, EPI_ISL_2548601, EPI_ISL_2548602, EPI_ISL_2548603, EPI_ISL_2548604, EPI_ISL_2548605, EPI_ISL_2548606, EPI_ISL_2548607, EPI_ISL_2548612, EPI_ISL_2548613, EPI_ISL_2548614, EPI_ISL_2548615, EPI_ISL_2548616, EPI_ISL_2548618, EPI_ISL_2548619, EPI_ISL_2548620, EPI_ISL_2548622, EPI_ISL_2548623, EPI_ISL_2548627, EPI_ISL_2548630, EPI_ISL_2548631, EPI_ISL_2548632, EPI_ISL_2548633, EPI_ISL_2548635, EPI_ISL_2548636, EPI_ISL_2548641, EPI_ISL_2548646, EPI_ISL_2548647, EPI_ISL_2548649, EPI_ISL_2548650, EPI_ISL_2548653, EPI_ISL_2548655, EPI_ISL_2548656, EPI_ISL_2548657, EPI_ISL_2548659, EPI_ISL_2548661, EPI_ISL_2548664, EPI_ISL_2548670, EPI_ISL_2548671, EPI_ISL_2548672, EPI_ISL_2548673, EPI_ISL_2548675, EPI_ISL_2548676, EPI_ISL_2548677, EPI_ISL_2548678, EPI_ISL_2548684, EPI_ISL_2548690, EPI_ISL_2548694, EPI_ISL_2548698, EPI_ISL_2548699, EPI_ISL_2548700, EPI_ISL_2548701, EPI_ISL_2548704, EPI_ISL_2548706, EPI_ISL_2548708, EPI_ISL_2548710, EPI_ISL_2548711, EPI_ISL_2548717, EPI_ISL_2548724, EPI_ISL_2548725, EPI_ISL_2548728, EPI_ISL_2548731, EPI_ISL_2548732, EPI_ISL_2548733, EPI_ISL_2548736, EPI_ISL_2548737, EPI_ISL_2548739, EPI_ISL_2548740, EPI_ISL_2548741, EPI_ISL_2548742, EPI_ISL_2548743, EPI_ISL_2548744, EPI_ISL_2548745, EPI_ISL_2548747, EPI_ISL_2548748, EPI_ISL_2548749, EPI_ISL_2548751, EPI_ISL_2548752, EPI_ISL_2548753, EPI_ISL_2548758, EPI_ISL_2548759, EPI_ISL_2548760, EPI_ISL_2548761, EPI_ISL_2548762, EPI_ISL_2548764, EPI_ISL_2548765, EPI_ISL_2548766, EPI_ISL_2548768, EPI_ISL_2548769, EPI_ISL_2548773, EPI_ISL_2548774, EPI_ISL_2548776, EPI_ISL_2548779, EPI_ISL_2548780, EPI_ISL_2548782, EPI_ISL_2548786, EPI_ISL_2548789, EPI_ISL_2548790, EPI_ISL_2548791, EPI_ISL_2548792, EPI_ISL_2548794, EPI_ISL_2548800, EPI_ISL_2548801, EPI_ISL_2548802, EPI_ISL_2548803, EPI_ISL_2548806, EPI_ISL_2548808, EPI_ISL_2548809, EPI_ISL_2548810, EPI_ISL_2548811, EPI_ISL_2548813, EPI_ISL_2548815, EPI_ISL_2548817, EPI_ISL_2548818, EPI_ISL_2548819, EPI_ISL_2548820, EPI_ISL_2548821, EPI_ISL_2548827, EPI_ISL_2548828, EPI_ISL_2548832, EPI_ISL_2548833, EPI_ISL_2548834, EPI_ISL_2548836, EPI_ISL_2548837, EPI_ISL_2548838, EPI_ISL_2548839, EPI_ISL_2548840, EPI_ISL_2548841, EPI_ISL_2548842, EPI_ISL_2548843, EPI_ISL_2548857, EPI_ISL_2548909, EPI_ISL_2548910, EPI_ISL_2548911, EPI_ISL_2548912, EPI_ISL_2548913, EPI_ISL_2548914, EPI_ISL_2548915, EPI_ISL_2548918, EPI_ISL_2548919, EPI_ISL_2548920, EPI_ISL_2548921, EPI_ISL_2548922, EPI_ISL_2548923, EPI_ISL_2548924, EPI_ISL_2548925, EPI_ISL_2548926, EPI_ISL_2548927, EPI_ISL_2548928, EPI_ISL_2548929, EPI_ISL_2548930, EPI_ISL_2548931, EPI_ISL_2548932, EPI_ISL_2548933, EPI_ISL_2548934, EPI_ISL_2548935, EPI_ISL_2548936, EPI_ISL_2548937, EPI_ISL_2548938, EPI_ISL_2548939, EPI_ISL_2548940, EPI_ISL_2548942, EPI_ISL_2548943, EPI_ISL_2548944, EPI_ISL_2548945, EPI_ISL_2548946, EPI_ISL_2548947, EPI_ISL_2548948, EPI_ISL_2548949, EPI_ISL_2548950, EPI_ISL_2548951, EPI_ISL_2548952, EPI_ISL_2548953, EPI_ISL_2548954, EPI_ISL_2548955, EPI_ISL_2548956, EPI_ISL_2548957, EPI_ISL_2548958, EPI_ISL_2548959, EPI_ISL_2548960, EPI_ISL_2548961, EPI_ISL_2548962, EPI_ISL_2548963, EPI_ISL_2548964, EPI_ISL_2548965, EPI_ISL_2548966, EPI_ISL_2548967, EPI_ISL_2548969, EPI_ISL_2548970, EPI_ISL_2548971, EPI_ISL_2548972, EPI_ISL_2548973, EPI_ISL_2548974, EPI_ISL_2548975, EPI_ISL_2548976, EPI_ISL_2548977, EPI_ISL_2548978, EPI_ISL_2548979, EPI_ISL_2548980, EPI_ISL_2548981, EPI_ISL_2548982, EPI_ISL_2548983, EPI_ISL_2548984, EPI_ISL_2548985, EPI_ISL_2548986, EPI_ISL_2548989, EPI_ISL_2548990, EPI_ISL_2548991, EPI_ISL_2548992, EPI_ISL_2548993, EPI_ISL_2548994, EPI_ISL_2548995, EPI_ISL_2548996, EPI_ISL_2548997, EPI_ISL_2548998, EPI_ISL_2548999, EPI_ISL_2549000, EPI_ISL_2549003, EPI_ISL_2549004, EPI_ISL_2549005, EPI_ISL_2549006, EPI_ISL_2549007, EPI_ISL_2549008, EPI_ISL_2549012, EPI_ISL_2549013, EPI_ISL_2549014, EPI_ISL_2549015, EPI_ISL_2549016, EPI_ISL_2549018, EPI_ISL_2549019, EPI_ISL_2549021, EPI_ISL_2549022, EPI_ISL_2549023, EPI_ISL_2549024, EPI_ISL_2549025, EPI_ISL_2549026, EPI_ISL_2549027, EPI_ISL_2549028, EPI_ISL_2549029, EPI_ISL_2549030, EPI_ISL_2549031, EPI_ISL_2549032, EPI_ISL_2549033, EPI_ISL_2549034, EPI_ISL_2549036, EPI_ISL_2549037, EPI_ISL_2549038, EPI_ISL_2549039, EPI_ISL_2549041, EPI_ISL_2549042, EPI_ISL_2549048, EPI_ISL_2549054, EPI_ISL_2549069, EPI_ISL_2675936, EPI_ISL_2675937, EPI_ISL_2675938, EPI_ISL_2675939, EPI_ISL_2675940, EPI_ISL_2675941, EPI_ISL_2675942, EPI_ISL_2675943, EPI_ISL_2675946, EPI_ISL_2675947, EPI_ISL_2675948, EPI_ISL_2675949, EPI_ISL_2675950, EPI_ISL_2675951, EPI_ISL_2675952, EPI_ISL_2675953, EPI_ISL_2675954, EPI_ISL_2675955, EPI_ISL_2675956, EPI_ISL_2675957, EPI_ISL_2675958, EPI_ISL_2675959, EPI_ISL_2675960, EPI_ISL_2675961, EPI_ISL_2675962, EPI_ISL_2675963, EPI_ISL_2675964, EPI_ISL_2675965, EPI_ISL_2675966, EPI_ISL_2675967, EPI_ISL_2675968, EPI_ISL_2675970, EPI_ISL_2675971, EPI_ISL_2675980, EPI_ISL_2675981, EPI_ISL_2675983, EPI_ISL_2675985, EPI_ISL_2675987, EPI_ISL_2675988, EPI_ISL_2675990, EPI_ISL_2675991, EPI_ISL_2675992, EPI_ISL_2675993, EPI_ISL_2675994, EPI_ISL_2675995, EPI_ISL_2675996, EPI_ISL_2675997, EPI_ISL_2675998, EPI_ISL_2675999, EPI_ISL_2676000, EPI_ISL_2676001, EPI_ISL_2676002, EPI_ISL_2676003, EPI_ISL_2676004, EPI_ISL_2676005, EPI_ISL_2676006, EPI_ISL_2676007, EPI_ISL_2676008, EPI_ISL_2676009, EPI_ISL_2676011, EPI_ISL_2676012, EPI_ISL_2676014, EPI_ISL_2676015, EPI_ISL_2676016, EPI_ISL_2676017, EPI_ISL_2676018, EPI_ISL_2676019, EPI_ISL_2676020, EPI_ISL_2676021, EPI_ISL_2676022, EPI_ISL_2676023, EPI_ISL_2676024, EPI_ISL_2676027, EPI_ISL_2676028, EPI_ISL_2676029, EPI_ISL_2676030, EPI_ISL_2676032, EPI_ISL_2676033, EPI_ISL_2676034, EPI_ISL_2676035, EPI_ISL_2676036, EPI_ISL_2676037, EPI_ISL_2676038, EPI_ISL_2676039, EPI_ISL_2676040, EPI_ISL_2676041, EPI_ISL_2676042, EPI_ISL_2676044, EPI_ISL_2676046, EPI_ISL_2676047, EPI_ISL_2676048, EPI_ISL_2676049, EPI_ISL_2676050, EPI_ISL_2676051, EPI_ISL_2676052, EPI_ISL_2676053, EPI_ISL_2676054, EPI_ISL_2676055, EPI_ISL_2676056, EPI_ISL_2676057, EPI_ISL_2676058, EPI_ISL_2676059, EPI_ISL_2676060, EPI_ISL_2676061, EPI_ISL_2676062, EPI_ISL_2676063, EPI_ISL_2676064, EPI_ISL_2676065, EPI_ISL_2676066, EPI_ISL_2676067, EPI_ISL_2676068, EPI_ISL_2676069, EPI_ISL_2676070, EPI_ISL_2676071, EPI_ISL_2676072, EPI_ISL_2676073, EPI_ISL_2676074, EPI_ISL_2676075, EPI_ISL_2676076, EPI_ISL_2676078, EPI_ISL_2676079, EPI_ISL_2676080, EPI_ISL_2676081, EPI_ISL_2676082, EPI_ISL_2676083, EPI_ISL_2676084, EPI_ISL_2676087, EPI_ISL_2676088, EPI_ISL_2676141, EPI_ISL_2676188, EPI_ISL_2676223, EPI_ISL_2676263			
see above	INSACOG-WB	National Institute of Biomedical Genomics – INSACOG	Ajay Chakraborti; Arindam Maitra; Bhaswati Bandyopadhyay; Nidhan Kumar Biswas; Saumitra Das; Sreedhar Chinnaswamy; Tamal Ghosh
EPI_ISL_826804, EPI_ISL_826818, EPI_ISL_826829	INSPI-CRN DE INFLUENZA Y OTROS VIRUS RESPIRATORIOS	Instituto de Salud Publica de Chile	Alfredo Bruno; Andres Castillo; Barbara Parra; Domenica de Mora; Gisselle Barra; Jaime Lagos; Javier Tognarelli; Jimmy Garcez; Jorge Fernandez; Loredana Arata; Manuel Gonzalez; Martiza Olmedo; Michelle Paez; Patricia Bustos; Rodrigo Fasce; Solon Narvaez
EPI_ISL_2488738	INSPI-CRN DE INFLUENZA Y OTROS VIRUS RESPIRATORIOS	NIC-INSPI	Alfredo Bruno; Domenica de Mora.; Jimmy Garcés; Johanna Laines; Lizbeth Patiño; Manuel Gonzalez; Maritza Olmedo; Michelle Páez
EPI_ISL_940769	INSPI-CRN de Influenza y otros virus respiratorios	INSPI-Centro de Investigación Multidisciplinaria de la DTIDI	Alberto Orlando.; Alfredo Bruno; Andrés Carrazco-Montalvo; Doménica de Mora; Leandro Patiño; Manuel González; Maritza Olmedo; Mary Regato-Arrata; Melissa Zambrano; Orson Mestanza

EPI_ISL_1701391	INT Fondazione Pascale	INT Fondazione Pascale	INT Fondazione Pascale
EPI_ISL_2604874, EPI_ISL_2604876, EPI_ISL_2604878, EPI_ISL_2604879	INTERLAB	Omics Sciences Laboratory	Darlyn Amaya; Derly Andrade Molina; Gabriel Morey León; Juan Carlos Fernández Cadena; Rubén Armas González
EPI_ISL_2577027	IRCCS San Gallicano Dermatological Institute	IRCCS Regina Elena National Cancer Institute	Aldo Morrone; Eleonora Sperandio; Fabrizio Ensoli; Frauke Goeman; Fulvia Pimpinelli; Gennaro Ciliberto; Giovanni Blandino; Giulia Orlandi; Maurizio Fanciulli; Valentina Ricca
EPI_ISL_1434565	Ibaraki Prefectural Institute of Public Health	Ibaraki Prefectural Institute of Public Health	Keiko Goto; Kentaro Itokawa; Makoto Kuroda; Masanori Hashino; Rina Tanaka; Tsuyoshi Sekizuka
EPI_ISL_2322996	Imeda Hospital	Imeda Hospital	Dagmar Obbels; Hanne Valgaeren; Johan Frans
EPI_ISL_2790553	Imelda Ziekenhuis	Imelda Ziekenhuis	Dagmar Obbels; Hanne Valgaeren; Johan Frans
EPI_ISL_481166	Immunogenomics lab, Institute of Life Sciences, Bhubaneswar	Immunogenomics lab, Institute of Life Sciences, Bhubaneswar	Ajay Parida; Amol Ratnakar Suryawanshi; Ankita Datey; Arup Ghosh; Atimukta Jha; Auromira Khuntia; Bharati Singh; DBT's PAN-INDIA 1000 SARS-CoV2 RNA genome sequencing consortium; Deepika Singh; Dileep Vasudevan; Ghulam Hussain Syed; Manasi Priyadarshini; Neha Singh; Orissa COVID-19 Study Group; P. Sushree Shyamli; Punit Prasad; Rajeeb Swain; Rupesh Dash; Shanti Senapati; Soma Chattopadhyay; Sunil Raghav; Swati Madhulika; Tsheten Sheroa; Tushar K. Beuria; Viplov K. Biswas
EPI_ISL_1662447, EPI_ISL_1662451, EPI_ISL_1663552, EPI_ISL_1663553, EPI_ISL_1663554, EPI_ISL_1663555, EPI_ISL_1663557, EPI_ISL_1663558, EPI_ISL_1663561, EPI_ISL_1663562, EPI_ISL_1663563, EPI_ISL_1663564			
see above	Immunogenomics lab, Institute of Life Sciences, Bhubaneswar	Institute of Life Sciences - INSACOG	Ajay Parida; Amol M. Kanampalliwar; Arup Ghosh; Atimukta Jha; INSACOG Consortium; Punit Prasad; Rajeeb Swain; Rupesh Dash; Safal Walia; Shifu Aggarwal; Sunil K. Raghav
EPI_ISL_1018089, EPI_ISL_2001078, EPI_ISL_2001090, EPI_ISL_2508378, EPI_ISL_2508380	Immunology, Noguchi Memorial Institute for Medical Research	Immunology, Noguchi Memorial Institute for Medical Research	Adu, B.; Adusei-Poku; Agbenyo; Agbodzi, B.; Ampofo; Appiah-Kubi, J.; Asare; Attiku; Bonney; Egyir, B.; J.K.; K.M.; K.O.; Kumordjie, S.; M.A.; Mohktar, Q.; Odoom; Opoku Frempong, H.; Oteng, F.; Owusu-Nyantakyi, C.; P.O.; S.B.; Uche; W.K.; Yeboah, C.
EPI_ISL_1517410, EPI_ISL_1517433	Inciensa, Instituto Costarricense de Investigación y Enseñanza en Nutrición y Salud	Inciensa, Instituto Costarricense de Investigación y Enseñanza en Nutrición y Salud	Barboza-Arguedas E & Centeno-Miranda M; Cristian Peréz-Corrales; Valeria Peralta-Barquero & César Cerdas Quesada
EPI_ISL_413522	Indian Council of Medical Research - National Institute of Virology	National Influenza Center, Indian Council of Medical Research - National Institute of Virology	Choudhary ML; Potdar V; Shete-Aich A; Yadav PD
EPI_ISL_2364567	Infectious Diseases and Tropical Medicine Research Center, Isfahan University of Medical Sciences, Isfahan, Iran	Genetics Research Center, University of Social Welfare and Rehabilitation Sciences	Behrooz Ataei; Hamed Fakhim; Hossein Najmabadi.; Kimia Kahrizi; Marzieh Mohseni; Zohreh Fattahi
EPI_ISL_2466400	Inst. for Med. Virology, University Hospital Frankfurt, Goethe University Frankfurt	Inst. for Med. Virology, University Hospital Frankfurt, Goethe University Frankfurt	Goetsch, U.; Gottschalk; H.F.; Pallas, C.; R. and Ciesek, S.; Rabenau; Toptan, T.; Widera, M.; Wilhelm, A.
EPI_ISL_1013421	Institut Pasteur de Guadeloupe	National Reference Center for Viruses of Respiratory Infections, Institut Pasteur, Paris	Angela Brisebarre; Camille Capel; Etienne Simon-Lorière; Marion Barbet; Maud Vanpeene; Méline Bizard; Sylvie Behillil; Sylvie van der Werf; Talarmin Antoine; Vincent Enouf
EPI_ISL_999032	Institut Pasteur de Guinée	Institut Pasteur de Dakar	Dia Ndongo; Diagne Moussa Moïse; Diallo Amadou; Diop Mamadou; Faye Ousmane; Grayo Solene; Loucoubar Cheikh; Mbengue Safietou Sankhe; Ndiaye Ndack; Sall Amadou Alpha; Tordo Noel
EPI_ISL_613421	Institut Pasteur de la Guadeloupe	Institut Pasteur de la Guadeloupe	Angela Brisebarre; Antoine Talarmin; Camille Capel; Etienne Simon-Lorière; Marion Barbet; Maud Vanpeene; Méline Bizard; Stéphanie Guyomard; Sylvie Behillil; Sylvie van der Werf; Sébastien Breurec; Vincent Enouf
EPI_ISL_437992	Institut für Virologie am Department für Hygiene, Mikrobiologie und Public Health	Bergthaler laboratory, CeMM Research Center for Molecular Medicine of the Austrian Academy of Sciences	Alexander Lercher; Alexandra Popa; Andreas Bergthaler; Benedikt Agerer; Christoph Bock; Dorothee von Laer; Elisabeth Puchhammer-Stoeckl; Guenter Weiss; Henrique Colaco; Jakob-Wendelin Genger; Jan Laine; Judith Aberle; Lukas Endler; Manfred Nairz; Mark Smyth; Martin Senekowitsch; Michael Schuster; Stephan Aberle; Thomas Penz; Wegene Borena
EPI_ISL_1498151, EPI_ISL_1657076, EPI_ISL_1915113	Institute for Developing Science and Health Initiatives (ideSHi)	Institute for Developing Science and Health Initiatives (ideSHi)	Fidausi Qadri; Hassan Afrad; Sadia Rahman; Tahmina Shirin
EPI_ISL_853861	Institute for Laboratory Diagnostics and Microbiology, Klinikum Klagenfurt am Worthersee	Bergthaler laboratory, CeMM Research Center for Molecular Medicine of the Austrian Academy of Sciences	Alexander Lercher; Alexandra Popa; Andreas Bergthaler; Anna Schedl; Benedikt Agerer; Christoph Bock; Jakob-Wendelin Genger; Jan Laine; Lukas Endler; Martin Senekowitsch; Michael Schuster; Thomas Penz
EPI_ISL_1591285, EPI_ISL_1904548	Institute for Public Health of Šibenik-Knin County	Croatian Institute of Public Health	Irena Tabain; Ivana Ferenčak
EPI_ISL_572333	Institute for Virology, University Hospital Duesseldorf, Medical Faculty, Heinrich-Heine-University Duesseldorf	Institute for Virology, University Hospital Duesseldorf, Medical Faculty, Heinrich-Heine-University Duesseldorf	; Alexander Killer; Andreas Walker; Annemarie Mohring; Anselm Kunstein; Ansgar Schulz; Björn Jensen; Caroline Klindt; Edwin Bölke; Gerald Antoch; Heiner Schaal; Jennifer Neubert; Johannes Bode; Johannes C. Fischer; Jörg Timm; Lisa Müller; Maximilian Damagnez; Nadine Lübke; Ortwin Adams; Philipp Albrecht; Philipp Ostermann; Saskia Elben; Tina Senff; Tom Lüdde; Torsten Feldt; Verena Keitel
EPI_ISL_2774071	Institute of Epidemiology, Disease Control and Research (IEDCR)	IEDCR-ideSHi-icddr,b	Firdausi Qadri; Hassan Afrad; Manjur Hossain Khan; Sadia Rahman; Tahmina Shirin
EPI_ISL_1582390, EPI_ISL_1582394, EPI_ISL_1582396, EPI_ISL_1915436, EPI_ISL_1915437, EPI_ISL_1915439, EPI_ISL_1938475, EPI_ISL_1938476, EPI_ISL_1938477			
see above	Institute of Epidemiology, Disease Control and Research (IEDCR)	Institute for Developing Science and Health Initiatives (ideSHi)	Fidausi Qadri; Hassan Afrad; Sadia Rahman; Tahmina Shirin
EPI_ISL_2105565	Institute of Epidemiology, Disease Control and Research (IEDCR)	ideSHi-IEDCR-icddr,b	Firdausi Qadri; Hassan Afrad; Manjur Hossain Khan; Sadia Rahman; Tahmina Shirin
EPI_ISL_2341665, EPI_ISL_2341682, EPI_ISL_2341695, EPI_ISL_2341698, EPI_ISL_2341699, EPI_ISL_2341701, EPI_ISL_2341704, EPI_ISL_2341706, EPI_ISL_2341712, EPI_ISL_2341763, EPI_ISL_2341779, EPI_ISL_2341783, EPI_ISL_2341785, EPI_ISL_2341842, EPI_ISL_2341847, EPI_ISL_2341924, EPI_ISL_2341925, EPI_ISL_2341926, EPI_ISL_2341941, EPI_ISL_2341942, EPI_ISL_2341998, EPI_ISL_2342017, EPI_ISL_2342021, EPI_ISL_2342028, EPI_ISL_2342062, EPI_ISL_2342072, EPI_ISL_2342074, EPI_ISL_2342104, EPI_ISL_2342115, EPI_ISL_2342120, EPI_ISL_2342260, EPI_ISL_2342284, EPI_ISL_2342359, EPI_ISL_2342360, EPI_ISL_2342361, EPI_ISL_2342362, EPI_ISL_2342363, EPI_ISL_2342604, EPI_ISL_2342639, EPI_ISL_2342645, EPI_ISL_2342652, EPI_ISL_2342666, EPI_ISL_2342671, EPI_ISL_2342695, EPI_ISL_2342846, EPI_ISL_2342980			
see above	Institute of Life Sciences, Bhubaneswar	Institute of Life Sciences - INSACOG	Ajay Parida; Amol M. Kanampalliwar; Arup Ghosh; Atimukta Jha; INSACOG Consortium; Omprakash Shiriwas; Punit Prasad; Rajeeb Swain; Rupesh Dash; Safal Walia; Sana Fatma; Shifu Aggarwal; Sunil K. Raghav
EPI_ISL_422563	Institute of Microbiology Universidad San Francisco de Quito	Institute of Microbiology Universidad San Francisco de Quito	Alejandra Ramones; Belen Prado-Vivar; Bernardo Gutierrez; Edison Ligña; Francisco Mora; Franklin Espinoza; Gabriel Trueba; Jorge Reyes; Juan Gaviria; Juan Jose Guadalupe; Michelle Grunauer; Patricio Rojas-Silva; Paul Cardenas; Sully Marquez; Veronica Barragan
EPI_ISL_477016, EPI_ISL_486849, EPI_ISL_516652, EPI_ISL_539792, EPI_ISL_697798, EPI_ISL_697799, EPI_ISL_728202, EPI_ISL_824284, EPI_ISL_1738805, EPI_ISL_1805659, EPI_ISL_2004104, EPI_ISL_2100427, EPI_ISL_2134872, EPI_ISL_2228102, EPI_ISL_2228104, EPI_ISL_2488768, EPI_ISL_2492350, EPI_ISL_2603826, EPI_ISL_2603828			
see above	Institute of Microbiology, Universidad San Francisco de Quito	Institute of Microbiology, Universidad San Francisco de Quito	Belén Prado-Vivar; Bernardo Darquea; Bernardo Gutiérrez; Carla Torres; Carlos Mena; Christian Zambrano; David Zuñiga; Diana Zambrano; Edison Chavez; Edy Quizhpe; Fernada Zurita; Fernanda Zurita; Fernando Serrano; Fredy Loor; Gabriel Trueba; Guzmán Bernabéu Lorenzo; Jonathan Araujo; Jorge Luis Velez; Juan Carlos Zuñiga; Juan José Guadalupe; Juan Zuñiga; Katherine Ojeda; Leofrein Vera; Ligia Briceño; Lofrein Vera; Luis Flores; Manuel Jibaja; Mayra Beltrán; Melissa Ortega; Michelle Grunauer; Milton Tobar; Monica Becerra-Wong; Nabih Dahik; Nelson Montalvan; Oscar Mena; Paola Dalgo; Patricio Reyes; Patricio Rojas-Silva; Paúl Cárdenas; Prado-Vivar; Raiza Briceño; Rommel Guevara; Sully Márquez; Tania Guayasamin; Tanya Guayasamin; Verónica Barragán; Yomara Napa
EPI_ISL_1443652	Institute of Microbiology, Universidad San Francisco de Quito	Omics Sciences Laboratory	; Andrea Cunguan; Belén Prado-Vivar; Bernardo Gutiérrez; Darlyn Amaya; Dayron Brossad; Derly Andrade Molina; Emily Sulay Saltos Montalvo; Fernanda Zurita; Gabriel Morey León; Gabriel Trueba; Juan Carlos Fernández Cadena; Juan José Guadalupe; Katheryn Sacheri Viteri; Michelle Grunauer; Monica Becerra-Wong; Nabih Dahik; Patricio Rojas-Silva; Paula Juliana Gavilanes Jarrín; Paúl Cárdenas; Rubén Armas González; Sully Márquez; Verónica Barragán
EPI_ISL_1745707, EPI_ISL_2346356, EPI_ISL_2346376	Institute of Molecular and Translational Medicine / Laboratory of Experimental Medicine, Faculty of Medicine and Dentistry, Palacky University and University Hospital Olomouc	Institute of Molecular and Translational Medicine / Laboratory of Experimental Medicine, Faculty of Medicine and Dentistry, Palacky University	Barbora Blumová; Hana Jaworek; Marián Hajdúch; Rastislav Slavkovský; Tomáš Pospíšil; Vladimíra Koudeláková
EPI_ISL_2420421	Institute of Public Health Varaždin County	Croatian Institute of Public Health	Irena Tabain; Ivana Ferenčak
EPI_ISL_2625681	Institute of Public Health of Zagreb County	Croatian Institute of Public Health	Irena Tabain; Ivana Ferenčak
EPI_ISL_852660, EPI_ISL_1805024	Institute of Virology, Medical Center, University of Freiburg, Freiburg, Germany	Institute of Virology, Clinial Virus Genomics, Medical Center, University of Freiburg, Freiburg, Germany	Hajo Grundmann; Jonas Fuchs; Lena Jaki; Lisa Kern; Marcus Panning; Sandra Reuter
EPI_ISL_2344266, EPI_ISL_2344303, EPI_ISL_2344426	Instituto Butantan	Instituto de Medicina Tropical de Sao Paulo	Brazil-UK Centre for Arbovirus Discovery Diagnosis Genomics and Epidemiology (CADDE) Genomic Network - Instituto de Medicina Tropical
EPI_ISL_2348777	Instituto Nacional De Investigación En Salud Pública-Crn De Influenza Y Otros Virus Respiratorios	NIC-Instituto Nacional de Investigación en Salud Pública	Alfredo Bruno; Domenica de Mora.; Jimmy Garcés; Johanna Laines; Lizbeth Patiño; Manuel Gonzalez; Maritza Olmedo; Michelle Páez
EPI_ISL_491948	Instituto Nacional de Investigación en Salud Pública - INSPI	INSPI - Charité	Alberto Orlando; Alexandra Usiña; Alfredo Bruno Caicedo; Andres Moreira-Soto; Anna-Lena Sander; Denisses Portugal; Domenica de Mora Coloma; Jan Felix Drexler; Juan Carlos Zeballos; Manuel Gonzalez; Maritza Olmedo; Nina Krause; Silvia Salgado
EPI_ISL_2492739, EPI_ISL_2492777, EPI_ISL_2492788, EPI_ISL_2492791, EPI_ISL_2492814, EPI_ISL_2492870, EPI_ISL_2492899, EPI_ISL_2493084, EPI_ISL_2493086			
see above	Instituto Nacional de Investigação em Saúde	CERI, Centre for Epidemic Response and Innvoation, Stellenbosch University and KRISP, KZN Research Innovation and Sequencing Platform, UKZN.	Afonso P; David K; Emmanuel SJ; Freitas RH; Giandhari J; Inglês L; Lutucuta S; Miranda J; Morais J; Mufinda M; Naidoo Y; Neto Z; Paulo A Carralero RR Paixão JP; Pereira A; Pillay S; Tegally H; Wilkinson E; de Oliveira T
EPI_ISL_1545310, EPI_ISL_1545372, EPI_ISL_2494743, EPI_ISL_2494862, EPI_ISL_2494867, EPI_ISL_2494868, EPI_ISL_2494944, EPI_ISL_2609491, EPI_ISL_2609532			
see above	Instituto Nacional de Investigação em Saúde	KRISP, KZN Research Innovation and Sequencing Platform	Afonso P; David K; Emmanuel SJ; Freitas RH; Giandhari J; Inglês L; Lutucuta S; Miranda J; Morais J; Mufinda M; Naidoo Y; Neto Z; Paulo A Carralero RR Paixão JP; Pereira A; Pillay S; Tegally H; Wilkinson E; de Oliveira T
EPI_ISL_2500966	Instituto Nacional de Salud	Instituto Nacional de Salud- Dirección de Investigación en Salud Pública	Carlos Franco-Muñoz; Carmen Osorio; Diana Malo; Diego A. Álvarez-Díaz; Diego Andrés Prada; Gerardo Santamaría; Hector Alejandro Ruiz-Moreno; Jhonnatan Reales-González; Jorge Rivera; Juan Camilo Martinez; Julian Naizaque; Katherine Laiton-Donato; Lisseth Pardo; Magdalena Wiesner; Marcela Mercado-Reyes; Maria T. Herrera-Sepúlveda; Marta Lopez Blanco; Martha Lucia Ospina Martinez; Paola Rojas; Sergio Gomez; Sheryll Corchuelo; Ángela Alarcon Cruz
EPI_ISL_456126	Instituto Nacional de Salud - Unidad de Secuenciación y Análisis Genómico	Instituto Nacional de Salud, Universidad Cooperativa de Colombia, Instituto Alexander von Humboldt, Imperial College-London, London School of Hygiene & Tropical Medicine	Astrid C. Flórez; Carlos Franco-Muñoz; Christian Julian Villabona-Arenas; Diana Marcela Walteros-Acero; Diego A. Álvarez-Díaz; Erika Ospitia; Gloria Puerto; Jose A. Usme-Ciro; Juliana Barbosa; Katherine Laiton-Donato; Liz Villabona-Arenas; Luz Dary Rodriguez; Mailyn A.Gonzalez; Marcela Mercado-Reyes.; Martha Lucia Ospina Martinez; Nicolas D. Franco-Sierra; Sergio Gomez-Rangel; Sussy Echeverria; Zulma M. Cucunubá
EPI_ISL_942006	Instituto Nacional de Salud, Bogotá, Colombia	Centro de Investigaciones en Microbiología y Biotecnología-UR (CIMBIUR), Facultad de Ciencias Naturales, Universidad del Rosario, Bogotá, Colombia Instituto Nacional de Salud, Bogotá, Colombia Icahn School of Medicine at Mount Sinai, New York, USA	Adriana van de Guchte; Alberto Paniz-Mondolfi; Ana S. Gonzalez-Reiche; Carolina Flórez; Carolina Hernández; Emilia Mia Sordillo; Hala Alejel Alshammary; Harm van Bakel; Jayeeta Dutta; Juan David Ramírez; Luz Helena Patiño; Marina Muñoz; Matthew M. Hernandez; Nathalia Ballesteros; Sergio Gomez; Viviana Simon; Zenab Khan
EPI_ISL_526964, EPI_ISL_653745,	Instituto Nacional de Salud, Bogotá, Colombia	Instituto Nacional de Salud, Bogotá, Colombia	Astrid C. Flórez; Carlos Andrés Durán; Carlos Franco-Muñoz; Carolina Ferro; Christian Julian Villabona-Arenas; Diana Marcela Walteros-Acero; Diego A. Álvarez-Díaz; Diego Andrés Prada; Edmilson F. de Oliveira-Filho; Felix Betzler; Franklin Prieto; Jeadran Malagón-Rojas; Jonathan Reales; Jose A. Usme-Ciro; Katherine Laiton-Donato; Liz Villabona-Arenas; Magdalena Weisner; Marcela Mercado-Reyes; Martha Lucia Ospina Martinez; Mauricio Pacheco-Montealegre; Sheryl

EPI_ISL_653757, EPI_ISL_653762, EPI_ISL_739663			Corchuelo; Sussy Echeverria; Wendy K. Jo; Zulma M. Cucunubá
EPI_ISL_1424061	Instituto Nacional de Salud- Dirección de Investigación en Salud Pública	Instituto Nacional de Salud- Dirección de Investigación en Salud Pública	Carlos Franco-Muñoz; Diego A. Álvarez-Díaz; Diego Andrés Prada; Gerardo Santamaría; Jhonnatan Reales-González; Julian Naizaque; Katherine Laiton-Donato; Magdalena Wiesner; Marcela Mercado-Reyes.; Maria T. Herrera-Sepúlveda; Martha Lucia Ospina Martínez; Mauricio Pacheco; Sheryll Corchuelo
EPI_ISL_956286	Instituto Nacional de Salud- Dirección de Redes de Laboratorios de Salud Pública	Instituto Nacional de Salud- Dirección de Investigación en Salud Pública	Carlos Franco-Muñoz; Diego A. Álvarez-Díaz; Diego Andrés Prada; Gerardo Santamaría; Hector Alejandro Ruiz-Moreno; Jhonnatan Reales-González; Julian Naizaque; Katherine Laiton-Donato; Magdalena Wiesner; Marcela Mercado-Reyes; Maria T. Herrera-Sepúlveda; Martha Lucia Ospina Martinez; Mauricio Pacheco-Montealegre; Sheryll Corchuelo
EPI_ISL_2601034	Instituto de Medicina Tropical & Salud Global, Universidad Iberoamericana (UNIBE)	Instituto de Medicina Tropical & Salud Global, Universidad Iberoamericana (UNIBE)	& Robert Paulino-Ramirez; Alejandro Vallejo Degaudenzi; Victor Virgilio Calderon
EPI_ISL_2505257	Islab, Etelä-Savon aluelaboratorio	Expert Microbiology, National Institute for Health and Welfare	Carita Savolainen-Kopra; Erika Lindh; Haider al-Hello; Jani Halkilahti; Kirsi Liitsola; Niina Ikonen; Olli Vapalahti; Pekka Ellonen; Phuoc Truong; Päivi Laurila; Ravi Kant; Sari Hannula; Soile Blomqvist; Teemu Smura
EPI_ISL_2506825	Islab, Pohjois-Savon aluelaboratorio	Expert Microbiology, National Institute for Health and Welfare	Carita Savolainen-Kopra; Erika Lindh; Haider al-Hello; Jani Halkilahti; Kirsi Liitsola; Niina Ikonen; Olli Vapalahti; Pekka Ellonen; Phuoc Truong; Päivi Laurila; Ravi Kant; Sari Hannula; Soile Blomqvist; Teemu Smura
EPI_ISL_956393	Isolation - Virology Unit, Institut Pasteur du Cambodge; Sequencing - US National Institute of Allergy and Infectious Diseases Cambodia, US Naval Medical Research Unit -2, Cambodia National Institute for Public Health	Virology Unit, Institut Pasteur du Cambodge	Chau Darapheak; Chin Savuth; Erik A Karlsson; Jennifer Bohl; Jessica Manning; Jose A Garcia-Rivera; Kraing Sidonn; Ly Sovann; Sophana Chea; Sreyngim Lay; Veasna Duong; Vireak Heang; Yi Sengdoeurn
EPI_ISL_1240650	Israel Central Virology Laboratory	Israel National Consortium for SARS-CoV-2 sequencing	Assaf Rokney; Dana Bar-Ilan; David A. Zeevi; Efrat Dahan Bucris; Efrat Glick-Saar; Efrat Rorman; Ella Mendelson; Ephraim Fass; Eva Nachum; Gal Zizelski Valenci; Gideon Rechavi; Israel Nissan; Joseph Jaffe; Maya Davidovich Cohen; Michal Mandelboim; Mor Rubinstein; Neta Zuckerman; Omer Murik; Omri Nayshool; Oran Erster; Orna Mor; Tzvia Mann
EPI_ISL_514284, EPI_ISL_776808	Israel Central Virology laboratory	Israel Central Virology laboratory	Efrat Dahan Bucris; Ella Mendelson; Michal Mandelboim; Neta Zuckerman; Oran Erster; Orna Mor
EPI_ISL_804097, EPI_ISL_1240651, EPI_ISL_1763441, EPI_ISL_2084505, EPI_ISL_2084619, EPI_ISL_2085056, EPI_ISL_2085745, EPI_ISL_2182030, EPI_ISL_2182923, EPI_ISL_2183026, EPI_ISL_2183029, EPI_ISL_2183057, EPI_ISL_2183059, EPI_ISL_2183060, EPI_ISL_2183062, EPI_ISL_2183112, EPI_ISL_2183114, EPI_ISL_2183116, EPI_ISL_2183117, EPI_ISL_2183141, EPI_ISL_2183341, EPI_ISL_2183420, EPI_ISL_2183422, EPI_ISL_2183424, EPI_ISL_2183427, EPI_ISL_2183429, EPI_ISL_2183431, EPI_ISL_2183433, EPI_ISL_2183622, EPI_ISL_2183640, EPI_ISL_2183660, EPI_ISL_2183769, EPI_ISL_2183842, EPI_ISL_2183852, EPI_ISL_2636189			
see above	Israel Central Virology laboratory	Israel National Consortium for SARS-CoV-2 sequencing	Assaf Rokney; Dana Bar-Ilan; David A. Zeevi; Efrat Dahan Bucris; Efrat Glick-Saar; Efrat Rorman; Ella Mendelson; Ephraim Fass; Eva Nachum; Gal Zizelski Valenci; Gideon Rechavi; Israel Nissan; Joseph Jaffe; Maya Davidovich Cohen; Michal Mandelboim; Miranda Geva; Mor Rubinstein; Neta Zuckerman; Netanel Abu; Omer Murik; Omri Nayshool; Oran Erster; Orna Mor; Tzvia Mann
EPI_ISL_778812, EPI_ISL_778814, EPI_ISL_837481, EPI_ISL_1630068, EPI_ISL_1715012, EPI_ISL_1759633, EPI_ISL_1793154, EPI_ISL_2020594, EPI_ISL_2282004, EPI_ISL_2356961, EPI_ISL_2550948, EPI_ISL_2550992, EPI_ISL_2551142			
see above	Istituto Zooprofilattico Sperimentale del Mezzogiorno	TIGEM	Andrea Ballabio; Anna Manfredi; Antonio Grimaldi; Antonio Grimaldi Patrizia Annunziata Francesco Panariello Biancamaria Pierri Claudia Tiberio Teresa Giuliano Valentina Bouche Chiara Colantuono Maria Concetta Cuomo Denise Di Concilio Lucio Di Filippo Anna Manfredi Marcello Salvi Antonio Limone Luigi Atripaldi Pellegrino Cerino Andrea Ballabio Davide Cacchiarelli; Antonio Limone; Biancamaria Pierri; Chiara Colantuono; Davide Cacchiarelli.; Denise Di Concilio; Francesco Panariello; Lucio Di Filippo; Marcello Salvi; Maria Concetta Cuomo; Patrizia Annunziata; Pellegrino Cerino; Valentina Bouche
EPI_ISL_2641780	Istituto Zooprofilattico Sperimentale del Mezzogiorno	Telethon Institute of Genetics and Medicine (TIGEM)	Antonio Grimaldi Patrizia Annunziata Francesco Panariello Biancamaria Pierri Claudia Tiberio Teresa Giuliano Valentina Bouche Chiara Colantuono Maria Concetta Cuomo Denise Di Concilio Lucio Di Filippo Anna Manfredi Marcello Salvi Antonio Limone Luigi Atripaldi Pellegrino Cerino Andrea Ballabio Davide Cacchiarelli
EPI_ISL_2341995, EPI_ISL_2342032, EPI_ISL_2342037, EPI_ISL_2342053, EPI_ISL_2342071, EPI_ISL_2342081, EPI_ISL_2342084, EPI_ISL_2342090, EPI_ISL_2342094, EPI_ISL_2342101, EPI_ISL_2342106, EPI_ISL_2342116, EPI_ISL_2342119, EPI_ISL_2342124, EPI_ISL_2342126, EPI_ISL_2342355			
see above	Itki Aarogyashala, Ranchi	Institute of Life Sciences - INSACOG	Ajay Parida; Amol M. Kanampalliwar; Arup Ghosh; Atimukta Jha; INSACOG Consortium; Omprakash Shiriwas; Punit Prasad; Rajeeb Swain; Rupesh Dash; Safal Walia; Sana Fatma; Shifu Aggarwal; Sunil K. Raghav
EPI_ISL_2723817, EPI_ISL_2723818, EPI_ISL_2723819, EPI_ISL_2723820	JIPMER Asia / India / Puducherry / Puducherry	inStem NCBS – INSACOG	Uma Ramakrishnan Dasaradhi Palakodeti Aswin SaiNarain
EPI_ISL_1939929, EPI_ISL_1939930, EPI_ISL_1939931, EPI_ISL_1939932, EPI_ISL_1939934, EPI_ISL_1939936, EPI_ISL_1939939, EPI_ISL_1939940, EPI_ISL_1939947, EPI_ISL_1939950, EPI_ISL_1939951, EPI_ISL_1939952, EPI_ISL_1939955, EPI_ISL_1939956, EPI_ISL_1939957, EPI_ISL_1939958, EPI_ISL_1939959, EPI_ISL_1939961, EPI_ISL_1939962, EPI_ISL_1939963, EPI_ISL_1939964, EPI_ISL_1939966, EPI_ISL_2272789, EPI_ISL_2272790, EPI_ISL_2272791, EPI_ISL_2272794, EPI_ISL_2272795, EPI_ISL_2272796, EPI_ISL_2272798, EPI_ISL_2272799, EPI_ISL_2272800, EPI_ISL_2272801, EPI_ISL_2272802, EPI_ISL_2272803, EPI_ISL_2272812, EPI_ISL_2272814, EPI_ISL_2272815, EPI_ISL_2272819, EPI_ISL_2272820, EPI_ISL_2272821, EPI_ISL_2272824, EPI_ISL_2272827, EPI_ISL_2272828, EPI_ISL_2272829, EPI_ISL_2272830, EPI_ISL_2272831, EPI_ISL_2272832, EPI_ISL_2272833, EPI_ISL_2272834, EPI_ISL_2272835, EPI_ISL_2272836, EPI_ISL_2272838, EPI_ISL_2272839, EPI_ISL_2272840, EPI_ISL_2272841, EPI_ISL_2272843, EPI_ISL_2272844, EPI_ISL_2272845, EPI_ISL_2272846, EPI_ISL_2272847, EPI_ISL_2272848, EPI_ISL_2272849, EPI_ISL_2272850, EPI_ISL_2272851, EPI_ISL_2272852, EPI_ISL_2272853, EPI_ISL_2272854, EPI_ISL_2272856, EPI_ISL_2272857, EPI_ISL_2272858, EPI_ISL_2272859, EPI_ISL_2272860, EPI_ISL_2272861, EPI_ISL_2272862, EPI_ISL_2272863, EPI_ISL_2272864, EPI_ISL_2272865, EPI_ISL_2272866, EPI_ISL_2272867, EPI_ISL_2272868, EPI_ISL_2272869, EPI_ISL_2272870, EPI_ISL_2272871, EPI_ISL_2272872, EPI_ISL_2272874, EPI_ISL_2272876, EPI_ISL_2272877, EPI_ISL_2272878, EPI_ISL_2272879, EPI_ISL_2272881, EPI_ISL_2272882, EPI_ISL_2272886, EPI_ISL_2272887, EPI_ISL_2272888, EPI_ISL_2272893, EPI_ISL_2272895, EPI_ISL_2272897, EPI_ISL_2272898, EPI_ISL_2272899, EPI_ISL_2272900, EPI_ISL_2272901, EPI_ISL_2272903, EPI_ISL_2272904, EPI_ISL_2272905, EPI_ISL_2272908, EPI_ISL_2272910, EPI_ISL_2272911, EPI_ISL_2272912, EPI_ISL_2272913, EPI_ISL_2272914, EPI_ISL_2272916, EPI_ISL_2272917, EPI_ISL_2272918, EPI_ISL_2272919, EPI_ISL_2272920, EPI_ISL_2272922, EPI_ISL_2272923, EPI_ISL_2272924, EPI_ISL_2272926, EPI_ISL_2272928, EPI_ISL_2272932, EPI_ISL_2272933, EPI_ISL_2272936, EPI_ISL_2272937			
see above	JIPMER Puducherry	inStem NCBS - INSACOG	Uma Ramakrishnan Dasaradhi Palakodeti Aswin SaiNarain
EPI_ISL_2450333	JJR NAGAR UPHC	INSACOG-KA, NIMHANS	Ananthapadmanabha Kotambail; Anita S Desai; Anson Kunjumon George; Chetan G K; Chitra Pattabiraman; Darshan Sreenivas; Gautham Arunachal Udupi; V Ravi
EPI_ISL_1753676, EPI_ISL_1922480, EPI_ISL_2007204, EPI_ISL_2422370, EPI_ISL_2422371, EPI_ISL_2422399, EPI_ISL_2462998, EPI_ISL_2491217, EPI_ISL_2601980, EPI_ISL_2646318, EPI_ISL_2675263, EPI_ISL_2774142, EPI_ISL_2774143			
see above	Jessa	Jessa	Berden et al. on behalf of Jessa_cmdLab; Berden et al. on behalf of the Jessa_cmdLab; Cruys et al. on behalf of the Jessa_cmdLab; Marijke Raymaekers et al. on behalf of the Jessa_cmdLab; Severine Berden et al. on behalf of the Jessa_cmdLab
EPI_ISL_1939969, EPI_ISL_1939970	KAMAKSHIPALYA	inStem NCBS - INSACOG	Uma Ramakrishnan Dasaradhi Palakodeti Aswin SaiNarain
EPI_ISL_1818617, EPI_ISL_1818618, EPI_ISL_2545211	KEMPEGOWDA INTERNATIONAL AIRPORT	INSACOG-KA, NIMHANS	Ananthapadmanabha Kotambail; Anita S Desai; Anson Kunjumon George; Chetan G K; Chitra Pattabiraman; Darshan Sreenivas; Gautham Arunachal Udupi; Pramada Prasad; V Ravi
EPI_ISL_1610621, EPI_ISL_1610632, EPI_ISL_2602970	KEMRI Center for Biotechnology Research and Development	KEMRI-Wellcome Trust Research Programme,Kilifi	; Githinji G.; Matoke D.; Mburu M.W.; Mohamed K.S.; Onyango C.; Thiongo K.; de Laurent Z.
EPI_ISL_2602898	KEMRI-CGHR	KEMRI-Wellcome Trust Research Programme,Kilifi	; Githinji G.; Matoke D.; Mburu M.W.; Mohamed K.S.; Onyango C.; de Laurent Z.
EPI_ISL_1440102, EPI_ISL_1440118, EPI_ISL_1440125, EPI_ISL_1610516, EPI_ISL_2602464, EPI_ISL_2602470, EPI_ISL_2602494, EPI_ISL_2602500, EPI_ISL_2602574, EPI_ISL_2602576, EPI_ISL_2602583, EPI_ISL_2602588, EPI_ISL_2602618, EPI_ISL_2602705, EPI_ISL_2602762, EPI_ISL_2602763, EPI_ISL_2603006, EPI_ISL_2603015, EPI_ISL_2603023, EPI_ISL_2603038, EPI_ISL_2603039, EPI_ISL_2603040, EPI_ISL_2603043, EPI_ISL_2603044, EPI_ISL_2603049, EPI_ISL_2603051, EPI_ISL_2603052, EPI_ISL_2603053, EPI_ISL_2603056, EPI_ISL_2603057, EPI_ISL_2603059			
see above	KEMRI-Wellcome Trust Research Programme,Kilifi	KEMRI-Wellcome Trust Research Programme,Kilifi	; Githinji G.; Matoke D.; Mburu M.W.; Mohamed K.S.; Onyango C.; Thiongo K.; de Laurent Z.; deLaurent Z.
EPI_ISL_855537, EPI_ISL_1039227	KEMRI-Wellcome Trust Research Programme/KEMRI-CGMR-C Kilifi	KEMRI-Wellcome Trust Research Programme/KEMRI-CGMR-C Kilifi	Githinji et al
EPI_ISL_1091296, EPI_ISL_1093306, EPI_ISL_1312211, EPI_ISL_2424439, EPI_ISL_2424440, EPI_ISL_2424441, EPI_ISL_2424442, EPI_ISL_2424443, EPI_ISL_2424444, EPI_ISL_2425097, EPI_ISL_2425122, EPI_ISL_2425254, EPI_ISL_2425255, EPI_ISL_2425256, EPI_ISL_2425257, EPI_ISL_2425258, EPI_ISL_2425316, EPI_ISL_2425317, EPI_ISL_2425320, EPI_ISL_2425321, EPI_ISL_2425322, EPI_ISL_2725597, EPI_ISL_2725611, EPI_ISL_2725612, EPI_ISL_2725613, EPI_ISL_2725614, EPI_ISL_2725615, EPI_ISL_2725616, EPI_ISL_2725620, EPI_ISL_2725621, EPI_ISL_2725622, EPI_ISL_2725638, EPI_ISL_2725659, EPI_ISL_2725661, EPI_ISL_2725662, EPI_ISL_2725663, EPI_ISL_2725664, EPI_ISL_2725665, EPI_ISL_2725700, EPI_ISL_2725709, EPI_ISL_2726311, EPI_ISL_2726312, EPI_ISL_2726502, EPI_ISL_2726503, EPI_ISL_2726504, EPI_ISL_2726516, EPI_ISL_2726517, EPI_ISL_2726518, EPI_ISL_2726519, EPI_ISL_2726520, EPI_ISL_2726521, EPI_ISL_2726522, EPI_ISL_2726523, EPI_ISL_2726524, EPI_ISL_2726525, EPI_ISL_2726526, EPI_ISL_2726527			
see above	KU Leuven, Rega Institute, Clinical and Epidemiological Virology	KU Leuven, Rega Institute, Clinical and Epidemiological Virology	Bert Vanmechelen; Joan Marti-Carerras; Piet Maes; Tony Wawina-Bokalanga
EPI_ISL_2544687	Kanagawa Prefectural Institute of Public Health	Kanagawa Prefectural Institute of Public Health	Itsumi Toyokura; Junichi Sakuragi; Kentaro Itokawa; Makiko Kondo; Makoto Kuroda; Masanori Hashino; Rieko Suzuki; Rina Tanaka; Sumi Watanabe; Takako Sano; Takayuki Hishiki; Tomohiko Takasaki; Tsuyoshi Sekizuka
EPI_ISL_2233091, EPI_ISL_2233092, EPI_ISL_2233093, EPI_ISL_2233096, EPI_ISL_2233097, EPI_ISL_2233098, EPI_ISL_2233101, EPI_ISL_2233103, EPI_ISL_2233105			
see above	Kantor Kesehatan Pelabuhan Kelas II Cilacap	National Institute of Health Research and Development	Arie Ardiansyah Nugraha; Hana Apsari Pawestri; Hartanti Dian Ikawati; Kartika Dewi Puspa; Krisna Pangesti; Nelly Puspandari; Subangkit; Triyani Soekarso; Vivi Setiawaty
EPI_ISL_2365354	Kariminejad-Najmabadi Pathology & Genetics Center, Tehran, Iran	Genetics Research Center, University of Social Welfare and Rehabilitation Sciences	Hossein Najmabadi.; Kimia Kahrizi; Mahdieh Koshki; Maryam Azad; Marzieh Mohseni; Siavash Ghaderi; Zohreh Fattahi
EPI_ISL_833500	Klinik Apotek Dein, Jakarta, Indonesia	Biosafety Level-3 Laboratory, Indonesian Institute of Sciences (LIPI)	Ade Andriani; Ahmad Fathoni; Andri Wardiana; Anggia Prasetyoputri; Anik Budhi Dharmayanthi; Isa Nuryana; Ratih Asmana Ningrum; Syam Budi Iryanto
EPI_ISL_2232706	Klinikum Wels-Grieskirchen	Bergthaler laboratory, CeMM Research Center for Molecular Medicine of the Austrian Academy of Sciences	Andreas Bergthaler; Anna Schedl; Bekir Erguner; Benedikt Agerer; Christoph Bock; Fabian Amman; Jan Laine; Lukas Endler; Maelle Le Moing; Martin Senekowitsch; Michael Schuster; Petr Triska; Thomas Penz
EPI_ISL_2397179, EPI_ISL_2397185, EPI_ISL_2397200, EPI_ISL_2612043	Klinisch Laboratorium ZNA	Klinisch Laboratorium ZNA	Verstrepen et al.
EPI_ISL_1398369	Kuningan Public Health	West Java Health Laboratory; School of Life Sciences and Technology, Institut Teknologi Bandung	Azzania Fibriani; Cut Nur Cinthia Alamanda; Ema Rahmawati; Isak Solihin; Kamila Tania; Karimatu Khoirunnisa; Miftahul Faridl; Rifky Waluyajati Rachman; Rini Robiani; Ryan Bayusantika Ristandi
EPI_ISL_747239	Kuningan Publlic Health Office	West Java Health Laboratory; School of Life Sciences and Technology, Institut Teknologi Bandung	Azzania Fibriani; Cut Nur Cinthia Alamanda; Ema Rahmawati; Isak Solihin; Karimatu Khoirunnisa; Miftahul Faridl; Rifky Waluyajati Rachman; Rini Robiani; Ryan Bayusantika Ristandi
EPI_ISL_2502740	LABORATORIO CLINICO LABIN	Inciensa, Instituto Costarricense de InvestigaciOn y Enseñanza en NutriciOn y Salud	Adriana Godínez; Claudio Soto-Garita; Estela Cordero; Francisco Duarte; Hebleen Porras; Jose Luis Vargas; Joselyn Prado & Pei Chan; Mariela Gutierrez; Melany CalderOn
EPI_ISL_1379427	LABORATORIO CLINICO LABIN	Inciensa, Instituto Costarricense de Investigación y Enseñanza en Nutrición y Salud	Adriana Godínez; Claudio Soto-Garita; Estela Cordero; Francisco Duarte; Hebleen Porras; Melany Calderón & Pei Ling Chan Ma
EPI_ISL_1821062	LABORATORIO CLINICO SYNLAB	Instituto Nacional de Salud- Dirección de Investigación en Salud Pública	Carlos Franco-Muñoz; Carmen Osorio; Diana Malo; Diego A. Álvarez-Díaz; Diego Andrés Prada; Gerardo Santamaría; Hector Alejandro Ruiz-Moreno; Jhonnatan Reales-González; Jorge Rivera; Juan Camilo Martinez; Julian Naizaque; Katherine Laiton-Donato; Lisseth Pardo; Magdalena Wiesner; Marcela Mercado-Reyes; Maria T. Herrera-Sepúlveda; Marta Lopez Blanco; Martha Lucia Ospina Martinez; Paola Rojas; Sergio Gomez; Sheryll Corchuelo; Ángela Alarcon Cruz
EPI_ISL_1220048	LABORATORIO CLÍNICO FUNDACIÓN HOSPITAL SAN PEDRO	Instituto Nacional de Salud- Dirección de Investigación en Salud Pública	Carlos Franco-Muñoz; Diego A. Álvarez-Díaz; Diego Andrés Prada; Gerardo Santamaría; Hector Alejandro Ruiz-Moreno; Jhonnatan Reales-González; Julian Naizaque; Katherine Laiton-Donato; Magdalena Wiesner; Marcela Mercado-Reyes.; Maria T. Herrera-Sepúlveda; Martha Lucia Ospina Martínez; Sheryll Corchuelo
EPI_ISL_1213335	LAFEM/UESC	Bioinformatics Laboratory / LNCC	Alessandra P Lamarca; Alexandra L Gerber; Ana Paula Melo Mariano; Ana Paula de C Guimarães; Ana Tereza R Vasconcelos; Angela Maria Guimarães Santos; Bianca Mendes Maciel; Danielle Angst Secco; Eduardo Sérgio Soares Sousa; Eloiza Helena Campana; Francisco Paulo Freire Neto; George Rego Albuquerque; Kátia Castanho Scortecci; Lucymara Fassarella Agnez Lima; Luiz G P de Almeida; Luís Cristóvão Porto; Otavio J. Brustolini; Paulo Ricardo Nascimento; Ronaldo

			da Silva Francisco Jr; Sandra Rocha Gadelha; Selma Maria Bezerra Jeronimo; Vinícius Pietta Perez
EPI_ISL_1904967, EPI_ISL_1904969, EPI_ISL_1904971, EPI_ISL_1904972, EPI_ISL_1904973, EPI_ISL_1904974	LAM ORIADE ABBAYE ST MARTIN D'HERES	CNR Virus des Infections Respiratoires - France SUD	Antonin Bal; Bruno Lina; Gregory Destras; Gwendolyne Burfin; Hadrien Regue; Laurence Josset; Martine Valette; Quentin Semanas
EPI_ISL_2342608	LBRKM Government Medical College, Jagdalpur	Institute of Life Sciences - INSACOG	Ajay Parida; Amol M. Kanampalliwar; Arup Ghosh; Atimukta Jha; INSACOG Consortium; Omprakash Shiriwas; Punit Prasad; Rajeeb Swain; Rupesh Dash; Safal Walia; Sana Fatma; Shifu Aggarwal; Sunil K. Raghav
EPI_ISL_1091786	LDSP	Universidad Nacional de Colombia - Laboratorio Genómico One Health	Andres F. Cardona-Rios; Carlos Franco-Muñoz; Daniel O. Maldonado-Perez; Diego A. Álvarez-Díaz; Idabely Betancur Ortiz; Jorge E. Osorio; Juan P. Hernandez-Ortiz; Karl A Ciuoderis; Laura Silvana Perez; Marcela Mercado-Reyes; Maria Angélica Maya; Sandra Ines Cano
EPI_ISL_1805646	LDSP CALDAS	Instituto Nacional de Salud- Dirección de Investigación en Salud Pública	Carlos Franco-Muñoz; Carmen Osorio; Diana Malo; Diego A. Álvarez-Díaz; Diego Andrés Prada; Gerardo Santamaría; Hector Alejandro Ruiz-Moreno; Jhonnatan Reales-González; Jorge Rivera; Juan Camilo Martinez; Julian Naizaque; Katherine Laiton-Donato; Lisseth Pardo; Magdalena Wiesner; Marcela Mercado-Reyes; Maria T. Herrera-Sepúlveda; Marta Lopez Blanco; Martha Lucia Ospina Martinez; Paola Rojas; Sergio Gomez; Sheryll Corchuelo; Ángela Alarcon Cruz
EPI_ISL_2362590	LDSP CUNDINAMARCA	Instituto Nacional de Salud- Dirección de Investigación en Salud Pública	Carlos Franco-Muñoz; Carmen Osorio; Diana Malo; Diego A. Álvarez-Díaz; Diego Andrés Prada; Gerardo Santamaría; Hector Alejandro Ruiz-Moreno; Jhonnatan Reales-González; Jorge Rivera; Juan Camilo Martinez; Julian Naizaque; Katherine Laiton-Donato; Lisseth Pardo; Magdalena Wiesner; Marcela Mercado-Reyes; Maria T. Herrera-Sepúlveda; Marta Lopez Blanco; Martha Lucia Ospina Martinez; Paola Rojas; Sergio Gomez; Sheryll Corchuelo; Ángela Alarcon Cruz
EPI_ISL_1805632	LDSP TOLIMA	Instituto Nacional de Salud- Dirección de Investigación en Salud Pública	Carlos Franco-Muñoz; Carmen Osorio; Diana Malo; Diego A. Álvarez-Díaz; Diego Andrés Prada; Gerardo Santamaría; Hector Alejandro Ruiz-Moreno; Jhonnatan Reales-González; Jorge Rivera; Juan Camilo Martinez; Julian Naizaque; Katherine Laiton-Donato; Lisseth Pardo; Magdalena Wiesner; Marcela Mercado-Reyes; Maria T. Herrera-Sepúlveda; Marta Lopez Blanco; Martha Lucia Ospina Martinez; Paola Rojas; Sergio Gomez; Sheryll Corchuelo; Ángela Alarcon Cruz
EPI_ISL_2016831, EPI_ISL_2545652, EPI_ISL_2545663, EPI_ISL_2628050, EPI_ISL_2628051, EPI_ISL_2628053, EPI_ISL_2628056, EPI_ISL_2628058, EPI_ISL_2628059, EPI_ISL_2628060, EPI_ISL_2714890, EPI_ISL_2714893, EPI_ISL_2714896, EPI_ISL_2714897, EPI_ISL_2714898, EPI_ISL_2714899, EPI_ISL_2714900, EPI_ISL_2714901			
see above	LHUB-ULB	Labo Klinische Biologie, UZA	Basil Britto Xavier; Christine Lammens; Herman Goossens; Ines Verbesselt; Jasmine Coppens; Kathleen Holemans; Marie Le Mercier; Veerle Matheeussen
EPI_ISL_1219272, EPI_ISL_2141780	LIC	Latvian Biomedical Research and Study Centre	Davids Fridmanis; Diana Dusacka; Elina Dimina; Guntars Zarins; Ivars Silamikelis; Janis Klovins; Janis Pjalkovskis; Jurijs Perevoscikovs; Kaspars Megnis; Laila Silamikele; Lauma Freimane; Laura Ansone; Liga Birzniece; Monta Ustinova; Nikita Zrelovs; Reinis Zeltmatis; Uga Dumpis; Una Krumina; Vita Rovite
EPI_ISL_1969244, EPI_ISL_1969245	Lab RSUP DR Mohammad Hoesin Palembang	National Institute of Health Research and Development	Arie Ardiansyah Nugraha; Hana Apsari Pawestri; Hartanti Dian Ikawati; Kartika Dewi Puspa; Krisna Pangesti; Nelly Puspandari; Subangkit; Triyani Soekarso; Vivi Setiawaty
EPI_ISL_1827216, EPI_ISL_2192265, EPI_ISL_2192404, EPI_ISL_2192405, EPI_ISL_2192420, EPI_ISL_2192463, EPI_ISL_2362819, EPI_ISL_2362860, EPI_ISL_2362935, EPI_ISL_2362951, EPI_ISL_2362955, EPI_ISL_2604978, EPI_ISL_2605014, EPI_ISL_2605046, EPI_ISL_2605052, EPI_ISL_2605092, EPI_ISL_2605104, EPI_ISL_2605118, EPI_ISL_2605240, EPI_ISL_2605297, EPI_ISL_2605356, EPI_ISL_2605437, EPI_ISL_2605502, EPI_ISL_2605509, EPI_ISL_2605553, EPI_ISL_2605579, EPI_ISL_2605596, EPI_ISL_2605622, EPI_ISL_2605647			
see above	Lab voor klinische biologie	Lab voor klinische biologie	Bruno Verhasselt; Hannelore Hamerlinck; Marija Janevska
EPI_ISL_2121121	LabKom - Labor Augsburg MVZ GmbH	Robert Koch Institute	
EPI_ISL_2121307	LabKom - Labor an der Salzbrucke MVZ GmbH	Robert Koch Institute	
EPI_ISL_2313482	LabKom - Labor an der Salzbrücke MVZ GmbH	Robert Koch Institute	
EPI_ISL_2233094	Labkesda Prov Kal Tim	National Institute of Health Research and Development	Arie Ardiansyah Nugraha; Hana Apsari Pawestri; Hartanti Dian Ikawati; Kartika Dewi Puspa; Krisna Pangesti; Nelly Puspandari; Subangkit; Triyani Soekarso; Vivi Setiawaty
EPI_ISL_1263004, EPI_ISL_1517463, EPI_ISL_1788929, EPI_ISL_1915155, EPI_ISL_1915156, EPI_ISL_2029687, EPI_ISL_2029713, EPI_ISL_2029727, EPI_ISL_2029740, EPI_ISL_2029751, EPI_ISL_2029877, EPI_ISL_2029916, EPI_ISL_2532185, EPI_ISL_2612577, EPI_ISL_2612650			
see above	Labo Analyses Med	National Reference Center for Viruses of Respiratory Infections, Institut Pasteur, Paris	Angela Brisebarre; Anne Holstein; Camille Capel; Christophe Malabat; Corinne Maufrais; Elodie Etienne; Emmanuelle Permal; Etienne Simon-Lorière; Fabienne Artur; Frédéric Lemoine; GréGoire Potiron; Hue; Jean Boyer; Judith Zerah; Louise Lefrançois; Marion Barbet; Maud Vanpeene; Méline Bizard; Pierre Lechat; Pierre Netzer; Said-Delattre OphéLie; Sophie Chalmin; Sylvie Behillil; Sylvie Van der Werf; Sylvie van der Werf; Victoire Baillet; Vincent Enouf
EPI_ISL_2422226	Labo Klinische Biologie, UZA	Labo Klinische Biologie, UZA	Basil Britto Xavier; Christine Lammens; Herman Goossens; Jasmine Coppens; Marie Le Mercier; Veerle Matheeussen
EPI_ISL_2348574, EPI_ISL_2348575, EPI_ISL_2348576, EPI_ISL_2348577, EPI_ISL_2348578, EPI_ISL_2348579, EPI_ISL_2348580, EPI_ISL_2348581, EPI_ISL_2348582, EPI_ISL_2348583, EPI_ISL_2348584, EPI_ISL_2348585, EPI_ISL_2348587, EPI_ISL_2348588, EPI_ISL_2348589, EPI_ISL_2348590, EPI_ISL_2348591, EPI_ISL_2348592, EPI_ISL_2382824			
see above	Labo Luc Olivier	GIGA Medical Genomics	Bouchra Boujemla; Cécile Meex; Keith Durkin; Maria Artesi; Marie-Pierre Hayette; Nathalie Renotte; Pierrette Melin; Raphaël Boreux; Sébastien Bontems; Vincent Bours
EPI_ISL_1843504, EPI_ISL_2109111, EPI_ISL_2121556, EPI_ISL_2121887, EPI_ISL_2121903, EPI_ISL_2125917, EPI_ISL_2260092, EPI_ISL_2260146, EPI_ISL_2260299, EPI_ISL_2260301, EPI_ISL_2260332			
see above	Labor 28 MVZ GmbH	Robert Koch Institute	
EPI_ISL_2386808, EPI_ISL_2386838, EPI_ISL_2386840, EPI_ISL_2389915, EPI_ISL_2389924, EPI_ISL_2389927, EPI_ISL_2389938, EPI_ISL_2389939, EPI_ISL_2389949			
see above	Labor Becker & Kollegen (Standort München)	Robert Koch Institute	
EPI_ISL_1921855	Labor Berlin Charite Vivantes GmbH / Institut für Virologie	Charité Universitätsmedizin Berlin, Institut für Virologie/Labor Berlin	Barbara Mühlemann; Christian Drosten; Christine Stephan; Peter Menzel; Rolf Schwarzer; Terry Jones; Victor M Corman
EPI_ISL_1840801, EPI_ISL_1840814, EPI_ISL_1840828, EPI_ISL_1886104, EPI_ISL_2107830, EPI_ISL_2107835, EPI_ISL_2107839, EPI_ISL_2694826			
see above	Labor Berlin Charité Vivantes GmbH / Institut für Virologie	Charité Universitätsmedizin Berlin, Institut für Virologie/Labor Berlin	Barbara Mühlemann; Christian Drosten; Christine Stephan; Peter Menzel; Rolf Schwarzer; Terry Jones; Victor M Corman
EPI_ISL_1904299	Labor Doz DDr Stefan Mustafa	AGES IMED Vienna	Alexander Indra; Elisabeth Walter; Johanna Schmitt; Sara Meschini; Stefan Mustafa; Theodhora Ziu
EPI_ISL_2384664	Labor Dr. Fenner und Kollegen	Heinrich Pette Institute, Leibniz Institute for Experimental Virology	Adam Grundhoff; Alexis Robitaille; Johannes Knobloch; Martin Aepfelbacher; Nicole Fischer; Thomas Günther
EPI_ISL_1566762, EPI_ISL_1851395, EPI_ISL_1851414, EPI_ISL_2632175	Labor Dr. Heidrich & Kollegen MVZ GmbH Hamburg	Robert Koch Institute	
EPI_ISL_2125466	Labor Dr. Schumacher MVZ	Robert Koch Institute	
EPI_ISL_2125129	Labor Dr. Spranger	Robert Koch Institute	
EPI_ISL_2111026	Labor Dr. Wisplinghoff - Kaln	Robert Koch Institute	
EPI_ISL_1848757	Labor MÃ¶nchengladbach MVZ Dr. Stein + Kollegen GbR	Robert Koch Institute	
EPI_ISL_2111454	Labor ZOTZ|KLIMAS; MVZ Dusseldorf-Centrum	Robert Koch Institute	
EPI_ISL_2111142	Laborarztpraxis Dres. med. Walther Weindel & Kollegen	Robert Koch Institute	
EPI_ISL_428961, EPI_ISL_434504	Laboratoire National de Sante, Microbiology, Virology	Laboratoire National de Sante, Microbiology, Epidemiology and Microbial Genomics	Anke Wienecke-Baldacchino; Ardashel Latsuzbaia; Catherine Ragimbeau; Guillaume Fournier; Jessica Tapp; Joel Mossong; Tamir Abdelrahman; Trung Nguyen Nguyen
EPI_ISL_2157368, EPI_ISL_2492506	Laboratoire National de Santé Publique – LNSP (HAITI - LNSP)	Laboratory of Respiratory Viruses and Measles, Oswaldo Cruz Institute, FIOCRUZ	Alice Sampaio Rocha; Ana Carolina Mendonca; Ana Carolina Paixao; Elisa Cavalcante Pereira; Fernando Motta; Ito Journel; Jaques Boncy; Luciana Appolinario; Marilda Siqueira on behalf of the Fiocruz COVID-19 Genomic Surveillance Network; Paola Resende; Patrick Delly; Renata Serrano Lopes; Taina Venas
EPI_ISL_2137841, EPI_ISL_2137843	Laboratoire Professeur Daniel GAHOUMA (LPDG)	Centre de Recherches Médicales de Lambaréné (CERMEL)	Bertrand Lell and Ayola Akim Adegnika; Davy Leger Mouangala; Georgelin Nguema Ondo; Guy Stéphane Padzys; Gédéon Prince Manouana; Jean Bernard Lekana-Douki; Joël-Fleury Djoba Siawaya; Ludovic Mewono; Moustapha Nzamba Maloum; Noé Patrick Mbondoukwe; Rodrigue Bikangui; Rodrigue Mintsa Nguema; Sam O'neilla Oye Bingono; Samira Zoa Assoumou; Sandrine Zeh Nfor; Srinivas reddy Pallerla; Steffen Borrmann; Thirumalaisamy P. Velavan
EPI_ISL_1760554, EPI_ISL_1760555	Laboratoire Professeur Daniel GAHOUMA (LPDG)	Centre de recherches médicales de Lambaréné (CERMEL)	Ayola A. Adegnika; Ayong Moure; Bertrand Lell; Bénédicte Ndeboko; Emilio Skarwan; Georgelin Nguema Ondo; Gédéon P. Manouana; Haruka Abe; Jiro Yasuda; Joel Fleury Djoba Siawaya; Rodrigue Bikangui; Rotimi Myrabelle Avome Houechenou; Samira Zoa-Assoumou; Yuri Ushijima
EPI_ISL_660451, EPI_ISL_660466, EPI_ISL_660472, EPI_ISL_660473, EPI_ISL_660479, EPI_ISL_660492, EPI_ISL_660512, EPI_ISL_660514, EPI_ISL_660523, EPI_ISL_2142720, EPI_ISL_2142730			
see above	Laboratoire de Microbiologie CHU Souro Sanou	Centre Muraz	Abdoul-Salam Ouedraogo; Amariane Koné; Armel Poda; Arsène Zongo; Essia Belarbi; Fabian Leendertz; Grit Schubert; Halidou Tinto; Lassana Sangaré; Soumeya Ouangraoua; Thérèse Kagoné; Yacouba Sawadogo; Zekiba Tarnagda
EPI_ISL_890130, EPI_ISL_1366066, EPI_ISL_2499845	Laboratoire de santé publique du Québec	Laboratoire de santé publique du Québec	Guillaume Bourque; Ioannis Ragoussis; Jesse Shapiro; Mark Lathrop and Michel Roger on behalf of the CoVSeQ research group; Mark Lathrop and Michel Roger on behalf of the CoVSeQ research group (http://covseq.ca/researchgroup); Sandrine Moreira
EPI_ISL_476825, EPI_ISL_476831, EPI_ISL_476833	Laboratoire des Fièvres Hémorragiques Virales du Benin	Charité-Universitätsmedizin Berlin	Anges; Drexler; Jan Felix; Moreira-Soto Andres; Sander Anna-Lena; Yadouleton
EPI_ISL_1383372, EPI_ISL_1383601, EPI_ISL_1383692, EPI_ISL_1918325, EPI_ISL_2401053	Laboratoire national de sante, Microbiology, Virology	Laboratoire national de sante, Microbiology, Microbial Genomics Platform	Anke Wienecke-Baldacchino; Catherine Ragimbeau; Fatu Djabi; Jessica Tapp; Lise Pignon; Raoul Salmon; Tamir Abdelrahman; Trung Nguyen Nguyen
EPI_ISL_740051, EPI_ISL_740103	Laboratoire national de santé, Microbiology, Virology	Laboratoire national de santé, Microbiology, Microbial Genomics Platform	Anke Wienecke-Baldacchino; Catherine Ragimbeau; Fatu Djabi; Jessica Tapp; Tamir Abdelrahman
EPI_ISL_1917963, EPI_ISL_1917983, EPI_ISL_1917984	Laboratoires Reunis	Laboratoire national de sante, Microbiology, Microbial Genomics Platform	Anke Wienecke-Baldacchino; Bernard Weber; Catherine Ragimbeau; Fatu Djabi; Jessica Tapp; Lise Pignon; Raoul Salmon; Tamir Abdelrahman
EPI_ISL_1384454, EPI_ISL_1522940, EPI_ISL_2400706	Laboratoires d'analyses medicales - Ketterthill	Laboratoire national de sante, Microbiology, Microbial Genomics Platform	Anke Wienecke-Baldacchino; Caroline Scheiber; Catherine Ragimbeau; Fatu Djabi; Jessica Tapp; Lise Pignon; Raoul Salmon; Serge Vedy; Tamir Abdelrahman
EPI_ISL_1395935	Laboratorio Central De Redes y Programas	Grupo de Genómica y Bioinformática del Instituto de Investigación de la Cadena Láctea CONICET-INTA on behalf of 'Proyecto Argentino Interinstitucional de genomica de SARS-CoV-2' (PAIS Consortium)	AF; Amadio; Antonieta Cayré; Eberhardt; Gerardo Andino; Irazoqui; Laura Lescano; MF; Natalia Ruiz Diaz
EPI_ISL_2534969	Laboratorio Central Noel Nutels	Bioinformatics Laboratory / LNCC	Alessandra P Lamarca; Alexandra L Gerber; Amilcar Tanuri; Ana Paula de C Guimaraes; Ana Tereza R Vasconcelos; Andrea Cony Cavalcanti; Caio Luiz Pereira Ribeiro; Cassia Alves; Cintia Policarpo; Claudia Maria Braga de Mello; Cristiane Gomes da Silva; Diana Mariani; Douglas Terra Machado; Flavio Dias da Silva; Gleidson da Silva de Oliveira; Leandro Magalhaes de Souza; Liliane Cavalcante; Luiz G P de Almeida; Marcio Henrique de Oliveira Garcia; Mario Sergio Ribeiro;

			Ronaldo da Silva F Jr; Silvia Carvalho
EPI_ISL_1395959	Laboratorio Central de Salud Pública de la Provincia de Jujuy	Instituto de Patologia Vegetal (CIAP-INTA) on behalf of 'Proyecto Argentino Interinstitucional de genomica de SARS-CoV-2' (PAIS Consortium)	A. Miguel Alejandro Charre; Amadio; Ariel David Fridman; Claudia Mamani; Debat, HJ.; FD; Fabiana Vaca.; Fernández; Irazoqui, M.; Marquez, N.
EPI_ISL_792515, EPI_ISL_1395866, EPI_ISL_1395898, EPI_ISL_2363542	Laboratorio Central de la Ciudad de Santa Fe	Grupo de Genómica y Bioinformática del Instituto de Investigación de la Cadena Láctea CONICET-INTA on behalf of 'Proyecto Argentino Interinstitucional de genomica de SARS-CoV-2' (PAIS Consortium)	AF; Amadio; C; Eberhardt; G; Irazoqui; JM; MF; Mugna; Ojeda; Pastor; Rompato; V
EPI_ISL_1395978, EPI_ISL_2271705	Laboratorio Central, Ministerio de Salud Cordoba	Instituto de Patologia Vegetal (CIAP-INTA) on behalf of 'Proyecto Argentino Interinstitucional de genomica de SARS-CoV-2' (PAIS Consortium)	Barbas, G.; Castro, G.; Debat, HJ.; FD; Fernandez; Fernández; M; M.B.; Marquez, N.; Pisano; Re, V.
EPI_ISL_2007567	Laboratorio Central, Ministerio de Salud Córdoba	Instituto de Patologia Vegetal (CIAP-INTA) on behalf of 'Proyecto Argentino Interinstitucional de genomica de SARS-CoV-2' (PAIS Consortium)	A; Amadio; Barbas, G.; Castro, G.; Debat, HJ.; FD; Fernández; Irazoqui; M; M.B.; Marquez, N.; Pisano; Re, V.
EPI_ISL_2139519	Laboratorio Exame	Universidade Federal de Ciencias da Saude de Porto Alegre	Gabriel Dickin Caldana et al.; Vinícius Bonetti Franceschi
EPI_ISL_2536361	Laboratorio HUB -Azienda Ospedaliero Universitaria - AOU – Cagliari	Laboratorio SPOKE Biologia Molecolare -Azienda Ospedaliero Universitaria - AOU – Cagliari	Alessandra Scano; Ferdinando Coghe; Germano Orrù; Miriam Loddo; Riccardo Cappai; Sara Fais; Valentina Medda
EPI_ISL_2462063	Laboratorio de Biologia Molecular, Hospital San Pedro Claver	Microbiologia Molecular, Instituto SELADIS, Universidad Mayor de San Andrés	Aneth Vasquez Michel; Carmen Delgado Barrera; Oscar M. Rollano-Peñaloza; Sandra Miranda Sardon
EPI_ISL_1278277, EPI_ISL_1278278, EPI_ISL_2600378	Laboratorio de Biologia Molecular, Hospital San Pedro Claver	Molecular Genetics Laboratory, Instituto de Investigaciones Químicas, Universidad Mayor de San Andrés	Aneth Vasquez Michel; Carmen Delgado Barrera; Oscar M. Rollano-Peñaloza; Sandra Miranda Sardon
EPI_ISL_1278281, EPI_ISL_1278284	Laboratorio de Biologia Molecular, SEDES-Potosi	Molecular Genetics Laboratory, Instituto de Investigaciones Químicas, Universidad Mayor de San Andrés	Aneth Vasquez Michel; Oscar M. Rollano-Peñaloza
EPI_ISL_626564	Laboratorio de Biología Molecular, Facultad de Medicina, Universidad de Atacama, Copiapo, Chile/ FONDAP CRG, Universidad Andrés Bello, Santiago, Chile	Center for Mathematical Modeling and Center for Genome Regulation. Santiago, Chile	Allende ML; Bastias M; Castro E; Echeverría C; González M; M; Maass A; Manríquez R; Meneses C.; Montecino; Orellana A; Sanhueza D; Travisany D
EPI_ISL_591534	Laboratorio de Infectologia y virologia molecular	Center for Mathematical Modeling and Center for Genome Regulation. Santiago, Chile	Allende ML; Ferres M.; Gaete A; González M; Maass A; Palma R; Travisany D; Urra C; Valiente F; Varas M
EPI_ISL_792441	Laboratorio de Inmunología del Hospital Perrando e Instituto de Medicina Regional de la UNNE	Instituto de Biotecnología, IABIMO (CONICET), Instituto de Virología, IVIT(CONICET), Instituto de Patobiología, IPVET(CONICET), CICVyA, INTA on behalf of 'Proyecto Argentino Interinstitucional de genomica de SARS-CoV-2' (PAIS Consortium)	A; AF; AJ; AV; Asurmendi; Ayala; Bengoa Luoni; Cacciabué; Cayré; D; Deluca; Distéfano; Farber; Fass; Foussal; G; GA; Giusiano; Gómez; H; König; L; LC; Lescano; Lozano Calderón; Lucero; M; MD; MG; MPD; MV; Marin; Muñoz Hidalgo; NA; NB; PA; Paniego; Pedroarias; Peralta; Puebla; Rivarola; S; VC; Vera; Viegas, M.; Zavallo
EPI_ISL_953422	Laboratorio de Investigaciones de Baney	"Swiss Tropical and Public Health Institute"	"Carlos Cortes; Bonifacio Manguire Nlavo; Claudia Daubenberger; Diosdado Odjama Nseng Ada; Elizabeth Nyakarungu; Guillermo Garcia; Maximilian Mpina; Mitoha Ondo O Ayekaba; Philip Wonder Phiri"; Salome Hosch; Tobias Schindler
EPI_ISL_1673329, EPI_ISL_1673330, EPI_ISL_1700675, EPI_ISL_1700678, EPI_ISL_1700685, EPI_ISL_1700686, EPI_ISL_1700687, EPI_ISL_2002669, EPI_ISL_2002670, EPI_ISL_2002686, EPI_ISL_2002687, EPI_ISL_2002688			
see above	Laboratorio de Investigaciones de Baney	Swiss Tropical and Public Health Institute	Bonifacio Manguire Nlavo; Carlos Cortes; Claudia Daubenberger; Diosdado Odjama Nseng Ada; Elizabeth Nyakarungu; Guillermo Garcia; Maximilian Mpina; Mitoha Ondo O Ayekaba; Philip Wonder Phiri; Philipp Wagner; Salome Hosch; Tobias Schindler; Yahya Maidane
EPI_ISL_648316, EPI_ISL_648339, EPI_ISL_648361	Laboratorio de Investigaciones de Baney	University Hospital Basel, Clinical Bacteriology	Adrian Egli; Alfredo Mari; Bonifacio Manguire Nlavo; Carlos Cortes; Claudia Daubenberger; Diosdado Odjama Nseng Ada; Elizabeth Nyakarungu; Guillermo Garcia; Helena Seth-Smith; Madlen Stange; Maximilian Mpina; Mitoha Ondo O Ayekaba; Philip Wonder Phiri; Salome Hosch; Tim Roloff; Tobias Schindler
EPI_ISL_2462066	Laboratorio de Referencia Departamental en Inmunología, Sedes-Pando	Molecular Genetics Laboratory, Instituto de Investigaciones Químicas, Universidad Mayor de San Andrés	Aneth Vasquez Michel; Carmen Delgado Barrera; Oscar M. Rollano-Peñaloza; Sandra Miranda Sardon
EPI_ISL_1137619	Laboratorio de Salud Publica de Cauca	Instituto Nacional de Salud- Dirección de Investigación en Salud Pública	Carlos Franco-Muñoz; Diego A. Álvarez-Díaz; Diego Andrés Prada; Gerardo Santamaría; Hector Alejandro Ruiz-Moreno; Jhonnatan Reales-González; Julian Naizaque; Katherine Laiton-Donato; Magdalena Wiesner; Marcela Mercado-Reyes.; Maria T. Herrera-Sepúlveda; Martha Lucia Ospina Martínez; Sheryll Corchuelo
EPI_ISL_1494950	Laboratorio de Salud Publica de Santander	Instituto Nacional de Salud- Dirección de Investigación en Salud Pública	Carlos Franco-Muñoz; Carmen Osorio; Diana Malo; Diego A. Álvarez-Díaz; Diego Andrés Prada; Gerardo Santamaría; Hector Alejandro Ruiz-Moreno; Jhonnatan Reales-González; Juan Camilo Martinez; Julian Naizaque; Katherine Laiton-Donato; Lisseth Pardo; Magdalena Wiesner; Marcela Mercado-Reyes; Maria T. Herrera-Sepúlveda; Marta Lopez Blanco; Martha Lucia Ospina Martinez; Paola Rojas; Sergio Gomez; Sheryll Corchuelo; Ángela Alarcon Cruz
EPI_ISL_1396071	Laboratorio de Salud Pública	Instituto de Patologia Vegetal (CIAP-INTA) on behalf of 'Proyecto Argentino Interinstitucional de genomica de SARS-CoV-2' (PAIS Consortium)	A. Mariana B. Selmerón; Amadio; Ana Maria Zamora; Dardo E. Costas; Debat, HJ.; FD; Fernández; Graciela Alabarse.; Gustavo Ruìz de Huidobro; Irazoqui, M.; Marquez, N.
EPI_ISL_1017701	Laboratorio de Salud Pública de Boyacá	Instituto Nacional de Salud- Dirección de Investigación en Salud Pública	Carlos Franco-Muñoz; Diego A. Álvarez-Díaz; Diego Andrés Prada; Gerardo Santamaría; Hector Alejandro Ruiz-Moreno; Jhonnatan Reales-González; Julian Naizaque; Katherine Laiton-Donato; Magdalena Wiesner; Marcela Mercado-Reyes; Maria T. Herrera-Sepúlveda; Martha Lucia Ospina Martinez; Sheryll Corchuelo
EPI_ISL_792358, EPI_ISL_792364	Laboratorio de Virologia - HIEAyC San Juan de Dios	Área de Secuenciación del Laboratorio de Virología del Hospital de Niños Dr. Ricardo Gutierrez on behalf of 'Proyecto Argentino Interinstitucional de genomica de SARS-CoV-2' (PAIS Consortium)	A; Colmeiro; Ercole; Ferioli; Gatelli; Goya; LE; Lusso; M; MI; MS; Nabaes Jodar; Natale; R; S; Valinotto; Viegas, M.
EPI_ISL_717860	Laboratorio de Virologia Molecular / UFRJ	Bioinformatics Laboratory / LNCC	Alexandra L Gerber; Amilcar Tanuri; Ana Paula de C Guimarães; Ana Tereza R de Vasconcelos; Andréa Cony Cavalcanti; Carolina M Voloch; Claudia dos Santos Rodrigues; Cynthia C Cardoso; Diana Mariani; Luiz G P de Almeida; Otavio Bustrolini; Ronaldo da Silva F Jr; Terezinha M P P Castiñeira
EPI_ISL_1396251	Laboratorio de Virología del Hospital de Niños Dr. Ricardo Gutierrez	Biocódices SA. on behalf of 'Proyecto Argentino Interinstitucional de genomica de SARS-CoV-2' (PAIS Consortium)	A; Acevedo; Alexay; Alvarez Lopez; Barreda Frank; Berros; C; Dopazo, H.; E; G; Grandis; J; JM; Jacques; Labarta; M; ME; Medina; Mistchenko; N; O; S; Streitenberger; Thomas; Villegas; Zubrzycki J
EPI_ISL_1395787, EPI_ISL_1396220, EPI_ISL_2007478	Laboratorio de Virología del Hospital de Niños Dr. Ricardo Gutierrez	Área de Secuenciación del Laboratorio de Virología del Hospital de Niños Dr. Ricardo Gutierrez on behalf of 'Proyecto Argentino Interinstitucional de genomica de SARS-CoV-2' (PAIS Consortium)	A; Acevedo; Acuña; Alexay; Alvarez Lopez; Barreda Frank; C; D; E; G; Goya; Grandis; Jacques; LE; Labarta; Lusso; M; ME; MI; Medina; Mistchenko; N; Nabaes Jodar; Natale; O; S; Streitenberger; Thomas; Valinotto; Viegas, M.; Villegas
EPI_ISL_1396112	Laboratorio de Virus Respiratorios y Neurovirosis. Hospital Señor del Milagro	Instituto de Patologia Vegetal (CIAP-INTA) on behalf of 'Proyecto Argentino Interinstitucional de genomica de SARS-CoV-2' (PAIS Consortium)	A. Dra. Raskovsky Viviana; Amadio; Debat, HJ.; Dr. Lavaque Esteban; Dra. Veronica Lesser. Tecnica: Pamela Cajal; FD; Fernanda Aguero.; Fernández; Irazoqui, M.; Marquez, N.
EPI_ISL_1395789, EPI_ISL_1395798, EPI_ISL_1395799	Laboratorio del Hospital Interzonal General de Agudos “Evita”	Área de Secuenciación del Laboratorio de Virología del Hospital de Niños Dr. Ricardo Gutierrez on behalf of 'Proyecto Argentino Interinstitucional de genomica de SARS-CoV-2' (PAIS Consortium)	Acuña; Alejandra Musto; Alexay; D; Erica Luczak; Goya; Isabel Desimone; LE; Lorena Serrano; Lusso; M; MI; Nabaes Jodar; Natale; Omar Grossi; Rubén Pelagamos; S; Valinotto; Viegas, M.
EPI_ISL_1396334	Laboratorio del Hospital Regional Ushuaia Gdor. Ernesto Campos	Nodo de Secuenciación Tierra del Fuego - Hospital Regional Ushuaia - Centro Austral De Investigaciones Científicas - Universidad Nacional De Tierra Del Fuego on behalf of 'Proyecto Argentino Interinstitucional de genomica de SARS-CoV-2' (PAIS Consortium)	Alejandro Ezequiel Rojas; Carina Andrea De Roccis; Carolina Beatriz Yulan; Cristina Fernanda Nardi; Fernando Gallego; Gabriel Alejandro Castro; Ivan Dario Gramundi; Manuel Fabian Boutureira; Santiago Guillermo Ceballos; Silvana Beatriz Cáceres
EPI_ISL_2467938	Laboratory for HIV and opportunistic infections diagnosis The Republican Research and Practical Center for Epidemiology and Microbiology (RRPCEM)	Laboratory for HIV and opportunistic infections diagnosis The Republican Research and Practical Center for Epidemiology and Microbiology (RRPCEM	Alina Drozd; Artur Akhremchuk; Elena Gasich; Hanna Gudel; Katsiaryna Belyakova; Kirill Bulda; Leonid Valentovich; Nastassia Kabankova
EPI_ISL_1138899, EPI_ISL_1209407, EPI_ISL_1508895, EPI_ISL_2080973	Laboratory for HIV and opportunistic infections diagnosis The Republican Research and Practical Center for Epidemiology and Microbiology (RRPCEM)	Laboratory for HIV and opportunistic infections diagnosis The Republican Research and Practical Center for Epidemiology and Microbiology (RRPCEM)	Alena Mikhalenka; Alexander Kilchevcky; Alina Drozd; Anatoly Krasko; Anna Gudel; Artur Akhremchuk; Elena Gasich; Katsiaryna Belyakova; Kirill Bulda; Leonid Valentovich; Nastassia Kabankova; Vladimir Gorbunov; Yauhen Sysaliatsin
EPI_ISL_435047	Laboratory of Applied Genetics	RSE "National Center for Biotechnology"	Alexandr Shevtsov; Asylulan Amirgazin; Ilyas Akhmetollayev; Ruslan Kalendar; Viktoriya Lutsay; Yerlan Ramanculov
EPI_ISL_2546901	Laboratory of Communicable Diseases	1. Laboratory of Communicable Diseases (Estonia); 2. Eurofins Genomics Europe Sequencing GmbH	Liidia Dotsenko et al.
EPI_ISL_1716736, EPI_ISL_2301723	Laboratory of Immunohematology, Division of Hematology	Greek Genome Center, Biomedical Research Foundation of the Academy of Athens (BRFAA)	Athanasia Mouzaki; Dimitrios Thanos; Emmanouil Athanasiadis; Giannis Vatsellas; Ioannis Vatsellas; Katerina Zoi; Theodoros Loupis
EPI_ISL_434478	Laboratory of Microbiology, Medical School, National and Kapodistrian University of Athens	Laboratory of Biology, Department of Medicine, Democritus University of Thrace	Bampali, M.; Dovrolis, N.; Froukala, E.; Gatzidou, E.; Kassela K.; N. and Karakasiliotis, I.; Spanakis; Stavropoulou, A.; Tsakris, A.; Veletza, S.
EPI_ISL_2301773, EPI_ISL_2312609	Laboratory of Molecular Biology, Mamatsio General Hospital of Kozani	Greek Genome Center, Biomedical Research Foundation of the Academy of Athens (BRFAA)	Dimitrios Thanos; Emmanouil Athanasiadis; Giannis Vatsellas; Katerina Zoi; Konstantina Gartzonika; Theodoros Loupis
EPI_ISL_2235248	Laboratory of Molecular Biology, Mamatsio General Hospital of Kozani	Institute of Applied Biosciences, Centre for Research and Technology Hellas	Anastasia Chatzidimitriou et al.
EPI_ISL_801676	Laboratory of Molecular Virology, Pontificia Universidad Católica de Chile	MSHS Pathogen Surveillance Program	Adolfo Garcia-Sastre; Adriana van De Guchte; Ajay Obla; Ana Maria Contreras; Ana S. Gonzalez-Reiche; Bremy Alburquerque; Carlos Palma; Constanza Maldonado; Edward C. Holmes; Eileen Serrano; Erick Salinas; Hala Alshammary; Harm van Bakel; Jayeeta Dutta; Jorge Levican; Juan Soto; Leonardo I. Almonacid; M. Belen Leyton; Marcela Ferres; Matthew M. Hernandez; Melissa Smith; Rafael A. Medina.; Robert Sebra; Shwetha Hara Sridhar; Tamara García-Salum; Viviana Simon; Ying-Chih Wang; Zenab Khan

EPI_ISL_1341150, EPI_ISL_1341381, EPI_ISL_1341504, EPI_ISL_1341641, EPI_ISL_1365638, EPI_ISL_1365743, EPI_ISL_1489929			
see above	Laboratory of Virology, National center of expertise	RSE "National Center of Expertise" and RSE "National center for Biotechnology"	Abdaliyev Askar; Amirgazin Asylulan; Balykbaev Kanat; Kamalova Dinara; Ramankulov Erlan; Sharipova Saule; Shevtsov Alexandr; Tungushbayev Talgat
EPI_ISL_454575	Laboratory of virology, National Center of Expertise	Laboratory of molecular-genetic research, National Center for Expertise, Kazakhstan National Center for Biotechnology, Kazakhstan	; Abdaliyev Askar; Akhmetollayev Ilyas; Amirgazin Asylulan; Aushakhmetova Zabira; Kalendar Ruslan; Lutsay Viktoriya; Rakhmetova Akbota; Ramankulov Yerlan; Shevtsov Alexandr
EPI_ISL_454585	Laboratory of virology, National Center of Expertise	Laboratory of molecular-genetic research, National Center for Expertise, Kazakhstan National Center for Biotechnology, Kazakhstan	; Abdaliyev Askar; Akhmetollayev Ilyas; Amirgazin Asylulan; Aushakhmetova Zabira; Kalendar Ruslan; Lutsay Viktoriya; Rakhmetova Akbota; Ramankulov Yerlan; Shevtsov Alexandr
EPI_ISL_454590	Laboratory of virology, National Center of Expertise	Laboratory of molecular-genetic research, National Center of Expertise, Kazakhstan National Center for Biotechnology, Kazakhstan	; Abdaliyev Askar; Akhmetollayev Ilyas; Amirgazin Asylulan; Aushakhmetova Zabira; Kalendar Ruslan; Lutsay Viktoriya; Rakhmetova Akbota; Ramankulov Yerlan; Shevtsov Alexandr
EPI_ISL_1334580, EPI_ISL_1364621, EPI_ISL_1364864, EPI_ISL_1448018, EPI_ISL_1448020	Laboratory of virology, National center of expertise	RSE "National Center for Biotechnology" and RSE "National Center of Expertise"	Abdaliyev Askar; Amirgazin Asylulan; Balykbaev Kanat; Kamalova Dinara; Ramankulov Yerlan; Sharipova Saule; Shevtsov Alexandr; Tungushbayev Talgat
EPI_ISL_2308475	Laboratório Central de Saúde Pública de Sergipe	Coordenação Geral de Laboratórios de Saúde Pública (CGLAB/DAEVS/SVS/MS)	Vagner Fonseca; et al.
EPI_ISL_792589, EPI_ISL_792613	Laboratório Central de Saúde Pública do Estado da Paraíba (LACEN-PB)	Laboratory of Respiratory Viruses and Measles, Oswaldo Cruz Institute, FIOCRUZ	Ana Carolina Mendonca; Anna Carolina Paixao; Dalane Loudal Florentino Teixeira; Fernando Motta; João Felipe Bezerra; Luciana Appolinario; Marilda Siqueira on behalf of the Fiocruz COVID-19 Genomic Surveillance Network; Paola Resende; Romero Henrique Teixeira de Vasconcelos; Thiago Franco de Oliveira Carneiro
EPI_ISL_729813	Laboratório Central de Saúde Pública do Estado do Rio Grande do Sul (LACEN-RS)	Laboratory of Respiratory Viruses and Measles, Oswaldo Cruz Institute, FIOCRUZ	Ana Carolina Mendonça; Anna Carolina Paixão; Fernando Motta; Luciana Appolinario; Marilda Siqueira on behalf of the Fiocruz COVID-19 Genomic Surveillance Network; Marilda Tereza Mar da Rosa; Paola Resende; Tatiana Schaffer Gregianini
EPI_ISL_2248765	Laboratório Central de Saúde Pública do Pará	Coordenação Geral de Laboratórios de Saúde Pública (CGLAB/DAEVS/SVS/MS)	Vagner Fonseca; et al.
EPI_ISL_2158599	Laboratório de Biologia Molecular Jean Piaget	MRCG at LSHTM, Genomics lab	Abdoulie Kanteh; Abdul Karim Sesay; Adul Candé; Aicha Balde; Aladje Balde; Bakary Sanyang; Bubacar Delgado Pinto Embaló; Dabiri Damilari; Edmira Maria da Costa; Erica Luis Maria Magalhães; Faatu Cassama; Mariama Kujabi; Milanca Agostinho Cá; Paulina Joãozinho da Costa Jarra Manneh; Rei José Pereira; Rui Inndi; Sainabou Laye Ndure; Simão Tchuda Bióté
EPI_ISL_779166	Laboratório de Microbiologia Molecular - Universidade FEEVALE	Bioinformatics Laboratory / LNCC	Alana Witt Hansen; Alessandra Pavan Lamarca da Silva; Alexandra L Gerber; Ana Karolina Eisen Antunes; Ana Luiza Ziulkoski; Ana Paula de C Guimarães; Ana Tereza R de Vasconcelos; Bruna Hermann; Fagner Henrique Heldt; Felipe Benites; Fernando Rosado Spilki; Juliana Schons; Juliane Deise Fleck; Karoline Schallenberger; Larissa Mallmann; Luiz G P de Almeida; Matheus Nunes Weber; Meriane Demoliner; Paula Rodrigues de Almeida; Ronaldo da Silva F Jr; Vyctoria Goes
EPI_ISL_2375797, EPI_ISL_2431436	Laboratório de Microbiologia Molecular - Universidade FEEVALE	Molecular Microbiology Laboratory	Alana Witt Hansen; Fernando Rosado Spilki; Flávio Silveira; Fágner Henrique Heldt; Juliana Schons Gularte; Juliane Deise Fleck; Mariana Soares da Silva; Matheus Nunes Weber; Meriane Demoliner; Micheli Filippi.; Paula Rodrigues de Almeida
EPI_ISL_1216917, EPI_ISL_2120258, EPI_ISL_2760396	Labormedizin Darmstadt	Robert Koch Institute	
EPI_ISL_1973556	Labormedizinisches Zentrum Dr Risch	Clinical Bacteriology	Adrian Egli; Alfredo Mari; Hans Hirsch; Helena MB Seth-Smith; Julia Bielicki; Karoline Leuzinger; Lorenz Risch; Madlen Stange; Manuel Battegay; Martin Risch; Nadia Wohlwend; Tim Roloff
EPI_ISL_1233663	Labormedizinisches Zentrum Dr Risch	University Hospital Basel, Clinical Bacteriology	Adrian Egli; Alfredo Mari; Hans Hirsch; Helena MB Seth-Smith; Julia Bielicki; Karoline Leuzinger; Lorenz Risch; Madlen Stange; Manuel Battegay; Martin Risch; Nadia Wohlwend; Tim Roloff
EPI_ISL_2614379	Labortorio Central de Saude Publica do Estado do Rio de Janeiro (LACEN/RJ)	Laboratory of Respiratory Viruses and Measles, Oswaldo Cruz Institute, FIOCRUZ	Alice Sampaio Rocha; Ana Carolina Mendonca; Andrea Cony Cavalcanti; Anna Carolina Paixao; Elisa Cavalcante Pereira; Fernando Motta; Luciana Appolinario; Marilda Siqueira on behalf of the Fiocruz COVID-19 Genomic Surveillance Network; Paola Resende; Renata Serrano Lopes; Taina Venas
EPI_ISL_1941367, EPI_ISL_1941374, EPI_ISL_1941376, EPI_ISL_2614390	Landesamt für Verbraucherschutz Sachsen Anhalt, Magdeburg	Institute of Medical Microbiology and Hospital Hygiene	Aljoscha Tersteegen; Prof. Dr. Achim Kaasch
EPI_ISL_2760184, EPI_ISL_2761652	Landesgesundheitsamt Baden-Württemberg	Robert Koch Institute	
EPI_ISL_812277	Landstuhl Regional Medical Center	United States Air Force School of Aerospace Medicine	Amanda Javorina; Anthony Fries; Clarise Starr; Cole Anderson; Elizabeth Macias; Fritz Castillo; Jennifer Meyer; Sarah Purves; William Gruner
EPI_ISL_2443545	Lboratorio Central de Saude Publica do Estado do Parana (LACEN/PR)	Laboratory of Respiratory Viruses and Measles, Oswaldo Cruz Institute, FIOCRUZ	Alice Sampaio Rocha; Ana Carolina Mendonca; Anna Carolina Paixao; Elisa Cavalcante Pereira; Fernando Motta; Irina Riediger; Luciana Appolinario; Marilda Siqueira on behalf of the Fiocruz COVID-19 Genomic Surveillance Network; Paola Resende; Renata Serrano Lopes; Taina Venas
EPI_ISL_2521701	Libramont	Plateforme de testing Namuroise	Degosserie Jonathan; Demars Aurore; Denis Olivier; Lesly Nyinkeu Kemamen; Maschietto Céline; Mullier François; Nobis Chloé; Otto Gaetan
EPI_ISL_1188862, EPI_ISL_1536300, EPI_ISL_1584529, EPI_ISL_1631042, EPI_ISL_1652724, EPI_ISL_1652738, EPI_ISL_1652761, EPI_ISL_1652763, EPI_ISL_1652850, EPI_ISL_1652890, EPI_ISL_1653187, EPI_ISL_1698227, EPI_ISL_1698319, EPI_ISL_1698325, EPI_ISL_1698416, EPI_ISL_1698440, EPI_ISL_1698543, EPI_ISL_1699101, EPI_ISL_1700038, EPI_ISL_1700236, EPI_ISL_1718381, EPI_ISL_1718501, EPI_ISL_1718585, EPI_ISL_1718588, EPI_ISL_1718597, EPI_ISL_1718598, EPI_ISL_1718630, EPI_ISL_1718683, EPI_ISL_1718721, EPI_ISL_1718772, EPI_ISL_1718882, EPI_ISL_1719616, EPI_ISL_1740600, EPI_ISL_1740616, EPI_ISL_1740654, EPI_ISL_1740707, EPI_ISL_1740750, EPI_ISL_1740763, EPI_ISL_1740778, EPI_ISL_1740819, EPI_ISL_1741186, EPI_ISL_1758301, EPI_ISL_1758376, EPI_ISL_1758890, EPI_ISL_1759190, EPI_ISL_1759274, EPI_ISL_1790461, EPI_ISL_1790538, EPI_ISL_1790547, EPI_ISL_1790596, EPI_ISL_1790745, EPI_ISL_1790771, EPI_ISL_1790809, EPI_ISL_1790990, EPI_ISL_1805965, EPI_ISL_1806511, EPI_ISL_1806517, EPI_ISL_1806521, EPI_ISL_1806550, EPI_ISL_1806561, EPI_ISL_1806594, EPI_ISL_1806605, EPI_ISL_1806633, EPI_ISL_1806670, EPI_ISL_1806695, EPI_ISL_1806837, EPI_ISL_1806846, EPI_ISL_1806852, EPI_ISL_1829212, EPI_ISL_1829220, EPI_ISL_1829233, EPI_ISL_1829237, EPI_ISL_1829254, EPI_ISL_1829258, EPI_ISL_1829279, EPI_ISL_1829281, EPI_ISL_1829282, EPI_ISL_1829360, EPI_ISL_1829382, EPI_ISL_1829413, EPI_ISL_1829430, EPI_ISL_1829440, EPI_ISL_1829458, EPI_ISL_1829818, EPI_ISL_1829838, EPI_ISL_1829839, EPI_ISL_1829843, EPI_ISL_1829854, EPI_ISL_1829860, EPI_ISL_1829878, EPI_ISL_1829914, EPI_ISL_1829930, EPI_ISL_1829964, EPI_ISL_1829966, EPI_ISL_1829979, EPI_ISL_1829983, EPI_ISL_1829994, EPI_ISL_1830046, EPI_ISL_1830065, EPI_ISL_1830069, EPI_ISL_1830077, EPI_ISL_1830080, EPI_ISL_1830356, EPI_ISL_1830480, EPI_ISL_1830543, EPI_ISL_1830558, EPI_ISL_1830575, EPI_ISL_1830586, EPI_ISL_1830711, EPI_ISL_1831002, EPI_ISL_1831023, EPI_ISL_1857806, EPI_ISL_1857812, EPI_ISL_1857859, EPI_ISL_1857877, EPI_ISL_1857887, EPI_ISL_1857942, EPI_ISL_1857943, EPI_ISL_1857977, EPI_ISL_1858006, EPI_ISL_1858037, EPI_ISL_1858086, EPI_ISL_1858108, EPI_ISL_1858134, EPI_ISL_1858139, EPI_ISL_1858156, EPI_ISL_1912014, EPI_ISL_1912090, EPI_ISL_1912101, EPI_ISL_1912160, EPI_ISL_1912162, EPI_ISL_1912163, EPI_ISL_1912170, EPI_ISL_1912184, EPI_ISL_1912361, EPI_ISL_1912364, EPI_ISL_1912365, EPI_ISL_1912394, EPI_ISL_1912396, EPI_ISL_1912401, EPI_ISL_1912409, EPI_ISL_1912413, EPI_ISL_1912418, EPI_ISL_1912430, EPI_ISL_1912443, EPI_ISL_1912455, EPI_ISL_1912461, EPI_ISL_1912486, EPI_ISL_1912498, EPI_ISL_1912516, EPI_ISL_1912521, EPI_ISL_1912523, EPI_ISL_1912553, EPI_ISL_1912564, EPI_ISL_1921169, EPI_ISL_1921174, EPI_ISL_1921190, EPI_ISL_1921197, EPI_ISL_1921211, EPI_ISL_1921217, EPI_ISL_1921227, EPI_ISL_1921241, EPI_ISL_1921251, EPI_ISL_1921253, EPI_ISL_1921262, EPI_ISL_1921263, EPI_ISL_1921278, EPI_ISL_1921279, EPI_ISL_1921290, EPI_ISL_1921297, EPI_ISL_1921313, EPI_ISL_1921333, EPI_ISL_1921343, EPI_ISL_1921353, EPI_ISL_1921360, EPI_ISL_1921362, EPI_ISL_1921370, EPI_ISL_1921376, EPI_ISL_1921377, EPI_ISL_1921379, EPI_ISL_1921390, EPI_ISL_1921396, EPI_ISL_1921400, EPI_ISL_1921403, EPI_ISL_1921404, EPI_ISL_1921411, EPI_ISL_1921429, EPI_ISL_1921438, EPI_ISL_1921459, EPI_ISL_1921496, EPI_ISL_1921572, EPI_ISL_1985222, EPI_ISL_1985411, EPI_ISL_1985811, EPI_ISL_1985957, EPI_ISL_1986032, EPI_ISL_1986099, EPI_ISL_1986107, EPI_ISL_1986136, EPI_ISL_1986139, EPI_ISL_1986167, EPI_ISL_1986218, EPI_ISL_1986589, EPI_ISL_1986617, EPI_ISL_1986626, EPI_ISL_1986639, EPI_ISL_1986692, EPI_ISL_1986707, EPI_ISL_1986712, EPI_ISL_1986733, EPI_ISL_1986741, EPI_ISL_1986756, EPI_ISL_1986810, EPI_ISL_1986812, EPI_ISL_1986813, EPI_ISL_1986858, EPI_ISL_1986871, EPI_ISL_1986900, EPI_ISL_1986933, EPI_ISL_1986972, EPI_ISL_1986974, EPI_ISL_1986993, EPI_ISL_1987000, EPI_ISL_1987001, EPI_ISL_1987041, EPI_ISL_1987050, EPI_ISL_1987056, EPI_ISL_1987126, EPI_ISL_1987140, EPI_ISL_1987152, EPI_ISL_1987156, EPI_ISL_1987188, EPI_ISL_1987207, EPI_ISL_1987214, EPI_ISL_1987239, EPI_ISL_1987267, EPI_ISL_1987269, EPI_ISL_1987276, EPI_ISL_1987341, EPI_ISL_1987366, EPI_ISL_2004359, EPI_ISL_2004369, EPI_ISL_2004385, EPI_ISL_2004457, EPI_ISL_2004537, EPI_ISL_2004539, EPI_ISL_2004579, EPI_ISL_2004912, EPI_ISL_2005010, EPI_ISL_2005025, EPI_ISL_2005065, EPI_ISL_2005114, EPI_ISL_2005117, EPI_ISL_2005120, EPI_ISL_2005129, EPI_ISL_2005239, EPI_ISL_2005241, EPI_ISL_2005285, EPI_ISL_2005288, EPI_ISL_2005291, EPI_ISL_2005295, EPI_ISL_2005347, EPI_ISL_2005413, EPI_ISL_2005426, EPI_ISL_2005438, EPI_ISL_2005476, EPI_ISL_2005519, EPI_ISL_2005548, EPI_ISL_2005573, EPI_ISL_2005588, EPI_ISL_2005637, EPI_ISL_2006080, EPI_ISL_2006197, EPI_ISL_2006210, EPI_ISL_2006228, EPI_ISL_2006274, EPI_ISL_2021410, EPI_ISL_2021711, EPI_ISL_2021728, EPI_ISL_2021747, EPI_ISL_2021784, EPI_ISL_2021813, EPI_ISL_2021828, EPI_ISL_2021843, EPI_ISL_2021971, EPI_ISL_2022066, EPI_ISL_2022080, EPI_ISL_2022979, EPI_ISL_2080421, EPI_ISL_2091509, EPI_ISL_2091604, EPI_ISL_2091638, EPI_ISL_2091649, EPI_ISL_2091656, EPI_ISL_2091672, EPI_ISL_2091685, EPI_ISL_2091694, EPI_ISL_2091754, EPI_ISL_2091772, EPI_ISL_2092475, EPI_ISL_2092484, EPI_ISL_2092496, EPI_ISL_2092516, EPI_ISL_2092526, EPI_ISL_2092548, EPI_ISL_2092553, EPI_ISL_2092565, EPI_ISL_2092586, EPI_ISL_2092595, EPI_ISL_2092599, EPI_ISL_2092612, EPI_ISL_2092613, EPI_ISL_2092645, EPI_ISL_2117851, EPI_ISL_2118638, EPI_ISL_2118835, EPI_ISL_2120591, EPI_ISL_2138082, EPI_ISL_2138321, EPI_ISL_2171965, EPI_ISL_2172116, EPI_ISL_2172121, EPI_ISL_2172139, EPI_ISL_2172153, EPI_ISL_2172182, EPI_ISL_2172223, EPI_ISL_2172229, EPI_ISL_2172255, EPI_ISL_2172265, EPI_ISL_2172279, EPI_ISL_2172324, EPI_ISL_2172328, EPI_ISL_2172366, EPI_ISL_2172374, EPI_ISL_2172380, EPI_ISL_2172381, EPI_ISL_2172386, EPI_ISL_2172401, EPI_ISL_2198894, EPI_ISL_2198964, EPI_ISL_2236076, EPI_ISL_2236240, EPI_ISL_2236309, EPI_ISL_2237059, EPI_ISL_2237091, EPI_ISL_2237098, EPI_ISL_2237307, EPI_ISL_2237357, EPI_ISL_2237547, EPI_ISL_2237628, EPI_ISL_2238374, EPI_ISL_2275348, EPI_ISL_2296700, EPI_ISL_2296702, EPI_ISL_2317213, EPI_ISL_2317305, EPI_ISL_2317647, EPI_ISL_2317712, EPI_ISL_2317957, EPI_ISL_2318018, EPI_ISL_2318038, EPI_ISL_2318104, EPI_ISL_2318122, EPI_ISL_2318267, EPI_ISL_2318311, EPI_ISL_2318328, EPI_ISL_2318395, EPI_ISL_2318416, EPI_ISL_2318623, EPI_ISL_2318697, EPI_ISL_2346591, EPI_ISL_2346744, EPI_ISL_2347582, EPI_ISL_2347591, EPI_ISL_2347593, EPI_ISL_2347771, EPI_ISL_2347822, EPI_ISL_2348024, EPI_ISL_2348474, EPI_ISL_2353654, EPI_ISL_2353656, EPI_ISL_2353777, EPI_ISL_2366247, EPI_ISL_2366375, EPI_ISL_2366596, EPI_ISL_2392312, EPI_ISL_2392657, EPI_ISL_2393029, EPI_ISL_2393224, EPI_ISL_2394464, EPI_ISL_2394786, EPI_ISL_2395231, EPI_ISL_2396264, EPI_ISL_2396284, EPI_ISL_2435213, EPI_ISL_2435618, EPI_ISL_2435642, EPI_ISL_2435763, EPI_ISL_2436037, EPI_ISL_2436119, EPI_ISL_2436659, EPI_ISL_2436704, EPI_ISL_2437001, EPI_ISL_2437002, EPI_ISL_2437215, EPI_ISL_2446310, EPI_ISL_2446424, EPI_ISL_2446497, EPI_ISL_2446596, EPI_ISL_2446859, EPI_ISL_2447029, EPI_ISL_2447706, EPI_ISL_2456130, EPI_ISL_2457113, EPI_ISL_2457394, EPI_ISL_2457439, EPI_ISL_2468137, EPI_ISL_2468228, EPI_ISL_2468495, EPI_ISL_2468860, EPI_ISL_2468939, EPI_ISL_2469049, EPI_ISL_2469171, EPI_ISL_2485572, EPI_ISL_2486650, EPI_ISL_2486673, EPI_ISL_2486698, EPI_ISL_2486787, EPI_ISL_2486814, EPI_ISL_2486997, EPI_ISL_2487913, EPI_ISL_2511446, EPI_ISL_2511575, EPI_ISL_2511629, EPI_ISL_2511701, EPI_ISL_2513190, EPI_ISL_2513212, EPI_ISL_2513628, EPI_ISL_2513873, EPI_ISL_2513963, EPI_ISL_2514252, EPI_ISL_2514946, EPI_ISL_2515030, EPI_ISL_2515153, EPI_ISL_2515554, EPI_ISL_2515671, EPI_ISL_2515783, EPI_ISL_2515839, EPI_ISL_2515865, EPI_ISL_2515922, EPI_ISL_2515939, EPI_ISL_2516029, EPI_ISL_2537543, EPI_ISL_2537560, EPI_ISL_2538070, EPI_ISL_2538143, EPI_ISL_2538268, EPI_ISL_2538470, EPI_ISL_2538649, EPI_ISL_2538760, EPI_ISL_2538914, EPI_ISL_2538949, EPI_ISL_2539036, EPI_ISL_2540387, EPI_ISL_2541733, EPI_ISL_2541787, EPI_ISL_2541796, EPI_ISL_2552342, EPI_ISL_2552383, EPI_ISL_2552427, EPI_ISL_2554023, EPI_ISL_2554041, EPI_ISL_2554143, EPI_ISL_2554251, EPI_ISL_2568046, EPI_ISL_2568076, EPI_ISL_2568622, EPI_ISL_2569319, EPI_ISL_2569501, EPI_ISL_2569627, EPI_ISL_2569868, EPI_ISL_2569958, EPI_ISL_2570036, EPI_ISL_2570114, EPI_ISL_2570198, EPI_ISL_2570221, EPI_ISL_2570519, EPI_ISL_2570661, EPI_ISL_2570689, EPI_ISL_2605241, EPI_ISL_2605296, EPI_ISL_2605786, EPI_ISL_2605788, EPI_ISL_2606028, EPI_ISL_2606103, EPI_ISL_2606170, EPI_ISL_2606192, EPI_ISL_2606298, EPI_ISL_2606326, EPI_ISL_2606344, EPI_ISL_2606757, EPI_ISL_2606940, EPI_ISL_2638216, EPI_ISL_2638345, EPI_ISL_2638642, EPI_ISL_2639518, EPI_ISL_2651746, EPI_ISL_2664362, EPI_ISL_2681809, EPI_ISL_2682415, EPI_ISL_2682451, EPI_ISL_2682913, EPI_ISL_2699870, EPI_ISL_2700047, EPI_ISL_2700629, EPI_ISL_2700642, EPI_ISL_2700845, EPI_ISL_2700859, EPI_ISL_2701777, EPI_ISL_2702011, EPI_ISL_2702565, EPI_ISL_2702612, EPI_ISL_2702715, EPI_ISL_2703045, EPI_ISL_2703106, EPI_ISL_2703432, EPI_ISL_2703734, EPI_ISL_2704276, EPI_ISL_2704417, EPI_ISL_2717169, EPI_ISL_2717685, EPI_ISL_2717687, EPI_ISL_2717781, EPI_ISL_2718002, EPI_ISL_2718159, EPI_ISL_2718378, EPI_ISL_2729619, EPI_ISL_2729737, EPI_ISL_2730547, EPI_ISL_2765990, EPI_ISL_2766719, EPI_ISL_2766911, EPI_ISL_2767054, EPI_ISL_2767262, EPI_ISL_2767944, EPI_ISL_2780194			
see above	Lighthouse Lab in Alderley Park	Wellcome Sanger Institute for the COVID-19 Genomics UK (COG-UK) Consortium	Cordelia Langford; David K. Jackson; Dominic Kwiatkowski; Ewan Harrison; Ian Johnston; Jacquelyn Wynn; Jeffrey Barrett; John Sillitoe on behalf of the Wellcome Sanger Institute COVID-19 Surveillance Team; Mairead Hyland; Roberto Amato; Sonia Goncalves; The Lighthouse Lab in Alderley Park and Alex Alderton
EPI_ISL_987831, EPI_ISL_1206341, EPI_ISL_1327583, EPI_ISL_1327672, EPI_ISL_1409773, EPI_ISL_1410101, EPI_ISL_1454606, EPI_ISL_1473648, EPI_ISL_1484087, EPI_ISL_1505036, EPI_ISL_1505038, EPI_ISL_1517918, EPI_ISL_1519271, EPI_ISL_1519290, EPI_ISL_1519300, EPI_ISL_1534758, EPI_ISL_1534917, EPI_ISL_1534938, EPI_ISL_1534945, EPI_ISL_1537321, EPI_ISL_1564086, EPI_ISL_1564102, EPI_ISL_1564139, EPI_ISL_1564187, EPI_ISL_1564328, EPI_ISL_1564772, EPI_ISL_1583942, EPI_ISL_1583979, EPI_ISL_1594728, EPI_ISL_1594918, EPI_ISL_1595457, EPI_ISL_1615751, EPI_ISL_1615877, EPI_ISL_1631162, EPI_ISL_1631219, EPI_ISL_1632008, EPI_ISL_1632316, EPI_ISL_1632335, EPI_ISL_1632384, EPI_ISL_1632412, EPI_ISL_1632426, EPI_ISL_1635371, EPI_ISL_1652964, EPI_ISL_1652981, EPI_ISL_1652993, EPI_ISL_1653090, EPI_ISL_1653104, EPI_ISL_1653197, EPI_ISL_1653403, EPI_ISL_1673061, EPI_ISL_1673142, EPI_ISL_1697491, EPI_ISL_1697908, EPI_ISL_1697919, EPI_ISL_1697963, EPI_ISL_1697964, EPI_ISL_1698273, EPI_ISL_1698274, EPI_ISL_1698283, EPI_ISL_1698332, EPI_ISL_1698361, EPI_ISL_1698410, EPI_ISL_1698429, EPI_ISL_1698436, EPI_ISL_1698521, EPI_ISL_1718942, EPI_ISL_1718959, EPI_ISL_1718989, EPI_ISL_1718997, EPI_ISL_1719027, EPI_ISL_1719042, EPI_ISL_1719062, EPI_ISL_1719099, EPI_ISL_1719134, EPI_ISL_1719142, EPI_ISL_1719584, EPI_ISL_1719618, EPI_ISL_1719629, EPI_ISL_1719736, EPI_ISL_1740580, EPI_ISL_1740781, EPI_ISL_1758509, EPI_ISL_1758558, EPI_ISL_1758645, EPI_ISL_1759267, EPI_ISL_1759884, EPI_ISL_1912066, EPI_ISL_1912114, EPI_ISL_1912119, EPI_ISL_1912157, EPI_ISL_1912173, EPI_ISL_1912203, EPI_ISL_1912205, EPI_ISL_2641718			
see above	Lighthouse Lab in Cambridge	Wellcome Sanger Institute for the COVID-19 Genomics UK (COG-UK) Consortium	Cordelia Langford; David K. Jackson; Dominic Kwiatkowski; Ewan Harrison; Ian Johnston; Jeffrey Barrett; John Sillitoe on behalf of the Wellcome Sanger Institute COVID-19 Surveillance Team; John Sillitoe on behalf of the Wellcome Sanger Institute COVID-19 Surveillance Team (http://www.sanger.ac.uk/covid-team); Rob Howes; Roberto Amato; Sonia Goncalves; The Lighthouse Lab in Cambridge and Alex Alderton
EPI_ISL_1175335, EPI_ISL_1484209, EPI_ISL_1583915, EPI_ISL_1632307, EPI_ISL_1634839, EPI_ISL_1634899, EPI_ISL_1634920, EPI_ISL_1634973, EPI_ISL_1719573, EPI_ISL_1719728, EPI_ISL_1740738, EPI_ISL_1741081, EPI_ISL_1741129, EPI_ISL_1741213, EPI_ISL_1741259, EPI_ISL_1790373, EPI_ISL_1806086, EPI_ISL_1806094, EPI_ISL_1806236, EPI_ISL_1806353, EPI_ISL_1829604, EPI_ISL_1829780, EPI_ISL_1830295, EPI_ISL_1831030, EPI_ISL_1912238, EPI_ISL_1912330, EPI_ISL_1912472, EPI_ISL_1986389, EPI_ISL_1987244, EPI_ISL_2005071, EPI_ISL_2005164, EPI_ISL_2005195, EPI_ISL_2005222, EPI_ISL_2005332, EPI_ISL_2005820, EPI_ISL_2005893, EPI_ISL_2005927, EPI_ISL_2006047, EPI_ISL_2006317, EPI_ISL_2006366, EPI_ISL_2006372, EPI_ISL_2021698, EPI_ISL_2021842, EPI_ISL_2092532, EPI_ISL_2092536, EPI_ISL_2117571, EPI_ISL_2117681, EPI_ISL_2117963, EPI_ISL_2171939, EPI_ISL_2237105, EPI_ISL_2238086, EPI_ISL_2296704, EPI_ISL_2316684, EPI_ISL_2353727, EPI_ISL_2366299, EPI_ISL_2394547, EPI_ISL_2395813, EPI_ISL_2436304, EPI_ISL_2446141, EPI_ISL_2457295, EPI_ISL_2486317, EPI_ISL_2487633, EPI_ISL_2511229, EPI_ISL_2511750, EPI_ISL_2511923, EPI_ISL_2513502, EPI_ISL_2513607, EPI_ISL_2513661, EPI_ISL_2515441, EPI_ISL_2516086, EPI_ISL_2552804, EPI_ISL_2554625, EPI_ISL_2568107, EPI_ISL_2568636, EPI_ISL_2605595, EPI_ISL_2605722, EPI_ISL_2640913, EPI_ISL_2651574, EPI_ISL_2663827, EPI_ISL_2682093, EPI_ISL_2717794, EPI_ISL_2718176, EPI_ISL_2718247, EPI_ISL_2718467, EPI_ISL_2730214, EPI_ISL_2780538, EPI_ISL_2782208			
see above	Lighthouse Lab in Glasgow	Wellcome Sanger Institute for the COVID-19 Genomics UK (COG-UK) Consortium	Anna Dominiczak and Alex Alderton; Carol Clugston; Cordelia Langford; David Gray; David K. Jackson; Dominic Kwiatkowski; Ewan Harrison; Harper VanSteenhouse; Ian Johnston; Jeffrey Barrett; John Sillitoe on behalf of the Wellcome Sanger Institute COVID-19 Surveillance Team; Roberto Amato; Sonia Goncalves; Yumi Kasai
EPI_ISL_1486890, EPI_ISL_1594669, EPI_ISL_1631645, EPI_ISL_1631923, EPI_ISL_1631945, EPI_ISL_1634507, EPI_ISL_1634517, EPI_ISL_1697977, EPI_ISL_1698079, EPI_ISL_1698093, EPI_ISL_1698106, EPI_ISL_1698144, EPI_ISL_1698322, EPI_ISL_1699414, EPI_ISL_1699676, EPI_ISL_1699753, EPI_ISL_1699770, EPI_ISL_1699979, EPI_ISL_1718344, EPI_ISL_1718359, EPI_ISL_1718509, EPI_ISL_1718668, EPI_ISL_1718691, EPI_ISL_1718934, EPI_ISL_1718935, EPI_ISL_1718951, EPI_ISL_1718987, EPI_ISL_1719015, EPI_ISL_1719056, EPI_ISL_1719079, EPI_ISL_1719162, EPI_ISL_1719212, EPI_ISL_1740858, EPI_ISL_1740882, EPI_ISL_1741398, EPI_ISL_1758205, EPI_ISL_1758283, EPI_ISL_1758919, EPI_ISL_1759178, EPI_ISL_1759218, EPI_ISL_1759272, EPI_ISL_1759278, EPI_ISL_1759335, EPI_ISL_1759819, EPI_ISL_1759847, EPI_ISL_1790473, EPI_ISL_1790475, EPI_ISL_1790529, EPI_ISL_1790532, EPI_ISL_1790560, EPI_ISL_1790590, EPI_ISL_1790610, EPI_ISL_1790613, EPI_ISL_1790633, EPI_ISL_1790679, EPI_ISL_1790701, EPI_ISL_1790728, EPI_ISL_1790753, EPI_ISL_1790774, EPI_ISL_1790783, EPI_ISL_1790799, EPI_ISL_1790800, EPI_ISL_1790817, EPI_ISL_1790829, EPI_ISL_1790832, EPI_ISL_1790848, EPI_ISL_1790883, EPI_ISL_1790888, EPI_ISL_1790893, EPI_ISL_1790904, EPI_ISL_1790905, EPI_ISL_1790917, EPI_ISL_1790928, EPI_ISL_1790940, EPI_ISL_1790949, EPI_ISL_1790954, EPI_ISL_1790998, EPI_ISL_1791005, EPI_ISL_1806011, EPI_ISL_1806342, EPI_ISL_1806406, EPI_ISL_1806433, EPI_ISL_1806601, EPI_ISL_1806709, EPI_ISL_1806761, EPI_ISL_1806767, EPI_ISL_1806848, EPI_ISL_1829206, EPI_ISL_1829207, EPI_ISL_1829225, EPI_ISL_1829226, EPI_ISL_1829228, EPI_ISL_1829230, EPI_ISL_1829236, EPI_ISL_1829246, EPI_ISL_1829248, EPI_ISL_1829249, EPI_ISL_1829250, EPI_ISL_1829259, EPI_ISL_1829261, EPI_ISL_1829268, EPI_ISL_1829277, EPI_ISL_1829283, EPI_ISL_1829293, EPI_ISL_1829312, EPI_ISL_1829313, EPI_ISL_1829334, EPI_ISL_1829340, EPI_ISL_1829355, EPI_ISL_1829370, EPI_ISL_1829372, EPI_ISL_1829386, EPI_ISL_1829392, EPI_ISL_1829394, EPI_ISL_1829396, EPI_ISL_1829398, EPI_ISL_1829399, EPI_ISL_1829402, EPI_ISL_1829417, EPI_ISL_1829418, EPI_ISL_1829421, EPI_ISL_1829422, EPI_ISL_1829425, EPI_ISL_1829428, EPI_ISL_1829466, EPI_ISL_1829472, EPI_ISL_1829481, EPI_ISL_1829483, EPI_ISL_1829536, EPI_ISL_1829729, EPI_ISL_1829744, EPI_ISL_1829774, EPI_ISL_1830111, EPI_ISL_1830177, EPI_ISL_1830179, EPI_ISL_1830186, EPI_ISL_1830269, EPI_ISL_1830305, EPI_ISL_1830328, EPI_ISL_1830341, EPI_ISL_1830372, EPI_ISL_1830374, EPI_ISL_1830403, EPI_ISL_1830531, EPI_ISL_1830568, EPI_ISL_1830574, EPI_ISL_1830578, EPI_ISL_1830589, EPI_ISL_1830614, EPI_ISL_1830623, EPI_ISL_1830637, EPI_ISL_1830649, EPI_ISL_1830650, EPI_ISL_1830667, EPI_ISL_1830692, EPI_ISL_1830720, EPI_ISL_1831269, EPI_ISL_1831271, EPI_ISL_1831273, EPI_ISL_1831277, EPI_ISL_1831278, EPI_ISL_1831283, EPI_ISL_1857870, EPI_ISL_1857944, EPI_ISL_1858024, EPI_ISL_1858239, EPI_ISL_1912013, EPI_ISL_1912020, EPI_ISL_1912030, EPI_ISL_1912032, EPI_ISL_1912034, EPI_ISL_1912039, EPI_ISL_1912048, EPI_ISL_1912051, EPI_ISL_1912058, EPI_ISL_1912117, EPI_ISL_1912140, EPI_ISL_1912159, EPI_ISL_1912207, EPI_ISL_1912319, EPI_ISL_1921439, EPI_ISL_1921445, EPI_ISL_1921455, EPI_ISL_1921469, EPI_ISL_1921470, EPI_ISL_1921476, EPI_ISL_1921492, EPI_ISL_1921501, EPI_ISL_1921505, EPI_ISL_1921517, EPI_ISL_1921519, EPI_ISL_1921521, EPI_ISL_1921531, EPI_ISL_1921542, EPI_ISL_1921548, EPI_ISL_1921556, EPI_ISL_1921563, EPI_ISL_1921579, EPI_ISL_1921586, EPI_ISL_1921602, EPI_ISL_1921638, EPI_ISL_1921640, EPI_ISL_1921649, EPI_ISL_1921653, EPI_ISL_1921661, EPI_ISL_1921665, EPI_ISL_1921678, EPI_ISL_1921682, EPI_ISL_1921683, EPI_ISL_1921688, EPI_ISL_1921704, EPI_ISL_1921705, EPI_ISL_1921707, EPI_ISL_1921719, EPI_ISL_1921736, EPI_ISL_1939171, EPI_ISL_1939178, EPI_ISL_1985193, EPI_ISL_1985207, EPI_ISL_1985243, EPI_ISL_1985265, EPI_ISL_1985275, EPI_ISL_1985284, EPI_ISL_1985351, EPI_ISL_1985369, EPI_ISL_1985380, EPI_ISL_1985403, EPI_ISL_1985416, EPI_ISL_1985418, EPI_ISL_1985423, EPI_ISL_1985453, EPI_ISL_1985464, EPI_ISL_1985465, EPI_ISL_1985481, EPI_ISL_1985500, EPI_ISL_1985533, EPI_ISL_1985539, EPI_ISL_1985540, EPI_ISL_1985543, EPI_ISL_1985575, EPI_ISL_1985623,			

EPI_ISL_1985624, EPI_ISL_1985701, EPI_ISL_1985708, EPI_ISL_1985713, EPI_ISL_1985749, EPI_ISL_1985750, EPI_ISL_1985754, EPI_ISL_1985833, EPI_ISL_1985837, EPI_ISL_1985842, EPI_ISL_1985870, EPI_ISL_1985875, EPI_ISL_1985877, EPI_ISL_1985884, EPI_ISL_1985907, EPI_ISL_1985911, EPI_ISL_1985917, EPI_ISL_1985922, EPI_ISL_1985935, EPI_ISL_1985944, EPI_ISL_1985964, EPI_ISL_1985969, EPI_ISL_1985974, EPI_ISL_1985987, EPI_ISL_1985996, EPI_ISL_1985998, EPI_ISL_1986011, EPI_ISL_1986014, EPI_ISL_1986033, EPI_ISL_1986052, EPI_ISL_1986053, EPI_ISL_1986054, EPI_ISL_1986059, EPI_ISL_1986077, EPI_ISL_1986080, EPI_ISL_1986090, EPI_ISL_1986120, EPI_ISL_1986150, EPI_ISL_1986153, EPI_ISL_1986183, EPI_ISL_1986222, EPI_ISL_1986225, EPI_ISL_1986226, EPI_ISL_1986240, EPI_ISL_1986248, EPI_ISL_1986261, EPI_ISL_1986262, EPI_ISL_1986287, EPI_ISL_1986291, EPI_ISL_1986293, EPI_ISL_1986303, EPI_ISL_1986333, EPI_ISL_1986356, EPI_ISL_1986367, EPI_ISL_1986388, EPI_ISL_1986500, EPI_ISL_1986555, EPI_ISL_1986578, EPI_ISL_1986584, EPI_ISL_1986704, EPI_ISL_1986729, EPI_ISL_1986752, EPI_ISL_1986754, EPI_ISL_1986760, EPI_ISL_1986769, EPI_ISL_1986827, EPI_ISL_1986834, EPI_ISL_1986851, EPI_ISL_1986856, EPI_ISL_1986897, EPI_ISL_1986950, EPI_ISL_1986966, EPI_ISL_1986969, EPI_ISL_1987010, EPI_ISL_1987043, EPI_ISL_1987052, EPI_ISL_1987202, EPI_ISL_1987217, EPI_ISL_1987288, EPI_ISL_1987306, EPI_ISL_1987318, EPI_ISL_1987346, EPI_ISL_2004627, EPI_ISL_2004648, EPI_ISL_2004653, EPI_ISL_2004670, EPI_ISL_2004702, EPI_ISL_2004745, EPI_ISL_2004753, EPI_ISL_2004787, EPI_ISL_2004788, EPI_ISL_2004790, EPI_ISL_2004799, EPI_ISL_2004837, EPI_ISL_2004850, EPI_ISL_2004852, EPI_ISL_2005341, EPI_ISL_2005355, EPI_ISL_2005359, EPI_ISL_2005421, EPI_ISL_2005441, EPI_ISL_2005461, EPI_ISL_2005484, EPI_ISL_2005510, EPI_ISL_2005525, EPI_ISL_2005537, EPI_ISL_2005578, EPI_ISL_2005581, EPI_ISL_2005597, EPI_ISL_2005725, EPI_ISL_2005754, EPI_ISL_2005862, EPI_ISL_2006036, EPI_ISL_2006051, EPI_ISL_2006528, EPI_ISL_2006530, EPI_ISL_2006538, EPI_ISL_2021047, EPI_ISL_2021104, EPI_ISL_2021109, EPI_ISL_2021132, EPI_ISL_2021167, EPI_ISL_2021219, EPI_ISL_2021270, EPI_ISL_2021384, EPI_ISL_2021433, EPI_ISL_2021458, EPI_ISL_2021480, EPI_ISL_2021522, EPI_ISL_2021549, EPI_ISL_2021597, EPI_ISL_2021626, EPI_ISL_2021632, EPI_ISL_2021677, EPI_ISL_2021685, EPI_ISL_2021688, EPI_ISL_2021741, EPI_ISL_2021753, EPI_ISL_2021832, EPI_ISL_2021834, EPI_ISL_2021839, EPI_ISL_2021841, EPI_ISL_2021844, EPI_ISL_2021846, EPI_ISL_2021848, EPI_ISL_2021863, EPI_ISL_2022118, EPI_ISL_2022494, EPI_ISL_2022501, EPI_ISL_2080477, EPI_ISL_2091487, EPI_ISL_2091528, EPI_ISL_2091542, EPI_ISL_2091586, EPI_ISL_2091636, EPI_ISL_2091639, EPI_ISL_2091642, EPI_ISL_2091665, EPI_ISL_2091699, EPI_ISL_2091728, EPI_ISL_2091746, EPI_ISL_2091757, EPI_ISL_2091761, EPI_ISL_2091818, EPI_ISL_2117812, EPI_ISL_2119549, EPI_ISL_2121847, EPI_ISL_2137870, EPI_ISL_2137955, EPI_ISL_2138400, EPI_ISL_2172099, EPI_ISL_2172104, EPI_ISL_2172107, EPI_ISL_2172143, EPI_ISL_2172174, EPI_ISL_2172196, EPI_ISL_2172210, EPI_ISL_2172230, EPI_ISL_2172249, EPI_ISL_2172253, EPI_ISL_2172263, EPI_ISL_2172318, EPI_ISL_2172325, EPI_ISL_2172348, EPI_ISL_2172360, EPI_ISL_2172372, EPI_ISL_2199124, EPI_ISL_2199126, EPI_ISL_2199979, EPI_ISL_2199980, EPI_ISL_2236173, EPI_ISL_2236787, EPI_ISL_2237444, EPI_ISL_2237792, EPI_ISL_2237847, EPI_ISL_2238695, EPI_ISL_2253923, EPI_ISL_2275056, EPI_ISL_2275182, EPI_ISL_2296701, EPI_ISL_2316346, EPI_ISL_2316616, EPI_ISL_2348339, EPI_ISL_2352708, EPI_ISL_2366570, EPI_ISL_2392847, EPI_ISL_2393350, EPI_ISL_2393442, EPI_ISL_2395375, EPI_ISL_2395464, EPI_ISL_2395488, EPI_ISL_2395831, EPI_ISL_2407002, EPI_ISL_2407041, EPI_ISL_2446188, EPI_ISL_2446456, EPI_ISL_2446673, EPI_ISL_2456157, EPI_ISL_2456349, EPI_ISL_2456445, EPI_ISL_2456464, EPI_ISL_2468260, EPI_ISL_2468346, EPI_ISL_2511055, EPI_ISL_2511057, EPI_ISL_2511695, EPI_ISL_2511715, EPI_ISL_2511738, EPI_ISL_2511795, EPI_ISL_2512438, EPI_ISL_2512609, EPI_ISL_2512824, EPI_ISL_2512839, EPI_ISL_2513000, EPI_ISL_2513248, EPI_ISL_2514699, EPI_ISL_2537708, EPI_ISL_2537739, EPI_ISL_2539650, EPI_ISL_2539789, EPI_ISL_2539980, EPI_ISL_2540098, EPI_ISL_2540150, EPI_ISL_2540698, EPI_ISL_2540789, EPI_ISL_2540830, EPI_ISL_2540969, EPI_ISL_2541081, EPI_ISL_2541219, EPI_ISL_2553969, EPI_ISL_2568736, EPI_ISL_2568803, EPI_ISL_2569082, EPI_ISL_2569919, EPI_ISL_2606702, EPI_ISL_2606707, EPI_ISL_2607256, EPI_ISL_2608285, EPI_ISL_2608286, EPI_ISL_2638678, EPI_ISL_2638943, EPI_ISL_2640360, EPI_ISL_2641331, EPI_ISL_2641716, EPI_ISL_2641717, EPI_ISL_2641719, EPI_ISL_2664142, EPI_ISL_2664241, EPI_ISL_2665064, EPI_ISL_2665094, EPI_ISL_2665314, EPI_ISL_2665390, EPI_ISL_2665391, EPI_ISL_2665392, EPI_ISL_2665393, EPI_ISL_2681731, EPI_ISL_2681764, EPI_ISL_2699522, EPI_ISL_2699766, EPI_ISL_2699940, EPI_ISL_2700019, EPI_ISL_2700040, EPI_ISL_2700445, EPI_ISL_2701422, EPI_ISL_2701473, EPI_ISL_2701851, EPI_ISL_2702015, EPI_ISL_2702143, EPI_ISL_2702283, EPI_ISL_2703039, EPI_ISL_2703716, EPI_ISL_2717014, EPI_ISL_2717044, EPI_ISL_2717083, EPI_ISL_2717233, EPI_ISL_2717243, EPI_ISL_2717473, EPI_ISL_2728778, EPI_ISL_2729255, EPI_ISL_2729640, EPI_ISL_2729998, EPI_ISL_2730398, EPI_ISL_2767532, EPI_ISL_2767774, EPI_ISL_2767802, EPI_ISL_2767975, EPI_ISL_2767997, EPI_ISL_2779792, EPI_ISL_2779866, EPI_ISL_2780310, EPI_ISL_2780537, EPI_ISL_2780733, EPI_ISL_2780883, EPI_ISL_2780937, EPI_ISL_2781324, EPI_ISL_2781702			
see above	Lighthouse Lab in Milton Keynes	Wellcome Sanger Institute for the COVID-19 Genomics UK (COG-UK) Consortium	Cordelia Langford; David K. Jackson; Dominic Kwiatkowski; Ewan Harrison; Ian Johnston; Jeffrey Barrett; John Sillitoe on behalf of the Wellcome Sanger Institute COVID-19 Surveillance Team; Roberto Amato; Sonia Goncalves; The Lighthouse Lab in Milton Keynes and Alex Alderton
EPI_ISL_2353258, EPI_ISL_2393328, EPI_ISL_2447704, EPI_ISL_2447716, EPI_ISL_2468032, EPI_ISL_2541563, EPI_ISL_2541592, EPI_ISL_2568916, EPI_ISL_2568956, EPI_ISL_2570328, EPI_ISL_2570684, EPI_ISL_2606498, EPI_ISL_2606723, EPI_ISL_2606950, EPI_ISL_2638431, EPI_ISL_2638533, EPI_ISL_2639232, EPI_ISL_2703837, EPI_ISL_2703904, EPI_ISL_2703923, EPI_ISL_2703966, EPI_ISL_2704052, EPI_ISL_2718386, EPI_ISL_2730093, EPI_ISL_2766418, EPI_ISL_2766480, EPI_ISL_2766679, EPI_ISL_2767506, EPI_ISL_2780006, EPI_ISL_2780354			
see above	Lighthouse Laboratory Plymouth	Wellcome Sanger Institute for the COVID-19 Genomics UK (COG-UK) Consortium	Cordelia Langford; David K. Jackson; Dominic Kwiatkowski; Ewan Harrison; Ian Johnston; Jeffrey Barrett; John Sillitoe on behalf of the Wellcome Sanger Institute COVID-19 Surveillance Team; Lighthouse Laboratory Plymouth and Alex Alderton; Roberto Amato; Sonia Goncalves
EPI_ISL_1727471, EPI_ISL_2389214	Limbach - MVZ Humangenetik Ulm	Robert Koch Institute	
EPI_ISL_1833022, EPI_ISL_2240447	Lincolnshire Hospitals and DeepSeq Nottingham	COVID-19 Genomics UK (COG-UK) Consortium	Christopher Moore; Fei Sang; Joeseph Chappell; Johnny Debebe; Jonathan Ball; Matthew Carlisle; Matthew Loose; Nadine Holmes; Nichola Duckworth; Patrick McClure; Sarah Walsh; Tim Sloan; Victoria Wright
EPI_ISL_636847, EPI_ISL_636880	Lithuanian University of Health Sciences Hospital, Department of Laboratory Medicine	Lithuanian University of Health Sciences, Molecular cardiology lab.	Arnoldas Pautienius; Astra Vitkauskiene; Dovydas Gecys; Ingrida Olendraitė; Kamile Tamusauskaite; Laura Pareckaite; Lukas Zemaitis; Vaiva Lesauskaite
EPI_ISL_1636862, EPI_ISL_1975018, EPI_ISL_2238875, EPI_ISL_2238876	Liverpool Clinical Laboratories	COVID-19 Genomics UK (COG-UK) Consortium	A Alrezaihi; Alessandro Gerada; Alistair Darby; Angela Cowell; Anita Lucaci; Anu Chawla; Cassie Olateju; Catherine Hartley; Charlotte Nelson; Ecaterina Vamos; Elaine O'Toole; Eleanor G Bentley; Ghada T Shawli; Hermione Webster; Isabel García-Dorival; James Johnson; James P Stewart; Jenifer Manson; Joanne Watts; Jones Benjamin; Jordan J Clark; Julian Hiscox; L Luu; Lucille Rainbow; M Almsaud; Margaret Hughes; Mark Whitehead; Matthew Gemmell; Miren Iturriza-Gomara; Muhannad Alruwaili; N.P Randle; Neil Swainston; PKF Gilmore; Parul Sharma; Rebekah Penrice-Randal; Richard Eccles; Richard Gregory; Sam Haldenby; Steve Paterson; Stuart D Armstrong; Trevor Ian Robinson; Ximeng Han
EPI_ISL_1827699, EPI_ISL_1827701, EPI_ISL_1827703	Lobamba	National Institute for Communicable Diseases of the National Health Laboratory Service	Amoako DG; Bhiman JN; Ismail A; Mahlangu B; Maphalala GP; Mohale T; Ntuli N; Scheepers C
EPI_ISL_1594158, EPI_ISL_2495509, EPI_ISL_2495627	MB-Cadham Provincial laboratory	National Microbiology Laboratory (NML)	Anna Majer; Anneliese Landgraff; CanCOGeN's metadata curation team; Darian Hole; David Alexander; Elsie Grudeski; Gary Van Domselaar; Grace Seo; Jared Bullard; Jennifer Tanner; Kerry Dust; Kirsten Biggar; Madison Chapel; Morag Graham; Natalie Knox; Nathalie Bastien; Paul Van Caeseele; Philip Mabon; Public Health Agency of Canada CanCOGeN team; Rhiannon Huzarewich; Russell Mandes; Shari Tyson; Timothy Booth; Yan Li
EPI_ISL_644555, EPI_ISL_2036369, EPI_ISL_2036390, EPI_ISL_2272600	MEPHI, Aix Marseille University	MEPHI, Aix Marseille University	Anthony LEVASSEUR
EPI_ISL_2342613, EPI_ISL_2342616, EPI_ISL_2342619, EPI_ISL_2342624, EPI_ISL_2342626, EPI_ISL_2342628, EPI_ISL_2342629, EPI_ISL_2342631, EPI_ISL_2342637, EPI_ISL_2342646, EPI_ISL_2342648, EPI_ISL_2342653, EPI_ISL_2342658, EPI_ISL_2342662, EPI_ISL_2342663, EPI_ISL_2342668, EPI_ISL_2342677, EPI_ISL_2342688, EPI_ISL_2342701, EPI_ISL_2342704, EPI_ISL_2342705, EPI_ISL_2342712, EPI_ISL_2342720, EPI_ISL_2342838, EPI_ISL_2342971, EPI_ISL_2342972, EPI_ISL_2342973, EPI_ISL_2342974, EPI_ISL_2342975, EPI_ISL_2342976, EPI_ISL_2342977, EPI_ISL_2342978, EPI_ISL_2342979			
see above	MGM Medical College & Hospital, Jamshedpur	Institute of Life Sciences - INSACOG	Ajay Parida; Amol M. Kanampalliwar; Arup Ghosh; Atimukta Jha; INSACOG Consortium; Omprakash Shiriwas; Punit Prasad; Rajeeb Swain; Rupesh Dash; Safal Walia; Sana Fatma; Shifu Aggarwal; Sunil K. Raghav
EPI_ISL_1663541, EPI_ISL_1663543, EPI_ISL_1663548, EPI_ISL_1663549	MGM Medical College, Jamshedpur	Institute of Life Sciences - INSACOG	Ajay Parida; Amol M. Kanampalliwar; Arup Ghosh; Atimukta Jha; INSACOG Consortium; Punit Prasad; Rajeeb Swain; Rupesh Dash; Safal Walia; Shifu Aggarwal; Sunil K. Raghav
EPI_ISL_2016024	MIRIALIS CLUSES BECHET	CNR Virus des Infections Respiratoires - France SUD	Antonin Bal; Bruno Lina; Gregory Destras; Gwendolyne Burfin; Hadrien Regue; Laurence Josset; Martine Valette; Quentin Semanas
EPI_ISL_903382, EPI_ISL_2556117, EPI_ISL_2557251, EPI_ISL_2557459	MOH - Jaber Al-Ahmad Hospital (Innovation Research Laboratory)	MOH - Jaber Al-Ahmad Hospital (Innovation Research Laboratory)	Mohammad Alghounaim; Salman Al-Sabah
EPI_ISL_471161, EPI_ISL_471164, EPI_ISL_561113, EPI_ISL_561174, EPI_ISL_561213, EPI_ISL_810979, EPI_ISL_810982, EPI_ISL_810986, EPI_ISL_811037, EPI_ISL_1216079, EPI_ISL_1216118, EPI_ISL_1234530, EPI_ISL_1234531, EPI_ISL_1234533, EPI_ISL_1620175, EPI_ISL_1620188, EPI_ISL_1620194, EPI_ISL_1731542, EPI_ISL_1731547, EPI_ISL_1731550, EPI_ISL_1731566			
see above	MRCG at LSHTM Genomics lab	MRCG at LSHTM Genomics lab	Abdoulie Kanteh; Abdul Karim sesay; Bakary Sanyang; Jarra Manneh; Mariama Kujabi; Sesay et al
EPI_ISL_428855	MRCG at LSHTM Geomics lab	MRCG at LSHTM Genomics lab	Sesay et al
EPI_ISL_2111238	MVZ Labor Dr. Fenner und Kollegen (Standort Hamburg)	Robert Koch Institute	
EPI_ISL_1847444, EPI_ISL_1852081, EPI_ISL_1852099, EPI_ISL_1852126, EPI_ISL_2260917, EPI_ISL_2313176	MVZ Labor Dr. Limbach & Kollegen GbR	Robert Koch Institute	
EPI_ISL_2261471	MVZ Labor Dr. Quade & Kollegen GmbH	Robert Koch Institute	
EPI_ISL_1641213, EPI_ISL_1851102, EPI_ISL_2111185, EPI_ISL_2115263, EPI_ISL_2115271	MVZ Labor Krone GbR	Robert Koch Institute	
EPI_ISL_1850317, EPI_ISL_1850326	MVZ Medizinisches Labor Hannover GmbH	Robert Koch Institute	
EPI_ISL_2342046, EPI_ISL_2342049, EPI_ISL_2342051, EPI_ISL_2342063, EPI_ISL_2342069, EPI_ISL_2342075, EPI_ISL_2342272, EPI_ISL_2342293, EPI_ISL_2342294, EPI_ISL_2342295, EPI_ISL_2342296, EPI_ISL_2342297			
see above	Mahatma Gandhi Memorial Medical College & Hospital Jamshedpur	Institute of Life Sciences - INSACOG	Ajay Parida; Amol M. Kanampalliwar; Arup Ghosh; Atimukta Jha; INSACOG Consortium; Omprakash Shiriwas; Punit Prasad; Rajeeb Swain; Rupesh Dash; Safal Walia; Sana Fatma; Shifu Aggarwal; Sunil K. Raghav
EPI_ISL_1936105, EPI_ISL_1936115, EPI_ISL_1936126, EPI_ISL_1936140, EPI_ISL_1936143, EPI_ISL_1936195, EPI_ISL_1936209, EPI_ISL_1936218, EPI_ISL_1936228, EPI_ISL_1936246, EPI_ISL_1936248, EPI_ISL_1936251, EPI_ISL_1936262, EPI_ISL_1936274, EPI_ISL_1936288, EPI_ISL_1936291			
see above	Main Chemical Laboratories Egypt Army	Main Chemical Laboratories Egypt Army	Abdullah Salama; AbedElrahman Zekri; Ahmed Gad; Bassem Elharty; Mervat Hassan; Mohamed Abdel-Monem; Mohamed El-Esawi; Mohamed Seadawy; Mohamed Shamel; Mostfa Elhoseiny; Sabah Ahmed
EPI_ISL_2360253	Maku Molecular Laboratory	National Influenza Center	A Nejati; F Ajaminejad and T Mokhtari Azad; J Yavarian; K Sadeghi; N Ghavvami; NZ Shafiei Jandaghi; V Salimi
EPI_ISL_1827698, EPI_ISL_1827700, EPI_ISL_1827704	Manzana	National Institute for Communicable Diseases of the National Health Laboratory Service	Amoako DG; Bhiman JN; Ismail A; Mahlangu B; Maphalala GP; Mohale T; Ntuli N; Scheepers C
EPI_ISL_2373628	Mapmygenome	CSIR-Centre for Cellular and Molecular Biology - INSACOG	Amareshwar Vodapalli; Ara Sreenivas; B Himasri; Divya Tej Sowpati; Karthik Bharadwaj Tallapaka; Lamuk Zaveri; Onkar Kulkarni; Rakesh K Mishra; Sharath Chandra Thota; Shreekant Verma; Sofia Banu; Viswagithe S L
EPI_ISL_2156920, EPI_ISL_2156932, EPI_ISL_2157019, EPI_ISL_2157021, EPI_ISL_2157022, EPI_ISL_2373320, EPI_ISL_2373323, EPI_ISL_2373331, EPI_ISL_2373340, EPI_ISL_2441344, EPI_ISL_2441355, EPI_ISL_2441358, EPI_ISL_2441365, EPI_ISL_2441366, EPI_ISL_2774658, EPI_ISL_2774666			
see above	Mapmygenome	CSIR-Centre for Cellular and Molecular Biology-INSACOG	; Amareshwar Vodapalli; Ara Sreenivas; Archana Bharadwaj Siva; B Himasri; Blessy B John; Divya Tej Sowpati; Jandhyala Sai Krishna; Karthik Bharadwaj Tallapaka; Lamuk Zaveri; Onkar Kulkarni; Payel Mukherjee; Priya Nurkurthy; Rakesh K Mishra; Sharath Chandra Thota; Shreekant Verma; Sofia Banu; Sumedha Avadhanula; Tulasi Nagabandi; Valli Nagalakshmi Undamatla; Vidhyadhari Methuku; Viswagithe S L
EPI_ISL_1910606, EPI_ISL_1910607, EPI_ISL_1910617, EPI_ISL_1913025	Mashrek Medical Diagnostic Center	Microbial Pathogenomics Lab - LAU	Georgi Merhi; Jad Koweyes; Sima Tokajian; Tamara Salloum
EPI_ISL_2006677, EPI_ISL_2095076, EPI_ISL_2095274, EPI_ISL_2450280	Max von Pettenkofer Institute, Virology, National Reference Center for Retroviruses, LMU Munich	Laboratory for Functional Genome Analysis; Dept. Genomics; Gene Center of the LMU Munich	Alexander Graf; Helmut Blum; Max Muenchhoff; Oliver Keppler; Stefan Krebs
EPI_ISL_1827706, EPI_ISL_1827707	Mbabane Public Health	National Institute for Communicable Diseases of the National Health Laboratory Service	Amoako DG; Bhiman JN; Ismail A; Mahlangu B; Maphalala GP; Mohale T; Ntuli N; Scheepers C
EPI_ISL_2234383	Medical Biology Department, Tokat Gaziosmanpasa University	Medical Biology Department, Tokat Gaziosmanpasa University	Al-Attar; B.O.; Ibrahim; Kanabe; Khailany; M.O.; M.S. and Ozaslan, M.; O.Q.; R.A.; Rahman

EPI_ISL_1347416, EPI_ISL_2380495	Medical Laboratories Duesseldorf	Center of Medical Microbiology, Virology, and Hospital Hygiene, University of Duesseldorf	Alexander Dilthey; Andreas Walker; Angelika Helmer; Christian Lange; Daniel Strelow; Jessica Nicolai; Jörg Timm; Klaus Pfeffer; Lisanna Hülse; Malte Kohns Vasconcelos; Maximilian Damagnez; Nadine Lübke; Tobias Wienemann; Torsten Houwaart
EPI_ISL_1302579	Medicina practica laboratorija	Lithuanian University of Health Sciences Hospital, Department of Genetics and Molecular Medicine	Astra Vitkauskiene; Darius Cereskevicius; Inga Nasvytiene; Mantas Sarauskas; Marius Sukys; Rasa Ugenskiene; Renaldas Jurkevicius; Zivile Zemeckiene
EPI_ISL_2471770, EPI_ISL_2762825	Medizinisch-Diagnostisches Labor Kempten allgäulab	Robert Koch Institute	
EPI_ISL_2116115	Medizinische Laboratorien Dusseldorf	Robert Koch Institute	
EPI_ISL_2348499	Megalab, Molecular and Cytogenetics Diagnostics	Department for Virology, Molecular Biology and Genome Research, R. G. Lugar Center for Public Health Research, National Center for Disease Control and Public Health (NCDC) of Georgia.	Adam Kotorashvili; Amiran Gamkrelidze.; Ana Papkiauri; Ann Machablishvili; Anna Kasradze; Davit Tsaguria; Ekaterine Khmaladze; Ekaterine Zangaladze; Ekaterine Zhghenti; Giorgi Gogoladze; Giorgi Tomashvili; Gvantsa Brachveli; Gvantsa Chanturia; Irma Burjanadze; Ketevan Sidamonidze; Khatuna Zakhashvili; Lela Sabadze; Lela Urushadze; Magda Dgebuadze; Maia Alkhazashvili; Mari Gavashelidze; Mariam Zakalashvili; Marine Murtskhvaladze; Meri Pantsulaia; Nato Kotaria; Nino Berishvili; Nino Chikhovani; Paata Imnadze; Roena Sukhiashvili; Tamar Jashiashvili; Tata Imnadze; Tea Tevdoradze
EPI_ISL_562729, EPI_ISL_564958, EPI_ISL_1249986, EPI_ISL_1249990, EPI_ISL_1249999, EPI_ISL_1250002, EPI_ISL_1250005, EPI_ISL_1913109, EPI_ISL_1913110, EPI_ISL_1913174, EPI_ISL_2379270, EPI_ISL_2379278, EPI_ISL_2379289, EPI_ISL_2379290, EPI_ISL_2379291, EPI_ISL_2405346, EPI_ISL_2405349, EPI_ISL_2405350, EPI_ISL_2405353, EPI_ISL_2482436, EPI_ISL_2482442, EPI_ISL_2482447, EPI_ISL_2482451, EPI_ISL_2482454, EPI_ISL_2482459, EPI_ISL_2482460			
see above	Microbiological Diagnostic Unit - Public Health Laboratory (MDU-PHL)	MDU-PHL	M.L.; N.L.; Sait; Sait, M.; Schultz M. B.; Seemann T.; Seemann, T.; Sherry; Sherry, N.
EPI_ISL_2621569, EPI_ISL_2621572	Microbiological Diagnostic Unit - Public Health Laboratory (MDU-PHL)	Microbiological Diagnostic Unit - Public Health Laboratory (MDU-PHL)	M.L.; N.L.; Sait; Seemann T.; Sherry
EPI_ISL_2250223, EPI_ISL_2250224, EPI_ISL_2250225, EPI_ISL_2250232	Microbiological Diagnostic Unit - Public Health Laboratory (MDU-PHL)	Microbiological Diagnostic Unit Public Health Laboratory (MDU-PHL)	M.L.; N.L.; Sait; Seemann T.; Sherry
EPI_ISL_427077	Microbiological Diagnostic Unit Public Health Laboratory	Microbiological Diagnostic Unit Public Health Laboratory	Sait, M.; Schultz M.; Seemann T.; Sherry, N.
EPI_ISL_2131838, EPI_ISL_2132223	Microbiology Department, Lu`an Center for Disease Control and Prevention	Microbiology Department, Lu`an Center for Disease Control and Prevention	Chang Hongwei; Chen Beilei; Chen Zhichao; Fan Yuzhen.; Gao Dawei; Li Zhaoyang; Yang Wei; Zhang Feng; Zhang Limei; Zhang Qin; Zhu Rui
EPI_ISL_2362674, EPI_ISL_2362675, EPI_ISL_2362678, EPI_ISL_2362680, EPI_ISL_2362683, EPI_ISL_2362684, EPI_ISL_2362686			
see above	Microbiology and Virology Unit, Florence Careggi University Hospital	Microbiology and Virology Unit, Florence Careggi University Hospital	Alberto Antonelli; Emanuele Gori; Fabio Morecchiato; Gian Maria Rossolini; Ilaria Baccani; Marco Coppi; Nicla Giovacchini; Noemi Aiezza; Vincenzo Di Pilato
EPI_ISL_1181801	Microbiology and Virology Unit,Azienda Ospedale Padova,Padova,Italy	Department of Molecular Medicine,Computational Medicine Group,Univeresity of Padova,Padova,Italy	Andrea Crisanti; Claudia Del Vecchio; Elisa Franchin; Enrico Lavezzo; Federico Bianca; Francesco Onelia; Laura Manuto; Marco Grazioli; Stefano Toppo
EPI_ISL_2348487	Middle East Institute of Health University Hospital	Microbial Pathogenomics Lab - LAU	Edmond Abboud; Georgi Merhi; Jad Koweyes; Sima Tokajian
EPI_ISL_2603843	Ministry of Health, Jaber Al-Ahmad Hospital	Virology Unit, Department of Microbiology, Faculty of Medicine, Kuwait University	Anfal Al-Adwani; Ebaa Al-Awadhi; Hussain Safar; Nada Madi
EPI_ISL_1634442	Mitra Kasih Hospital	West Java Health Laboratory; School of Life Sciences and Technology, Institut Teknologi Bandung	Aulia Saraswati Wicaksono; Azzania Fibriani; Cut Nur Cinthia Alamanda; Ema Rahmawati; Karimatu Khoirunnisa; Miftahul Faridl; Rifky Waluyajati Rachman; Rini Robiani; Ryan Bayusantika Ristandi
EPI_ISL_2101098	Molecular Biology Laboratory, Faculty Medicine and Health Sciences, Warmadewa University	Eijkman Institute for Molecular Biology, National Agency for Research and Innovation; Molecular Biology Laboratory, Faculty Medicine and Health Sciences, Warmadewa University	Amin Soebandrio; Edison Johar; Erly Sintya; Frilasita A Yudhaputri; Hidayat Trimarsanto; Iskandar Adnan; Khin Saw Myint; Lidwina Priliani; Lydia V. Panggalo; Muhammad Rezki Rasyak; Safarina G Malik; Sri Masyeni; Sukma Oktavianthi; Willy Agustine
EPI_ISL_1993548	Molecular Diagnostic Laboratory (Hormozghan University of Medical Sciences)	National Influenza Center	A Nejati; F Ajaminejad and T Mokhtari Azad; J Yavarian; K Sadeghi; N Ghavvami; NZ Shafiei Jandaghi; V Salimi
EPI_ISL_2350812	Molecular Lab, Evercare hospital Dhaka	International Institute for Zoonosis Control, Hokkaido university	Junya Yamagishi; Mizanur Rahman
EPI_ISL_895786	Molecular biology division, Institute of Clinical Biochemistry and Diagnostics, Charles University, Faculty of Medicine in Hradec Králové and University Hospital Hradec Králové	Molecular biology division, Institute of Clinical Biochemistry and Diagnostics, Charles University, Faculty of Medicine in Hradec Králové and University Hospital Hradec Králové	Helena Kovaříková; Ivana Baranová; Jitka Novotná; Kateřina Hrochová; Kateřina Pehlíková; Petr Brož; Tereza Baťková; Vladimír Palička. Cooperation project with BioVendor-R&D and bioinformatics company BIOXSYS s r.o.
EPI_ISL_1367682, EPI_ISL_1367691, EPI_ISL_1367694, EPI_ISL_1663657, EPI_ISL_1663659, EPI_ISL_1663666, EPI_ISL_1663668, EPI_ISL_1663671, EPI_ISL_1663674, EPI_ISL_1663677, EPI_ISL_1663679			
see above	Molecular diagnostic unit for viral haemorrhagic fevers and emerging viruses, Bouaké CHU Laboratory	Molecular diagnostic unit for viral haemorrhagic fevers and emerging viruses, Bouaké CHU Laboratory	Adjaratou Traoré; Bamba Fatoumata Touré; Chantal Akoua-Koffi; Coulibaly Mbegnan; Diané Bamourou; Essia Belarbi; Etilé Anoh; Fabian Leendertz; Grit Schubert; Kra Ouffoué; Monemo Pacome; Oby Wayoro; Safiatou Karidioula; Soundélé Maïté
EPI_ISL_614347, EPI_ISL_614394, EPI_ISL_681842, EPI_ISL_1662592	Molecular diagnostic unit for viral haemorrhagic fevers and emerging viruses, Bouaké CHU Laboratory	Project group Epidemology of Highly Pathogenic Microorganisms, Robert Koch-Institute	Adjaratou Traoré; Bamba Fatoumata Touré; Chantal Akoua-Koffi; Coulibaly Mbegnan; Diané Bamourou; Essia Belarbi; Etilé Anoh; Fabian Leendertz; Grit Schubert; Kra Ouffoué; Monemo Pacome; Safiatou Karidioula; Soundélé Maïté
EPI_ISL_1865437	Molekylær Medicinsk Afdeling, Aarhus University Hospital, Aarhus, Denmark	Aalborg University	Danish Covid-19 Genome Consortium
EPI_ISL_2426098, EPI_ISL_2429125	Mubarak Al-Kabeer Hospital	Virology Unit, Department of Microbiology, Faculty of Medicine, Kuwait University	Anfal Al-Adwani; Hussain Safar; Nada Madi
EPI_ISL_2429129	Mubaral Al-Kabeer Hospital	Virology Unit, Department of Microbiology, Faculty of Medicine, Kuwait University	Anfal Al-Adwani; Hussain Safar; Nada Madi
EPI_ISL_2017761, EPI_ISL_2017762, EPI_ISL_2391477, EPI_ISL_2391478, EPI_ISL_2391481, EPI_ISL_2391482, EPI_ISL_2391483, EPI_ISL_2391484, EPI_ISL_2391486, EPI_ISL_2391487, EPI_ISL_2405118, EPI_ISL_2405119, EPI_ISL_2405120, EPI_ISL_2405121, EPI_ISL_2405122, EPI_ISL_2405123, EPI_ISL_2509934, EPI_ISL_2509935, EPI_ISL_2509936, EPI_ISL_2509937, EPI_ISL_2509939, EPI_ISL_2509940, EPI_ISL_2509941, EPI_ISL_2509942, EPI_ISL_2509943, EPI_ISL_2509944, EPI_ISL_2509945, EPI_ISL_2509946, EPI_ISL_2509949, EPI_ISL_2509950, EPI_ISL_2509951, EPI_ISL_2509952, EPI_ISL_2509953, EPI_ISL_2509954, EPI_ISL_2509955, EPI_ISL_2509957, EPI_ISL_2509958, EPI_ISL_2543891, EPI_ISL_2543892, EPI_ISL_2543893, EPI_ISL_2543894, EPI_ISL_2543895, EPI_ISL_2543896, EPI_ISL_2543897, EPI_ISL_2543898			
see above	N.H.L Municipal Medical College, Ahmedabad	Gujarat Biotechnology Research Centre	Chaitanya Joshi; Dinesh Kumar; Janvi Raval; Jayshri Pethani; Madhvi Joshi; Nitesh Shah; Nitin Savaliya; Ramesh Pandit; Sonal Sharma; Twinkle Soni; Umang Mishra; Zarna Patel; Zuber Saiyed
EPI_ISL_1588070, EPI_ISL_1588074, EPI_ISL_1588081, EPI_ISL_1588264, EPI_ISL_1588269, EPI_ISL_2550561, EPI_ISL_2550569, EPI_ISL_2550589, EPI_ISL_2550605			
see above	NB-Hôpital Georges L. Dumont	National Microbiology Laboratory (NML)	Anna Majer; Anneliese Landgraff; CanCOGeN's metadata curation team; Darian Hole; Elsie Grudeski; Gary Van Domselaar; Grace Seo; Guillaume Desnoyers; Jennifer Tanner; Kirsten Biggar; Madison Chapel; Morag Graham; Natalie Knox; Nathalie Bastien; Philip Mabon; Public Health Agency of Canada CanCOGeN team; Rhiannon Huzarewich; Richard Garceau; Russell Mandes; Shari Tyson; Timothy Booth; Yan Li
EPI_ISL_1663366, EPI_ISL_1663367, EPI_ISL_1663375, EPI_ISL_1663376, EPI_ISL_2341673, EPI_ISL_2341791, EPI_ISL_2342630, EPI_ISL_2342642, EPI_ISL_2342644, EPI_ISL_2342656, EPI_ISL_2342722, EPI_ISL_2342919, EPI_ISL_2342930, EPI_ISL_2342931, EPI_ISL_2342932, EPI_ISL_2342937, EPI_ISL_2342938, EPI_ISL_2342946, EPI_ISL_2342947, EPI_ISL_2342953, EPI_ISL_2342956, EPI_ISL_2342964			
see above	NCCS, Pune	Institute of Life Sciences - INSACOG	Ajay Parida; Amol M. Kanampalliwar; Arup Ghosh; Atimukta Jha; INSACOG Consortium; Omprakash Shiriwas; Punit Prasad; Rajeeb Swain; Rupesh Dash; Safal Walia; Sana Fatma; Shifu Aggarwal; Sunil K. Raghav
EPI_ISL_854982	NGS Lab, DNA SOLUTION LTD.	NGS Lab, DNA SOLUTION LTD.	Chowdhury, M.; H.U.; Haider; Hasan; Hosen; K.N.; Khaleque, A.; Khan; Khan, M.; M.B.; M.F.A.; M.H.; M.I.; Rabbi; Rahman, M.; Razu; Sufian, A.
EPI_ISL_420037	NIC Viral Respiratory Unit - Institut Pasteur of Algeria	National Reference Center for Viruses of Respiratory Infections, Institut Pasteur, Paris	Angela Brisebarre; Etienne Simon-Lorière; Fawzi Derrar; Flora Donati; Marion Barbet; Maud Vanpeene; Mélanie Albert; Méline Bizard; Sylvie Behillil; Sylvie van der Werf; Vincent Enouf
EPI_ISL_2379357, EPI_ISL_2450328, EPI_ISL_2450329, EPI_ISL_2450334, EPI_ISL_2450339, EPI_ISL_2728444, EPI_ISL_2728455, EPI_ISL_2728456, EPI_ISL_2728480, EPI_ISL_2728484, EPI_ISL_2728485, EPI_ISL_2728486, EPI_ISL_2728495, EPI_ISL_2728496, EPI_ISL_2728500, EPI_ISL_2728504, EPI_ISL_2728505, EPI_ISL_2728507, EPI_ISL_2728509, EPI_ISL_2728510, EPI_ISL_2728511, EPI_ISL_2728512, EPI_ISL_2728513, EPI_ISL_2728514, EPI_ISL_2728515, EPI_ISL_2728516, EPI_ISL_2728517, EPI_ISL_2728518, EPI_ISL_2728519, EPI_ISL_2728520, EPI_ISL_2728521, EPI_ISL_2728522			
see above	NIMHANS	INSACOG-KA, NIMHANS	Ananthapadmanabha Kotambail; Anita S Desai; Anson Kunjumon George; Chetan G K; Chitra Pattabiraman; Darshan Sreenivas; Ellango Ramasamy; Gautham Arunachal Udupi; Mahesh Kumar.C.S; Pramada Prasad; Sony Sharma; V Ravi
EPI_ISL_1939986, EPI_ISL_1939987, EPI_ISL_1939999, EPI_ISL_1940055, EPI_ISL_1940057, EPI_ISL_1940095, EPI_ISL_1940096, EPI_ISL_1940097, EPI_ISL_1940099, EPI_ISL_1940107, EPI_ISL_1940108, EPI_ISL_1940133, EPI_ISL_1940134			
see above	NIMHANS	inStem NCBS - INSACOG	Uma Ramakrishnan Dasaradhi Palakodeti Aswin SaiNarain
EPI_ISL_1278177, EPI_ISL_1278185, EPI_ISL_1587827, EPI_ISL_1587878, EPI_ISL_1587899, EPI_ISL_1587903, EPI_ISL_2161784, EPI_ISL_2161797, EPI_ISL_2484596, EPI_ISL_2484610, EPI_ISL_2484613, EPI_ISL_2484620, EPI_ISL_2484640, EPI_ISL_2484644			
see above	NL-Dr. Leonard A. Miller Centre for Health Services	National Microbiology Laboratory (NML)	Adel Malek; Anna Majer; Anneliese Landgraff; CanCOGeN's metadata curation team; Darian Hole; Elsie Grudeski; Gary Van Domselaar; George Zahariadis; Grace Seo; Jennifer Tanner; Kerri Smith; Kirsten Biggar; Laura Gilbert; Madison Chapel; Morag Graham; Natalie Knox; Nathalie Bastien; Philip Mabon; Public Health Agency of Canada CanCOGeN team; Rhiannon Huzarewich; Robert Needle; Russell Mandes; Shari Tyson; Timothy Booth; Yan Li; Yang Yu
EPI_ISL_1579578	NMVRVI	National Public Health Surveillance Laboratory	Ana Steponkiene; Danas Baksa; Jelena Razmuk; Lukas Vasionis; Lukas Zemaitis; Migle Gabrielaite; Svajune Muralyte
EPI_ISL_1666863, EPI_ISL_1666941, EPI_ISL_2162072, EPI_ISL_2162120	NS-QEII Health Sciences Centre	National Microbiology Laboratory (NML)	Anna Majer; Anneliese Landgraff; CanCOGeN's metadata curation team; Dan Gaston; Darian Hole; Elsie Grudeski; Gary Van Domselaar; Grace Seo; Janice Pettipas; Jason LeBlanc; Jennifer Tanner; Kirsten Biggar; Madison Chapel; Morag Graham; Natalie Knox; Nathalie Bastien; Philip Mabon; Public Health Agency of Canada CanCOGeN team; Rhiannon Huzarewich; Russell Mandes; Shari Tyson; Timothy Booth; Todd Hatchette; Yan Li
EPI_ISL_2445121	NUCLEO DE ESPECIALIDADES EM SAUDE	Instituto Butantan	Antonio Jorge Martins; Claudia Renata dos Santos Barros; David Schlesinger; Debora Botequio Moretti; Dimas Tadeu Covas; Elaine Cristina Marqueze; Elaine Vieira Santos; Evandra Strazza Rodrigues; Heidge Fukumasu; Jayme Augusto de Souza-Neto; José Salvatore Leister Patané; Luiz Alcantara; Luiz Lehmann Coutinho; Maria Carolina Elias; Maurício Lacerda Nogueira; Rafael dos Santos Bezerra; Raul Machado Neto; Rejane Maria Tommasini Grotto; Ricardo Haddad; Sandra Coccuzzo Sampaio Vessoni; Simone Kashima; Svetoslav Nanev Slavov; Vincent Louis Viala
EPI_ISL_1654649	NVMRVI	National Public Health Surveillance Laboratory	Ana Steponkiene; Danas Baksa; Jelena Razmuk; Lukas Vasionis; Lukas Zemaitis; Migle Gabrielaite; Svajune Muralyte
EPI_ISL_1579921	NVSPL	National Public Health Surveillance Laboratory	Ana Steponkiene; Danas Baksa; Jelena Razmuk; Lukas Vasionis; Lukas Zemaitis; Migle Gabrielaite; Svajune Muralyte
EPI_ISL_1718282, EPI_ISL_1718287, EPI_ISL_1718291, EPI_ISL_1718299, EPI_ISL_1718303, EPI_ISL_1718304	National Center of Disease Control and Prevention of the Republic of Armenia	Institute of Molecular Biology NAS RA, Republic of Armenia, Department of Bioengineering, BioinformaticsInstitute and Molecular Biology IBMPh RAU, Republic of Armenia	Andranik Chavushyan; Arsen Arakelyan; Diana Avetyan; Gayane Melik-Pashayan; Gisane Khachatyan; Hovsep Ghazaryan; Maria Nikoghosyan; Nelli Muradyan; Roksana Zakharyan; Shushan Sargrsyan; Siras Hakobyan; Tamara Sirunyan; Zaven Karalyan

EPI_ISL_1854605, EPI_ISL_1854627, EPI_ISL_1854632, EPI_ISL_1854633, EPI_ISL_1854634, EPI_ISL_1854639	National Center of Disease Control and Prevention of the Republic of Armenia	UW Virology Lab	Alexander Greninger; Anahit Hovhannisyan; Andranik Chavushyan; Anna Khazaryan; Arsen Arakelyan; Diana Avetyan; Gisane Khachatyan; Hong Xie; Hovsep Ghazaryan; Keith R. Jerome; Lasata Shrestha; Lyudmila Niazyan; Maria Nikoghosyan; Meei-Li Huang; Michelle Lin; Nelli Muradyan; Pavitra Roychoudhury; Roksana Zakharyan; Shah Mohamed Bakhash; Siras Hakobyan; Tamara Sirunyan
EPI_ISL_1302021, EPI_ISL_1401127, EPI_ISL_1401146, EPI_ISL_1401209, EPI_ISL_1509817, EPI_ISL_1509830, EPI_ISL_1509880, EPI_ISL_1608257, EPI_ISL_1786218, EPI_ISL_1786337, EPI_ISL_2081837, EPI_ISL_2081899, EPI_ISL_2081935, EPI_ISL_2082000, EPI_ISL_2180061, EPI_ISL_2379109, EPI_ISL_2379203, EPI_ISL_2621277, EPI_ISL_2621428, EPI_ISL_2621436, EPI_ISL_2621444, EPI_ISL_2621471, EPI_ISL_2621494			
see above	National Center of Infectious and Parasitic Diseases	National Center of Infectious and Parasitic Diseases	Alexiev; Alexiev et al; Dimitrova; Dobrinov; Donchev; Grigorova I.; Grigorova L.; Hristova; Ivanov; Kantardjiev; Korsun; Philipova; Stoitsova; Stoykov; Trifonova
EPI_ISL_1544014, EPI_ISL_1544070, EPI_ISL_2162392, EPI_ISL_2162393, EPI_ISL_2162395, EPI_ISL_2162411, EPI_ISL_2162417, EPI_ISL_2162423, EPI_ISL_2162425, EPI_ISL_2162427, EPI_ISL_2162429, EPI_ISL_2162435, EPI_ISL_2162437, EPI_ISL_2162438, EPI_ISL_2162441, EPI_ISL_2162444, EPI_ISL_2162445, EPI_ISL_2162455, EPI_ISL_2162461, EPI_ISL_2225632, EPI_ISL_2225633, EPI_ISL_2225637, EPI_ISL_2225642, EPI_ISL_2225644, EPI_ISL_2225645, EPI_ISL_2225647, EPI_ISL_2225649, EPI_ISL_2225654, EPI_ISL_2225658, EPI_ISL_2225660, EPI_ISL_2225661, EPI_ISL_2225662, EPI_ISL_2225664, EPI_ISL_2225666, EPI_ISL_2225669, EPI_ISL_2225671, EPI_ISL_2225672, EPI_ISL_2225673, EPI_ISL_2225675, EPI_ISL_2225676, EPI_ISL_2225677, EPI_ISL_2225678, EPI_ISL_2225679, EPI_ISL_2225681, EPI_ISL_2225682, EPI_ISL_2225684, EPI_ISL_2225685, EPI_ISL_2225686, EPI_ISL_2225688, EPI_ISL_2225689, EPI_ISL_2225690, EPI_ISL_2225691, EPI_ISL_2225692, EPI_ISL_2225693, EPI_ISL_2233145, EPI_ISL_2233146, EPI_ISL_2304047, EPI_ISL_2304050, EPI_ISL_2304057, EPI_ISL_2304058, EPI_ISL_2304062, EPI_ISL_2304073, EPI_ISL_2304079, EPI_ISL_2304082, EPI_ISL_2304085, EPI_ISL_2332704, EPI_ISL_2332706, EPI_ISL_2332707, EPI_ISL_2332708, EPI_ISL_2332709, EPI_ISL_2332711, EPI_ISL_2332713, EPI_ISL_2332714, EPI_ISL_2332716, EPI_ISL_2332717, EPI_ISL_2332718, EPI_ISL_2332719, EPI_ISL_2332721, EPI_ISL_2332722, EPI_ISL_2332723, EPI_ISL_2332724, EPI_ISL_2332725, EPI_ISL_2332726, EPI_ISL_2332727, EPI_ISL_2332728, EPI_ISL_2332729, EPI_ISL_2332730, EPI_ISL_2332731, EPI_ISL_2332736, EPI_ISL_2332737, EPI_ISL_2332739, EPI_ISL_2332740, EPI_ISL_2332743, EPI_ISL_2332744, EPI_ISL_2332745, EPI_ISL_2332746, EPI_ISL_2332747, EPI_ISL_2332748, EPI_ISL_2332749, EPI_ISL_2332750, EPI_ISL_2332751, EPI_ISL_2332752, EPI_ISL_2332753, EPI_ISL_2332754, EPI_ISL_2332755, EPI_ISL_2332756, EPI_ISL_2332757, EPI_ISL_2332758, EPI_ISL_2332759, EPI_ISL_2332760, EPI_ISL_2332761, EPI_ISL_2332763, EPI_ISL_2332764, EPI_ISL_2332765, EPI_ISL_2332768, EPI_ISL_2332772, EPI_ISL_2332773, EPI_ISL_2332778, EPI_ISL_2332779, EPI_ISL_2332780, EPI_ISL_2332781, EPI_ISL_2332784, EPI_ISL_2332786, EPI_ISL_2332787, EPI_ISL_2332788			
see above	National Centre For Cell Science	National Centre For Cell Science – INSACOG	Ajay Pillai; Dhiraj Paul; INSACOG Consortium team; Manoj Kumar Bhat; Mitali Inamdar; Mohak P Gujare; Shivang P. Bhanushali; Sonal Manik Chavan; Yogesh Shouche
EPI_ISL_2382970, EPI_ISL_2382971, EPI_ISL_2382972, EPI_ISL_2382974, EPI_ISL_2382975, EPI_ISL_2382977, EPI_ISL_2382986, EPI_ISL_2382988, EPI_ISL_2382996, EPI_ISL_2382998, EPI_ISL_2383003, EPI_ISL_2383004, EPI_ISL_2383005			
see above	National Centre For Cell Science	National Centre For Cell Science-INSACOG	Ajay Pillai; Dhiraj Paul; INSACOG Consortium team; Manoj Kumar Bhat; Mitali Inamdar; Mohak P Gujare; Shivang P. Bhanushali; Yogesh Shouche
EPI_ISL_2107009, EPI_ISL_2107010, EPI_ISL_2107017, EPI_ISL_2107020, EPI_ISL_2107022, EPI_ISL_2107023, EPI_ISL_2107025, EPI_ISL_2107027, EPI_ISL_2107033, EPI_ISL_2107034, EPI_ISL_2107060, EPI_ISL_2107062, EPI_ISL_2107063, EPI_ISL_2107066, EPI_ISL_2107070, EPI_ISL_2107071, EPI_ISL_2107072, EPI_ISL_2107075, EPI_ISL_2107077, EPI_ISL_2107080, EPI_ISL_2107081, EPI_ISL_2107083, EPI_ISL_2107085, EPI_ISL_2107087, EPI_ISL_2107088, EPI_ISL_2107089, EPI_ISL_2107090			
see above	National Centre For Cell Science – INSACOG	National Centre For Cell Science	Ajay Pillai; Dhiraj Paul; INSACOG Consortium team; Manoj Kumar Bhat; Mitali Inamdar; Mohak P Gujare; Shivang P. Bhanushali; Yogesh Shouche
EPI_ISL_2099644, EPI_ISL_2233495, EPI_ISL_2233544, EPI_ISL_2233550, EPI_ISL_2233595, EPI_ISL_2233667	National Centre for Disease Control	CDFD -INSACOG	Ashwin Dalal; Asmita Gupta; Divya Vashisht; Murali Bashyam; Pratyusha Bala; To be addded later; Vinay Donipadi
EPI_ISL_2448188, EPI_ISL_2459692, EPI_ISL_2459973, EPI_ISL_2460052, EPI_ISL_2460533, EPI_ISL_2460546, EPI_ISL_2460547, EPI_ISL_2460550, EPI_ISL_2460605, EPI_ISL_2460627, EPI_ISL_2460633, EPI_ISL_2460681, EPI_ISL_2460703, EPI_ISL_2460707, EPI_ISL_2460786, EPI_ISL_2460794, EPI_ISL_2460798, EPI_ISL_2460863, EPI_ISL_2460868, EPI_ISL_2460869, EPI_ISL_2460870, EPI_ISL_2460871, EPI_ISL_2460872, EPI_ISL_2460873, EPI_ISL_2460874, EPI_ISL_2460884, EPI_ISL_2460886, EPI_ISL_2460894, EPI_ISL_2460895, EPI_ISL_2460896, EPI_ISL_2460901, EPI_ISL_2460903, EPI_ISL_2460906, EPI_ISL_2460909, EPI_ISL_2460912, EPI_ISL_2460913, EPI_ISL_2460914, EPI_ISL_2460915, EPI_ISL_2460916, EPI_ISL_2460917, EPI_ISL_2460918, EPI_ISL_2460919, EPI_ISL_2460927, EPI_ISL_2460929, EPI_ISL_2460930, EPI_ISL_2460931, EPI_ISL_2460932, EPI_ISL_2460933, EPI_ISL_2460935, EPI_ISL_2460936, EPI_ISL_2460937, EPI_ISL_2460940, EPI_ISL_2460942, EPI_ISL_2460943, EPI_ISL_2460944, EPI_ISL_2460945, EPI_ISL_2460950, EPI_ISL_2460951, EPI_ISL_2460952, EPI_ISL_2460953, EPI_ISL_2460956, EPI_ISL_2460957, EPI_ISL_2460958, EPI_ISL_2460960, EPI_ISL_2460961, EPI_ISL_2460963, EPI_ISL_2460965, EPI_ISL_2460970, EPI_ISL_2460971, EPI_ISL_2460973, EPI_ISL_2460975, EPI_ISL_2460976, EPI_ISL_2460978, EPI_ISL_2460979, EPI_ISL_2460982, EPI_ISL_2460984, EPI_ISL_2460985, EPI_ISL_2460986, EPI_ISL_2460988, EPI_ISL_2460989, EPI_ISL_2460990, EPI_ISL_2460991, EPI_ISL_2460996, EPI_ISL_2461001, EPI_ISL_2461005, EPI_ISL_2461007, EPI_ISL_2461010, EPI_ISL_2461011, EPI_ISL_2461013, EPI_ISL_2461014, EPI_ISL_2461015, EPI_ISL_2461016, EPI_ISL_2461021, EPI_ISL_2461023, EPI_ISL_2461024, EPI_ISL_2461025, EPI_ISL_2461026, EPI_ISL_2461027, EPI_ISL_2461028, EPI_ISL_2461029, EPI_ISL_2461030, EPI_ISL_2461031, EPI_ISL_2461032, EPI_ISL_2461033, EPI_ISL_2461035, EPI_ISL_2461036, EPI_ISL_2461037, EPI_ISL_2461038, EPI_ISL_2461039, EPI_ISL_2461040, EPI_ISL_2461042, EPI_ISL_2461043, EPI_ISL_2461044, EPI_ISL_2461045, EPI_ISL_2461046, EPI_ISL_2461047, EPI_ISL_2461048, EPI_ISL_2461049, EPI_ISL_2461050, EPI_ISL_2461051, EPI_ISL_2461052, EPI_ISL_2461053, EPI_ISL_2461054, EPI_ISL_2461055, EPI_ISL_2461056, EPI_ISL_2461057, EPI_ISL_2461058, EPI_ISL_2461059, EPI_ISL_2461060, EPI_ISL_2461061, EPI_ISL_2461062, EPI_ISL_2461063, EPI_ISL_2461064, EPI_ISL_2461065, EPI_ISL_2461066, EPI_ISL_2461067, EPI_ISL_2461068, EPI_ISL_2461069, EPI_ISL_2461070, EPI_ISL_2461071, EPI_ISL_2461072, EPI_ISL_2461073, EPI_ISL_2461075, EPI_ISL_2461076, EPI_ISL_2461078, EPI_ISL_2461080, EPI_ISL_2461081, EPI_ISL_2461082, EPI_ISL_2461083, EPI_ISL_2461084, EPI_ISL_2461086, EPI_ISL_2461088, EPI_ISL_2461089, EPI_ISL_2461090, EPI_ISL_2461091, EPI_ISL_2461093, EPI_ISL_2461094, EPI_ISL_2461095, EPI_ISL_2461096, EPI_ISL_2461097, EPI_ISL_2461098, EPI_ISL_2461099, EPI_ISL_2461109, EPI_ISL_2461113, EPI_ISL_2461116, EPI_ISL_2461117, EPI_ISL_2461120, EPI_ISL_2461122, EPI_ISL_2461123, EPI_ISL_2461124, EPI_ISL_2461125, EPI_ISL_2461127, EPI_ISL_2461128, EPI_ISL_2461132, EPI_ISL_2461135, EPI_ISL_2461136, EPI_ISL_2461137, EPI_ISL_2461139, EPI_ISL_2461143, EPI_ISL_2461146, EPI_ISL_2461149, EPI_ISL_2461150, EPI_ISL_2461151, EPI_ISL_2461152, EPI_ISL_2461153, EPI_ISL_2461154, EPI_ISL_2461155, EPI_ISL_2461156, EPI_ISL_2461158, EPI_ISL_2461159, EPI_ISL_2461160, EPI_ISL_2461161, EPI_ISL_2461162, EPI_ISL_2461163, EPI_ISL_2461164, EPI_ISL_2461165, EPI_ISL_2461167, EPI_ISL_2461169, EPI_ISL_2461170, EPI_ISL_2461171, EPI_ISL_2461172, EPI_ISL_2461173, EPI_ISL_2461174, EPI_ISL_2461181, EPI_ISL_2461184, EPI_ISL_2461185, EPI_ISL_2461186, EPI_ISL_2461193, EPI_ISL_2461194, EPI_ISL_2461195, EPI_ISL_2461196, EPI_ISL_2461203, EPI_ISL_2461210, EPI_ISL_2461211, EPI_ISL_2461213, EPI_ISL_2461214, EPI_ISL_2461215, EPI_ISL_2461217, EPI_ISL_2461218, EPI_ISL_2461219, EPI_ISL_2461223, EPI_ISL_2461224, EPI_ISL_2461228, EPI_ISL_2461229, EPI_ISL_2461230, EPI_ISL_2461233, EPI_ISL_2461234, EPI_ISL_2461237, EPI_ISL_2461238, EPI_ISL_2461239, EPI_ISL_2461240, EPI_ISL_2461242, EPI_ISL_2461243, EPI_ISL_2461245, EPI_ISL_2461246, EPI_ISL_2461249, EPI_ISL_2461250, EPI_ISL_2461252, EPI_ISL_2461253, EPI_ISL_2461254, EPI_ISL_2461255, EPI_ISL_2461256, EPI_ISL_2461257, EPI_ISL_2461258, EPI_ISL_2461259, EPI_ISL_2461260, EPI_ISL_2461261, EPI_ISL_2461263, EPI_ISL_2461264, EPI_ISL_2461265, EPI_ISL_2461266, EPI_ISL_2461267, EPI_ISL_2461270, EPI_ISL_2461271, EPI_ISL_2461272, EPI_ISL_2461273, EPI_ISL_2461274, EPI_ISL_2461275, EPI_ISL_2461276, EPI_ISL_2461279, EPI_ISL_2461280, EPI_ISL_2461282, EPI_ISL_2461283, EPI_ISL_2461285, EPI_ISL_2461286, EPI_ISL_2461287, EPI_ISL_2461295, EPI_ISL_2461316, EPI_ISL_2461317, EPI_ISL_2461318, EPI_ISL_2461341, EPI_ISL_2461349, EPI_ISL_2461358, EPI_ISL_2461359, EPI_ISL_2461362, EPI_ISL_2461363, EPI_ISL_2461364, EPI_ISL_2461365, EPI_ISL_2461366, EPI_ISL_2461367, EPI_ISL_2461368, EPI_ISL_2461369, EPI_ISL_2461376, EPI_ISL_2461377, EPI_ISL_2461378, EPI_ISL_2461379, EPI_ISL_2461380, EPI_ISL_2461381, EPI_ISL_2461382, EPI_ISL_2461383, EPI_ISL_2461384, EPI_ISL_2461385, EPI_ISL_2461386, EPI_ISL_2461387, EPI_ISL_2461388, EPI_ISL_2461389, EPI_ISL_2461391, EPI_ISL_2461392, EPI_ISL_2461393, EPI_ISL_2461394, EPI_ISL_2461395, EPI_ISL_2461396, EPI_ISL_2461397, EPI_ISL_2461398, EPI_ISL_2461399, EPI_ISL_2461400, EPI_ISL_2461401, EPI_ISL_2461402, EPI_ISL_2461403, EPI_ISL_2461404, EPI_ISL_2461405, EPI_ISL_2461406, EPI_ISL_2461407, EPI_ISL_2461408, EPI_ISL_2461409, EPI_ISL_2461410, EPI_ISL_2461411, EPI_ISL_2461412, EPI_ISL_2461414, EPI_ISL_2461415, EPI_ISL_2461416, EPI_ISL_2461417, EPI_ISL_2461418, EPI_ISL_2461419, EPI_ISL_2461420, EPI_ISL_2461421, EPI_ISL_2461422, EPI_ISL_2461423, EPI_ISL_2461424, EPI_ISL_2461425, EPI_ISL_2461426, EPI_ISL_2461427, EPI_ISL_2461428, EPI_ISL_2461429, EPI_ISL_2461430, EPI_ISL_2461431, EPI_ISL_2461432, EPI_ISL_2461433, EPI_ISL_2461434, EPI_ISL_2461435, EPI_ISL_2461436, EPI_ISL_2461437, EPI_ISL_2461438, EPI_ISL_2461439, EPI_ISL_2461440, EPI_ISL_2461441, EPI_ISL_2461442, EPI_ISL_2461443, EPI_ISL_2461444, EPI_ISL_2461445, EPI_ISL_2461446, EPI_ISL_2461447, EPI_ISL_2461448, EPI_ISL_2461449, EPI_ISL_2461450, EPI_ISL_2461451, EPI_ISL_2461452, EPI_ISL_2461453, EPI_ISL_2461454, EPI_ISL_2461455, EPI_ISL_2461456, EPI_ISL_2461457, EPI_ISL_2461458, EPI_ISL_2461459, EPI_ISL_2461460, EPI_ISL_2461461, EPI_ISL_2461462, EPI_ISL_2461463, EPI_ISL_2461464, EPI_ISL_2461465, EPI_ISL_2461466, EPI_ISL_2461467, EPI_ISL_2461468, EPI_ISL_2461469, EPI_ISL_2461470, EPI_ISL_2461471, EPI_ISL_2461472, EPI_ISL_2461473, EPI_ISL_2461474, EPI_ISL_2461475, EPI_ISL_2461496, EPI_ISL_2461501, EPI_ISL_2461509, EPI_ISL_2461510, EPI_ISL_2461511, EPI_ISL_2461513, EPI_ISL_2461514, EPI_ISL_2461515, EPI_ISL_2461517, EPI_ISL_2461519, EPI_ISL_2461521, EPI_ISL_2461522, EPI_ISL_2461524, EPI_ISL_2461526, EPI_ISL_2461527, EPI_ISL_2461528, EPI_ISL_2461530, EPI_ISL_2461531, EPI_ISL_2461532, EPI_ISL_2461534, EPI_ISL_2461535, EPI_ISL_2461536, EPI_ISL_2461537, EPI_ISL_2461538, EPI_ISL_2461539, EPI_ISL_2461541, EPI_ISL_2461542, EPI_ISL_2461543, EPI_ISL_2461544, EPI_ISL_2461545, EPI_ISL_2461546, EPI_ISL_2461547, EPI_ISL_2461548, EPI_ISL_2461549, EPI_ISL_2461550, EPI_ISL_2461551, EPI_ISL_2461552, EPI_ISL_2461554, EPI_ISL_2461555, EPI_ISL_2461556, EPI_ISL_2461558, EPI_ISL_2461559, EPI_ISL_2461560, EPI_ISL_2461561, EPI_ISL_2461562, EPI_ISL_2461563, EPI_ISL_2461564, EPI_ISL_2461565, EPI_ISL_2461566, EPI_ISL_2461567, EPI_ISL_2461568, EPI_ISL_2461569, EPI_ISL_2461570, EPI_ISL_2461571, EPI_ISL_2461572, EPI_ISL_2461573, EPI_ISL_2461574, EPI_ISL_2461575, EPI_ISL_2461576, EPI_ISL_2461577, EPI_ISL_2461578, EPI_ISL_2461579, EPI_ISL_2461580, EPI_ISL_2461581, EPI_ISL_2461582, EPI_ISL_2461583, EPI_ISL_2461584, EPI_ISL_2461585, EPI_ISL_2461586, EPI_ISL_2461587, EPI_ISL_2461589, EPI_ISL_2461590, EPI_ISL_2461591, EPI_ISL_2461592, EPI_ISL_2461593, EPI_ISL_2461594, EPI_ISL_2461595, EPI_ISL_2461596, EPI_ISL_2461597, EPI_ISL_2461598, EPI_ISL_2461599, EPI_ISL_2461600, EPI_ISL_2461601, EPI_ISL_2461602, EPI_ISL_2461603, EPI_ISL_2461604, EPI_ISL_2461623, EPI_ISL_2461624, EPI_ISL_2461631, EPI_ISL_2461632, EPI_ISL_2461635, EPI_ISL_2461637, EPI_ISL_2461638, EPI_ISL_2461640, EPI_ISL_2461642, EPI_ISL_2461644, EPI_ISL_2461645, EPI_ISL_2461647, EPI_ISL_2461648, EPI_ISL_2461652, EPI_ISL_2461653, EPI_ISL_2461661, EPI_ISL_2461662, EPI_ISL_2461665, EPI_ISL_2461671, EPI_ISL_2461672, EPI_ISL_2461679, EPI_ISL_2461680, EPI_ISL_2461682, EPI_ISL_2461688, EPI_ISL_2461690, EPI_ISL_2461692, EPI_ISL_2461693, EPI_ISL_2461694, EPI_ISL_2461696, EPI_ISL_2461697, EPI_ISL_2461698, EPI_ISL_2461699, EPI_ISL_2461700, EPI_ISL_2461701, EPI_ISL_2461702, EPI_ISL_2461703, EPI_ISL_2461704, EPI_ISL_2461705, EPI_ISL_2461706, EPI_ISL_2461707, EPI_ISL_2461708, EPI_ISL_2461709, EPI_ISL_2461710, EPI_ISL_2461711, EPI_ISL_2461712, EPI_ISL_2461713, EPI_ISL_2461714, EPI_ISL_2461715, EPI_ISL_2461716, EPI_ISL_2461717, EPI_ISL_2461718, EPI_ISL_2461719, EPI_ISL_2461720, EPI_ISL_2461721, EPI_ISL_2461722, EPI_ISL_2461723, EPI_ISL_2461724, EPI_ISL_2461725, EPI_ISL_2461726, EPI_ISL_2461727, EPI_ISL_2461728, EPI_ISL_2461729, EPI_ISL_2461730, EPI_ISL_2461731, EPI_ISL_2461732, EPI_ISL_2461733, EPI_ISL_2461734, EPI_ISL_2461735, EPI_ISL_2461736, EPI_ISL_2461737, EPI_ISL_2461738, EPI_ISL_2461739, EPI_ISL_2461740, EPI_ISL_2461741, EPI_ISL_2461742, EPI_ISL_2461743, EPI_ISL_2461744, EPI_ISL_2461745, EPI_ISL_2461746, EPI_ISL_2461747, EPI_ISL_2461748, EPI_ISL_2461749, EPI_ISL_2461750, EPI_ISL_2461751, EPI_ISL_2461752, EPI_ISL_2461753, EPI_ISL_2461754, EPI_ISL_2461755, EPI_ISL_2461756, EPI_ISL_2461757, EPI_ISL_2461758, EPI_ISL_2461759, EPI_ISL_2461771, EPI_ISL_2461775, EPI_ISL_2461780, EPI_ISL_2461781, EPI_ISL_2461784, EPI_ISL_2461785, EPI_ISL_2461786, EPI_ISL_2461787, EPI_ISL_2461788, EPI_ISL_2461789, EPI_ISL_2461790, EPI_ISL_2461791, EPI_ISL_2461793, EPI_ISL_2461795, EPI_ISL_2461796, EPI_ISL_2461798, EPI_ISL_2461801, EPI_ISL_2461802, EPI_ISL_2461803, EPI_ISL_2461804, EPI_ISL_2461805, EPI_ISL_2461806, EPI_ISL_2461808, EPI_ISL_2461809, EPI_ISL_2461810, EPI_ISL_2461811, EPI_ISL_2461812, EPI_ISL_2461814, EPI_ISL_2461815, EPI_ISL_2461816, EPI_ISL_2461817, EPI_ISL_2461818, EPI_ISL_2461819, EPI_ISL_2461820, EPI_ISL_2461821, EPI_ISL_2461822, EPI_ISL_2461823, EPI_ISL_2461824, EPI_ISL_2461825, EPI_ISL_2461826, EPI_ISL_2461827, EPI_ISL_2461828, EPI_ISL_2461829, EPI_ISL_2461830, EPI_ISL_2461831, EPI_ISL_2461832, EPI_ISL_2461833, EPI_ISL_2461835, EPI_ISL_2461836, EPI_ISL_2461922, EPI_ISL_2461924, EPI_ISL_2461929, EPI_ISL_2461930, EPI_ISL_2461939, EPI_ISL_2461940, EPI_ISL_2461941, EPI_ISL_2461942, EPI_ISL_2461944, EPI_ISL_2461945, EPI_ISL_2461948, EPI_ISL_2461949, EPI_ISL_2461954, EPI_ISL_2461955, EPI_ISL_2461956, EPI_ISL_2461957, EPI_ISL_2461958, EPI_ISL_2461959, EPI_ISL_2461960, EPI_ISL_2461961, EPI_ISL_2461962, EPI_ISL_2461963, EPI_ISL_2461964, EPI_ISL_2461965, EPI_ISL_2461966, EPI_ISL_2461967, EPI_ISL_2461971, EPI_ISL_2461975, EPI_ISL_2461976, EPI_ISL_2461977, EPI_ISL_2461979, EPI_ISL_2461980, EPI_ISL_2461981, EPI_ISL_2461982, EPI_ISL_2461984, EPI_ISL_2461985, EPI_ISL_2461986, EPI_ISL_2461987, EPI_ISL_2461988, EPI_ISL_2461991, EPI_ISL_2461992, EPI_ISL_2461993, EPI_ISL_2461994, EPI_ISL_2461995, EPI_ISL_2461996, EPI_ISL_2461997, EPI_ISL_2461998, EPI_ISL_2461999, EPI_ISL_2462000, EPI_ISL_2462001, EPI_ISL_2462002, EPI_ISL_2462003, EPI_ISL_2462004, EPI_ISL_2462005, EPI_ISL_2462006, EPI_ISL_2462007, EPI_ISL_2462008, EPI_ISL_2462009, EPI_ISL_2462010, EPI_ISL_2462011, EPI_ISL_2462012, EPI_ISL_2462013, EPI_ISL_2462014, EPI_ISL_2462015, EPI_ISL_2462016, EPI_ISL_2462017, EPI_ISL_2462018, EPI_ISL_2462019, EPI_ISL_2462020, EPI_ISL_2462021, EPI_ISL_2462022, EPI_ISL_2462023, EPI_ISL_2462024, EPI_ISL_2462025, EPI_ISL_2462026, EPI_ISL_2462027, EPI_ISL_2502028, EPI_ISL_2502029, EPI_ISL_2502030, EPI_ISL_2502031, EPI_ISL_2502036, EPI_ISL_2502059, EPI_ISL_2502060, EPI_ISL_2502065, EPI_ISL_2502071, EPI_ISL_2502083, EPI_ISL_2502085, EPI_ISL_2502088, EPI_ISL_2502095, EPI_ISL_2502098, EPI_ISL_2502106, EPI_ISL_2502109, EPI_ISL_2502110, EPI_ISL_2502113, EPI_ISL_2502120, EPI_ISL_2502129, EPI_ISL_2502171, EPI_ISL_2502172, EPI_ISL_2502174, EPI_ISL_2502178, EPI_ISL_2502180, EPI_ISL_2502182, EPI_ISL_2502185, EPI_ISL_2502188, EPI_ISL_2502192, EPI_ISL_2502196, EPI_ISL_2502212, EPI_ISL_2502214, EPI_ISL_2502215, EPI_ISL_2502217, EPI_ISL_2502218, EPI_ISL_2502222, EPI_ISL_2502223, EPI_ISL_2502231, EPI_ISL_2502232, EPI_ISL_2502237, EPI_ISL_2502247, EPI_ISL_2502248, EPI_ISL_2502266, EPI_ISL_2502268, EPI_ISL_2502269, EPI_ISL_2502270, EPI_ISL_2502272, EPI_ISL_2502273, EPI_ISL_2502274, EPI_ISL_2502275, EPI_ISL_2502278, EPI_ISL_2502279, EPI_ISL_2502289, EPI_ISL_2502291, EPI_ISL_2502296, EPI_ISL_2502297, EPI_ISL_2502298, EPI_ISL_2502299, EPI_ISL_2502300, EPI_ISL_2502301, EPI_ISL_2502304, EPI_ISL_2504284, EPI_ISL_2504861, EPI_ISL_2555551, EPI_ISL_2555552, EPI_ISL_2555553, EPI_ISL_2555554, EPI_ISL_2555555, EPI_ISL_2555558, EPI_ISL_2555562, EPI_ISL_2555563, EPI_ISL_2555564, EPI_ISL_2555565, EPI_ISL_2555566, EPI_ISL_2555567, EPI_ISL_2555568, EPI_ISL_2555569, EPI_ISL_2555571, EPI_ISL_2555575, EPI_ISL_2555576, EPI_ISL_2555577, EPI_ISL_2555580, EPI_ISL_2555581, EPI_ISL_2555583, EPI_ISL_2555584, EPI_ISL_2555585, EPI_ISL_2555586, EPI_ISL_2555587, EPI_ISL_2555588, EPI_ISL_2555589, EPI_ISL_2555590, EPI_ISL_2555592, EPI_ISL_2555594, EPI_ISL_2555596, EPI_ISL_2555600, EPI_ISL_2555606, EPI_ISL_2555607, EPI_ISL_2555608, EPI_ISL_2555609, EPI_ISL_2555611, EPI_ISL_2555612, EPI_ISL_2555613, EPI_ISL_2555617, EPI_ISL_2555618, EPI_ISL_2555619, EPI_ISL_2555620, EPI_ISL_2555622, EPI_ISL_2555623, EPI_ISL_2555624, EPI_ISL_2555625, EPI_ISL_2555626, EPI_ISL_2555627, EPI_ISL_2555628, EPI_ISL_2555629, EPI_ISL_2555630, EPI_ISL_2555631, EPI_ISL_2555632, EPI_ISL_2555634, EPI_ISL_2555635, EPI_ISL_2555636, EPI_ISL_2555637, EPI_ISL_2555640, EPI_ISL_2555642, EPI_ISL_2555643, EPI_ISL_2555645, EPI_ISL_2555646, EPI_ISL_2555648, EPI_ISL_2555650, EPI_ISL_2555651, EPI_ISL_2555652, EPI_ISL_2555653, EPI_ISL_2555655, EPI_ISL_2555656, EPI_ISL_2555657, EPI_ISL_2555658, EPI_ISL_2555733, EPI_ISL_2555735, EPI_ISL_2555736, EPI_ISL_2555741, EPI_ISL_2555742, EPI_ISL_2555743, EPI_ISL_2555744, EPI_ISL_2555745, EPI_ISL_2555747, EPI_ISL_2555750, EPI_ISL_2555751, EPI_ISL_2555756, EPI_ISL_2555757, EPI_ISL_2555758, EPI_ISL_2555760, EPI_ISL_2555761, EPI_ISL_2555762, EPI_ISL_2555763, EPI_ISL_2555764, EPI_ISL_2555765, EPI_ISL_2555766, EPI_ISL_2555767, EPI_ISL_2555769, EPI_ISL_2555773, EPI_ISL_2555774, EPI_ISL_2555777, EPI_ISL_2555778, EPI_ISL_2555779, EPI_ISL_2555780, EPI_ISL_2555782, EPI_ISL_2555783, EPI_ISL_2555784, EPI_ISL_2555787, EPI_ISL_2555789, EPI_ISL_2555790, EPI_ISL_2555792, EPI_ISL_2555793, EPI_ISL_2555796, EPI_ISL_2555807, EPI_ISL_2555808, EPI_ISL_2555811, EPI_ISL_2555812, EPI_ISL_2555813, EPI_ISL_2555814, EPI_ISL_2555815, EPI_ISL_2555816, EPI_ISL_2555817, EPI_ISL_2555818, EPI_ISL_2555819, EPI_ISL_2555820, EPI_ISL_2555821, EPI_ISL_2555822, EPI_ISL_2555827, EPI_ISL_2555829, EPI_ISL_2555830, EPI_ISL_2555833, EPI_ISL_2555842, EPI_ISL_2555843, EPI_ISL_2555849, EPI_ISL_2555851, EPI_ISL_2555852, EPI_ISL_2555853, EPI_ISL_2555857, EPI_ISL_2555859, EPI_ISL_2555863, EPI_ISL_2555865, EPI_ISL_2555866, EPI_ISL_2555867, EPI_ISL_2555868, EPI_ISL_2555869, EPI_ISL_2555870, EPI_ISL_2555872, EPI_ISL_2555874, EPI_ISL_2555877, EPI_ISL_2555878, EPI_ISL_2555879, EPI_ISL_2555880, EPI_ISL_2555881, EPI_ISL_2555883, EPI_ISL_2555885, EPI_ISL_2555886, EPI_ISL_2555889, EPI_ISL_2555894, EPI_ISL_2555896, EPI_ISL_2555899, EPI_ISL_2555900, EPI_ISL_2555901, EPI_ISL_2555907, EPI_ISL_2555909, EPI_ISL_2555911, EPI_ISL_2555929, EPI_ISL_2555931, EPI_ISL_2555937, EPI_ISL_2555941, EPI_ISL_2555942, EPI_ISL_2555943, EPI_ISL_2555947, EPI_ISL_2555950, EPI_ISL_2555951, EPI_ISL_2555963, EPI_ISL_2555966, EPI_ISL_2555969, EPI_ISL_2555970, EPI_ISL_2555971, EPI_ISL_2555972, EPI_ISL_2555976, EPI_ISL_2555978, EPI_ISL_2555979, EPI_ISL_2555980, EPI_ISL_2555984, EPI_ISL_2555989, EPI_ISL_2555995, EPI_ISL_2555997, EPI_ISL_2555998, EPI_ISL_2555999, EPI_ISL_2556006, EPI_ISL_2556011, EPI_ISL_2556012, EPI_ISL_2556014, EPI_ISL_2556021, EPI_ISL_2556022, EPI_ISL_2556025, EPI_ISL_2556026, EPI_ISL_2556027, EPI_ISL_2556030, EPI_ISL_2556034, EPI_ISL_2556039, EPI_ISL_2556040, EPI_ISL_2556043, EPI_ISL_2556044, EPI_ISL_2556047, EPI_ISL_2556050, EPI_ISL_2556052, EPI_ISL_2556053, EPI_ISL_2556055, EPI_ISL_2556060, EPI_ISL_2556061, EPI_ISL_2556062, EPI_ISL_2556066, EPI_ISL_2556067, EPI_ISL_2556071, EPI_ISL_2556073, EPI_ISL_2556074, EPI_ISL_2556075, EPI_ISL_2556076, EPI_ISL_2556077, EPI_ISL_2556078, EPI_ISL_2556079, EPI_ISL_2556080, EPI_ISL_2556081, EPI_ISL_2556085, EPI_ISL_2556086, EPI_ISL_2556088, EPI_ISL_2556090, EPI_ISL_2556100, EPI_ISL_2556101, EPI_ISL_2556102, EPI_ISL_2556104, EPI_ISL_2556105, EPI_ISL_2556106, EPI_ISL_2556108, EPI_ISL_2556109, EPI_ISL_2556110, EPI_ISL_2556187, EPI_ISL_2556200, EPI_ISL_2556447, EPI_ISL_2556448, EPI_ISL_2556524, EPI_ISL_2556527, EPI_ISL_2556566, EPI_ISL_2556582, EPI_ISL_2556583, EPI_ISL_2556605, EPI_ISL_2556679, EPI_ISL_2556857, EPI_ISL_2556971, EPI_ISL_2556976, EPI_ISL_2556979, EPI_ISL_2556999, EPI_ISL_2557000, EPI_ISL_2557002, EPI_ISL_2557021, EPI_ISL_2557032			
see above	National Centre for Disease Control (NCDC) Biotechnology Division, Delhi	NCDC Delhi, Biotechnology Division INSACOG	Hema Gogia; Hemlata Lall; Kalaiarasan Ponnusamy; Mahesh S Dhar; Manoj K Singh; Meena Datta; Partha Rakshit; Preeti Madan; Priyanka Singh; Radhakrishnan V. S; Robin Marwal; Sandhya Kabra; Sujeet K Singh; Uma Sharma
EPI_ISL_435082	National Centre for Disease control (NCDC), CSIR-Institute of Genomics and Integrative Biology (CSIR-IGIB)	NCDC/CSIR-IGIB	Aarti Tewari; Anurag Agrawal; Bharathram Uppili; Bibhash Nandi; Debasis Dash; Dhirendra Kumar; Hema Gogia; Hemlata Lall; Himanshu Vashisht; Mahesh Dhar; Manju Bala; Meena Datta; Mitali Mukerji; Mohammed Faruq; Nidhi Saini; Nishu Tyagi; Partha Rakshit; Pooja Sharma; Poonam Gupta; Pramod Kumar; Prateek Singh; Preeti Madan; Priyanka Singh; Rajesh Pandey; Sandhya Kabra; Saruchi Wadhwa; Satyabrata Bag; Simrita Singh; Sujeet Singh; Uma Sharma; Varun Jaiswal; Vivekanand A
EPI_ISL_2492624, EPI_ISL_2492684, EPI_ISL_2492699, EPI_ISL_2492700, EPI_ISL_2492734, EPI_ISL_2492945, EPI_ISL_2493032, EPI_ISL_2493068, EPI_ISL_2493069			
see above	National HIV Reference Laboratory, Ministry of Health, Public Health Institute of Malawi	CERI, Centre for Epidemic Response and Innvoation, Stellenbosch University and KRISP, KZN Research Innovation and Sequencing Platform, UKZN.	Auld A; Chilima B; Chiwaula M; Emmanuel SJ; Giandhari J; Kaba M; Kampira E; Kasambara W; Kim L; Lessells R; Maida A; Mvula B; Mwangomba W; Naidoo Y; Panja L; Pillay S; Tegally H; Wadonda N; Wilkinson E; de Oliveira T
EPI_ISL_2494782, EPI_ISL_2494899, EPI_ISL_2494924, EPI_ISL_2494966, EPI_ISL_2494972, EPI_ISL_2495038, EPI_ISL_2495039, EPI_ISL_2495042, EPI_ISL_2495043			
see above	National HIV Reference Laboratory, Ministry of Health, Public Health Institute of Malawi	KRISP, KZN Research Innovation and Sequencing Platform	Auld A; Chilima B; Chiwaula M; Emmanuel SJ; Giandhari J; Kaba M; Kampira E; Kasambara W; Kim L; Lessells R; Maida A; Mvula B; Mwangomba W; Naidoo Y; Panja L; Pillay S; Tegally H; Wadonda N; Wilkinson E; de Oliveira T
EPI_ISL_560387	National Health Laboratory	Botswana Institute for Technology Research and Innovation	Dineo Emang Tshiamo. Gape Nyepetsi; Kefentse Arnold Tumedi; Madisa Mine; Maitshwarelo Ignatius Matsheka; Thongbotho Mphoyakgosi
EPI_ISL_770470	National Health laboratory	Botswana Institute for Technology Research and Innovation	Dineo Emang Tshiamo. Gape Nyepetsi; Kefentse Arnold Tumedi; Madisa Mine; Maitshwarelo Ignatius Matsheka; Malebogo Kebabonye; Thongbotho Mphoyakgosi
EPI_ISL_2161032, EPI_ISL_2161167	National Hematology and Transfusiology Center	National Hematology and Transfusiology Center	Aghayev AR
EPI_ISL_486888	National Influenza Center, Bahrain	National Influenza Center, Bahrain	AlWasti, H.; Altaif, Z.; Shehab, F.; Zaed, A.
EPI_ISL_421671	National Influenza Center, Indian Council of Medical Research - National Institute of Virology	Indian Council of Medical Research-National Institute of Virology, Microbial Containment Complex	Amita Jain; Anita Shete-Aich; Atanu Basu; Bharti Malhotra; Deepika Chaudhary; Dimpal A. Nyayanit; Gajanan Sapkal; Gururaj Deshpande; Hitesh Dighe; Manohar. L. Chaudhary; Padinjaremattathil Thankappan Ullas; Pragya D. Yadav; Priya Abraham; Sarah Cherian; Savita Patil; Sreelekshmy Mohandas; Triparna Majumdar; Varsha Potdar
EPI_ISL_862076, EPI_ISL_862078, EPI_ISL_1014676, EPI_ISL_1014685, EPI_ISL_1014686, EPI_ISL_1993549	National Influenza Center, Virology Department	National Influenza Center	A Nejati; F Ajaminejad; F Ajaminejad and T Mokhtari Azad; J Yavarian; K Sadeghi; N Ghavvami; N Ghavvami and T Mokhtari Azad; NZ Shafiei Jandaghi; V Salimi
EPI_ISL_2285848, EPI_ISL_2285850, EPI_ISL_2285852, EPI_ISL_2285862, EPI_ISL_2285863, EPI_ISL_2535823, EPI_ISL_2535828, EPI_ISL_2535830, EPI_ISL_2535837			
see above	National Influenza Centre	National Influenza Centre	; Benjamiin B. Lindsey; Benjamin H. Foulkes; Dennis Laryea; Ernest Asiedu; Frank Asiedu-Bekoe; Gordon Awandare; Ivy A. Asante; Joseph Oliver-Commey; Joyce Ngoi; Linda Boatemaa; Lorreta Kwasah; Mathew D. Parker; Michael Marks;

			Mildred Adusei-Poku; Sharon Hsu; Thushan I de Silva; William K. Ampofo
EPI_ISL_1910219, EPI_ISL_1910228	National Institute for Food and Veterinary Risk Assessment (NMVRVI)	National Public Health Surveillance Laboratory	Ana Steponkiene; Danas Baksa; Jelena Razmuk; Lukas Vasionis; Lukas Zemaitis; Migle Gabrielaite; Svajune Muralyte
EPI_ISL_469255	National Institute for Viral Disease Control and Prevention, China CDC	Institute of Viral Disease Control and Prevention, China CDC	Chun Huang; Dayan Wang; George F. Gao; Guizhen Wu; Jun Han; Shiwen Wang; Weifeng Shi; Wenbo Xu; William J. Liu; Xiang Zhao , Lijuan Chen; Yanhai Wang; Yao Meng , Zhixiao Chen , Yuchao Wu; Yong Zhang
EPI_ISL_408481	National Institute for Viral Disease Control and Prevention, China CDC	National Institute for Viral Disease Control & Prevention, CCDC	Baoying Huang; Fei Ye; George F. Gao; Guizhen Wu; Hengqin Wang; Li Zhao; Peihua Niu; Roujian Lu; Sheng Ye; Wenbo Xu; Wenjie Tan; Wenling Wang; Xiang Zhao
EPI_ISL_850946, EPI_ISL_850950	National Institute for Viral Disease Control and Prevention, China CDC	National Institute for Viral Disease Control and Prevention, China CDC	Cao Chen; Dayan Wang; Ji Wang; Jun Han; Kai Nie; Shiwen Wang; Weimin Zhou; Wenbo Xu; Wenjie Tan; Xiang Zhao; Yang Song; Yanhai Wang; Yao Meng; Yenan Feng; Yong Zhang; Yuchao Wu; Zhixiao Chen
EPI_ISL_1824609, EPI_ISL_1915521, EPI_ISL_1915538	National Institute of Health Research and Development	National Institute of Health Research and Development	Agustiningsih; Arie Ardiansyah Nugraha; Fauzul Muna; Hana Apsari Pawestri; Hartanti Dian Ikawati; Herna; Holy Arif Wibowo; Irene Lorinda Indalao; Kartika Dewi Puspa; Kindi Adam; Krisna Nur Andriana Pangesti; Natalie Laurencia Kipuw; Nelly Puspandari; Ni Ketut Susilarini; Nike Susanti; Nurika Hariastuti; Reni Herman; Ririn Ramadhany; Subangkit; Tati Febriyanti; Triyani Soekarso; Ully Alfi Nikmah; Vivi Setiawaty.; Yuni Rukminiati
EPI_ISL_1509924, EPI_ISL_1531555, EPI_ISL_1531561, EPI_ISL_2433937, EPI_ISL_2467942	National Institute of Laboratory Medicine and Referral Center	Genomic Research Lab, BCSIR	A. K. M. Shamsuzzaman; Abu Sayeed Mohammad Mahmud; Arifa Akram; Asish Kumar Ghosh; Barna Goswami; Eshrar Osman; Iffat Jahan; Mahmuda Yeasmin; Md. Ahasan Habib; Md. Kamrul Islam; Md. Maruf Ahmed Molla; Md. Murshed Hasan Sarkar; Md. Saddam Hossain; Md. Salim Khan; Mohammad Mohi Uddin; Mohammad Samir Uzzaman; Shahina Akter; Tanjina Akhter Banu; Tasnim Nafisa
EPI_ISL_1588472, EPI_ISL_2420841	National Institute of Public Health	National Institute of Public Health	Alexander Nagy; Dusan Trnka; Helena Jirincova; Jaromira Vecerova; Timotej Suri
EPI_ISL_2357744, EPI_ISL_2562068, EPI_ISL_2562069, EPI_ISL_2562070, EPI_ISL_2562071, EPI_ISL_2562072, EPI_ISL_2562073, EPI_ISL_2620893, EPI_ISL_2620894, EPI_ISL_2620895, EPI_ISL_2620896, EPI_ISL_2620897, EPI_ISL_2674220			
see above	National Institute of Public Health	State Veterinary Institute Prague	A; Cernikov; D; H; J; Jirincova; L; M; Nagy; Stara; Suri; T; Trnka; Vecerova
EPI_ISL_2301430, EPI_ISL_2304141, EPI_ISL_2304142, EPI_ISL_2304143, EPI_ISL_2483841, EPI_ISL_2483846, EPI_ISL_2545435, EPI_ISL_2545447, EPI_ISL_2545452, EPI_ISL_2545453, EPI_ISL_2545454, EPI_ISL_2545461, EPI_ISL_2545470, EPI_ISL_2545473, EPI_ISL_2548370, EPI_ISL_2548539, EPI_ISL_2549196, EPI_ISL_2687944, EPI_ISL_2687960, EPI_ISL_2687968, EPI_ISL_2687977, EPI_ISL_2756206, EPI_ISL_2756208, EPI_ISL_2756210, EPI_ISL_2756211, EPI_ISL_2756215, EPI_ISL_2756216, EPI_ISL_2756217, EPI_ISL_2756218, EPI_ISL_2784722			
see above	National Platform bis UMONS/Jolimont	National Platform bis UMONS/Jolimont	Eric Tarantino; Florian Juszczak; François Dufrasne; Gautier Detry; Guillaume Bayon-Vicente; Ruddy Wattiez
EPI_ISL_1095617, EPI_ISL_1195207, EPI_ISL_1447312, EPI_ISL_1447313, EPI_ISL_1447314	National Public Health Center, COVID Laboratory	National Public Health Center, National Biosafety Laboratory	Bernadett Pályi; Dániel Déri; Judit Henczkó; Norbert Solymosi; Nóra Magyar; Zoltán Kis
EPI_ISL_845561, EPI_ISL_845565	National Public Health Laboratory, Cameroon	African Centre of Excellence for Genomics of Infectious Diseases (ACEGID), Redeemer's University	Oluniyi P.E. et al
EPI_ISL_1122419, EPI_ISL_1122420, EPI_ISL_1122421	National Public Health Laboratory, National Centre for Infectious Diseases	National Virology Reference Laboratory	Lin Cui; Raymond Tzer Pin Lin; Surita Taib; Tze Minn Mak; Zainun Zaini; Zhenyang Zhou
EPI_ISL_1921165, EPI_ISL_1921288, EPI_ISL_1921739, EPI_ISL_1921764, EPI_ISL_1921765, EPI_ISL_1921768	National Public Health Organization	Institute of Applied Biosciences, Centre for Research and Technology Hellas	Anastasia Chatzidimitriou et al.
EPI_ISL_1273391, EPI_ISL_1273392, EPI_ISL_1273394	National Reference Laboratory - Ministry of Health Maseru Lesotho	National Institute for Communicable Diseases of the National Health Laboratory Service	Amoako DG; Banda R; Bhiman JN; Gorova V; Ismail A; Mahlangu B; Mathabo M; Mohale T; Mooko M; Ntuli N; Scheepers C
EPI_ISL_625683, EPI_ISL_833578	National Reference Laboratory for COVID-19, Pasteur Institute of Iran	National Reference Laboratory for COVID-19, Pasteur Institute of Iran	Ahmad Ghasemi; Farideh Niknam; Heasam Nemati; Hessam Nemati; Kayhan Azadmanesh; Mahsa Tavakkoli; Mahsa Tavakoli; Maryam Rostamtabar; Marzieh Sadjadi; Marzieh Sajjadi; Mohamad Sadegh Shams Nosrati; Mohammad Hassan Pouriayevali; Mohammad Mehdi Mortazavipour; Mostafa Salehi-Vaziri; Parastoo Yekta; Sahar Khakifirouz; Sana Eybpoosh; Sanam Azad-Manjiri; Sepideh Gerdooei; Setareh Kashanian; Tahereh Mohammadi; Tahmineh Jalali; Zabihollah Shoja; Zahra Ahmadi; Zahra Fereydouni; Zeynab VeisiZadeh
EPI_ISL_435675	National Virology Reference Laboratory	National Public Health Laboratory, National Centre for Infectious Diseases	Chavatte Jean-Marc; Cui Lin; Lin Raymond Tzer Pin; Mak Tze Minn; Octavia Sophie; Taib Surita; Zaini Zainun
EPI_ISL_605063, EPI_ISL_605069	National Virus Reference Laboratory	Irish Coronavirus Sequencing Consortium - Helixworks	Conor Crosbie; Nimesh Pinnamaneni; Sachin Chalapati
EPI_ISL_578322, EPI_ISL_1191342, EPI_ISL_1398743, EPI_ISL_1398857, EPI_ISL_1500021, EPI_ISL_1577403, EPI_ISL_1620440, EPI_ISL_1651763, EPI_ISL_1731106, EPI_ISL_1731198, EPI_ISL_1731252, EPI_ISL_1731330, EPI_ISL_1785273, EPI_ISL_1785341, EPI_ISL_1890977, EPI_ISL_1890990, EPI_ISL_1904652, EPI_ISL_1904670, EPI_ISL_1904703, EPI_ISL_1904829, EPI_ISL_1904838, EPI_ISL_1911144, EPI_ISL_1960669, EPI_ISL_1960691, EPI_ISL_1960795, EPI_ISL_1960834, EPI_ISL_1960854, EPI_ISL_1972662, EPI_ISL_1972667, EPI_ISL_1972679, EPI_ISL_1972682, EPI_ISL_1972731, EPI_ISL_1972735, EPI_ISL_1972737, EPI_ISL_1972739, EPI_ISL_1972740, EPI_ISL_1972833, EPI_ISL_2029129, EPI_ISL_2029134, EPI_ISL_2029136, EPI_ISL_2029137, EPI_ISL_2029153, EPI_ISL_2029171, EPI_ISL_2029172, EPI_ISL_2029173, EPI_ISL_2029174, EPI_ISL_2029353, EPI_ISL_2029354, EPI_ISL_2087953, EPI_ISL_2088054, EPI_ISL_2088086, EPI_ISL_2131943, EPI_ISL_2131950, EPI_ISL_2132109, EPI_ISL_2132111, EPI_ISL_2132152, EPI_ISL_2132155, EPI_ISL_2193904, EPI_ISL_2193922, EPI_ISL_2240843, EPI_ISL_2240930, EPI_ISL_2284223, EPI_ISL_2284263, EPI_ISL_2284264, EPI_ISL_2284332, EPI_ISL_2284339, EPI_ISL_2284347, EPI_ISL_2284369, EPI_ISL_2284371, EPI_ISL_2284378, EPI_ISL_2284388, EPI_ISL_2363015, EPI_ISL_2363324, EPI_ISL_2363328, EPI_ISL_2363379, EPI_ISL_2363380, EPI_ISL_2455018, EPI_ISL_2533393, EPI_ISL_2533454, EPI_ISL_2601108, EPI_ISL_2601113, EPI_ISL_2601114, EPI_ISL_2601116, EPI_ISL_2601119, EPI_ISL_2601122, EPI_ISL_2601124, EPI_ISL_2601177, EPI_ISL_2710640			
see above	National Virus Reference Laboratory	National Virus Reference Laboratory	Calum Walsh; Charlene Bennet; Charlene Bennett; Cillian F De Gascun; Fiona Crispie; Gabriel Gonzalez; Guerrino Macori; Jonathan Dean; Matthew McCabe; Michael Carr; Paul Cotter; Seamus Fanning; Suzie Coughlan; Zoe Yandle
EPI_ISL_2533833, EPI_ISL_2533834, EPI_ISL_2533835, EPI_ISL_2533836, EPI_ISL_2533839, EPI_ISL_2533845, EPI_ISL_2533846, EPI_ISL_2533849, EPI_ISL_2533850, EPI_ISL_2533864, EPI_ISL_2533869, EPI_ISL_2533870, EPI_ISL_2533871, EPI_ISL_2533872, EPI_ISL_2533873, EPI_ISL_2533874, EPI_ISL_2533876, EPI_ISL_2533879			
see above	Naval Medical Research Unit No. 3	Naval Medical Research Center Biological Defense Research Directorate	Andrea E. Luquette; Andrew J. Bennett; Bishwo N. Adhikari; Catherine E. Arnold; Chaselynn M. Watters; Emily K. Stefanov; Francisco Malagon; Kyle A. Long; Logan J. Voegtly; Luis A. Estrella; Michael V. Deschenes; Regina Z. Cer; Stephen M. Eggan; and Kimberly A. Bishop-Lilly
EPI_ISL_1010709, EPI_ISL_1010714, EPI_ISL_1061034, EPI_ISL_1061036, EPI_ISL_1061037, EPI_ISL_1121977, EPI_ISL_1121978, EPI_ISL_1121982, EPI_ISL_1121984, EPI_ISL_1121985, EPI_ISL_1121988, EPI_ISL_1184094, EPI_ISL_1315066, EPI_ISL_1315070, EPI_ISL_1697280, EPI_ISL_1756027, EPI_ISL_1756028, EPI_ISL_1756030, EPI_ISL_1756032, EPI_ISL_2107443, EPI_ISL_2107459, EPI_ISL_2107499, EPI_ISL_2107500, EPI_ISL_2107504, EPI_ISL_2249257, EPI_ISL_2249258, EPI_ISL_2455226, EPI_ISL_2455227, EPI_ISL_2455228, EPI_ISL_2455229, EPI_ISL_2455230, EPI_ISL_2696274			
see above	New South Wales Health Pathology Royal Prince Alfred Hospital	Microbiology RPAH	Au, J.; Bull, R.; Deveson, I.; Foster, C.; Rawlinson, W.; Ruiz Silva, M.; Van Hal, S.
EPI_ISL_2259700, EPI_ISL_2259713	Niedersächsisches Landesgesundheitsamt (NLGA)	Robert Koch Institute	
EPI_ISL_685307	Niigata Prefectural Institute of Public Health and Environmental Sciences	Pathogen Genomics Center, National Institute of Infectious Diseases	Kentaro Itokawa; Makoto Kuroda; Masanori Hashino; Rina Tanaka; Tsuyoshi Sekizuka
EPI_ISL_2361921, EPI_ISL_2376384, EPI_ISL_2376385	Noguchi Memorial Institute for Medical Research, University of Ghana, Legon, Ghana	Institute of Tropical Medicine, Universitätsklinikum Tübingen, Germany	Abraham Kwabena Anang; Bright Adu; Dorothy Yeboah-Manu; Hilda Opoku Frempong; John Kofi Odoom; Joseph Humphrey Kofi Bonney; Joyce Appiah-Kubi; Keren Okyerebea Attiku; Le Thi Kieu Linh; Quaneeta Mohktar; Sivaramakrishna Rachakonda; Srinivas-reddy Pallerla; Thirumalaisamy P Velavan
EPI_ISL_2363940	NordLab Oulu	Expert Microbiology, National Institute for Health and Welfare	Carita Savolainen-Kopra; Erika Lindh; Haider al-Hello; Jani Halkilahti; Kirsi Liitsola; Niina Ikonen; Pekka Ellonen; Päivi Laurila; Sari Hannula; Soile Blomqvist; Teemu Smura
EPI_ISL_2601630	North Estonia Medical Centre	1. Laboratory of Communicable Diseases (Estonia); 2. Eurofins Genomics Europe Sequencing GmbH	Liidia Dotsenko et al.
EPI_ISL_1637187, EPI_ISL_1730407, EPI_ISL_1978425, EPI_ISL_1978672, EPI_ISL_1978729, EPI_ISL_1978893, EPI_ISL_2126212, EPI_ISL_2126321, EPI_ISL_2126374, EPI_ISL_2126425, EPI_ISL_2239933, EPI_ISL_2239944, EPI_ISL_2240213, EPI_ISL_2240218, EPI_ISL_2356069, EPI_ISL_2434582, EPI_ISL_2434724, EPI_ISL_2518416, EPI_ISL_2518429, EPI_ISL_2518757, EPI_ISL_2518783, EPI_ISL_2518866, EPI_ISL_2518894, EPI_ISL_2518910, EPI_ISL_2623160, EPI_ISL_2705945, EPI_ISL_2706032, EPI_ISL_2706110, EPI_ISL_2706245, EPI_ISL_2706313, EPI_ISL_2706332			
see above	Northumbria University / South Tees Hospitals NHS Foundation Trust / North Cumbria Integrated Care NHS Foundation Trust / North Tees and Hartlepool NHS Foundation Trust / Newcastle Hospitals NHS Foundation Trust	COVID-19 Genomics UK (COG-UK) Consortium	Andrew Nelson; Brendan Payne; Clive Graham; Darren L Smith; Debra Padgett; Edward Barton; Emma Swindells; Garren Scott; Gary Black; Gary Eltringham; Giles S Holt; Greg R Young; Jane Greenaway; Jennifer Collins; John Allan; Joshua Loh; Lynn Dover; Matthew Bashton; Mohammad A Tariq; Paul Baker; Sarah Essex; Steve Liggett; Wen C Yew; Yusri Taha
EPI_ISL_1685365	Noth Estonia Medical Centre	1. Laboratory of Communicable Diseases (Estonia); 2. Eurofins Genomics Europe Sequencing GmbH	Liidia Dotsenko et al.
EPI_ISL_534326	Notre Dame Intermedica Saude AS	Instituto Adolfo Lutz, Interdiciplinary Procedures Center, Strategic Laboratory	Claudia Regina Gonçalves; Claudio Tavares Sacchi; Erica Valessa Ramos Gomes
EPI_ISL_2372342, EPI_ISL_2372361, EPI_ISL_2372381	Nyangabwe Hospital HIV Reference Laboratory	Botswana Harvard HIV Reference Laboratory	Boitumelo Zuze; Botshelo Radibe; Dorcas Maruapula; Joseph Makhema; Keoratile Ntshambiwa; Kgomotso Moruisi; Legodile Kooepile; Madisa Mine; Mosepele Mosepele; Ontlametse T. Bareng; Pamela Smith-Lawrence; Roger Shapiro; Shahin Lockman; Sikhulile Moyo; Simani Gaseitsiwe; Thongbotho Mphoyakgosi; Wonderful T. Choga
EPI_ISL_1904870, EPI_ISL_2017488, EPI_ISL_2017490, EPI_ISL_2178978, EPI_ISL_2402535, EPI_ISL_2402541, EPI_ISL_2544905, EPI_ISL_2544907, EPI_ISL_2773986, EPI_ISL_2773988, EPI_ISL_2773989, EPI_ISL_2773990, EPI_ISL_2773991, EPI_ISL_2773993, EPI_ISL_2773994, EPI_ISL_2773995, EPI_ISL_2773996, EPI_ISL_2773997, EPI_ISL_2773998, EPI_ISL_2773999, EPI_ISL_2774001, EPI_ISL_2774002			
see above	OLVZ Aalst	OLVZ Aalst	Anne Vankeerberghen; Astrid Holderbeke
EPI_ISL_2342007, EPI_ISL_2342009, EPI_ISL_2342023, EPI_ISL_2342034, EPI_ISL_2342044, EPI_ISL_2342050, EPI_ISL_2342111, EPI_ISL_2342263, EPI_ISL_2342264, EPI_ISL_2342280, EPI_ISL_2342358			
see above	Old District Headquarter Hospital, Jharsuguda	Institute of Life Sciences - INSACOG	Ajay Parida; Amol M. Kanampalliwar; Arup Ghosh; Atimukta Jha; INSACOG Consortium; Omprakash Shiriwas; Punit Prasad; Rajeeb Swain; Rupesh Dash; Safal Walia; Sana Fatma; Shifu Aggarwal; Sunil K. Raghav
EPI_ISL_1443671, EPI_ISL_1443679, EPI_ISL_2604869, EPI_ISL_2604870, EPI_ISL_2604871, EPI_ISL_2604872, EPI_ISL_2604882			
see above	Omics Sciences Laboratory	Omics Sciences Laboratory	Darlyn Amaya; Derly Andrade Molina; Emily Sulay Saltos Montalvo; Gabriel Morey León; Juan Carlos Fernández Cadena; Katheryn Sacheri Viteri; Paula Juliana Gavilanes Jarrín; Rubén Armas González
EPI_ISL_1740447, EPI_ISL_1740451	Ontario's COVID-19 Genomics Rapid Response Coalition	McMaster University	Ahmed Draia; Allison McGeer; Andrew G. McArthur; Angel Li; Emily Panousis; Hooman Derakhshani; Jalees Nasir; Kuganya Nirmalarajah; Michael Surette; Patryk Aftanas; Samira Mubareka
EPI_ISL_1040030	Original detection - Virology Unit, Institut Pasteur du Cambodge; Sequencing - US National Institute of Allergy and Infectious Diseases	Virology Unit, Institut Pasteur du Cambodge	Chau Darapheak; Chin Savuth; Erik A Karlsson; Jennifer Bohl; Jessica Manning; Kraing Sidonn; Ly Sovann; Sophana Chea; Sreyngim Lay; Veasna Duong; Yi Sengdoeurn

	Cambodia		
EPI_ISL_1730648, EPI_ISL_1730650, EPI_ISL_1730652, EPI_ISL_1730654, EPI_ISL_1730662, EPI_ISL_1730804, EPI_ISL_1979483, EPI_ISL_1979485, EPI_ISL_2707461			
see above	Originating lab: Wales Specialist Virology Centre Sequencing lab: Pathogen Genomics Unit	Public Health Wales Microbiology Cardiff Wales Specialist Virology Centre	Alec Birchley; Alexander Adams; Amy Gaskin; Angela Marchbank; Bree Gatica-Wilcox; Catherine Moore; Jason Coombes; Joanne Watkins; Joel Southgate; Johnathan Evans; Laura Gifford; Lauren Gilbert; Lee Graham; Malorie Perry; Matthew Bull; Nicole Pacchiarini; Sally Corden; Sara Kumziene-Summerhayes; Sara Rey; Sarah Taylor; Simon Cottrell; Sophie Jones; Tom Connor
EPI_ISL_2376420	Ospedale Magalini	Istituto Zooprofilattico Sperimentale delle Venezie	Adelaide Milani; Alessia Schivo; Alice Fusaro; Ambra Pastori; Annalisa Salviato; Antonia Ricci; Calogero Terregino; Edoardo Giussani; Elisa Palumbo; Erika Giorgia Quaranta; Isabella Monne; Luca Tassoni
EPI_ISL_1919755, EPI_ISL_1919757	Ospedale Santa Caterina Novella	Istituto Zooprofilattico Sperimentale della Puglia e della Basilicata	Bianco A.; Bruno A.; Capozzi L.; Del Sambro L.; Difato L.; Parisi A.; Simone D.
EPI_ISL_1443893	Outre mer	National Reference Center for Viruses of Respiratory Infections, Institut Pasteur, Paris	Angela Brisebarre; Camille Capel; Christophe Malabat; Corinne Maufrais; Etienne Simon-Lorière; Frédéric Lemoine; Louise Lefrançois; Marion Barbet; Maud Vanpeene; Méline Bizard; StéPhanie Guyomard-Rabenirina; Sylvie Behillil; Sylvie van der Werf; Vincent Enouf
EPI_ISL_2660887, EPI_ISL_2660891	PASTEUR MOLECULAR LABORATORY - INSACOG MEGHALAYA	CSIR-Centre for Cellular and Molecular Biology-INSACOG	Amareshwar Vodapalli; Ara Sreenivas; Archana Bharadwaj Siva; B Himasri; Divya Tej Sowpati; E Marak; E Shadap; Karthik Bharadwaj Tallapaka; Lamuk Zaveri; Onkar Kulkarni; Payel Mukherjee; Priya Nurkurthy; R. Lyngdoh; Rakesh K Mishra; Shreekant Verma; Sofia Banu; Sumedha Avadhanula; Tulasi Nagabandi; Valli Nagalakshmi Undamatla; Vidhyadhari Methuku
EPI_ISL_1170952, EPI_ISL_1241839, EPI_ISL_1300528, EPI_ISL_1300534, EPI_ISL_1465881, EPI_ISL_1465882, EPI_ISL_1465883, EPI_ISL_1516885, EPI_ISL_1563660, EPI_ISL_1563670, EPI_ISL_1563675, EPI_ISL_1910856, EPI_ISL_1910859, EPI_ISL_2001058, EPI_ISL_2001060, EPI_ISL_2137035, EPI_ISL_2385968, EPI_ISL_2385970, EPI_ISL_2385971, EPI_ISL_2385973, EPI_ISL_2507084, EPI_ISL_2507085, EPI_ISL_2507087, EPI_ISL_2507088, EPI_ISL_2507090, EPI_ISL_2507091, EPI_ISL_2507096, EPI_ISL_2603822			
see above	PHV-FSS	PHV-FSS	Son Nguyen
EPI_ISL_1824607	PKC Jagakarsa	National Institute of Health Research and Development	Arie Ardiansyah Nugraha; Hana Apsari Pawestri; Hartanti Dian Ikawati; Kartika Dewi Puspa; Krisna Pangesti; Nelly Puspandari; Subangkit; Vivi Setiawaty
EPI_ISL_2342002, EPI_ISL_2342020, EPI_ISL_2342058, EPI_ISL_2342083, EPI_ISL_2342307, EPI_ISL_2342633, EPI_ISL_2342716			
see above	PRM Medical College & Hospital, Baripada	Institute of Life Sciences - INSACOG	Ajay Parida; Amol M. Kanampalliwar; Arup Ghosh; Atimukta Jha; INSACOG Consortium; Omprakash Shiriwas; Punit Prasad; Rajeeb Swain; Rupesh Dash; Safal Walia; Sana Fatma; Shifu Aggarwal; Sunil K. Raghav
EPI_ISL_2345614	PRONTO SOCORRO MUNICIPAL DE PARAIBUNA	Instituto Butantan / ESALQ-Piracicaba	Antonio Jorge Martins; Claudia Renata dos Santos Barros; David Schlesinger; Debora Botequio Moretti; Dimas Tadeu Covas; Elaine Cristina Marqueze; Elaine Vieira Santos; Evandra Strazza Rodrigues; Heidge Fukumasu; Jayme Augusto de Souza-Neto; José Salvatore Leister Patané; Luiz Alcantara; Luiz Lehmann Coutinho; Maria Carolina Elias; Maurício Lacerda Nogueira; Rafael dos Santos Bezerra; Raul Machado Neto; Rejane Maria Tommasini Grotto; Ricardo Haddad; Sandra Coccuzzo Sampaio Vessoni; Simone Kashima; Svetoslav Nanev Slavov; Vincent Louis Viala
EPI_ISL_1824604	PRVKP FK UI	National Institute of Health Research and Development	Arie Ardiansyah Nugraha; Hana Apsari Pawestri; Hartanti Dian Ikawati; Kartika Dewi Puspa; Krisna Pangesti; Nelly Puspandari; Subangkit; Vivi Setiawaty
EPI_ISL_1312707	PSKUS	Latvian Biomedical Research and Study Centre	Davids Fridmanis; Elina Dimina; Guntars Zarins; Ivars Silamikelis; Janis Klovins; Janis Meisters; Janis Pjalkovskis; Jurijs Perevoscikovs; Kaspars Megnis; Laila Silamikele; Lauma Freimane; Laura Ansone; Liga Birzniece; Monta Ustinova; Nikita Zrelovs; Uga Dumpis; Una Krumina; Vita Rovite; Zane Dobele
EPI_ISL_2504097, EPI_ISL_2504098, EPI_ISL_2504105, EPI_ISL_2504109, EPI_ISL_2504111	Palapye Primary Hospital Laboratory	Botswana Harvard HIV Reference Laboratory	Boitumelo Zuze; Botshelo Radibe; Dorcas Maruapula; Joseph Makhema; Keoratile Ntshambiwa; Legodile Kooepile; Letsibogo Gaoraelwe; Madisa Mine; Modisa Motswaledi; Mosepele Mosepele; Ontlametse T. Bareng; Roger Shapiro; Shahin Lockman; Sikhulile Moyo; Simani Gaseitsiwe; Thela Tefelo; Thongbotho Mphoyakgosi; Wonderful T. Choga
EPI_ISL_2484799	Pamela Youde Nethersole Eastern Hospital	Hong Kong Department of Health	Alan K.L. Tsang; Dominic N.C. Tsang; Edman T.K. Lam; Gannon C.K. Mak; Ken H.L. Ng; Peter C.W. Yip; Peter K.C. Cheng; Rickjason C.W. Chan
EPI_ISL_463749	Pasteur Institute of Iran	Rapid Response Team	Afsaneh Zokaei; Ahmad Ghasemi; Ahmad Mahmoudi; Akram Abouie Mehrizi; Ali Maleki; Ali Torabi; Amir Hesam Nemati; Arash Arashkia; Azam Amirian; Azita Eshratkhah mohammadnejad; Elmira Vadaye kheiri; Farideh niknam oskouei; Fatemeh Fotouhi-Chahooki; Hadiseh Shokouhi Targhi; Hajarossadat Ghaderi; Hesam Karimi; Kayhan Azadmanesh; Kazem Baesi; Mahboobeh Rafigh; Mahmood Barati; Mahsa Ghalejoogh; Mahsa Tavakolirad; Mehdi Fazlalipour; Mehdi Rohani; Mehri Habibi; Mina Agharezaei; Mohamad Mahdi Mortazavipour; Mohammad Hassan Pouriayevali; Mohammad Reza Asadi Karam; Mohammad Sadegh Shams Nosrati; Mostafa Salehi-Vaziri; Neda Afzali; Parastoo Yekta Sanati; Saber Esmaeili; Sahar Khakifirouz; Sanam Azad-Mazjiri; Seyed Dawood Mousavi Nasab; Seyedeh Atefe Hosseini; Seyedeh Sahar Bathaeian; Seyedeh Zahra Moravej; Tahereh Mohammadi; Tahmineh Jalali; Zabihollah Shoja; Zahra Ahmadi; Zahra Fereydouni; Zahra Nejatipour
EPI_ISL_420456, EPI_ISL_470851	PathWest Laboratory Medicine WA	PathWest Laboratory Medicine WA	Avram Levy; Chisha Sikazwe; David Smith and David Speers; Jurissa Lang
EPI_ISL_1911192	PathWest Laboratory Medicine WA	PathWest Laboratory Medicine WA Microbial Surveillance UNit	PathWest Laboratory Medicine WA Microbial Surveillance UNit
EPI_ISL_512719, EPI_ISL_512720, EPI_ISL_512753, EPI_ISL_596703, EPI_ISL_596718, EPI_ISL_596764, EPI_ISL_596841, EPI_ISL_596843, EPI_ISL_596867, EPI_ISL_605853, EPI_ISL_672628, EPI_ISL_708790, EPI_ISL_708796, EPI_ISL_794673, EPI_ISL_794710, EPI_ISL_794726, EPI_ISL_810804, EPI_ISL_933795, EPI_ISL_933798, EPI_ISL_1069392, EPI_ISL_1069393, EPI_ISL_1184507, EPI_ISL_1198801, EPI_ISL_1198803, EPI_ISL_1295930, EPI_ISL_1295931, EPI_ISL_1295936, EPI_ISL_1384812, EPI_ISL_1416323, EPI_ISL_1416325, EPI_ISL_1448429, EPI_ISL_1508993, EPI_ISL_1508994, EPI_ISL_1508996, EPI_ISL_1508997, EPI_ISL_1620802, EPI_ISL_1633675, EPI_ISL_1633676, EPI_ISL_1633678, EPI_ISL_1633679, EPI_ISL_1633680, EPI_ISL_1633683, EPI_ISL_1669119, EPI_ISL_1669121, EPI_ISL_1669126, EPI_ISL_1750963, EPI_ISL_1750964, EPI_ISL_1816917, EPI_ISL_1816918, EPI_ISL_1816919, EPI_ISL_1816920, EPI_ISL_1816921, EPI_ISL_1816922, EPI_ISL_1816923, EPI_ISL_1816924, EPI_ISL_1817558, EPI_ISL_1817559, EPI_ISL_1817560, EPI_ISL_1828698, EPI_ISL_1914668, EPI_ISL_1972908, EPI_ISL_1972909, EPI_ISL_1972911, EPI_ISL_1972912, EPI_ISL_2098723, EPI_ISL_2226229, EPI_ISL_2304306, EPI_ISL_2598297			
see above	PathWest Laboratory Medicine WA	PathWest Laboratory Medicine WA Microbial Surveillance Unit	PathWest Laboratory Medicine WA Microbial Surveillance Unit
EPI_ISL_685278, EPI_ISL_687029, EPI_ISL_688097, EPI_ISL_736906, EPI_ISL_894521, EPI_ISL_901183, EPI_ISL_901369, EPI_ISL_902354, EPI_ISL_1430722, EPI_ISL_1432595, EPI_ISL_1927720, EPI_ISL_1932100, EPI_ISL_1932196, EPI_ISL_1932952, EPI_ISL_1933633, EPI_ISL_1934296, EPI_ISL_2327916, EPI_ISL_2328100, EPI_ISL_2329810, EPI_ISL_2330495, EPI_ISL_2335415, EPI_ISL_2337324, EPI_ISL_2768236			
see above	Pathogen Genomics Center, National Institute of Infectious Diseases	Pathogen Genomics Center, National Institute of Infectious Diseases	Hazuka Y Furihata; Kentaro Itokawa; Makoto Kuroda; Masanori Hashino; Masumichi Saito; Naomi Nojiri; Nozomu Hanaoka; Rina Tanaka; Sana Uchikoba; Tsuguto Fujimoto; Tsuyoshi Sekizuka
EPI_ISL_410717, EPI_ISL_414414	Pathology Queensland	Public Health Virology Laboratory	Alyssa Pyke; Amanda De Jong; Andrew Van Den Hurk; Ben Huang; Bixing Huang; Carmel Taylor; David Warrilow; Doris Genge; Elisabeth Gamez; Glen Hewitson; Ian Maxwell Mackay; Inga Sultana; Jamie McMahon; Jean Barcelon; Judy Northill; Mitchell Finger; Natalie Simpson; Neelima Nair; Peter Burtonclay; Peter Moore; Sarah Wheatley; Sean Moody; Sonja Hall-Mendelin; Timothy Gardam; and Frederick Moore; and Frederick Moore.
EPI_ISL_545050	Pathology West - NSW Health Pathology	NSW Health Pathology - Institute of Clinical Pathology and Medical Research; Westmead Hospital; University of Sydney	CIDM-PH et al.
EPI_ISL_2013035	Plateforme Covid IDF, site Broussais	HEGP - Laboratoire de Virologie	David Veyer; Hélène Péré; Julien Puech; Maxime Wack; Nabil Gastli; Nicolas Robillard; Sandrine Imbeaud
EPI_ISL_2346305, EPI_ISL_2346306, EPI_ISL_2346307, EPI_ISL_2397205, EPI_ISL_2397207, EPI_ISL_2521698, EPI_ISL_2521962, EPI_ISL_2521963, EPI_ISL_2521968, EPI_ISL_2521971, EPI_ISL_2521979, EPI_ISL_2532758, EPI_ISL_2600929, EPI_ISL_2600931, EPI_ISL_2600932, EPI_ISL_2600933, EPI_ISL_2600936, EPI_ISL_2600949, EPI_ISL_2600950, EPI_ISL_2673685, EPI_ISL_2673687, EPI_ISL_2673689			
see above	Plateforme de testing Namuroise	Plateforme de testing Namuroise	Degosserie Jonathan; Demars Aurore; Denis Olivier; Lesly NYINKEU K.; Lesly NYINKEU KEMAMEN; Lesly Nyinkeu Kemamen; Maschietto Céline; Mullier François; Nicolas GILLIARD; Nicolas Gilliard; Nobis Chloé; Otto Gaetan
EPI_ISL_1443706, EPI_ISL_1745199, EPI_ISL_2016711, EPI_ISL_2016712, EPI_ISL_2016714, EPI_ISL_2016715, EPI_ISL_2047646, EPI_ISL_2047647, EPI_ISL_2047648, EPI_ISL_2047649, EPI_ISL_2047650, EPI_ISL_2047651, EPI_ISL_2047652, EPI_ISL_2047653, EPI_ISL_2047654, EPI_ISL_2047655, EPI_ISL_2047656, EPI_ISL_2047657, EPI_ISL_2047658, EPI_ISL_2047659, EPI_ISL_2047662, EPI_ISL_2131296, EPI_ISL_2131297, EPI_ISL_2131298, EPI_ISL_2131303, EPI_ISL_2131310, EPI_ISL_2131345, EPI_ISL_2134689, EPI_ISL_2246063, EPI_ISL_2246064, EPI_ISL_2246065, EPI_ISL_2246066, EPI_ISL_2246103, EPI_ISL_2246115, EPI_ISL_2246121, EPI_ISL_2272670, EPI_ISL_2272671, EPI_ISL_2272672, EPI_ISL_2272678, EPI_ISL_2272679, EPI_ISL_2272687, EPI_ISL_2272690, EPI_ISL_2272760, EPI_ISL_2272768, EPI_ISL_2272769, EPI_ISL_2272775, EPI_ISL_2292722, EPI_ISL_2292727, EPI_ISL_2331830, EPI_ISL_2362614, EPI_ISL_2362616, EPI_ISL_2362624, EPI_ISL_2362625, EPI_ISL_2422173, EPI_ISL_2422181, EPI_ISL_2443384, EPI_ISL_2545595, EPI_ISL_2545603, EPI_ISL_2545604, EPI_ISL_2545607, EPI_ISL_2545608, EPI_ISL_2545612, EPI_ISL_2545634, EPI_ISL_2545635, EPI_ISL_2545642, EPI_ISL_2626854, EPI_ISL_2626858, EPI_ISL_2626861, EPI_ISL_2626867, EPI_ISL_2626869, EPI_ISL_2626870, EPI_ISL_2626890, EPI_ISL_2626894, EPI_ISL_2626900, EPI_ISL_2626901, EPI_ISL_2626902, EPI_ISL_2628074, EPI_ISL_2628086, EPI_ISL_2628100, EPI_ISL_2628103, EPI_ISL_2628111, EPI_ISL_2628121, EPI_ISL_2628123, EPI_ISL_2628125, EPI_ISL_2714772, EPI_ISL_2714773, EPI_ISL_2714775, EPI_ISL_2714779, EPI_ISL_2714787, EPI_ISL_2714789, EPI_ISL_2714791, EPI_ISL_2714807, EPI_ISL_2714808, EPI_ISL_2714811, EPI_ISL_2714816, EPI_ISL_2714818, EPI_ISL_2714823, EPI_ISL_2714825, EPI_ISL_2714831, EPI_ISL_2714832, EPI_ISL_2714836, EPI_ISL_2714866, EPI_ISL_2714870, EPI_ISL_2714877, EPI_ISL_2714878, EPI_ISL_2714888, EPI_ISL_2714889, EPI_ISL_2786930			
see above	Platform BIS UZA/UAntwerpen	Labo Klinische Biologie, UZA	Basil Britto Xavier; Christine Lammens; Herman Goossens; Ines Verbesselt; Jasmine Coppens; Kathleen Holemans; Marie Le Mercier; Veerle Matheeussen
EPI_ISL_2178964	Praava Health	Child Health Research Foundation	CHRF Bangladesh Genomics Team; Shafiul Azam; Zaheed Husain
EPI_ISL_1819254, EPI_ISL_2757688	Presidio di Brindisi Di Summa - Perrino	Istituto Zooprofilattico Sperimentale della Puglia e della Basilicata	Bianco A.; Capozzi L.; Del Sambro L.; Difato L.; Giannico A.; Parisi A.; Ridolfi D.; Santoro A.; Simone D.
EPI_ISL_2341679, EPI_ISL_2341692, EPI_ISL_2341693, EPI_ISL_2341708, EPI_ISL_2341709, EPI_ISL_2341721, EPI_ISL_2341722, EPI_ISL_2341724, EPI_ISL_2341790, EPI_ISL_2341862, EPI_ISL_2341863, EPI_ISL_2341864, EPI_ISL_2341866, EPI_ISL_2341867, EPI_ISL_2341869, EPI_ISL_2341871, EPI_ISL_2341872, EPI_ISL_2341875, EPI_ISL_2341876, EPI_ISL_2341880, EPI_ISL_2341881, EPI_ISL_2341883, EPI_ISL_2341885, EPI_ISL_2341887, EPI_ISL_2341889, EPI_ISL_2341890, EPI_ISL_2341891, EPI_ISL_2341892, EPI_ISL_2341896, EPI_ISL_2341897, EPI_ISL_2341898, EPI_ISL_2341899, EPI_ISL_2341900, EPI_ISL_2341901, EPI_ISL_2341902, EPI_ISL_2341904, EPI_ISL_2341905, EPI_ISL_2341906, EPI_ISL_2341907, EPI_ISL_2341909, EPI_ISL_2341910, EPI_ISL_2341911, EPI_ISL_2341912, EPI_ISL_2341913, EPI_ISL_2341914, EPI_ISL_2341920, EPI_ISL_2341921			
see above	Pt. Jawahar Lal Nehru Memorial Medical College, Raipur	Institute of Life Sciences - INSACOG	Ajay Parida; Amol M. Kanampalliwar; Arup Ghosh; Atimukta Jha; INSACOG Consortium; Omprakash Shiriwas; Punit Prasad; Rajeeb Swain; Rupesh Dash; Safal Walia; Sana Fatma; Shifu Aggarwal; Sunil K. Raghav
EPI_ISL_1311998	Public Health Institute of Zagreb County	Croatian Institute of Public Health	Irena Tabain; Ivana Ferenčak
EPI_ISL_849681, EPI_ISL_849682, EPI_ISL_849685, EPI_ISL_849694	Public Health Virology Laboratory, Forensic and Scientific Services (PHV-FSS)	Public Health Virology Laboratory, Forensic and Scientific Services (PHV-FSS)	Son Nguyen et al
EPI_ISL_985234, EPI_ISL_1061309, EPI_ISL_1061310, EPI_ISL_1098644, EPI_ISL_1098645, EPI_ISL_1121040, EPI_ISL_1137503, EPI_ISL_1137505, EPI_ISL_1804614			
see above	Public Health Virology-Forensic and Scientific Services	Public Health Virology-Forensic and Scientific Services	Alyssa T. Pyke et al.; Son Nguyen; Son Nguyen et al
EPI_ISL_962180, EPI_ISL_1273080, EPI_ISL_1306128, EPI_ISL_1306131, EPI_ISL_1306132, EPI_ISL_1306134, EPI_ISL_1404917, EPI_ISL_1465888, EPI_ISL_1483028, EPI_ISL_1483029, EPI_ISL_1483031, EPI_ISL_1516882, EPI_ISL_1516883, EPI_ISL_1516884, EPI_ISL_1633659, EPI_ISL_1754856, EPI_ISL_1754859, EPI_ISL_1754866, EPI_ISL_1754867			
see above	Public Health Virology-Forensic and Scientific Services (PHV-FSS)	Public Health Virology-Forensic and Scientific Services (PHV-FSS)	Son Nguyen; Son Nguyen et al
EPI_ISL_513313	Public Health, United States Air Force School of Aerospace Medicine	Public Health, United States Air Force School of Aerospace Medicine	A.C.; A.K.; A.W.; B.C.; C.R.; Chapleau; Connors; E.A.; Fries; J.R.; Javorina; Lambert; Macias; Meyer; Purves; R.R. and Starr; S.M.
EPI_ISL_574610	Puskesmas Tambora	Eijkman Institute for Molecular Biology, Ministry of Research and Technology/National Agency for Research and Innovation	Amin Soebandrio; David H Muljono; Edison Johar; Frilasita A Yudhaputri; Herawati Sudoyo; Hidayat Trimarsanto; Iskandar A Adnan; Khin Saw Myint; Safarina G Malik; Willy Agustine
EPI_ISL_1055469, EPI_ISL_1055689, EPI_ISL_1055691, EPI_ISL_1055692	QEII Health Sciences Centre	National Microbiology Laboratory (NML)	Anna Majer; Anneliese Landgraff; CanCOGeN's metadata curation team; Dan Gaston; Darian Hole; Elsie Grudeski; Gary Van Domselaar; Grace Seo; Janice Pettipas; Jason LeBlanc; Jennifer Tanner; Kirsten Biggar; Madison Chapel; Morag Graham; Natalie Knox; Nathalie Bastien; Philip Mabon; Public Health Agency of Canada CanCOGeN team; Rhiannon Huzarewich; Russell Mandes; Shari Tyson; Timothy Booth; Todd Hatchette; Yan Li
EPI_ISL_1832826, EPI_ISL_2519075, EPI_ISL_2519268, EPI_ISL_2519546, EPI_ISL_2519813, EPI_ISL_2519976, EPI_ISL_2520006, EPI_ISL_2520053, EPI_ISL_2520132, EPI_ISL_2520136, EPI_ISL_2623665, EPI_ISL_2623697, EPI_ISL_2623698, EPI_ISL_2624029, EPI_ISL_2624085, EPI_ISL_2624196, EPI_ISL_2624375, EPI_ISL_2706713, EPI_ISL_2706718, EPI_ISL_2706880, EPI_ISL_2706986, EPI_ISL_2707011, EPI_ISL_2707038, EPI_ISL_2707078, EPI_ISL_2707279			
see above	Quadram Institute Bioscience	COVID-19 Genomics UK (COG-UK) Consortium	Alexander J Trotter; Alison E. Mather; Alp Aydin; Ana P. Tedim; Anastasia Kolyva; Andrew Bell; Andrew J. Page; Claire Stuart; Dave J. Baker; Gemma L. Kay; John Wain; Justin O'Grady; Leonardo de Oliveira Martins; Lizzie Meadows; Maria Diaz; Mark Webber; Muhammed Yasir; Nabil-Fareed Alikhan; Ngozi Elumogo; Nicholas M. Thomson; Rachael Stanley; Rachel Gilroy; Reenesh Prakash; Samir Dervisevic; Samuel Bloomfield; Steven Rudder; Thanh Le-Viet
EPI_ISL_2484759	Queen Elizabeth Hospital	Hong Kong Department of Health	Alan K.L. Tsang; Dominic N.C. Tsang; Edman T.K. Lam; Gannon C.K. Mak; Ken H.L. Ng; Peter C.W. Yip; Peter K.C. Cheng; Rickjason C.W. Chan
EPI_ISL_2484761	Queen Mary Hospital	Hong Kong Department of Health	Alan K.L. Tsang; Dominic N.C. Tsang; Edman T.K. Lam; Gannon C.K. Mak; Ken H.L. Ng; Peter C.W. Yip; Peter K.C. Cheng; Rickjason C.W. Chan
EPI_ISL_1833002, EPI_ISL_2520497,	Queens Medical Centre, Clinical Microbiology Department / DeepSeq Nottingham	COVID-19 Genomics UK (COG-UK) Consortium	Christopher Moore; Emily Park; Fei Sang; Gemma Clark; Hannah Howson-Wells; Jack Hill; Jayasree Dey; Johnny Debebe; Jonathan Ball; Joseph Chappell; Manjinder Khakh; Matthew Carlisle; Matthew Loose; Michelle M Lister; Nadine Holmes; Patrick McClure; Theocharis Tsoleridis; Timothy Byaruhanga; Vicki M Fleming; Victoria Wright; Wendy Smith

EPI_ISL_2624424, EPI_ISL_2624524			
EPI_ISL_530236, EPI_ISL_530245, EPI_ISL_530254, EPI_ISL_530277	Queensland Health Forensic and Scientific Services, Public Health Virology	Public Health Virology Laboratory, Forensic and Scientific Services, Queensland Health	Son Nguyen et al
EPI_ISL_415642, EPI_ISL_415644	R. G. Lugar Center for Public Health Research, National Center for Disease Control and Public Health (NCDC) of Georgia.	R. G. Lugar Center for Public Health Research, National Center for Disease Control and Public Health (NCDC) of Georgia.	Adam Kotorashvili; Amiran Gamkrelidze.; Ana Papkiauri; Ann Machablishvili; Anna Kasradze; Davit Tsaguria; Ekaterine Khmaladze; Ekaterine Zangaladze; Ekaterine Zhghenti; Giorgi Tomashvili; Gvantsa Brachveli; Gvantsa Chanturia; Irma Burjanadze; Ketevan Sidamonidze; Khatuna Zakhashvili; Lela Sabadze; Lela Urushadze; Magda Dgebuadze; Maia Alkhazashvili; Mari Gavashelidze; Mariam Zakalashvili; Marine Murtskhvaladze; Meri Pantsulaia; Nato Kotaria; Nino Berishvili; Paata Imnadze; Roena Sukhiashvili; Tamar Jashiashvili; Tata Imnadze; Tea Tevdoradze
EPI_ISL_2379315, EPI_ISL_2379316, EPI_ISL_2379318, EPI_ISL_2379320, EPI_ISL_2379321, EPI_ISL_2450340, EPI_ISL_2450341, EPI_ISL_2450342			
see above	RAILWAY	INSACOG-KA, NIMHANS	Ananthapadmanabha Kotambail; Anita S Desai; Anson Kunjumon George; Chetan G K; Chitra Pattabiraman; Darshan Sreenivas; Gautham Arunachal Udupi; Pramada Prasad; V Ravi
EPI_ISL_1940102, EPI_ISL_1940106	RAILWAY	inStem NCBS - INSACOG	Uma Ramakrishnan Dasaradhi Palakodeti Aswin SaiNarain
EPI_ISL_2379347	RAILWAY H	INSACOG-KA, NIMHANS	Ananthapadmanabha Kotambail; Anita S Desai; Anson Kunjumon George; Chetan G K; Chitra Pattabiraman; Darshan Sreenivas; Gautham Arunachal Udupi; Pramada Prasad; V Ravi
EPI_ISL_1940061	RAILWAY H	inStem NCBS - INSACOG	Uma Ramakrishnan Dasaradhi Palakodeti Aswin SaiNarain
EPI_ISL_2450338, EPI_ISL_2728459, EPI_ISL_2728478, EPI_ISL_2728481, EPI_ISL_2728482, EPI_ISL_2728487, EPI_ISL_2728488, EPI_ISL_2728489, EPI_ISL_2728492, EPI_ISL_2728493, EPI_ISL_2728494, EPI_ISL_2728498, EPI_ISL_2728499, EPI_ISL_2728525, EPI_ISL_2728526, EPI_ISL_2728527, EPI_ISL_2728528			
see above	RAILWAY HOSPITAL	INSACOG-KA, NIMHANS	Ananthapadmanabha Kotambail; Anita S Desai; Anson Kunjumon George; Chetan G K; Chitra Pattabiraman; Darshan Sreenivas; Ellango Ramasamy; Gautham Arunachal Udupi; Mahesh Kumar.C.S; Sony Sharma; V Ravi
EPI_ISL_1663529, EPI_ISL_1663534	REGIONAL VRDL, ICMR-RMRC BBSR	Institute of Life Sciences - INSACOG	Ajay Parida; Amol M. Kanampalliwar; Arup Ghosh; Atimukta Jha; INSACOG Consortium; Punit Prasad; Rajeeb Swain; Rupesh Dash; Safal Walia; Shifu Aggarwal; Sunil K. Raghav
EPI_ISL_2450330, EPI_ISL_2450331	RH HOSAHALLI	INSACOG-KA, NIMHANS	Ananthapadmanabha Kotambail; Anita S Desai; Anson Kunjumon George; Chetan G K; Chitra Pattabiraman; Darshan Sreenivas; Gautham Arunachal Udupi; V Ravi
EPI_ISL_2531908, EPI_ISL_2531922	RIIP	National Reference Center for Viruses of Respiratory Infections, Institut Pasteur, Paris	Angela Brisebarre; Camille Capel; Christophe Malabat; Corinne Maufrais; Etienne Simon-Lorière; Frédéric Lemoine; Louise Lefrançois; Marion Barbet; Maud Vanpeene; Méline Bizard; StéPhanie Guyomard-Rabenirina; Sylvie Behillil; Sylvie Van der Werf; Vincent Enouf
EPI_ISL_2614728	RS Grha Kedoya	Eijkman Institute for Molecular Biology, National Research and Innovation Agency	Amin Soebandrio; Edison Johar; Frilasita A Yudhaputri; Hidayat Trimarsanto; Iskandar Adnan; Khin Saw Myint; Lidwina Priliani; Lydia V. Panggalo; Muhammad Rezki Rasyak; Safarina G Malik; Sukma Oktavianthi; Willy Agustine
EPI_ISL_574616	RS Kramat 128	Eijkman Institute for Molecular Biology, Ministry of Research and Technology/National Agency for Research and Innovation	Amin Soebandrio; David H Muljono; Edison Johar; Frilasita A Yudhaputri; Herawati Sudoyo; Hidayat Trimarsanto; Iskandar A Adnan; Khin Saw Myint; Safarina G Malik; Willy Agustine
EPI_ISL_833504	RS MMC, Jakarta, Indonesia	Biosafety Level-3 Laboratory, Indonesian Institute of Sciences (LIPI)	Ade Andriani; Ahmad Fathoni; Ahmad Randy; Andri Wardiana; Anggia Prasetyoputri; Anik Budhi Dharmayanthi; Isa Nuryana; Listiana Oktavia; Ratih Asmana Ningrum; Syam Budi Iryanto
EPI_ISL_2617493	RS Mitra Keluarga Kalideres	National Institute of Health Research and Development	Arie Ardiansyah Nugraha; Hana Apsari Pawestri; Hartanti Dian Ikawati; Kartika Dewi Puspa; Krisna Pangesti; Nelly Puspandari; Subangkit; Triyani Soekarso; Vivi Setiawaty
EPI_ISL_1622425	RS Mitra Keluarga Kelapa Gading	Eijkman Institute for Molecular Biology, Ministry of Research and Technology/National Agency for Research and Innovation	Amin Soebandrio; Edison Johar; Frilasita A Yudhaputri; Hidayat Trimarsanto; Iskandar Adnan; Khin Saw Myint; Lydia V. Panggalo; Muhammad Rezki Rasyak; Safarina G Malik; Sukma Oktavianthi; Willy Agustine
EPI_ISL_2631484	RS Primaya	Indonesian Institute of Sciences (LIPI)/National Agency of Research and Innovation (BRIN) and National Institute of Health Research and Development (NIHRD)	Alfi T. Fathurahman; Andri Wardiana; Anggia Prasetyoputri; Anik B. Dharmayanthi; Asep M. Ridwanulloh; Erwin F. Hasrianda; Hariyatun; Ruby Setiawan; Subangkit; Sugiyono Saputra; Syam B. Iryanto; Vivi Setiawaty; Yayuk Sri Rahayu
EPI_ISL_2262263	RS Umum Kartika Pulomas	National Institute of Health Research and Development	Arie Ardiansyah Nugraha; Hana Apsari Pawestri; Hartanti Dian Ikawati; Kartika Dewi Puspa; Krisna Pangesti; Nelly Puspandari; Subangkit; Triyani Soekarso; Vivi Setiawaty
EPI_ISL_2162130	RSE ON REM "National Center for Biotechnology"	Reference laboratory for the control of viral infections	Aidar Usserbayev; Aknur Mutaliyeva; Andrey Komissarov; Artem Fadeev; Azamat Kenessov; Bekzhan Maikotov; Gaukhar Nussupbayeva; Madina Tleubergenova; Maria Pisareva; Nazym Tleumbetova
EPI_ISL_2614677	RSJ Dr. Soeharto Heerdjan	Eijkman Institute for Molecular Biology, National Research and Innovation Agency	Amin Soebandrio; Edison Johar; Frilasita A Yudhaputri; Hidayat Trimarsanto; Iskandar Adnan; Khin Saw Myint; Lidwina Priliani; Lydia V. Panggalo; Muhammad Rezki Rasyak; Safarina G Malik; Sukma Oktavianthi; Willy Agustine
EPI_ISL_2262283, EPI_ISL_2262285	RSPI Prof. Dr. Sulianti Saroso	National Institute of Health Research and Development	Arie Ardiansyah Nugraha; Hana Apsari Pawestri; Hartanti Dian Ikawati; Kartika Dewi Puspa; Krisna Pangesti; Nelly Puspandari; Subangkit; Triyani Soekarso; Vivi Setiawaty
EPI_ISL_833392	RSU Sayang Bunda, Makasar,Indonesia	Biosafety Level-3 Laboratory, Indonesian Institute of Sciences (LIPI)	Ade Andriani; Ahmad Fathoni; Andri Wardiana; Anggia Prasetyoputri; Anik Budhi Dharmayanthi; Dini Nurdiani; Isa Nuryana; Ratih Asmana Ningrum; Syam Budi Iryanto; Wahyuni
EPI_ISL_2233089	RSUD Abdul Moeis	National Institute of Health Research and Development	Arie Ardiansyah Nugraha; Hana Apsari Pawestri; Hartanti Dian Ikawati; Kartika Dewi Puspa; Krisna Pangesti; Nelly Puspandari; Subangkit; Triyani Soekarso; Vivi Setiawaty
EPI_ISL_2233106	RSUD Arifin Ahmad Riau	National Institute of Health Research and Development	Arie Ardiansyah Nugraha; Fajri Marinda; Hana Apsari Pawestri; Hartanti Dian Ikawati; Kartika Dewi Puspa; Krisna Pangesti; Nelly Puspandari; Subangkit; Triyani Soekarso; Vivi Setiawaty
EPI_ISL_2106979	RSUD Bali Mandara	National Institute of Health Research and Development	Arie Ardiansyah Nugraha; Hana Apsari Pawestri; Hartanti Dian Ikawati; I Wayan Agus Gede Manik Saputra; Kartika Dewi Puspa; Krisna Pangesti; Nelly Puspandari; Subangkit; Triyani Soekarso; Vivi Setiawaty
EPI_ISL_2262294	RSUD Brebes	National Institute of Health Research and Development	Arie Ardiansyah Nugraha; Hana Apsari Pawestri; Hartanti Dian Ikawati; Kartika Dewi Puspa; Krisna Pangesti; Nelly Puspandari; Subangkit; Triyani Soekarso; Vivi Setiawaty
EPI_ISL_2226647	RSUD Dr. Soetomo	Institute of Tropical Disease, Universitas Airlangga	Acub Zaenal; Aldise M Nastri; Gatot Soegiarto; Jezzy R Dewantari; Joni Wahyuhadi; Kazufumi Shimizu; Krisnoadi Rahardjo; Laksmi Wulandari; Maria I Lusida; Neneng D Kurniati; Resti Yudhawati; Rima R Prasetya; Soetjipto; Yasuko Mori
EPI_ISL_2617536	RSUD Kebayoran Baru	National Institute of Health Research and Development	Arie Ardiansyah Nugraha; Hana Apsari Pawestri; Hartanti Dian Ikawati; Kartika Dewi Puspa; Krisna Pangesti; Nelly Puspandari; Subangkit; Triyani Soekarso; Vivi Setiawaty
EPI_ISL_2101276	RSUD Mangusada	Eijkman Institute for Molecular Biology, National Agency for Research and Innovation; Molecular Biology Laboratory, Faculty Medicine and Health Sciences, Warmadewa University	Amin Soebandrio; Edison Johar; Erly Sintya; Frilasita A Yudhaputri; Hidayat Trimarsanto; Ida Ayu Wayan Mahayani; Iskandar Adnan; Khin Saw Myint; Lidwina Priliani; Lydia V. Panggalo; Muhammad Rezki Rasyak; Safarina G Malik; Sri Masyeni; Sukma Oktavianthi; Willy Agustine
EPI_ISL_2502764	RSUD dr. LOEKMONO HADI	Genetics Working Group (Pokja Genetik) Faculty of Medicine, Public Health and Nursing Universitas Gadjah Mada (FK-KMK UGM); Disease Investigation Center Wates Ministry of Agriculture Indonesia; Department of Microbiology FK-KMK UGM; Laboratorium Diagnostik Yayasan Tahija World Mosquito Program (WMP) Yogyakarta Center for Tropical Medicine FK-KMK UGM; Integrated Research Center FK-KMK UGM; Department of Computer Science and Electronics FMIPA UGM; RSUP Dr. Sardjito	Afiahayati; Dwi AA Nugrahaningsih; Dwi Indaryati; Edwin W. Daniwijaya; Eggi Arguni; Endah Supriyati; Gunadi; Hana F Hanifin; Hendra Wibawa; Indarto Sulistiyono; Irene Tania; Kristy Iskandar; Ludhang P. Rizki; Marcellus; Mohamad S. Hakim; Nungki Anggorowati; Pramesti G Dewi; Siswanto; Titik Nuryastuti; Tri Wibawa
EPI_ISL_2661382, EPI_ISL_2661383, EPI_ISL_2661412, EPI_ISL_2661414, EPI_ISL_2661418, EPI_ISL_2661420	RUHS College of Medical Sciences	CSIR-Centre for Cellular and Molecular Biology - INSACOG	Amareshwar Vodapalli; Ara Sreenivas; Archana Bharadwaj Siva; B Himasri; Divya Tej Sowpati; Dr. Gaurav Dalela; Dr. Jitendra Panda; Dr. Nilofer Khayyam; Dr. Raja Babu Panwar; Dr. Rajeev Gupta; Dr. Ramesh Sharma; Dr. Sonali Sharma; Dr. Sudhanshu Kacker; Dr. Vaseem Naheed Baig; Jandhyala Sai Krishna; Karthik Bharadwaj Tallapaka; Lamuk Zaveri; Onkar Kulkarni; Payel Mukherjee; Priya Nurkurthy; Rakesh K Mishra; Shreekant Verma; Sofia Banu; Sumedha Avadhanula; Tulasi Nagabandi; Valli Nagalakshmi Undamatla; Vidhyadhari Methuku
EPI_ISL_637112, EPI_ISL_637113	Rafik Hariri University Hospital	Microbial Pathogenomics Lab	Georgi Merhi; Rita Feghali; Sima Tokajian; Tamara Salloum
EPI_ISL_450509	Rafik Hariri University Hospital	Rafik Hariri University Hospital	Rita Feghali
EPI_ISL_2342004, EPI_ISL_2342013, EPI_ISL_2342054, EPI_ISL_2342064, EPI_ISL_2342066, EPI_ISL_2342067, EPI_ISL_2342298, EPI_ISL_2342611, EPI_ISL_2342664, EPI_ISL_2342682, EPI_ISL_2342706			
see above	Rajendra Institute of Medical Sciences, Ranchi	Institute of Life Sciences - INSACOG	Ajay Parida; Amol M. Kanampalliwar; Arup Ghosh; Atimukta Jha; INSACOG Consortium; Omprakash Shiriwas; Punit Prasad; Rajeeb Swain; Rupesh Dash; Safal Walia; Sana Fatma; Shifu Aggarwal; Sunil K. Raghav
EPI_ISL_1544317, EPI_ISL_1544487, EPI_ISL_1564356, EPI_ISL_1631164, EPI_ISL_1631170, EPI_ISL_1631314, EPI_ISL_1631836, EPI_ISL_1631914, EPI_ISL_1632122, EPI_ISL_1632256, EPI_ISL_1652717, EPI_ISL_1652897, EPI_ISL_1697989, EPI_ISL_1700313, EPI_ISL_1700494, EPI_ISL_1718700, EPI_ISL_1740551, EPI_ISL_1740611, EPI_ISL_1740633, EPI_ISL_1740691, EPI_ISL_1740754, EPI_ISL_1740766, EPI_ISL_1741325, EPI_ISL_1758982, EPI_ISL_1790217, EPI_ISL_1790323, EPI_ISL_1790372, EPI_ISL_1790378, EPI_ISL_1790415, EPI_ISL_1829814, EPI_ISL_1829841, EPI_ISL_1829894, EPI_ISL_1829951, EPI_ISL_1829958, EPI_ISL_1829967, EPI_ISL_1830001, EPI_ISL_1830033, EPI_ISL_1830108, EPI_ISL_1830209, EPI_ISL_1830245, EPI_ISL_1857631, EPI_ISL_1857673, EPI_ISL_1857699, EPI_ISL_1857728, EPI_ISL_1857758, EPI_ISL_1857770, EPI_ISL_1857779, EPI_ISL_1921463, EPI_ISL_1921523, EPI_ISL_1921538, EPI_ISL_1921568, EPI_ISL_1921596, EPI_ISL_1921631, EPI_ISL_1921691, EPI_ISL_1921713, EPI_ISL_1921731, EPI_ISL_1986688, EPI_ISL_1986695, EPI_ISL_1986757, EPI_ISL_1986870, EPI_ISL_1986956, EPI_ISL_1986979, EPI_ISL_1987022, EPI_ISL_1987044, EPI_ISL_1987170, EPI_ISL_1987178, EPI_ISL_1987183, EPI_ISL_1987210, EPI_ISL_1987256, EPI_ISL_1987329, EPI_ISL_1987335, EPI_ISL_1987336, EPI_ISL_2004448, EPI_ISL_2005162, EPI_ISL_2005192, EPI_ISL_2005617, EPI_ISL_2005649, EPI_ISL_2005664, EPI_ISL_2005671, EPI_ISL_2005705, EPI_ISL_2005715, EPI_ISL_2005716, EPI_ISL_2005784, EPI_ISL_2005792, EPI_ISL_2005804, EPI_ISL_2005808, EPI_ISL_2005837, EPI_ISL_2005838, EPI_ISL_2005839, EPI_ISL_2005845, EPI_ISL_2005858, EPI_ISL_2005866, EPI_ISL_2005868, EPI_ISL_2005886, EPI_ISL_2005898, EPI_ISL_2005905, EPI_ISL_2005906, EPI_ISL_2005947, EPI_ISL_2005993, EPI_ISL_2006007, EPI_ISL_2006010, EPI_ISL_2006012, EPI_ISL_2006062			
see above	Randox Laboratories	Wellcome Sanger Institute for the COVID-19 Genomics UK (COG-UK) Consortium	Cordelia Langford; David K. Jackson; Dominic Kwiatkowski; Ewan Harrison; Ian Johnston; Jeffrey Barrett; John Sillitoe on behalf of the Wellcome Sanger Institute COVID-19 Surveillance Team; Randox Laboratories and Alex Alderton; Roberto Amato; Sonia Goncalves
EPI_ISL_2157350, EPI_ISL_2157551, EPI_ISL_2157552	Red - Regional de Vigilancia Genómica del COVID-19	Laboratory of Respiratory Viruses and Measles, Oswaldo Cruz Institute, FIOCRUZ	Alice Sampaio Rocha; Ana Carolina Mendonca; Anna Carolina Paixao; Claudia Díaz; Eilisa Cavalcante Pereira; Fernando Motta; Luciana Appolinario; Marilda Siqueira on behalf of the Fiocruz COVID-19 Genomic Surveillance Network; Mitzi Castro; Paola Resende; Renata Serrano Lopes; Sandra Paola Paz; Taina Venas
EPI_ISL_1415409	Reference Laboratory of the Ministry of Health	Laboratory of Respiratory Viruses and Measles, Oswaldo Cruz Institute, FIOCRUZ	Alice Sampaio Rocha; Ana Carolina Mendonca; Anna Carolina Paixao; Fernando Motta; Indira Martins; Jessica Edwards; Luciana Appolinario; Marilda Siqueira on behalf of the Fiocruz COVID-19 Genomic Surveillance Network; Paola Resende; Renata Serrano Lopes
EPI_ISL_2710003, EPI_ISL_2710004, EPI_ISL_2710008, EPI_ISL_2710009	Regional VRDL,Dibrugarh	Regional VRDL,Dibrugarh	A Jakharia; B Borkakoty; C Bhattacharya; D Biswas; K Narain; Mandakini Das Sarma; R Hazarika
EPI_ISL_2741538, EPI_ISL_2741628, EPI_ISL_2741632, EPI_ISL_2741638, EPI_ISL_2741640, EPI_ISL_2741643, EPI_ISL_2741757, EPI_ISL_2742160, EPI_ISL_2742191, EPI_ISL_2742194, EPI_ISL_2742204, EPI_ISL_2742225, EPI_ISL_2742228, EPI_ISL_2742229, EPI_ISL_2742231, EPI_ISL_2742235, EPI_ISL_2742236, EPI_ISL_2742253, EPI_ISL_2742254, EPI_ISL_2742262, EPI_ISL_2742263, EPI_ISL_2742267, EPI_ISL_2742521, EPI_ISL_2743257, EPI_ISL_2743440, EPI_ISL_2743680, EPI_ISL_2743905, EPI_ISL_2745306, EPI_ISL_2745630, EPI_ISL_2746375, EPI_ISL_2746491, EPI_ISL_2746623, EPI_ISL_2746695, EPI_ISL_2746847, EPI_ISL_2747197, EPI_ISL_2747466, EPI_ISL_2748367, EPI_ISL_2748451, EPI_ISL_2748462, EPI_ISL_2748648, EPI_ISL_2748702, EPI_ISL_2749454, EPI_ISL_2749456, EPI_ISL_2749610, EPI_ISL_2749622, EPI_ISL_2749647, EPI_ISL_2749686, EPI_ISL_2749694			
see above	Respiratory Virus Unit, Microbiology Services Colindale, Public Health England	COVID-19 Genomics UK (COG-UK) Consortium	PHE Covid Sequencing Team
EPI_ISL_407071	Respiratory Virus Unit, Microbiology Services Colindale, Public Health England	Respiratory Virus Unit, Microbiology Services Colindale, Public Health England	Angie Lackenby; Joanna Ellis; Jonathan Hubb; Kirstin Edwards; Leena Bhaw; Maria Zambon; Monica Galiano; Omolola Akinbami; Richard Myers; Shahjahan Miah; Tiina Talts

EPI_ISL_1590137	Riga East University Hospital-National Microbiology Reference Laboratory; Eurofins Genomics Europe Sequencing GmbH	Riga East University Hospital-National Microbiology Reference Laboratory; Eurofins Genomics Europe Sequencing GmbH	Arzu Algulieva; Diāna Dusacka; Dārta Pūpola; Ilva Pole; Reinis Vangravs; Reinis Zeltmatis; Sergejs Nikisins; Ģirts Šķenders
EPI_ISL_521860, EPI_ISL_521861, EPI_ISL_779409, EPI_ISL_779410, EPI_ISL_779413, EPI_ISL_2321157, EPI_ISL_2321158, EPI_ISL_2321160, EPI_ISL_2321162, EPI_ISL_2321164, EPI_ISL_2321165, EPI_ISL_2321166, EPI_ISL_2321168, EPI_ISL_2321169, EPI_ISL_2379269			
see above	Royal Darwin Hospital Pathology	MDU-PHL	Caly L.; Druce J.; M.L.; Meumann, E.; N.L.; Sait; Sait, M.; Schultz M.; Seemann T.; Sherry; Sherry, N.
EPI_ISL_2250189, EPI_ISL_2250190, EPI_ISL_2250191, EPI_ISL_2250193, EPI_ISL_2250196, EPI_ISL_2250197, EPI_ISL_2250198, EPI_ISL_2250199, EPI_ISL_2250200, EPI_ISL_2250202, EPI_ISL_2250204, EPI_ISL_2250206, EPI_ISL_2250207, EPI_ISL_2250208, EPI_ISL_2250209, EPI_ISL_2250210, EPI_ISL_2250211, EPI_ISL_2250216, EPI_ISL_2250217, EPI_ISL_2250218, EPI_ISL_2250219, EPI_ISL_2250220			
see above	Royal Darwin Hospital Pathology	Microbiological Diagnostic Unit Public Health Laboratory (MDU-PHL)	Caly L.; Druce J.; M.L.; Meumann, E.; N.L.; Sait; Seemann T.; Sherry
EPI_ISL_577607	Royal Hobart Hospital	Royal Hobart Hospital	Cooley L.; van Haeften R.
EPI_ISL_522621, EPI_ISL_522686, EPI_ISL_522688, EPI_ISL_522711	Royal Hobart Hospital Microbiology Department	MDU-PHL	Cooley L.; M.B.; Sait M.; Schultz; Seemann T.; Sherry N.; van Haeften R.
EPI_ISL_1969246, EPI_ISL_1969247, EPI_ISL_1969248	Rumah Sakit Umum Daerah Palangkaraya	National Institute of Health Research and Development	Arie Ardiansyah Nugraha; Hana Apsari Pawestri; Hartanti Dian Ikawati; Kartika Dewi Puspa; Krisna Pangesti; Nelly Puspandari; Subangkit; Triyani Soekarso; Vivi Setiawaty
EPI_ISL_2341681, EPI_ISL_2341685, EPI_ISL_2341697, EPI_ISL_2341714, EPI_ISL_2341717, EPI_ISL_2341728, EPI_ISL_2341774, EPI_ISL_2341795, EPI_ISL_2341852, EPI_ISL_2341853, EPI_ISL_2341855, EPI_ISL_2341856, EPI_ISL_2341859, EPI_ISL_2341938			
see above	S.C.B. Medical College and Hospital, Cuttack	Institute of Life Sciences - INSACOG	Ajay Parida; Amol M. Kanampalliwar; Arup Ghosh; Atimukta Jha; INSACOG Consortium; Omprakash Shiriwas; Punit Prasad; Rajeeb Swain; Rupesh Dash; Safal Walia; Sana Fatma; Shifu Aggarwal; Sunil K. Raghav
EPI_ISL_467989, EPI_ISL_468032, EPI_ISL_468037, EPI_ISL_508605, EPI_ISL_510544, EPI_ISL_513913, EPI_ISL_603125, EPI_ISL_622764, EPI_ISL_654799, EPI_ISL_732960, EPI_ISL_812357, EPI_ISL_812358, EPI_ISL_1029956, EPI_ISL_1701832, EPI_ISL_1704673, EPI_ISL_1704675, EPI_ISL_1704676, EPI_ISL_1704677, EPI_ISL_1704678, EPI_ISL_1704786, EPI_ISL_1704787, EPI_ISL_1704788, EPI_ISL_1704789, EPI_ISL_1704790, EPI_ISL_1704791, EPI_ISL_1704792, EPI_ISL_1704793, EPI_ISL_1704794, EPI_ISL_1704798, EPI_ISL_1704799, EPI_ISL_1704802, EPI_ISL_1704803, EPI_ISL_1704808, EPI_ISL_1706365, EPI_ISL_1706367, EPI_ISL_2462337, EPI_ISL_2462338, EPI_ISL_2462342, EPI_ISL_2462343, EPI_ISL_2462344, EPI_ISL_2462345, EPI_ISL_2462346, EPI_ISL_2462348, EPI_ISL_2462349, EPI_ISL_2462352, EPI_ISL_2462353, EPI_ISL_2462356, EPI_ISL_2462357, EPI_ISL_2462358, EPI_ISL_2462359, EPI_ISL_2462361, EPI_ISL_2462362, EPI_ISL_2462363, EPI_ISL_2462364, EPI_ISL_2462365, EPI_ISL_2462366, EPI_ISL_2462367, EPI_ISL_2462368			
see above	SA Pathology	SA Pathology	Caitlin Selway; Chuan Kok Lim; Geoff Higgins; Ivan Bastian; Julien Soubrier; Karin Kassahn; Lex Leong; Mark Turra; Song Gao
EPI_ISL_455603	SA Pathology	VPRL	Beard, MR.; C.K.; Coldbeck-Shackley, R.; Kirby, E.; L.E.X.; Leong; Lim; Llamas, B.; Merrett, J.; Shue, B.; Van Der Hoek, K.
EPI_ISL_2450337	SANJAY GANDHI INSTITUTE OF TRAUMA AND ORTHOPAEDICS	INSACOG-KA, NIMHANS	Ananthapadmanabha Kotambail; Anita S Desai; Anson Kunjumon George; Chetan G K; Chitra Pattabiraman; Darshan Sreenivas; Gautham Arunachal Udupi; V Ravi
EPI_ISL_1927305, EPI_ISL_1927400, EPI_ISL_1927401, EPI_ISL_1927403, EPI_ISL_1927404, EPI_ISL_1927405, EPI_ISL_1927407, EPI_ISL_1927408, EPI_ISL_1927410, EPI_ISL_1927411, EPI_ISL_1927413, EPI_ISL_1927414, EPI_ISL_1927416, EPI_ISL_1927417, EPI_ISL_1927419, EPI_ISL_1927420, EPI_ISL_1927422, EPI_ISL_1927423, EPI_ISL_1927424, EPI_ISL_1927426, EPI_ISL_2131667, EPI_ISL_2131671, EPI_ISL_2131676, EPI_ISL_2131677, EPI_ISL_2131691, EPI_ISL_2131692, EPI_ISL_2131693, EPI_ISL_2131694, EPI_ISL_2131695, EPI_ISL_2131696, EPI_ISL_2131697, EPI_ISL_2131698, EPI_ISL_2131699, EPI_ISL_2131701, EPI_ISL_2131702, EPI_ISL_2131703, EPI_ISL_2131704, EPI_ISL_2131705, EPI_ISL_2131706, EPI_ISL_2131707, EPI_ISL_2131708, EPI_ISL_2131709, EPI_ISL_2131710, EPI_ISL_2131711, EPI_ISL_2131712, EPI_ISL_2131714, EPI_ISL_2131715, EPI_ISL_2131716, EPI_ISL_2131717, EPI_ISL_2131718, EPI_ISL_2131719, EPI_ISL_2131730, EPI_ISL_2131734, EPI_ISL_2131769, EPI_ISL_2253119, EPI_ISL_2253124, EPI_ISL_2330685, EPI_ISL_2331140, EPI_ISL_2337175, EPI_ISL_2621766, EPI_ISL_2621779, EPI_ISL_2770206			
see above	SARS-CoV-2 testing team, National Institute of Infectious Diseases	Pathogen Genomics Center, National Institute of Infectious Diseases	Hazuka Y Furihata; Hiromizu Takahashi; Kentaro Itokawa; Makoto Kuroda; Masanori Hashino; Masumichi Saito; Naomi Nojiri; Nozomu Hanaoka; Rina Tanaka; Sana Uchikoba; Tsuguto Fujimoto; Tsuyoshi Sekizuka
EPI_ISL_1647029	SC (UCO) Igiene e Sanità Pubblica (funzione integrata con SC Microbiologia e Virologia)	ARGO Laboratorio Genomica ed Epigenomica	D'Agaro P; Dal Monego S; Degasperi M; Licastro D; Lombardo F
EPI_ISL_2209145	SECRETARIA MUNICIPAL DE SAUDE DE CORDEIROPOLIS	Instituto Butantan / FZEA-USP-Pirassununga	Antonio Jorge Martins; Claudia Renata dos Santos Barros; David Schlesinger; Debora Botequio Moretti; Dimas Tadeu Covas; Elaine Cristina Marqueze; Elaine Vieira Santos; Evandra Strazza Rodrigues; Heidge Fukumasu; Jayme Augusto de Souza-Neto; José Salvatore Leister Patané; Luiz Alcantara; Luiz Lehmann Coutinho; Maria Carolina Elias; Maurício Lacerda Nogueira; Rafael dos Santos Bezerra; Raul Machado Neto; Rejane Maria Tommasini Grotto; Ricardo Haddad; Sandra Coccuzzo Sampaio Vessoni; Simone Kashima; Svetoslav Nanev Slavov; Vincent Louis Viala
EPI_ISL_2015901, EPI_ISL_2015915	SELARL MIRIALIS CLUSES	CNR Virus des Infections Respiratoires - France SUD	Antonin Bal; Bruno Lina; Gregory Destras; Gwendolyne Burfin; Hadrien Regue; Laurence Josset; Martine Valette; Quentin Semanas
EPI_ISL_2308605	SIESP DIPARTIMENTO DI PREVENZIONE CHIETI	Istituto Zooprofilattico Sperimentale dell'Abruzzo e Molise "G. Caporale"	Ancora M; Calistri P; Cammà C; Caporale M; Curini V; Delli Compagni E; Di Domenico M; Di Lollo Valeria; Di Pasquale A; Lorusso A; Mangone I; Marcacci M; Puglia I; Rinaldi A; Savini G; Scialabba S
EPI_ISL_2536217, EPI_ISL_2536220	SISP Lecce	University of Bari Biomedical Sciences and Human Oncology	Accogli M.; Chironna M.; Loconsole D.; Maria Chironna; Sallustio A.
EPI_ISL_2562991, EPI_ISL_2563614, EPI_ISL_2565476, EPI_ISL_2586557, EPI_ISL_2586939, EPI_ISL_2587054, EPI_ISL_2587530			
see above	SK-Roy Romanow Provincial Laboratory	National Microbiology Laboratory (NML)	Amanda Lang; Anna Majer; Anneliese Landgraff; CanCOGeN's metadata curation team; Darian Hole; Elsie Grudeski; Gary Van Domselaar; Grace Seo; Jennifer Tanner; Jessica Minion; Kirsten Biggar; Madison Chapel; Morag Graham; Natalie Knox; Nathalie Bastien; Philip Mabon; Public Health Agency of Canada CanCOGeN team; Rachel DePaulo; Rhiannon Huzarewich; Russell Mandes; Ryan McDonald; Shari Tyson; Timothy Booth; Yan Li
EPI_ISL_2341727	SLN Medical College and Hospital, Koraput	Institute of Life Sciences - INSACOG	Ajay Parida; Amol M. Kanampalliwar; Arup Ghosh; Atimukta Jha; INSACOG Consortium; Omprakash Shiriwas; Punit Prasad; Rajeeb Swain; Rupesh Dash; Safal Walia; Sana Fatma; Shifu Aggarwal; Sunil K. Raghav
EPI_ISL_2728471	SRI JAYADEVA INSTITUTE OF CARDIOLOGY	INSACOG-KA, NIMHANS	Ananthapadmanabha Kotambail; Anita S Desai; Anson Kunjumon George; Chetan G K; Chitra Pattabiraman; Darshan Sreenivas; Ellango Ramasamy; Gautham Arunachal Udupi; Mahesh Kumar.C.S; Sony Sharma; V Ravi
EPI_ISL_1939973	SUBRAMANYA NAGAR	inStem NCBS - INSACOG	Uma Ramakrishnan Dasaradhi Palakodeti Aswin SaiNarain
EPI_ISL_1939974	SUBRAMNAYA NAGAR	inStem NCBS - INSACOG	Uma Ramakrishnan Dasaradhi Palakodeti Aswin SaiNarain
EPI_ISL_1171610, EPI_ISL_1493029, EPI_ISL_1671957, EPI_ISL_1919655, EPI_ISL_2003304, EPI_ISL_2348573, EPI_ISL_2382812, EPI_ISL_2402378, EPI_ISL_2402390, EPI_ISL_2402399, EPI_ISL_2402443, EPI_ISL_2426583, EPI_ISL_2426585, EPI_ISL_2426596, EPI_ISL_2426602, EPI_ISL_2426608, EPI_ISL_2426614, EPI_ISL_2426618, EPI_ISL_2483893, EPI_ISL_2483899, EPI_ISL_2483900, EPI_ISL_2483903, EPI_ISL_2483905, EPI_ISL_2483909, EPI_ISL_2483911, EPI_ISL_2483921, EPI_ISL_2483927, EPI_ISL_2483931, EPI_ISL_2612770, EPI_ISL_2612771, EPI_ISL_2612772, EPI_ISL_2612773, EPI_ISL_2612774, EPI_ISL_2612775, EPI_ISL_2612776, EPI_ISL_2612777, EPI_ISL_2612786, EPI_ISL_2612787, EPI_ISL_2612788, EPI_ISL_2612789, EPI_ISL_2612791, EPI_ISL_2612792, EPI_ISL_2612794, EPI_ISL_2612796, EPI_ISL_2612797, EPI_ISL_2612798, EPI_ISL_2612805, EPI_ISL_2612837, EPI_ISL_2612838, EPI_ISL_2685852, EPI_ISL_2685853, EPI_ISL_2685856, EPI_ISL_2685858, EPI_ISL_2685859, EPI_ISL_2685870, EPI_ISL_2685873, EPI_ISL_2685882, EPI_ISL_2685884, EPI_ISL_2685885, EPI_ISL_2685898, EPI_ISL_2728623, EPI_ISL_2728627, EPI_ISL_2728630, EPI_ISL_2728632, EPI_ISL_2728633, EPI_ISL_2728638, EPI_ISL_2728642, EPI_ISL_2728643, EPI_ISL_2728645			
see above	SYNLAB	GIGA Medical Genomics	Bouchra Boujemla; Claire Gourzonès; Cécile Meex; Keith Durkin; Laurent Gillet; Maria Artesi; Marie-Pierre Hayette; Nadine Cambisano; Nathalie Renotte; Olivier Ek; Pierrette Melin; Raphaël Boreux; Sébastien Bontems; Vincent Bours
EPI_ISL_1565666, EPI_ISL_1572788, EPI_ISL_2117300, EPI_ISL_2128376	SYNLAB Jena Oncoscreen	Robert Koch Institute	
EPI_ISL_1847907	SYNLAB Labor MÃ¼nchen Zentrum LMZ	Robert Koch Institute	
EPI_ISL_1565909, EPI_ISL_1572490	SYNLAB MVZ Ettlingen	Robert Koch Institute	
EPI_ISL_2114976, EPI_ISL_2115085, EPI_ISL_2115100, EPI_ISL_2128794	SYNLAB MVZ Heidelberg	Robert Koch Institute	
EPI_ISL_1352033, EPI_ISL_1441377, EPI_ISL_1847807, EPI_ISL_2112265, EPI_ISL_2264324	SYNLAB MVZ Leinfelden-Echterdingen	Robert Koch Institute	
EPI_ISL_1728331, EPI_ISL_1728761, EPI_ISL_2130398	SYNLAB MVZ Leverkusen	Robert Koch Institute	
EPI_ISL_2507977	SYNLAB Suomi	Expert Microbiology, National Institute for Health and Welfare	Carita Savolainen-Kopra; Erika Lindh; Haider al-Hello; Jani Halkilahti; Kirsi Liitsola; Niina Ikonen; Olli Vapalahti; Pekka Ellonen; Phuoc Truong; Päivi Laurila; Ravi Kant; Sari Hannula; Soile Blomqvist; Teemu Smura
EPI_ISL_1824071, EPI_ISL_2420473	San Gallicano Dermatological Institute I.F.O.	INMI Lazzaro Spallanzani IRCCS	A Di Caro; A Massacci; B Bartolini; CEM Gruber; E Giombini; E Trento; F De Nicola; F Ensoli; F Messina; F Pimpinelli; F Santini; G Bonfiglio; G Orlandi; M Rueca; MR Capobianchi; O Butera
EPI_ISL_1811232	Santo Domingo	Inciensa, Instituto Costarricense de Investigación y Enseñanza en Nutrición y Salud	Pérez-Corrales C & Blanco-Delgado C
EPI_ISL_479800	Sapporo City Institute of Public Health	Pathogen Genomics Center, National Institute of Infectious Diseases	Asami Ohnishi; Hajime Kamiya; Kentaro Itokawa; Makoto Kuroda; Masanori Hashino; Motoi Suzuki; Rina Tanaka; Tsuyoshi Sekizuka
EPI_ISL_1009128, EPI_ISL_1009160	School of Pharmacy	School of Pharmacy	Ahmed Kandeil; Ghazi Kayali; Mina Nabil Kamel; Mohamed A Ali; Rabeh El-Shesheny; Walid Abi Habib
EPI_ISL_1469666	Secretaria Municipal de Saúde de Montenegro	Epiclin	Ana Paula Muterle; Carolina Comerlato; Eliana Márcia Da Ros Wendland; Fernando Hayashi Sant'Anna; Janira Prichula; Juliana Comerlato
EPI_ISL_2589782	Seinäjoen keskussairaala	Expert Microbiology, National Institute for Health and Welfare	Carita Savolainen-Kopra; Erika Lindh; Haider al-Hello; Jani Halkilahti; Kirsi Liitsola; Niina Ikonen; Olli Vapalahti; Pekka Ellonen; Phuoc Truong; Päivi Laurila; Ravi Kant; Sari Hannula; Soile Blomqvist; Teemu Smura
EPI_ISL_849294, EPI_ISL_849319, EPI_ISL_849353, EPI_ISL_856785, EPI_ISL_2135137, EPI_ISL_2135153, EPI_ISL_2135300, EPI_ISL_2135682, EPI_ISL_2135705, EPI_ISL_2136031, EPI_ISL_2136066, EPI_ISL_2136070, EPI_ISL_2136080, EPI_ISL_2136100, EPI_ISL_2136107, EPI_ISL_2136146, EPI_ISL_2140069, EPI_ISL_2140071, EPI_ISL_2158716, EPI_ISL_2158719, EPI_ISL_2158730, EPI_ISL_2158761, EPI_ISL_2158766, EPI_ISL_2158776, EPI_ISL_2158790			
see above	Servicio Virosis Respiratorias-Departamento Virología-INEI	Instituto Nacional Enfermedades Infecciosas C.G.Malbran	Avaro M.; Baumeister E.; Benedetti E.; Campos J.; Cisterna D.; Dattero ME; Lorenzo F.; Molina V.; Perandones C.; Poklepovich T.; Pontoriero A.; Russo M.; Tuduri E.
EPI_ISL_2360252, EPI_ISL_2360254	Shahid Ghodossi Laboratory	National Influenza Center	A Nejati; F Ajaminejad; F Ajaminejad and T Mokhtari Azad; J Yavarian; K Sadeghi; N Ghavvami; N Ghavvami and T Mokhtari Azad; NZ Shafiei Jandaghi; V Salimi
EPI_ISL_2545226, EPI_ISL_2545227, EPI_ISL_2545234, EPI_ISL_2545236, EPI_ISL_2545240, EPI_ISL_2545243, EPI_ISL_2545246, EPI_ISL_2545247, EPI_ISL_2545251, EPI_ISL_2545254, EPI_ISL_2545257, EPI_ISL_2545259, EPI_ISL_2545262, EPI_ISL_2545264, EPI_ISL_2545265, EPI_ISL_2545267, EPI_ISL_2545270, EPI_ISL_2545276, EPI_ISL_2545277, EPI_ISL_2545290, EPI_ISL_2545292, EPI_ISL_2545295, EPI_ISL_2545296, EPI_ISL_2545297, EPI_ISL_2545299, EPI_ISL_2545302, EPI_ISL_2545303, EPI_ISL_2567026, EPI_ISL_2811556, EPI_ISL_2811617, EPI_ISL_2811636, EPI_ISL_2811680			
see above	Shamir Medical Center (Asaf Harofe)	Shamir Medical Center (Asaf Harofe)	Abu Hamad Ramzia; Adina Bar Chaim; Anna Vishnevsky; Chen Weiner; Netta Zuckerman; Nir Rainy; Patricia Benveniste-Lekovitz; Reut Sorek Abramovich; Yevgeni Yegorov
EPI_ISL_2604957	Shared Scientific Laboratory, Research Institute for Biological Safety Problems, RIBSP	Shared Scientific Laboratory, Research Institute for Biological Safety Problems, RIBSP	A.A.; Burashev; K.D.; K.T.; Kozhabergenov; Kutumbetov; L.B.; M.B.; N.S. and Kerimbayev; Orynbayev; Sultankulova; Y.D.; Zakarya
EPI_ISL_2342006, EPI_ISL_2342010, EPI_ISL_2342016, EPI_ISL_2342022, EPI_ISL_2342025, EPI_ISL_2342043, EPI_ISL_2342045, EPI_ISL_2342055, EPI_ISL_2342091, EPI_ISL_2342092, EPI_ISL_2342121, EPI_ISL_2342125, EPI_ISL_2342308, EPI_ISL_2342309, EPI_ISL_2342310, EPI_ISL_2342311, EPI_ISL_2342312, EPI_ISL_2342313, EPI_ISL_2342314, EPI_ISL_2342315, EPI_ISL_2342316, EPI_ISL_2342317, EPI_ISL_2342318, EPI_ISL_2342319, EPI_ISL_2342320, EPI_ISL_2342321, EPI_ISL_2342324, EPI_ISL_2342325, EPI_ISL_2342326, EPI_ISL_2342327			

see above	Sheikh Bhikhari Medical College, Hazaribag	Institute of Life Sciences - INSACOG	Ajay Parida; Amol M. Kanampalliwar; Arup Ghosh; Atimukta Jha; INSACOG Consortium; Omprakash Shiriwas; Punit Prasad; Rajeeb Swain; Rupesh Dash; Safal Walia; Sana Fatma; Shifu Aggarwal; Sunil K. Raghav
EPI_ISL_2405171, EPI_ISL_2405177, EPI_ISL_2500849	Shenzhen Center for Disease Control and Prevention	National Institute for Viral Disease Control and Prevention, China CDC	Can Zhu; ChunLi Wu; DaNa Huang; Hang Zhang; Jiancheng Chen; Le Zuo; Long Chen; Peihua Niu; RenLi Zhang; Renli Zhang; ShaoYu Deng; Shaoyu Deng; WeiHua Wu; Weihua Wu; Xiang Zhao; XiaoLiang Xiao; XinYi Wei; Xinyi Wei; YaQing He; Yaqing He; Yenan Feng; Ying Sun; Yue Li
EPI_ISL_2106239	Siem Reap Provincial Laboratory	Virology Unit, Institut Pasteur du Cambodge	Cecile Troupin; Chau Darapheak; Chin Savuth; Erik A Karlsson; Jurre Y Siegers; Kraing Sidonn; Lamleav Leak; Leakhena Pum; Ly Sovann; Veasna Duong; Yi Sengdoeurn
EPI_ISL_1844241	Sonic - MVZ Medizinisches Labor Bremen GmbH	Robert Koch Institute	
EPI_ISL_490027, EPI_ISL_490040, EPI_ISL_544970, EPI_ISL_593689, EPI_ISL_1098650, EPI_ISL_1098651, EPI_ISL_1098652, EPI_ISL_1383240, EPI_ISL_1383242, EPI_ISL_1383243, EPI_ISL_1904449, EPI_ISL_1904454, EPI_ISL_1904457, EPI_ISL_1904459, EPI_ISL_1904460, EPI_ISL_1904461, EPI_ISL_1904462, EPI_ISL_1904463, EPI_ISL_1904464, EPI_ISL_1904465, EPI_ISL_1904466, EPI_ISL_1904468, EPI_ISL_1904469, EPI_ISL_1904470, EPI_ISL_2405115, EPI_ISL_2462422, EPI_ISL_2462430, EPI_ISL_2462432			
see above	South Eastern Area Laboratory Services (SEALS)	NSW Health Pathology - Institute of Clinical Pathology and Medical Research; Westmead Hospital; University of Sydney	CIDM-PH et al.
EPI_ISL_526200	St Vincent's Pathology (SydPath)	NSW Health Pathology - Institute of Clinical Pathology and Medical Research; Westmead Hospital; University of Sydney	CIDM-PH et al.
EPI_ISL_1731752, EPI_ISL_1731753, EPI_ISL_1731754, EPI_ISL_1731755, EPI_ISL_1914584, EPI_ISL_1914588, EPI_ISL_1914591, EPI_ISL_1914592, EPI_ISL_1914593, EPI_ISL_1914594, EPI_ISL_1914595, EPI_ISL_1914596, EPI_ISL_1914597, EPI_ISL_1914598, EPI_ISL_1969753, EPI_ISL_2098710, EPI_ISL_2098714, EPI_ISL_2098715, EPI_ISL_2098716, EPI_ISL_2098717, EPI_ISL_2098718, EPI_ISL_2098719, EPI_ISL_2098720, EPI_ISL_2098721, EPI_ISL_2098722, EPI_ISL_2307102, EPI_ISL_2307104, EPI_ISL_2307105, EPI_ISL_2307107, EPI_ISL_2307108, EPI_ISL_2307109, EPI_ISL_2307110, EPI_ISL_2307116, EPI_ISL_2307120, EPI_ISL_2307123, EPI_ISL_2307131, EPI_ISL_2307132, EPI_ISL_2307133, EPI_ISL_2620738, EPI_ISL_2620739, EPI_ISL_2620740, EPI_ISL_2620741, EPI_ISL_2620742			
see above	State Virus Research and Diagnostic Laboratory (VRDL), AIIMS Raipur	State Virus Research and Diagnostic Laboratory (VRDL), AIIMS Raipur	Anudita Bhargava; Jiyan Chandrawanshi; Kuldeep Sharma; MD Rafiqullah Khan; Priyanka Singh; Pushpendra Singh; Sanjay Singh Negi; Somya Sharma
EPI_ISL_455072	Sullivan Nicolaides Pathology	NSW Health Pathology - Institute of Clinical Pathology and Medical Research; Westmead Hospital; University of Sydney	CIDM-PH et al.
EPI_ISL_1969243	Swissbel Hotel Airport	National Institute of Health Research and Development	Arie Ardiansyah Nugraha; Hana Apsari Pawestri; Hartanti Dian Ikawati; Kartika Dewi Puspa; Krisna Pangesti; Nelly Puspandari; Subangkit; Triyani Soekarso; Vivi Setiawaty
EPI_ISL_526157, EPI_ISL_526160	Sydney South West Pathology Service (SSWPS) - Liverpool Hospital - NSW Health Pathology	NSW Health Pathology - Institute of Clinical Pathology and Medical Research; Westmead Hospital; University of Sydney	CIDM-PH et al.
EPI_ISL_845799, EPI_ISL_2405111, EPI_ISL_2405112, EPI_ISL_2462421, EPI_ISL_2462429	Sydney South West Pathology Service (SSWPS) - Royal Prince Alfred Hospital - NSW Health Pathology	NSW Health Pathology - Institute of Clinical Pathology and Medical Research; Westmead Hospital; University of Sydney	CIDM-PH et al.
EPI_ISL_1259057, EPI_ISL_1259137, EPI_ISL_1319260, EPI_ISL_1319303, EPI_ISL_1319390, EPI_ISL_2097104, EPI_ISL_2107178, EPI_ISL_2546921, EPI_ISL_2546955, EPI_ISL_2547065			
see above	Synlab Eesti OÜ	1. Laboratory of Communicable Diseases (Estonia); 2. Eurofins Genomics Europe Sequencing GmbH	Liidia Dotsenko et al.
EPI_ISL_1851555	Synlab MVZ Augsburg	Robert Koch Institute	
EPI_ISL_2450349, EPI_ISL_2450350, EPI_ISL_2450352	THE KOLAR MOBILE TEAM	INSACOG-KA, NIMHANS	Ananthapadmanabha Kotambail; Anita S Desai; Anson Kunjumon George; Chetan G K; Chitra Pattabiraman; Darshan Sreenivas; Gautham Arunachal Udupi; V Ravi
EPI_ISL_1827708	TLC	National Institute for Communicable Diseases of the National Health Laboratory Service	Amoako DG; Bhiman JN; Ismail A; Mahlangu B; Maphalala GP; Mohale T; Ntuli N; Scheepers C
EPI_ISL_2363998, EPI_ISL_2364003, EPI_ISL_2364006	TYKS, Kliininen mikrobiologia	Expert Microbiology, National Institute for Health and Welfare	Carita Savolainen-Kopra; Erika Lindh; Haider al-Hello; Jani Halkilahti; Kirsi Liitsola; Niina Ikonen; Pekka Ellonen; Päivi Laurila; Sari Hannula; Soile Blomqvist; Teemu Smura
EPI_ISL_2162143	Taldykorgan Anti-Plague Station	Reference laboratory for the control of viral infections	Aidar Usserbayev; Aknur Mutaliyeva; Andrey Komissarov; Artem Fadeev; Azamat Kenessov; Bekzhan Maikotov; Gaukhar Nussupbayeva; Madina Tleubergenova; Maria Pisareva; Nazym Tleumbetova
EPI_ISL_2341993, EPI_ISL_2341997, EPI_ISL_2342000, EPI_ISL_2342012, EPI_ISL_2342015, EPI_ISL_2342027, EPI_ISL_2342029, EPI_ISL_2342033, EPI_ISL_2342036, EPI_ISL_2342040, EPI_ISL_2342061, EPI_ISL_2342068, EPI_ISL_2342077, EPI_ISL_2342078, EPI_ISL_2342089, EPI_ISL_2342093, EPI_ISL_2342107, EPI_ISL_2342117, EPI_ISL_2342118, EPI_ISL_2342123, EPI_ISL_2342127, EPI_ISL_2342242, EPI_ISL_2342249, EPI_ISL_2342273, EPI_ISL_2342278, EPI_ISL_2342329, EPI_ISL_2342331, EPI_ISL_2342332, EPI_ISL_2342333, EPI_ISL_2342334, EPI_ISL_2342335, EPI_ISL_2342336, EPI_ISL_2342337, EPI_ISL_2342338, EPI_ISL_2342340, EPI_ISL_2342343, EPI_ISL_2342344, EPI_ISL_2342345, EPI_ISL_2342346, EPI_ISL_2342347, EPI_ISL_2342348, EPI_ISL_2342349, EPI_ISL_2342350, EPI_ISL_2342351, EPI_ISL_2342352, EPI_ISL_2342353			
see above	Tata Main Hospital, Jharkhand	Institute of Life Sciences - INSACOG	Ajay Parida; Amol M. Kanampalliwar; Arup Ghosh; Atimukta Jha; INSACOG Consortium; Omprakash Shiriwas; Punit Prasad; Rajeeb Swain; Rupesh Dash; Safal Walia; Sana Fatma; Shifu Aggarwal; Sunil K. Raghav
EPI_ISL_2501033	Tboung Khmum Rapid Response Team	Virology Unit, Institut Pasteur du Cambodge	Cecile Troupin; Chau Darapheak; Chin Savuth; Erik A Karlsson; Jurre Y Siegers; Kraing Sidonn; Leakhena Pum; Ly Sovann; Veasna Duong; Yi Sengdoeurn
EPI_ISL_1591335	Teaching Institute for Public Health	Croatian Institute of Public Health	Irena Tabain; Ivana Ferenčak
EPI_ISL_1443584	Teaching Institute for Public Health of Split-Dalmatia County	Croatian Institute of Public Health	Irena Tabain; Ivana Ferenčak
EPI_ISL_2626555	Teaching Institute of Public Heath of Split- Dalmatia County	Croatian Institute of Public Health	Irena Tabain; Ivana Ferenčak
EPI_ISL_2484762, EPI_ISL_2484765, EPI_ISL_2611664	Temporary Specimen Collection Centre at the AsiaWorld-Expo	Hong Kong Department of Health	Alan K.L. Tsang; Dominic N.C. Tsang; Edman T.K. Lam; Gannon C.K. Mak; Ken H.L. Ng; Peter C.W. Yip; Peter K.C. Cheng; Rickjason C.W. Chan
EPI_ISL_2230692, EPI_ISL_2230693, EPI_ISL_2230694, EPI_ISL_2402558, EPI_ISL_2478980, EPI_ISL_2500850	The Caribbean Public Health Agency	Carrington Lab, Department of PreClinical Sciences, Faculty of Medical Sciences, The University of the West Indies	Adesh Ramsubhag; Aisha Andrewin; Anushka Ramjag; Arianne Brown-Jordan; Avery Hinds; Ayoola Oyinloye; Chinna Chinnadurai; Christine V. F. Carrington; Christopher Oura; Gabriel Escobar; Jaya Jayaraman; Jerome Foster; Karla Georges; Marsha Ivey; Naresh Nandram; Narine Singh; Nikita S. D. Sahadeo; Nuno Faria; Oliver Pybus; Rahul Naidu; Rajini Haraksingh; Risha Singh; Sarah Hill; Stanley Giddings; SueMin Nathaniel; Vernie Ramkissoon
EPI_ISL_2621679, EPI_ISL_2621680	The Caribbean Public Health Agency	Carrington Lab, Department of Preclinical Sciences, Faculty of Medical Sciences, The University of the West Indies	Anushka Ramjag; Arianne Brown-Jordan; Avery Hinds; Christine V. F. Carrington; Christopher Oura; Gabriel Escobar; Karla Georges; Kenneth George; Nikita S. D. Sahadeo; Nuno Faria; Oliver Pybus; Risha Singh; Sarah Hill; SueMin Nathaniel; Vernie Ramkissoon
EPI_ISL_1837991, EPI_ISL_1838012, EPI_ISL_1838023, EPI_ISL_1838042, EPI_ISL_1838045, EPI_ISL_1838063, EPI_ISL_1838073, EPI_ISL_1838080, EPI_ISL_1838083, EPI_ISL_1838086, EPI_ISL_1838109, EPI_ISL_1838114, EPI_ISL_1838123, EPI_ISL_1838125, EPI_ISL_1838134, EPI_ISL_1838151, EPI_ISL_1838194, EPI_ISL_1838217, EPI_ISL_1838225, EPI_ISL_1838229, EPI_ISL_1838260, EPI_ISL_1838262, EPI_ISL_1838265, EPI_ISL_1838282, EPI_ISL_1838285, EPI_ISL_1838292, EPI_ISL_1838312, EPI_ISL_1838315, EPI_ISL_1838322, EPI_ISL_1838327, EPI_ISL_1838337, EPI_ISL_1972350			
see above	The National Centre for Cell Science	CSIR-Centre for Cellular and Molecular Biology-INSACOG	Ajay Pillai; Amareshwar Vodapalli; Ara Sreenivas; Archana Bharadwaj Siva; B Himasri; Blessy B John; Dhiraj Paul; Divya Tej Sowpati; INSACOG Consortium team; Karthik Bharadwaj Tallapaka; Lamuk Zaveri; Manoj Kumar Bhat; Mitali Inamdar; Mohak P Gujare; Onkar Kulkarni; Payel Mukherjee; Rakesh K Mishra; Sharath Chandra Thota; Shivang P. Bhanushali; Shreekant Verma; Sofia Banu; Sonal Manik Chavan; Tulasi Nagabandi; Valli Nagalakshmi Undamatla; Viswagithe S L; Yogesh Shouche
EPI_ISL_584075, EPI_ISL_792695	The National Institute of Public Health	State Veterinary Institute Prague	A; D; H; J; Jirincova; L; Nagy; Novakova; Trnka; Vecerova
EPI_ISL_827293, EPI_ISL_827880, EPI_ISL_830102, EPI_ISL_1586693	The National University Hospital of Iceland	deCODE genetics	Agnar Helgason; Alma Moller; Arna B Agustsdottir; Arnaldur Gylfason; Asgeir Sigurdsson; Aslaug Jonasdottir; Berglind Eiriksdottir; Bjarni Thorbjornsson; Brynjar O Jensson; Daniel F Gudbjartsson; Droplaug N Magnusdottir; Elisabet E Gardarsdottir; Emil A Thorarensen; Gardar Sveinbjornsson; Gisli Masson; Gudmundur Georgsson; Gudmundur L Norddahl; Gudrun Sigmundsdottir; Hakon Jonsson; Hannes Eggertsson; Hilma Holm; Ingileif Jonsdottir; Jona Saemundsdottir; Kamilla S Josefsdottir; Kari Stefansson; Karl G Kristinsson; Kjartan R Gudmundsson; Kristin E Sveinsdottir; Louise le Roux; Maney Sveinsdottir; Olafia S Gretarsdottir; Olafur T Magnusson; Pall Melsted; Patrick Sulem; Run Fridriksdottir; Solvi Rognvaldsson; Thora R Gunnarsdottir; Thordur Kristjansson; Thorolfur Gudnason; Unnur Thorsteinsdottir
EPI_ISL_754232	The Republican Research and Practical Center for Epidemiology and Microbiology (RRPCEM)	WHO National Influenza Centre Russian Federation	Anatoly Krasko; Andrey Komissarov; Anna Ivanova; Artem Fadeev; Daria Danilenko; Dmitry Bazhenov; Dmitry Lioznov; Elena Gasich; Elena Nabieva; Georgii Bazykin; Kirill Bulda; Ksenia Safina; Kseniya Komissarova
EPI_ISL_2033745, EPI_ISL_2350787, EPI_ISL_2350789, EPI_ISL_2350790	Translational Health Science and Technology Institute -ESIC medical college and hospital, Faridabad	THSTI Bioassay laboratory	Akshay Kanakan; Anil Pandey; Guruprasad R Medigeshi; Heena Shaman; Janani Srinivasa Vasudevan; Janmejay Singh; Jigme Wangchuk; Kamal Pargai; Naseem Ahmed Khan; Neha Jha; Priyanka Mehta; Rajesh Pandey; Ranjeet Maurya
EPI_ISL_2497530, EPI_ISL_2625900	UAB "Rezus.lt"	Institute of Biotechnology, Life Sciences Center, Vilnius University	Albertas Timinskas; Alma Gedvilaite; Danguole Ziogiene; Emilija Vasiliunaite; Emilija Vasiliunaite__ Milda Norkiene__ Danguole Ziogiene__ Albertas Timinskas__ Alma Gedvilaite; Milda Norkiene
EPI_ISL_2625896, EPI_ISL_2625909, EPI_ISL_2625921	UAB Medicina practica laboratorija	Institute of Biotechnology, Life Sciences Center, Vilnius University	Emilija Vasiliunaite__ Milda Norkiene__ Danguole Ziogiene__ Albertas Timinskas__ Alma Gedvilaite
EPI_ISL_648124	UHAS COVID-19 Lab	UHAS COVID-19 Lab	John O. Gyapong and the UHAS COVID-19 Lab Team; Jones Gyamfi; Kwabena O. Duedu; Reuben Ayivor-Djanie
EPI_ISL_2376292, EPI_ISL_2376294	ULSS 2 Marca Trevigiana	Istituto Zooprofilattico Sperimentale delle Venezie	Adelaide Milani; Alessia Schivo; Alice Fusaro; Ambra Pastori; Annalisa Salviato; Antonia Ricci; Calogero Terregino; Edoardo Giussani; Elisa Palumbo; Erika Giorgia Quaranta; Isabella Monne; Luca Tassoni
EPI_ISL_2376289, EPI_ISL_2376290, EPI_ISL_2376291, EPI_ISL_2376379, EPI_ISL_2376380, EPI_ISL_2392108	ULSS 3 Venezia	Istituto Zooprofilattico Sperimentale delle Venezie	Adelaide Milani; Alessia Schivo; Alice Fusaro; Ambra Pastori; Annalisa Salviato; Antonia Ricci; Calogero Terregino; Edoardo Giussani; Elisa Palumbo; Erika Giorgia Quaranta; Isabella Monne; Luca Tassoni
EPI_ISL_2376285, EPI_ISL_2376288	ULSS 4 Veneto Orientale	Istituto Zooprofilattico Sperimentale delle Venezie	Adelaide Milani; Alessia Schivo; Alice Fusaro; Ambra Pastori; Annalisa Salviato; Antonia Ricci; Calogero Terregino; Edoardo Giussani; Elisa Palumbo; Erika Giorgia Quaranta; Isabella Monne; Luca Tassoni
EPI_ISL_2376296, EPI_ISL_2376297,	ULSS 8 Berica	Istituto Zooprofilattico Sperimentale delle Venezie	Adelaide Milani; Alessia Schivo; Alice Fusaro; Ambra Pastori; Annalisa Salviato; Antonia Ricci; Calogero Terregino; Edoardo Giussani; Elisa Palumbo; Erika Giorgia Quaranta; Isabella Monne; Luca Tassoni

EPI_ISL_2448428, EPI_ISL_2448429, EPI_ISL_2448430, EPI_ISL_2448618			
EPI_ISL_1623614	UNIBIO ROMANS GAMBETTA	CNR Virus des Infections Respiratoires - France SUD	Antonin Bal; Bruno Lina; Gregory Destras; Gwendolyne Burfin; Hadrien Regue; Laurence Josset; Martine Valette; Quentin Semanas
EPI_ISL_2445463	UNIDADE BASICA SAUDE NATAL ABADIO LACERDA	Instituto Butantan	Antonio Jorge Martins; Claudia Renata dos Santos Barros; David Schlesinger; Debora Botequio Moretti; Dimas Tadeu Covas; Elaine Cristina Marqueze; Elaine Vieira Santos; Evandra Strazza Rodrigues; Heidge Fukumasu; Jayme Augusto de Souza-Neto; José Salvatore Leister Patané; Luiz Alcantara; Luiz Lehmann Coutinho; Maria Carolina Elias; Maurício Lacerda Nogueira; Rafael dos Santos Bezerra; Raul Machado Neto; Rejane Maria Tommasini Grotto; Ricardo Haddad; Sandra Coccuzzo Sampaio Vessoni; Simone Kashima; Svetoslav Nanev Slavov; Vincent Louis Viala
EPI_ISL_1820935	UNIVERSIDAD DE CARTAGENA	Instituto Nacional de Salud- Dirección de Investigación en Salud Pública	Carlos Franco-Muñoz; Carmen Osorio; Christian Romero; Diana Malo; Diego A. Álvarez-Díaz; Diego Andrés Prada; Gerardo Santamaría; Hector Alejandro Ruiz-Moreno; Jhonnatan Reales-González; Jorge Rivera; Juan Camilo Martinez; Julian Naizaque; Katherine Laiton-Donato; Lisseth Pardo; Magdalena Wiesner; Marcela Mercado-Reyes; Maria T. Herrera-Sepúlveda; Marta Lopez Blanco; Martha Lucia Ospina Martinez; Paola Rojas; Patricia del Portillo; Sergio Gomez; Sheryll Corchuelo; Ángela Alarcon Cruz
EPI_ISL_1820926	UNIVERSIDAD DE Magdalena	Instituto Nacional de Salud- Dirección de Investigación en Salud Pública	Carlos Franco-Muñoz; Carmen Osorio; Christian Romero; Diana Malo; Diego A. Álvarez-Díaz; Diego Andrés Prada; Gerardo Santamaría; Hector Alejandro Ruiz-Moreno; Jhonnatan Reales-González; Jorge Rivera; Juan Camilo Martinez; Julian Naizaque; Katherine Laiton-Donato; Lisseth Pardo; Magdalena Wiesner; Marcela Mercado-Reyes; Maria T. Herrera-Sepúlveda; Marta Lopez Blanco; Martha Lucia Ospina Martinez; Paola Rojas; Patricia del Portillo; Sergio Gomez; Sheryll Corchuelo; Ángela Alarcon Cruz
EPI_ISL_1715130	UOC laboratorio di analisi Istituto Giannina Gaslini	TIGEM	Antonio Grimaldi Patrizia Annunziata Francesco Panariello Teresa Giuliano Valentina Bouche Chiara Colantuono Lucio Di Filippo Anna Manfredi Marcello Salvi Andrea Ballabio Davide Cacchiarelli
EPI_ISL_2450332	UPHC GANGONDANAHALLI	INSACOG-KA, NIMHANS	Ananthapadmanabha Kotambail; Anita S Desai; Anson Kunjumon George; Chetan G K; Chitra Pattabiraman; Darshan Sreenivas; Gautham Arunachal Udupi; V Ravi
EPI_ISL_1966102	USF TRES PONTES	Instituto Butantan / Mendelics	Antonio Jorge Martins; Bianca Cechetto Carlos. Mendelics: Bibiana Santos; Claudia Renata dos Santos Barros; Cíntia Bittar; David Schlesinger. Hemocentro Ribeirão Preto: Simone Kashima; Debora Botequio Moretti; Elaine Cristina Marqueze; Elaine Vieira dos Santos; Elisangela Chicaroni Mattos; Erika Freitas; Evandra Strazza Rodrigues; Felipe Allan da Silva da Costa; Flavia Aburjaile; Fábio Sossai Possebon; Guilherme Campos; Guilherme Targino Valente; Heidge Fukumasu. USP-Botucatu: Rejane Maria Tommasini Grotto; Helena Lage Ferreira; Instituto Butantan: Dimas Tadeu Covas; Jardelina de Souza Todao Bernardino; Jayme A. Souza-Neto; Jessika Cristina Chagas Lesbon; Jorge A. Petroli Marchesi; José Salvatore Leister Patané; João Paulo Kitajima; João Pessoa Araújo Jr.; Leila Sabrina Ullmann; Loyze Paola Oliveira de Lima; Luiz Aurelio de Campos Crispin. Centro de Genômica Funcional da ESALQ: Luiz Lehmann Coutinho; Luiz Carlos Junior de Alcantara; Lívia Sacchetto; Maisa C. Pereira Parra; Maria Carolina Elias; Marta Giovanetti; Marília Moraes; Maurício Lacerda Nogueira. Prefeitura de Sao Paulo: Melissa Palmieri.; Patricia Akemi Assato; Paula Rahal; Paulo Inacio da Costa; Rafael dos Santos Bezerra; Raquel de Lello Rocha Campos Cassano. NGS Soluções Genômicas: Pilar Drummond Sampaio Corrêa Mariani. FZEA-USP Pirassununga: Mirele Daiana Poleti; Raul Machado Neto; Ricardo Augusto Brassaloti; Ricardo Haddad; Rodrigo Tocantins Calado. FAMERP-SJRP: Cecília Artico Banho; Sandra Coccuzzo Sampaio; Svetoslav Nanev Slavov; Vagner Fonseca; Vincent Louis Viala
EPI_ISL_1324141, EPI_ISL_1324148	UW Virology Lab	UW Virology Lab	Alexander Greninger; Hong Xie; Keith R Jerome; Lasata Shrestha; Margaret Mills; Meei-Li Huang; Michelle Lin; Noah Baker; Pavitra Roychoudhury; Saraswathi Sathees; Sean Ellis; Shah Mohamed Bakhash
EPI_ISL_734696, EPI_ISL_734730, EPI_ISL_734769	UZ Leuven, National Reference Laboratory for Coronaviruses, Laboratory Medicine, Leuven, Belgium	KU Leuven, Rega Institute, Clinical and Epidemiological Virology	Bert Vanmechelen; Joan Marti-Carerras; Piet Maes; Tony Wawina-Bokalanga
EPI_ISL_1846441	Uniklinikum Carl Gustav Carus an der TU Dresden; Institut fÃ¼r Virologie	Robert Koch Institute	
EPI_ISL_1299861, EPI_ISL_1299871, EPI_ISL_1299884	Unit of lab surveillance of viral emerging diseases, National Lab of Influenza	Respiratory Virus Unit, National Infection Service, Public Health England	Iris Hasibra; PHE Covid Sequencing Team; Prof Albana Fico; Prof Silvia Bino
EPI_ISL_1490709	Unity Health Toronto	Ontario Institute for Cancer Research	Bernard Lam; Felicia Vincelli; Ilinca Lungu; Jared T. Simpson; Jeremy Johns; Karel Boissinot; Larissa M. Matukas; Le Luu; Mark Downing; Paul Krzyzanowski; Philip Zuzarte; Ramzi Fattouh; Richard de Borja; Samira Mubareka; TIBDN; Trina Otterman; Yan Chen
EPI_ISL_523812	Universidad Iberoamericana, Instituto de Medicina Tropical & Salud Global	International Centre for Genetic Engineering and Biotechnology (ICGEB) and ARGO Open Lab Platform	Alejandro Vallejo Degaudenzi; Danilo Licastro; Eileen Riego; Leandro Tapia; Robert Paulino-Ramirez; Simeone Dal Monego; Sreejith Rajasekharan and Alessandro Marcello.; Victor Virgilio Calderon
EPI_ISL_1577026, EPI_ISL_1628474	Universidad Industrial de Santander (Laboratorio Central de Investigaciones - Clínica Chicamocha).	Universidad Industrial de Santander.	Carlos Barrios-Hernández; Carolina S. Torres-Jiménez; Cristian E. Cadena-Caballero; Diego Rueda-Plata; Erika Lizarazo-Gutiérrez; Francisco Martinez-Perez; Lina M. Vera-Cala.; Lizeth J. Forero-Buitrago
EPI_ISL_697796	Universidad Regional Amazonica IKIAM	Institute of Microbiology, Universidad San Francisco de Quito	Andrea Carrera; Belén Prado-Vivar; Bernardo Gutiérrez; Carolina Proaño-Bolaños; Fabian Aguilar; Gabriel Trueba; Giovanna Moran; Juan José Guadalupe; Katherine Apunte; Marcelo Ortiz; Michelle Grunauer; Monica Becerra-Wong; Nina Espinoza de los Monteros; Patricio Rojas-Silva; Paúl Cárdenas; Sonia Sislema; Sully Márquez; Verónica Barragán; Yeimy Rojas
EPI_ISL_2339864, EPI_ISL_2339865, EPI_ISL_2603125	Universidad de Cordoba, Montería, Colombia	Centro de Investigaciones en Microbiología y Biotecnología-UR (CIMBIUR), Facultad de Ciencias Naturales, Universidad del Rosario, Bogotá, Colombia	Ader Alemán; Alejandra Garcia; Alfonso Calderón; Andrés Diaz; Bertha Gastelbondo; Camilo Guzmán; Caty Martinez; Evelin Garay; Germán Arrieta; Hector Contreras; Hector Serrano; Jorge Miranda; José Berrocal; Juan David Ramírez; Ketty Galeano; Luz H. Patiño; Maria Auxiliadora Badillo; Marina Muñoz; Nathalia Ballesteros; Ricardo Rivero; Salim Mattar; Sergio Castañeda; Veronica Contreras; Yesica Botero; Yesica Lopez
EPI_ISL_1582993	Universidad del Atlántico Laboratorio de Investigación en Biología Molecular	Instituto Nacional de Salud- Dirección de Investigación en Salud Pública	Carlos Franco-Muñoz; Carmen Osorio; Diana Malo; Diego A. Álvarez-Díaz; Diego Andrés Prada; Gerardo Santamaría; Hector Alejandro Ruiz-Moreno; Jhonnatan Reales-González; Juan Camilo Martinez; Julian Naizaque; Katherine Laiton-Donato; Lisseth Pardo; Magdalena Wiesner; Marcela Mercado-Reyes; Maria T. Herrera-Sepúlveda; Marta Lopez Blanco; Martha Lucia Ospina Martinez; Paola Rojas; Sergio Gomez; Sheryll Corchuelo; Ángela Alarcon Cruz
EPI_ISL_1730346, EPI_ISL_1832582, EPI_ISL_1832584, EPI_ISL_1832590, EPI_ISL_1832591, EPI_ISL_1832599, EPI_ISL_1832608, EPI_ISL_1832614, EPI_ISL_1832643, EPI_ISL_1832646, EPI_ISL_1975127, EPI_ISL_1975163, EPI_ISL_1975165, EPI_ISL_2517537, EPI_ISL_2517624, EPI_ISL_2517639, EPI_ISL_2517671, EPI_ISL_2517749, EPI_ISL_2622580, EPI_ISL_2622904, EPI_ISL_2705776, EPI_ISL_2705813, EPI_ISL_2723079, EPI_ISL_2723114			
see above	University College London, Great Ormond Street Hospital for Children NHS Foundation Trust, Imperial College Healthcare NHS Trust	COVID-19 Genomics UK (COG-UK) Consortium	Alison Holmes; Charlotte Williams; Helena Tutill; Jacqueline Findlay; James Price; Judith Breuer; Julianne Brown; Kathryn Harris; Leysa Forrest; Mark Kristiansen; Paola Niola; Paola Resende Silva; Patricia Dyal; Paul Randell; Rachel Williams; Samuel Weeks; Sergi Castellano; Sunando Roy; Tony Brooks; Yasmin Panchbhaya
EPI_ISL_1436022	University Hospital Centre Split	Croatian Institute of Public Health	Irena Tabain; Ivana Ferenčak
EPI_ISL_710548	University Hospital Dubrava	Ruđer Boškovic Institute; Forensic Science Centre Ivan Vučetić; University of Zagreb Faculty of Science	Ana Livun; Antonela Blažeković; Boris Maček; Danilo Licastro; Dunja Glavaš; Fran Borovečki; Fuad Ćosović; Gordana Maravić Vlahoviček; Ivan Šamija; Ivana Čelap; Jasna Kašman; Josipa Skelin; Katarina Marija Tupek; Kristian Vlahoviček; Kristina Gotovac Jerećić; Lidija Cvetko-Krajinović; Lucija Basić; Lucija Markulin; Maja Kuzman; Marina Korolija; Mario Štefanović; Mirjana Domazet-Lošo; Paula Štancl; Petra Vrabec; Robert Belužić; Rosa Karlić; Sanja Tadinac; Senčica Pejša; Tomislav Domazet-Lošo; Valentina Đumlijan-Combaj; Vjekoslav Tomaić; Vladimir Krajinović; Željka Mačak Šafranko
EPI_ISL_1832337, EPI_ISL_1832340, EPI_ISL_2125921	University Hospitals Of Leicester NHS Trust and DeepSeq Nottingham	COVID-19 Genomics UK (COG-UK) Consortium	Christopher Holmes; Christopher Moore; Fei Sang; Joeseph Chappell; Johnny Debebe; Jonathan Ball; Julian Tang; Karlie Fallon; Matthew Carlisle; Matthew Loose; Nadine Holmes; Patrick McClure; Paul Bird; Thomas Helmer; Victoria Wright
EPI_ISL_1072985	University of Balamand	Microbial Genomics Lab, Lebanese American University, Byblos	Mira El Chaar; Sima Tokajian; Youssef Bassim
EPI_ISL_1922125	University of Bari Biomedical Sciences and Human Oncology	University of Bari Biomedical Sciences and Human Oncology	Accogli M.; Chironna M.; Loconsole D.; Sallustio A.
EPI_ISL_2365935, EPI_ISL_2365936, EPI_ISL_2365945	University of Bari Biomedical Sciences and Human Oncology-Policlinico	University of Bari Biomedical Sciences and Human Oncology	Accogli M.; Chironna M.; Loconsole D.; Sallustio A.
EPI_ISL_1832153, EPI_ISL_1832157, EPI_ISL_1974723	University of Birmingham	COVID-19 Genomics UK (COG-UK) Consortium	Alex Richter; Andrew Bosworth; Andrew Bosworth. Queen Elizabeth Hospital: Anna Casey; Andrew D Beggs PHE Heartlands Lab: Husam Osman; Charlotte Poxon; Institute of Microbiology; Joanne Stockton; Josh Quick; Kasun Wanigasooriya; Mike Kidd; Nicholas Loman. University of Birmingham Testing Laboratory: Celina M Whalley; Oliver Pickles; Radoslaw Poplawski; Samuel Nicholls; University of Birmingham: Claire McMurray; Will Rowe
EPI_ISL_1970752, EPI_ISL_2003297, EPI_ISL_2003438, EPI_ISL_2139737, EPI_ISL_2245238, EPI_ISL_2289002, EPI_ISL_2382845, EPI_ISL_2382848, EPI_ISL_2382863, EPI_ISL_2382870, EPI_ISL_2483940, EPI_ISL_2500000, EPI_ISL_2500006, EPI_ISL_2500007, EPI_ISL_2500011, EPI_ISL_2500013, EPI_ISL_2612839, EPI_ISL_2728649, EPI_ISL_2728650, EPI_ISL_2728652, EPI_ISL_2728653, EPI_ISL_2728654, EPI_ISL_2728657, EPI_ISL_2728658, EPI_ISL_2728659, EPI_ISL_2728660, EPI_ISL_2728661, EPI_ISL_2728662, EPI_ISL_2728663, EPI_ISL_2728664, EPI_ISL_2728665, EPI_ISL_2728667, EPI_ISL_2728668, EPI_ISL_2728670, EPI_ISL_2728674, EPI_ISL_2728675, EPI_ISL_2728676, EPI_ISL_2728679, EPI_ISL_2728680			
see above	University of Liège COVID-19 testing center	GIGA Medical Genomics	Bouchra Boujemla; Claire Gourzonès; Cécile Meex; Fabrice Bureau; Keith Durkin; Laurent Gillet; Maria Artesi; Marie-Pierre Hayette; Nadine Cambisano; Nathalie Renotte; Olivier Ek; Pierrette Melin; Raphaël Boreux; Sébastien Bontems; Vincent Bours; Wouter Coppieters
EPI_ISL_955144, EPI_ISL_955151, EPI_ISL_1016508, EPI_ISL_1016969	University of Sarajevo, Veterinary Faculty, Laboratory for Molecular Diagnostic and Research Laboratory	University of Sarajevo, Veterinary Faculty, Laboratory for Molecular Diagnostic and Research Laboratory	Alic-Seho A.; Alic-Šeho A.; Alić-Šeho A.; Goletic S.; Goletic T.; Goletic Š.; Goletić T.; Goletić Š.; Hodzic A.; Hodžic A.; Hodžić A.; Jazic A.; Jažic A.; Jažić A.; Nicevic M.; Nicević M.; Sabic E.; Softic A.; Softić A.; Terzic I.; Terzić I.; Terzić I. Jažić A.; Šabic E.; Šabić E.
EPI_ISL_2315667, EPI_ISL_2390189	Universitätsklinikum Bonn - Institut für Virologie	Robert Koch Institute	
EPI_ISL_1355643	Universitätsmedizin Mannheim	Robert Koch Institute	
EPI_ISL_2020930, EPI_ISL_2020931, EPI_ISL_2020932, EPI_ISL_2020933, EPI_ISL_2020934, EPI_ISL_2020935, EPI_ISL_2020936, EPI_ISL_2020937, EPI_ISL_2020938, EPI_ISL_2020939, EPI_ISL_2020940, EPI_ISL_2020941, EPI_ISL_2020942, EPI_ISL_2020943, EPI_ISL_2020944, EPI_ISL_2020945, EPI_ISL_2020946, EPI_ISL_2020947, EPI_ISL_2020948, EPI_ISL_2020949, EPI_ISL_2020950, EPI_ISL_2020951, EPI_ISL_2020952, EPI_ISL_2020953, EPI_ISL_2020954, EPI_ISL_2020955, EPI_ISL_2020956, EPI_ISL_2020957, EPI_ISL_2020958, EPI_ISL_2020959, EPI_ISL_2020960			
see above	Urban Public Health Centre (UPHC), SECTOR-2, Gandhinagar	Gujarat Biotechnology Research Centre	Amit Kanani; Amrutlal Patel; Bhargav Waghela; Bhumika Prajapati; Chaitanya Joshi; Dinesh Kumar; Janvi Raval; Kalpesh Goswami; Madhvi Joshi; Nilen Patel; Nitesh Shah; Nitin Savaliya; Ramesh Pandit; Sonal Sharma; Tejendra Purohit; Twinkle Soni; Umang Mishra; Zarna Patel; Zuber Saiyed
EPI_ISL_2017748	VRDL, Goverment Medical College (GMC) and Hospital, Valsad	Gujarat Biotechnology Research Centre	Chaitanya Joshi; Dinesh Kumar; Janvi Raval; Madhvi Joshi; Nitesh Shah; Nitin Savaliya; Ramesh Pandit; Sonal Sharma; Twinkle Soni; Umang Mishra; Vicky Gandhi; Zarna Patel; Zuber Saiyed
EPI_ISL_2001187, EPI_ISL_2001188, EPI_ISL_2001189, EPI_ISL_2001190, EPI_ISL_2001191, EPI_ISL_2001192, EPI_ISL_2001193, EPI_ISL_2001194, EPI_ISL_2001195, EPI_ISL_2001196, EPI_ISL_2001201, EPI_ISL_2001203, EPI_ISL_2001211, EPI_ISL_2001212, EPI_ISL_2017728, EPI_ISL_2017731, EPI_ISL_2017734, EPI_ISL_2017735, EPI_ISL_2017737, EPI_ISL_2017738, EPI_ISL_2017740, EPI_ISL_2017744, EPI_ISL_2017763, EPI_ISL_2017764			
see above	VRDL, Goverment Medical College (GMC), Surat	Gujarat Biotechnology Research Centre	Chaitanya Joshi; Dinesh Kumar; Janvi Raval; Madhvi Joshi; Neeta Khandelwal; Nitesh Shah; Nitin Savaliya; Ramesh Pandit; Sonal Sharma; Twinkle Soni; Umang Mishra; Zarna Patel; Zuber Saiyed
EPI_ISL_1662284, EPI_ISL_1663498, EPI_ISL_1663501, EPI_ISL_1663502, EPI_ISL_2341666	Veer Surendra Sai Institute of Medical Sciences and Research, Burla, Sambalpur	Institute of Life Sciences - INSACOG	Ajay Parida; Amol M. Kanampalliwar; Arup Ghosh; Atimukta Jha; INSACOG Consortium; Omprakash Shiriwas; Punit Prasad; Rajeeb Swain; Rupesh Dash; Safal Walia; Sana Fatma; Shifu Aggarwal; Sunil K. Raghav
EPI_ISL_426639, EPI_ISL_436113, EPI_ISL_456438, EPI_ISL_456510,	Victorian Infectious Diseases Reference Laboratory (VIDRL)	Microbiological Diagnostic Unit Public Health Laboratory and Victorian Infectious Diseases Reference Laboratory, Doherty Institute	Caly L.; Druce J.; Sait, M.; Schultz M.; Seemann T.; Sherry, N.

EPI_ISL_456524			
EPI_ISL_430695	Victorian Infectious Diseases Reference Laboratory (VIDRL)	Microbiological Diagnostic Unit Public Health Laboratory and Victorian Infectious Diseases Reference Laboratory, The Peter Doherty Institute for Infection and Immunity	Caly L.; Druce J.; Sait, M.; Schultz M.; Seemann T.; Sherry, N.
EPI_ISL_521478, EPI_ISL_522079, EPI_ISL_522121, EPI_ISL_591894, EPI_ISL_854752, EPI_ISL_933779, EPI_ISL_1249993, EPI_ISL_1249996, EPI_ISL_1913199, EPI_ISL_1913200, EPI_ISL_1913201, EPI_ISL_1913202, EPI_ISL_1913203, EPI_ISL_1913204, EPI_ISL_1913205, EPI_ISL_1913206, EPI_ISL_1913207, EPI_ISL_1913208, EPI_ISL_1913209, EPI_ISL_1913212			
see above	Victorian Infectious Diseases Reference Laboratory (VIDRL)	VIDRL and MDU-PHL	Caly L.; Druce J.; M. B.; M.L.; N.L.; Sait; Sait, M.; Schultz; Schultz M.; Seemann T.; Sherry; Sherry, N.
EPI_ISL_416411, EPI_ISL_416415	Victorian Infectious Diseases Reference Laboratory (VIDRL)	Victorian Infectious Diseases Reference Laboratory and Microbiological Diagnostic Unit Public Health Laboratory, Doherty Institute	Caly L.; Druce J.; Schultz M.; Seemann T.; Taiaroa, G.
EPI_ISL_1495574	Vienna COVID-19 Detection Initiative (VCDI)	Bergthaler laboratory, CeMM Research Center for Molecular Medicine of the Austrian Academy of Sciences	Andreas Bergthaler; Anna Schedl; Bekir Erguner; Benedikt Agerer; Christoph Bock; Fabian Amman; Jan Laine; Lukas Endler; Maelle Le Moing; Martin Senekowitsch; Michael Schuster; Petr Triska; Thomas Penz
EPI_ISL_2625915	Viesoji istaiga Klaipedos universitetine ligonine	Institute of Biotechnology, Life Sciences Center, Vilnius University	Emilija Vasiliunaite__ Milda Norkiene__ Danguole Ziogiene__ Albertas Timinskas__ Alma Gedvilaite
EPI_ISL_2427870	Viesoji istaiga Vilniaus universiteto ligonine Santaros klinikos	National Public Health Surveillance Laboratory	Ana Steponkiene; Danas Baksa; Jelena Razmuk; Lukas Vasionis; Lukas Zemaitis; Migle Gabrielaite; Svajune Muralyte
EPI_ISL_2342605	Vikash Multispeciality Hospital, Bargarh	Institute of Life Sciences - INSACOG	Ajay Parida; Amol M. Kanampalliwar; Arup Ghosh; Atimukta Jha; INSACOG Consortium; Omprakash Shiriwas; Punit Prasad; Rajeeb Swain; Rupesh Dash; Safal Walia; Sana Fatma; Shifu Aggarwal; Sunil K. Raghav
EPI_ISL_904945	Vilnius University Hospital Santaros Klinikos, Vilnius University	Institute of Biotechnology, Life Sciences Center, Vilnius University	Albertas Timinskas; Alma Gedvilaite; Aurelija Zvirbliene; Daniel Naumovas; Emilija Vasiliunaite; Laimonas Griskevicius; Milda Norkiene
EPI_ISL_933905	Vilnius university hospital Santaros Klinikos, Center of Laboratory Medicine	Vilnius university hospital Santaros Klinikos, Center of Laboratory Medicine	Daniel Naumovas; Dovile Ezerskyte; Gytis Dudas; Ingrida Olendraite; Justinas Slikas; Rimvydas Norvilas
EPI_ISL_496499, EPI_ISL_591084, EPI_ISL_591085, EPI_ISL_591086, EPI_ISL_965122, EPI_ISL_965123, EPI_ISL_965124, EPI_ISL_1623756, EPI_ISL_1623757, EPI_ISL_1623758, EPI_ISL_1785567, EPI_ISL_1972306, EPI_ISL_1972309, EPI_ISL_1972314, EPI_ISL_1972326, EPI_ISL_2135837, EPI_ISL_2135838, EPI_ISL_2135839, EPI_ISL_2135840, EPI_ISL_2135841, EPI_ISL_2135842, EPI_ISL_2135843, EPI_ISL_2135844, EPI_ISL_2135845, EPI_ISL_2135846			
see above	Viral Respiratory Lab, National Institute for Biomedical Research (INRB)	Pathogen Sequencing Lab, National Institute for Biomedical Research (INRB)	Allison Black; Amuri Aziza; Andrew Rambaut; Catherine Pratt; Eddy Kinganda-Lusamaki; Edith Nkwembe; Emmanuel Lokilo Lofiko; Francisca Muyembe Mawete; Ian Goodfellow; James Hadfield; Jean Claude Makangara; Jean-Claude Makangara Cigolo; Jean-Jacques Muyembe Tamfum; Josh Quick; Kristian Andersen; Matthias Pauthner; Michael Wiley; Nick Loman; Placide Mbala-Kingebeni; Steve Ahuka-Mundeke; Trevor Bedford
EPI_ISL_435404	Virological Research Group, Szentágothai Research Centre	Bioinformatics Research Group, Szentágothai Research Centre	Attila Gyenesei; Endre Gábor Tóth; Ferenc Jakab; Gábor Kemenesi; Péter Urbán; Róbert Herczeg
EPI_ISL_2115511	Virologisches Institut des Universitatsklinikums Erlangen	Robert Koch Institute	
EPI_ISL_1731019, EPI_ISL_1980438, EPI_ISL_2128473, EPI_ISL_2128478, EPI_ISL_2128479, EPI_ISL_2625453, EPI_ISL_2708673, EPI_ISL_2708720, EPI_ISL_2708835			
see above	Virology Department, Sheffield Teaching Hospitals NHS Foundation Trust/Department of Infection, Immunity and Cardiovascular Disease, The Medical School, University of Sheffield	COVID-19 Genomics UK (COG-UK) Consortium	Adri Angyal; Alex Keeley; Benjamin Lindsey; Cariad Evans; Danielle Groves; Dave Partridge; Katie Johnson; Laura Carrilero; Luke Green; Matthew Parker; Matthew Wyles; Mehmet Yavuz; Mohammad Raza; Nikki Smith; Paul Parsons; Rachel Tucker; Rebecca Brown; Thushan de Silva
EPI_ISL_1793786, EPI_ISL_2233377	Virology Laboratory, International Centre for Diarrhoeal Disease Research, Bangladesh (ICDDR,B)	Virology Laboratory, International Centre for Diarrhoeal Disease Research, Bangladesh (ICDDR,B)	Md. Mahfuzur Rahman; Mohammad Enayet Hossain; Mohammed Ziaur Rahman; Mojnu Miah; Mustafizur Rahman; Rashedul Hasan
EPI_ISL_1855026, EPI_ISL_2081229	Virology Laboratory, Scientific Department, Army Medical Center	Virology Laboratory, Scientific Department, Army Medical Center	Anella Monte; Anna Anselmo; Antonella Fortunato; Filippo Molinari; Florigio Lista; Francesco Giordani; Giancarlo Petralito; Giandomenico Cerreto; Giulia Campoli; Lucia Nicosia; Marzia Cavalli; Riccardo De Sanctis; Rossella Brandi; Silvia Fillo; Stella Lia; Vanessa Vera Fain
EPI_ISL_1660232, EPI_ISL_1660255, EPI_ISL_1660262, EPI_ISL_1660278, EPI_ISL_1660288, EPI_ISL_1660289, EPI_ISL_1660290, EPI_ISL_1660291, EPI_ISL_1660330, EPI_ISL_1660331			
see above	Virology Unit, Institut Pasteur de Madagascar	Virology Unit, Institut Pasteur de Madagascar	Angela Brisebarre; Camille Capel; Cara E. Brook; Christian Ranaivoson; Christophe Malabat; Corinne Maufrais; Cristina M. Tato; Emmanuelle Permal; Etienne Simon-Lorière; Frédéric Lemoine; Helisoa Razafimanjato; Jean-Michel Heraud; Joseph L. DeRisi; Louise Lefrançois; Marion Barbet; Maud Vanpeene; Michelle Tan; Méline Bizard; Norosoa Razanajatovo; Philippe Dussart; Soa Fy Andriamandimby; Sylvie Behillil; Sylvie van der Werf; Tsiry Randriambolamanantsoa; Vida Ahyong; Vincent Enouf; Vololoniaina Raharinosy
EPI_ISL_1098607, EPI_ISL_1532802, EPI_ISL_1532815, EPI_ISL_1534529, EPI_ISL_1534537, EPI_ISL_1534542, EPI_ISL_1711981, EPI_ISL_1711982, EPI_ISL_1818959, EPI_ISL_1818960, EPI_ISL_1969671, EPI_ISL_2106243, EPI_ISL_2106247, EPI_ISL_2231585, EPI_ISL_2501043, EPI_ISL_2501046, EPI_ISL_2501061, EPI_ISL_2608433			
see above	Virology Unit, Institut Pasteur du Cambodge	Virology Unit, Institut Pasteur du Cambodge	Cecile Troupin; Chau Darapheak; Chin Savuth; Erik A Karlsson; Jurre Y Siegers; Kraing Sidonn; Leakhena Pum; Ly Sovann; Sokhoun Yann; Teyputita Ou; Veasna Duong; Yi Sengdoeurn
EPI_ISL_2447937	Virology, Universitätsklinikum des Saarlandes	Epigenetics, Saarland University	Jörn Walter; Kathrin Kattler; Nastasja Seiwert; Sascha Tierling; Sigrun Smola; Stefan Lohse; Thorsten Pfuhl
EPI_ISL_2364019, EPI_ISL_2508228	Vita Laboratoriot Oy	Expert Microbiology, National Institute for Health and Welfare	Carita Savolainen-Kopra; Erika Lindh; Haider al-Hello; Jani Halkilahti; Kirsi Liitsola; Niina Ikonen; Olli Vapalahti; Pekka Ellonen; Phuoc Truong; Päivi Laurila; Ravi Kant; Sari Hannula; Soile Blomqvist; Teemu Smura
EPI_ISL_1402358	Vyskov hospital	CMBG FN Brno	Bezdicek M.; Hanslianova M.; Lengerova M.; Svaton J.
EPI_ISL_2365407, EPI_ISL_2365408, EPI_ISL_2365409, EPI_ISL_2365411, EPI_ISL_2365413	WWF Bayanga field laboratory	Robert Koch Institute	F. H. Leendertz; F. S. Niatou-Singa; M. Ulrich; S. Calvignac-Spencer; T. B. Tombolomako; T. Fuh-Neba; U. Vickos
EPI_ISL_2365414, EPI_ISL_2365415, EPI_ISL_2365416, EPI_ISL_2365417	WWF Bayanga field laboratory	WWF Bayanga field laboratory	F. H. Leendertz; F. S. Niatou-Singa; M. Ulrich; S. Calvignac-Spencer; T. B. Tombolomako; T. Fuh-Neba; U. Vickos
EPI_ISL_2422506, EPI_ISL_2422508, EPI_ISL_2422529, EPI_ISL_2422542, EPI_ISL_2422547, EPI_ISL_2422552, EPI_ISL_2422557, EPI_ISL_2422569			
see above	West African Centre for Cell Biology of Infectious Pathogen, University of Ghana, Legon	WACCBIP, University of Ghana, Volta Road, Legon, Accra	Collins M. Morang'a; Dominic S. Y. Amuzu; Dominic S.Y. Amuzu; Edward Danso Fenteng; Emmanuel Kudjo; Evelyn B. Quansah; Evelyn Y Bonney; Frederick Tei-Maya; Ivy A Asante; Joe K. Mutungi; John K. Odoom; Joseph HK Bonney; Joyce M. Ngoi; Lawrence Ofori-Boadu; Lucas N. Amenga-Etego and Gordon A Awandare; Lucas N. Amenga-Etego and Gordon A. Awandare; Mildred Adusei-Poku; Nicaise T. Ndam; Patrick Tetteh Ababio; Peter K Quashie; Peter K. Quashie; Philip M Soglo; Philip M. Soglo; Samirah Saiid; Theophilus Odoom; Vincent Appiah; Violet MCormack; Violette M'cormack; William K. Ampofo; Yaw Bediako
EPI_ISL_1255111, EPI_ISL_1255164, EPI_ISL_1255165, EPI_ISL_1255236	West African Centre for Cell Biology of Infectious Pathogens (WACCBIP), University of Ghana, Accra, Ghana	West African Centre for Cell Biology of Infectious Pathogens (WACCBIP), University of Ghana, Volta Road, Legon-Accra, Ghana	; Abdoulaye B Diallo; Abdul-Karim Abass; Aisha Mohammed; Benjamin Demah Nuertey; Collins M. Morang'a; Dam Kenneth Mibut; Dominic S.Y. Amuzu; Emmanuella Amoako4; Evelyn B. Quansah; Frederick Kumi-Ansah; Frederick Tei-Maya; Gordon A Awandare; Joyce M. Ngoi; Kesego Tapela; Lucas N. Amenga-Etego; Nelson Kibinge; Oliver D Boakye5; Peter K Quashie; Philip M. Soglo; Samirah Saiid; Samuel Kaba Akoriyea; Theophilus Odoom; Vanessa Magnussen; Vincent Appiah; Yaw Bediako
EPI_ISL_2601960, EPI_ISL_2601961, EPI_ISL_2646302, EPI_ISL_2675257, EPI_ISL_2675259, EPI_ISL_2681451, EPI_ISL_2681452, EPI_ISL_2727876, EPI_ISL_2774133, EPI_ISL_2774515			
see above	ZOL	Jessa	Berden et al. on behalf of Jessa_cmdLab; Berden et al. on behalf of the Jessa_cmdLab; Marijke Raymaekers et al. on behalf of the Jessa_cmdLab; Severine Berden et al. on behalf of the Jessa_cmdLab
EPI_ISL_2693479	Zavod za javno zdravstvo Međimurske Županije	Hrvatski zavod za javno zdravstvo	Irena Tabain; Ivana Ferenčak
EPI_ISL_1850498	amedes MVZ Hannover	Robert Koch Institute	
EPI_ISL_2115877	amedes MVZ Zentrum fÃ¼r Pathologie und Zytodiagnostik GmbH (KÃ¶ln)	Robert Koch Institute	
EPI_ISL_2561981, EPI_ISL_2561982, EPI_ISL_2561983, EPI_ISL_2561993, EPI_ISL_2561998, EPI_ISL_2561999, EPI_ISL_2562010, EPI_ISL_2562012			
see above	cerballiance-IDF	Cerba lab	Aude Lessenne; Bénédicte Roquebert; Emmanuel Lecorche; Kader Merah; Laura Verdurme; Patrice Herisson; Sabine Trombert-Paolantoni; Stéphanie Haïm-Boukobza; Thierry Collin
EPI_ISL_826957, EPI_ISL_827346, EPI_ISL_829334	deCODE genetics	deCODE genetics	Agnar Helgason; Alma Moller; Arna B Agustsdottir; Arnaldur Gylfason; Asgeir Sigurdsson; Aslaug Jonasdottir; Berglind Eiriksdottir; Bjarni Thorbjornsson; Brynjar O Jensson; Daniel F Gudbjartsson; Droplaug N Magnusdottir; Elisabet E Gardarsdottir; Emil A Thorarensen; Gardar Sveinbjornsson; Gisli Masson; Gudmundur Georgsson; Gudmundur L Norddahl; Gudrun Sigmundsdottir; Hakon Jonsson; Hannes Eggertsson; Hilma Holm; Ingileif Jonsdottir; Jona Saemundsdottir; Kamilla S Josefsdottir; Kari Stefansson; Karl G Kristinsson; Kjartan R Gudmundsson; Kristin E Sveinsdottir; Louise le Roux; Maney Sveinsdottir; Olafia S Gretarsdottir; Olafur T Magnusson; Pall Melsted; Patrick Sulem; Run Fridriksdottir; Solvi Rognvaldsson; Thora R Gunnarsdottir; Thordur Kristjansson; Thorolfur Gudnason; Unnur Thorsteinsdottir
EPI_ISL_2617442	laboratorio Microbiologia PO Cardarelli	laboratorio Microbiologia PO Cardarelli	Felice V.; Niro G. Scutella' M.
EPI_ISL_434569	unknown	Microbiology, The University of Hong Kong	K.-Y.; K.K.W. and Yuen; To
EPI_ISL_1938308	unknown	PHV-FSS	Son Nguyen

We gratefully acknowledge the following Authors from the Originating laboratories responsible for obtaining the specimens, as well as the Submitting laboratories where the genome data were generated and shared via GISAID, on which this research is based.

All Submitters of data may be contacted directly via www.gisaid.org

Authors are sorted alphabetically.

Accession ID	Originating Laboratory	Submitting Laboratory	Authors
EPI_ISL_2229118	"AR Dept. of Health-PHL, Molecular Diagnostics"	Centers for Disease Control and Prevention Division of Viral Diseases, Pathogen Discovery	Alison Laufer Halpin; Ben L. Rambo-Martin; Clinton R. Paden; Dakota Howard; Darlene Wagner; Dave Wentworth; Dhwani Batra; Jasmine Padilla; Justin Lee; Katie Dillon; Krista Queen; Kristen Knipe; Kristine Lacek; Mark Burroughs; Matthew Schmerer; Mili Sheth; Peter Cook; Sam Shepard; Sarah Nobles; Shoshona Le; Suxiang Tong; Vivien Dugan; Yvette Unoarumhi
EPI_ISL_1711803	"OK Public Health Laboratory, Oklahoma State DOH"	Centers for Disease Control and Prevention Division of Viral Diseases, Pathogen Discovery	Alison Laufer Halpin; Ben L. Rambo-Martin; Clinton R. Paden; Dakota Howard; Darlene Wagner; Dave Wentworth; Dhwani Batra; Jasmine Padilla; Justin Lee; Katie Dillon; Krista Queen; Kristen Knipe; Kristine Lacek; Mark Burroughs; Matthew Schmerer; Mili Sheth; Peter Cook; Sam Shepard; Sarah Nobles; Shoshona Le; Suxiang Tong; Vivien Dugan; Yvette Unoarumhi
EPI_ISL_763065, EPI_ISL_763067	1-Laboratory of Microbiology, National Reference Lab, Charles Nicolle Hospital; 2-University of Tunis ElManar, Faculty of Medicine of Tunis, LR99ES09, Tunis, Tunisia	1-Clinical and Experimental Pharmacology Lab, LR16SP02, National Center of Pharmacovigilance, University of Tunis El Manar, Tunis, Tunisia. 2-Neurodegenerative diseases and psychiatric troubles, LR18SP03, Razi Hospital, University of Tunis El Manar, Tunis, Tunisia. 3- Ministry of Health, National Observatory of New and Emerging Diseases, 1006, Tunis, Tunisia	Alia Ben Kahla; Asma Ferjani; Awatef El MOussi; Awatef El Moussi; Gaies Emna; Habiba Ben Romdhane; Hanen El Jebari; Ilhem Boutiba-Ben Boubaker; Ines Mdini; Jalila Ben Khelil; Maher Kharrat; Mouna Ben Sassi; Mouna Safer; Nissaf Ben Alaya; Riadh Daghfous; Riadh Gouider.; Salma Abid; Sameh Trabelsi; Sana Ferjani; Souissi Amira
EPI_ISL_2532745	1. Glowny Inspektorat Sanitarny. 2. Diagnostyka. Laboratoria Medyczne.	1. ViroGenetics - BSL3 Laboratory of Virology, Małopolska Centre of Biotechnology, Jagiellonian University; 2. genXone SA, Research & Development Laboratory	Aleksandra Gidlewicz; Anna Brylak; Gromowski, T.; Grzegorz Nowicki; Jakub Grabowski; Karol Szeszko; Kowalski, M.; Labaj; Maciej Sykulski; Mazur-Panasiuk, N.; Michał Kaszuba; Natalia Drweska-Matelska; P.P.; Pyrc, K.; Sylwia Januszczak; Szulc, P.; Łukasz Krych
EPI_ISL_2144062	1. Glowny Inspektorat Sanitarny. 2. Diagnostyka. Laboratoria Medyczne.	1. ViroGenetics - BSL3 Laboratory of Virology, Małopolska Centre of Biotechnology, Jagiellonian University; 2. genXone SA, Research & Development Laboratory	Aleksandra Gidlewicz; Anna Brylak; Gromowski, T.; Grzegorz Nowicki; Jakub Grabowski; Karol Szeszko; Kowalski, M.; Labaj; Maciej Sykulski; Mazur-Panasiuk, N.; Michał Kaszuba; Natalia Drweska-Matelska; P.P.; Pyrc, K.; Sylwia Januszczak; Szulc, P.; Łukasz Krych
EPI_ISL_455448	1. ViroGenetics - BSL3 Laboratory of Virology, Małopolska Centre of Biotechnology, Jagiellonian University; 2. II Department of Internal Medicine, Faculty of Medicine, Jagiellonian University Medical College; 3. Narodowy Instytut Zdrowia Publicznego – Państwowy Zakład Higieny (NIZP-PZH)	1. ViroGenetics - BSL3 Laboratory of Virology, Małopolska Centre of Biotechnology, Jagiellonian University; 2. II Department of Internal Medicine, Faculty of Medicine, Jagiellonian University Medical College; 3. Narodowy Instytut Zdrowia Publicznego – Państwowy Zakład Higieny (NIZP-PZH).	Agnieszka Kołakowska-Kulesza; Aleksandra A. Zasada; Aleksandra Milewska; Ewelina Hallman-Szelińska; Katarzyna Owczarek; Katarzyna Pancer; Katarzyna Zacharczuk; Krzysztof Pyrć; Magdalena Rzeczkowska; Marek Sanak; Natalia Wolaniuk; Paweł P Łabaj; Tomasz Wołkowicz; Wojciech Branicki
EPI_ISL_2383982, EPI_ISL_2383984	AL Dept. of Public Health Bureau of Clinical Laboratories	Centers for Disease Control and Prevention Division of Viral Diseases, Pathogen Discovery	Alison Laufer Halpin; Ben L. Rambo-Martin; Clinton R. Paden; Dakota Howard; Darlene Wagner; Dave Wentworth; Dhwani Batra; Jasmine Padilla; Justin Lee; Katie Dillon; Krista Queen; Kristen Knipe; Kristine Lacek; Mark Burroughs; Matthew Schmerer; Mili Sheth; Peter Cook; Sam Shepard; Sarah Nobles; Shoshona Le; Suxiang Tong; Vivien Dugan; Yvette Unoarumhi
EPI_ISL_2097235, EPI_ISL_2098125	ALBANY MEDICAL CENTER	Wadsworth Center, New York State Department of Health	Alexis Russell; Catharine Prussing; Daryl M. Lamson; Erasmus Schneider; Erica Lasek-Nesselquist; John Kelly; Jonathan Plitnick; Kirsten St. George; Matthew Shudt; Melissa A Leisner; Navjot Singh
EPI_ISL_2323453	AR Dept. of Health-PHL, Molecular Diagnostics	Genomics and Discovery, Respiratory Viruses Branch, Division of Viral Diseases, Centers for Disease Control and Prevention	Adam Retchless; Anna Kelleher; Anna Uehara; Brian Lynch; Clinton R. Paden; Dhwani Batra; Haibin Wang; Han Jia Justin Ng; Jasmine Padilla; Jing Zhang; Justin Lee; Krista Queen; Mark Burroughs; Mili Sheth; Morgan Davis; Peter Cook; Rachel Marine; Sarah Nobles; Suxiang Tong; Tara Coalter; Yan Li; Ying Tao
EPI_ISL_517616, EPI_ISL_517634, EPI_ISL_518804	Academic Hospital Paramaribo	Erasmus Medical Center	Bas Oude Munnink; Dion Gajadin; Ed Ijzerman; Emmanuelle Munger; Gary Gummels; Ingrid Krishnadath; Lycke Woittiez; Marion Koopmans; Mireille Van de Veer; Princes Wongsowidjojo; Radjesh Ori; Rohma Banwari; Stephen Vreden
EPI_ISL_1445311, EPI_ISL_1445368, EPI_ISL_1550496, EPI_ISL_1560538, EPI_ISL_1562503, EPI_ISL_1563434, EPI_ISL_1650622, EPI_ISL_1650830, EPI_ISL_1667445, EPI_ISL_1685617, EPI_ISL_1685730, EPI_ISL_1686631, EPI_ISL_1687575, EPI_ISL_1688138, EPI_ISL_1688167, EPI_ISL_1689063, EPI_ISL_1690245, EPI_ISL_1690269, EPI_ISL_1701963, EPI_ISL_1701966, EPI_ISL_1735828, EPI_ISL_1736936, EPI_ISL_1737880, EPI_ISL_1834704, EPI_ISL_1835363, EPI_ISL_1835379, EPI_ISL_1836007, EPI_ISL_1836096, EPI_ISL_1836151, EPI_ISL_1923114, EPI_ISL_1925583, EPI_ISL_1995876, EPI_ISL_1997014, EPI_ISL_1997354, EPI_ISL_1997404, EPI_ISL_1997765, EPI_ISL_1997967, EPI_ISL_1998231, EPI_ISL_1998831, EPI_ISL_1999092, EPI_ISL_1999775, EPI_ISL_2000028, EPI_ISL_2000090, EPI_ISL_2000128, EPI_ISL_2000209, EPI_ISL_2000226, EPI_ISL_2000262, EPI_ISL_2000388, EPI_ISL_2038980, EPI_ISL_2038982, EPI_ISL_2038983, EPI_ISL_2039008, EPI_ISL_2039072, EPI_ISL_2039154, EPI_ISL_2039226, EPI_ISL_2039266, EPI_ISL_2039336, EPI_ISL_2039389, EPI_ISL_2039637, EPI_ISL_2039776, EPI_ISL_2039864, EPI_ISL_2039878, EPI_ISL_2040187, EPI_ISL_2040317, EPI_ISL_2040338, EPI_ISL_2040357, EPI_ISL_2040384, EPI_ISL_2040461, EPI_ISL_2040467, EPI_ISL_2040473, EPI_ISL_2040517, EPI_ISL_2040778, EPI_ISL_2040892, EPI_ISL_2040936, EPI_ISL_2040938, EPI_ISL_2040976, EPI_ISL_2041437, EPI_ISL_2041513, EPI_ISL_2041621, EPI_ISL_2041655, EPI_ISL_2041656, EPI_ISL_2041779, EPI_ISL_2041833, EPI_ISL_2041929, EPI_ISL_2041972, EPI_ISL_2042053, EPI_ISL_2042272, EPI_ISL_2042497, EPI_ISL_2042585, EPI_ISL_2042588, EPI_ISL_2042655, EPI_ISL_2042756, EPI_ISL_2042772, EPI_ISL_2042773, EPI_ISL_2042805, EPI_ISL_2042920, EPI_ISL_2042937, EPI_ISL_2042958, EPI_ISL_2042959, EPI_ISL_2043088, EPI_ISL_2043219, EPI_ISL_2043292, EPI_ISL_2043293, EPI_ISL_2043302, EPI_ISL_2043303, EPI_ISL_2043405, EPI_ISL_2043426, EPI_ISL_2043454, EPI_ISL_2043494, EPI_ISL_2089545, EPI_ISL_2089601, EPI_ISL_2089684, EPI_ISL_2089750, EPI_ISL_2089944, EPI_ISL_2090006, EPI_ISL_2090085, EPI_ISL_2090114, EPI_ISL_2090115, EPI_ISL_2090122, EPI_ISL_2090169, EPI_ISL_2090200, EPI_ISL_2090227, EPI_ISL_2090228, EPI_ISL_2090280, EPI_ISL_2090420, EPI_ISL_2090429, EPI_ISL_2145844, EPI_ISL_2145852, EPI_ISL_2145882, EPI_ISL_2146645, EPI_ISL_2148058, EPI_ISL_2148548, EPI_ISL_2148604, EPI_ISL_2148677, EPI_ISL_2149224, EPI_ISL_2149225, EPI_ISL_2149227, EPI_ISL_2149329, EPI_ISL_2149359, EPI_ISL_2149395, EPI_ISL_2149523, EPI_ISL_2149524, EPI_ISL_2149545, EPI_ISL_2149546, EPI_ISL_2149579, EPI_ISL_2149646, EPI_ISL_2149654, EPI_ISL_2149672, EPI_ISL_2149677, EPI_ISL_2149713, EPI_ISL_2149796, EPI_ISL_2149802, EPI_ISL_2149931, EPI_ISL_2150060, EPI_ISL_2150095, EPI_ISL_2150121, EPI_ISL_2150164, EPI_ISL_2150210, EPI_ISL_2150223, EPI_ISL_2150247, EPI_ISL_2150283, EPI_ISL_2150411, EPI_ISL_2150417, EPI_ISL_2150516, EPI_ISL_2150584, EPI_ISL_2150585, EPI_ISL_2150587, EPI_ISL_2150880, EPI_ISL_2159217, EPI_ISL_2159258, EPI_ISL_2159264, EPI_ISL_2181050, EPI_ISL_2181113, EPI_ISL_2181219, EPI_ISL_2181400, EPI_ISL_2186179, EPI_ISL_2187606, EPI_ISL_2201543, EPI_ISL_2201546, EPI_ISL_2201789, EPI_ISL_2202123, EPI_ISL_2203031, EPI_ISL_2203200, EPI_ISL_2203265, EPI_ISL_2203267, EPI_ISL_2204920, EPI_ISL_2241909, EPI_ISL_2242729, EPI_ISL_2243080, EPI_ISL_2243482, EPI_ISL_2243877, EPI_ISL_2244518, EPI_ISL_2244718, EPI_ISL_2268011, EPI_ISL_2280359, EPI_ISL_2280619, EPI_ISL_2280794, EPI_ISL_2283878, EPI_ISL_2370030, EPI_ISL_2370334, EPI_ISL_2372300, EPI_ISL_2421624, EPI_ISL_2421819, EPI_ISL_2421902, EPI_ISL_2421958, EPI_ISL_2489432, EPI_ISL_2527972, EPI_ISL_2527992, EPI_ISL_2528014, EPI_ISL_2528151, EPI_ISL_2528343, EPI_ISL_2528378, EPI_ISL_2528394, EPI_ISL_2528548, EPI_ISL_2528635, EPI_ISL_2528646, EPI_ISL_2528727, EPI_ISL_2528758, EPI_ISL_2528843, EPI_ISL_2528851, EPI_ISL_2528879, EPI_ISL_2528893, EPI_ISL_2600023, EPI_ISL_2600197, EPI_ISL_2686963, EPI_ISL_2687101, EPI_ISL_2687104, EPI_ISL_2687456, EPI_ISL_2784894, EPI_ISL_2784987, EPI_ISL_2785171			
see above	Aegis Sciences Corporation	Centers for Disease Control and Prevention Division of Viral Diseases, Pathogen Discovery	Adrian Paskey; Alec Vest; Benjamin Rambo-Martin; Christopher Gulvick; Clinton R. Paden; Cyndi Clark; Dakota Howard; Darlene Wagner; Dhwani Batra; Dillon Nall; Duncan MacCannell; Ethan Sanders; Holly Houdeshell; Jason Caravas; Kara Moser; Matthew Hardison; Matthew Schmerer; Ola Kvalvaag; Patrick Campbell; Peter W. Cook; Rob Case; Scott Sammons; Shatavia Morrison; Shaun Westlund; Vikramsinha Ghorpade; Yvette Unoarumhi
EPI_ISL_2617036, EPI_ISL_2617050, EPI_ISL_2617084, EPI_ISL_2617085	Africa_CDC - Malawi P4 S2	CERI, Centre for Epidemic Response and Innvoation, Stellenbosch University and KRISP, KZN Research Innovation and Sequencing Platform, UKZN.	Auld A; Chilima B; Chiwaula M; Emmanuel SJ; Giandhari J; Kaba M; Kampira E; Kasambara W; Kim L; Lessells R; Maida A; Mvula B; Mwangomba W; Naidoo Y; Panja L; Pillay S; Tegally H; Wadonda N; Wilkinson E; de Oliveira T
EPI_ISL_2617052, EPI_ISL_2617053, EPI_ISL_2617089, EPI_ISL_2617090	Africa_CDC - Malawi P6 S2	CERI, Centre for Epidemic Response and Innvoation, Stellenbosch University and KRISP, KZN Research Innovation and Sequencing Platform, UKZN.	Auld A; Chilima B; Chiwaula M; Emmanuel SJ; Giandhari J; Kaba M; Kampira E; Kasambara W; Kim L; Lessells R; Maida A; Mvula B; Mwangomba W; Naidoo Y; Panja L; Pillay S; Tegally H; Wadonda N; Wilkinson E; de Oliveira T
EPI_ISL_1317489, EPI_ISL_1819941, EPI_ISL_2225843, EPI_ISL_2333510, EPI_ISL_2566163	Akershus University Hospital, Department for Microbiology and Infectious Disease Control	Norwegian Institute of Public Health, Department of Virology	'Kathrine Stene-Johansen; Atiya R Ali; Debech Nadia; Engebretsen Serina Beate; Garcia Llorente Ignacio; Hilde Elshaug; Hilde Vollan; Jon Bråte; Kamilla Heddeland Instefjord; Karoline Bragstad; Kathrine Stene-Johansen; Line Victoria Moen; Marie Paulsen Madsen; Olav Hungnes; Pedersen Benedikte Nevjen; Rasmus Riis Kopperud
EPI_ISL_1575111, EPI_ISL_1653901, EPI_ISL_1701714, EPI_ISL_2008985	Alaska State Virology Laboratory	Alaska State Virology Laboratory	Elva House; Jack Chen; Jacob Zidek; Lisa Smith; Ph.D.; Stephanie DeRonde
EPI_ISL_1385807, EPI_ISL_1385811	Alfa Diagnostica LLC	ONCOGENE LLC	ONCOGENE LLC
EPI_ISL_1385804	Alfa Diagnostica, Republic of Moldova	ONCOGENE LLC	ONCOGENE LLC
EPI_ISL_1468729	Altius Institute	Seattle Flu Study	Alex Nguyen; Amanda Adler; Andrew Meuser; Barry R. Lutz; Benjamin Pelle; Caitlin R. Wolf; Chris D. Frazar; Clem Green; Daniel Bates; Deborah A. Nickerson; Elisabeth Brandstetter; Erica Ryke; Hannah Petersen; Helen Y. Chu; Jacob Rodriguez; Janet A. Englund; Jay Shendure; Jessica Halow; John Stamatoyannopoulos; Joshua Richards; Jover Lee; Julia Wald; Kairsten Fay; Kirsten Lacombe; Kneshay Harper; Lea M. Starita; Mark J. Rieder; Matt Hartman; Matthew Richardson; Matthew Thompson; Melissa Truong; Michael Boeckh; Michael Famulare; Misja Ilcisin; Muhammad Halimun; Olivia Waltner; Peter D. Han; Rebecca Bruders; Ryan Alexander; Sadie Patraw; Sofia Olsson; Stephanie DeBaun; Thomas R. Sibley; Tobias Ragoczy; Trevor Bedford; Truong Nguyen
EPI_ISL_2484895, EPI_ISL_2484897, EPI_ISL_2484898, EPI_ISL_2484899	Amdavad Municipal Corporation, Ahmedabad	Gujarat Biotechnology Research Centre	Chaitanya Joshi; Dalip Singh Rathore; Dinesh Kumar; Hiren Mandalia; Madhvi Joshi; Manish Kumar; Nitin Savaliya; Ramesh Pandit; Sonal Sharma; Twinkle Soni
EPI_ISL_1994608	Ampath - Kingsway	KRISP, KZN Research Innovation and Sequencing Platform	Emmanuel SJ; Giandhari J; Khan S; Lessells R; Mdlalose K; Naidoo Y; Pillay S; Ramphal U; Tegally H; Wilkinson E; York D; de Oliveira T
EPI_ISL_2178786	Ampath Laboratories	National Institute for Communicable Diseases of the National Health Laboratory Service	Amoako DG; Bhiman JN; Glass A; Gottberg A; Mahlangu B; Marshall T; Mohale T; Ntuli N; Scheepers C; Viana R
EPI_ISL_1910791	Arizona State University	Arizona State University	Efrem S. Lim; Joshua LaBaer; Joy M. Blain; LaRinda A. Holland; Matthew F. Smith; Nicholas J. Mellor; Peter T. Skidmore; Rabia Maqsood; Valerie Harris; Vel Murugan
EPI_ISL_2156780, EPI_ISL_2157307	Arkansas Children's Hospital	Center for Global Health, University of New Mexico Health Sciences Center	Bobby Boyanton; Catherine Kirkpatrick; Darrell Dinwiddie; Daryl Domman; Joshua L. Kennedy; Kurt Schwalm; Valerie Morley
EPI_ISL_2484683, EPI_ISL_2484748, EPI_ISL_2484751	Arkansas Department of Health	Center for Global Health, University of New Mexico Health Sciences Center	Atul Kothari; Darrell Dinwiddie; Daryl Domman; Joshua L. Kennedy; Kurt Schwalm; Valerie Morley
EPI_ISL_413490	Auckland Hospital	Institute of Environmental Science and Research (ESR)	Erasmus Smit; Gary McAuliffe; Joep de Ligt; Lauren Jelly; Matt Storey; Matthew Blakiston; Sally Roberts; Xiaoyun Ren
EPI_ISL_2001099	BIOSCIENCE, King Faisal Hospital Research Center	BIOSCIENCE, King Faisal Hospital Research Center	Al-Qahtani A, A.; Alahideb B, M.; Alhamlan F, S.; Almaghrabi R, S.; Alsanea M, S.; Althawadi S, I.; K. and Balavenkatesh Mani, M.; Mutabagani M, S.; UdayaRaja G
EPI_ISL_859570, EPI_ISL_859604, EPI_ISL_859621, EPI_ISL_859637, EPI_ISL_859724, EPI_ISL_859774, EPI_ISL_860070			
see above	BTC, Khalifa University	BTC, Khalifa University	Al Safar et al
EPI_ISL_833333	Batangas City Health Office	Research Institute for Tropical Medicine	Catalino Demetria; Daria Manalo; Edelwisa Mercado; Francisco Gerardo Polotan; Hannah Leah Morito; Inez Andrea Medado; John Leonard Chan; Kirstyn Brunker; Ma Angelica Tujan; Othoniel Jan Onza

EPI_ISL_2529850, EPI_ISL_2529852, EPI_ISL_2529854, EPI_ISL_2529856, EPI_ISL_2529858, EPI_ISL_2529861	Baylor College of Medicine/ GCID	Baylor College of Medicine/ GCID	Charu Agrawal; Donna Muzny; Harsha Doddapaneni; Joseph Pertosino; Paige Farinholt; Pedro A. Piedra; Qinchang Meng; Qing Meng; Richard A. Gibbs; Timothy Farinholt; Vipin Menon; Xiang Qin
EPI_ISL_2083033	Baylor Scott & White-Grapevine	Baylor Scott & White-Temple	Ari Rao; Kimberly Walker; Linden Morales; Marcus Volz; Shelby Hendrickson
EPI_ISL_529216	Beijing Institute of Microbiology and Epidemiology	Beijing Institute of Microbiology and Epidemiology	Cui, Y.; Fan; Guo, Y.; Hang; Hou, J.; Li, B.; Mi, Z.; Mu, J.; Qin, E.; Song; Teng; Wu, Y.; Xu, Z.; Yajun.; Yang, R.; Yong, Y.; Yue; Zhang, X.
EPI_ISL_1423207, EPI_ISL_2615868, EPI_ISL_2758377	Berkeley Medical Center	WVU and Marshall University Combined Genomics Core Facilities	"James Denvir; James Denvir; Peter Perrotta; Peter Stoilov; Ryan Percifield; Ryan Percifield"; Wesley Kimble
EPI_ISL_2107513	Biochemistry and Molecular Biology Department-Faculty of Medicine, Al-Quds University	Biochemistry and Molecular Biology Department-Faculty of Medicine, Al-Quds University	Al-Jawabreh, A.; Ereqat, S.; Naserddin, A.
EPI_ISL_2608391, EPI_ISL_2608395	Borneo Medical Centre	Institute of Health and Community Medicine	Chan Chia Jui; Chua Hock Hin; David Perera; Ooi Mong How; Tonnii Sia Loong Loong; Wong Jyn Shan; Wong Kiing Aik
EPI_ISL_900708	Bozeman Health Deaconess Hospital	Wiedenheft lab, Montana State University	Anna Nemudraia; Artem Nemudryi; Calvin Cicha; Deann T. Snyder; Diane Bimczok; Helen Lee; Jodi F. Hedges; Joseph Nichols; Karl K. Vanderwood; Mark A. Jutila and Blake Wiedenheft; Tanner Wiegand
EPI_ISL_1253816, EPI_ISL_1743907, EPI_ISL_1744188, EPI_ISL_1744257, EPI_ISL_1786987, EPI_ISL_1825316, EPI_ISL_1825321, EPI_ISL_1825322, EPI_ISL_1825553, EPI_ISL_1825586, EPI_ISL_1918919, EPI_ISL_1968000, EPI_ISL_1968007, EPI_ISL_1968369, EPI_ISL_1968371, EPI_ISL_1968372, EPI_ISL_1968435, EPI_ISL_1971653, EPI_ISL_1971724, EPI_ISL_1971811, EPI_ISL_1971855, EPI_ISL_1971900, EPI_ISL_1971917, EPI_ISL_2016398, EPI_ISL_2016438, EPI_ISL_2096743, EPI_ISL_2104340			
see above	Broad Institute Clinical Research Sequencing Platform	Infectious Disease Program, Broad Institute of Harvard and MIT	Adams, G.; B.L.; B.W.; Bauer, M.; Birren; Blumenstiel, B.; Brown, C.; Carter, A.; Chaluvadi, S.; D.J.; DeFelice, M.; DeRuff, K.; Dodge, S.; Gabriel, S.; Gallagher, G.; Gladden-Young, A.; Granger, B.; J.E.; K.J.; Lagerborg, K.; Larkin, K.; Lee, M.; Lemieux; Lennon, N.; Loreth, C.; MacInnis; Madoff, L.; McGovern, S.; Meldrim, J.; Normandin, E.; P.C.; Park; Pearlman, L.; Reilly, S.; Rudy, M.; Sabeti; Siddle; Smole, S.; Tomkins-Tinch, C.; Vicente, G.; and MacInnis; and Sabeti
EPI_ISL_2558058, EPI_ISL_2558061	Bureau Of Quarantine (BOQ)	Philippine Genome Center	Alethea R. de Guzman; Anna Ong-Lim; Arianne A. Zamora; Asia Louisa U. Chong; Benedict A. Maralit; Candice Francheska B. Tambaoan; Carlo M. Lapid; Celia Carlos; Devon Ray Pacial; Edsel Maurice Salvaña; El King D. Morado; Elcid Aaron R. Pangilinan; Eva Maria Cutiongco-de la Paz; Francis A. Tablizo; Irish Coleen A. Asin; Jaime C. Montoya; Jan Michael C. Yap; Jo-Hannah S. Llames; John Q. Wong; Joshua Gregor A. Dizon; Juan Antonio R. Magalang; Karol Sophia Agape R. Padilla; Kenneth M. Kim; Kris P. Punayan; Marc Edsel C. Ayes; Maria Rosario Singh-Vergeire and Cynthia P. Saloma; Maria Sofia L. Yangzon; Marissa Alejandria; Razel Nikka M. Hao; Renato Jacinto Q. Mantaring; Rianna Patricia S. Cruz; Sheila Mae M. Araiza
EPI_ISL_1298474	Burshtyn CCH	The Institute of Molecular Biology and Genetics of NASU	M.Tukalo et al.
EPI_ISL_2188097	Butuan Medical Center	Philippine Genome Center	Alethea R. de Guzman; Anna Ong-Lim; Arianne A. Zamora; Asia Louisa U. Chong; Benedict A. Maralit; Candice Francheska B. Tambaoan; Carlo M. Lapid; Celia Carlos; Devon Ray Pacial; Edsel Maurice Salvaña; El King D. Morado; Elcid Aaron R. Pangilinan; Eva Maria Cutiongco-de la Paz; Francis A. Tablizo; Irish Coleen A. Asin; Jaime C. Montoya; Jan Michael C. Yap; Jo-Hannah S. Llames; John Q. Wong; Joshua Gregor A. Dizon; Juan Antonio R. Magalang; Karol Sophia Agape R. Padilla; Kenneth M. Kim; Kris P. Punayan; Marc Edsel C. Ayes; Marc Jerrone R. Castro; Maria Rosario Singh-Vergeire and Cynthia P. Saloma; Maria Sofia L. Yangzon; Marissa Alejandria; Razel Nikka M. Hao; Rianna Patricia S. Cruz; Sheila Mae M. Araiza
EPI_ISL_1371898, EPI_ISL_1371900, EPI_ISL_1371905	C H DE LA POLYNESIE FRANCAISE	CNR Virus des Infections Respiratoires - France SUD	Antonin Bal; Bruno Lina; Gregory Destras; Gwendolyne Burfin; Hadrien Regue; Laurence Josset; Martine Valette; Quentin Semanas
EPI_ISL_1135056	C.H. PRINCESSE GRACE	CNR Virus des Infections Respiratoires - France SUD	Antonin Bal; Bruno Lina; Gregory Destras; Gwendolyne Burfin; Hadrien Regue; Laurence Josset; Martine Valette; Quentin Semanas
EPI_ISL_2341631, EPI_ISL_2500090, EPI_ISL_2500168, EPI_ISL_2500194	CA DPH Viral and Rickettsial Disease Laboratory	Chan-Zuckerberg Biohub	CZB Cliahub Consortium
EPI_ISL_1629150	CERBALLIANCE	UMR PIMIT	Dr Camille Lebarbenchon; Dr David A Wilkinson; Dr Patrick Mavingui; Magali Turpin
EPI_ISL_2562031, EPI_ISL_2562045	CH Princesse Grace	Cerba lab	Aude Lessenne; Bénédicte Roquebert; Emmanuel Lecorche; Kader Merah; Laura Verdurme; Patrice Herisson; Sabine Trombert-Paolantoni; Stéphanie Haïm-Boukobza; Thierry Collin
EPI_ISL_2694671	CHRIS HANI BARAGWANATH	National Institute for Communicable Diseases of the National Health Laboratory Service	Amoako DG; Bhiman JN; Everatt J; Ismail A; Mahlangu B; Mnguni A; Mohale T; Ntuli N; Scheepers C
EPI_ISL_2454656, EPI_ISL_2454659	CHRIS HANI BARAGWANATH LABORATORY	National Institute for Communicable Diseases of the National Health Laboratory Service	Amoako DG; Bhiman JN; Everatt J; Ismail A; Mahlangu B; Mohale T; Ntuli N; Scheepers C
EPI_ISL_1369440	CHUV	Laboratory of genomics and metagenomics, Institute of Microbiology, University Hospital Centre and University of Lausanne, Switzerland	Claire Bertelli; Damien Jacot; Gilbert Greub; Sébastien Aeby; Trestan Pillonel
EPI_ISL_2533930, EPI_ISL_2533931, EPI_ISL_2533932	CIAD-Hermosillo	Immunology Laboratory	; Alejandra García-Gasca; Alejandra Hernández-Terán; Alejandro Sánchez-Flores; Alfredo Herrera-Estrella; Alicia Ocaña-Mondragón; Andreu Comas-García; Angel Gustavo Salas-Lais; Antonio Loza Román; Bernardo Martínez-Miguel; Blanca Taboada; Brenda Irasema Maldonado-Meza; Bruno Gómez-Gil; Carla Ivón Herrera-Najera; Carlos F. Arias; Celia Boukadida; Clara Esperanza Santacruz-Tinoco; Concepción Grajales-Muñiz; Consorcio Mexicano de Vigilancia Genómica (CoViGen-Mex). Authors (in alphabetical order): Julio Elias Alvarado-Yaah; Cristóbal Cháidez-Quiróz; Célida Duque Molina; Célida Martínez- Rodríguez; Daniel Fregoso-Rueda; Daniel Lira Morales; Eduardo Becerril-Vargas; Fernando Fontove-Herrera; Fidencio Mejía-Nepomuceno; Francisco Pulido; Gloria Elena Espinosa-Ayala; Gloria María Molina-Salinas; Gloria Vazquez; Hector Esteban Paz-Juárez; Hector Montoya-Fuentes; Helen Haydee Fernanda Ramírez-Plascencia; Irvin González-López; Jean Pierre González; Jesús Hernández; Joel Armando Vázquez-Pérez.; Jorge Salas-Hernández; José Antonio Enciso-Moreno; José Arturo Martínez-Orozco; José Esteban Muñoz-Medina; José de Jesús Nuñez-Contreras; Juan Bautista Chale-Dzul; Julissa Enciso-Ibarra; Luis Alberto Ochoa-Carrera; Margarita Matías-Florentino; Mario Mújica-Sánchez; Marissa Perez-Garcia; María Guadalupe Santiago-Mauricio; María Guadalupe de Jesús Mireles-Rivera; Nelly Sélem-Mojica; Pavel Isa; Ricardo Ciria Merce; Ricardo Grande; Rosa María Gutiérrez Rios; Santiago Ávila-Ríos; Selene Zárate; Susana Lopez; Verónica Mata-Haro; Victor Eduardo García-Arias; Víctor Hugo Borja-Aburto
EPI_ISL_487465	CICM-Mali	Bundeswehr Institut of Microbiology	Antwerpen; Bestehorn-Willmann; Dürr; Heitzer; Kouriba; Maiga; Quedraogo; Rehn; Sangaré; Sogodogo; Traoré; Walter; Wölfel; Zimmermann
EPI_ISL_2017662, EPI_ISL_2017663	CLILAB	Microbiology Department	Aida Gonzalez-Diaz; Carmen Ardanuy; Jordi Camara; Jordi Niubó; Laura Calatayud; M Angeles Domínguez; Miguel Fernandez-Huerta; Sara Marti
EPI_ISL_2105884	CSL, Maryland Department of Health	CSL, Maryland Department of Health	Keller, E.; Loomis, J.; Low, K.
EPI_ISL_1710532	CURATIVE	New Mexico Department of Health Scientific Laboratory	Anastacia Griego-Fisher; D'eldra Malone; Ellie Johnson; Jennifer Benoit; Linda Salazar; Ratheesh Rajan
EPI_ISL_2234907	CYRLAB	IICS-UNA	Adriana Valenzuela; Alejandra Rojas; Chyntia Diaz; Eva Nara; Fatima Cardozo; Florencia del Puerto; Joel Ortiz; Jonas Fernandez; Laura Franco; Laura Mendoza; Leticia Rojas; Magaly Martinez; Maria Eugenia Galeano.
EPI_ISL_2105276, EPI_ISL_2155703	Cagayan Valley Medical Center Molecular Laboratory	Philippine Genome Center	Alethea R. de Guzman; Anna Ong-Lim; Arianne A. Zamora; Asia Louisa U. Chong; Benedict A. Maralit; Candice Francheska B. Tambaoan; Carlo M. Lapid; Celia Carlos; Devon Ray Pacial; Edsel Maurice Salvaña; El King D. Morado; Eva Maria Cutiongco-de la Paz; Francis A. Tablizo; Irish Coleen A. Asin; Jaime C. Montoya; Jan Michael C. Yap; Jo-Hannah S. Llames; John Q. Wong; Joshua Gregor A. Dizon; Juan Antonio R. Magalang; Karol Sophia Agape R. Padilla; Kenneth M. Kim; Kris P. Punayan; Marc Edsel C. Ayes; Marc Jerrone R. Castro; Maria Rosario Singh-Vergeire and Cynthia P. Saloma; Maria Sofia L. Yangzon; Marissa Alejandria; Razel Nikka M. Hao; Rianna Patricia S. Cruz; Sheila Mae M. Araiza
EPI_ISL_2537412, EPI_ISL_2537461	California Department of Public Health	California Department of Public Health	CDPH IDLB COVIDNet et al
EPI_ISL_2778814, EPI_ISL_2778817, EPI_ISL_2778831, EPI_ISL_2778833, EPI_ISL_2778873, EPI_ISL_2778886, EPI_ISL_2778903, EPI_ISL_2778937, EPI_ISL_2778944, EPI_ISL_2778951			
see above	California Department of Public Health Valencia Branch Laboratory (CDPH VBL)	California Department of Public Health	CDPH-COVIDNet; UCLA Technology Center for Genomics & Bioinformatics
EPI_ISL_2774008	Cantacuzino National Military-Medical Institute, Viral Respiratory Infections Laboratory	Cantacuzino Institute Virology	Luiza Ustea; Mihaela Lazar; Mihaela Oprea; Nicoleta Paraschiv; Sorin Dinu
EPI_ISL_579066, EPI_ISL_579071, EPI_ISL_579072, EPI_ISL_579480, EPI_ISL_579496, EPI_ISL_622780, EPI_ISL_622795, EPI_ISL_622799, EPI_ISL_622800, EPI_ISL_622808, EPI_ISL_622824, EPI_ISL_622827, EPI_ISL_755638, EPI_ISL_843195, EPI_ISL_843198, EPI_ISL_1082255, EPI_ISL_1904855, EPI_ISL_1967890, EPI_ISL_2406488			
see above	Canterbury Health Laboratories	Institute of Environmental Science and Research (ESR)	Anja Werno; Antje van der Linden; Arlo Upton; Chris Mansell; David Hammer; Dragana Drinkovic; Erasmus Smit; Gary McAuliffe; Hana Sofia Andersson; Hermes Perez; James Ussher; Jill Sherwood; Jing Wang; Joep de Ligt; Josh Freeman; Julia Howard; Juliet Elvy; Lauren Jelly; Mary DeAlmeida; Matt Blakiston; Matt Storey; Matthew Rogers; Max Bloomfield; Michael Addidle; Michelle Balm; Muhammad Faisal; Nikki Freed; Olin Silander; Olivia Stroeven; Paula scholes; Rachel Boyle; Sally Roberts; SallyAnn Harbison; Sarah Jefferies; Sharmini Muttaiyah; Susan Lin; Susan Morpeth; Susan Taylor; Timothy Blackmore; Vani Sathyendran; Veronica Playle; Virginia Hope; Xiaoyun Ren
EPI_ISL_977539	Caribbean Public Health Agency	Carrington Lab, Department of PreClinical Sciences	Adesh Ramsubhag; Arianne Brown-Jordan; Avery Hinds; Chinna Chinnadurai; Christine V. F. Carrington; Christopher Oura; Dr. Sharon Belmar-George; Gabriel Escobar; Jaya Jayaraman; Jerome Foster; Karla Georges; Marsha Ivey; Naresh Nandram; Nikita S. D. Sahadeo; Nuno Faria; Oliver Pybus; Rahul Naidu; Rajini Haraksingh; Risha Singh; Sarah Hill; Stanley Giddings; SueMin Nathaniel; Vernie Ramkissoon
EPI_ISL_872196, EPI_ISL_1588899, EPI_ISL_1589003, EPI_ISL_1589115, EPI_ISL_1591250, EPI_ISL_1591265, EPI_ISL_1591351			
see above	Caribbean Public Health Agency	Carrington Lab, Department of PreClinical Sciences, Faculty of Medical Sciences, The University of the West Indies	Adesh Ramsubhag; Arianne Brown-Jordan; Avery Hinds; Chinna Chinnadurai; Christine V. F. Carrington; Christopher Oura; Gabriel Escobar; Jaya Jayaraman; Jerome Foster; Karla Georges; Marsha Ivey; Naresh Nandram; Nikita S. D. Sahadeo; Nuno Faria; Oliver Pybus; Rahul Naidu; Rajini Haraksingh; Risha Singh; Roshan Parasram; Sarah Hill; Stanley Giddings; SueMin Nathaniel; Vernie Ramkissoon
EPI_ISL_526741	Center for Laboratory Control of Infectious Diseases, Korea Centers for Diseases Control and Prevention	Center for Laboratory Control of Infectious Diseases, Korea Centers for Diseases Control and Prevention	Ae Kyung Park; Eunkyung Shin; Heui Man Kim; Jeong-Min Kim; Jin Sun No; Junyoung Kim; Myung Guk Han; Yoon-Seok Chung
EPI_ISL_1704136, EPI_ISL_2484844	Center for Laboratory Medicine	Center for Laboratory Medicine	Yannick Gerth
EPI_ISL_2385379	Center of Hygiene and Epidemiology in Belgorod Region	WHO National Influenza Centre Russian Federation	Alexey Masharsky; Andrey Komissarov; Artem Fadeev; Daria Danilenko; Dmitry Lioznov; Elena Nabieva; Georgii Bazykin; Kirill Varchenko; Ksenia Safina; Kseniya Komissarova; Maria Baturova; Maria Pisareva; Maria Timofeeva; Mikhail Bakaev; Nikita Yolshin; Oula Masour; Tamila Musaeva; Veronika Eder
EPI_ISL_1760221	Centers for Disease Control and Prevention, Dengue Branch	Centers for Disease Control and Prevention, Dengue Branch	Betzabel Flores; Gabriela Paz-Bailey; Gilberto A. Santiago; Glenda Gonzalez; Jorge L. Munoz-Jordan; Keyla Charriez
EPI_ISL_2455327, EPI_ISL_2455330	Centers for Disease Control, R.O.C. (Taiwan)	Centers for Disease Control, R.O.C. (Taiwan)	Ji-Rong Yang; Jung-Jung Mu; Ming-Tsan-Liu; Yu-Chi-Lin
EPI_ISL_815390	Centogene	Centogene	Krishna Kumar Kandaswamy; Peter Bauer; Vivi Hue-Trang Lieu
EPI_ISL_2285196	Central Health Laboratory	National Institute for Communicable Diseases of the National Health Laboratory Service	Allam M; Amoako DG; Baboo SB; Bhiman JN; Ismail A; Mahlangu B; Manraj SS; Mohale T; Ntuli N; Ramuth M; Sonoo J
EPI_ISL_2502389, EPI_ISL_2502391, EPI_ISL_2502393, EPI_ISL_2502398, EPI_ISL_2502414, EPI_ISL_2502415, EPI_ISL_2502419, EPI_ISL_2502430, EPI_ISL_2502437, EPI_ISL_2502453, EPI_ISL_2502464, EPI_ISL_2502467, EPI_ISL_2502468, EPI_ISL_2502473, EPI_ISL_2502487, EPI_ISL_2502489, EPI_ISL_2502491, EPI_ISL_2502495, EPI_ISL_2502496, EPI_ISL_2502497, EPI_ISL_2502514, EPI_ISL_2502521, EPI_ISL_2502549, EPI_ISL_2502551, EPI_ISL_2502555, EPI_ISL_2502560, EPI_ISL_2502561, EPI_ISL_2502563			

see above	Central Laboratory, Bureau of Public Health (BOG) and Academic Hospital Paramaribo	Erasmus Medical Center	Bas B Oude Munnink; Cherise Beek; Consuella Partowidjojo; Dion Gajadin; Ed PF IJzerman; Emmanuelle Munger; Gary Gummels; Ingrid SK Krishnadath; Lycke Woittiez; Marion PG Koopmans; Mireille Van de Veer; Phyllis Pinas; Princes Wongsowidjojo; Radjesh Ori; Ranisha Doerbalie; Rohma Banwari; Soeradj Harkisoen; Stephen Vreden; Tilotmadebie Ramlal; Verne Nanhoe
EPI_ISL_693477, EPI_ISL_693478, EPI_ISL_693479, EPI_ISL_693480, EPI_ISL_693481, EPI_ISL_693482, EPI_ISL_693483, EPI_ISL_1307644, EPI_ISL_1307676, EPI_ISL_1307678, EPI_ISL_1322321, EPI_ISL_1322330			
see above	Central Public Health Laboratory	National Public Health Laboratory, National Centre for Infectious Diseases	Esorom Daoni; Lin Cui; Raymond Tzer Pin Lin; Sophie Octavia; Theresa Palou; Tze Minn Mak; Zhenyang Zhou
EPI_ISL_1827727	Centralny Szpital Kliniczny MSWiA w Warszawie	1. Virogenetics Laboratory of Virology, Małopolska Centre of Biotechnology, Jagiellonian University. 2. Intercollegiate Faculty of Biotechnology University of Gdansk and Medical University of Gdansk	Adam Sybilski; Anna Meler; Krystyna Bienkowska-Szewczyk; Krzysztof Pyrc; Lukasz Rabalski; Maciej Kosinski; Michał Hampel; Natalia Mazur-Panasiuk; Sławomir Butkiewicz
EPI_ISL_525476	Centre for Dengue Research	Centre for Dengue Research	Chandima Jeewandara; Deshni Jayathilaka; Dinuka Ariyaratne; Dinuka Guruge; Diyanath Ranasinghe; Gathsaurie Neelika Malavige; Laksiri Gomes; Ruwan Wijayamuni
EPI_ISL_525474	Centre for Dengue Research	Centre for Dengue Research, USJ, SL	Chandima Jeewandara; Deshni Jayathilaka; Dinuka Ariyaratne; Dinuka Guruge; Diyanath Ranasinghe; Gathsaurie Neelika Malavige; Laksiri Gomes; Ruwan Wijayamuni
EPI_ISL_1233058, EPI_ISL_1233066, EPI_ISL_1233070, EPI_ISL_1233081, EPI_ISL_1233104, EPI_ISL_1233110, EPI_ISL_1233129, EPI_ISL_1239365, EPI_ISL_1533838, EPI_ISL_1533849, EPI_ISL_1970360, EPI_ISL_1970377, EPI_ISL_1970379, EPI_ISL_1970380, EPI_ISL_1970396, EPI_ISL_1970418, EPI_ISL_2481409, EPI_ISL_2481411, EPI_ISL_2481428, EPI_ISL_2481438, EPI_ISL_2600339, EPI_ISL_2600342, EPI_ISL_2600343, EPI_ISL_2600345			
see above	Centre for Dengue Research and AICBU, Department of Immunology and Molecular Medicine	Centre for Dengue Research and AICBU, Department of Immunology and Molecular Medicine	Chandima Jeewandara; Deshan Madhusanka; Deshni Jayathilaka; Dinuka Ariyaratne; Diyanath Ranasinghe; Gathsaurie Neelika Malavige; Laksiri Gomes; Tibutius Thanesh Pramanayagam
EPI_ISL_2399460	Centre for Human Virology and Genomics, Microbiology Department, Nigerian Institute of Medical Research	Central Research Laboratory, Nigerian Institute of Medical Research	Ayorinde Babatunde James; Azuka Patrick Okwuraiwe; Babatunde Lawal Salako; Bamidele Iwalokun; Chika Kingsley Onwuamah; Grace Oni; Joseph Ojonugwa Shaibu; Josiah Ayoola Isong; Joy Ayoola; Muinah Adenike Fowora; Ngozi Mirabel Otuonye; Nyam Itse Yusuf; Olufemi Samuel Amoo; Phasha-Muchemenye Mmatshepho; Rahaman A. Ahmed; Rosemary Ajuma Audu; Sharon Abimbola; Sola Ajibaye; Uyi Emokpae; Yusuf Jimoh
EPI_ISL_413550	Centre for Human and Zoonotic Virology (CHAZVY), College of Medicine University of Lagos/Lagos University Teaching Hospital (LUTH), part of the Laboratory Network of the Nigeria Centre for Disease Control (NCDC)	African Centre of Excellence for Genomics of Infectious Diseases (ACEGID), Redeemer's University, Ede, Osun State, Nigeria	Ajogbasile F.V.; Folarin O.A.; Ihekweazu C. Happi C.T.; Kayode A.; Oguzie J.; Oluniyi P.E.
EPI_ISL_2231325	Centro de Diagnostico COVID-19 UABC Tijuana	Andersen lab at Scripps Research	German Ibarra; Jonathan Vincent Baena; Jorge Luis Jimenez Niebla; Manuel Sanchez Alavez; Oscar Efren Zazueta Fierro; SEARCH Alliance San Diego with Idanya Rubi Serafin Higuera
EPI_ISL_2031711, EPI_ISL_2031718, EPI_ISL_2031746, EPI_ISL_2031748	Centro de Innovación en Vigilancia Epidemiológica (CiVE), Institut Pasteur Montevideo, Uruguay	Centro de Innovación en Vigilancia Epidemiológica (CiVE), Institut Pasteur Montevideo, Uruguay	Alicia Costábile; Alvaro Fajardo; Andrés Lizosaín; Belén González; Bernardina Rivera; Cecilia Alonso; Cecilia Salazar; Gonzalo Moratorio; Gregorio Iraola; Henry Albornoz; Ignacio Ferrés; Inés Bellini; Juan Zanetti; Julio Medina; Lucia Bilbao; Luciana Griffero; Lucía Spangenberg; Ma Noel Bentancor; Ma Pía Techera; Mailen Arleo; Martina Alonso; María José Benítez; Matías Maidana; Mauricio Méndez; Melissa Duquía; Mercedes Paz; Natalia Rego; Natalia Reyes; Odhille Chappos; Paula Perbolianachis; Pilar Moreno; Rodney Colina; Rodrigo Arce; Tamara Fernández; Tania Possi
EPI_ISL_1662123, EPI_ISL_1662125, EPI_ISL_1662127	Centro de Investigacion Biomedica de Occidente (CIBO)	Unidad de Genomica Avanzada	Alejandro Sanchez-Flores; Alfredo Herrera-Estrella; Alicia Ocana-Mondragon; Angel Gustavo Salas-Lais; Bernardo Martinez-Miguel; Blanca Taboada; Brenda Irasema Maldonado-Meza; Carla Ivon Herrera-Najera; Carlos F. Arias; Celia Boukadida; Celida Duque Molina; Clara Esperanza Santacruz-Tinoco; Concepcion Grajales-Muniz; Consorcio Mexicano de Vigilancia Genomica (CoViGen-Mex). Authors (in alphabetical order): Julio Elias Alvarado-Yaah; Fernando Fontove-Herrera; Francisco Pulido; Gloria Elena Espinosa-Ayala; Gloria Maria Molina-Salinas; Gloria Vazquez; Hector Esteban Paz-Juarez; Hector Montoya-Fuentes; Helen Haydee Fernanda Ramirez-Plascencia; Jose Antonio Enciso-Moreno; Jose Esteban Munoz-Medina; Jose de Jesus Nunez-Contreras; Juan Bautista Chale-Dzul; Luis Alberto Ochoa-Carrera; Margarita Matias-Florentino; Maria Guadalupe Santiago-Mauricio; Maria Guadalupe de Jesus Mireles-Rivera; Nelly Selem-Mojica; Pavel Isa; Ricardo Grande; Santiago Ávila-Rios; Victor Eduardo Garcia-Arias; Victor Hugo Borja-Aburto
EPI_ISL_1351549, EPI_ISL_1351552, EPI_ISL_1351617	Centro de InvestigaciÃ³n BiomÃ©dica de Occidente (CIBO)	Unidad de Genomica Avanzada	Alejandro Sanchez-Flores; Alfredo Herrera-Estrella; Alicia Ocana-Mondragon; Angel Gustavo Salas-Lais; Bernardo Martinez-Miguel; Blanca Taboada; Brenda Irasema Maldonado-Meza; Carla Ivon Herrera-Najera; Carlos F. Arias; Celia Boukadida; Clara Esperanza Santacruz-Tinoco; Concepcion Grajales-Muniz; Consorcio Mexicano de Vigilancia Genomica (CoViGen-Mex). Authors (in alphabetical order): Julio Elias Alvarado-Yaah; Fernando Fontove-Herrera; Francisco Pulido; Gloria Elena Espinoza-Ayala; Gloria Maria Molina-Salinas; Gloria Vazquez; Hector Esteban Paz-Juarez; Hector Montoya-Fuentes; Helen Haydee Fernanda Ramirez-Plascencia; Jorge Ivan Salinal-Nevarez; Jose Antonio Enciso-Moreno; Jose Esteban Munoz-Medina; Jose de Jesus Nunez-Contreras; Juan Bautista Chale-Dzul; Luis Alberto Ochoa-Carrera; Margarita Matias-Florentino; Maria Guadalupe Santiago-Mauricio; Maria Guadalupe de Jesus Mireles-Rivera; Nelly Selem-Mojica; Pavel Isa; Ricardo Grande; Santiago Avila-Rios; Victor Hugo Borja-Aburto
EPI_ISL_1351416, EPI_ISL_1351437, EPI_ISL_1351439	Centro de InvestigaciÃ³n BiomÃ©dica del Noreste (CIBIN)	Unidad de Genomica Avanzada	Alejandro Sanchez-Flores; Alfredo Herrera-Estrella; Alicia Ocana-Mondragon; Angel Gustavo Salas-Lais; Bernardo Martinez-Miguel; Blanca Taboada; Brenda Irasema Maldonado-Meza; Carla Ivon Herrera-Najera; Carlos F. Arias; Celia Boukadida; Clara Esperanza Santacruz-Tinoco; Concepcion Grajales-Muniz; Consorcio Mexicano de Vigilancia Genomica (CoViGen-Mex). Authors (in alphabetical order): Julio Elias Alvarado-Yaah; Fernando Fontove-Herrera; Francisco Pulido; Gloria Elena Espinoza-Ayala; Gloria Maria Molina-Salinas; Gloria Vazquez; Hector Esteban Paz-Juarez; Hector Montoya-Fuentes; Helen Haydee Fernanda Ramirez-Plascencia; Jorge Ivan Salinal-Nevarez; Jose Antonio Enciso-Moreno; Jose Esteban Munoz-Medina; Jose de Jesus Nunez-Contreras; Juan Bautista Chale-Dzul; Luis Alberto Ochoa-Carrera; Margarita Matias-Florentino; Maria Guadalupe Santiago-Mauricio; Maria Guadalupe de Jesus Mireles-Rivera; Nelly Selem-Mojica; Pavel Isa; Ricardo Grande; Santiago Avila-Rios; Victor Hugo Borja-Aburto
EPI_ISL_2490395, EPI_ISL_2490398, EPI_ISL_2490495	Centro de Investigación Biomédica de Occidente (CIBO)	Centro de Investigación en Enfermedades Infecciosas (CIENI), Instituto Nacional de Enfermedades Respiratorias (INER)	; Alejandra Garcia-Gasca; Alejandra Hernández-Terán; Alejandro Sanchez-Flores; Alfredo Herrera-Estrella; Alicia Ocaña-Mondragón; Andreu Comas-García; Angel Gustavo Salas-Lais; Antonio Loza Román; Bernardo Martínez-Miguel; Blanca Taboada; Brenda Irasema Maldonado-Meza; Bruno Gomez-Gil; Carla Ivón Herrera-Najera; Carlos F. Arias; Celia Boukadida; Clara Esperanza Santacruz-Tinoco; Concepción Grajales-Muñiz; Consorcio Mexicano de Vigilancia Genómica (CoViGen-Mex). Authors (in alphabetical order): Julio Elias Alvarado-Yaah; Cristóbal Cháidez-Quiróz; Célida Duque Molina; Célida Martínez- Rodríguez; Daniel Fregoso-Rueda; Daniel Lira Morales; Eduardo Becerril-Vargas; Fernando Fontove-Herrera; Fidencio Mejía-Nepomuceno; Francisco Pulido; Gloria Elena Espinosa-Ayala; Gloria María Molina-Salinas; Gloria Vazquez; Hector Esteban Paz-Juárez; Hector Montoya-Fuentes; Helen Haydee Fernanda Ramírez-Plascencia; Irvin González-López; Jean Pierre González; Joel Armando Vázquez-Pérez.; Jorge Salas-Hernández; José Antonio Enciso-Moreno; José Arturo Martínez-Orozco; José Esteban Muñoz-Medina; José de Jesús Nuñez-Contreras; Juan Bautista Chale-Dzul; Julissa Enciso-Ibarra; Luis Alberto Ochoa-Carrera; Margarita Matías-Florentino; Mario Mújica-Sánchez; Marissa Perez-Garcia; María Guadalupe Santiago-Mauricio; María Guadalupe de Jesús Mireles-Rivera; Nelly Sélem-Mojica; Pavel Isa; Ricardo Ciria Merce; Ricardo Grande; Rosa María Gutierrez Rios; Santiago Ávila-Ríos; Selene Zárate; Susana Lopez; Victor Eduardo Garcia-Arias; Víctor Hugo Borja-Aburto
EPI_ISL_1585494	Centro de Investigación Biomédica de Occidente (CIBO)	Instituto Nacional de Enfermedades Respiratorias (INER): Centro de Investigación en Enfermedades Infecciosas (CIENI)	Alejandro Sanchez-Flores; Alfredo Herrera-Estrella; Alicia Ocaña-Mondragón; Angel Gustavo Salas-Lais; Bernardo Martinez-Miguel; Blanca Taboada; Brenda Irasema Maldonado-Meza; Carla Ivón Herrera-Najera; Carlos F. Arias; Celia Boukadida; Clara Esperanza Santacruz-Tinoco; Concepción Grajales-Muñiz; Consorcio Mexicano de Vigilancia Genómica (CoViGen-Mex). Authors (in alphabetical order): Julio Elias Alvarado-Yaah; Célida Duque Molina; Fernando Fontove-Herrera; Francisco Pulido; Gloria Elena Espinosa-Ayala; Gloria María Molina-Salinas; Gloria Vazquez; Hector Esteban Paz-Juárez; Hector Montoya-Fuentes; Helen Haydee Fernanda Ramírez-Plascencia; José Antonio Enciso-Moreno; José Esteban Muñoz-Medina; José de Jesús Nuñez-Contreras; Juan Bautista Chale-Dzul; Luis Alberto Ochoa-Carrera; Margarita Matías-Florentino; María Guadalupe Santiago-Mauricio; María Guadalupe de Jesús Mireles-Rivera; Nelly Sélem-Mojica; Pavel Isa; Ricardo Grande; Santiago Ávila-Ríos; Victor Eduardo Garcia-Arias; Víctor Hugo Borja-Aburto
EPI_ISL_2402142, EPI_ISL_2402153	Centro de Investigación Biomédica de Occidente (CIBO)	Unidad de Genomica Avanzada	Alejandro Sanchez-Flores; Alfredo Herrera-Estrella; Alicia Ocana-Mondragon; Angel Gustavo Salas-Lais; Bernardo Martinez-Miguel; Blanca Taboada; Brenda Irasema Maldonado-Meza; Carla Ivon Herrera-Najera; Carlos F. Arias; Celia Boukadida; Clara Esperanza Santacruz-Tinoco; Concepcion Grajales-Muniz; Consorcio Mexicano de Vigilancia Genomica (CoViGen-Mex). Authors (in alphabetical order): Julio Elias Alvarado-Yaah; Fernando Fontove-Herrera; Francisco Pulido; Gloria Elena Espinoza-Ayala; Gloria Maria Molina-Salinas; Gloria Vazquez; Hector Esteban Paz-Juarez; Hector Montoya-Fuentes; Helen Haydee Fernanda Ramirez-Plascencia; Jorge Ivan Salinal-Nevarez; Jose Antonio Enciso-Moreno; Jose Esteban Munoz-Medina; Jose de Jesus Nunez-Contreras; Juan Bautista Chale-Dzul; Luis Alberto Ochoa-Carrera; Margarita Matias-Florentino; Maria Guadalupe Santiago-Mauricio; Maria Guadalupe de Jesus Mireles-Rivera; Nelly Selem-Mojica; Pavel Isa; Ricardo Grande; Santiago Avila-Rios; Victor Hugo Borja-Aburto
EPI_ISL_2091174, EPI_ISL_2091179, EPI_ISL_2091183, EPI_ISL_2091193, EPI_ISL_2091208, EPI_ISL_2490339, EPI_ISL_2490442			
see above	Centro de Investigación Biomédica del Noreste (CIBIN)	Centro de Investigación en Enfermedades Infecciosas (CIENI), Instituto Nacional de Enfermedades Respiratorias (INER)	; Alejandra Garcia-Gasca; Alejandra Hernández-Terán; Alejandro Sanchez-Flores; Alfredo Herrera-Estrella; Alicia Ocaña-Mondragón; Andreu Comas-García; Angel Gustavo Salas-Lais; Antonio Loza Román; Bernardo Martínez-Miguel; Blanca Taboada; Brenda Irasema Maldonado-Meza; Bruno Gomez-Gil; Carla Ivón Herrera-Najera; Carlos F. Arias; Celia Boukadida; Clara Esperanza Santacruz-Tinoco; Concepción Grajales-Muñiz; Consorcio Mexicano de Vigilancia Genómica (CoViGen-Mex). Authors (in alphabetical order): Julio Elias Alvarado-Yaah; Cristóbal Cháidez-Quiróz; Célida Duque Molina; Célida Martínez- Rodríguez; Daniel Fregoso-Rueda; Daniel Lira Morales; Eduardo Becerril-Vargas; Fernando Fontove-Herrera; Fidencio Mejía-Nepomuceno; Francisco Pulido; Gloria Elena Espinosa-Ayala; Gloria María Molina-Salinas; Gloria Vazquez; Hector Esteban Paz-Juárez; Hector Montoya-Fuentes; Helen Haydee Fernanda Ramírez-Plascencia; Irvin González-López; Jean Pierre González; Joel Armando Vázquez-Pérez.; Jorge Salas-Hernández; José Antonio Enciso-Moreno; José Arturo Martínez-Orozco; José Esteban Muñoz-Medina; José de Jesús Nuñez-Contreras; Juan Bautista Chale-Dzul; Julissa Enciso-Ibarra; Luis Alberto Ochoa-Carrera; Margarita Matías-Florentino; Mario Mújica-Sánchez; Marissa Perez-Garcia; María Guadalupe Santiago-Mauricio; María Guadalupe de Jesús Mireles-Rivera; Nelly Sélem-Mojica; Pavel Isa; Ricardo Ciria Merce; Ricardo Grande; Rosa María Gutierrez Rios; Santiago Ávila-Ríos; Selene Zárate; Susana Lopez; Victor Eduardo Garcia-Arias; Víctor Hugo Borja-Aburto
EPI_ISL_1279502, EPI_ISL_1279529, EPI_ISL_1585390	Centro de Investigación Biomédica del Noreste (CIBIN)	Instituto Nacional de Enfermedades Respiratorias (INER): Centro de Investigación en Enfermedades Infecciosas (CIENI)	Alejandro Sanchez-Flores; Alfredo Herrera-Estrella; Alicia Ocaña-Mondragón; Angel Gustavo Salas-Lais; Bernardo Martinez-Miguel; Blanca Taboada; Brenda Irasema Maldonado-Meza; Carla Ivón Herrera-Najera; Carlos F. Arias; Celia Boukadida; Clara Esperanza Santacruz-Tinoco; Concepción Grajales-Muñiz; Consorcio Mexicano de Vigilancia Genómica (CoViGen-Mex). Authors (in alphabetical order): Julio Elias Alvarado-Yaah; Consorcio Mexicano de Vigilancia Genómica (CoViGen-Mex). Authors (in alphabetical order): Julio Elias Alvarado-Yaah; Célida Duque Molina; Fernando Fontove-Herrera; Francisco Pulido; Gloria Elena Espinosa-Ayala; Gloria Elena Espinoza-Ayala; Gloria María Molina-Salinas; Gloria Vazquez; Hector Esteban Paz-Juárez; Hector Montoya-Fuentes; Helen Haydee Fernanda Ramírez-Plascencia; Jorge Ivan Salinal-Nevarez; José Antonio Enciso-Moreno; José Esteban Muñoz-Medina; José de Jesús Nuñez-Contreras; Juan Bautista Chale-Dzul; Luis Alberto Ochoa-Carrera; Margarita Matías-Florentino; María Guadalupe Santiago-Mauricio; María Guadalupe de Jesús Mireles-Rivera; Nelly Sélem-Mojica; Pavel Isa; Ricardo Grande; Santiago Ávila-Ríos; Victor Eduardo Garcia-Arias; Víctor Hugo Borja-Aburto
EPI_ISL_2391588	Centro de Investigación Biomédica del Noreste (CIBIN)	Instituto de Biotecnología de la UNAM	; Alejandra Garcia-Gasca; Alejandra Hernández-Terán; Alejandro Sanchez-Flores; Alfredo Herrera-Estrella; Alicia Ocaña-Mondragón; Andreu Comas-García; Angel Gustavo Salas-Lais; Antonio Loza Román; Bernardo Martínez-Miguel; Blanca Taboada; Brenda Irasema Maldonado-Meza; Bruno Gomez-Gil; Carla Ivón Herrera-Najera; Carlos F. Arias; Celia Boukadida; Clara Esperanza Santacruz-Tinoco; Concepción Grajales-Muñiz; Consorcio Mexicano de Vigilancia Genómica (CoViGen-Mex). Authors (in alphabetical order): Julio Elias Alvarado-Yaah; Cristóbal Cháidez-Quiróz; Célida Duque Molina; Célida Martínez- Rodríguez; Daniel Fregoso-Rueda; Daniel Lira Morales; Eduardo Becerril-Vargas; Fernando Fontove-Herrera; Fidencio Mejía-Nepomuceno; Francisco Pulido; Gloria Elena Espinosa-Ayala; Gloria María Molina-Salinas; Gloria Vazquez; Hector Esteban Paz-Juárez; Hector Montoya-Fuentes; Helen Haydee Fernanda Ramírez-Plascencia; Irvin González-López; Jean Pierre González; Joel Armando Vázquez-Pérez.; Jorge Salas-Hernández; José Antonio Enciso-Moreno; José Arturo Martínez-Orozco; José Esteban Muñoz-Medina; José de Jesús Nuñez-Contreras; Juan Bautista Chale-Dzul; Julissa Enciso-Ibarra; Luis Alberto Ochoa-Carrera; Margarita Matías-Florentino; Mario Mújica-Sánchez; María Guadalupe Santiago-Mauricio; María Guadalupe de Jesús Mireles-Rivera; Nelly Sélem-Mojica; Pavel Isa; Ricardo Ciria Merce; Ricardo Grande; Rosa María Gutierrez Rios; Santiago Ávila-Ríos; Selene Zárate; Susana Lopez; Victor Eduardo Garcia-Arias; Víctor Hugo Borja-Aburto
EPI_ISL_2401935, EPI_ISL_2401949	Centro de Investigación Biomédica del Noreste (CIBIN)	Unidad de Genomica Avanzada	Alejandro Sanchez-Flores; Alfredo Herrera-Estrella; Alicia Ocana-Mondragon; Angel Gustavo Salas-Lais; Bernardo Martinez-Miguel; Blanca Taboada; Brenda Irasema Maldonado-Meza; Carla Ivon Herrera-Najera; Carlos F. Arias; Celia Boukadida; Clara Esperanza Santacruz-Tinoco; Concepcion Grajales-Muniz; Consorcio Mexicano de Vigilancia Genomica (CoViGen-Mex). Authors (in alphabetical order): Julio Elias Alvarado-Yaah; Fernando Fontove-Herrera; Francisco Pulido; Gloria Elena Espinoza-Ayala; Gloria Maria Molina-Salinas; Gloria Vazquez; Hector Esteban Paz-Juarez; Hector Montoya-Fuentes; Helen Haydee Fernanda Ramirez-Plascencia; Jorge Ivan Salinal-Nevarez; Jose Antonio Enciso-Moreno; Jose Esteban Munoz-Medina; Jose de Jesus Nunez-Contreras; Juan Bautista Chale-Dzul; Luis Alberto Ochoa-Carrera; Margarita Matias-Florentino; Maria Guadalupe Santiago-Mauricio; Maria Guadalupe de Jesus Mireles-Rivera; Nelly Selem-Mojica; Pavel Isa; Ricardo Grande; Santiago Avila-Rios; Victor Hugo Borja-Aburto
EPI_ISL_2442091, EPI_ISL_2442092, EPI_ISL_2442093, EPI_ISL_2442094, EPI_ISL_2442097	Centro de Investigação em Saúde de Manhiça (CISM) & ISGlobal, Institut de Salut Global de Barcelona	Instituto de Biomedicina de València - CSIC	Alfredo Mayor; Arsenia Massinga; Inacio Mandomando; Irving Cancino; Iñaki Comas; Manoli Torres Puente; Santiago Jiménez-Serrano
EPI_ISL_2620975	Centrum Medycyny Klinicznej dr n.med. Karol Majewski - DiMedical	1. Academic Center for Pathomorphological and Genetic-Molecular Diagnostics ltd, Bialystok, Poland 2. National Institute of Public Health - National Institute of Hygiene, Warsaw, Poland	Anetta Sulewska; Jacek Nikliński; Janusz Dzięcioł; Joanna Kiśluk; Katarzyna Zacharczuk; Konrad Raczkowski; Magdalena Nowakowska; Małgorzata Sadkowska-Todys; Piotr Karabowicz; Piotr Majewski; Przemysław Biecek. Joanna Reszeć; Radosław Charkiewicz; Tomasz Wołkowicz
EPI_ISL_2523859	City Hospital No 40	WHO National Influenza Centre Russian Federation	Andrey Komissarov; Artem Fadeev; Daria Danilenko; Dmitry Lioznov; Elena Nabieva; Georgii Bazykin; Kirill Varchenko; Ksenia Safina; Kseniya Komissarova; Maria Pisareva; Maria Timofeeva; Mikhail Bakaev; Nikita Yolshin; Olga Shneider; Oula Mansour; Sergey Scherbak; Tamila Musaeva; Veronika Eder
EPI_ISL_2617583	City of Milwaukee Health Department Laboratory	City of Milwaukee Health Department Laboratory	Amy Bauer; Jennifer Lentz; Manjeet Khubbar; Samantha Scott; Sanjib Bhattacharyya
EPI_ISL_2100243, EPI_ISL_2100244, EPI_ISL_2100245, EPI_ISL_2100246, EPI_ISL_2100247	Clinical Hospital of Infectious and Tropical Diseases “Dr. Victor Babes”	Cantacuzino Institute Virology	Carmen Cherciu; Mihaela Lazar; Mihaela Oprea; Sorin Dinu

EPI_ISL_1965786, EPI_ISL_2500703	Clinical Microbiology Laboratory, NewYork Presbyterian Hospital/Columbia University Irving Medical Center	Uhlemann Laboratory, Columbia University Irving Medical Center	Anne Kelley; Anne-Catrin Uhlemann; Maya Tagliavia; Medini K. Annavajhala
EPI_ISL_1239194, EPI_ISL_1968884, EPI_ISL_2103718	Clinical Microbiology, Infection Prevention and Control	Section for Molecular Diagnostics	Björn Hallström; Jonas Björkman
EPI_ISL_877661	Clinical Molecular Microbiology Laboratory, UNC Hospital	Dirk Dittmer	Angelica Juarez; Blossom Damania.; Brent A. Eason; Cameroon Grant; Carolina Caro-Vegas; Cecilia Thompson; Dirk Dittmer; Evelyn Hoffman; Jason Wong; Justin T. Landis; Linda Pluta; Melissa B. Miller; Patricio Cano; Razia Moorad
EPI_ISL_2608411	Clinical Research Centre, Sarawak General Hospital	Institute of Health and Community Medicine	Chan Chia Jui; Chua Hock Hin; David Perera; Ooi Mong How; Tonnii Sia Loong Loong; Wong Jyn Shan; Wong Kiing Aik
EPI_ISL_1748310, EPI_ISL_1833679, EPI_ISL_1993840, EPI_ISL_1993870, EPI_ISL_1993871, EPI_ISL_1993872, EPI_ISL_1993906, EPI_ISL_2484232			
see above	Clinical Virology	Clinical Bacteriology	Adrian Egli; Alfredo Mari; Fanny Wegner; Hans Hirsch; Helena MB Seth-Smith; Julia Bielicki; Karoline Leuzinger; Madlen Stange; Manuel Battegay; Tim Roloff
EPI_ISL_2035560	Clinical Virology	Clinical Virology	Anissa Chouikha; Henda Triki; Kais Ghedira; Mariem Gdoura; Sondos Haddad; Wasfi Fares
EPI_ISL_2272780, EPI_ISL_2272781, EPI_ISL_2272783, EPI_ISL_2272784, EPI_ISL_2272785, EPI_ISL_2272786	Clinical Hospital of Infectious and Tropical Diseases "Dr. Victor Babes"	Cantacuzino Institute Virology	Carmen Cherciu; Mihaela Lazar; Mihaela Oprea; Sorin Dinu; Usein Codruta
EPI_ISL_1534274	CoVid WC Garden Route	NHLS/UCT	Arash Iranzadeh; Bruna Galvao; Carolyn Williamson; Deelan Doolabh; Diana Hardie; Innocent Mudau; Kruger Marais; Lynn Tyers; Marvin Hsiao; Stephen Korsman
EPI_ISL_1539351, EPI_ISL_1539563, EPI_ISL_2195263, EPI_ISL_2309138, EPI_ISL_2309139, EPI_ISL_2309413, EPI_ISL_2309414, EPI_ISL_2310076, EPI_ISL_2310077, EPI_ISL_2310423, EPI_ISL_2310645, EPI_ISL_2311033			
see above	Colorado Department of Public Health and Environment	Colorado Department of Public Health and Environment	Alexandria Rossheim; Diana Ir; Emily A. Travanty; Laura Bankers; Molly C. Hetherington-Rauth; Sarah Elizabeth Totten; Shannon Ely; Shannon R. Matzinger
EPI_ISL_1516188, EPI_ISL_2383896	Commonwealth Healthcare Center	Centers for Disease Control and Prevention Division of Viral Diseases, Pathogen Discovery	Alison Laufer Halpin; Ben L. Rambo-Martin; Clinton R. Paden; Dakota Howard; Darlene Wagner; Dave Wentworth; Dhwani Batra; Jasmine Padilla; Justin Lee; Katie Dillon; Krista Queen; Kristen Knipe; Kristine Lacek; Mark Burroughs; Matthew Schmerer; Mili Sheth; Peter Cook; Sam Shepard; Sarah Nobles; Shoshona Le; Suxiang Tong; Vivien Dugan; Yvette Unoarumhi
EPI_ISL_1169510, EPI_ISL_1169542	Commonwealth Healthcare Center	Genomics and Discovery, Respiratory Viruses Branch, Division of Viral Diseases, Centers for Disease Control and Prevention	Anna Montmayeur; Anna Uehara; Ben L. Rambo-Martin; Clinton R. Paden; Dhwani Batra; Haibin Wang; Jasmine Padilla; Jing Zhang; Justin Lee; Krista Queen; Lori Rowe; Mark Burroughs; Mili Sheth; Peter W. Cook; Rachel Marine; Sarah Nobles; Suxiang Tong; Yan Li; Ying Tao
EPI_ISL_729743, EPI_ISL_729753	Connecticut Department of Health	Grubaugh Lab - Yale School of Public Health	Adam Moore; Akiko Iwasaki; Albert Ko; Alice Lu; Allison Nelson; Anderson Brito; Anne Wyllie; Annie Watkins; Arnau Casanovas; Catherine Muenker; Chaney Kalinich; Chantal Vogels; Charlese Dela Cruz; Isabel Ott; Joseph Fauver; Maria Tokuyama; Mary Petrone; Nathan Grubaugh; Patrick Wong; Peiwen Lu; Richard Martinello; Saad Omer; Shelli Farhadian; Tara Alpert
EPI_ISL_1017594	Connecticut Veterans' Affairs Hospital	Grubaugh Lab - Yale School of Public Health	Chantal Vogels; Danielle Plank; Isabel Ott; Joseph Fauver; Mary Petrone; Nathan Grubaugh; Shaili Gupta; Tara Alpert
EPI_ISL_2496919, EPI_ISL_2496920, EPI_ISL_2496924, EPI_ISL_2496932, EPI_ISL_2496933, EPI_ISL_2496935, EPI_ISL_2496936, EPI_ISL_2496938, EPI_ISL_2496939, EPI_ISL_2496942, EPI_ISL_2497675, EPI_ISL_2497677, EPI_ISL_2497678, EPI_ISL_2497679, EPI_ISL_2497681			
see above	Contra Costa County Public Health Lab	Chan-Zuckerberg Biohub	CZB Cliahub Consortium
EPI_ISL_1817707	Conville CDC wc CVC	NHLS/UCT	Arash Iranzadeh; Bruna Galvao; Carolyn Williamson; Deelan Doolabh; Diana Hardie; Innocent Mudau; Kruger Marais; Lynn Tyers; Marvin Hsiao; Stephen Korsman
EPI_ISL_2507131, EPI_ISL_2507150, EPI_ISL_2507152, EPI_ISL_2507163, EPI_ISL_2507197, EPI_ISL_2507199, EPI_ISL_2507231, EPI_ISL_2507356, EPI_ISL_2755034, EPI_ISL_2755117, EPI_ISL_2755119, EPI_ISL_2755122, EPI_ISL_2755176, EPI_ISL_2755181, EPI_ISL_2755207, EPI_ISL_2755788			
see above	Curative Labs	Curative Labs	Elias L. Salfati; Eugenia Khorosheva; George Way; J.Cesar Ignacio-Espinoza; Janet Chen; Mikhail Hanewich-Hollatz; Nabjot Sandhu; Sophia Quasem; Vladimir Slepnev; Zhiyi Xie
EPI_ISL_1446973	DC Public Health Lab/ Dept. of Forensic Sciences	Centers for Disease Control and Prevention Division of Viral Diseases, Pathogen Discovery	Alison Laufer Halpin; Ben L. Rambo-Martin; Clinton R. Paden; Dakota Howard; Darlene Wagner; Dave Wentworth; Dhwani Batra; Jasmine Padilla; Justin Lee; Katie Dillon; Krista Queen; Kristen Knipe; Kristine Lacek; Mark Burroughs; Matthew Schmerer; Mili Sheth; Peter Cook; Sam Shepard; Sarah Nobles; Shoshona Le; Suxiang Tong; Vivien Dugan; Yvette Unoarumhi
EPI_ISL_1753005, EPI_ISL_1819266, EPI_ISL_2176248	DC Public Health Lab/ Dept. of Forensic Sciences	DC Public Health Lab/ Dept. of Forensic Sciences	Brittany Hamilton; Connie Maza; David Payne; Elizabeth Zelaya; Janis Doss; Jocelyn Hauser; Monica Mann; Sarah Scott; Scott Nguyen
EPI_ISL_2342558	DNA Laboratories Sdn Bhd	Malaysia Genome Institute	Azrin Ahmad; Enizza Kasim; Irni Suhayu Sapian; Mohd Faizal Abu Bakar; Mohd Noor Mat Isa; Nor Azfa Johari.; Nurhezreen Md Iqbal; Shamsidar Sopie; Siti Noraini Othman; Wong Yong Wee; Yusuf Muhammad Noor
EPI_ISL_1337507	DOHMH Corona	New York City Public Health Laboratory	Jade Wang; et al.
EPI_ISL_1181690, EPI_ISL_1181692	DPHL	Delaware Public Health Lab	Gregory Hovan
EPI_ISL_2592630, EPI_ISL_2593764, EPI_ISL_2595725, EPI_ISL_2595726, EPI_ISL_2595860, EPI_ISL_2596341, EPI_ISL_2596803, EPI_ISL_2597228, EPI_ISL_2597229, EPI_ISL_2597312			
see above	DSMRC	DSMRC	Khine Zaw Oo; Ko Ko Lwin; Ko Ko Win; Kyee Myint; Nay Myo Aung; Pai Khant Kyaw; Phyo Kyaw Aung; Thet Wai Oo; Zaw Win Htun
EPI_ISL_593976	Delaware Public Health Lab	Delaware Public Health Lab	Gregory Hovan
EPI_ISL_693708, EPI_ISL_693711, EPI_ISL_693750, EPI_ISL_766979	Delaware Public Health Laboratory	Delaware Public Health Laboratory	Gregory Hovan
EPI_ISL_2432518, EPI_ISL_2432922, EPI_ISL_2432953	Demy Health	National Reference Laboratory, Nigeria Centre for Disease Control	Anthony Ahumibe; Catherine Okoi; Chimaobi Chukwu; Dr Chikwe Ihekweazu; Dr Ndodo Nnaemeka; Dr Omoare Adesuyi; Grace Esebanmen; Naidoo Dhamari; Nwando Mba; Olusola Anuoluwapo Akanbi
EPI_ISL_1340751, EPI_ISL_1340752, EPI_ISL_1340753, EPI_ISL_1340754, EPI_ISL_1340756, EPI_ISL_1340759, EPI_ISL_1340761, EPI_ISL_1340764			
see above	Departamento de Virologia, Laboratorio Central de Salud Pública, Avenida Venezuela y Teniente Escurra, Asunción, Paraguay	Laboratory of Respiratory Viruses and Measles, Oswaldo Cruz Institute, FIOCRUZ	Alice Sampaio Rocha; Ana Carolina Mendonca; Anna Carolina Paixao; Cynthia Vazquez; Fernando Motta; Luciana Appolinario; Marilda Siqueira on behalf of the Fiocruz COVID-19 Genomic Surveillance Network; Paola Resende; Renata Serrano Lopes
EPI_ISL_516923, EPI_ISL_516924	Department for Molecular Diagnostics, Centre for Medical Microbiology, Institute of Public Health of Montenegro	Charité Universitätsmedizin Berlin, Institut für Virologie	Barbara Muehlemann; Christian Drosten; Julia Schneider; Jörn Beheim-Schwarzbach; Marija Govedarica and Danijela Vujošević; Talitha Veith; Terry Jones; Victor M Corman
EPI_ISL_632934	Department of Acute Infectious Diseases Control and Prevention,Yunnan Provincial Center for Disease Control and Prevention	Department of Acute Infectious Diseases Control and Prevention,Yunnan Provincial Center for Disease Control and Prevention	Jienan Zhou; Meiling Zhang; Senquan Jia; Xiaonan Zhao; Xiaoqing Fu
EPI_ISL_1280117, EPI_ISL_1280147	Department of Genetics, Medirex	Laboratory of Genomics and Bioinformatics, Comenius University Science Park	Anna Gičová; Diana Rusňáková; Gabriel Minárik; Jaroslav Budiš; Miroslav Böhmer; Renáta Lukačková; Tatiana Sedláčková; Tomáš Szemes; Werner Krampl
EPI_ISL_417444	Department of Healthcare Biotechnology, National University of Sciences and Technology (NUST)	Department of Healthcare Biotechnology, National University of Sciences and Technology (NUST)	Corman; Ghani, E.; H.A.; Janjua; Javed, A.; Niazi; S.K.; Saqib, M.; V.M. and Zohaib, A.
EPI_ISL_413020	Department of Internal Medicine, Triemli Hospital	Institute of Medical Virology, University of Zurich	Alexandra Trkola; Andrea Zbinden; Fiona Steiner; Gerhard Eich; Jon Huder; Jürg Böni; Maryam Zaheri; Michael Huber; Patrick Redli; Riccarda Capaul; Stefan Schmutz; Verena Kufner
EPI_ISL_447619, EPI_ISL_534336, EPI_ISL_740547, EPI_ISL_1667471	Department of Laboratory Medicine, National Taiwan University Hospital	Microbial Genomics Core Lab, National Taiwan University Centers of Genomic and Precision Medicine	Chiao-Ling Li; Pei-Jer Chen; Shan-Chwen Chang; Shiou-Hwei Yeh; Sui-Yuan Chang; Ya-Yun Lai; You-Yu Lin
EPI_ISL_648741, EPI_ISL_648772	Department of Laboratory Medicine, Tan Tock Seng Hospital	Department of Laboratory Medicine, Tan Tock Seng Hospital	Barkham TMS; Chen YYC; Li C; Lim JX; Maurer-Stroh S; Nagarajan N; Sessions OM; Tang WY; Zair X
EPI_ISL_1919364, EPI_ISL_2491497	Department of Medical Microbiology - section Molde, Molde Hospital	Norwegian Institute of Public Health, Department of Virology	Atiya R Ali; Debech Nadia; Engebretsen Serina Beate; Garcia Llorente Ignacio; Hilde Elshaug; Hilde Vollan; Jon Bråte; Kamilla Heddeland Instefjord; Karoline Bragstad; Kathrine Stene-Johansen; Line Victoria Moen; Marie Paulsen Madsen; Olav Hungnes; Pedersen Benedikte Nevjen; Rasmus Riis Kopperud
EPI_ISL_977585	Department of Medical Microbiology, Hospital Pengajar Universiti Putra Malaysia	Malaysia Genome Institute	Avisha Richards; Azrin Ahmad; Enizza Kasim; Hui-Yee Chee; Irni Suhayu Sapian; Mohd Faizal Abu Bakar; Mohd Noor Mat Isa; Muhammad MI; Narcisse Joseph; Nor Azfa Johari; Nor Zahrin Hasran; Nurhezreen Md Iqbal; Shamsidar Sopie; Siti Noraini Othman; Syafinaz Amin-Nordin; Yusuf Muhammad Noor
EPI_ISL_501208, EPI_ISL_501227	Department of Medical Microbiology, University Malaya Medical Centre	Department of Medical Microbiology, Faculty of Medicine, University of Malaya	I-Ching SAM; Jennifer Chong; University Malaya Medical Centre COVID Team; Yoke Fun CHAN; Yoong Min CHONG
EPI_ISL_512844	Department of Medical Research	DMR_Myanmar	Aung Kyaw Kyaw; Aung Zaw Latt; Hlaing Myat Thu; Hnin Ohnmar Soe; Htin Lin; Kay Thi Aye; Lai Lai San; Myat Htut Nyunt; Nan Aye Thida Oo; Ni Ni Zaw; Phyu Win Ei; Su Mon Win; Theingi Win Myat; Wah Wah Aung; Yi Yi Kyaw; Zaw Than Htun
EPI_ISL_1495375, EPI_ISL_1495382, EPI_ISL_1495914	Department of Microbiology, University Innsbruck	Bergthaler laboratory, CeMM Research Center for Molecular Medicine of the Austrian Academy of Sciences	Andreas Bergthaler; Anna Schedl; Bekir Erguner; Benedikt Agerer; Christoph Bock; Fabian Amman; Jan Laine; Lukas Endler; Maelle Le Moing; Martin Senekowitsch; Michael Schuster; Petr Triska; Thomas Penz
EPI_ISL_2099879, EPI_ISL_2190101, EPI_ISL_2190107, EPI_ISL_2566446	Department of Public Health Bucharest	National Institute of Infectious Diseases-Prof. Dr. Matei Bals Molecular Diagnostics Laboratory	Andreea Tudor; Corina Casangiu; Dan Otelea; Leontina Banica; Marius Surleac; Ovidiu Vlaicu; Petre Milu; Robert Hohan; Simona Paraschiv
EPI_ISL_2566461, EPI_ISL_2566462, EPI_ISL_2566463, EPI_ISL_2566464	Department of Public Health Buzau	National Institute of Infectious Diseases-Prof. Dr. Matei Bals Molecular Diagnostics Laboratory	Corina Casangiu; Dan Otelea; Leontina Banica; Marius Surleac; Petre Milu; Robert Hohan; Simona Paraschiv

EPI_ISL_2324642	Department of Public Health Microbiology Ljubljana, National Laboratory for Health, Environment and Food	Department of Public Health Microbiology Ljubljana, National Laboratory for Health, Environment and Food	José Gonçalves; Katarina Prosenc; Marija Trkov; Martin Bosilj; Metka Paragi; Tom Koritnik; Verica Mioč
EPI_ISL_582513	Department of Respiratory and other Viral Infections of L.V.Gromashevsky Institute of Epidemiology & Infectious Diseases NAMS of Ukraine	Department of Respiratory and other Viral Infections of L.V.Gromashevsky Institute of Epidemiology & Infectious Diseases NAMS of Ukraine, JSC "Farmak"	Alla Mironenko; Andriy Goy; Ihor Kravchuk; Larysa Radchenko; Ludmyla Bolotova; Nataliia Teteriuk
EPI_ISL_732955	Department of Tropical Parasitology	Laboratory of Recombinant Vaccines	Boguslaw Szewczyk; Kirsi Aaltonen; Lukasz Rabalski; Maciej Grzybek; Maciej Kosinski; Ravi Kant; Tarja Sironen; Teemu Smura
EPI_ISL_1385791, EPI_ISL_1385808, EPI_ISL_1397958, EPI_ISL_1406393, EPI_ISL_1406394, EPI_ISL_1732274, EPI_ISL_2313099, EPI_ISL_2314185, EPI_ISL_2438666			
see above	Department of Virology	Department of Virology	Aamer Ikram; Abdul Ahad; Adnan Haider; Ammar Amjad; Austin Leach; Joel Montgomery; John Klena; Ketan Patel; Massab Umair; Melissa Mobley; Muhammad Ammar; Muhammad Salman; Nazish Badar; Qasim Ali; Sana Tamim; Shannon Whitmer; Syed Adnan Haider; Zaira Rehman
EPI_ISL_1819493	Dept. of Medical Microbiology, Stavanger University Hospital, Helse Stavanger HF	Norwegian Institute of Public Health, Department of Virology	Atiya R Ali; Debech Nadia; Engebretsen Serina Beate; Garcia Llorente Ignacio; Hilde Elshaug; Hilde Vollan; Jon Bråte; Kamilla Heddeland Instefjord; Karoline Bragstad; Kathrine Stene-Johansen; Marie Paulsen Madsen; Olav Hungnes; Pedersen Benedikte Nevjen; Rasmus Riis Kopperud
EPI_ISL_1118155, EPI_ISL_1322310, EPI_ISL_1731370, EPI_ISL_1994923, EPI_ISL_1994941, EPI_ISL_2566936, EPI_ISL_2566953			
see above	Dept. of Microbiology and Infection Control, Akershus University Hospital HF	Dept. of Microbiology and Infection Control, Akershus University Hospital HF	Alexander Hesselberg Løvestad; Hege Vangstein Aamot
EPI_ISL_2171041, EPI_ISL_2171281	Dhulikhel Hospital, Kathmandu University Hospital	Molecular and Genomics Research Lab, Dhulikhel Hospital, Kathmandu University Hospital	Dipesh Tamrakar; Manu Vanaerschot; Meghnath Dhimal; Navin Adhikari; Nishan Katuwal; Pradip Gyanwali; Rajeev Shrestha; Saroj Bhattarai; Surendra Kumar Madhup
EPI_ISL_2246972	Diagnostyka Sp. Z o.o.	1. Academic Center for Pathomorphological and Genetic-Molecular Diagnostics ltd, Bialystok, Poland 2. National Institute of Public Health - National Institute of Hygiene, Warsaw, Poland	Anetta Sulewska; Jacek Nikliński; Janusz Dzięcioł; Joanna Kiśluk; Katarzyna Zacharczuk; Konrad Raczkowski; Magdalena Nowakowska; Małgorzata Sadkowska-Todys; Piotr Karabowicz; Piotr Majewski; Przemysław Biecek. Joanna Reszeć; Radosław Charkiewicz; Tomasz Wołkowicz
EPI_ISL_2319093	Diagnostyka. Laboratoria Medyczne.	1. ViroGenetics - BSL3 Laboratory of Virology, Małopolska Centre of Biotechnology, Jagiellonian University; 2. genXone SA, Research & Development Laboratory	Brylak A; Drweska-Matelska N; Gidlewicz A; Grabowski J; Gromowski, T.; Januszczak S; Kaszuba M; Kowalski, M.; Krych L; Labaj; Mazur-Panasiuk, N.; Nowicki G; P.P.; Pyrc, K.; Sykulski M; Szeszko K; Szulc, P.; Wydmanski W.
EPI_ISL_747302, EPI_ISL_747312, EPI_ISL_747319, EPI_ISL_747349, EPI_ISL_747399, EPI_ISL_850183, EPI_ISL_850188, EPI_ISL_959500, EPI_ISL_1138940, EPI_ISL_1138975, EPI_ISL_1252440, EPI_ISL_1319005, EPI_ISL_1489580, EPI_ISL_1489958, EPI_ISL_1490143, EPI_ISL_1936604, EPI_ISL_2332276, EPI_ISL_2332456, EPI_ISL_2332565, EPI_ISL_2361252, EPI_ISL_2464316, EPI_ISL_2464341, EPI_ISL_2464343, EPI_ISL_2464344, EPI_ISL_2464345, EPI_ISL_2464346, EPI_ISL_2464354			
see above	Division of Emerging Infectious Diseases, Bureau of Infectious Diseases Diagnosis Control, Korea Disease Control and Prevention Agency	Division of Emerging Infectious Diseases, Bureau of Infectious Diseases Diagnosis Control, Korea Disease Control and Prevention Agency	Ae Kyung Park; Chae Young Lee; Chaeyoung Lee; Eun-Jin Kim; Heui Man Kim; Il-Hwan Kim; Jeong-Ah Kim; Jeong-Ah Kimg; Jeong-Min Kim; Jin Sun No; Namjoo Lee; Sang Hee Woo
EPI_ISL_2104744	Division of Epidemiology	COVID-19 Network Investigations (CONI) Alliance	Angkana Huang; Anthony R. Jones; Arporn Wangwiwatsin; Arthicha Wongkumma; Bhakbhoom Panthan; Chonticha Klungtong; Duangkamon Loesbanluechai; Ekawat Pasomsub; Elizabeth Batty; Insee Sensorn; Janjira Thaipadungpanit; Kawinna Kerdsalung; Khajohn Joonlasak; Kingkan Rakmanee; Krittikorn Kumpornsin; Namfon Kotanan; Pantila Taweewigyakarn; Prangsiri Nalaem; Stefan Fernandez; Thanat Chookajorn; Theerarat Kochakarn; Treewat Watthanachockchai; Wasun Chantratita; Wudtichai Manasatienkij
EPI_ISL_412871, EPI_ISL_481378, EPI_ISL_498045, EPI_ISL_510590, EPI_ISL_515037	Division of Viral Diseases, Center for Laboratory Control of Infectious Diseases, Korea Centers for Diseases Control and Prevention	Division of Viral Diseases, Center for Laboratory Control of Infectious Diseases, Korea Centers for Diseases Control and Prevention	Daesang Lee; Dong Hyun Song; Heui Man Kim; Hye-Jun Jo; Jeong-Min Kim; Jun-Sub Kim; Mi-Seon Kim; Myung Guk Han; Namjoo Lee; Sang Hee Woo; Sehee Park; Seong Tae Jeong; Yoon-Seok Chung
EPI_ISL_523211, EPI_ISL_578032, EPI_ISL_632485, EPI_ISL_722275, EPI_ISL_2145143, EPI_ISL_2145682, EPI_ISL_2145703, EPI_ISL_2145782, EPI_ISL_2449950, EPI_ISL_2449965, EPI_ISL_2449966, EPI_ISL_2449987, EPI_ISL_2449988, EPI_ISL_2449989, EPI_ISL_2483493			
see above	Dutch COVID-19 response team	Erasmus Medical Center	Anne van der Linden; Annemiek van der Eijk; Aura Timen; Bas Oude Munnink; Claudia Schapendonk; Corien Swaan; Corine GeurtsvanKessel; David Nieuwenhuijse; Emmanuelle Munger; Irina Chestakova; Jeroen van Kampen; Jolanda Voermans; Madelief Mollers; Manon Haverkate; Marion Koopmans; Marjan Boter; Mark Pronk; Mart Stein; OH consortium; Pascal Lexmond; Reina Sikkema; Richard Molenkamp; Sandra Kengne Kamga Mobou; Stefan van Nieuwkoop; Theo Bestebroer; on behalf of the Dutch national COVID-19 respo; on behalf of the Dutch national COVID-19 response team.
EPI_ISL_1158773, EPI_ISL_1443459	Dutch COVID-19 response team	Medical Microbiology, Maastricht University Medical Centre	Brian van der Veer*; Carmen Reumkens; Christian Hoebe; Erik Beuken; Jozef Dingemans*; Lieke van Alphen; Paul Savelkoul
EPI_ISL_943476, EPI_ISL_1013678, EPI_ISL_1035238, EPI_ISL_1089880, EPI_ISL_1165369, EPI_ISL_1232400, EPI_ISL_1287892, EPI_ISL_1289068, EPI_ISL_1370844, EPI_ISL_1456826, EPI_ISL_1520479, EPI_ISL_1520505, EPI_ISL_1521321, EPI_ISL_1522126, EPI_ISL_1522182, EPI_ISL_1522200, EPI_ISL_1522201, EPI_ISL_1522203, EPI_ISL_1522208, EPI_ISL_1522212, EPI_ISL_1522215, EPI_ISL_1522218, EPI_ISL_1595932, EPI_ISL_1596267, EPI_ISL_1596475, EPI_ISL_1596975, EPI_ISL_1705145, EPI_ISL_1705429, EPI_ISL_1705535, EPI_ISL_1705811, EPI_ISL_1792906, EPI_ISL_1792907, EPI_ISL_1962964, EPI_ISL_1962965, EPI_ISL_1962966, EPI_ISL_2094189, EPI_ISL_2094352, EPI_ISL_2094353, EPI_ISL_2094386, EPI_ISL_2094387, EPI_ISL_2094388, EPI_ISL_2094389, EPI_ISL_2094390, EPI_ISL_2094391, EPI_ISL_2094392, EPI_ISL_2094393, EPI_ISL_2215916, EPI_ISL_2216012, EPI_ISL_2220505, EPI_ISL_2220507, EPI_ISL_2220513, EPI_ISL_2220518, EPI_ISL_2303627, EPI_ISL_2303975, EPI_ISL_2303978, EPI_ISL_2405837, EPI_ISL_2474331, EPI_ISL_2476316, EPI_ISL_2610513, EPI_ISL_2610915, EPI_ISL_2610918, EPI_ISL_2673172, EPI_ISL_2673388, EPI_ISL_2788294, EPI_ISL_2788295, EPI_ISL_2788315			
see above	Dutch COVID-19 response team	National Institute for Public Health and the Environment (RIVM)	Adam Meijer; AnneMarie van den Brandt; Annelies Kroneman; Bas van der Veer; Chantal Reusken; Dennis Schmitz; Dirk Eggink; Euníce Then; Florian Zwagemaker; Harry Vennema; James Groot; Jeroen Cremer; Jolienke Hardeman; Karim Hajji; Kim Freriks; Linda van de Nes; Lisa Wijsman; Lynn Aarts; Melissa van Tuil; Robert Kohl; Ryanne Jaarsma; Sanne Bos; Sharon van den Brink; Sjoerd Kuiling; on behalf of the national COVID-19 response team
EPI_ISL_1915786, EPI_ISL_1915787	EHA Clinics	National Reference Laboratory, Nigeria Centre for Disease Control	Anthony Ahumibe; Catherine Okoi; Chimaobi Chukwu; Dr Chikwe Ihekweazu; Dr Ndodo Nnaemeka; Dr Omoare Adesuyi; Grace Esebanmen; Naidoo Dhamari; Nwando Mba; Olusola Anuoluwapo Akanbi
EPI_ISL_1913220, EPI_ISL_1913224	EL CAMINO HOSPITAL	Santa Clara County public Health Laboratory	Santa Clara County Public Health Department
EPI_ISL_1812276, EPI_ISL_1815579	EXCITE Lab	Andersen lab at Scripps Research	Alexandre Bolze; Alice Summerfield; Celena Andrade; Charlotte Rivera-Garcia; David Becker; Efren Sandoval; Elizabeth Cirulli; Francisco Tanudjaja; Geraint Levan; James Lu + SEARCH; Jason Nguyen; Jimmy Ramirez; Kelly Schiabor Barrett; Magnus Isaksson; Marc Laurent; Nicole L Washington; Ryan Cho; Sherry Wang; Simon White; Tyler Cassens; William Lee
EPI_ISL_2659068, EPI_ISL_2659069, EPI_ISL_2659070	El Camino Hospital	Santa Clara County Public Health Laboratory	Santa Clara County Public Health Department
EPI_ISL_1824117, EPI_ISL_1824118	El Camino Hospital	Santa Clara County public Health Laboratory	Santa Clara County Public Health Department
EPI_ISL_2321503	Emory University	Centers for Disease Control and Prevention Division of Viral Diseases, Pathogen Discovery	Alison Laufer Halpin; Ben L. Rambo-Martin; Clinton R. Paden; Dakota Howard; Darlene Wagner; Dave Wentworth; Dhwani Batra; Jasmine Padilla; Justin Lee; Katie Dillon; Krista Queen; Kristen Knipe; Kristine Lacek; Mark Burroughs; Matthew Schmerer; Mili Sheth; Peter Cook; Sam Shepard; Sarah Nobles; Shoshona Le; Suxiang Tong; Vivien Dugan; Yvette Unoarumhi
EPI_ISL_2135484	Epimol – Molecular Epidemiology Group (UV-FISABIO)	SeqCOVID-SPAIN consortium/IBV(CSIC)	María Alma Bracho and SeqCOVID-SPAIN consortium
EPI_ISL_883245, EPI_ISL_883825, EPI_ISL_1097646, EPI_ISL_1404993	Eurofins Diatherix	Hudsonalpha Genome Sequencing Center	Ada Stewart; Jane Grimwood; Jenell Webber; Jennifer Cart; John Lovell; Joshua Stough; Leslie Malone; Lori H. Handley; Melissa Williams; Stefan Brzezinski; Teresa Jones; and Jeremy Schmutz
EPI_ISL_419560	FL Bureau of Public Health Laboratories-Tampa	Pathogen Discovery, Respiratory Viruses Branch, Division of Viral Diseases, Centers for Disease Control and Prevention	Anna Uehara; Clinton R. Paden; Haibin Wang; Jasmine Padilla; Jing Zhang; Justin Lee; Krista Queen; Suxiang Tong; Yan Li; Ying Tao
EPI_ISL_1545668	Faculty of Medicine Vajira Hospital	COVID-19 Network Investigations (CONI) Alliance	Ampan Vimonvattana; Anan Manomaipiboon; Angkana Huang; Anthony R. Jones; Arporn Wangwiwatsin; Bhakbhoom Panthan; Chayanit Phutthanu; Chonticha Klungtong; Duangkamon Loesbanluechai; Ekawat Pasomsub; Elizabeth Batty; Insee Sensorn; Jakravoot Maneerit; Janjira Thaipadungpanit; Khajohn Joonlasak; Kingkan Rakmanee; Krittikorn Kumpornsin; Namfon Kotanan; Stefan Fernandez; Sunisa Dongphooyao; Thanat Chookajorn; Theerarat Kochakarn; Tonsan Hansirisathit; Treewat Watthanachockchai; Uraporn Phumisantiphong; Wasun Chantratita; Wipawee Thongsopa; Wudtichai Manasatienkij
EPI_ISL_1273102	Faculty of Medicine, Al-Quds University	Faculty of Medicine, Al-Quds University	Al-Jawabreh, A.; Dumaidi, K.; Ereqat, S.; Nasereddin, A.
EPI_ISL_2032644, EPI_ISL_2038893	Federal Budget Health Care Institution "Center of Hygiene and Epidemiology in Tver region"	Group of Genomics and Postgenomic Technologies of Central Research Institute of Epidemiology	Akimkin VG; Kaptelova VV; Kondrasheva LY; Korneenko EV; Melushenkova TG; Nadtoka MI; Saenko SS; Samoilov AE; Smirnova YS; Speranskaya AS; Tivanova EV
EPI_ISL_1859607	Federal State Budgetary Educational Institution of Higher Education B«Privolzhsky Research Medical UniversityB» of the Ministry of Health of the Russian Federation, Academic Consortium	FEDERAL RESEARCH AND CLINICAL CENTER OF PHYSICAL-CHEMICAL MEDICINE OF FEDERAL MEDICAL BIOLOGICAL AGENCY, Academic Consortium	Alexander Manolov; Alexander Pavlenko; Alexander Rumyantsev; Alexander Samoilov; Alexander Troitsky; Alexander Tulichev; Alexander Vasiliev; Alexey Volnukhin; Alla Petrova; Andrey Koritsky; Anna Blagonravova; Anna Varizhuk; Daria Boldyreva; Daria Kharlampieva; Daria Matyushkina; Dmitry Fedorov; Dmitry Pushkar; Ekaterina Moskaleva; Elena Galova; Elena Ilina; Elizaveta Orlova; Galina Trifonova; Gaukhar Yusubalieva; Ilshat Gafurov; Irina Shirokova; Konstantin Ivanov; Konstantin Kolontarev; Ksenia Klimina; Larisa Balykova; Lyubov Rychkova; Nadezhda Kolyshkina; Natalia Pozhenko; Oleg Ogarkov; Pavel Bashkirov; Polina Khromova; Polina Tikhonova; Ramiz Bakhtyev; Raushaniya Gaifullina; Sergey Rumyantsev; Svetlana Solovieva; Vadim Govorun; Valentin Manuvera; Varvara Musarova; Vasily Lazarev; Vladimir Baklaushev; Vladimir Rumyantsev; Vladimir Veselovsky; Vladislav Babenko; Vyacheslav Kuropatkin; Vyacheslav Sinkov; Yulia Galeeva; Yuri Udalov
EPI_ISL_2558053, EPI_ISL_2558057	First Aide Diagnostic Center	Philippine Genome Center	Alethea R. de Guzman; Anna Ong-Lim; Arianne A. Zamora; Asia Louisa U. Chong; Benedict A. Maralit; Candice Francheska B. Tambaoan; Carlo M. Lapid; Celia Carlos; Devon Ray Pacial; Edsel Maurice Salvaña; El King D. Morado; Elcid Aaron R. Pangilinan; Eva Maria Cutiongco-de la Paz; Francis A. Tablizo; Irish Coleen A. Asin; Jaime C. Montoya; Jan Michael C. Yap; Jo-Hannah S. Llames; John Q. Wong; Joshua Gregor A. Dizon; Juan Antonio R. Magalang; Karol Sophia Agape R. Padilla; Kenneth M. Kim; Kris P. Punayan; Marc Edsel C. Ayes; Maria Rosario Singh-Vergeire and Cynthia P. Saloma; Maria Sofia L. Yangzon; Marissa Alejandria; Razel Nikka M. Hao; Renato Jacinto Q. Mantaring; Rianna Patricia S. Cruz; Sheila Mae M. Araiza
EPI_ISL_508720, EPI_ISL_1674735, EPI_ISL_2008869, EPI_ISL_2230013, EPI_ISL_2246887, EPI_ISL_2246888, EPI_ISL_2246889			
see above	Florida Bureau of Public Health Laboratories	Florida Bureau of Public Health Laboratories	Jason Blanton; Sarah Schmedes
EPI_ISL_476139	Folkhalsomyndigheten	The Public Health Agency of Sweden	Anna Risberg; Anna-Malin Linde; Karin Tegmark-Wisell; Maria Lind Karlberg; Mattias Haukland; Olov Svartstrom; Oskar Karlsson Lindsjo; Petra Edquist; Reza Advani; Sandra Broddesson; Shamam Muradrasoli
EPI_ISL_1557163, EPI_ISL_1614854, EPI_ISL_1666166, EPI_ISL_1666372, EPI_ISL_1666373, EPI_ISL_1666377, EPI_ISL_1666379, EPI_ISL_1666384, EPI_ISL_1666437, EPI_ISL_1666438, EPI_ISL_1666447, EPI_ISL_1666495, EPI_ISL_1667140, EPI_ISL_1667173, EPI_ISL_1667317, EPI_ISL_1667332, EPI_ISL_1667390, EPI_ISL_1839105, EPI_ISL_1839106, EPI_ISL_1839341, EPI_ISL_1839348, EPI_ISL_1839441, EPI_ISL_1839578, EPI_ISL_1839605, EPI_ISL_1839969, EPI_ISL_1840029, EPI_ISL_1840145, EPI_ISL_1992039, EPI_ISL_1992110, EPI_ISL_1992125, EPI_ISL_1992133, EPI_ISL_1992137, EPI_ISL_1992142, EPI_ISL_1992240, EPI_ISL_1992245, EPI_ISL_1992266, EPI_ISL_1992270, EPI_ISL_1992271, EPI_ISL_1992272, EPI_ISL_1992302, EPI_ISL_1992395, EPI_ISL_1992594, EPI_ISL_1992667, EPI_ISL_1992684, EPI_ISL_1992823, EPI_ISL_1992943, EPI_ISL_1992993, EPI_ISL_1993014, EPI_ISL_1993031, EPI_ISL_1993033, EPI_ISL_1993034, EPI_ISL_1993045, EPI_ISL_1993068, EPI_ISL_1993069, EPI_ISL_1993113, EPI_ISL_1993142, EPI_ISL_1993160, EPI_ISL_1993161, EPI_ISL_1993163, EPI_ISL_1993295, EPI_ISL_1993297, EPI_ISL_2038558, EPI_ISL_2038729, EPI_ISL_2038753, EPI_ISL_2043610, EPI_ISL_2043635, EPI_ISL_2043905, EPI_ISL_2043918, EPI_ISL_2134475, EPI_ISL_2134533, EPI_ISL_2134535, EPI_ISL_2480006, EPI_ISL_2480014, EPI_ISL_2525759, EPI_ISL_2525769, EPI_ISL_2598756, EPI_ISL_2598771, EPI_ISL_2686716			
see above	Fulgent Genetics	Centers for Disease Control and Prevention Division of Viral Diseases, Pathogen Discovery	Adrian Paskey; Becky Tsai; Benafsh Sapra; Benjamin Rambo-Martin; Christopher Gulvick; Clinton R. Paden; Dakota Howard; Darlene Wagner; Dhwani Batra; Doreen Ng; Duncan MacCannell; Harry Gao; James Xie; Jason Caravas; John Gao; Joseph Fierro; Kara Moser; Matthew Schmerer; Mickey Li; Peter W. Cook; Scott Sammons; Shatavia Morrison; Yan Meng; Yvette Unoarumhi
EPI_ISL_1232130, EPI_ISL_1937710	Fulgent Genetics	Fulgent Genetics	Becky Tsai; Benafsh Sapra; Doreen Ng; Harry Gao; James Xie; John Gao; Joseph Fierro; Mickey Li; Yan Meng
EPI_ISL_1110540,	Furst Medical Laboratory	Norwegian Institute of Public Health, Department of Virology	'Kathrine Stene-Johansen; Atiya R Ali; Debech Nadia; Engebretsen Serina Beate; Garcia Llorente Ignacio; Hilde Elshaug; Hilde Vollan; Jon Bråte; Kamilla Heddeland Instefjord; Karoline Bragstad; Kathrine Stene-Johansen; Line Victoria Moen; Marie Paulsen

EPI_ISL_1317425, EPI_ISL_1578753, EPI_ISL_1578825, EPI_ISL_1994063, EPI_ISL_2333503			Madsen; Olav Hungnes; Pedersen Benedikte Nevjen; Rasmus Riis Kopperud
EPI_ISL_2467521, EPI_ISL_2629587	GA Department of Public Health	GA Department of Public Health	Aliyah Fields; Cynthia Dixey; Jonathan Edwards; Stacy Reeves; Taylor Smith; Tonia Parrott
EPI_ISL_1528524	GA Department of Public Health Laboratory	Centers for Disease Control and Prevention Division of Viral Diseases, Pathogen Discovery	Alison Laufer Halpin; Ben L. Rambo-Martin; Clinton R. Paden; Dakota Howard; Darlene Wagner; Dave Wentworth; Dhwani Batra; Jasmine Padilla; Justin Lee; Katie Dillon; Krista Queen; Kristen Knipe; Kristine Lacek; Mark Burroughs; Matthew Schmerer; Mili Sheth; Peter Cook; Sam Shepard; Sarah Nobles; Shoshona Le; Suxiang Tong; Vivien Dugan; Yvette Unoarumhi
EPI_ISL_2311763, EPI_ISL_2323452, EPI_ISL_2376424, EPI_ISL_2422459, EPI_ISL_2692535	GA Department of Public Health Laboratory	Genomics and Discovery, Respiratory Viruses Branch, Division of Viral Diseases, Centers for Disease Control and Prevention	Adam Retchless; Anna Kelleher; Anna Uehara; Brian Lynch; Clinton R. Paden; Dhwani Batra; Haibin Wang; Han Jia Justin Ng; Jasmine Padilla; Jing Zhang; Justin Lee; Krista Queen; Mark Burroughs; Mili Sheth; Morgan Davis; Peter Cook; Rachel Marine; Sarah Nobles; Suxiang Tong; Tara Coalter; Yan Li; Ying Tao
EPI_ISL_812178	GA Department of Public Health Laboratory	Pathogen Discovery, Respiratory Viruses Branch, Division of Viral Diseases, Centers for Disease Control and Prevention	Anna Montmayeur; Anna Uehara; Brian Lynch; Clinton R. Paden; Haibin Wang; Jing Zhang; Krista Queen; Peter Cook; Rachel Marine; Suxiang Tong; Yan Li; Ying Tao
EPI_ISL_678252	General Hospital - Kumanovo	Research Center for Genetic Engineering and Biotechnology "Georgi D. Efremov" , Macedonian Academy of Sciences and Arts	RCGEB - MASA
EPI_ISL_1970999	General Hospital - Prilep	Research Center for Genetic Engineering and Biotechnology "Georgi D. Efremov" , Macedonian Academy of Sciences and Arts	Aleksandar J. Dimovski; Dijana Plasheska-Karanfilska; Gjorgji Bozinovski; Milena Jakimovska; Predrag Noveski
EPI_ISL_918486	General Hospital - Struga	Research Center for Genetic Engineering and Biotechnology "Georgi D. Efremov" , Macedonian Academy of Sciences and Arts	RCGEB - MASA
EPI_ISL_1972216, EPI_ISL_2001037	General Hospital - Tetovo	Research Center for Genetic Engineering and Biotechnology "Georgi D. Efremov" , Macedonian Academy of Sciences and Arts	Aleksandar J. Dimovski; Dijana Plasheska-Karanfilska; Gjorgji Bozinovski; Milena Jakimovska; Predrag Noveski
EPI_ISL_934415	General Hospital - Veles	Research Center for Genetic Engineering and Biotechnology "Georgi D. Efremov" , Macedonian Academy of Sciences and Arts	RCGEB - MASA
EPI_ISL_406798	General Hospital of Central Theater Command of People's Liberation Army of China	BGI & Institute of Microbiology, Chinese Academy of Sciences & Shandong First Medical University & Shandong Academy of Medical Sciences & General Hospital of Central Theater Command of People's Liberation Army of China	Weifeng Shi and Zhenhong Hu; Weijun Chen; Yuhai Bi
EPI_ISL_735325, EPI_ISL_735361	Genomic Laboratory (GLAB) (Conjoint lab of Health Directorate of Istanbul and Istanbul Technical University)	Genomic Laboratory (GLAB), Istanbul Technical University	Arzu Irvem; Ayse Serra Ozel; Betsi Kose; Gizem Alkurt; Gizem Dinler Doganay; Ilker Karacan; Jale Yildiz; Levent Doganay; Mehtap Aydin; Nihat Bugra Agaoglu; Nilsun Altunal; Nisan Denizce Can; Ozlem Akgun Dogan; Payam Zolfagharian; Tugba Kizilboga Akgun; Yasemin Kendir Demirkol
EPI_ISL_2659073	Good Samaritan Hospital	Santa Clara County Public Health Laboratory	Santa Clara County Public Health Department
EPI_ISL_415152	Gorgas Memorial Institute for Health Studies	Gorgas Memorial Institute for Health Studies	Alexander A. Martinez.; Ambar Moreno; Claudia Gonzalez; Danilo Franco; Elimelec Valdespino; Juan M. Pascale; Leyda Abrego; Oris Chavarria; Sandra Lopez-Verges; Yamilka Diaz
EPI_ISL_1502891, EPI_ISL_1502957, EPI_ISL_1502988, EPI_ISL_1503101, EPI_ISL_1503109, EPI_ISL_1503110, EPI_ISL_1503119			
see above	Gorgas Memorial Laboratory of Health Studies	Gorgas Memorial Laboratory of Health Studies	Castillo Jorge; Franco Danilo; Gonzalez Claudia; Jessica Gondola; Leyda Abrego; Lopez-Verges Sandra; Marlenne Castillo; Martinez Alexander; Moreno Ambar; Moreno Brechla; Oris Chavarria; Ortiz Alma
EPI_ISL_2105482	Governor Celestino Gallares Memorial Hospital	Philippine Genome Center	Alethea R. de Guzman; Anna Ong-Lim; Arianne A. Zamora; Asia Louisa U. Chong; Benedict A. Maralit; Candice Francheska B. Tambaoan; Carlo M. Lapid; Celia Carlos; Devon Ray Pacial; Edsel Maurice Salvaña; El King D. Morado; Eva Maria Cutiongco-de la Paz; Francis A. Tablizo; Irish Coleen A. Asin; Jaime C. Montoya; Jan Michael C. Yap; Jo-Hannah S. Llames; John Q. Wong; Joshua Gregor A. Dizon; Juan Antonio R. Magalang; Karol Sophia Agape R. Padilla; Kenneth M. Kim; Kris P. Punayan; Marc Edsel C. Ayes; Marc Jerrone R. Castro; Maria Rosario Singh-Vergeire and Cynthia P. Saloma; Maria Sofia L. Yangzon; Marissa Alejandria; Razel Nikka M. Hao; Rianna Patricia S. Cruz; Sheila Mae M. Araiza
EPI_ISL_640043	Groote Schuur Hospital wc GSH	NHLS/UCT	Arash Iranzadeh; Bruna Galvao; Carolyn Williamson; Deelan Doolabh; Diana Hardie; Innocent Mudau; Kruger Marais; Lynn Tyers; Marvin Hsiao; Stephen Korsman
EPI_ISL_699040, EPI_ISL_699161	Group 42 (G42) Healthcare, Abu Dhabi, United Arab Emirates; Department of Health, The United Arab Emirates	G42 Healthcare	Ashish Koshy; Budoor Alqarni; Denghui Liu; Fang Chen; Hanif Khalak; Huanming Yang; Javier Quilez; Jian Wang; Junhua Li; Ke Liang; Long Lin; Mohammed Saifuddin Fasihuddin; Nan Qiao; Nawal Ahmed Mohamed Al Kaabi; Pauline Ogrodzki; Pei Wu; Peng Xiao; Pengjuan Liu; Rong Liu; Sally Mahmoud; Siyang Liu; Stephen S. Francis; Tao Ma; Vinay Kusuma; Walid Abbas Zaher; Weibin Liu; Wenjun He; Xavier Anton; Xin Jin; Xin Meng; Xinyu Huang; Xun Xu; Zhaorong Yuan
EPI_ISL_1798902, EPI_ISL_1798906, EPI_ISL_1823584, EPI_ISL_2229105, EPI_ISL_2451679, EPI_ISL_2451690	Guam Public Health Laboratory	Centers for Disease Control and Prevention Division of Viral Diseases, Pathogen Discovery	Alison Laufer Halpin; Ben L. Rambo-Martin; Clinton R. Paden; Dakota Howard; Darlene Wagner; Dave Wentworth; Dhwani Batra; Jasmine Padilla; Justin Lee; Katie Dillon; Krista Queen; Kristen Knipe; Kristine Lacek; Mark Burroughs; Matthew Schmerer; Mili Sheth; Peter Cook; Sam Shepard; Sarah Nobles; Shoshona Le; Suxiang Tong; Vivien Dugan; Yvette Unoarumhi
EPI_ISL_1708562	Gundersen Clinical Microbiology Laboratory	Kabara Cancer Research Institute	Craig S. Richmond; Paraic A. Kenny
EPI_ISL_547703, EPI_ISL_661031	Gundersen Molecular Diagnostics Laboratory	Kabara Cancer Research Institute	Craig S. Richmond; Paraic A. Kenny
EPI_ISL_2694956	H Fernando Fonseca	Instituto Nacional de Saude (INSA)	Borges et al
EPI_ISL_733351, EPI_ISL_872972, EPI_ISL_872983, EPI_ISL_1652566, EPI_ISL_1664679, EPI_ISL_1919516, EPI_ISL_1919546, EPI_ISL_2385226, EPI_ISL_2385230, EPI_ISL_2385261, EPI_ISL_2385266, EPI_ISL_2385280, EPI_ISL_2385308, EPI_ISL_2450590			
see above	HELIX LLC	WHO National Influenza Centre Russian Federation	Andrey Komissarov; Anna Ivanova; Artem Fadeev; Daria Danilenko; Dmitry Bazhenov; Dmitry Lioznov; Elena Nabieva; Georgii Bazykin; Kirill Varchenko; Ksenia Safina; Kseniya Komissarova; Maria Pisareva; Maria Timofeeva; Mikhail Bakaev; Nikita Yolshin; Oula Mansour; Oula Masour; Tamila Musaeva; Veronika Eder
EPI_ISL_2452134	HI Dept. of Health, State Laboratories Division	Centers for Disease Control and Prevention Division of Viral Diseases, Pathogen Discovery	Alison Laufer Halpin; Ben L. Rambo-Martin; Clinton R. Paden; Dakota Howard; Darlene Wagner; Dave Wentworth; Dhwani Batra; Jasmine Padilla; Justin Lee; Katie Dillon; Krista Queen; Kristen Knipe; Kristine Lacek; Mark Burroughs; Matthew Schmerer; Mili Sheth; Peter Cook; Sam Shepard; Sarah Nobles; Shoshona Le; Suxiang Tong; Vivien Dugan; Yvette Unoarumhi
EPI_ISL_1336649	HOPITAL PRINCESSE GRACE	CNR Virus des Infections Respiratoires - France SUD	Antonin Bal; Bruno Lina; Gregory Destras; Gwendolyne Burfin; Hadrien Regue; Laurence Josset; Martine Valette; Quentin Semanas
EPI_ISL_2331774	HOSPITAL GENERAL VIRGEN DE LA LUZ	Instituto de Salud Carlos III	A. Monzón; F. Casas; I. Jiménez; I.RODRIGUEZ ESCUDERO; Iglesias-Caballero; M. Sandonís; MARIA JOSE; P. Zaballos; S. Camarero; S. Cuesta; S. Pozo; S. Varona; V. Vázquez-Morón
EPI_ISL_2020128	HOSPITAL MATEU ORFILA	HOSPITAL UNIVERSITARIO SON ESPASES	Antonio Oliver; Carla López-Causapé; Pablo Fraile-Ribot; SeqCovid
EPI_ISL_636977	HP Pemba	KRISP, KZN Research Innovation and Sequencing Platform	Giandhari J; Ismael N; Nadia Sitoe; Nedio Mabunda; Paulo Arnaldo; Pillay S; Tegally H; Wilkinson E; de Oliveira T
EPI_ISL_2738567	Hackensack Medical Center	New York Genome Center	Andre Corvelo; Barry Kreiswirth; David Perlin; Dayna M. Oschwald; Jose Mediavilla; Kaelea Composto; Kar Chow; Liang Chen; Marcus Cunningham; Michael Zody; Samantha Fennessey; Tom Maniatis
EPI_ISL_1089048	Helix / Illumina	Respiratory Viruses Branch, Division of Viral Diseases, Centers for Disease Control and Prevention	Alexandre Bolze; Ary Ascencio; Ben L. Rambo-Martin; Brad Sickler; Charlotte Rivera-Garcia; Christine Tran; Clinton R. Paden; Dakota Howard; David Becker; Dhwani Batra; Duncan MacCannell; Efren Sandoval; Eileen de Feo; Elizabeth Cirulli; Eric Allen; Geraint Levan; James Lu; Jan Antico; Jason Nguyen; Jimmy Ramirez; Jingtao Liu; Kelly Schiabor Barrett; Kim Gietzen; Magnus Isaksson; Marc Laurent; Matthew Tolentino; Nicole L. Washington; Peter W. Cook; Phil Febbo; Ryan Cho; Shannon Wickline; Sherry Wang; Simon White; Summer Galloway; Suxiang Tong; Tyler Cassens; William Lee
EPI_ISL_1262036, EPI_ISL_1391995, EPI_ISL_1420974, EPI_ISL_1460961, EPI_ISL_1512099, EPI_ISL_1554417, EPI_ISL_1576106, EPI_ISL_1581075, EPI_ISL_1592421, EPI_ISL_1592485, EPI_ISL_1615349, EPI_ISL_1615384, EPI_ISL_1679967, EPI_ISL_1680048, EPI_ISL_1690467, EPI_ISL_1735500, EPI_ISL_1735545, EPI_ISL_1797026, EPI_ISL_1797206, EPI_ISL_1803813, EPI_ISL_1803921, EPI_ISL_1804135, EPI_ISL_1804282, EPI_ISL_1804494, EPI_ISL_1907338, EPI_ISL_1907632, EPI_ISL_1907995, EPI_ISL_1923331, EPI_ISL_1923362, EPI_ISL_1923401, EPI_ISL_1923785, EPI_ISL_1923911, EPI_ISL_1923955, EPI_ISL_1924007, EPI_ISL_1924031, EPI_ISL_1942165, EPI_ISL_1990614, EPI_ISL_1990723, EPI_ISL_1990741, EPI_ISL_1990856, EPI_ISL_1990910, EPI_ISL_1990921, EPI_ISL_1991008, EPI_ISL_1991368, EPI_ISL_1991454, EPI_ISL_1991572, EPI_ISL_1991775, EPI_ISL_2009735, EPI_ISL_2010071, EPI_ISL_2010188, EPI_ISL_2010380, EPI_ISL_2010430, EPI_ISL_2010478, EPI_ISL_2010812, EPI_ISL_2010872, EPI_ISL_2097653, EPI_ISL_2097683, EPI_ISL_2097858, EPI_ISL_2098013, EPI_ISL_2144255, EPI_ISL_2144340, EPI_ISL_2144346, EPI_ISL_2144363, EPI_ISL_2144594, EPI_ISL_2145008, EPI_ISL_2145113, EPI_ISL_2145142, EPI_ISL_2145272, EPI_ISL_2145592, EPI_ISL_2145734, EPI_ISL_2159577, EPI_ISL_2159811, EPI_ISL_2159870, EPI_ISL_2160115, EPI_ISL_2187341, EPI_ISL_2248098, EPI_ISL_2248220, EPI_ISL_2269629, EPI_ISL_2269932, EPI_ISL_2269968, EPI_ISL_2320077			
see above	Helix/Illumina	Centers for Disease Control and Prevention Division of Viral Diseases, Pathogen Discovery	Adrian Paskey; Alexandre Bolze; Ary Ascencio; Ben L. Rambo-Martin; Benjamin Rambo-Martin; Brad Sickler; Charlotte Rivera-Garcia; Christine Tran; Christopher Gulvick; Clinton R. Paden; Dakota Howard; Darlene Wagner; David Becker; Dhwani Batra; Duncan MacCannell; Efren Sandoval; Eileen de Feo; Elizabeth Cirulli; Eric Allen; Geraint Levan; James Lu; Jan Antico; Jason Caravas; Jason Nguyen; Jimmy Ramirez; Jingtao Liu; Kara Moser; Kelly Schiabor Barrett; Kim Gietzen; Magnus Isaksson; Marc Laurent; Matthew Schmerer; Matthew Tolentino; Nicole L. Washington; Peter W. Cook; Phil Febbo; Ryan Cho; Scott Sammons; Shannon Wickline; Shatavia Morrison; Sherry Wang; Simon White; Summer Galloway; Suxiang Tong; Tyler Cassens; William Lee; Yvette Unoarumhi
EPI_ISL_850626	Helix/Illumina	Genomics and Discovery, Respiratory Viruses Branch, Division of Viral Diseases, Centers for Disease Control and Prevention	Alexandre Bolze; Ary Ascencio; Ben L. Rambo-Martin Eileen de Feo; Brad Sickler; Charlotte Rivera-Garcia; Christine Tran; Clinton R. Paden; David Becker; Dhwani Batra; Duncan MacCannell; Efren Sandoval; Elizabeth Cirulli; Eric Allen; Geraint Levan; James Lu; Jan Antico; Jason Nguyen; Jimmy Ramirez; Jingtao Liu; Kelly Schiabor Barrett; Kim Gietzen; Magnus Isaksson; Marc Laurent; Matthew Tolentino; Nicole L. Washington; Peter W. Cook; Phil Febbo; Ryan Cho; Shannon Wickline; Sherry Wang; Simon White; Summer Galloway; Suxiang Tong; Tyler Cassens; William Lee
EPI_ISL_978735	Helix/Illumina	Respiratory Viruses Branch, Division of Viral Diseases, Centers for Disease Control and Prevention	; Alexandre Bolze; Ary Ascencio; Ben L. Rambo-Martin; Brad Sickler; Charlotte Rivera-Garcia; Christine Tran; Clinton R. Paden; Dakota Howard; David Becker; Dhwani Batra; Duncan MacCannell; Efren Sandoval; Eileen de Feo; Elizabeth Cirulli; Eric Allen; Geraint Levan; James Lu; Jan Antico; Jason Nguyen; Jimmy Ramirez; Jingtao Liu; Kelly Schiabor Barrett; Kim Gietzen; Magnus Isaksson; Marc Laurent; Matthew Tolentino; Nicole L. Washington; Peter W. Cook; Phil Febbo; Ryan Cho; Shannon Wickline; Sherry Wang; Simon White; Summer Galloway; Suxiang Tong; Tyler Cassens; William Lee
EPI_ISL_906752	Hematology Laboratory, Section of Molecular Diagnostics, University Clinical Centre, Medical University of Gdansk	Laboratory of Recombinant Vaccines	Adam Sodol; Aneta Szulc; Boguslaw Szewczyk; Ewa Milosz; Krystyna Bienkowska-Szewczyk; Krzysztof Lewandowski; Lukasz Rabalski; Maciej Grzybek; Maciej Kosinski; Marlena Robakowska
EPI_ISL_1201507	Hopital	National Reference Center for Viruses of Respiratory Infections, Institut Pasteur, Paris	Angela Brisebarre; Camille Capel; Durivault JéRôMe; Etienne Simon-Lorière; Marion Barbet; Maud Vanpeene; Méline Bizard; Sylvie Behillil; Sylvie van der Werf; Vincent Enouf
EPI_ISL_2484022, EPI_ISL_2601724	Hospital General Universitario Gregorio Marañón	Hospital General Universitario Gregorio Marañón	Cristina Rodriguez-Grande; Darío García de Viedma; Julia Suárez; Laura Pérez-Lago; Marta Herranz Martin; Patricia Muñoz; Pedro Sola Campoy; Pilar Catalán; Sergio Buenestado Serrano; Victor Manuel de la Cueva
EPI_ISL_1196770	Hospital Melaka	Institute for Medical Research, Infectious Disease Research Centre, National Institutes of Health, Ministry of Health Malaysia	Kamel K; Mohd Zawawi Z; Ramly N; Robert F; Suppiah J; Thayan R

EPI_ISL_1482631, EPI_ISL_1491363	Hospital Sharp	Microbial Genomics Laboratory	Alejandra Garcia-Gasca; Bruno Gomez-Gil; Daniel Fregoso-Rueda; Julissa Enciso-Ibarra
EPI_ISL_455351	Hospital Txagorritxu	Instituto de Salud Carlos III	A. Monzón; C. Gómez; F. Casas; I; I. Jiménez; Iglesias-Caballero; M. Camarero; M. Cuesta; M. González-Esguevillas; M. Molinero Calamita; M. Zaballos; P. Jiménez; S. Juliá; S. Pozo; S. Varona
EPI_ISL_2524770	Hospital Universitari Arnau de Vilanova	Hospital Universitari Vall d'Hebron - Vall d'Hebron Institut de Recerca	Alejandra González-Sánchez; Andrés Antón; Ariadna Rando; Carla Castillo; Cristina Andrés; Damir Garcia-Cehic; Josep Quer; Juliana Esperalba; Maria Carmen Martin; Maria Gema Codina; Maria Piñana; Tomàs Pumarola
EPI_ISL_2352009, EPI_ISL_2352011, EPI_ISL_2516487, EPI_ISL_2637349	Hospital Universitari Bellvitge	Microbiology Department	Aida Gonzalez-Diaz; Carmen Ardanuy; Jordi Camara; Jordi Niubó; Laura Calatayud; M Angeles Domínguez; Miguel Fernandez-Huerta; Sara Marti
EPI_ISL_1582891	Hospital Universitari Vall d'Hebron - Vall Hebron Institut de Recerca	Hospital Universitari Vall d'Hebron - Vall Hebron Institut de Recerca	Andrés Antón; Ariadna Rando; Carla Castillo; Cristina Andrés; Damir Garcia-Cehic; Josep F Abril; Josep Quer; Juliana Esperalba; Maria Carmen Martin; Maria Gema Codina; Maria Piñana; Tomàs Pumarola
EPI_ISL_1908856, EPI_ISL_2047772, EPI_ISL_2047773, EPI_ISL_2284923, EPI_ISL_2493132	Hospital Universitari Vall d'Hebron - Vall d'Hebron Institut de Recerca	Hospital Universitari Vall d'Hebron - Vall d'Hebron Institut de Recerca	Alejandra González-Sánchez; Andrés Antón; Ariadna Rando; Carla Castillo; Cristina Andrés; Damir Garcia-Cehic; Josep Quer; Juliana Esperalba; Maria Carmen Martin; Maria Gema Codina; Maria Piñana; Tomàs Pumarola
EPI_ISL_2271215	Hospital Universitario Clínico San Cecilio	SeqCOVID-SPAIN consortium/IBV(CSIC)	Adolfo de Salazar; Federico García; Laura Viñuela; Natalia Chueca and SeqCOVID-SPAIN consortium
EPI_ISL_2091014	Hospital Universitario Cruces	Biocruces Bizkaia	Ana Belen de la Hoz; Mikel Gallego
EPI_ISL_582109, EPI_ISL_1671520	Hospital Universitario Marqués de Valdecilla - IDIVAL (Santander, Cantabria)	SeqCOVID-SPAIN consortium/IBV(CSIC)	Daniel Pablo Marcos; Jesús Rodríguez Rodríguez; Jose Manuel Méndez Legaza; María Eliecer Cano García; María Siller Ruiz and SeqCOVID-SPAIN consortium; Mónica Gozalo Margüello
EPI_ISL_693535	Hospital Vila Franca de Xira	Instituto Nacional de Saude (INSA)	Borges et al
EPI_ISL_1490405	Hospital of Southern Norway - Kristiansand, Department of Medical Microbiology	Norwegian Institute of Public Health, Department of Virology	Atiya R Ali; Debech Nadia; Engebretsen Serina Beate; Garcia Llorente Ignacio; Hilde Elshaug; Hilde Vollan; Jon Bråte; Kamilla Heddeland Instefjord; Karoline Bragstad; Kathrine Stene-Johansen; Marie Paulsen Madsen; Olav Hungnes; Pedersen Benedikte Nevjen; Rasmus Riis Kopperud
EPI_ISL_784021, EPI_ISL_1237692, EPI_ISL_2225235, EPI_ISL_2225238	Houston Methodist Hospital	Houston Methodist Hospital	David W. Bernard; Heather Hendrickson; Ilya J. Finkelstein; James J. Davis; Jessica Cambric; Jimmy Gollihar; Kristina Reppond; Layne Pruitt; Madison N. Shyer; Marcus Nguyen; Matthew Ojeda Saavedra; Maulik Shukla; Paul A. Christensen; Prasanti Yerramilli; Randall J. Olsen; Robert Olson; Ryan Gadd; S. Wesley Long; Sishir Subedi; and James M. Musser
EPI_ISL_645059	Human Genome Variation Research Group, Malopolska Centre of Biotechnology	Human Genome Variation Research Group, Malopolska Centre of Biotechnology	Botwina, P.; Branicki, W.; Dabrowska, A.; Foremny, J.; Gromowski, T.; Klajmon, A.; Kopera, K.; Kowalski, M.; Labaj; Marszalek, K.; Owczarek, K.; P.P.; Pisarek, A.; Pospiech, E.; Pyrc, K.; Sanak, M.; Swadzba, J.; Szczepanski, A.
EPI_ISL_1272929	IA State Hygienic Laboratory	Centers for Disease Control and Prevention Division of Viral Diseases, Pathogen Discovery	Anna Montmayeur; Anna Uehara; Ben L. Rambo-Martin; Clinton R. Paden; Dhwani Batra; Haibin Wang; Jasmine Padilla; Jing Zhang; Justin Lee; Katie Dillon; Krista Queen; Kristen Knipe; Kristine Lacek; Lori Rowe; Mark Burroughs; Matthew Schmerer; Mili Sheth; Peter W. Cook; Rachel Marine; Sam Shepard; Sarah Nobles; Shoshona Le; Suxiang Tong; Yan Li; Ying Tao
EPI_ISL_2234879, EPI_ISL_2234883, EPI_ISL_2234900, EPI_ISL_2234903, EPI_ISL_2444780, EPI_ISL_2444783, EPI_ISL_2444788, EPI_ISL_2444791, EPI_ISL_2444793, EPI_ISL_2444794, EPI_ISL_2444799, EPI_ISL_2444801, EPI_ISL_2444803, EPI_ISL_2444807, EPI_ISL_2444812, EPI_ISL_2444814, EPI_ISL_2444817, EPI_ISL_2444818, EPI_ISL_2444819, EPI_ISL_2444820, EPI_ISL_2444821, EPI_ISL_2444822, EPI_ISL_2444825, EPI_ISL_2444827, EPI_ISL_2444835, EPI_ISL_2444836, EPI_ISL_2444839			
see above	IICS-UNA	IICS-UNA	Adriana Valenzuela; Alejandra Rojas; Chyntia Diaz; Eva Nara; Fatima Cardozo; Florencia del Puerto; Joel Ortiz; Jonas Fernandez; Laura Franco; Laura Mendoza; Leticia Rojas; Magaly Martinez; Maria Eugenia Galeano.
EPI_ISL_1626577, EPI_ISL_1994891, EPI_ISL_1994892, EPI_ISL_2134586, EPI_ISL_2134606, EPI_ISL_2134610, EPI_ISL_2242591, EPI_ISL_2557220, EPI_ISL_2688209			
see above	IN State Department of Health Laboratory Services	IN State Department of Health Laboratory Services	Ankita Kashikar; Brian Pope; Cassandra Campion; Jamie Yeadon; Kyle Brownlee; Lixia Liu; Mark Glazier; Melissa Hindenlang
EPI_ISL_1577798, EPI_ISL_1577817	INHRR	Laboratorio de Virología Molecular	Aguilar M; Alarcon V; D Angelo P; Delgado M; Garzaro D; Jaspe RC; Loureiro CL; Pujol FH; Rangel HR; Rodriguez L; Zambrano JL
EPI_ISL_2484167	INSP-Bamako	Malaria Research and Training Center	Abdoulaye Djimde; Amadou Daou; Antoine Dara; Demba Koita; Ibrehima Guindo
EPI_ISL_1630259	IRESSEF	Abbott	Adbou Padane; Ambroise Ahouidi; Aminata Dia; Aminata Mboup; Ana Olivo; Anna julienne selbe Ndiaye; Barbara Harris; Cyrille Diedhiou; Gavin Cloherty; Mary Rodgers; Moustapha Mbow; Nafissatou Leye; Ndeye Diabou Diagne; Papa Alassane Diaw; Souleymane Mboup; Todd Meyer
EPI_ISL_1661919, EPI_ISL_1663581	IU-Cerrahpasa, Cerrahpasa School of Medicine, COVID-19 Lab	IU-Cerrahpasa, Cerrahpasa School of Medicine, COVID-19 Lab	Kenan Midilli; Mert Kuskucu; Yesim Tuyji Tok
EPI_ISL_1806250	Idaho Bureau of Laboratories	Center for Global Health, University of New Mexico Health Sciences Center	Christopher Ball; Darrell Dinwiddie; Daryl Domman; Kurt Schwalm; Matthew Burns; Robert Voermans; Valerie Morley
EPI_ISL_1363182, EPI_ISL_2031610	Idaho Bureau of Laboratories	Idaho Bureau of Laboratories	"R. Beukelman; Aimee Ceniseros; Christopher Ball; Christopher Ball"; Matthew Charles Burns; R. Beukelman; Robert L. Voermans
EPI_ISL_1742405, EPI_ISL_1809681, EPI_ISL_2140020, EPI_ISL_2140036, EPI_ISL_2300529	Illinois Department of Public Health	Illinois Department of Public Health - Chicago Lab	Ira Heimler; Joel Price; Vineet K. Dhiman
EPI_ISL_1443242, EPI_ISL_2135048	Illinois Department of Public Health - Springfield Lab	Illinois Department of Public Health - Springfield Lab	Bryan Sim; Gordon McCall
EPI_ISL_2001062, EPI_ISL_2151339, EPI_ISL_2151340, EPI_ISL_2151341, EPI_ISL_2151342, EPI_ISL_2151343, EPI_ISL_2151344			
see above	Immunology, Noguchi Memorial Institute for Medical Research	Immunology, Noguchi Memorial Institute for Medical Research	A.W.; Adu, B.; Agbodzi, B.; Appiah-Kubi, J.; Asare; Assane, H.; Awunyo, S.; Bonney; Dagnran, A.; Dorkenoo, A.; Egyir, B.; Halatoko; Issa, Z.; J.K.; K.J.; K.M.; Kossi, K.; Kumordjie, S.; Lamboni, L.; Layibo, Y.; Maman, I.; Mohktar, Q.; Sadji; Salah, D.; Salou, M.; Y.A.
EPI_ISL_2533903	Imágenes Diagnósticas de Mazatlán	Microbial Genomics Laboratory	; Alejandra García-Gasca; Alejandra Hernández-Terán; Alejandro Sánchez-Flores; Alfredo Herrera-Estrella; Alicia Ocaña-Mondragón; Andreu Comas-García; Angel Gustavo Salas-Lais; Antonio Loza Román; Bernardo Martínez-Miguel; Blanca Taboada; Brenda Irasema Maldonado-Meza; Bruno Gómez-Gil; Carla Ivón Herrera-Najera; Carlos F. Arias; Celia Boukadida; Clara Esperanza Santacruz-Tinoco; Concepción Grajales-Muñiz; Consorcio Mexicano de Vigilancia Genómica (CoViGen-Mex). Authors (in alphabetical order): Julio Elias Alvarado-Yaah; Cristóbal Cháidez-Quiróz; Célida Duque Molina; Célida Martínez- Rodríguez; Daniel Fregoso-Rueda; Daniel Lira Morales; Eduardo Becerril-Vargas; Fernando Fontove-Herrera; Fidencio Mejía-Nepomuceno; Francisco Pulido; Gloria Elena Espinosa-Ayala; Gloria María Molina-Salinas; Gloria Vazquez; Hector Esteban Paz-Juárez; Hector Montoya-Fuentes; Helen Haydee Fernanda Ramírez-Plascencia; Irvin González-López; Jean Pierre González; Jesús Hernández; Joel Armando Vázquez-Pérez.; Jorge Salas-Hernández; José Antonio Enciso-Moreno; José Arturo Martínez-Orozco; José Esteban Muñoz-Medina; José de Jesús Nuñez-Contreras; Juan Bautista Chale-Dzul; Julissa Enciso-Ibarra; Luis Alberto Ochoa-Carrera; Margarita Matías-Florentino; Mario Mújica-Sánchez; Marissa Perez-Garcia; María Guadalupe Santiago-Mauricio; María Guadalupe de Jesús Mireles-Rivera; Nelly Sélem-Mojica; Pavel Isa; Ricardo Ciria Merce; Ricardo Grande; Rosa María Gutiérrez Rios; Santiago Ávila-Ríos; Selene Zárate; Susana Lopez; Verónica Mata-Haro; Víctor Eduardo García-Arias; Víctor Hugo Borja-Aburto
EPI_ISL_1628597, EPI_ISL_1628633, EPI_ISL_1628655	InDRE	Instituto Nacional de Medicina Genomica	Alcaraz N; Canseco Mendez JC; Cedro-Tanda A; Garcia-Cardenas FJ; Gisela Barrera-Badillo; Gonzalez-Barrera D; Gonzalez-Woge MA; Herrera-Montalvo LA.; Hidalgo-Miranda A; Irma Lopez-Martinez; Jose Ernesto Ramirez González; Mendoza-Vargas A; Miranda-Ortiz H; Munguia-Garza P; Ramirez-Vega O; Rangel-DeLeon D; Reyes-Grajeda JP; Rosas-Escobar P
EPI_ISL_476702	Incubadora Venezolana de Ciencia, Venezuela	Incubadora Venezolana de Ciencia, Venezuela / Instituto Nacional de Salud, Bogotá, Colombia / Grupo de Investigaciones Microbiológicas-UR (GIMUR), Departamento de Biología, Facultad de Ciencias Naturales, Universidad del Rosario, Bogotá, Colombia / Icahn School of Medicine at Mount Sinai, New York, USA	Alberto Paniz-Mondolfi; Ana S. Gonzalez-Reiche; Angelica Rico; Aníbal A. Teherán; Carolina Florez; Carolina Hernández; Emilia Mia Sordillo; Esther C. Barros; Harm van Bakel; Jesús E. Jaimes; Juan David Ramírez; Lisseth Pardo; Lourdes Delgado; Luis Perez-Garcia; Marina Muñoz; Matthew M. Hernandez; Sergio Gomez; Viviana Simon
EPI_ISL_2304357, EPI_ISL_2304405, EPI_ISL_2304415, EPI_ISL_2304444	Indiana Animal Disease Diagnostic Laboratory	Carpi Laboratory - Purdue University	Abebe A Fola; G Kenitra Hendrix; Giovanna Carpi; Ilinca I Ciubotariu; Jack Dorman; Jobin J Kattoor; Lev Gorenstein; Nicole M Perry; Rebecca P Wilkes
EPI_ISL_1525013	Indiana State Department of Health	Quantigen Biosciences	Paul J Childress
EPI_ISL_884410	Infectious Diseases, Quest Diagnostics	Infectious Diseases, Quest Diagnostics	Anderson, B.; Bernstein; D.F.; Gerasimova, A.; Hua, M.; K.E.; Kagan; L.E.; Lacbawan, F.; Liu, Y.; Livingston; Owen, R.; R.M.; Rosenthal; S.H.; Shalhout
EPI_ISL_2107507, EPI_ISL_2151336, EPI_ISL_2151337	Infectious Diseases, King Faisal Hospital Research Center	Infectious Diseases, King Faisal Hospital Research Center	Al-Qahtani A, A.; Alahideb B, M.; Alhamlan F, S.; Almaghrabi R, S.; Alsanea M; Alsanea M, S.; Althawadi S, I.; Balavenkatesh Mani; Balavenkatesh Mani, M.; M. and UdayaRaja G, K.; Mutabagani M, S.; S. and Alahideb B, M.; UdayaRaja G, K.
EPI_ISL_1587647, EPI_ISL_1803140, EPI_ISL_2013703, EPI_ISL_2087219, EPI_ISL_2087232, EPI_ISL_2087519, EPI_ISL_2087590, EPI_ISL_2087703, EPI_ISL_2087762, EPI_ISL_2088485, EPI_ISL_2089042, EPI_ISL_2089192, EPI_ISL_2089221, EPI_ISL_2089293, EPI_ISL_2186510, EPI_ISL_2186522, EPI_ISL_2186947, EPI_ISL_2187138			
see above	Infinity Biologix	Centers for Disease Control and Prevention Division of Viral Diseases, Pathogen Discovery	Adrian Paskey; Benjamin Rambo-Martin; Chirayu Goswami; Christian Bixby; Christopher Gulvick; Clinton R. Paden; Dakota Howard; Darlene Wagner; Dhwani Batra; Duncan MacCannell; Jason Caravas; Jonathan Schultz; Kara Moser; Matthew Schmerer; Peter W. Cook; Robin Grimwood; Russ Hager; Scott Sammons; Shatavia Morrison; Yihe Wang; Yvette Unoarumhi
EPI_ISL_1908147, EPI_ISL_1908155, EPI_ISL_1909244, EPI_ISL_1909250, EPI_ISL_1909253, EPI_ISL_1912936, EPI_ISL_1913014, EPI_ISL_1913051, EPI_ISL_1913059, EPI_ISL_1913077			
see above	Institut National d'Hygiène	Laboratoire de Biotechnologie	Abdelomunim Essabbar; Fatima El Falaki; Fatima ElFalaki; Hicham Oumzil; Lahcen Belyamani and Azeddine Ibrahimi; Mohamde Rhajaoui; Mohamed Rhajaoui; Mouna Ouadghiri; Saaïd Amzazi; Tarik Aanniz
EPI_ISL_1443003	Institut National d'hygiène	"Unité Mixte Internationale TransVIHMI (UMI 233 IRD – U1175 INSERM - Université de Montpellier) IRD (Institut de recherche pour le développement)"	Abla A. KONOU; Adodo SADJI; Ahidjo AYOUBA; Akoélé SILIADIN; Alassane OURO-MEDELI; Amivi EHLAN; Améyo DORKENOO; Anoumou DAGNRA; Christelle BUTEL; Déléma MABA; Eric DELAPORTE; Issaka Maman; Kokou TEGUENI; Laetitia SERRANO; Martine PEETERS; Messanh DOUFFAN; Mireille PRINCE-DAVID; Mounerou SALOU; Sidonie A.M.KAGNISSODE; Sika DOSSIM; Wembo A. HALATOKO
EPI_ISL_2278010	Institut National d'Hygiène (INH)	Unité Mixte Internationale TransVIHMI (UMI 233 IRD – U1175 INSERM - Université de Montpellier)IRD (Institut de recherche pour le développement)	Abla A. KONOU; Adodo SADJI; Ahidjo AYOUBA; Akoélé SILIADIN; Alassane OURO-MEDELI; Amivi EHLAN; Améyo DORKENOO; Anoumou DAGNRA; Christelle BUTEL; Déléma MABA; Eric DELAPORTE; Issaka Maman; Kokou TEGUENI; Laetitia SERRANO; Martine PEETERS; Messanh DOUFFAN; Mireille PRINCE-DAVID; Mounerou SALOU; Sidonie A.M.KAGNISSODE; Sika DOSSIM; Wembo A. HALATOKO

EPI_ISL_418206, EPI_ISL_418213, EPI_ISL_480783, EPI_ISL_485713	Institut Pasteur Dakar	Institut Pasteur de Dakar	Amadou Alpha Sall; Amadou Alpha Sall.; Mamadou Diop; Mamadou Malado Jallow; Mamadou diop; Marie Henriette Dior Ndione; Moussa Moise Diagne; Ndongo Dia; Ousmane Faye; Safietou Sanke
EPI_ISL_2110643	Institut Pasteur du Maroc	Functional Genomics platform/CNRST	Abdelkrim Meziane Bellefquih; Alaoui Sanaa-amine; Chouati Taha; EL FAHIME Elmostafa; Elannaz Hicham; Ennibi Khalid; Hemlali Mouhsine; MERABET MOUAD; Maaroufi Abderrahmane; Melloul Marouane; NOURLIL Jalal; Touil Nadia; Youbi Mohammed
EPI_ISL_955165	Institute for Biocides and Medical Ecology	Institute of microbiology and Immunology, Faculty of Medicine, University of Belgrade	Jankovic, M.; Jovanovic, T.; Knezevic, A.; Milicevic, O.; Sekler, M.; Tesovic, B.; Vidanovic, D.
EPI_ISL_1827888	Institute for Health Research, Epidemiological Surveillance and Training (IRESSEF)	Abbott Laboratories	Adbou Padane; Ambroise Ahouidi; Aminata Dia; Aminata Mboup; Ana Olivo; Anna julienne selbe Ndiaye; Barbara Harris; Cyrille Diedhiou; Gavin Cloherty; Mary Rodgers; Moustapha Mbow; Nafissatou Leye; Ndeye Diabou Diagne; Papa Alassane Diaw; Souleymane Mboup; Todd Meyer
EPI_ISL_1760647, EPI_ISL_1916510	Institute for Infectious Diseases, University of Bern, Switzerland	Institute for Infectious Diseases, University of Bern, Switzerland	Alban Ramette; Christian Baumann; Cora Sägesser; Franziska Suter-Riniker; Miguel A Terrazos Miani; Pascal Bittel; Peter Keller; Stefan Neuenschwander; Stephen L Leib
EPI_ISL_912397, EPI_ISL_1406146, EPI_ISL_1406294	Institute for Medical Research, Infectious Disease Research Centre, National Institutes of Health, Ministry of Health Malaysia	Institute for Medical Research, Infectious Disease Research Centre, National Institutes of Health, Ministry of Health Malaysia	Azizan MA; Kamel K; Mohd Zawawi Z; Ramly N; Robert F; Suppiah J; Thayan R
EPI_ISL_707707, EPI_ISL_2348811	Institute for Urban Disease Control and Prevention	COVID-19 Network Investigations (CONI) Alliance	Amornmas Kongklieng; Anek Mungaomklang; Angkana Huang; Anthony R. Jones; Arporn Wangwiwatsin; Bhakbhoom Panthan; Chonticha Klungtong; Duangkamon Loesbanluechai; Ekawat Pasomsub; Elizabeth Batty; Insee Sensorn; Janjira Thaipadungpanit; Kamolthip Atsawawaranunt; Khajohn Joonlasak; Khajohn Joonsalak; Kingkan Rakmanee; Krittikorn Kumpornsin; Namfon Kotanan; Prayuth Kaewmalang; Pukkaporn Parnwijitkul; Stefan Fernandez; Thanat Chookajorn; Theerarat Kochakarn; Treewat Watthanachockchai; Vichan Pawun; Wasun Chantratita; Wudtichai Manasatienkij
EPI_ISL_1209341, EPI_ISL_1209345, EPI_ISL_1583415	Institute for Water Quality and Resource Management, Technical University Vienna	Bergthaler laboratory, CeMM Research Center for Molecular Medicine of the Austrian Academy of Sciences	Andreas Bergthaler; Anna Schedl; Bekir Erguner; Benedikt Agerer; Christoph Bock; Fabian Amman; Jan Laine; Lukas Endler; Maelle Le Moing; Martin Senekowitsch; Michael Schuster; Petr Triska; Thomas Penz
EPI_ISL_918411	Institute of Biocides and Medical Ecology	Virology Department Institute of Microbiology and Immunology Faculty of Medicine University of Belgrade	Abazovic Dzihan; Banko Ana; Despot Dragana; Loncar Ana; Milicevic Ognjen; Miljanovic Danijela
EPI_ISL_1118849, EPI_ISL_1265328, EPI_ISL_1935543, EPI_ISL_1964279, EPI_ISL_1964351, EPI_ISL_2030196, EPI_ISL_2321813, EPI_ISL_2322223, EPI_ISL_2322290, EPI_ISL_2491984, EPI_ISL_2492325			
see above	Institute of Microbiology and Immunology, Faculty of Medicine, University of Ljubljana	Institute of Microbiology and Immunology, Faculty of Medicine, University of Ljubljana	Alen Suljič; Andraž Celar; Andrej Celar; Dominika Šturm; Doroteja Vlaj; Mario Poljak; Matic Brvar; Miša Korva; Patricija Pozvek; Samo Zakotnik; Tatjana Avšič – Županc; Tomaž Mark Zorec; Špela Pleh
EPI_ISL_548946	Institute of Microbiology, University of Veterinary and Animal sciences	Institute of Microbiology, University of Veterinary and Animal sciences	Ali; Altaf, I.; Anwar, M.; Ashraf; Asif, A.; Attique; Awan; Aziz; Bhatti; Cheema; Fazal, S.; Hassan, S.; Khan; Khan, N.; M.A.; M.B.; M.M.; M.S.; M.T.; M.U.; M.W.; M.Z.; Mehmood, A.; Mukhtar, N.; N.A.; Nawaz, M.; Rafique, S.; Rana; Raza, S.; S.Q.; S.Z.; Sardar, N.; Sarwar, H.; Shabbir; Shah; Tahir, Z.; Yaqub, T.; Younis
EPI_ISL_583481, EPI_ISL_1234442, EPI_ISL_1239413, EPI_ISL_1547902, EPI_ISL_2444294	Institute of Virology, Biomedical Research Center of the Slovak Academy of Sciences, Bratislava	Faculty of Natural Sciences, Comenius University, Bratislava	Boris Klempa; Brona Brejova; Broňa Brejová; Dominika Fričová; Edita Staroňová; Elena Tichá; Jozef Nosek; Juraj Kopacek; Juraj Kopáček; Kristina Borsova; Kristína Boršová; Lubomira Lukacikova; Martina Lickova; Martina Ličková; Martina Nebohacova; Martina Neboháčová; Monika Slavikova; Monika Sláviková; Sabina Fumacova Havlikova; Sabina Fumačová Havlíková; Tomas Vinar; Tomáš Vinař; Viktoria Cabanova; Viktoria Hodorova; Viktória Hodorová; Viktória Čabanová; Ľubomíra Lukáčiková
EPI_ISL_1654825, EPI_ISL_1654830, EPI_ISL_1654831, EPI_ISL_1654832, EPI_ISL_1654833, EPI_ISL_1654836, EPI_ISL_1654838, EPI_ISL_1654839, EPI_ISL_1654840			
see above	Institute of Virology, Vaccines and Sera "Torlak"	Institute of microbiology and Immunology, Faculty of Medicine, University of Belgrade	Jankovic, M.; Jovanovic, T.; Knezevic, A.; Milicevic, O.; Sekler, M.; Tesovic, B.; Vidanovic, D.
EPI_ISL_837728	Instituto Nacional de Enfermedades Respiratorias (INER)	Instituto Nacional de Enfermedades Respiratorias (INER)	Alejandra Hernández-Terán; Alma Rincón-Rubio; Celia Boukadida; Edgar Sevilla-Reyes; Eduardo Becerril-Vargas; Fidencio Mejía-Nepomuceno; Hector Esteban Paz-Juárez; Joel Armando Vázquez-Pérez; Jorge Salas-Hernández; José Arturo Martínez-Orozco; Margarita Matías-Florentino; Mario Mújica-Sánchez; Olivia Briceño; Santiago Ávila-Ríos
EPI_ISL_1824457	Instituto Nacional de Medicina Genomica	Centro de Investigación en Enfermedades Infecciosas (CIENI), Instituto Nacional de Enfermedades Respiratorias (INER)	Arriaga-Canon C; Avila-Rios S; Boukadida C; Cedro-Tanda A; Herrera-Montalvo LA; Hidalgo-Miranda A; Matias-Florentino M; Mendoza-Vargas A; Perez-Garcia M; Reyes-Grajeda JP; Reyes-Teran G
EPI_ISL_933522, EPI_ISL_1181717, EPI_ISL_1591591, EPI_ISL_2350097	Instituto Nacional de Medicina Genomica	Instituto Nacional de Medicina Genomica	Alcaraz N; Canseco Mendez JC; Cedro-Tanda A; Cisneros- Villanueva M; Cisneros-Villanueva M; Escobar-Arrazola; Garcia-Cardenas FJ; Gonzalez-Barrera D; Gonzalez-Woge MA; Herrera-Montalvo LA; Herrera-Montalvo LA.; Hidalgo-Miranda A; M; Mendoza-Vargas A; Miranda-Ortiz H; Munguia-Garza P; Peñaloza-Figueroa F; Ramirez-Vega O; Rangel-DeLeon D; Reyes-Grajeda JP; Rosas-Escobar P; Sifuentes-Rojas C
EPI_ISL_536486	Instituto Nacional de Salud	Laboratorio de Infecciones Respiratorias Agudas	David Tarazona; Dennis Carhuaricra; Eduardo Juscamayta Lopez; Faviola Valdivia Guerrero; Lenin Maturrano Hernandez; Nancy Rojas Serrano; Ronnie Gavilan Chavez
EPI_ISL_2617094, EPI_ISL_2617103, EPI_ISL_2617120, EPI_ISL_2617127, EPI_ISL_2617139, EPI_ISL_2617149, EPI_ISL_2617152, EPI_ISL_2617155, EPI_ISL_2617168			
see above	Instituto Nacional de Saude (INS), Mozambique	CERI, Centre for Epidemic Response and Innvoation, Stellenbosch University and KRISP, KZN Research Innovation and Sequencing Platform, UKZN.	Emmanuel S; Giandhari J; Nadia Sitoe; Nalia Ismael; Nedio Mabunda; Paulo Arnaldo; Pillay S; Tegally H; Wilkinson E; de Oliveira T
EPI_ISL_887437, EPI_ISL_887438, EPI_ISL_887502	Instituto Nacional de Saude (INS), Mozambique	KRISP, KZN Research Innovation and Sequencing Platform	Giandhari J; Nadia Sitoe; Nalia Ismael; Nedio Mabunda; Paulo Arnaldo; Pillay S; Tegally H; Wilkinson E; de Oliveira T
EPI_ISL_693525, EPI_ISL_693610, EPI_ISL_801521, EPI_ISL_1399293, EPI_ISL_1399957, EPI_ISL_1853678, EPI_ISL_2536040, EPI_ISL_2536047, EPI_ISL_2536070, EPI_ISL_2536072, EPI_ISL_2536078, EPI_ISL_2536079, EPI_ISL_2536080, EPI_ISL_2536092, EPI_ISL_2536101			
see above	Instituto Nacional de Saude (INSA)	Instituto Nacional de Saude (INSA)	Borges et al
EPI_ISL_2340402, EPI_ISL_2340436	Instituto Nacional de Saude (INSA)	Instituto Nacional de Saude (INSA) and BioSystems & Integrative Sciences Institute (BioISI) Genomics Unit, FCUL	Borges et al
EPI_ISL_1853820	Instituto Nacional de Saude (INSA) and Centro de Investigaçao em Biodiversidade e Recursos Geneticos (CIBIO), Universidade do Porto	Instituto Nacional de Saude (INSA) and Centro de Investigaçao em Biodiversidade e Recursos Geneticos (CIBIO), Universidade do Porto	Borges et al
EPI_ISL_1399855, EPI_ISL_1853605, EPI_ISL_1853624	Instituto Nacional de Saude (INSA) and Instituto Gulbenkian de Ciencia (IGC)	Instituto Nacional de Saude (INSA) and Instituto Gulbenkian de Ciencia (IGC)	Borges et al
EPI_ISL_2004296, EPI_ISL_2004324	Instituto Nacional de Saude (INSA) and i3S - Instituto de Investigação e Inovação em Saúde	Instituto Nacional de Saude (INSA)	Borges et al
EPI_ISL_2004225	Instituto Nacional de Saude (INSA) and i3S - Instituto de Investigação e Inovação em Saúde	Instituto Nacional de Saude (INSA) and i3S - Instituto de Investigação e Inovação em Saúde	Borges et al
EPI_ISL_455435, EPI_ISL_872093	Instituto de Diagnostico y Referencia Epidemiologicos (INDRE)	Instituto de Diagnostico y Referencia Epidemiologicos (INDRE)	Abril Rodriguez-Maldonado; Adnan-Araiza Rodríguez; Araiza-Rodriguez Adnan; Barrera-Badillo Gisela; Claudia Wong-Arambula; Dayanira Arellano-Suarez; Edgar Mendieta-Condado; Ernesto Ramirez-Gonzalez; Fabiola Garces-Ayala; Garces-Ayala Fabiola. Taboada Ramírez Blanca. Ramirez-Gonzalez Ernesto; Gisela Barrera-Badillo; Hernandez-Rivas Lucia; Irma Lopez-Martinez.; Lopez-Martinez Irma; Lucia Hernandez-Rivas; Mendieta-Condado Edgar; Natividad Cruz-Ortiz; Rodriguez-Maldonado Abril; Tatiana Nunez-Garcia; Wong-Arambula Claudia
EPI_ISL_452139	Instituto de Diagnostico y Referencia Epidemiologicos (INDRE)	Instituto de diagnóstico y Referencia Epidemiologicos (INDRE)	Araiza-Rodriguez Adnan; Barrera-Badillo Gisela; Garces-Ayala Fabiola; Hernandez-Rivas Lucia; Lopez-Martinez Irma; Mendieta-Condado Edgar; Ramirez-Gonzalez Ernesto; Rodriguez-Maldonado Abril; Wong-Arambula Claudia
EPI_ISL_913935, EPI_ISL_913947, EPI_ISL_913961, EPI_ISL_933712, EPI_ISL_1054959, EPI_ISL_1054963, EPI_ISL_1054995, EPI_ISL_1168463, EPI_ISL_1168527, EPI_ISL_1168617			
see above	Instituto de Diagnostico y Referencia Epidemiologicos INDRE_RNLSP	Instituto de Diagnostico y Referencia Epidemiologicos (INDRE)	Abril Rodriguez-Maldonado; Adnan Araiza-Rodriguez; Ariadna Medina-Benitez; Claudia Wong-Arambula; David Fragoso-Fonseca; Ernesto Ramirez-Gonzalez.; Fabiola Garces-Ayala; Gisela Barrera-Badillo; Irma Lopez-Martinez; Joaquin Quiroz-Mercado; Lucia Hernandez-Rivas; Mayra Jimenez-Morales; Nancy Munoz-Hernandez; Natividad Cruz-Ortiz; Sergio Rangel-Guerrero; Tatiana Nunez-Garcia; Vanessa Rivero-Arredondo
EPI_ISL_1301444, EPI_ISL_1301480, EPI_ISL_1301687, EPI_ISL_1302300	Instituto de Diagnostico y Referencia Epidemiologicos InDRE_RNLSP	Instituto de Biotecnología de la UNAM	Alejandra Hernández-Terán; Alejandro Sanchez-Flores; Alma Rincón-Rubio; Andrea Santos Coy-Arechavaleta; Authors from IBT; Blanca Taboada; Celia Boukadida; Clara Esperanza Santacruz-Tinoco; Edgar Mendieta-Condado; Eduardo Becerril-Vargas; Fidencio Mejía-Nepomuceno; Francisco Pulido; Gisela Barrera-Badillo; Gloria Vazquez; Hector Esteban Paz-Juárez; IMSS; InDRE and INER (in alphabetical order): Carlos F. Arias; Irma Lopez-Martinez; Jerome Jean Verleyen; Joel Armando Vázquez-Pérez; Jorge Salas-Hernández; José Arturo Martínez-Orozco; José Ernesto Ramírez-González; José Esteban Muñoz-Medina; Larissa Fernandes-Matano; Lucia Hernandez-Rivas; Luis Alberto Ochoa-Carrera; Margarita Matías-Florentino; Mario Mújica-Sánchez; Natividad Cruz-Ortiz; Pavel Isa; Ricardo Grande; Santiago Ávila-Ríos; Tatiana Nunez-Garcia; Teresita Rojas-Mendoza
EPI_ISL_1629792, EPI_ISL_1629794, EPI_ISL_1629798, EPI_ISL_1629800, EPI_ISL_1629801, EPI_ISL_1629804, EPI_ISL_1629806			
see above	Instituto de Medicina Tropical Alexander Von Humboldt, Universidad Peruana Cayetano Heredia	Laboratorio de Genómica Microbiana, Universidad Peruana Cayetano Heredia	Alejandra Dávila-Barclay; Diego Cuicapuza; Guillermo Salvatierra; Janet Huancachoque; Lenin Maturrano; Luis González; Pablo Tsukayama; Pedro E. Romero; Pool Marcos
EPI_ISL_1652056, EPI_ISL_1652062	Integrated Biorepository of H3Africa Uganda – IBRH3AU	Molecular Biology Laboratory	Ashaba Fred Katabazi; Bernard Ssentalo Bagaya; David Patrick Kateete; Edgar Kigozi; Emmanuel Nasinghe; Eric Katagirya; Gerald Mboowa; Lwanga Newton; Misaki Wayengera; Moses Joloba; Moses Luutu; Nsubuga Gideon; Rogers Kamulegeya; Samuel Kirimunda; Sarah Stanley; Savannah Mwesigwa
EPI_ISL_2001653	Intrepid Nepal	Center for Molecular Dynamics Nepal	Ajit Poudel; Anupama Gurung; Dibesh Karmacharya; Kavya Dhital; Prajwol Manandhar; Rajindra Napit; Roji Raut; Saman Pradhan; Sapana Shakya
EPI_ISL_2290665, EPI_ISL_2290666	Invicta	1. National Institute of Public Health - National Institute of Hygiene, Warsaw, Poland 2. Biobank Lab, University of Lodz 3. Laboratory of Respiratory Viruses, Teaching and Clinical Center of the Medical University of Lodz	Dominik Strapagiel; Izabela Dróżdż; Jakub Lach; Katarzyna Zacharczuk; Klaudyna Królikowska; Maciej Borowiec; Magdalena Nowakowska; Magdalena Traczyk-Borszyńska; Marcin Słomka; Marta Sobalska-Kwapis; Małgorzata Sadkowska-Todys; Tomasz Płoszaj; Tomasz Wołkowicz
EPI_ISL_1919422	Ipoh Public Health Laboratory (MKAI), Ministry of Health Malaysia	Institute for Medical Research, Infectious Disease Research Centre, National Institutes of Health, Ministry	Kamel K; Mohd Zawawi Z; Suppiah J; Thayan R
EPI_ISL_1821883, EPI_ISL_2621621	Ipoh Public Health Laboratory (MKAI), Ministry of Health Malaysia	Institute for Medical Research, Infectious Disease Research Centre, National Institutes of Health, Ministry of Health Malaysia	Azizan MA; Kamel K; Mohd Zawawi Z; Ramly N; Robert F; Suppiah J; Thayan R

EPI_ISL_1910386, EPI_ISL_1910387, EPI_ISL_1910388, EPI_ISL_1910390, EPI_ISL_1910391, EPI_ISL_1910392, EPI_ISL_1910393, EPI_ISL_1910394, EPI_ISL_2620888, EPI_ISL_2620889			
see above	Iressef Genomics lab	IRESSEF	Abdou PADANE; Ambroise AHOUIDI; Aminata DIA; Aminata MBOUP; Astou Gaye GAYE; Barada CISSE; Birahim Piere NDIAYE; Diabou Diagne; Gora LO; Khadim GUEYE; Moustapha MBOW; Nafisatou LEYE; Ndeye Coumba Toure KANE; Papa Alassane DIAW; Samba Ndiour; Souleymane MBOUP; Yacine DIA
EPI_ISL_1167128	Iressef Genomics lab	L'institut de Recherche en Santé, de Surveillance Épidémiologique et de Formation (IRESSEF)	Abdou PADANE; Abdoulie KANTEH; Abdul Karim SESAY; Ambroise AHOUIDI; Aminata DIA; Aminata MBOUP; Astou Gaye GAYE; Barada CISSE; Birahim Piere NDIAYE; Gora LO; Khadim GUEYE; Moustapha MBOW; Nafisatou LEYE; Ndeye Coumba Toure KANE; Papa Alassane DIAW; Souleymane MBOUP; Yacine DIA
EPI_ISL_1298481	Ivano-Frankivsk CCH#1	The Institute of Molecular Biology and Genetics of NASU	M.Tukalo et al.
EPI_ISL_1742834, EPI_ISL_2195378, EPI_ISL_2195723, EPI_ISL_2195974, EPI_ISL_2195975, EPI_ISL_2204264	J.W. Ruby Memorial Hospital	WVU and Marshall University Combined Genomics Core Facilities	James Denvir; Peter Perrotta; Peter Stoilov; Ryan Percifield; Wesley Kimble
EPI_ISL_779276, EPI_ISL_779288, EPI_ISL_2466716, EPI_ISL_2466724, EPI_ISL_2466729, EPI_ISL_2466732	Jamil-ur-Rahman Center for Genome Research, Dr. Panjwani Center for Molecular Medicine and Drug Research	Jamil-ur-Rahman Center for Genome Research, Dr. Panjwani Center for Molecular Medicine and Drug Research	Ain, Nu.; Ansari, S.; B.T.; I.A.; Iqbal, W.; Irfan, M.; Jahan, S.; Javed; Kakar, N.; Khan; Khan, I.; Khan, S.; Nisa, Z.; Rashid, M.; Rehman, Z.; Sarwar, B.; Shakeel, M.; Siddiqi, S.; Zehra, M.
EPI_ISL_451958	Jamil-ur-Rahman Center for Genome Research, Dr. Panjwani Center for Molecular Medicine and Drug Research, International Center for Chemical and Biological Sciences, University of Karachi	Jamil-ur-Rahman Center for Genome Research, Dr. Panjwani Center for Molecular Medicine and Drug Research, International Center for Chemical and Biological Sciences, University of Karachi	B.A.; I.A; Khan; Khan, S.; M.A.and Khan; Qureshi; Raza; S.A.; Shakeel, M.; Zahid, M.
EPI_ISL_1468625, EPI_ISL_1662783, EPI_ISL_2103264, EPI_ISL_2331496	Johns Hopkins Hospital Department of Pathology	Johns Hopkins Hospital Department of Pathology	Adannaya Amadi; C. Paul Morris; Chun Huai Luo; Heba H. Mostafa; Matthew Schwartz; Nicholas Gallagher
EPI_ISL_2695635, EPI_ISL_2695636, EPI_ISL_2695637, EPI_ISL_2695639, EPI_ISL_2695640, EPI_ISL_2695641, EPI_ISL_2695647, EPI_ISL_2695653, EPI_ISL_2695655, EPI_ISL_2695656, EPI_ISL_2695657, EPI_ISL_2695658, EPI_ISL_2695662, EPI_ISL_2695663, EPI_ISL_2695664, EPI_ISL_2695665, EPI_ISL_2695666, EPI_ISL_2695668, EPI_ISL_2695669, EPI_ISL_2695672, EPI_ISL_2695673, EPI_ISL_2695674, EPI_ISL_2695675, EPI_ISL_2695676, EPI_ISL_2695679, EPI_ISL_2695681, EPI_ISL_2695682, EPI_ISL_2695683, EPI_ISL_2695688, EPI_ISL_2695689, EPI_ISL_2695696, EPI_ISL_2695697, EPI_ISL_2695700			
see above	KIMBERLEY LABORATORY	National Institute for Communicable Diseases of the National Health Laboratory Service	Amoako DG; Bhiman JN; Everatt J; Ismail A; Mahlangu B; Mnguni A; Mohale T; Ntuli N; Scheepers C
EPI_ISL_455639	KRISP, KZN Research Innovation and Sequencing Platform	KRISP, KZN Research Innovation and Sequencing Platform	Chimukangara B; Deforche K; Giandhari J; Lessells R; Pillay S; Tegally H; Wilkinson E; de Oliveira T
EPI_ISL_1823618	KY State Public Health Lab	Centers for Disease Control and Prevention Division of Viral Diseases, Pathogen Discovery	Alison Laufer Halpin; Ben L. Rambo-Martin; Clinton R. Paden; Dakota Howard; Darlene Wagner; Dave Wentworth; Dhwani Batra; Jasmine Padilla; Justin Lee; Katie Dillon; Krista Queen; Kristen Knipe; Kristine Lacek; Mark Burroughs; Matthew Schmerer; Mili Sheth; Peter Cook; Sam Shepard; Sarah Nobles; Shoshona Le; Suxiang Tong; Vivien Dugan; Yvette Unoarumhi
EPI_ISL_2658984	Kaiser Santa Clara	Santa Clara County Public Health Laboratory	Santa Clara County Public Health Department
EPI_ISL_510833	Kalmar klinisk mikrobiologi	The Public Health Agency of Sweden	Anna Risberg; Anna-Malin Linde; Karin Tegmark-Wisell; Maria Lind Karlberg; Mattias Haukland; Mia Brytting; Olov Svartstrom; Oskar Karlsson Lindsjo; Petra Edquist; Reza Advani; Sandra Broddesson
EPI_ISL_1805463, EPI_ISL_1805464, EPI_ISL_1857081, EPI_ISL_2007100, EPI_ISL_2534247	Kansas Health and Environmental Lab	Kansas Health and Environmental Lab	Ben Olsen; Jonathan Barnell; Katherine Wiggins; Mike Grose; and Phil Adam
EPI_ISL_1748173	Kantonsarztamt Solothurn	Clinical Bacteriology	Adrian Egli; Alfredo Mari; Fanny Wegner; Fenner; Hans Hirsch; Helena MB Seth-Smith; Julia Bielicki; Karoline Leuzinger; Lukas; Madlen Stange; Manuel Battegay; Tim Roloff
EPI_ISL_1791065, EPI_ISL_1791066	Kantonsspital Baden AG	Institute of Medical Virology, University of Zurich	Alexandra Trkola; Annette Audigé; Cyril Shah; Gabriela Ziltener; Guido Bloemberg; Jon Huder; Jürg Böni; Kevin Steiner; Maria Grünberg; Maryam Zaheri; Michael Huber; Riccarda Capaul; Stefan Schmutz; Verena Kufner
EPI_ISL_1859481	Kazan Federal University, Academic Consortium	FEDERAL RESEARCH AND CLINICAL CENTER OF PHYSICAL-CHEMICAL MEDICINE OF FEDERAL MEDICAL BIOLOGICAL AGENCY, Academic Consortium	Alexander Manolov; Alexander Pavlenko; Alexander Rumyantsev; Alexander Samoilov; Alexander Troitsky; Alexander Tulichev; Alexander Vasiliev; Alexey Volnukhin; Alla Petrova; Andrey Koritsky; Anna Blagonravova; Anna Varizhuk; Daria Boldyreva; Daria Kharlampieva; Daria Matyushkina; Dmitry Fedorov; Dmitry Pushkar; Ekaterina Moskaleva; Elena Galova; Elena Ilina; Elizaveta Orlova; Galina Trifonova; Gaukhar Yusubalieva; Ilshat Gafurov; Irina Shirokova; Konstantin Ivanov; Konstantin Kolontarev; Ksenia Klimina; Larisa Balykova; Lyubov Rychkova; Nadezhda Kolyshkina; Natalia Pozhenko; Oleg Ogarkov; Pavel Bashkirov; Polina Khromova; Polina Tikhonova; Ramiz Bakhtyev; Raushaniya Gaifullina; Sergey Rumyantsev; Svetlana Solovieva; Vadim Govorun; Valentin Manuvera; Varvara Musarova; Vasily Lazarev; Vladimir Baklaushev; Vladimir Rumyantsev; Vladimir Veselovsky; Vladislav Babenko; Vyacheslav Kuropatkin; Vyacheslav Sinkov; Yulia Galeeva; Yuri Udalov
EPI_ISL_1122013	Kentucky State Public Health Lab	Kentucky State Public Health Lab	Joshua Tobias; Karim George; Matthew Johnson; Rachel Zinner; Rhonda Lucas; Stephanie Lunn; Vaneet Arora; William Grooms
EPI_ISL_510869	Klinisk mikrobiologi NAL Trollhattan	The Public Health Agency of Sweden	Anna Risberg; Anna-Malin Linde; Karin Tegmark-Wisell; Maria Lind Karlberg; Mattias Haukland; Mia Brytting; Olov Svartstrom; Oskar Karlsson Lindsjo; Petra Edquist; Reza Advani; Sandra Broddesson
EPI_ISL_2333636	Klinisk mikrobiologi, Region Västerbotten	CBRN Defence and Security, Swedish Defence Research Agency	Andreas Sjödin; Anna-Lena Johansson; Annika Allard; Annika Osterman; Caroline Öhrman; David Sundell; Emelie Näslund Salomonsson; Ingrid Dacklin; Jonas Näslund; Kerstin Myrtennäs; Linda Karlsson; Malin Granberg; Mats Forsman; Stina Bäckman
EPI_ISL_1298485	Kosov CRH	The Institute of Molecular Biology and Genetics of NASU	M.Tukalo et al.
EPI_ISL_2229272	LA Office of Public Health Laboratories	Centers for Disease Control and Prevention Division of Viral Diseases, Pathogen Discovery	Alison Laufer Halpin; Ben L. Rambo-Martin; Clinton R. Paden; Dakota Howard; Darlene Wagner; Dave Wentworth; Dhwani Batra; Jasmine Padilla; Justin Lee; Katie Dillon; Krista Queen; Kristen Knipe; Kristine Lacek; Mark Burroughs; Matthew Schmerer; Mili Sheth; Peter Cook; Sam Shepard; Sarah Nobles; Shoshona Le; Suxiang Tong; Vivien Dugan; Yvette Unoarumhi
EPI_ISL_1318264	LA Office of Public Health Laboratories	Genomics and Discovery, Respiratory Viruses Branch, Division of Viral Diseases, Centers for Disease Control and Prevention	Anna Kelleher; Anna Uehara; Brian Lynch; Clinton R. Paden; Haibin Wang; Han Jia Justin Ng; Jing Zhang; Krista Queen; Peter Cook; Suxiang Tong; Yan Li; Ying Tao
EPI_ISL_1941773	LA SOURCE	Laboratory of genomics and metagenomics	Claire Bertelli; Damien Jacot; Gilbert Greub; Sebastien Aeby; Trestan Pillonel
EPI_ISL_1539136	LABORATORIUM BADAŃ KLINICZNYCH WSSE w OPOLU	1. National Institute of Public Health - National Institute of Hygiene; 2. Eurofins Genomics Europe Sequencing GmbH	ECDC COVID-19 WGS support team; Eurofins Genomics Europe Sequencing Team; Gierczyński Rafał; Sadkowska-Todys Małgorzata; Wołkowicz Tomasz; Zacharczuk Katarzyna
EPI_ISL_2433867	LANCET, LABORATORIES	National Institute for Communicable Diseases of the National Health Laboratory Service	Amoako DG; Bhiman JN; Ismail A; Mahlangu B; Mohale T; Ntuli N; Scheepers C
EPI_ISL_1516786	LESP Aguascalientes	Instituto de Diagnostico y Referencia Epidemiologicos (INDRE)	Abril Rodriguez-Maldonado; Ariadna Medina-Benitez; Claudia Wong-Arambula; Ernesto Ramirez-Gonzalez.; Gisela Barrera-Badillo; Irma Lopez-Martinez; Joaquin Quiroz-Mercado; Lucia Hernandez-Rivas; Natividad Cruz-Ortiz; Sergio Rangel-Guerrero; Tatiana Nunez-Garcia; Vanessa Rivero-Arredondo
EPI_ISL_2443047, EPI_ISL_2545705, EPI_ISL_2545807	LESP Baja California	Instituto de Diagnostico y Referencia Epidemiologicos (INDRE)	Abril Rodriguez-Maldonado; Ariadna Medina-Benitez; Claudia Wong-Arambula; Ernesto Ramirez-Gonzalez.; Gisela Barrera-Badillo; Irma Lopez-Martinez; Joaquin Quiroz-Mercado; Lucia Hernandez-Rivas; Natividad Cruz-Ortiz; Sergio Rangel-Guerrero; Tatiana Nunez-Garcia; Vanessa Rivero-Arredondo
EPI_ISL_1805487	LESP Baja California Sur	Instituto de Diagnostico y Referencia Epidemiologicos (INDRE)	Abril Rodriguez-Maldonado; Ariadna Medina-Benitez; Claudia Wong-Arambula; Ernesto Ramirez-Gonzalez.; Gisela Barrera-Badillo; Irma Lopez-Martinez; Joaquin Quiroz-Mercado; Lucia Hernandez-Rivas; Natividad Cruz-Ortiz; Sergio Rangel-Guerrero; Tatiana Nunez-Garcia; Vanessa Rivero-Arredondo
EPI_ISL_1400299, EPI_ISL_2094512	LESP Campeche	Instituto de Diagnostico y Referencia Epidemiologicos (INDRE)	Abril Rodriguez-Maldonado; Ariadna Medina-Benitez; Claudia Wong-Arambula; Ernesto Ramirez-Gonzalez.; Gisela Barrera-Badillo; Irma Lopez-Martinez; Joaquin Quiroz-Mercado; Lucia Hernandez-Rivas; Natividad Cruz-Ortiz; Sergio Rangel-Guerrero; Tatiana Nunez-Garcia; Vanessa Rivero-Arredondo
EPI_ISL_2343024	LESP Chiapas	Instituto de Diagnostico y Referencia Epidemiologicos (INDRE)	Abril Rodriguez-Maldonado; Ariadna Medina-Benitez; Claudia Wong-Arambula; Ernesto Ramirez-Gonzalez.; Gisela Barrera-Badillo; Irma Lopez-Martinez; Joaquin Quiroz-Mercado; Lucia Hernandez-Rivas; Natividad Cruz-Ortiz; Sergio Rangel-Guerrero; Tatiana Nunez-Garcia; Vanessa Rivero-Arredondo
EPI_ISL_1558791	LESP Chihuahua	Instituto de Diagnostico y Referencia Epidemiologicos (INDRE)	Abril Rodriguez-Maldonado; Ariadna Medina-Benitez; Claudia Wong-Arambula; Ernesto Ramirez-Gonzalez.; Gisela Barrera-Badillo; Irma Lopez-Martinez; Joaquin Quiroz-Mercado; Lucia Hernandez-Rivas; Natividad Cruz-Ortiz; Sergio Rangel-Guerrero; Tatiana Nunez-Garcia; Vanessa Rivero-Arredondo
EPI_ISL_2101901	LESP Coahuila	Instituto de Diagnostico y Referencia Epidemiologicos (INDRE)	Abril Rodriguez-Maldonado; Ariadna Medina-Benitez; Claudia Wong-Arambula; Ernesto Ramirez-Gonzalez.; Gisela Barrera-Badillo; Irma Lopez-Martinez; Joaquin Quiroz-Mercado; Lucia Hernandez-Rivas; Natividad Cruz-Ortiz; Sergio Rangel-Guerrero; Tatiana Nunez-Garcia; Vanessa Rivero-Arredondo
EPI_ISL_2455980	LESP Colima	Instituto de Diagnostico y Referencia Epidemiologicos (INDRE)	Abril Rodriguez-Maldonado; Ariadna Medina-Benitez; Claudia Wong-Arambula; Ernesto Ramirez-Gonzalez.; Gisela Barrera-Badillo; Irma Lopez-Martinez; Joaquin Quiroz-Mercado; Lucia Hernandez-Rivas; Natividad Cruz-Ortiz; Sergio Rangel-Guerrero; Tatiana Nunez-Garcia; Vanessa Rivero-Arredondo
EPI_ISL_2545805	LESP Durango	Instituto de Diagnostico y Referencia Epidemiologicos (INDRE)	Abril Rodriguez-Maldonado; Ariadna Medina-Benitez; Claudia Wong-Arambula; Ernesto Ramirez-Gonzalez.; Gisela Barrera-Badillo; Irma Lopez-Martinez; Joaquin Quiroz-Mercado; Lucia Hernandez-Rivas; Natividad Cruz-Ortiz; Sergio Rangel-Guerrero; Tatiana Nunez-Garcia; Vanessa Rivero-Arredondo
EPI_ISL_1340616, EPI_ISL_2341011	LESP Guanajuato	Instituto de Diagnostico y Referencia Epidemiologicos (INDRE)	Abril Rodriguez-Maldonado; Ariadna Medina-Benitez; Claudia Wong-Arambula; Ernesto Ramirez-Gonzalez.; Gisela Barrera-Badillo; Irma Lopez-Martinez; Joaquin Quiroz-Mercado; Lucia Hernandez-Rivas; Natividad Cruz-Ortiz; Sergio Rangel-Guerrero; Tatiana Nunez-Garcia; Vanessa Rivero-Arredondo
EPI_ISL_1673265, EPI_ISL_2495988	LESP Guerrero	Instituto de Diagnostico y Referencia Epidemiologicos (INDRE)	Abril Rodriguez-Maldonado; Ariadna Medina-Benitez; Claudia Wong-Arambula; Ernesto Ramirez-Gonzalez.; Gisela Barrera-Badillo; Irma Lopez-Martinez; Joaquin Quiroz-Mercado; Lucia Hernandez-Rivas; Natividad Cruz-Ortiz; Sergio Rangel-Guerrero; Tatiana Nunez-Garcia; Vanessa Rivero-Arredondo
EPI_ISL_1424008, EPI_ISL_1661541, EPI_ISL_1805483	LESP Hidalgo	Instituto de Diagnostico y Referencia Epidemiologicos (INDRE)	Abril Rodriguez-Maldonado; Ariadna Medina-Benitez; Claudia Wong-Arambula; Ernesto Ramirez-Gonzalez.; Gisela Barrera-Badillo; Irma Lopez-Martinez; Joaquin Quiroz-Mercado; Lucia Hernandez-Rivas; Natividad Cruz-Ortiz; Sergio Rangel-Guerrero; Tatiana Nunez-Garcia; Vanessa Rivero-Arredondo
EPI_ISL_1405908, EPI_ISL_2094505, EPI_ISL_2101905	LESP Jalisco	Instituto de Diagnostico y Referencia Epidemiologicos (INDRE)	Abril Rodriguez-Maldonado; Ariadna Medina-Benitez; Claudia Wong-Arambula; Ernesto Ramirez-Gonzalez.; Gisela Barrera-Badillo; Irma Lopez-Martinez; Joaquin Quiroz-Mercado; Lucia Hernandez-Rivas; Natividad Cruz-Ortiz; Sergio Rangel-Guerrero; Tatiana Nunez-Garcia; Vanessa Rivero-Arredondo
EPI_ISL_1558830	LESP Jalisco/UdeG	Instituto de Diagnostico y Referencia Epidemiologicos (INDRE)	Abril Rodriguez-Maldonado; Ariadna Medina-Benitez; Claudia Wong-Arambula; Ernesto Ramirez-Gonzalez.; Gisela Barrera-Badillo; Irma Lopez-Martinez; Joaquin Quiroz-Mercado; Lucia Hernandez-Rivas; Natividad Cruz-Ortiz; Sergio Rangel-Guerrero; Tatiana Nunez-Garcia; Vanessa Rivero-Arredondo

EPI_ISL_2246831, EPI_ISL_2545702	LESP Michoacan	Instituto de Diagnostico y Referencia Epidemiologicos (INDRE)	Abril Rodriguez-Maldonado; Ariadna Medina-Benitez; Claudia Wong-Arambula; Ernesto Ramirez-Gonzalez.; Gisela Barrera-Badillo; Irma Lopez-Martinez; Joaquin Quiroz-Mercado; Lucia Hernandez-Rivas; Natividad Cruz-Ortiz; Sergio Rangel-Guerrero; Tatiana Nunez-Garcia; Vanessa Rivero-Arredondo
EPI_ISL_1400303, EPI_ISL_1400308, EPI_ISL_1710967, EPI_ISL_2296094	LESP Morelos	Instituto de Diagnostico y Referencia Epidemiologicos (INDRE)	Abril Rodriguez-Maldonado; Ariadna Medina-Benitez; Claudia Wong-Arambula; Ernesto Ramirez-Gonzalez.; Gisela Barrera-Badillo; Irma Lopez-Martinez; Joaquin Quiroz-Mercado; Lucia Hernandez-Rivas; Natividad Cruz-Ortiz; Sergio Rangel-Guerrero; Tatiana Nunez-Garcia; Vanessa Rivero-Arredondo
EPI_ISL_1483041	LESP Nayarit	Instituto de Diagnostico y Referencia Epidemiologicos (INDRE)	Abril Rodriguez-Maldonado; Ariadna Medina-Benitez; Claudia Wong-Arambula; Ernesto Ramirez-Gonzalez.; Gisela Barrera-Badillo; Irma Lopez-Martinez; Joaquin Quiroz-Mercado; Lucia Hernandez-Rivas; Natividad Cruz-Ortiz; Sergio Rangel-Guerrero; Tatiana Nunez-Garcia; Vanessa Rivero-Arredondo
EPI_ISL_1365648, EPI_ISL_1516771, EPI_ISL_1661555, EPI_ISL_1661563	LESP Nuevo Leon	Instituto de Diagnostico y Referencia Epidemiologicos (INDRE)	Abril Rodriguez-Maldonado; Ariadna Medina-Benitez; Claudia Wong-Arambula; Ernesto Ramirez-Gonzalez.; Gisela Barrera-Badillo; Irma Lopez-Martinez; Joaquin Quiroz-Mercado; Lucia Hernandez-Rivas; Natividad Cruz-Ortiz; Sergio Rangel-Guerrero; Tatiana Nunez-Garcia; Vanessa Rivero-Arredondo
EPI_ISL_2157331	LESP Oaxaca	Instituto de Diagnostico y Referencia Epidemiologicos (INDRE)	Abril Rodriguez-Maldonado; Ariadna Medina-Benitez; Claudia Wong-Arambula; Ernesto Ramirez-Gonzalez.; Gisela Barrera-Badillo; Irma Lopez-Martinez; Joaquin Quiroz-Mercado; Lucia Hernandez-Rivas; Natividad Cruz-Ortiz; Sergio Rangel-Guerrero; Tatiana Nunez-Garcia; Vanessa Rivero-Arredondo
EPI_ISL_2340953	LESP Puebla	Instituto de Diagnostico y Referencia Epidemiologicos (INDRE)	Abril Rodriguez-Maldonado; Ariadna Medina-Benitez; Claudia Wong-Arambula; Ernesto Ramirez-Gonzalez.; Gisela Barrera-Badillo; Irma Lopez-Martinez; Joaquin Quiroz-Mercado; Lucia Hernandez-Rivas; Natividad Cruz-Ortiz; Sergio Rangel-Guerrero; Tatiana Nunez-Garcia; Vanessa Rivero-Arredondo
EPI_ISL_1424021, EPI_ISL_1558795, EPI_ISL_1821132, EPI_ISL_2455898, EPI_ISL_2455906, EPI_ISL_2455961, EPI_ISL_2495926, EPI_ISL_2495959, EPI_ISL_2545720			
see above	LESP Queretaro	Instituto de Diagnostico y Referencia Epidemiologicos (INDRE)	Abril Rodriguez-Maldonado; Ariadna Medina-Benitez; Claudia Wong-Arambula; Ernesto Ramirez-Gonzalez.; Gisela Barrera-Badillo; Irma Lopez-Martinez; Joaquin Quiroz-Mercado; Lucia Hernandez-Rivas; Natividad Cruz-Ortiz; Sergio Rangel-Guerrero; Tatiana Nunez-Garcia; Vanessa Rivero-Arredondo
EPI_ISL_2559454	LESP San Luis Potosi	Instituto de Diagnostico y Referencia Epidemiologicos (INDRE)	Abril Rodriguez-Maldonado; Ariadna Medina-Benitez; Claudia Wong-Arambula; Ernesto Ramirez-Gonzalez.; Gisela Barrera-Badillo; Irma Lopez-Martinez; Joaquin Quiroz-Mercado; Lucia Hernandez-Rivas; Natividad Cruz-Ortiz; Sergio Rangel-Guerrero; Tatiana Nunez-Garcia; Vanessa Rivero-Arredondo
EPI_ISL_1337389, EPI_ISL_2496013	LESP Sinaloa	Instituto de Diagnostico y Referencia Epidemiologicos (INDRE)	Abril Rodriguez-Maldonado; Ariadna Medina-Benitez; Claudia Wong-Arambula; Ernesto Ramirez-Gonzalez.; Gisela Barrera-Badillo; Irma Lopez-Martinez; Joaquin Quiroz-Mercado; Lucia Hernandez-Rivas; Natividad Cruz-Ortiz; Sergio Rangel-Guerrero; Tatiana Nunez-Garcia; Vanessa Rivero-Arredondo
EPI_ISL_1821193, EPI_ISL_2495979	LESP Tabasco	Instituto de Diagnostico y Referencia Epidemiologicos (INDRE)	Abril Rodriguez-Maldonado; Ariadna Medina-Benitez; Claudia Wong-Arambula; Ernesto Ramirez-Gonzalez.; Gisela Barrera-Badillo; Irma Lopez-Martinez; Joaquin Quiroz-Mercado; Lucia Hernandez-Rivas; Natividad Cruz-Ortiz; Sergio Rangel-Guerrero; Tatiana Nunez-Garcia; Vanessa Rivero-Arredondo
EPI_ISL_1424041	LESP Tamaulipas	Instituto de Diagnostico y Referencia Epidemiologicos (INDRE)	Abril Rodriguez-Maldonado; Ariadna Medina-Benitez; Claudia Wong-Arambula; Ernesto Ramirez-Gonzalez.; Gisela Barrera-Badillo; Irma Lopez-Martinez; Joaquin Quiroz-Mercado; Lucia Hernandez-Rivas; Natividad Cruz-Ortiz; Sergio Rangel-Guerrero; Tatiana Nunez-Garcia; Vanessa Rivero-Arredondo
EPI_ISL_1821166	LESP Tlaxcala	Instituto de Diagnostico y Referencia Epidemiologicos (INDRE)	Abril Rodriguez-Maldonado; Ariadna Medina-Benitez; Claudia Wong-Arambula; Ernesto Ramirez-Gonzalez.; Gisela Barrera-Badillo; Irma Lopez-Martinez; Joaquin Quiroz-Mercado; Lucia Hernandez-Rivas; Natividad Cruz-Ortiz; Sergio Rangel-Guerrero; Tatiana Nunez-Garcia; Vanessa Rivero-Arredondo
EPI_ISL_1366666, EPI_ISL_2340908, EPI_ISL_2340927	LESP Yucatan	Instituto de Diagnostico y Referencia Epidemiologicos (INDRE)	Abril Rodriguez-Maldonado; Ariadna Medina-Benitez; Claudia Wong-Arambula; Ernesto Ramirez-Gonzalez.; Gisela Barrera-Badillo; Irma Lopez-Martinez; Joaquin Quiroz-Mercado; Lucia Hernandez-Rivas; Natividad Cruz-Ortiz; Sergio Rangel-Guerrero; Tatiana Nunez-Garcia; Vanessa Rivero-Arredondo
EPI_ISL_2495971, EPI_ISL_2495973	LESP Zacatecas	Instituto de Diagnostico y Referencia Epidemiologicos (INDRE)	Abril Rodriguez-Maldonado; Ariadna Medina-Benitez; Claudia Wong-Arambula; Ernesto Ramirez-Gonzalez.; Gisela Barrera-Badillo; Irma Lopez-Martinez; Joaquin Quiroz-Mercado; Lucia Hernandez-Rivas; Natividad Cruz-Ortiz; Sergio Rangel-Guerrero; Tatiana Nunez-Garcia; Vanessa Rivero-Arredondo
EPI_ISL_1700833	LESPZacatecas	Instituto de Diagnostico y Referencia Epidemiologicos (INDRE)	Abril Rodriguez-Maldonado; Ariadna Medina-Benitez; Claudia Wong-Arambula; Ernesto Ramirez-Gonzalez.; Gisela Barrera-Badillo; Irma Lopez-Martinez; Joaquin Quiroz-Mercado; Lucia Hernandez-Rivas; Natividad Cruz-Ortiz; Sergio Rangel-Guerrero; Tatiana Nunez-Garcia; Vanessa Rivero-Arredondo
EPI_ISL_579031	LSUHS Emerging Viral Threat Laboratory	Microbial Genome Sequencing Center	Andrew D. Yurochko; Christopher G. Kevil; Daniel J. Snyder; Jeremy P. Kamil; John A. Vanchiere; Katarzyna Zwolinska; Maarten Van Diest; Malgorzata Bienkowska-Haba; Martin J. Sapp; Rona S. Scott; Vaughn S. Cooper
EPI_ISL_2695351	Lab Dra Celeste Formosinho	Instituto Nacional de Saude (INSA)	Borges et al
EPI_ISL_456382, EPI_ISL_456390, EPI_ISL_548084, EPI_ISL_579425, EPI_ISL_637089, EPI_ISL_877212, EPI_ISL_1016867, EPI_ISL_1172029, EPI_ISL_1172030, EPI_ISL_1172032, EPI_ISL_1250705, EPI_ISL_1250710, EPI_ISL_1315314, EPI_ISL_1315323, EPI_ISL_1469103, EPI_ISL_1621294, EPI_ISL_1621296, EPI_ISL_1621303, EPI_ISL_1621312, EPI_ISL_1621314, EPI_ISL_1621315, EPI_ISL_1621316, EPI_ISL_1621323, EPI_ISL_1621324, EPI_ISL_1621325, EPI_ISL_1904849, EPI_ISL_1904852, EPI_ISL_1904857, EPI_ISL_1967902, EPI_ISL_2103202, EPI_ISL_2406487, EPI_ISL_2406490, EPI_ISL_2406495			
see above	LabPLUS	Institute of Environmental Science and Research (ESR)	Anja Werno; Antje van der Linden; Arlo Upton; Chris Mansell; David Hammer; Dragana Drinkovic; Erasmus Smit; Gary McAuliffe; Hana Sofia Andersson; Hermes Perez; James Ussher; Jill Sherwood; Jing Wang; Joep de Ligt; Josh Freeman; Julia Howard; Juliet Elvy; Lauren Jelly; Mary DeAlmeida; Matt Blakiston; Matt Storey; Matthew Rogers; Max Bloomfield; Michael Addidle; Michelle Balm; Muhammad Faisal; Nikki Freed; Olin Silander; Olivia Stroeven; Rachel Boyle; Sally Roberts; SallyAnn Harbison; Sarah Jefferies; Sharmini Muttaiyah; Susan Morpeth; Susan Taylor; Timothy Blackmore; Vani Sathyendran; Veronica Playle; Virginia Hope; Xiaoyun Ren
EPI_ISL_1113757	LabTests	Institute of Environmental Science and Research (ESR)	Anja Werno; Antje van der Linden; Arlo Upton; Chris Mansell; David Hammer; Dragana Drinkovic; Erasmus Smit; Gary McAuliffe; Hana Sofia Andersson; Hermes Perez; James Ussher; Jill Sherwood; Jing Wang; Joep de Ligt; Josh Freeman; Julia Howard; Juliet Elvy; Lauren Jelly; Mary DeAlmeida; Matt Blakiston; Matt Storey; Matthew Rogers; Max Bloomfield; Michael Addidle; Michelle Balm; Muhammad Faisal; Nikki Freed; Olin Silander; Olivia Stroeven; Rachel Boyle; Sally Roberts; SallyAnn Harbison; Sarah Jefferies; Sharmini Muttaiyah; Susan Morpeth; Susan Taylor; Timothy Blackmore; Vani Sathyendran; Veronica Playle; Virginia Hope; Xiaoyun Ren
EPI_ISL_1219949, EPI_ISL_1219962, EPI_ISL_1219963, EPI_ISL_2188484, EPI_ISL_2188485, EPI_ISL_2188486, EPI_ISL_2188487, EPI_ISL_2188488, EPI_ISL_2188489			
see above	Labo Analyses Med	National Reference Center for Viruses of Respiratory Infections, Institut Pasteur, Paris	Adamou Lagare; Angela Brisebarre; Camille Capel; Christophe Malabat; Corinne Maufrais; Durivault JéRôMe; Etienne Simon-Lorière; Frédéric Lemoine; Gael Millot; Louise Lefrançois; Marion Barbet; Maud Vanpeene; Méline Bizard; Sylvie Behillil; Sylvie Van der Werf; Sylvie van der Werf; Vincent Enouf
EPI_ISL_1508944, EPI_ISL_1508947, EPI_ISL_1508949, EPI_ISL_1509098, EPI_ISL_1509100	Laboratoire Biolim/FSS/UL	Unité Mixte Internationale TransVIHMI (UMI 233 IRD – U1175 INSERM - Université de Montpellier) IRD (Institut de recherche pour le développement)	Abla A. KONOU; Adodo SADJI; Ahidjo AYOUBA; Akoélé SILIADIN; Alassane OURO-MEDELI; Amivi EHLAN; Améyo DORKENOO; Anoumou DAGNRA; Christelle BUTEL; Déléma MABA; Eric DELAPORTE; Issaka Maman; Kokou TEGUENI; Koku AGBODEKA; Laetitia SERRANO; Martine PEETERS; Messanh DOUFFAN; Mireille PRINCE-DAVID; Mounerou SALOU; Sidonie A.M.KAGNISSODE; Sika DOSSIM; Syntyche DEVATCHAGNI; Wembo A. HALATOKO
EPI_ISL_2318037, EPI_ISL_2318899	Laboratoire Central de Virologie	Laboratoire de Biotechnologie	Abdelomunim Essabbar; Amal Zouaki; Ghizlane EL Amin; Hakima Kabbaj; Lahcen Belyamani and Azeddine Ibrahimi; Mouna Ouadghiri; Myriam Seffar; Saaïd Amzazi; Tarik Aanniz
EPI_ISL_1895101, EPI_ISL_1904295, EPI_ISL_1904876	Laboratoire central de Virologie	Laboratoire de Biotechnologie	Abdelomunim Essabbar; Amal Zouaki; Ghizlane EL Amin; Hakima Kabbaj; Lahcen Belyamani and Azeddine Ibrahimi; Mouna Ouadghiri; Myriam Seffar; Saaïd Amzazi; Tarik Aanniz
EPI_ISL_1197037	Laboratoire de virologie clinique - Institut Pasteur de Tunis	1-Laboratory of Microbiology, National Reference Lab, Charles Nicolle Hospital; 2-University of Tunis ElManar, Faculty of Medicine of Tunis, LR99ES09, Tunis, Tunisia	Alia BenKahla; Anissa Chouikha; Fares Wasfi; Henda Triki; Ilhem Boutiba-Ben Boubaker.; Imen Kacem; Ines Mdini; Jalila Ben Khelil; Maher Kharrat; Manel Ben Sassi; Mariem Gdoura; Mouna Ben Sassi; Mouna Safer; Nissaf Ben Alaya; Riadh Daghfous; Riadh Gouider; Roua Ben Othman; Salma Abid; Sameh Trabelsi; Sana Ferjani; Sara Chammam; Sondes Haddad
EPI_ISL_2036262, EPI_ISL_2036263, EPI_ISL_2036264, EPI_ISL_2036265, EPI_ISL_2036266, EPI_ISL_2036267, EPI_ISL_2036268, EPI_ISL_2036269			
see above	Laboratori de Referencia de Catalunya	Laboratori de Referencia de Catalunya	Bellosillo B.; Canal M.; Hernandez JJ.; Padilla E.; Ramirez A.; Vilas A.
EPI_ISL_1661978, EPI_ISL_1662201	Laboratorio Central de Epidemiologia (LCE)	Unidad de Genomica Avanzada	Alejandro Sanchez-Flores; Alfredo Herrera-Estrella; Alicia Ocana-Mondragon; Angel Gustavo Salas-Lais; Bernardo Martinez-Miguel; Blanca Taboada; Brenda Irasema Maldonado-Meza; Carla Ivon Herrera-Najera; Carlos F. Arias; Celia Boukadida; Celida Duque Molina; Clara Esperanza Santacruz-Tinoco; Concepcion Grajales-Muniz; Consorcio Mexicano de Vigilancia Genomica (CoViGen-Mex). Authors (in alphabetical order): Julio Elias Alvarado-Yaah; Fernando Fontove-Herrera; Francisco Pulido; Gloria Elena Espinosa-Ayala; Gloria Maria Molina-Salinas; Gloria Vazquez; Hector Esteban Paz-Juarez; Hector Montoya-Fuentes; Helen Haydee Fernanda Ramirez-Plascencia; Jose Antonio Enciso-Moreno; Jose Esteban Munoz-Medina; Jose de Jesus Nunez-Contreras; Juan Bautista Chale-Dzul; Luis Alberto Ochoa-Carrera; Margarita Matias-Florentino; Maria Guadalupe Santiago-Mauricio; Maria Guadalupe de Jesus Mireles-Rivera; Nelly Selem-Mojica; Pavel Isa; Ricardo Grande; Santiago Ávila-Rios; Victor Eduardo Garcia-Arias; Victor Hugo Borja-Aburto
EPI_ISL_1351883, EPI_ISL_1351905	Laboratorio Central de EpidemiologÃa (LCE)	Unidad de Genomica Avanzada	Alejandro Sanchez-Flores; Alfredo Herrera-Estrella; Alicia Ocana-Mondragon; Angel Gustavo Salas-Lais; Bernardo Martinez-Miguel; Blanca Taboada; Brenda Irasema Maldonado-Meza; Carla Ivon Herrera-Najera; Carlos F. Arias; Celia Boukadida; Clara Esperanza Santacruz-Tinoco; Concepcion Grajales-Muniz; Consorcio Mexicano de Vigilancia Genomica (CoViGen-Mex). Authors (in alphabetical order): Julio Elias Alvarado-Yaah; Fernando Fontove-Herrera; Francisco Pulido; Gloria Elena Espinoza-Ayala; Gloria Maria Molina-Salinas; Gloria Vazquez; Hector Esteban Paz-Juarez; Hector Montoya-Fuentes; Helen Haydee Fernanda Ramirez-Plascencia; Jorge Ivan Salinal-Nevarez; Jose Antonio Enciso-Moreno; Jose Esteban Munoz-Medina; Jose de Jesus Nunez-Contreras; Juan Bautista Chale-Dzul; Luis Alberto Ochoa-Carrera; Margarita Matias-Florentino; Maria Guadalupe Santiago-Mauricio; Maria Guadalupe de Jesus Mireles-Rivera; Nelly Selem-Mojica; Pavel Isa; Ricardo Grande; Santiago Avila-Rios; Victor Hugo Borja-Aburto
EPI_ISL_2091427, EPI_ISL_2490543, EPI_ISL_2490582	Laboratorio Central de Epidemiología (LCE)	Centro de Investigación en Enfermedades Infecciosas (CIENI), Instituto Nacional de Enfermedades Respiratorias (INER)	; Alejandra Garcia-Gasca; Alejandra Hernández-Terán; Alejandro Sanchez-Flores; Alfredo Herrera-Estrella; Alicia Ocaña-Mondragón; Andreu Comas-García; Angel Gustavo Salas-Lais; Antonio Loza Román; Bernardo Martínez-Miguel; Blanca Taboada; Brenda Irasema Maldonado-Meza; Bruno Gomez-Gil; Carla Ivón Herrera-Najera; Carlos F. Arias; Celia Boukadida; Clara Esperanza Santacruz-Tinoco; Concepción Grajales-Muñiz; Consorcio Mexicano de Vigilancia Genómica (CoViGen-Mex). Authors (in alphabetical order): Julio Elias Alvarado-Yaah; Cristóbal Cháidez-Quiróz; Célida Duque Molina; Célida Martínez- Rodríguez; Daniel Fregoso-Rueda; Daniel Lira Morales; Eduardo Becerril-Vargas; Fernando Fontove-Herrera; Fidencio Mejía-Nepomuceno; Francisco Pulido; Gloria Elena Espinosa-Ayala; Gloria María Molina-Salinas; Gloria Vazquez; Hector Esteban Paz-Juárez; Hector Montoya-Fuentes; Helen Haydee Fernanda Ramírez-Plascencia; Irvin González-López; Jean Pierre González; Joel Armando Vázquez-Pérez.; Jorge Salas-Hernández; José Antonio Enciso-Moreno; José Arturo Martínez-Orozco; José Esteban Muñoz-Medina; José de Jesús Nuñez-Contreras; Juan Bautista Chale-Dzul; Julissa Enciso-Ibarra; Luis Alberto Ochoa-Carrera; Margarita Matías-Florentino; Mario Mújica-Sánchez; Marissa Perez-Garcia; María Guadalupe Santiago-Mauricio; María Guadalupe de Jesús Mireles-Rivera; Nelly Sélem-Mojica; Pavel Isa; Ricardo Ciria Merce; Ricardo Grande; Rosa María Gutierrez Rios; Santiago Ávila-Ríos; Selene Zárate; Susana Lopez; Victor Eduardo Garcia-Arias; Víctor Hugo Borja-Aburto
EPI_ISL_1279269, EPI_ISL_1279416, EPI_ISL_1279426, EPI_ISL_1279461, EPI_ISL_1279560, EPI_ISL_1279562, EPI_ISL_1279565, EPI_ISL_1279585, EPI_ISL_1279590, EPI_ISL_1585595, EPI_ISL_1585619, EPI_ISL_1585626			
see above	Laboratorio Central de Epidemiología (LCE)	Instituto Nacional de Enfermedades Respiratorias (INER): Centro de Investigación en Enfermedades Infecciosas (CIENI)	Alejandro Sanchez-Flores; Alfredo Herrera-Estrella; Alicia Ocaña-Mondragón; Angel Gustavo Salas-Lais; Bernardo Martínez-Miguel; Blanca Taboada; Brenda Irasema Maldonado-Meza; Carla Ivón Herrera-Najera; Carlos F. Arias; Celia Boukadida; Clara Esperanza Santacruz-Tinoco; Concepción Grajales-Muñiz; Consorcio Mexicano de Vigilancia Genómica (CoViGen-Mex). Authors (in alphabetical order): Julio Elias Alvarado-Yaah; Consorcio Mexicano de Vigilancia Genómica (CoViGen-Mex). Authors (in alphabetical order): Julio Elias Alvarado-Yaah; Célida Duque Molina; Fernando Fontove-Herrera; Francisco Pulido; Gloria Elena Espinosa-Ayala; Gloria Elena Espinoza-Ayala; Gloria María Molina-Salinas; Gloria Vazquez; Hector Esteban Paz-Juárez; Hector Montoya-Fuentes; Helen Haydee Fernanda Ramírez-Plascencia; Jorge Ivan Salinal-Nevarez; José Antonio Enciso-Moreno; José Esteban Muñoz-Medina; José de Jesús Nuñez-Contreras; Juan Bautista Chale-Dzul; Luis Alberto Ochoa-Carrera; Margarita Matías-Florentino; María Guadalupe Santiago-Mauricio; María Guadalupe de Jesús Mireles-Rivera; Nelly Sélem-Mojica; Pavel Isa; Ricardo Grande; Santiago Ávila-Ríos; Victor Eduardo Garcia-Arias; Víctor Hugo Borja-Aburto
EPI_ISL_1288416, EPI_ISL_1416385, EPI_ISL_1416553, EPI_ISL_1416559, EPI_ISL_1416628, EPI_ISL_1416630, EPI_ISL_1416662, EPI_ISL_1811379, EPI_ISL_1811475, EPI_ISL_2391618, EPI_ISL_2391649, EPI_ISL_2391651			
see above	Laboratorio Central de Epidemiología (LCE)	Instituto de Biotecnología de la UNAM	; Alejandra Garcia-Gasca; Alejandra Hernández-Terán; Alejandro Sanchez-Flores; Alfredo Herrera-Estrella; Alicia Ocaña-Mondragón; Andreu Comas-García; Angel Gustavo Salas-Lais; Antonio Loza Román; Bernardo Martínez-Miguel; Blanca Taboada; Brenda Irasema Maldonado-Meza; Bruno Gomez-Gil; Carla Ivón Herrera-Najera; Carlos F. Arias; Celia Boukadida; Clara Esperanza Santacruz-Tinoco; Concepción Grajales-Muñiz; Consorcio Mexicano de Vigilancia Genómica (CoViGen-Mex). Authors (in alphabetical order): Julio Elias Alvarado-Yaah; Cristóbal Cháidez-Quiróz; Célida Duque Molina; Célida Duque-Molina; Célida Martínez- Rodríguez; Daniel Fregoso-Rueda; Daniel Lira Morales; Eduardo Becerril-Vargas; Fernando Fontove-Herrera; Fidencio Mejía-Nepomuceno; Francisco Pulido; Gloria Elena Espinosa-Ayala; Gloria Elena Espinoza-Ayala; Gloria María Molina-Salinas; Gloria Vazquez; Hector Esteban Paz-Juárez; Hector Montoya-Fuentes; Helen Haydee Fernanda Ramírez-Plascencia; Irvin González-López; Jean Pierre González; Joel Armando Vázquez-Pérez.; Jorge Ivan Salinal-Nevarez; Jorge Salas-Hernández; José Antonio Enciso-Moreno; José Arturo Martínez-Orozco; José Esteban Muñoz-Medina; José de Jesús Nuñez-Contreras; Juan Bautista Chale-Dzul; Julio Elias Alvarado-Yaah; Julissa Enciso-Ibarra; Luis Alberto Ochoa-Carrera; Margarita Matías-Florentino; Mario Mújica-Sánchez; María Guadalupe Santiago-Mauricio; María Guadalupe de Jesús Mireles-Rivera; Nelly Sélem-Mojica; Pavel Isa; Ricardo Ciria Merce; Ricardo Grande; Rosa

			María Gutierrez Rios; Santiago Ávila-Ríos; Selene Zárate; Susana Lopez; Victor Eduardo Garcia-Arias; Víctor Hugo Borja-Aburto
EPI_ISL_1301473, EPI_ISL_1301552, EPI_ISL_1301714	Laboratorio Central de Epidemiología IMSS	Instituto de Biotecnología de la UNAM	Alejandra Hernández-Terán; Alejandro Sanchez-Flores; Alma Rincón-Rubio; Andrea Santos Coy-Arechavaleta; Authors from IBT; Blanca Taboada; Celia Boukadida; Clara Esperanza Santacruz-Tinoco; Edgar Mendieta-Condado; Eduardo Becerril-Vargas; Fidencio Mejía-Nepomuceno; Francisco Pulido; Gisela Barrera-Badillo; Gloria Vazquez; Hector Esteban Paz-Juárez; IMSS; InDRE and INER (in alphabetical order): Carlos F. Arias; Irma Lopez-Martinez; Jerome Jean Verleyen; Joel Armando Vázquez-Pérez; Jorge Salas-Hernández; José Arturo Martínez-Orozco; José Ernesto Ramírez-González; José Esteban Muñoz-Medina; Larissa Fernandes-Matano; Lucia Hernandez-Rivas; Luis Alberto Ochoa-Carrera; Margarita Matías-Florentino; Mario Mújica-Sánchez; Natividad Cruz-Ortiz; Pavel Isa; Ricardo Grande; Santiago Ávila-Ríos; Tatiana Nunez-Garcia; Teresita Rojas-Mendoza
EPI_ISL_2402196, EPI_ISL_2402216	Laboratorio Central de Epidemiología (LCE)	Unidad de Genomica Avanzada	Alejandro Sanchez-Flores; Alfredo Herrera-Estrella; Alicia Ocana-Mondragon; Angel Gustavo Salas-Lais; Bernardo Martinez-Miguel; Blanca Taboada; Brenda Irasema Maldonado-Meza; Carla Ivon Herrera-Najera; Carlos F. Arias; Celia Boukadida; Clara Esperanza Santacruz-Tinoco; Concepcion Grajales-Muniz; Consorcio Mexicano de Vigilancia Genomica (CoViGen-Mex). Authors (in alphabetical order): Julio Elias Alvarado-Yaah; Fernando Fontove-Herrera; Francisco Pulido; Gloria Elena Espinoza-Ayala; Gloria Maria Molina-Salinas; Gloria Vazquez; Hector Esteban Paz-Juarez; Hector Montoya-Fuentes; Helen Haydee Fernanda Ramirez-Plascencia; Jorge Ivan Salinal-Nevarez; Jose Antonio Enciso-Moreno; Jose Esteban Munoz-Medina; Jose de Jesus Nunez-Contreras; Juan Bautista Chale-Dzul; Luis Alberto Ochoa-Carrera; Margarita Matias-Florentino; Maria Guadalupe Santiago-Mauricio; Maria Guadalupe de Jesus Mireles-Rivera; Nelly Selem-Mojica; Pavel Isa; Ricardo Grande; Santiago Avila-Rios; Victor Hugo Borja-Aburto
EPI_ISL_1492643, EPI_ISL_1492645, EPI_ISL_1492646, EPI_ISL_1492653, EPI_ISL_1492674, EPI_ISL_1492675	Laboratorio Central de Salud Publica de Paraguay	Laboratorio Central de Salud Publica de Paraguay	Andrea Gómez de la Fuente; Cynthia Vázquez; Flavia Aburjaile; Juan Torales; Luiz Carlos Junior Alcantara; Marta Giovanetti; María José Ortega; María Liz Gamarra; Shirley Villalba; Talita Adelino; Vagner Fonseca
EPI_ISL_1091264	Laboratorio PGM	Laboratorio de Infectología Molecular, Departamento de Bioquímica y Medicina Molecular,Facultad de Medicina - Universidad Autónoma de Nuevo León	Ana M. Rivas-Estilla; Daniel Arellanos-Soto; Eduardo Garza-de-la-Peña; Gabriela Elizondo; Javier Ramos-Jimenez; Kame A. Galán-Huerta; María F. Herrera-Saldivar; Natalia Martínez-Acuña; Sonia A. Lozano-Sepúlveda
EPI_ISL_457940, EPI_ISL_457962, EPI_ISL_480437	Laboratorio de Biología Molecular Asociación Española Primera en Salud	Departments of Pathology and Medicine, New York University School of Medicine	Adriana Heguy; Christian Marier; Gael Westby; Gonzalo Manrique; Maria Noel Zubillaga; Maria Victoria Elizondo; Matthew T Maurano; Paul Zappile
EPI_ISL_2427590, EPI_ISL_2427592, EPI_ISL_2427605, EPI_ISL_2427616, EPI_ISL_2427619, EPI_ISL_2427627, EPI_ISL_2427628, EPI_ISL_2427644, EPI_ISL_2427655, EPI_ISL_2427661, EPI_ISL_2427692, EPI_ISL_2427723, EPI_ISL_2427753, EPI_ISL_2427754, EPI_ISL_2427763, EPI_ISL_2427775			
see above	Laboratorio de Biología Molecular Médica Uruguaya	Departments of Pathology and Medicine, New York University School of Medicine	Adriana Heguy; Cecilia Sorhouet; Christian Marier; Dacia Dimartino; Gonzalo Manrique; Maria Cristina Mogdasy; Maria Noel Zubillaga; Maria Victoria Elizondo; Paul Zappile
EPI_ISL_1324759, EPI_ISL_1324763	Laboratorio de Diagnostico de Enfermedades Emergentes y Reemergentes	Instituto de Diagnostico y Referencia Epidemiologicos (INDRE)	Abril Rodriguez-Maldonado; Ariadna Medina-Benitez; Claudia Wong-Arambula; Ernesto Ramirez-Gonzalez.; Gisela Barrera-Badillo; Irma Lopez-Martinez; Joaquin Quiroz-Mercado; Lucia Hernandez-Rivas; Natali Vega-Magana; Natividad Cruz-Ortiz; Sergio Rangel-Guerrero; Tatiana Nunez-Garcia; Vanessa Rivero-Arredondo
EPI_ISL_1111423	Laboratorio de Referencia Nacional de Enteropatógenos. Instituto Nacional de Salud del Perú	Laboratorio de Referencia Nacional de Enteropatógenos. Instituto Nacional de Salud del Perú	Fiorella Orellana Peralta; Iris Silva Molina; Junior Caro Castro; Ronnie Gavilan Chavez; Veronica Hurtado Vela; Willi Quino Sifuentes
EPI_ISL_516685	Laboratorio de Referencia Nacional de Virus Respiratorio. Centro Nacional de Salud Publica. Instituto Nacional de Salud Peru.	Laboratorio de Referencia Nacional de Biotecnología y Biología Molecular. Centro Nacional de Salud Publica. Instituto Nacional de Salud Peru.	Carlos Padilla Rojas; Henri Bailon Calderon; Johanna Balbuena Torres; Karolyn Vega Chozo; Marco Galarza Perez; Maribel Huaringa Nuñez; Nancy Rojas Serrano.; Omar Caceres Rey; Priscila Lope Pari
EPI_ISL_491433, EPI_ISL_1092348, EPI_ISL_1137485, EPI_ISL_1137491, EPI_ISL_1532227, EPI_ISL_1532228	Laboratorio de Referencia Nacional de Virus Respiratorio. Instituto Nacional de Salud Perú	Laboratorio de Referencia Nacional de Biotecnología y Biología Molecular. Instituto Nacional de Salud Perú	Carlos Padilla Rojas; Henri Bailon Calderon; Johanna Balbuena Torres; Johanna Balbuena Torrez; Karolyn Vega Chozo; Luis Barcena; Marco Galarza Perez; Maribel Huaringa Nuñez; Nancy Rojas Serrano; Nancy Rojas Serrano.; Omar Caceres Rey; Priscila Lope Pari
EPI_ISL_1111180, EPI_ISL_1111203, EPI_ISL_1111247, EPI_ISL_1111290, EPI_ISL_1111291, EPI_ISL_1111296, EPI_ISL_1111458			
see above	Laboratorio de Referencia Nacional de Virus Respiratorio. Instituto Nacional de Salud Perú	Laboratorio de Referencia Nacional de Enteropatógenos. Instituto Nacional de Salud del Perú	Fiorella Orellana Peralta; Iris Silva Molina; Junior Caro Castro; Ronnie Gavilan Chavez; Veronica Hurtado Vela; Willi Quino Sifuentes
EPI_ISL_812488, EPI_ISL_1629783, EPI_ISL_1629808	Laboratorio de Referencia Nacional de Virus Respiratorios, Instituto Nacional de Salud Peru	Laboratorio de Genómica Microbiana, Universidad Peruana Cayetano Heredia	Alejandra Dávila-Barclay; Brenda Ayzanoa; Camila Castillo-Vilcahuamán; Diego Cuicapuza; Guillermo Salvatierra; Janet Huancachoque; Lenin Maturrano; Luis González; Maribel Huaringa; Nancy Rojas; Oscar Escalante; Pablo Tsukayama; Pedro E. Romero; Pool Marcos; Priscila Lope; Priscilla Lope
EPI_ISL_2536702, EPI_ISL_2536705, EPI_ISL_2536728, EPI_ISL_2536745, EPI_ISL_2536764, EPI_ISL_2536772, EPI_ISL_2536776, EPI_ISL_2536779, EPI_ISL_2536787, EPI_ISL_2536788, EPI_ISL_2536790, EPI_ISL_2536791, EPI_ISL_2536793, EPI_ISL_2536810, EPI_ISL_2536822, EPI_ISL_2536823, EPI_ISL_2536824, EPI_ISL_2536848, EPI_ISL_2536863, EPI_ISL_2536876, EPI_ISL_2562267, EPI_ISL_2562268			
see above	Laboratorio de Referencial Nacional de Virus Respiratorios	Laboratorio de Referencial Nacional de Virus Respiratorios	Carlos Padilla Rojas; Henri Bailon Calderon; Iris Silva Molina; Joseph Huayra Niquen; Lely Solari Zerpa; Luis Barcena Flores; Marco Galarza Perez; Nancy Rojas Serrano; Omar Caceres Rey; Orson Mestanza Millones; Priscila Lope Pari; Sandra Morales Ruiz; Steve Acedo Lazo; Veronica Hurtado Vela
EPI_ISL_2544206	Laboratorio di Microbiologia	Laboratorio di Microbiologia	Martinetti Lucchini Gladys; Valeria Spina
EPI_ISL_1746033	Laboratorium Analiz Lekarskich Alab Rzeszów	1. National Institute of Public Health - National Institute of Hygiene; 2. Eurofins Genomics Europe Sequencing GmbH	ECDC COVID-19 WGS support team; Eurofins Genomics Europe Sequencing Team; Gierczyńki Rafał; Sadkowska-Todys Małgorzata; Wołkowicz Tomasz; Zacharczuk Katarzyna
EPI_ISL_1221236, EPI_ISL_1221498, EPI_ISL_1221598, EPI_ISL_1221862, EPI_ISL_1222319, EPI_ISL_1222343, EPI_ISL_1298343, EPI_ISL_1320824, EPI_ISL_1338963, EPI_ISL_1514376, EPI_ISL_1515032, EPI_ISL_1515630, EPI_ISL_1515661, EPI_ISL_1515706, EPI_ISL_1548630, EPI_ISL_1610960, EPI_ISL_1611370, EPI_ISL_1612169, EPI_ISL_1612208, EPI_ISL_1681305, EPI_ISL_1681478, EPI_ISL_1681577, EPI_ISL_1681600, EPI_ISL_1682964, EPI_ISL_1683153, EPI_ISL_1683867, EPI_ISL_1683986, EPI_ISL_1684717, EPI_ISL_1685045, EPI_ISL_1799003, EPI_ISL_1799336, EPI_ISL_1799474, EPI_ISL_1800829, EPI_ISL_1800981, EPI_ISL_1800987, EPI_ISL_1801017, EPI_ISL_1801330, EPI_ISL_1801360, EPI_ISL_1801624, EPI_ISL_1801691, EPI_ISL_1801976, EPI_ISL_1802058, EPI_ISL_1802476, EPI_ISL_1802644, EPI_ISL_1925625, EPI_ISL_1925681, EPI_ISL_1925936, EPI_ISL_1926073, EPI_ISL_1926250, EPI_ISL_1926283, EPI_ISL_1926372, EPI_ISL_1926482, EPI_ISL_1926580, EPI_ISL_1926717, EPI_ISL_1926968, EPI_ISL_1927603, EPI_ISL_1927971, EPI_ISL_1928600, EPI_ISL_1928896, EPI_ISL_1929055, EPI_ISL_1929232, EPI_ISL_1929301, EPI_ISL_1929356, EPI_ISL_1929448, EPI_ISL_1930528, EPI_ISL_1930706, EPI_ISL_1930789, EPI_ISL_1930858, EPI_ISL_1931019, EPI_ISL_1931052, EPI_ISL_1931275, EPI_ISL_1931296, EPI_ISL_1931734, EPI_ISL_1932000, EPI_ISL_1932009, EPI_ISL_2044007, EPI_ISL_2044013, EPI_ISL_2044036, EPI_ISL_2044077, EPI_ISL_2044210, EPI_ISL_2044211, EPI_ISL_2044343, EPI_ISL_2044454, EPI_ISL_2044524, EPI_ISL_2044588, EPI_ISL_2044622, EPI_ISL_2044694, EPI_ISL_2044695, EPI_ISL_2044706, EPI_ISL_2044786, EPI_ISL_2044941, EPI_ISL_2044942, EPI_ISL_2045014, EPI_ISL_2045112, EPI_ISL_2045385, EPI_ISL_2045440, EPI_ISL_2045668, EPI_ISL_2045783, EPI_ISL_2045847, EPI_ISL_2045856, EPI_ISL_2045974, EPI_ISL_2046278, EPI_ISL_2046354, EPI_ISL_2046357, EPI_ISL_2046388, EPI_ISL_2046570, EPI_ISL_2181883, EPI_ISL_2182032, EPI_ISL_2183055, EPI_ISL_2183147, EPI_ISL_2183734, EPI_ISL_2184155, EPI_ISL_2185103, EPI_ISL_2185253, EPI_ISL_2185260, EPI_ISL_2307071, EPI_ISL_2397967, EPI_ISL_2398058, EPI_ISL_2480895, EPI_ISL_2481235, EPI_ISL_2481952, EPI_ISL_2482161, EPI_ISL_2611161, EPI_ISL_2611207, EPI_ISL_2611321, EPI_ISL_2611400, EPI_ISL_2611407, EPI_ISL_2611486, EPI_ISL_2611493, EPI_ISL_2611575, EPI_ISL_2611589, EPI_ISL_2611628, EPI_ISL_2611659, EPI_ISL_2611707, EPI_ISL_2611723, EPI_ISL_2611746, EPI_ISL_2611766, EPI_ISL_2611789			
see above	Laboratory Corporation of America	Centers for Disease Control and Prevention Division of Viral Diseases, Pathogen Discovery	Adrian Paskey; Amanda Douglas; Amanda Suchanek; Andrea Throop; Ayla Burns; Ben L. Rambo-Martin; Benjamin Rambo-Martin; Bobbi Croy; Brian Krueger; Brian Norvell; Christopher Gulvick; Christos Petropoulos; Clinton R. Paden; Craig Lukasik; Dakota Howard; Darlene Wagner; Debbie Boles; Dhwani Batra; Duncan MacCannell; Eyad Almasri; Goran Stevovic; Howard Engler; Hrushikesh Deshmukh; Jake Humphrey; Jana Schroth; Jason Caravas; Joe Voshell; John Pruitt; Jonathan Meltzer; Jonathan Williams; Kara Moser; Kimberly Wagner; Lax Iyer; Lisa Pfefferle; Lyndon Tilson; Manoj Jain; Marcia Eisenberg; Mary Ann Cristobal; Mary Williamson; Matthew Robinson; Matthew Schmerer; Michael Levandoski; Mike Sapeta; Mindy Nye; Minoo Agarwal; Mohan Kolli; Nuthawin Charoensri; Oren Cohen; Peter W. Cook; Prashant Gupta; Qian Zeng; Rama Ghatti; Scott Parker; Scott Ryan; Scott Sammons; Shatavia Morrison; Stanley Letovsky; Steven Ragan; Suresh Babu Selvaraju; Susan Countryman; Susan Hicks; Suxiang Tong; Suzanne Dale; Thomas Urban; Tim Kuphal; Tricia Zwiefelhofer; Vincent Drouillon; Yvette Unoarumhi
EPI_ISL_1029731, EPI_ISL_1030673, EPI_ISL_1031874	Laboratory Corporation of America	Respiratory Viruses Branch, Division of Viral Diseases, Centers for Disease Control and Prevention	Ben L. Rambo-Martin; Clinton R. Paden; Dakota Howard; Dhwani Batra; Duncan MacCannell; Peter W. Cook; Suxiang Tong
EPI_ISL_754905	Laboratory Diagnostics and Clinical Immunology of Developmental Age, Medical University of Warsaw	genXone SA, Research & Development Laboratory; The Faculty of Mathematics, Informatics and Mechanics of the University of Warsaw	Anna Gambin; Grzegorz Nowicki; Jakub Grabowski; Maciej Sykulski; Michał Kaszuba; Monika Mańkowska-Woźniak; Natalia Drwęska-Matelska; Urszula Demkow; Łukasz Krych
EPI_ISL_415743, EPI_ISL_660543, EPI_ISL_660545, EPI_ISL_2249597, EPI_ISL_2250151, EPI_ISL_2250184	Laboratory Medicine	Department of Laboratory Medicine, Lin-Kou Chang Gung Memorial Hospital, Taoyuan, Taiwan	Cheng-Hsun Chiu; Cheng-Ta Yang; Chi-Hsien Huang; Chung-Guei Huang; Guang-Wu Chen; Kuang-Tso Le; Kuo-Chien Tsao; Kuo-Ming Lee; Mei-Jen Hsiao; Peng-Nien Huang; Po-Wei Huang; Shin-Ru Shih; Shu-Li Yang; Shu-Min Lin; Yi-Chun Liu; Yu-Nong Gong
EPI_ISL_1381307, EPI_ISL_1381327, EPI_ISL_1381360, EPI_ISL_1381361, EPI_ISL_1591117	Laboratory for Respiratory Viruses, Cantacuzino National Military-Medical Institute for Research and Development	Cantacuzino Institute Virology	Catalina Pascu; Cherciu Carmen; Luiza Ustea; Mihaela Lazar; Mihaela Oprea; Nicoleta Paraschiv; Sorin Dinu
EPI_ISL_2153433, EPI_ISL_2154331	Laboratory of Microbiology, National Reference Lab, Charles Nicolle Hospital; 2-University of Tunis ElManar, Faculty of Medicine of Tunis, LR99ES09, Tunis, Tunisia	Clinical and Experimental Pharmacology Lab, LR16SP02, National Center of Pharmacovigilance, University of Tunis El Manar, Tunis, Tunisia. 2-Neurodegenerative diseases and psychiatric troubles, LR18SP03, Razi Hospital, University of Tunis El Manar, Tunis, Tunisia. 3- Ministry of Health, National Observatory of New and Emerging Diseases, 1006, Tunis, Tunisia	Alia BenKahla; Ilhem Boutiba-Ben Boubaker.; Imen Kacem; Imen Mdini; Jalila Ben Khelil; Maher Kharrat; Manel Ben Sassi; Mouna Ben Sassi; Mouna Safer; Nissaf Ben Alaya; Raja Mahfoudh; Riadh Daghfous; Riadh Gouider; Roua Ben Othman; Salma Abid; Sameh Trabelsi; Sana Ferjani; Sarra Chamman; Taha Maatoug
EPI_ISL_1198644	Laboratory of Virology and Molecular Diagnostics	Laboratory of Virology and Molecular Diagnostics, Institute of Public Health of Republic of North Macedonia	Boshevska Golubinka; Janchevska Elizabeta; Kuzmanovska Maja
EPI_ISL_1191740, EPI_ISL_1191780	Laboratory of virology and molecular diagnostics	Laboratory of virology and molecular diagnostics	Boshevska Golubinka; Janchevska Elizabeta; Kuzmanovska Maja
EPI_ISL_1669975, EPI_ISL_1669995, EPI_ISL_2392162	Laboratory of virology and molecular diagnostics, Institute of Public Health	Laboratory of virology and molecular diagnostics, Institute of Public Health	Boshevska G; Janchevska E.; Kuzmanovska M
EPI_ISL_2274445	Labormedizinisches Zentrum Dr Risch	Clinical Bacteriology	Adrian Egli; Alfredo Mari; Hans Hirsch; Helena MB Seth-Smith; Julia Bielicki; Karoline Leuzinger; Lorenz Risch; Madlen Stange; Manuel Battegay; Martin Risch; Nadia Wohlwend; Tim Roloff
EPI_ISL_1499599	Lafene Health Center	Kansas State Veterinary Diagnostic Laboratory	Andreea Lu; Elizabeth Porter; Emily Cox; Jamie Henningson; Jianfa Bai; Kasey Hogan; Lance Noll; Rachel Palinski; Tyler Doerksen
EPI_ISL_447900	Lednicky Laboratory at Emerging Pathogens Institute	University of Florida	Alam; C.J.; Elbadry; Gibson; J.A.; J.C.; J.G.; Lednicky; M.A. and Morris; M.M.; Stephenson
EPI_ISL_1534272	Lentegeur Hospital wc LGH	NHLS/UCT	Arash Iranzadeh; Bruna Galvao; Carolyn Williamson; Deelan Doolabh; Diana Hardie; Innocent Mudau; Kruger Marais; Lynn Tyers; Marvin Hsiao; Stephen Korsman

EPI_ISL_1224742, EPI_ISL_1912729	Lighthouse Lab in Alderley Park	Wellcome Sanger Institute for the COVID-19 Genomics UK (COG-UK) Consortium	Cordelia Langford; David K. Jackson; Dominic Kwiatkowski; Ewan Harrison; Ian Johnston; Jacquelyn Wynn; Jeffrey Barrett; John Sillitoe on behalf of the Wellcome Sanger Institute COVID-19 Surveillance Team; Mairead Hyland; Roberto Amato; Sonia Goncalves; The Lighthouse Lab in Alderley Park and Alex Alderton
EPI_ISL_1635379, EPI_ISL_1635400, EPI_ISL_1653755, EPI_ISL_1673100	Lighthouse Lab in Cambridge	Wellcome Sanger Institute for the COVID-19 Genomics UK (COG-UK) Consortium	Cordelia Langford; David K. Jackson; Dominic Kwiatkowski; Ewan Harrison; Ian Johnston; Jeffrey Barrett; John Sillitoe on behalf of the Wellcome Sanger Institute COVID-19 Surveillance Team; Rob Howes; Roberto Amato; Sonia Goncalves; The Lighthouse Lab in Cambridge and Alex Alderton
EPI_ISL_1208020, EPI_ISL_1635330, EPI_ISL_1741623, EPI_ISL_1741651, EPI_ISL_1741677, EPI_ISL_1741762, EPI_ISL_1741838, EPI_ISL_1741909, EPI_ISL_1758819, EPI_ISL_1758829, EPI_ISL_1806892, EPI_ISL_1829541, EPI_ISL_1829569, EPI_ISL_1830056, EPI_ISL_1830493, EPI_ISL_1830765, EPI_ISL_1830794, EPI_ISL_1830879, EPI_ISL_1830886, EPI_ISL_1831174, EPI_ISL_1912648, EPI_ISL_1912669, EPI_ISL_1912682, EPI_ISL_1912695, EPI_ISL_1912732, EPI_ISL_1939630, EPI_ISL_1939651, EPI_ISL_1939724, EPI_ISL_1939726, EPI_ISL_1939750, EPI_ISL_1939759, EPI_ISL_1939772, EPI_ISL_1939774, EPI_ISL_1939791, EPI_ISL_1939812, EPI_ISL_1986954, EPI_ISL_1986989, EPI_ISL_1987194, EPI_ISL_1987292, EPI_ISL_2022555, EPI_ISL_2022593, EPI_ISL_2022634, EPI_ISL_2022661, EPI_ISL_2022726, EPI_ISL_2022753, EPI_ISL_2022978, EPI_ISL_2092754, EPI_ISL_2122710, EPI_ISL_2122724, EPI_ISL_2122728, EPI_ISL_2122807, EPI_ISL_2122809, EPI_ISL_2122813, EPI_ISL_2122856, EPI_ISL_2122872, EPI_ISL_2122903, EPI_ISL_2122913, EPI_ISL_2152801, EPI_ISL_2236110, EPI_ISL_2237466, EPI_ISL_2238168, EPI_ISL_2318468, EPI_ISL_2318717, EPI_ISL_2353957, EPI_ISL_2353975, EPI_ISL_2354089, EPI_ISL_2354307, EPI_ISL_2354415, EPI_ISL_2395377, EPI_ISL_2395933, EPI_ISL_2395950, EPI_ISL_2396605, EPI_ISL_2396746, EPI_ISL_2406693, EPI_ISL_2406838, EPI_ISL_2407531, EPI_ISL_2407738, EPI_ISL_2407861, EPI_ISL_2435330, EPI_ISL_2436848, EPI_ISL_2437430, EPI_ISL_2437635, EPI_ISL_2438407, EPI_ISL_2438479, EPI_ISL_2438483, EPI_ISL_2457768, EPI_ISL_2457790, EPI_ISL_2457880, EPI_ISL_2457967, EPI_ISL_2486027, EPI_ISL_2487320, EPI_ISL_2487391, EPI_ISL_2487450, EPI_ISL_2487610, EPI_ISL_2488124, EPI_ISL_2488222, EPI_ISL_2488391, EPI_ISL_2542015, EPI_ISL_2542155, EPI_ISL_2542227, EPI_ISL_2543114, EPI_ISL_2543354, EPI_ISL_2543364, EPI_ISL_2543369, EPI_ISL_2553162, EPI_ISL_2554374, EPI_ISL_2554923, EPI_ISL_2555058, EPI_ISL_2555065, EPI_ISL_2555103, EPI_ISL_2555231, EPI_ISL_2555276, EPI_ISL_2555390, EPI_ISL_2607714, EPI_ISL_2607778, EPI_ISL_2607866, EPI_ISL_2607867, EPI_ISL_2607870, EPI_ISL_2607872, EPI_ISL_2607945, EPI_ISL_2607989, EPI_ISL_2608090, EPI_ISL_2608236, EPI_ISL_2608249, EPI_ISL_2640028, EPI_ISL_2640034, EPI_ISL_2640075, EPI_ISL_2640219, EPI_ISL_2641324, EPI_ISL_2651642, EPI_ISL_2651748, EPI_ISL_2651757, EPI_ISL_2651877, EPI_ISL_2681932, EPI_ISL_2681948, EPI_ISL_2681985, EPI_ISL_2682769, EPI_ISL_2683124, EPI_ISL_2683168, EPI_ISL_2683169, EPI_ISL_2683241, EPI_ISL_2683316, EPI_ISL_2683435, EPI_ISL_2699526, EPI_ISL_2700416, EPI_ISL_2700506, EPI_ISL_2701346, EPI_ISL_2701688, EPI_ISL_2701849, EPI_ISL_2702106, EPI_ISL_2704620, EPI_ISL_2704636, EPI_ISL_2730642, EPI_ISL_2730746, EPI_ISL_2730811, EPI_ISL_2730826, EPI_ISL_2730832, EPI_ISL_2730873, EPI_ISL_2730979, EPI_ISL_2731005, EPI_ISL_2731147, EPI_ISL_2731172, EPI_ISL_2731189			
see above	Lighthouse Lab in Glasgow	Wellcome Sanger Institute for the COVID-19 Genomics UK (COG-UK) Consortium	Anna Dominiczak and Alex Alderton; Carol Clugston; Cordelia Langford; David Gray; David K. Jackson; Dominic Kwiatkowski; Ewan Harrison; Harper VanSteenhouse; Ian Johnston; Jeffrey Barrett; John Sillitoe on behalf of the Wellcome Sanger Institute COVID-19 Surveillance Team; Roberto Amato; Sonia Goncalves; Yumi Kasai
EPI_ISL_533151, EPI_ISL_600469	Lighthouse Lab in Glasgow	Wellcome Sanger Institute for the COVID-19 Genomics UK (COG-UK) consortium	Anna Dominiczak and Alex Alderton; Carol Clugston; Cordelia Langford; David Gray; David K. Jackson; Dominic Kwiatkowski; Ewan Harrison; Harper VanSteenhouse; Ian Johnston; John Sillitoe; John Sillitoe on behalf of the Wellcome Sanger Institute COVID-19 Surveillance Team (http://www.sanger.ac.uk/covid-team); Roberto Amato; Sonia Goncalves; Yumi Kasai
EPI_ISL_1806897, EPI_ISL_1806899, EPI_ISL_1806900, EPI_ISL_1806901, EPI_ISL_1806902, EPI_ISL_1806903, EPI_ISL_1806905, EPI_ISL_1829218, EPI_ISL_1829280, EPI_ISL_1829449, EPI_ISL_1831275, EPI_ISL_1912752, EPI_ISL_1912753, EPI_ISL_1912757, EPI_ISL_1912758, EPI_ISL_1939714, EPI_ISL_1985832, EPI_ISL_1986101, EPI_ISL_2006668, EPI_ISL_2022513, EPI_ISL_2022526, EPI_ISL_2023030, EPI_ISL_2023031, EPI_ISL_2091666, EPI_ISL_2199981, EPI_ISL_2199982, EPI_ISL_2254122			
see above	Lighthouse Lab in Milton Keynes	Wellcome Sanger Institute for the COVID-19 Genomics UK (COG-UK) Consortium	Cordelia Langford; David K. Jackson; Dominic Kwiatkowski; Ewan Harrison; Ian Johnston; Jeffrey Barrett; John Sillitoe on behalf of the Wellcome Sanger Institute COVID-19 Surveillance Team; Roberto Amato; Sonia Goncalves; The Lighthouse Lab in Milton Keynes and Alex Alderton
EPI_ISL_1908494, EPI_ISL_1936188, EPI_ISL_2598080	MD PHL	MD PHL	Maryland Department of Health Laboratories Administration
EPI_ISL_1447036	MI - Michigan Department of Health and Human Services - Bureau of Laboratories	Centers for Disease Control and Prevention Division of Viral Diseases, Pathogen Discovery	Alison Laufer Halpin; Ben L. Rambo-Martin; Clinton R. Paden; Dakota Howard; Darlene Wagner; Dave Wentworth; Dhwani Batra; Jasmine Padilla; Justin Lee; Katie Dillon; Krista Queen; Kristen Knipe; Kristine Lacek; Mark Burroughs; Matthew Schmerer; Mili Sheth; Peter Cook; Sam Shepard; Sarah Nobles; Shoshona Le; Suxiang Tong; Vivien Dugan; Yvette Unoarumhi
EPI_ISL_1373999	MO State Public Health Laboratory	Pathogen Discovery, Respiratory Viruses Branch, Division of Viral Diseases, Centers for Disease Control and Prevention	Anna Kelleher; Anna Uehara; Brian Lynch; Clinton R. Paden; Haibin Wang; Han Jia Justin Ng; Jing Zhang; Krista Queen; Peter Cook; Suxiang Tong; Yan Li; Ying Tao
EPI_ISL_2346381, EPI_ISL_2346386, EPI_ISL_2346388, EPI_ISL_2346402, EPI_ISL_2346409, EPI_ISL_2346415, EPI_ISL_2346419, EPI_ISL_2346420, EPI_ISL_2346422, EPI_ISL_2346427, EPI_ISL_2346428, EPI_ISL_2346434			
see above	MRC/UVRI & LSHTM Uganda Research Unit, Central Public Health Laboratories	MRC/UVRI & LSHTM Uganda Research Unit, Central Public Health Laboratories	Dan Lule Bugembe; Isaac Sseeewanyana; Matthew Cotten; My V.T. Phan; Patrick Semanda; Pontiano Kaleebu; Susan Nabadda
EPI_ISL_1970565, EPI_ISL_1970566, EPI_ISL_1970567, EPI_ISL_1970568	MRC/UVRI & LSHTM Uganda Research Unit	MRC/UVRI & LSHTM Uganda Research Unit	Dan Lule Bugembe; Isaac Sseeewanyana; Matthew Cotten; My V.T. Phan; Patrick Semanda; Pontiano Kaleebu; Susan Nabadda
EPI_ISL_1469351, EPI_ISL_1469365, EPI_ISL_1469379	MRC/UVRI & LSHTM Uganda Research Unit	Where sequence data have been generated and submitted to GISAID	Dan Lule Bugembe; Isaac Sseeewanyana; Matthew Cotten; My V.T. Phan; Patrick Semanda; Pontiano Kaleebu; Susan Nabadda
EPI_ISL_2348511	MRC/UVRI & LSHTM Uganda Research Unit, Central Public Health Laboratories	MRC/UVRI & LSHTM Uganda Research Unit, Central Public Health Laboratories	Dan Lule Bugembe; Isaac Sseeewanyana; Matthew Cotten; My V.T. Phan; Patrick Semanda; Pontiano Kaleebu; Susan Nabadda
EPI_ISL_2690505	MRC/UVRI & LSHTM Uganda Research Unit, Central Public Health Laboratories, Rakai Health Sciences Program	MRC/UVRI & LSHTM Uganda Research Unit, Central Public Health Laboratories	Dan Lule Bugembe; Hellen Nansumba; Isaac Sseeewanyana; Matthew Cotten; My V.T. Phan; Patrick Semanda; Pontiano Kaleebu; Susan Nabadda
EPI_ISL_2290768, EPI_ISL_2290769	MSHS Clinical Microbiology Laboratories	MSHS Pathogen Surveillance Program	Adolfo García-Sastre; Adriana van de Guchte; Ajay Obla; Alberto Paniz-Mondolfi; Ana S. Gonzalez-Reiche; Angela Amoako; Ashley Salimbangon; Betsaida Salom Melo; Bremy Alburquerque; Brianne Ciferri; Charles Gleason; Daniel Floda; Deena R. Altman; Denise Jurczyszak; Emilia Mia Sordillo; Gintaras Deikus; Giulio Kleiner; Gopi Patel; Hala Alshammary; Harm van Bakel; Irina Oussenko; Jayeeta Dutta; Juan Soto; Julia Matthews; Katherine Beach; Kathryn Twyman; Kayla Russo; Komal Srivastava; Levy Sominsky; Mahmoud Awawda; Marta Luksza; Matthew M. Hernandez; Melissa Gitman; Michael D. Nowak; Mitchell J. Sullivan; Nancy Francoeur; Robert Sebra; Sarah Schaefer; Shelcie Fabre; Shwetha Hara Sridhar; Viviana Simon; Ying-Chih Wang; Zenab Khan
EPI_ISL_1446480, EPI_ISL_2787805	MT Public Health Laboratory	Centers for Disease Control and Prevention Division of Viral Diseases, Pathogen Discovery	Alison Laufer Halpin; Ben L. Rambo-Martin; Clinton R. Paden; Dakota Howard; Darlene Wagner; Dave Wentworth; Dhwani Batra; Jasmine Padilla; Justin Lee; Katie Dillon; Krista Queen; Kristen Knipe; Kristine Lacek; Mark Burroughs; Matthew Schmerer; Mili Sheth; Peter Cook; Sam Shepard; Sarah Nobles; Shoshona Le; Suxiang Tong; Vivien Dugan; Yvette Unoarumhi
EPI_ISL_1482810, EPI_ISL_1482857, EPI_ISL_1482906	MUSC Molecular Pathology Laboratory	MUSC Molecular Pathology Laboratory	Dariusz Pytel; Frederick S. Nolte; Jaclyn Dunne; Julie W. Hirschhorn; Kristen Maurer; W. Bailey Glen Jr
EPI_ISL_648548	Madera County Department of Public Health	Chan-Zuckerberg Biohub	CZB Cliahub Consortium
EPI_ISL_1372628	Maine Health and Environmental Testing Laboratory	Tewhey Lab, The Jackson Laboratory	Barter, M.; Dewey, H.; H. and Tewhey, R.; Iosue, F.; Lynch, R.; Matluk, N.; Munger
EPI_ISL_2646554, EPI_ISL_2647882, EPI_ISL_2786052, EPI_ISL_2786288, EPI_ISL_2786444, EPI_ISL_2786497	Mako Medical	Centers for Disease Control and Prevention Division of Viral Diseases, Pathogen Discovery	Adrian Paskey; Benjamin Rambo-Martin; Christopher Gulvick; Clinton R. Paden; Dakota Howard; Darlene Wagner; Dhwani Batra; Duncan MacCannell; Jason Caravas; Kara Moser; Lauren Moon; Matthew Schmerer; Matthew Tugwell; Peter W. Cook; Scott Sammons; Shatavia Morrison; Yvette Unoarumhi
EPI_ISL_528743	Malaysia Genome Institute	Malaysia Genome Institute	Azrin Ahmad; Enizza Kasim; Irni Suhayu Sapian; Mohd Faizal Abu Bakar; Mohd Noor Mat Isa; Nor Azfa Johari; Nurhezreen Md Iqbal; Shahrul Hisham Zainal Ariffin; Shamsidar Sopie; Siti Noraini Othman; Yusuf Muhammad Noor
EPI_ISL_1805093, EPI_ISL_2096213, EPI_ISL_2102347, EPI_ISL_2161520, EPI_ISL_2161528, EPI_ISL_2319503, EPI_ISL_2400474, EPI_ISL_2400478, EPI_ISL_2400492			
see above	Maryland Genomics, Institute for Genome Sciences, University of Maryland School of Medicine	Maryland Genomics, Institute for Genome Sciences, University of Maryland School of Medicine	Aditya; Claire M; Fraser; Holly; Humphrys; Jacques; Kranthi; Lisa D; Luke J; Mehta; Mike; Ott; Ravel; Roussey; Sadzewicz; Sandra; Tallon; Vavikolanu
EPI_ISL_765770, EPI_ISL_765805	Massachusetts General Hospital	Infectious Disease Program, Broad Institute of Harvard and MIT	A.E.; Adams, G.; Anahtar, M.; B.L.; B.W.; Bauer, M.; Birren; Branda, J.; Carter, A.; Cerrato, F.; Chaluvadi, S.; Chapman; Cusick, C.; D.J.; DeRuff, K.; E. and Sabeti; Flowers, K.; Gallagher, G.; Gladden-Young, A.; Gnirke, A.; Harris, J.; J.E.; K.J.; LaRocque, R.; Lagerborg, K.; Lemieux; Lin; Loreth, C.; MacInnis; Neumann, A.; Normandin, E.; P.C.; Park; Pierce, V.; Reilly, S.; Rosenberg; Rudy, M.; Ryan, E.; S.B.; Shaw, B.; Siddle; Slater, D.; Smole, S.; Tomkins-Tinch, C.; Turbett, S.
EPI_ISL_1233296, EPI_ISL_1503654, EPI_ISL_1527109, EPI_ISL_1624659, EPI_ISL_2008146, EPI_ISL_2008162, EPI_ISL_2008164, EPI_ISL_2008369, EPI_ISL_2229531, EPI_ISL_2688817			
see above	Massachusetts State Public Health Laboratory	Massachusetts State Public Health Laboratory	Andrew Lang; Glen Gallagher; Sandra Smole; Timelia Fink
EPI_ISL_456377, EPI_ISL_456378	MedLab Central Ltd	Institute of Environmental Science and Research (ESR)	Anja Werno; Antje van der Linden; Arlo Upton; Chris Mansell; David Hammer; Dragana Drinkovic; Erasmus Smit; Gary McAuliffe; Hana Sofia Andersson; James Ussher; Jill Sherwood; Joep de Ligt; Josh Freeman; Julia Howard; Juliet Elvy; Lauren Jelly; Mary DeAlmeida; Matt Blakiston; Matt Storey; Matthew Rogers; Max Bloomfield; Michael Addidle; Michelle Balm; Sally Roberts; Sarah Jefferies; Sharmini Muttaiyah; Susan Morpeth; Susan Taylor; Timothy Blackmore; Vani Sathyendran; Veronica Playle; Virginia Hope; Xiaoyun Ren
EPI_ISL_1508843, EPI_ISL_1508847	Medical Genetics Laboratory, Regional Centre of Medical Genetics, Emergency County Hospital Craiova	Medical Genetics Laboratory, Regional Centre of Medical Genetics, Emergency County Hospital Craiova	Adina Dragos; Ana-Maria Buga; Anca-Lelia (Riza) Costache; Andrei Pirvu; Elena Plesea; Ioana Streata; Mihai Cucu; Mihai Ioana; Monica Cara; Razvan Plesea; Stefania Dorobantu
EPI_ISL_2107512, EPI_ISL_2107524, EPI_ISL_2107525, EPI_ISL_2107526	Medical Laboratory Sciences, Arab American University	Medical Laboratory Sciences, Arab American University	Al-Jawabreh, A.; Al-Jawabreh, H.; Dumaidi, K.; Ereqat, S.; Nasereddin, A.
EPI_ISL_2450073, EPI_ISL_2483192	Medical Microbiology Unit, Department for Laboratory Medicine, Drammen Hospital, Vestre Viken Health Trust,	Norwegian Institute of Public Health, Department of Virology	Atiya R Ali; Debech Nadia; Engebretsen Serina Beate; Garcia Llorente Ignacio; Hilde Elshaug; Hilde Vollan; Jon Bråte; Kamilla Heddeland Instefjord; Karoline Bragstad; Kathrine Stene-Johansen; Line Victoria Moen; Marie Paulsen Madsen; Olav Hungnes; Pedersen Benedikte Nevjen; Rasmus Riis Kopperud
EPI_ISL_2102114	Medics Labor AG	Clinical Bacteriology	Adrian Egli; Alfredo Mari; Hans Hirsch; Helena MB Seth-Smith; Julia Bielicki; Karoline Leuzinger; Madlen Stange; Manuel Battegay; Tim Roloff
EPI_ISL_480336	Microbial Genomics Laboratory, Institut Pasteur de Montevideo	Microbial Genomics Laboratory, Institut Pasteur de Montevideo	Cecilia Salazar; Gonzalo Moratorio; Gregorio Iraola; Ignacio Ferrés; Marianoel Pereira; Pilar Moreno
EPI_ISL_2650303	Microbiology Department, Laboratori Clínic Metropolitana Nord. Hospital Universitari Germans Trias i Pujol	Can Ruti SARS-CoV-2 Sequencing Hub (HUGTiP/IrsiCaixa/IGTP)	Alba Sánchez; Anna Not; Antoni E Bordoy; Bonaventura Clotet; Cristina Casañ; Cristina Esteban; Francesc Catala-Moll; Gemma Clara; Ignacio Blanco; Marc Noguera-Julian; Maria Casadellà; Mariona Parera; Mercedes Guerrero; Montserrat Giménez; Pere-Joan Cardona; Pilar Armengol; Roger Paredes; Verónica Saludes; and Elisa Martró on behalf of the Can Ruti SARS-CoV-2 Sequencing Hub
EPI_ISL_1208559, EPI_ISL_1999791	Microbiology Department, Laboratori Clínic Metropolitana Nord. Hospital Universitari Germans Trias i Pujol.	Can Ruti SARS-CoV-2 Sequencing Hub (HUGTiP/IrsiCaixa/IGTP)	Alba Sánchez; Anna Not; Antoni E Bordoy; Bonaventura Clotet; Carol Galvez Maria Casadellà; Cristina Casañ; Cristina Esteban; Francesc Catala-Moll; Gemma Clara; Ignacio Blanco; Irina Pey; Jordi Barretina; Julia G Prado; Marc Noguera-Julian; Maria Casadellà; Mariona Parera; Mercedes Guerrero; Montserrat Giménez; Pere-Joan Cardona; Pilar Armengol; Roger Paredes; Verónica Saludes; and Elisa Martró on behalf of the Can Ruti SARS-CoV-2 Sequencing Hub.
EPI_ISL_456385, EPI_ISL_548132, EPI_ISL_548133, EPI_ISL_548144, EPI_ISL_548146, EPI_ISL_579102, EPI_ISL_622787, EPI_ISL_732968, EPI_ISL_755624, EPI_ISL_1082256, EPI_ISL_1082259, EPI_ISL_1082262, EPI_ISL_1113756, EPI_ISL_1113758, EPI_ISL_1315316, EPI_ISL_1315318, EPI_ISL_1469096, EPI_ISL_1469105, EPI_ISL_1621293, EPI_ISL_1967891, EPI_ISL_1967893, EPI_ISL_2406491			
see above	Middlemore Hospital	Institute of Environmental Science and Research (ESR)	Anja Werno; Antje van der Linden; Arlo Upton; Chris Mansell; David Hammer; Dragana Drinkovic; Erasmus Smit; Gary McAuliffe; Hana Sofia Andersson; Hermes Perez; James Ussher; Jill Sherwood; Jing Wang; Joep de Ligt; Josh Freeman; Julia Howard; Juliet Elvy; Lauren Jelly; Mary DeAlmeida; Matt Blakiston; Matt Storey; Matthew Rogers; Max Bloomfield; Michael Addidle; Michelle Balm; Muhammad Faisal; Nikki Freed; Olin Silander; Olivia Stroeven; Paula scholes; Rachel Boyle; Sally Roberts; SallyAnn Harbison; Sarah Jefferies; Sharmini Muttaiyah; Susan Lin; Susan Morpeth; Susan Taylor; Timothy Blackmore; Vani Sathyendran; Veronica Playle; Virginia Hope; Xiaoyun Ren
EPI_ISL_678279	Mikrobiologie, RARI	Mikrobiologie, RARI	A.V.; Badanin; D.V.; E.A.; Fedorov; Guseva; Krasnov; Kutyrev; N.A.; N.P.; Naryshkina; Portenko; S.A.; Sharapova; Shcherbakova; Sosedova; V.V.; Y.M.
EPI_ISL_2379972, EPI_ISL_2379983, EPI_ISL_2380007,	Ministry of Health Hospitals	Institute of Health and Community Medicine	Chan Chia Jui; Chua Hock Hin; David Perera; Ooi Mong How; Tonnii Sia Loong Loong; Wong Jyn Shan; Wong Kiing Aik

EPI_ISL_2535774, EPI_ISL_2535776			
EPI_ISL_1171908, EPI_ISL_1272166, EPI_ISL_1278224, EPI_ISL_1583169, EPI_ISL_1620528, EPI_ISL_1922101, EPI_ISL_2107322, EPI_ISL_2158049, EPI_ISL_2158057, EPI_ISL_2158065, EPI_ISL_2158066, EPI_ISL_2232626, EPI_ISL_2363624, EPI_ISL_2363755, EPI_ISL_2363758, EPI_ISL_2363775, EPI_ISL_2716318, EPI_ISL_2716319			
see above	Ministry of Health Turkey	Ministry of Health Turkey	Fatma Bayrakdar; Gulay Korukluoglu; Suleyman Yalcin; Yasemin Cosgun
EPI_ISL_1712690, EPI_ISL_1713201, EPI_ISL_1713310, EPI_ISL_1713462, EPI_ISL_1713623, EPI_ISL_1713637, EPI_ISL_1713663, EPI_ISL_1713673, EPI_ISL_1713677, EPI_ISL_1713690, EPI_ISL_1713714, EPI_ISL_1713728, EPI_ISL_1713751, EPI_ISL_1713767, EPI_ISL_2274323, EPI_ISL_2274379, EPI_ISL_2274381, EPI_ISL_2274383, EPI_ISL_2274397, EPI_ISL_2274399, EPI_ISL_2274404, EPI_ISL_2274410, EPI_ISL_2408244, EPI_ISL_2408315, EPI_ISL_2408316, EPI_ISL_2408321, EPI_ISL_2408324, EPI_ISL_2408352, EPI_ISL_2408358, EPI_ISL_2408359, EPI_ISL_2408360, EPI_ISL_2408364, EPI_ISL_2408368, EPI_ISL_2408383			
see above	Ministry of Public Health / Hamad Medical Corporation	Biomedical Research Center (BRC), Qatar University / Qatar Genome Project (QGP)	Asmaa A. Al-Thani. MOPH and HMC: Abdullatif Al-Khal; Asmaa A. Al-Thani. MOPH and HMC: Abdullatif Al-Khal; BRC: Fatiha M. Benslimane; Chadi Saad; Dana Al-Batesh; Dina Elgakhlab QGP: Fatima H. Al-Kuwari; Dina Elgakhlab QGP: Fatima H. Al-Kuwari; Einas A. E. Al-Kuwari; Hadi M. Yassine; Hamad E. Al-Romaihi; Hamda Alromaihi; Heba A. Al-Khatib; Mashael A. Al-Bader; Mohammed Al-Thani; Muna A. S. Al-Maslamani; Oal Al-Jamal; Peter V. Coyle; Reham A. El-Kahlout. QBB: Tasneem Al-Hamad; Reham A. El-Kahlout. QBB: Tasneem Al-Hamad; Roberto Bertollini; Salih Al-Marri
EPI_ISL_1714224, EPI_ISL_1714261, EPI_ISL_1714469, EPI_ISL_1714569	Ministry of Public Health / Hamad Medical Corporation	Weill Cornell Medical College - Qatar (WCM-Q), Genomics Core Laboratory / Qatar Genome Project (QGP)	Chadi Saad MOPH and HMC: Abdullatif Al-Khal; Dina Elgakhlab; Einas A. E. Al-Kuwari; Hamad E. Al-Romaihi; Hamda Alromaihi; Joel A Malek. QGP: Fatima H. Al-Kuwari; Laith Abu-Raddad; Mashael A. Al-Bader; Meryem Bensaad; Mohammed Al-Thani; Muna A. S. Al-Maslamani; Peter V. Coyle; Reham A. El-Kahlout. QBB: Tasneem Al-Hamad; Roberto Bertollini; Salih Al-Marri; Shameem Younuskunju; WCMQ: Ayeda A. Ahmed; Yasmin Mohamoud
EPI_ISL_1857556, EPI_ISL_1857569, EPI_ISL_2232545, EPI_ISL_2536900, EPI_ISL_2598148	Minnesota Department of Health, Public Health Laboratory	Minnesota Department of Health, Public Health Laboratory	Alexandra Lorentz; Jacob Garfin; Matt Plumb; and Xiong Wang
EPI_ISL_2614742	Miri Hospital	Institute for Medical Research, Infectious Disease Research Centre, National Institutes of Health, Ministry of Health Malaysia	Azizan MA; Kamel K; Mohd Zawawi Z; Ramly N; Robert F; Suppiah J; Thayan R
EPI_ISL_2035597, EPI_ISL_2753757, EPI_ISL_2753842	Missouri State Public Health Laboratory	Missouri State Public Health Laboratory	Ashley New; Joshua Barry; Matthew Sinn
EPI_ISL_1312791	Molecular & Genomic Pathology Laboratory, Thomas Jefferson University Hospital	Molecular & Genomic Pathology Laboratory, Thomas Jefferson University Hospital	Nitika Badjatia; Run Jin; Zi-xuan Wang
EPI_ISL_2095423, EPI_ISL_2095702	Molecular Diagnostics Mater Dei Hospital Pathology Department Malta	Molecular Diagnostics Mater Dei Hospital Pathology Department Malta	C Cilia; G Zahra; L Grech; R Borg
EPI_ISL_2006673, EPI_ISL_2101112, EPI_ISL_2101201, EPI_ISL_2101748, EPI_ISL_2102066, EPI_ISL_2105878, EPI_ISL_2105879, EPI_ISL_2105942, EPI_ISL_2105959, EPI_ISL_2107823, EPI_ISL_2107982, EPI_ISL_2113866			
see above	Molecular Diagnostics Pathology Department Mater Dei Hospital Malta	Molecular Diagnostics Pathology Department Mater Dei Hospital Malta	C Cilia; G Zahra; L Grech; R Borg
EPI_ISL_482716	Molecular Diagnostics Services (MDS)	KRISP, KZN Research Innovation and Sequencing Platform	Chimukangara B; Giandhari J; Khan S; Lessells R; Mdlalose K; Pillay S; Tegally H; Wilkinson E; York D; de Oliveira T
EPI_ISL_2031957	Molecular diagnostic laboratory of Federal Budget Institution of Science "Central Research Institute of Epidemiology" of The Federal Service on Customers' Rights Protection and Human Well-being Surveillance	Group of Genomics and Postgenomic Technologies of Central Research Institute of Epidemiology	Akimkin V.G.; Kaptelova V.V.; Kondrasheva L.Y.; Korneenko E.V.; Nadtoka M.I.; Saenko S.S.; Samojlov A.E.; Shipulina O.Y.; Sinicyn S.O.; Smirnova Y.S.; Speranskaya A.S.; Tivanova E.V.
EPI_ISL_2626269, EPI_ISL_2626279, EPI_ISL_2626319, EPI_ISL_2626324, EPI_ISL_2626341, EPI_ISL_2626392, EPI_ISL_2626429			
see above	Molecular diagnostic laboratory of Federal Budget Institution of Science "Central Research Institute of Epidemiology" of The Federal Service on Customers' Rights Protection and Human Well-being Surveillance	Group of Genomics and Postgenomic Technologies of Central Research Institute of Epidemiology	Akimkin V.G.; Kaptelova V.V.; Kondrasheva L.Y.; Korneenko E.V.; Nadtoka M.I.; Saenko S.S.; Samoilov A.E.; Sinitsyn S.O.; Smirnova Y.S.; Speranskaya A.S.; Tivanova E.V.
EPI_ISL_2350979	Mongkutwattana General Hospital	Division of Genomic Medicine and Innovation support,Department of Medical Sciences, Ministry of Public Health, Thailand	Archawin Rojanawiwat; Jirapha Pakdee; Natthakul Bunneang; Nuanjun Wichukchinda; Penpitcha Thawong; Pilailuk Akkapaiboon Okada; Pundharika Piboonsiri; Surakameth Mahasirimongkol; Waritta Sawaengdee
EPI_ISL_2023368, EPI_ISL_2604223, EPI_ISL_2604228	Montana Public Health Laboratory	Montana Public Health Laboratory	Carrie Biskupiak; Deborah Gibson; Joy Ritter; Michael Dills; Michelle Mozer
EPI_ISL_1017573	Murphy Medical Associates	Grubaugh Lab - Yale School of Public Health	Annie Watkins; Caleb Neal; Chantal Vogels; Joseph Fauver; Mallery Breban; Mary Petrone; Nathan Grubaugh; Steven Murphy; Tara Alpert
EPI_ISL_1591094, EPI_ISL_1591097	NAMRU-6	Pathogen Discovery, Respiratory Viruses Branch, Division of Viral Diseases, Centers for Disease Control and Prevention	Adam Retchless; Anna Kelleher; Anna Montmayeur; Anna Uehara; Brian Lynch; Clinton R. Paden; Haibin Wang; Han Jia Justin Ng; Jing Zhang; Justin Lee; Krista Queen; Mark Burroughs; Peter Cook; Rachel Marine; Suxiang Tong; Yan Li; Ying Tao
EPI_ISL_1678360	NC State Laboratory of Public Health	Centers for Disease Control and Prevention Division of Viral Diseases, Pathogen Discovery	Alison Laufer Halpin; Ben L. Rambo-Martin; Clinton R. Paden; Dakota Howard; Darlene Wagner; Dave Wentworth; Dhwani Batra; Jasmine Padilla; Justin Lee; Katie Dillon; Krista Queen; Kristen Knipe; Kristine Lacek; Mark Burroughs; Matthew Schmerer; Mili Sheth; Peter Cook; Sam Shepard; Sarah Nobles; Shoshona Le; Suxiang Tong; Vivien Dugan; Yvette Unoarumhi
EPI_ISL_1036244	NCSLPH	NCSLPH	Chase K; Glover W; Greene S; Miller MC
EPI_ISL_1937844	NH Dept. of Health and Human Services Public Health Labs	Centers for Disease Control and Prevention Division of Viral Diseases, Pathogen Discovery	Alison Laufer Halpin; Ben L. Rambo-Martin; Clinton R. Paden; Dakota Howard; Darlene Wagner; Dave Wentworth; Dhwani Batra; Jasmine Padilla; Justin Lee; Katie Dillon; Krista Queen; Kristen Knipe; Kristine Lacek; Mark Burroughs; Matthew Schmerer; Mili Sheth; Peter Cook; Sam Shepard; Sarah Nobles; Shoshona Le; Suxiang Tong; Vivien Dugan; Yvette Unoarumhi
EPI_ISL_751712	NH Dept. of Health and Human Services Public Health Labs	Genomics and Discovery, Respiratory Viruses Branch, Division of Viral Diseases, Centers for Disease Control and Prevention	Anna Montmayeur; Anna Uehara; Clinton R. Paden; Haibin Wang; Jing Zhang; Justin Lee; Krista Queen; Mili Sheth; Peter W. Cook; Rachel Marine; Suxiang Tong; Yan Li; Ying Tao
EPI_ISL_2710253, EPI_ISL_2710259, EPI_ISL_2770461	NHLS Charlotte Maxeke Johannesburg Academic Hospital and the University of the Witwatersrand	KRISP, KZN Research Innovation and Sequencing Platform	Bulelani Manene; Florette Treurnicht; Giandhari Jennifer; Kathleen Subramoney; Naidoo Yeshnee; Pillay Sureshnee; San James; Tegally Houriiyah; Tshabuila Derek; Wilkinson Eduan; Yajna Ramphal; de Oliveira Tulio
EPI_ISL_912456	NHLS Universitas Academic	UFS Virology	D Goedhals; MM Nyaga; MT Mogotsi; P Nthiga; PA Bester; T de Oliveira
EPI_ISL_2802196	NHLS/UCT	NHLS/UCT	Arash Iranzadeh; Bruna Galvao; Carolyn Williamson; Deelan Doolabh; Diana Hardie; Gert Marais; Innocent Mudau; Lynn Tyers; Marvin Hsiao; Stephen Korsman
EPI_ISL_2494449	NHLS_VIRO	KRISP, KZn Research Innovation and Sequencing Platform	Baillie Vicky; Giandhari Jennifer; Madhi Shabir; Naidoo Yeshnee; Pillay Sureshnee; San James; Tegally Houriiyah; Tshabuila Derek; Wilkinson Eduan; de Oliveira Tulio; du Plessis Jeanine
EPI_ISL_1060793	NJ Rapid Response Teams	New Jersey Public Health Environmental Laboratories (NJ_PHEL)	Byeong Jeong; Dana Woell; Lindsey Bodnar; Shiv Verma
EPI_ISL_1960012, EPI_ISL_1969044, EPI_ISL_2185596, EPI_ISL_2281845, EPI_ISL_2282038, EPI_ISL_2282040, EPI_ISL_2376100, EPI_ISL_2727592			
see above	NJDOH, Public Health and Environmental Laboratories	NJ_PHEL	Allison Roder; Byeong Jeong; Dana Woell; Jacquelyn Deverell; Lindsey Bodnar; Matthew Scarnati; Ryan Pachucki; Shiv K. Verma
EPI_ISL_2018180	NM Dept. Health, Scientific Laboratory Division	Centers for Disease Control and Prevention Division of Viral Diseases, Pathogen Discovery	Alison Laufer Halpin; Ben L. Rambo-Martin; Clinton R. Paden; Dakota Howard; Darlene Wagner; Dave Wentworth; Dhwani Batra; Jasmine Padilla; Justin Lee; Katie Dillon; Krista Queen; Kristen Knipe; Kristine Lacek; Mark Burroughs; Matthew Schmerer; Mili Sheth; Peter Cook; Sam Shepard; Sarah Nobles; Shoshona Le; Suxiang Tong; Vivien Dugan; Yvette Unoarumhi
EPI_ISL_1482556, EPI_ISL_1671604	NORTHWELL HEALTH LABORATORIES	Wadsworth Center, New York State Department of Health	Alexis Russell; Catharine Prussing; Daryl M. Lamson; Erasmus Schneider; Erica Lasek-Nesselquist; John Kelly; Jonathan Plitnick; Kirsten St. George; Matthew Shudt; Melissa A Leisner; Navjot Singh
EPI_ISL_632312	NU-sjukvården	Clinical microbiology, Sahlgrenska University Hospital	Hedvig Engström Jakobsson; Johan Ringlander; Josefin Olausson; Magnus Lindh
EPI_ISL_1678079, EPI_ISL_1823954	NYC Department of Health and Mental Hygiene	Centers for Disease Control and Prevention Division of Viral Diseases, Pathogen Discovery	Alison Laufer Halpin; Ben L. Rambo-Martin; Clinton R. Paden; Dakota Howard; Darlene Wagner; Dave Wentworth; Dhwani Batra; Jasmine Padilla; Justin Lee; Katie Dillon; Krista Queen; Kristen Knipe; Kristine Lacek; Mark Burroughs; Matthew Schmerer; Mili Sheth; Peter Cook; Sam Shepard; Sarah Nobles; Shoshona Le; Suxiang Tong; Vivien Dugan; Yvette Unoarumhi
EPI_ISL_1398304	NYC Pandemic Response Lab	Wadsworth Center, New York State Department of Health	Alexis Russel; Daryl M. Lamson; Erasmus Schneider; Erica Lasek-Nesselquist; John Kelly; Jonathan Plitnick; Kirsten St. George; Matthew Shudt; Melissa A Leisner; Navjot Singh
EPI_ISL_1790019, EPI_ISL_1790023, EPI_ISL_1922839	NYU Langone Health	Departments of Pathology and Medicine, New York University School of Medicine	Adriana Heguy; Christian Marier; Dacia Dimartino; Emily Guzman; Gael Westby; Guiqing Wang; Paolo Cotzia; Paul Zappile; Peter Meyn; Sitharam Ramaswami; Yutong Zhang
EPI_ISL_625581	NaN	Chan-Zuckerberg Biohub	CZB Cliahub Consortium
EPI_ISL_1298480	Nadvirna CRH	The Institute of Molecular Biology and Genetics of NASU	M.Tukalo et al.
EPI_ISL_1805651, EPI_ISL_1805717, EPI_ISL_1805789, EPI_ISL_1805933, EPI_ISL_1805957, EPI_ISL_1805958, EPI_ISL_1805960, EPI_ISL_1805961, EPI_ISL_1805962			
see above	National Center for Communicable Diseases (NCCD) National Influenza Center	National Center for Communicable Diseases (NCCD) National Influenza Center	Ankhbayar S; Battur L; Bayasgalan N; Darmaa B; Hideka M; Khishigmunkh Ch; Mina N; Naranzul Ts; Nymadawa P; Seiichiro F; Shinji W; Tsogzolmaa G
EPI_ISL_1805697	National Center for Communicable Diseases (NCCD) National Influenza Center	National Centre for Disease Control (NCDC) National Influenza Center	Ankhbayar S; Battur L; Bayasgalan N; Darmaa B; Hideka M; Khishigmunkh Ch; Mina N; Naranzul Ts; Nymadawa P; Seiichiro F; Shinji W; Tsogzolmaa G
EPI_ISL_1407199, EPI_ISL_1407245, EPI_ISL_1407253, EPI_ISL_2494937, EPI_ISL_2609599,	National HIV Reference Laboratory, Ministry of Health, Public Health Institute of Malawi	KRISP, KZN Research Innovation and Sequencing Platform	Auld A; Chilima B; Chiwaula M; Emmanuel SJ; Giandhari J; Kaba M; Kampira E; Kasambara W; Kim L; Lessells R; Maida A; Mvula B; Mwangomba W; Naidoo Y; Panja L; Pillay S; Tegally H; Wadonda N; Wilkinson E; de Oliveira T

EPI_ISL_2609601			
EPI_ISL_2494886, EPI_ISL_2495053	National Health Laboratory Service, South Africa	KRISP, KZN Research Innovation and Sequencing Platform	Emmanuel SJ; Giandhari J; Khan S; Lessells R; Mdlalose K; Naidoo Y; Pillay S; Ramphal U; Tegally H; Wilkinson E; York D; de Oliveira T
EPI_ISL_1371929, EPI_ISL_2617216	National Health Laboratory Service, South Africa	KRISP, KZn Research Innovation and Sequencing Platform	Emmanuel SJ; Giandhari J; Giandhari Jennifer; Khan S; Lessells R; Maslo C; Mdlalose K; Naidoo Yeshnee; Pillay S; Pillay Sureshnee; San James; Sitharam L; Tegally H; Tegally Houriiyah; Tshabuila Derek; Wilkinson E; Wilkinson Eduan; Yajna Ramphal; York D; de Oliveira T; de Oliveira Tulio
EPI_ISL_456608	National Health Laboratory, Timor-Leste	Microbiological Diagnostic Unit Public Health Laboratory, The Peter Doherty Institute for Infection and Immunity	Antonia da Costa, E.; Baird, R.; Barreto, I.; Caly, L.; Canisia, D.; Dakh, F.; Dolores de Jesus da Costa, M.; Douglas, N.; Francis, J.; Freeman, K.; Jayanti Pereira Tilman, A.; Marr, I.; Sait, M.; Salles de Sousa, A.; Schultz, M.; Seemann, T.; Sherry, N.; Soares da Silva, E.; Wapling, J.; Ximenes, J.
EPI_ISL_2455483, EPI_ISL_2455493, EPI_ISL_2455494, EPI_ISL_2455495, EPI_ISL_2455496, EPI_ISL_2455497, EPI_ISL_2455506, EPI_ISL_2455895, EPI_ISL_2457173			
see above	National Hospital for Tropical Diseases	Oxford University Clinical Research Unit, Hanoi, Vietnam	H.Rogier van Doorn on behalf of the OUCRU COVID-19 research group; Le Van Duyet; Nguyen Thi Hong Thuong; Nguyen Thi Kim Chi; Nguyen Thi Tam; Nguyen Thu Trang; Pham Ngoc Thach; Phan Manh Cuong; Thomas Kesteman; Van Dinh Trang
EPI_ISL_416427, EPI_ISL_416429	National Influenza Center, National Institute of Hygiene and Epidemiology (NIHE)	National Influenza Center, National Institute of Hygiene and Epidemiology (NIHE)	Dang Duc Anh; Futoshi Hasebe; Hoang Vu Mai Phuong; Kouichi Morita; Le Quynh Mai; Le Thi Thanh; Meng Ling Moi; Nguyen Le Khanh Hang; Nguyen Phuong Anh; Nguyen Vu Son; Pham Hong Quynh Anh; Pham Thi Hien; Taichiro Takemura; Takeshi Nabeshima; Tran Thu Huong; Ung Thi Hong Trang; Vuong Duc Cuong
EPI_ISL_410301	National Influenza Centre, National Public Health Laboratory, Kathmandu, Nepal	The University of Hong Kong	Alfonso J. Rodriguez-Morales; Anup Bastola; Basu Dev Pandey; Bibek Kumar Lal; Daniel Chu; Haogao Gu; Leo Poon; Malik Peiris; Ranjit Sah; Runa Jha
EPI_ISL_402125	National Institute for Communicable Disease Control and Prevention (ICDC) Chinese Center for Disease Control and Prevention (China CDC)	National Institute for Communicable Disease Control and Prevention (ICDC) Chinese Center for Disease Control and Prevention (China CDC)	Chen; Dai; F.-H.; Hu, Y.; J.-H.; J.-J.; J.-L. and Zhu; Liu, Y.; Pei; Q.-M.; She; Song; T.-Y.; Tao; Tian; Wang; Wang, W.; Wu, F.; Xu, L.; Y.-L.; Y.-M.; Y.-Y.; Y.-Z.; Yu, B.; Z.-G.; Z.-W.; Zhang; Zhao, S.; Zheng
EPI_ISL_490257, EPI_ISL_2444003	National Institute for Communicable Diseases of the National Health Laboratory Service	National Institute for Communicable Diseases of the National Health Laboratory Service	Allam M; Amoako DG; Bhiman JN; Ismail A; Khumalo Z; Kwenda S; Mahlangu B; Mnyameni F; Mohale T; Mtshali P; Ntuli N; Scheepers C; Subramoney K
EPI_ISL_591278, EPI_ISL_2170893	National Institute for Viral Disease Control and Prevention, China CDC	National Institute for Viral Disease Control and Prevention, China CDC	; 10; 4&; 7; Baoying Huang3; Cao Chen; Cao Chen3&; Dayan Wang; Dayan Wang3; Dongyan Wang3; Fengqin Li6; George F.Gao; George Fu Gao1; Haibo Sun5; Hong Wang; Hong Wang3; Huilai Ma; Huilai Ma1&; Ji Wang; Ji Wang3&; Jian Cai1; Jianqun Zhang2&; Jianxing Yu1; Jingdong Song; Jun Han; Jun Meng2; Li Bai6; Li Zhao3; Liang Wang1; Lingling Mao5; Mingchun Luan2; Naiying Mao3; Ning Li6; Peihua Niu3; Qian Yang3; Ruqin Gao; Shaofeng Jiang9; Shihong Yang2; Shuangli Zhu3; Tao Ma1; Tianjiao Ji3; Wei Yao2*; Wenbo Xu; Wenbo Xu3*; Wenjie Tan3; Wenqing Yao5*; Xiang Ren1; Xiang Zhao; Xiang Zhao3; Yan Zhang3; Yang Song3&; Yanhai Wang3; Yao Meng; Yecheng Yao11; Ying Qin1&; Yingwei Sun5; Yong Zhang; Yong Zhang3; Yuchao Wu; Yunting Xia8; Zhaoguo Wang; Zhen Zhu3; Zhijian Bo2; Zhixiao Chen; Zhongjie Li1; Zijian Feng1*
EPI_ISL_2308255	National Institute of Health (NIH) - Federal Government of Somalia	African Centre of Excellence for Genomics of Infectious Diseases (ACEGID), Redeemer's University	A.T.; Abechi; Ajogbasile; Akano; C.A.; C.T.; Eromon; F.V.; Folarin, O.; Happi; I.B.; J.N.; J.U.; K.O.; Kayode; Nosamiefan, I.; Oguzie; Olawoye; Olumade; Oluniyi; P.E.; P.S.; T.J.; Ugwu; Uwanibe
EPI_ISL_875346, EPI_ISL_1116430, EPI_ISL_1116435, EPI_ISL_1116444, EPI_ISL_1116455, EPI_ISL_1186023, EPI_ISL_1186116, EPI_ISL_1279948, EPI_ISL_1279949, EPI_ISL_2099853, EPI_ISL_2190106, EPI_ISL_2566459, EPI_ISL_2566460			
see above	National Institute of Infectious Diseases-Prof. Dr. Matei Bals Molecular Diagnostics Laboratory	National Institute of Infectious Diseases-Prof. Dr. Matei Bals Molecular Diagnostics Laboratory	Andreea Tudor; Corina Casangiu; Dan Otelea; Leontina Banica; Marius Surleac; Ovidiu Vlaicu; Petre Milu; Robert Hohan; Simona Paraschiv
EPI_ISL_1091415	National Institute of Public Health - National Institute of Hygiene	1. National Institute of Public Health - National Institute of Hygiene; 2. Eurofins Genomics Europe Sequencing GmbH	ECDC COVID-19 WGS support team; Eurofins Genomics Europe Sequencing Team; Gierczyński Rafał; Sadkowska-Todys Małgorzata; Wołkowicz Tomasz; Zacharczuk Katarzyna
EPI_ISL_1826917	National Institute of Public Health - National Institute of Hygiene	1. Tricity SARS-CoV-2 sequencing consortium: University of Gdansk, Medical University of Gdansk, Vaxican Ltd., Invicta Ltd. 2. National Institute of Public Health - National Institute of Hygiene, Warsaw, Poland	Celina Cybulska; Karolina Gackowska; Katarzyna Groth; Katarzyna Zacharczuk; Krystyna Bienkowska Szewczyk; Lukasz Rabalski; Maciej Grzybek; Maciej Kosinski; Magdalena Nowakowska; Małgorzata Sadkowska-Todys; Tomasz Wołkowicz
EPI_ISL_1260750	National Laboratory for Health, Environment and Food	Department for Public Health Microbiology Ljubljana, National Laboratory for Health, Environment and Food	Jose Goncalves; Katarina Prosenc Trilar; Marija Trkov; Martin Bosilj; Metka Paragi; Tom Koritnik; Verica Mioc
EPI_ISL_2273628	National Laboratory for Health, Environment and Food, OMM, Celje	NLZOH (National Laboratory for Health, Environment and Food) / CISLD (Clinical Institute of Special Laboratory Diagnostics), University Children's Hospital, University Medical Center Ljubljana	Aleksander Kocuvan; Aleksander Mahnic; Alenka Štorman; Ana Grom; Barbara Jenko Bizjan; Daša Kavka / Jernej Kovač; Kaja Tominc; Katarina Kozmos; Maja Rupnik; Marko Pokorn; Maruša Debeljak; Mateja Borinc; Maša Jarčič; Nika Gobec; Robert Šket; Sandra Janezic; Tadej Battelino; Tine Tesovnik; Tjasa Žohar Čretnik
EPI_ISL_1111965, EPI_ISL_1112006, EPI_ISL_1112093, EPI_ISL_1266335, EPI_ISL_1266586	National Laboratory for Health, Environment and Food, OMM, Koper	CISLD (Clinical Institute of Special Laboratory Diagnostics), University Children's Hospital, University Medical Center Ljubljana	Ana Grom; Barbara Jenko Bizjan; Jernej Kovač; Katarina Kozmos; Marko Pokorn; Maruša Debeljak; Robert Šket; Tadej Battelino; Tine Tesovnik
EPI_ISL_1625399, EPI_ISL_2463617	National Laboratory for Health, Environment and Food, OMM, Koper	NLZOH (National Laboratory for Health, Environment and Food) / CISLD (Clinical Institute of Special Laboratory Diagnostics), University Children's Hospital, University Medical Center Ljubljana	Aleksander Kocuvan; Aleksander Mahnic; Alenka Štorman; Ana Grom; Barbara Jenko Bizjan; Gašper Strugar; Kaja Tominc; Katarina Kozmos; Maja Rupnik; Marko Pokorn; Maruša Debeljak; Maša Jarčič; Mitja Rak / Jernej Kovač; Nika Gobec; Robert Šket; Sandra Janezic; Tadej Battelino; Tina Cvetković; Tine Tesovnik; Tjasa Žohar Čretnik
EPI_ISL_2463375	National Laboratory for Health, Environment and Food, OMM, Maribor	NLZOH (National Laboratory for Health, Environment and Food) / CISLD (Clinical Institute of Special Laboratory Diagnostics), University Children's Hospital, University Medical Center Ljubljana	Aleksander Kocuvan; Aleksander Mahnic; Alenka Štorman; Ana Grom; Andrej Golle / Jernej Kovač; Barbara Jenko Bizjan; Kaja Tominc; Katarina Kozmos; Maja Rupnik; Marko Pokorn; Maruša Debeljak; Maša Jarčič; Mojca Cimerman; Nika Gobec; Nika Volmajer; Robert Šket; Sandra Janezic; Tadej Battelino; Tine Tesovnik; Tjasa Žohar Čretnik
EPI_ISL_647978, EPI_ISL_1191782, EPI_ISL_1191783, EPI_ISL_1191819	National Microbiology Reference Laboratory	Quadram Institute Bioscience	Agnes Juru; Alexander Goredema; Ana-Victoria Gutierrez; Andrew J. Page; Andrew Tarupiwa; Barbra Murwira; Beuty Makamure; Charles Nyagupe; David Baker; Faustinos T Takawira; Gaetan Thilliez; Gemma Kay; Gibson Mhlanga; Hlanai Gumbo; Isaac Phiri; Justin O'Grady; Kenneth K Maeka; Leonardo de Oliveira Martins; Muchaneta Mugabe; Portia Manangazira; Raiva Simbi; Robert Kingsley; Sekesai Zinyowera; Tapfumanei Mashe; Tatenda Takawira; Thanh Le Viet
EPI_ISL_2492564, EPI_ISL_2492582, EPI_ISL_2492586, EPI_ISL_2492596, EPI_ISL_2492628, EPI_ISL_2492654, EPI_ISL_2492693, EPI_ISL_2492793, EPI_ISL_2492918			
see above	National Microbiology Reference Laboratory, Ministry of Health, Harare, Zimbabwe	CERI, Centre for Epidemic Response and Innvoation, Stellenbosch University and KRISP, KZN Research Innovation and Sequencing Platform, UKZN.	Agnes Juru; Air Comodor Dr J. Chimedza; Charles Nyagupe; Dr Raiva Simbi; Emmanuel SJ; Giandhari J; Hlanai Gumbo; Kenneth Maeka; Naidoo Y; Pillay S; Tapfumanei Mashe; Tatenda Takawira; Tegally H; Wilkinson E; de Oliveira T
EPI_ISL_2479956, EPI_ISL_2479957, EPI_ISL_2479966, EPI_ISL_2479968, EPI_ISL_2479972, EPI_ISL_2479975, EPI_ISL_2479976, EPI_ISL_2479979, EPI_ISL_2479981, EPI_ISL_2479982, EPI_ISL_2479983, EPI_ISL_2479984, EPI_ISL_2479985			
see above	National Public Health Laboartory	CSIR-Institute of Genomics and Integrative Biology	Jyoti Acharya; Lokbandhu Chaudhary; Priya Jha; Runa Jha; Suni Dangol
EPI_ISL_1972889, EPI_ISL_2001482	National Public Health Laboratory Malaysia	National Public Health Laboratory Malaysia	Aziyati O; Chong CK; Hani MH; Hannah PYP; JL Tan; Kamal HKZ; Mohd AY; Noorliza MN; Norazimah T Rehan SAB Selvanesan S; Norhayati R; NurulAina MCA; Nurulsyahida I; Ushananthiny R; W.NurAfiza WMA Zirwatul AA; YF Ngeow; Yukie C
EPI_ISL_2450770, EPI_ISL_2450790, EPI_ISL_2450791, EPI_ISL_2450793, EPI_ISL_2450794, EPI_ISL_2450796, EPI_ISL_2450798, EPI_ISL_2450803, EPI_ISL_2450809			
see above	National Public Health Laboratory, Ministry of Health, Ministry of Health, Republic of South Sudan	South Sudan Ministry of Health, WHO South Sudan, MRC/UVRI & LSHTM Uganda Research Unit	Abe G. Abias; Dan Lule Bugembe; Dennis Kenyi Lodiongo; James Ayei; John Rumunu; Joseph Francis Wamala; Juma John HM; Lul Lojok Deng; Matthew Cotten; My V.T. Phan; Pontiano Kaleebu; Richard Lino Loro Lako; Sudhir Bunga
EPI_ISL_443201, EPI_ISL_498582, EPI_ISL_596456, EPI_ISL_596477, EPI_ISL_626632, EPI_ISL_654811, EPI_ISL_693321, EPI_ISL_754105, EPI_ISL_833380, EPI_ISL_891244, EPI_ISL_1081930, EPI_ISL_1081947, EPI_ISL_1098834, EPI_ISL_1098837, EPI_ISL_1252448, EPI_ISL_1476995, EPI_ISL_1477018, EPI_ISL_1489726, EPI_ISL_1524792, EPI_ISL_1543981, EPI_ISL_1620131, EPI_ISL_1620148, EPI_ISL_1620158, EPI_ISL_1620159, EPI_ISL_1634428, EPI_ISL_1634431, EPI_ISL_1634433, EPI_ISL_1652113, EPI_ISL_1652114, EPI_ISL_1652117, EPI_ISL_1652118, EPI_ISL_1652119, EPI_ISL_1652120, EPI_ISL_1652122, EPI_ISL_1652123, EPI_ISL_1652532, EPI_ISL_1704830, EPI_ISL_1704831, EPI_ISL_1704832, EPI_ISL_1704833, EPI_ISL_1704834, EPI_ISL_1704835, EPI_ISL_1704836, EPI_ISL_1704837, EPI_ISL_1704838, EPI_ISL_1704839, EPI_ISL_1704840, EPI_ISL_1704841, EPI_ISL_1704842, EPI_ISL_1719885, EPI_ISL_1719886, EPI_ISL_1719887, EPI_ISL_1719888, EPI_ISL_1719889, EPI_ISL_1719890, EPI_ISL_1719891, EPI_ISL_1719892, EPI_ISL_1719893, EPI_ISL_1719894, EPI_ISL_1719895, EPI_ISL_1719896, EPI_ISL_1719897, EPI_ISL_1719898, EPI_ISL_1719899, EPI_ISL_1719900, EPI_ISL_1719904, EPI_ISL_1752677, EPI_ISL_1816935, EPI_ISL_1816944, EPI_ISL_1816945, EPI_ISL_1816946, EPI_ISL_1816947, EPI_ISL_1816948, EPI_ISL_1816949, EPI_ISL_1816950, EPI_ISL_1816951, EPI_ISL_1816952, EPI_ISL_1816953, EPI_ISL_1816954, EPI_ISL_1816955, EPI_ISL_1816956, EPI_ISL_1816957, EPI_ISL_1816958, EPI_ISL_1816959, EPI_ISL_1816960, EPI_ISL_1816961, EPI_ISL_1816962, EPI_ISL_1816963, EPI_ISL_1816964, EPI_ISL_1816965, EPI_ISL_1816966, EPI_ISL_1816967, EPI_ISL_1816968, EPI_ISL_1816969, EPI_ISL_1816970, EPI_ISL_1816971, EPI_ISL_1816972, EPI_ISL_1816973, EPI_ISL_1897601, EPI_ISL_1897602, EPI_ISL_1897603, EPI_ISL_1897604, EPI_ISL_1897605, EPI_ISL_1897606, EPI_ISL_1897607, EPI_ISL_1897608, EPI_ISL_1897609, EPI_ISL_1897610, EPI_ISL_1897611, EPI_ISL_1897612, EPI_ISL_1897613, EPI_ISL_1897614, EPI_ISL_1897615, EPI_ISL_1897616, EPI_ISL_1897617, EPI_ISL_1897618, EPI_ISL_1897619, EPI_ISL_1897620, EPI_ISL_1897621, EPI_ISL_1914639, EPI_ISL_1914640, EPI_ISL_1914641, EPI_ISL_1914642, EPI_ISL_1914643, EPI_ISL_1914644, EPI_ISL_2349711, EPI_ISL_2349712, EPI_ISL_2349717, EPI_ISL_2349723, EPI_ISL_2349727, EPI_ISL_2349730, EPI_ISL_2349735, EPI_ISL_2349736, EPI_ISL_2349737, EPI_ISL_2349738, EPI_ISL_2349741, EPI_ISL_2349742, EPI_ISL_2349743, EPI_ISL_2349745, EPI_ISL_2349746, EPI_ISL_2349748, EPI_ISL_2349752, EPI_ISL_2349753, EPI_ISL_2349755, EPI_ISL_2349759, EPI_ISL_2349761, EPI_ISL_2349765, EPI_ISL_2349766, EPI_ISL_2349767, EPI_ISL_2349791, EPI_ISL_2349832, EPI_ISL_2349870, EPI_ISL_2508853, EPI_ISL_2508863, EPI_ISL_2509047, EPI_ISL_2509095, EPI_ISL_2621888, EPI_ISL_2621927, EPI_ISL_2621946, EPI_ISL_2621961			
see above	National Public Health Laboratory, National Centre for Infectious Diseases	National Public Health Laboratory, National Centre for Infectious Diseases	Chavatte JM; Chavatte Jean-Marc; Cui L; Cui Lin; Grace Jie Yin Ngan; Lin Cui; Lin RTP; Lin Raymond Tzer Pin; Mak TM; Mak Tze Minn; Octavia S; Octavia Sophie; Raymond Tzer Pin Lin; Royce Ang; Sophie Octavia; Tze Minn Mak; Zhenyang Zhou; Zhou Z
EPI_ISL_1911215	Naval Infectious Diseases Diagnostic Laboratory	Naval Medical Research Center Biological Defense Research Directorate	Andrea Luquette; Andrew Bennett; Bishwo Adhikari; Catherine Arnold; Francisco Malagon Bautista; Gregory Rice; Kimberly Bishop-Lilly; Kyle Long; Lindsay Glang; Logan Voegtly; Megan Schilling; Michael Deschenes; Regina Cer; Victor Sugiharto
EPI_ISL_2533882, EPI_ISL_2533883	Naval Medical Research Unit No. 3	Naval Medical Research Center Biological Defense Research Directorate	Andrea E. Luquette; Andrew J. Bennett; Bishwo N. Adhikari; Catherine E. Arnold; Chaselynn M. Watters; Emily K. Stefanov; Francisco Malagon; Kyle A. Long; Logan J. Voegtly; Luis A. Estrella; Michael V. Deschenes; Regina Z. Cer; Stephen M. Eggan; and Kimberly A. Bishop-Lilly
EPI_ISL_1169915, EPI_ISL_1626541, EPI_ISL_1648135	Nebraska Public Health Laboratory	NPHL COVID-19 Response Team	NPHL COVID-19 Response Team
EPI_ISL_475200	Nebraska Public Health Laboratory	UNMC COVID-19 Response Team	UNMC COVID-19 Response Team
EPI_ISL_1219819, EPI_ISL_1219842, EPI_ISL_1263138, EPI_ISL_2612502	Nevada State Public Health Laboratory	Nevada State Public Health Laboratory	Andrew Gorzalski; Mark Pandori
EPI_ISL_2598136	New Mexico Department of Health Scientific Laboratory	Center for Global Health, University of New Mexico Health Sciences Center	Anastacia Griego; Darrell Dinwiddie; Daryl Domman; Joseph Hicks; Kurt Schwalm; Michael Edwards; Twila Kunde; Valerie Morley
EPI_ISL_1256029, EPI_ISL_1805388	New Mexico Department of Health Scientific Laboratory	New Mexico Department of Health Scientific Laboratory	Anastacia Griego-Fisher; D'eldra Malone; Ellie Johnson; Jennifer Benoit; Linda Salazar; Ratheesh Rajan

EPI_ISL_1227165	New York Presbyterian Hospital	Wadsworth Center, New York State Department of Health	Alexis Russel; Daryl M. Lamson; Erasmus Schneider; Erica Lasek-Nesselquist; John Kelly; Jonathan Plitnick; Kirsten St. George; Matthew Shudt; Melissa A Leisner; Navjot Singh
EPI_ISL_2565897	Nigeria Centre for Disease Control (NCDC)	Africa Centre for Excellence for Genomics of Infectious Diseases (ACEGID), Redeemer's University	A.T.; Abechi; Ajogbasile; Akano; C.A.; C.T.; Eromon; F.V.; Folarin, O.; Happi; I.B.; J.N.; J.U.; K.O.; Kayode; Nosamiefan, I.; Oguzie; Olawoye; Olumade; Oluniyi; P.E.; P.S.; T.J.; Ugwu; Uwanibe
EPI_ISL_1970547, EPI_ISL_1970551	Nigeria Centre for Disease Control (NCDC)	African Centre for Excellence for Genomics of Infectious Diseases (ACEGID), Redeemer's University	A.T.; Abechi; Ajogbasile; Akano; C.A.; C.T.; Eromon; F.V.; Folarin, O.; Happi; I.B.; J.N.; J.U.; K.O.; Kayode; Oguzie; Olawoye; Olumade; Oluniyi; P.E.; P.S.; T.J.; Ugwu; Uwanibe
EPI_ISL_941277, EPI_ISL_1242017, EPI_ISL_1242018, EPI_ISL_1242019	Nigeria Centre for Disease Control (NCDC)	African Centre of Excellence for Genomics of Infectious Diseases (ACEGID), Redeemer's University	Oluniyi P.E. et al
EPI_ISL_455429, EPI_ISL_527882, EPI_ISL_729925, EPI_ISL_729985, EPI_ISL_729991, EPI_ISL_730026, EPI_ISL_730035, EPI_ISL_730048			
see above	Nigeria Centre for Disease Control (NCDC)	African Centre of Excellence for Genomics of Infectious Diseases (ACEGID), Redeemer's University, Ede, Osun State, Nigeria	Ajogbasile F.V.; Folarin O.A.; Happi C.T.; Ihekweazu C.; Kayode A.; Oguzie J.; Olawoye I.; Olumade T.; Oluniyi P.E.; Oluniyi P.E. et al; Uwanibe J.
EPI_ISL_2240750, EPI_ISL_2240755, EPI_ISL_2240758, EPI_ISL_2240762, EPI_ISL_2285317	Nigeria Centre for Disease Control (NCDC)	African Centre of Excellence for Genomics of Infectious Diseases (ACEGID), Redeemer's University	A.T.; Abechi; Ajogbasile; Akano; C.A.; C.T.; Eromon; F.V.; Folarin, O.; Happi; I.B.; J.N.; J.U.; K.O.; Kayode; Nosamiefan, I.; Oguzie; Olawoye; Olumade; Oluniyi; P.E.; P.S.; T.J.; Ugwu; Uwanibe
EPI_ISL_2335052, EPI_ISL_2384261, EPI_ISL_2565933, EPI_ISL_2565965	North Dakota Department of Health, Public Health Laboratory	North Dakota Department of Health, Public Health Laboratory	Lisa Wingerter
EPI_ISL_579096, EPI_ISL_579406	North Shore Hospital	Institute of Environmental Science and Research (ESR)	Anja Werno; Antje van der Linden; Arlo Upton; Chris Mansell; David Hammer; Dragana Drinkovic; Erasmus Smit; Gary McAuliffe; Hana Sofia Andersson; Hermes Perez; James Ussher; Jill Sherwood; Jing Wang; Joep de Ligt; Josh Freeman; Julia Howard; Juliet Elvy; Lauren Jelly; Mary DeAlmeida; Matt Blakiston; Matt Storey; Matthew Rogers; Max Bloomfield; Michael Addidle; Michelle Balm; Muhammad Faisal; Nikki Freed; Olin Silander; Sally Roberts; Sarah Jefferies; Sharmini Muttaiyah; Susan Morpeth; Susan Taylor; Timothy Blackmore; Vani Sathyendran; Veronica Playle; Virginia Hope; Xiaoyun Ren
EPI_ISL_2156187	Northern Mindanao TB Regional Center	Philippine Genome Center	Alethea R. de Guzman; Anna Ong-Lim; Arianne A. Zamora; Asia Louisa U. Chong; Benedict A. Maralit; Candice Francheska B. Tambaoan; Carlo M. Lapid; Celia Carlos; Devon Ray Pacial; Edsel Maurice Salvaña; El King D. Morado; Eva Maria Cutiongco-de la Paz; Francis A. Tablizo; Irish Coleen A. Asin; Jaime C. Montoya; Jan Michael C. Yap; Jo-Hannah S. Llames; John Q. Wong; Joshua Gregor A. Dizon; Juan Antonio R. Magalang; Karol Sophia Agape R. Padilla; Kenneth M. Kim; Kris P. Punayan; Marc Edsel C. Ayes; Marc Jerrone R. Castro; Maria Rosario Singh-Vergeire and Cynthia P. Saloma; Maria Sofia L. Yangzon; Marissa Alejandria; Razel Nikka M. Hao; Rianna Patricia S. Cruz; Sheila Mae M. Araiza
EPI_ISL_2372961	Northwestern Memorial Hospital	Northwestern University - Ozer Lab	Chad J. Achenbach; Chao Qi; Egon A. Ozer; Judd F. Hultquist; Lacy M. Simons; Lawrence J. Jennings; Michael G. Ison; Ramon Lorenzo-Redondo; Taylor J. Dean
EPI_ISL_936958	Northwestern Memorial Hospital	Ozer Lab	Chad J. Achenbach; Egon A. Ozer; Judd F. Hultquist; Lacy M. Simons; Lawrence J. Jennings; Michael G. Ison; Ramon Lorenzo-Redondo
EPI_ISL_735447	Nucleic Acid Testing - Rwanda National Reference Laboratory	GIGA Medical Genomics	Bouchra Boujemla; Esperence Umumararungu; Jacob Souopgui; Keith Durkin; Léon Mutesa; Maria Artesi; Marie-Pierre Hayette; Patrick Tuyisenge; Robert Rutayisire; Sabin Nsanzimana; Swaibu Gatare; Sébastien Bontems; Vincent Bours; Yvan Butera
EPI_ISL_925892, EPI_ISL_960251, EPI_ISL_960300, EPI_ISL_960302, EPI_ISL_2362488, EPI_ISL_2362491, EPI_ISL_2362493, EPI_ISL_2362508, EPI_ISL_2362511, EPI_ISL_2362515, EPI_ISL_2362517, EPI_ISL_2362523, EPI_ISL_2362526, EPI_ISL_2365914, EPI_ISL_2521981, EPI_ISL_2521988			
see above	Nucleic Acid Testing, National Reference Laboratory	GIGA Medical Genomics	Bouchra Boujemla; Corinne Fasquelle; Esperence Umumararungu; Jacob Souopgui; Keith Durkin; Léon Mutesa; Maria Artesi; Marie-Pierre Hayette; Nathalie Renotte; Patrick Tuyisenge; Robert Rutayisire; Sabin Nsanzimana; Swaibu Gatare; Sébastien Bontems; Vincent Bours; Yvan Butera
EPI_ISL_2080768, EPI_ISL_2227694, EPI_ISL_2227695, EPI_ISL_2462074	OHSU Lab Services Molecular Microbiology Lab	Oregon SARS-CoV-2 Genome Sequencing Center	Alec J. Hirsch; Andrew C. Adey; Benjamin N. Bimber; Brendan L. O'Connell; Brian J. O'Roak; Cierra LaBlanc; Daniel N. Streblow; Donna Hansel; Guang Fan; Kayla Carter; Ruth V. Nichols; Sally Grindstaff; Sonia Acharya; William B. Messer; Xuan Qin
EPI_ISL_1272810, EPI_ISL_1272812	OK Public Health Laboratory, Oklahoma State DOH	Centers for Disease Control and Prevention Division of Viral Diseases, Pathogen Discovery	Anna Montmayeur; Anna Uehara; Ben L. Rambo-Martin; Clinton R. Paden; Dhwani Batra; Haibin Wang; Jasmine Padilla; Jing Zhang; Justin Lee; Katie Dillon; Krista Queen; Kristen Knipe; Kristine Lacek; Lori Rowe; Mark Burroughs; Matthew Schmerer; Mili Sheth; Peter W. Cook; Rachel Marine; Sam Shepard; Sarah Nobles; Shoshona Le; Suxiang Tong; Yan Li; Ying Tao
EPI_ISL_2295285, EPI_ISL_2311769	OK Public Health Laboratory, Oklahoma State DOH	Genomics and Discovery, Respiratory Viruses Branch, Division of Viral Diseases, Centers for Disease Control and Prevention	Adam Retchless; Anna Kelleher; Anna Uehara; Brian Lynch; Clinton R. Paden; Dhwani Batra; Haibin Wang; Han Jia Justin Ng; Jasmine Padilla; Jing Zhang; Justin Lee; Krista Queen; Mark Burroughs; Mili Sheth; Morgan Davis; Peter Cook; Rachel Marine; Sarah Nobles; Suxiang Tong; Tara Coalter; Yan Li; Ying Tao
EPI_ISL_1196869	OK Public Health Laboratory, Oklahoma State DOH	Pathogen Discovery, Respiratory Viruses Branch, Division of Viral Diseases, Centers for Disease Control and Prevention	Anna Kelleher; Anna Uehara; Brian Lynch; Clinton R. Paden; Haibin Wang; Han Jia Justin Ng; Jing Zhang; Krista Queen; Peter Cook; Suxiang Tong; Yan Li; Ying Tao
EPI_ISL_1678169	OR State PHL-Virology/Immunology Section	Centers for Disease Control and Prevention Division of Viral Diseases, Pathogen Discovery	Alison Laufer Halpin; Ben L. Rambo-Martin; Clinton R. Paden; Dakota Howard; Darlene Wagner; Dave Wentworth; Dhwani Batra; Jasmine Padilla; Justin Lee; Katie Dillon; Krista Queen; Kristen Knipe; Kristine Lacek; Mark Burroughs; Matthew Schmerer; Mili Sheth; Peter Cook; Sam Shepard; Sarah Nobles; Shoshona Le; Suxiang Tong; Vivien Dugan; Yvette Unoarumhi
EPI_ISL_468702	Ochsner Health	Bioinfoexperts, LLC	Amy Feehan; David J. Nolan; David Moraga Amador; Julia-Garcia-Diaz; Luke Caruso; Lydia Von Borstel; Rebecca Rose; Sissy Cross; Susanna L. Lamers; Tong Yang; Wayra Navia; Xiao Hui Zhou
EPI_ISL_882796, EPI_ISL_1138851	Office of Diseases Prevention and Control Region 4 Saraburi	COVID-19 Network Investigations (CONI) Alliance	Amornmas Kongklieng; Anek Mungaomklang; Angkana Huang; Anthony R. Jones; Arporn Wangwiwatsin; Bhakbhoom Panthan; Chonticha Klungtong; Duangkamon Loesbanluechai; Ekawat Pasomsub; Elizabeth Batty; Insee Sensorn; Janjira Thaipadungpanit; Jutikul Kaewmalakul; Kamolthip Atsawawaranunt; Khajohn Joonlasak; Khajohn Joonsalak; Kingkan Rakmanee; Krittikorn Kumpornsin; Namfon Kotanan; Nathamon Runnachot; Pakjira Rimdusit; Payon Pengyo; Praima Moonmuang; Prayuth Kaewmalang; Pukkaporn Parnwijitkul; Sataporn Hatsadichart; Sirinapa Singthong; Siriporn Lakesukthom; Siriwan Yaemnimnual; Stefan Fernandez; Suttiruk Changchawai; Thanat Chookajorn; Theerarat Kochakarn; Treewat Watthanachockchai; Vichan Pawun; Wasun Chantratita; Wonvimol Lemprasert; Wudtichai Manasatienkij
EPI_ISL_2752169	Ohio Department of Health Laboratory	Ohio Department of Health Laboratory	Allison Black; Brent Lee; Caitlin McDonnell; Eric Brandt; Erica Leasure; Glen McGillivary; Heather Blankenship; Holmes; Jade Mowery; Jennifer; Kelsey Florek; Keoni Omura; Kirtana Ramadugu; Quanta Brown; Stephanie Mccracken; Tyler Payne; and Tammy Bannerman
EPI_ISL_492014	Oman-NIC	Department of Microbiology and Immunology-SQUH	Abdulla Balkhair; Ahlam Al-Amri; Aisha Al-Amri; Aisha Al-Busaidi; Amina Al Jardani; Fahad Zadjali; Fatma BaAlawi; Hamida AL Barwani; Hanan Al-kindi; Intisar Al-Shukri; Khulood Al-Mammary; Mohammed Al-Tobi; Samiha Al Kharusi; Samira Al-Maruqi; Zeyana AL-Dahmani
EPI_ISL_1795052	Oman-NIC	Oman-National Influenza Center-Department of Microbiology and Immunology-SQUH	Abdulla Balkhair; Ahlam Al-Amri; Aisha Al-Amri; Aisha Al-Busaidi; Amina Al Jardani; Fahad Zadjali; Fatma BaAlawi; Hamida AL Barwani; Hanan Al-kindi; Intisar Al-Shukri; Khulood Al-Mammary; Mohammed Al-Tobi; Samiha Al Kharusi; Samira Al-Maruqi; Zeyana AL-Dahmani
EPI_ISL_491144, EPI_ISL_1517192, EPI_ISL_1532283, EPI_ISL_1532301, EPI_ISL_2603841, EPI_ISL_2603855	Oman-National Influenza Center	Biotechnology & OMICs Laboratory	Abdul Latif Khan; Adil Al-Wahaibi; Adil Khan; Ahlam Al-Amri; Ahmed Al Harrasi; Ahmed Al-Harrasi; Ahmed Al-Rawahi; Ahmed N Al-Rawahi; Aisha Al-Amri; Aisha Al-Busaidi; Amal Al-Maani; Amina Al-Jardani; Amina Al-Jardani.; Bilal Hussain; Fahad Al-Balushi; Hanan Al-Kindi; Intisar Al-Shukri; Laila Al-Balushi; Sajjad Asaf; Samiha Al-Kharusi; Samira Al-Mahruqi; Samiya Al-Zadjali; Saqib Bilal; Seif Al-Abri
EPI_ISL_2166354	Omega Diagnostics at Mounes	Omega Diagnostics at Mounes	Ashleigh McGrail; Cynthia Corley; Latira HAynes-Jacob; Vivek Khare MD
EPI_ISL_1848351, EPI_ISL_1897475, EPI_ISL_2139553, EPI_ISL_2473673, EPI_ISL_2557940	Oregon State Public Health Laboratory	Oregon State Public Health Laboratory	Eugene Yeboah; John Fontana and Shane Sevey; Laura Tsaknaridis; Rafia Razzaque; Vanda Makris
EPI_ISL_1253654	Oregon State University TRACE Project	OSU Center for Genome Research and Biocomputing	Oregon State University TRACE Project
EPI_ISL_1730635, EPI_ISL_1730640, EPI_ISL_1979258, EPI_ISL_1979296, EPI_ISL_1979362, EPI_ISL_1979377, EPI_ISL_1979393, EPI_ISL_2624685, EPI_ISL_2624902, EPI_ISL_2707793, EPI_ISL_2707795, EPI_ISL_2707913, EPI_ISL_2707947, EPI_ISL_2708019, EPI_ISL_2749956, EPI_ISL_2749962, EPI_ISL_2750058, EPI_ISL_2750131, EPI_ISL_2750271, EPI_ISL_2750323, EPI_ISL_2750439, EPI_ISL_2750473, EPI_ISL_2750518, EPI_ISL_2750689			
see above	Originating lab: Wales Specialist Virology Centre Sequencing lab: Pathogen Genomics Unit	Public Health Wales Microbiology Cardiff Wales Specialist Virology Centre	Alec Birchley; Alexander Adams; Amy Gaskin; Angela Marchbank; Bree Gatica-Wilcox; Catherine Moore; Jason Coombes; Joanne Watkins; Joel Southgate; Johnathan Evans; Laura Gifford; Lauren Gilbert; Lee Graham; Malorie Perry; Matthew Bull; Nicole Pacchiarini; Sally Corden; Sara Kumziene-Summerhayes; Sara Rey; Sarah Taylor; Simon Cottrell; Sophie Jones; Tom Connor
EPI_ISL_1578944, EPI_ISL_1599391, EPI_ISL_1819813, EPI_ISL_2323330	Ostfold Hospital Trust - Kalnes, Centre for Laboratory Medicine, Section for gene technology and infection serology	Norwegian Institute of Public Health, Department of Virology	Atiya R Ali; Debech Nadia; Engebretsen Serina Beate; Garcia Llorente Ignacio; Hilde Elshaug; Hilde Vollan; Jon Bråte; Kamilla Heddeland Instefjord; Karoline Bragstad; Kathrine Stene-Johansen; Line Victoria Moen; Marie Paulsen Madsen; Olav Hungnes; Pedersen Benedikte Nevjen; Rasmus Riis Kopperud
EPI_ISL_1273214, EPI_ISL_1293350	Oxford University Clinical Research Unit (OUCRU)	Oxford University Clinical Research Unit (OUCRU)	Guy Thwaites; Huynh Trung Trieu; Lam Minh Yen; Le Manh Hung; Le Nguyen Truc Nhu; Le Thi Thu Huong; Le Van Tan; Nghiem My Ngoc; Ngo Ngoc Quang Minh; Nguyen Thanh Dung; Nguyen Thanh Phong; Nguyen Thanh Truong; Nguyen Thi Thu Hong; Nguyen To Anh; Nguyen Tri Dung; Nguyen Van Vinh Chau; Tran Nguyen Hoang Tu; Tran Tan Thanh; le Thi Thu Huong; le Van Tan
EPI_ISL_2283729, EPI_ISL_2283735, EPI_ISL_2283747, EPI_ISL_2283748, EPI_ISL_2283750, EPI_ISL_2283751, EPI_ISL_2283752, EPI_ISL_2283754, EPI_ISL_2283755, EPI_ISL_2283756, EPI_ISL_2283757, EPI_ISL_2283759, EPI_ISL_2283761, EPI_ISL_2283762, EPI_ISL_2283763			
see above	PHV-FSS	PHV-FSS	Son Nguyen
EPI_ISL_596516, EPI_ISL_596519, EPI_ISL_596553, EPI_ISL_596565	Palestinian Ministry of Health	Molecular Genetics Lab	Damien Richard; Dana Najjar; Francois Balloux; Hisham Darwish; Husam Sallam; Issa Shtayeh; Lucy van Dorp; Mahmoud Ruzayqat; Nouar Qutob; Osama Najjar; Zaidoun Salah
EPI_ISL_707937	Pamukkale University Hospital	Pamukkale University Department of Medical Genetics	Onur TOKGUN et al.
EPI_ISL_1470937, EPI_ISL_1470959, EPI_ISL_1635519, EPI_ISL_1828111, EPI_ISL_1828260, EPI_ISL_1828349, EPI_ISL_1828425, EPI_ISL_1828512, EPI_ISL_1828556, EPI_ISL_1828565, EPI_ISL_1828653, EPI_ISL_1828694, EPI_ISL_2037012, EPI_ISL_2037056, EPI_ISL_2037333, EPI_ISL_2631390			
see above	Pandemic Response Lab - NYC	Pandemic Response Lab, R&D	Cybill del Castillo; Dylan Law; Haiping Hao; Henry Lee; Jon Laurent; Katharine Nelson; Melissa Hopkins; Michael Hammerling; Pradeep Bugga; Shinyoung Clair Kang; Sol Rey; William Ward
EPI_ISL_2189050	Pasig City Children's Hospital - Child's Hope	Philippine Genome Center	Alethea R. de Guzman; Anna Ong-Lim; Arianne A. Zamora; Asia Louisa U. Chong; Benedict A. Maralit; Candice Francheska B. Tambaoan; Carlo M. Lapid; Celia Carlos; Devon Ray Pacial; Edsel Maurice Salvaña; El King D. Morado; Elcid Aaron R. Pangilinan; Eva Maria Cutiongco-de la Paz; Francis A. Tablizo; Irish Coleen A. Asin; Jaime C. Montoya; Jan Michael C. Yap; Jo-Hannah S. Llames; John Q. Wong; Joshua Gregor A. Dizon; Juan Antonio R. Magalang; Karol Sophia Agape R. Padilla; Kenneth M. Kim; Kris P. Punayan; Marc Edsel C. Ayes; Maria Rosario Singh-Vergeire and Cynthia P. Saloma; Maria Sofia L. Yangzon; Marissa Alejandria; Razel Nikka M. Hao; Renato Jacinto Q. Mantaring; Rianna Patricia S. Cruz; Sheila Mae M. Araiza

EPI_ISL_2035941, EPI_ISL_2035942, EPI_ISL_2035945, EPI_ISL_2035946, EPI_ISL_2035949, EPI_ISL_2035988	Pasteur Institute - Laboratory of Clinical Virology	Pasteur Institute - Laboratory of Clinical Virology	Anissa Chouikha; Henda Triki; Kais Ghedira; Mariem Gdoura; Sondos Haddad; Wasfi Fares
EPI_ISL_2695822	PathCare, Cape Town	Division of Medical Virology, National Health Laboratory Service (NHLS), Tygerberg Hospital / Stellenbosch University	Bronwyn Kleinhans; Gert van Zyl; Jean Maritz; Petra Raimond; San Emmanual James; Susan Engelbrecht; Tania Stander; Tongai Maponga; Tulio de Oliveira; Wolfgang Preiser
EPI_ISL_456163, EPI_ISL_456222, EPI_ISL_456301, EPI_ISL_582018, EPI_ISL_582021, EPI_ISL_622805, EPI_ISL_755626			
see above	PathLab Bay of Plenty	Institute of Environmental Science and Research (ESR)	Anja Werno; Antje van der Linden; Arlo Upton; Chris Mansell; David Hammer; Dragana Drinkovic; Erasmus Smit; Gary McAuliffe; Hana Sofia Andersson; Hermes Perez; James Ussher; Jill Sherwood; Jing Wang; Joep de Ligt; Josh Freeman; Julia Howard; Juliet Elvy; Lauren Jelly; Mary DeAlmeida; Matt Blakiston; Matt Storey; Matthew Rogers; Max Bloomfield; Michael Addidle; Michelle Balm; Muhammad Faisal; Nikki Freed; Olin Silander; Sally Roberts; Sarah Jefferies; Sharmini Muttaiyah; Susan Morpeth; Susan Taylor; Timothy Blackmore; Vani Sathyendran; Veronica Playle; Virginia Hope; Xiaoyun Ren
EPI_ISL_2013036	Pathcare-Vermaak Centurion	National Institute for Communicable Diseases of the National Health Laboratory Service	Amoako DG; Bhiman JN; Glass A; Gottberg A; Mahlangu B; Mohale T; Ntuli N; Oliveira TD; Scheepers C; Tegally H; Viana R
EPI_ISL_437483, EPI_ISL_437695, EPI_ISL_437743, EPI_ISL_512923, EPI_ISL_513113, EPI_ISL_513178, EPI_ISL_513211, EPI_ISL_678159			
see above	Pathogen Genomics Lab King Abdullah University of Science and Technology(KAUST)	Pathogen Genomics Lab King Abdullah University of Science and Technology(KAUST)	Abbas Al Mutair; Abdulaziz Alahmadi; Afrah Alsomali; Ahmad Bakur Mahmoud; Amanda; Amanda Ooi; Amit Kumar Subudhi; Amit Subudhi; Anwar Hashem; Arnab Pain; Asim Khogeer; Awad Al-Omari; Fadwa Alofi; Fathia Ben Rached; Fathia Ben-Rached; Fathia Rached; Jumana Taha; Kahled Algithami; Khaled Algithami; Luke; Luke Esau; Naif Almontashiri; Nashwa Al-khotani; Raeece Naeem; Rahul P Salunke; Rahul Salunke; Samer Salih; Sara Mfarrej; Sharif Hala
EPI_ISL_2153940	Philippine Genome Center - Biobank (NCR)	Philippine Genome Center	Alethea R. de Guzman; Anna Ong-Lim; Arianne A. Zamora; Asia Louisa U. Chong; Benedict A. Maralit; Candice Francheska B. Tambaoan; Carlo M. Lapid; Celia Carlos; Devon Ray Pacial; Edsel Maurice Salvaña; El King D. Morado; Eva Maria Cutiongco-de la Paz; Francis A. Tablizo; Irish Coleen A. Asin; Jaime C. Montoya; Jan Michael C. Yap; Jo-Hannah S. Llames; John Q. Wong; Joshua Gregor A. Dizon; Juan Antonio R. Magalang; Karol Sophia Agape R. Padilla; Kenneth M. Kim; Kris P. Punayan; Marc Edsel C. Ayes; Marc Jerrone R. Castro; Maria Rosario Singh-Vergeire and Cynthia P. Saloma; Maria Sofia L. Yangzon; Marissa Alejandria; Razel Nikka M. Hao; Rianna Patricia S. Cruz; Sheila Mae M. Araiza
EPI_ISL_2153914	Philippine Genome Center - Biobank (Region IV-A)	Philippine Genome Center	Alethea R. de Guzman; Anna Ong-Lim; Arianne A. Zamora; Asia Louisa U. Chong; Benedict A. Maralit; Candice Francheska B. Tambaoan; Carlo M. Lapid; Celia Carlos; Devon Ray Pacial; Edsel Maurice Salvaña; El King D. Morado; Eva Maria Cutiongco-de la Paz; Francis A. Tablizo; Irish Coleen A. Asin; Jaime C. Montoya; Jan Michael C. Yap; Jo-Hannah S. Llames; John Q. Wong; Joshua Gregor A. Dizon; Juan Antonio R. Magalang; Karol Sophia Agape R. Padilla; Kenneth M. Kim; Kris P. Punayan; Marc Edsel C. Ayes; Marc Jerrone R. Castro; Maria Rosario Singh-Vergeire and Cynthia P. Saloma; Maria Sofia L. Yangzon; Marissa Alejandria; Razel Nikka M. Hao; Rianna Patricia S. Cruz; Sheila Mae M. Araiza
EPI_ISL_2558052	Philippine Red Cross - Clark Molecular Laboratory	Philippine Genome Center	Alethea R. de Guzman; Anna Ong-Lim; Arianne A. Zamora; Asia Louisa U. Chong; Benedict A. Maralit; Candice Francheska B. Tambaoan; Carlo M. Lapid; Celia Carlos; Devon Ray Pacial; Edsel Maurice Salvaña; El King D. Morado; Elcid Aaron R. Pangilinan; Eva Maria Cutiongco-de la Paz; Francis A. Tablizo; Irish Coleen A. Asin; Jaime C. Montoya; Jan Michael C. Yap; Jo-Hannah S. Llames; John Q. Wong; Joshua Gregor A. Dizon; Juan Antonio R. Magalang; Karol Sophia Agape R. Padilla; Kenneth M. Kim; Kris P. Punayan; Marc Edsel C. Ayes; Maria Rosario Singh-Vergeire and Cynthia P. Saloma; Maria Sofia L. Yangzon; Marissa Alejandria; Razel Nikka M. Hao; Renato Jacinto Q. Mantaring; Rianna Patricia S. Cruz; Sheila Mae M. Araiza
EPI_ISL_2105487	Philippine Red Cross Logistics and Multipurpose Center	Philippine Genome Center	Alethea R. de Guzman; Anna Ong-Lim; Arianne A. Zamora; Asia Louisa U. Chong; Benedict A. Maralit; Candice Francheska B. Tambaoan; Carlo M. Lapid; Celia Carlos; Devon Ray Pacial; Edsel Maurice Salvaña; El King D. Morado; Eva Maria Cutiongco-de la Paz; Francis A. Tablizo; Irish Coleen A. Asin; Jaime C. Montoya; Jan Michael C. Yap; Jo-Hannah S. Llames; John Q. Wong; Joshua Gregor A. Dizon; Juan Antonio R. Magalang; Karol Sophia Agape R. Padilla; Kenneth M. Kim; Kris P. Punayan; Marc Edsel C. Ayes; Marc Jerrone R. Castro; Maria Rosario Singh-Vergeire and Cynthia P. Saloma; Maria Sofia L. Yangzon; Marissa Alejandria; Razel Nikka M. Hao; Rianna Patricia S. Cruz; Sheila Mae M. Araiza
EPI_ISL_2759188	Population Medicine and Diagnostic Sciences, Cornell University	Population Medicine and Diagnostic Sciences, Cornell University	Anderson; B.D.; Caserta; Cronk; D.G.; Diel; L.C.; Laverack, M.; Mitchell; P.K.; Plocharczyk, E.; R.R.; Venugopalan, R.
EPI_ISL_2550758, EPI_ISL_2550759	Port Dickson Hospital	Institute for Medical Research, Infectious Disease Research Centre, National Institutes of Health, Ministry of Health Malaysia	Azizan MA; Kamel K; Mohd Zawawi Z; Ramly N; Robert F; Suppiah J; Thayan R
EPI_ISL_712071	Port Elizabeth Provincial Hospital, National Health Laboratory Services, Eastern Cape, South Africa	National Institute for Communicable Diseases of the National Health Laboratory Service	Allam M; Bhiman JN; Ismail A; Mahlangu B; Mohale T; Ntuli N
EPI_ISL_2188209	Prime Care Alpha Covid-19 Testing Laboratory	Philippine Genome Center	Alethea R. de Guzman; Anna Ong-Lim; Arianne A. Zamora; Asia Louisa U. Chong; Benedict A. Maralit; Candice Francheska B. Tambaoan; Carlo M. Lapid; Celia Carlos; Devon Ray Pacial; Edsel Maurice Salvaña; El King D. Morado; Elcid Aaron R. Pangilinan; Eva Maria Cutiongco-de la Paz; Francis A. Tablizo; Irish Coleen A. Asin; Jaime C. Montoya; Jan Michael C. Yap; Jo-Hannah S. Llames; John Q. Wong; Joshua Gregor A. Dizon; Juan Antonio R. Magalang; Karol Sophia Agape R. Padilla; Kenneth M. Kim; Kris P. Punayan; Marc Edsel C. Ayes; Marc Jerrone R. Castro; Maria Rosario Singh-Vergeire and Cynthia P. Saloma; Maria Sofia L. Yangzon; Marissa Alejandria; Razel Nikka M. Hao; Rianna Patricia S. Cruz; Sheila Mae M. Araiza
EPI_ISL_1477046	Private clinic of Biogen Med, Tashkent, Uzbekistan	Center of Genomics and bioinformatics, Bioinformatics laboratory	Abdurakhmon N Yusupov; Ibrokhim Y Abdurakhmonov.; Mirzakamol S Ayubov; Mukhammadjon H Mirzakhmedov; Shukhrat E Shermatov; Zabardast T Buriev
EPI_ISL_1789673, EPI_ISL_1840246	Pro-Vitam Diagnostics and Research Laboratory	Pro-Vitam Diagnostics and Research Laboratory	Istvan Horvath; Kinga Rakosi; Monika Korodi; Szilard N. Fejer; Zsuzsanna Jenei
EPI_ISL_2259439	Providence Little Company of Mary Medical Center	Los Angeles County PHL	P. Hemarajata et al.
EPI_ISL_2551452	Providence Regional Medical Center Everett	Providence St. Joseph Health Molecular Genomics Laboratory	Alexa K Dowdell; Brian D Piening; Carlo B Bifulco; Fred L Robinson; Mary Campbell; Rogan Rattray
EPI_ISL_1908865, EPI_ISL_1909256, EPI_ISL_1909924	Provincial Public Health Reference Laboratory	Provincial Public Health Reference Laboratory	ANDLEEB HANIF; Andleeb Hanif
EPI_ISL_2543485	Provincial Public Health Reference Laboratory (PPHRL)	Provincial Public Health Reference Laboratory	ANDLEEB HANIF; MARIAM JAVED & HASSAN NAWAZ
EPI_ISL_2544198, EPI_ISL_2544508	Provincial Public Health Reference Laboratory (PPHRL) P&SHD	Provincial Public Health Reference Laboratory (PPHRL) P&SHD	ANDLEEB HANIF
EPI_ISL_1520298, EPI_ISL_1520315, EPI_ISL_1524143, EPI_ISL_1526698, EPI_ISL_1647753, EPI_ISL_2448726, EPI_ISL_2448846, EPI_ISL_2598497			
see above	Public Health Authority of the Slovak Republic	Laboratory of Genomics and Bioinformatics, Comenius University Science Park	Anna Gičová; Diana Rusňáková; Jakub Styk; Jaroslav Budiš; Miroslav Böhmer; Tatiana Sedláčková; Tomáš Szemes
EPI_ISL_2380140	Public Health Authority of the Slovak Republic	Public Health Authority of the Slovak Republic	Anna Gičová; Barbora Kotvasová; Elena Tichá; Lucia Ševčíková; Miroslav Böhmer; Pavol Mišenko; Terézia Vrabľová; Tomáš Szemes
EPI_ISL_2351056	Public Health Center 23 Siphraya	Division of Genomic Medicine and Innovation support,Department of Medical Sciences, Ministry of Public Health, Thailand	Archawin Rojanawiwat; Jirapha Pakdee; Natthakul Bunneang; Nuanjun Wichukchinda; Penpitcha Thawong; Pilailuk Akkapaiboon Okada; Pundharika Piboonsiri; Surakameth Mahasirimongkol; Waritta Sawaengdee
EPI_ISL_2351115, EPI_ISL_2351142	Public Health Center 52 Samsennok	Division of Genomic Medicine and Innovation support,Department of Medical Sciences, Ministry of Public Health, Thailand	Archawin Rojanawiwat; Jirapha Pakdee; Natthakul Bunneang; Nuanjun Wichukchinda; Penpitcha Thawong; Pilailuk Akkapaiboon Okada; Pundharika Piboonsiri; Surakameth Mahasirimongkol; Waritta Sawaengdee
EPI_ISL_1112297, EPI_ISL_1112753	Public Health Center of Ukraine	Charité Universitätsmedizin Berlin, Institute of Virology	Barbara Mühlemann; Christian Drosten; Ihor Kuzin; Iryna Demchyshyna; Julia Schneider; Jörn Beheim-Schwarzbach; Liudmyla Chernenko; Roman Rodyna; Talitha Veith; Terry Jones; Victor M Corman
EPI_ISL_2035726	Public Institution of Health Care "Diagnostic Center (Laboratory Testing Center) of Moscow Health Department "	Group of Genomics and Postgenomic Technologies of Central Research Institute of Epidemiology	Akimkin VG; Kaptelova VV; Kondrasheva LY; Korneenko EV; Nadtoka MI; Saenko SS; Samoilov AE; Smirnova YS; Spakova OG; Speranskaya AS; Tivanova EV
EPI_ISL_434542	Puerto Rico Department of Health	Centers for Disease Control and Prevention, Dengue Branch	Betzabel Flores; Chaney Kalinich; Fabiola Cruz; Gilberto A. Santiago; Glenda Gonzalez; Jessica I. Falcon; Jorge L. Munoz-Jordan; Joseph Fauver; Keyla Charriez; Nathan Grubaugh
EPI_ISL_1424758, EPI_ISL_1424786, EPI_ISL_1424816, EPI_ISL_1424838, EPI_ISL_1424839	Queensland Medical Laboratories	Melbourne Diagnostic Unit Public Health Laboratory (MDU-PHL)	N.L.; Palou, T.; Seemann, T.; Sherry; Vaccher, S.
EPI_ISL_1424523, EPI_ISL_1424528, EPI_ISL_1424534, EPI_ISL_1424563, EPI_ISL_1424565, EPI_ISL_1424568, EPI_ISL_1424571, EPI_ISL_1424588, EPI_ISL_1424609, EPI_ISL_1424619, EPI_ISL_1424625			
see above	Queensland Medical Laboratories	Victorian Infectious Diseases Reference Laboratory (VIDRL) and the Melbourne Diagnostic Unit Public Health Laboratory (MDU-PHL)	N.L.; Palou, T.; Seemann, T.; Sherry; Vaccher, S.
EPI_ISL_2259251	Quest Diagnostics	Los Angeles County PHL	P. Hemarajata et al.
EPI_ISL_498712, EPI_ISL_571572, EPI_ISL_937026	Quest Diagnostics	Quest Diagnostics	Anderson, B.; D.F.; Gerasimova, A.; Grover, D.; Hua, M.; K.E.; Kagan; Lacbawan, F.; Liu Y.; Livingston; Owen, R.; R.M.; R.M. and Owen, R.; Rosenthal; S.H.; Shalhout
EPI_ISL_1194304, EPI_ISL_1220518, EPI_ISL_1220603, EPI_ISL_1252990, EPI_ISL_1253022, EPI_ISL_1253029, EPI_ISL_1253394, EPI_ISL_1253515, EPI_ISL_1254018, EPI_ISL_1267467, EPI_ISL_1340714, EPI_ISL_1422462, EPI_ISL_1648208, EPI_ISL_1648249, EPI_ISL_1738201, EPI_ISL_1753041, EPI_ISL_1753105, EPI_ISL_1753145, EPI_ISL_1761938, EPI_ISL_1798071, EPI_ISL_1798096, EPI_ISL_1818187, EPI_ISL_1818383, EPI_ISL_1834352, EPI_ISL_1840480, EPI_ISL_1840498, EPI_ISL_1906045, EPI_ISL_1924396, EPI_ISL_1924670, EPI_ISL_1924762, EPI_ISL_1924796, EPI_ISL_1924930, EPI_ISL_1925063, EPI_ISL_1989753, EPI_ISL_1989865, EPI_ISL_1989866, EPI_ISL_1989891, EPI_ISL_1989941, EPI_ISL_1990160, EPI_ISL_2011131, EPI_ISL_2046851, EPI_ISL_2046943, EPI_ISL_2047029, EPI_ISL_2047254, EPI_ISL_2047258, EPI_ISL_2090484, EPI_ISL_2090489, EPI_ISL_2090542, EPI_ISL_2133258, EPI_ISL_2133308, EPI_ISL_2133309, EPI_ISL_2133312, EPI_ISL_2133434, EPI_ISL_2133436, EPI_ISL_2133456, EPI_ISL_2133475, EPI_ISL_2142939, EPI_ISL_2142947, EPI_ISL_2143008, EPI_ISL_2143023, EPI_ISL_2143064, EPI_ISL_2143096, EPI_ISL_2143229, EPI_ISL_2143359, EPI_ISL_2143363, EPI_ISL_2143449, EPI_ISL_2143679, EPI_ISL_2143750, EPI_ISL_2143921, EPI_ISL_2143986, EPI_ISL_2180479, EPI_ISL_2180501, EPI_ISL_2204104, EPI_ISL_2268965, EPI_ISL_2368037, EPI_ISL_2371936, EPI_ISL_2439373, EPI_ISL_2526761, EPI_ISL_2529125, EPI_ISL_2529161, EPI_ISL_2599725, EPI_ISL_2599745, EPI_ISL_2599854, EPI_ISL_2611845			
see above	Quest Diagnostics Incorporated	Centers for Disease Control and Prevention Division of Viral Diseases, Pathogen Discovery	A. Gerasimova; A. Perez; Adrian Paskey; B. Anderson; Ben L. Rambo-Martin; Benjamin Rambo-Martin; Christopher Gulvick; Clinton R. Paden; Dakota Howard; Darlene Wagner; Dhwani Batra; Duncan MacCannell; F. Lacbawan; I. A. Shlyakhter; Jason Caravas; K.E. Livingston; Kara Moser; L.E. Bernstein; M. Hua; Matthew Schmerer; P. Tanpaiboon; Peter W. Cook; R. M. Kagan; R. Owen; R. V. Rolando; S. H. Rosenthal; Scott Sammons; Shatavia Morrison; Suxiang Tong; Y. Liu; Yvette Unoarumhi
EPI_ISL_1087176, EPI_ISL_1139328	Quest Diagnostics Incorporated	Respiratory Viruses Branch, Division of Viral Diseases, Centers for Disease Control and Prevention	A. Gerasimova; A. Perez; B. Anderson; Ben L. Rambo-Martin; Clinton R. Paden; Dakota Howard; Dhwani Batra; Duncan MacCannell; F. Lacbawan; I. A. Shlyakhter; K.E. Livingston; L.E. Bernstein; M. Hua; P. Tanpaiboon; Peter W. Cook; R. M. Kagan; R. Owen; R. V. Rolando; S. H. Rosenthal; Suxiang Tong; Y. Liu
EPI_ISL_2284685	ROB FERREIRA LABORATORY	National Institute for Communicable Diseases of the National Health Laboratory Service	Amoako DG; Bhiman JN; Ismail A; Mahlangu B; Mohale T; Ntuli N; Scheepers C
EPI_ISL_2348642, EPI_ISL_2348646	Rakai Health Sciences Program	MRC/UVRI & LSHTM Uganda Research Unit	Charles Ssuuna; Dan Lule Bugembe; Matthew Cotten; My V.T. Phan; Pontiano Kaleebu; Ronald Moses Galiwango; Steven J Reynolds
EPI_ISL_447025,	Ramathibodi Hospital	COVID-19 Network Investigations (CONI) Alliance	Angkana Huang; Anthony R. Jones; Arporn Wangwiwatsin; Bhakbhoom Panthan; Chonticha Klungtong; Duangkamon Loesbanluechai; Ekawat Pasomsub; Elizabeth Batty; Insee Sensorn; Janjira Thaipadungpanit; Khajohn Joonlasak; Khajohn Joonsalak; Kingkan

EPI_ISL_1073958, EPI_ISL_1138844, EPI_ISL_1491555			Rakmanee; Krittikorn Kumpornsin; Namfon Kotanan; Stefan Fernandez; Thanat Chookajorn; Theerarat Kochakarn; Treewat Watthanachockchai; Wasun Chantratita; Wudtichai Manasatienkij
EPI_ISL_1806953, EPI_ISL_1912766, EPI_ISL_1912771, EPI_ISL_1912782, EPI_ISL_2022507, EPI_ISL_2022525, EPI_ISL_2022578			
see above	Randox Laboratories	Wellcome Sanger Institute for the COVID-19 Genomics UK (COG-UK) Consortium	Cordelia Langford; David K. Jackson; Dominic Kwiatkowski; Ewan Harrison; Ian Johnston; Jeffrey Barrett; John Sillitoe on behalf of the Wellcome Sanger Institute COVID-19 Surveillance Team; Randox Laboratories and Alex Alderton; Roberto Amato; Sonia Goncalves
EPI_ISL_2473913, EPI_ISL_2662287	Reditus Laboratories	Reditus Laboratories	Alexa Eichelberger; Cassy Philips; Joshua J. Geltz; M.S.; Ph.D.; Rex Dyer; Robert M. Sgambelluri
EPI_ISL_1942265	Regional Authority of Public Health Banská Bystrica Slovakia	SVFI, Veterinary institute in Zvolen, Slovakia	Dirbáková Z.; Mancoš M.; Maďarová L.; Mojžiš M.; Mokryšová S.; Strhársky J.; Sujová S.; Tinák M.
EPI_ISL_2433518	Regional Medical Sciences Center 1/1 Chiangrai	Division of Genomic Medicine and Innovation support,Department of Medical Sciences, Ministry of Public Health, Thailand	Archawin Rojanawiwat; Jirapha Pakdee; Natthakul Bunneang; Nuanjun Wichukchinda; Penpitcha Thawong; Pilailuk Akkapaiboon Okada; Pundharika Piboonsiri; Surakameth Mahasirimongkol; Waritta Sawaengdee
EPI_ISL_768537	Regional Medical Sciences Center 5 Samut Songkhram	National Institute of Health, Department of Medical Sciences, Ministry of Public Health, Thailand	; Natchaya Khiadsang; Pakorn Piromtong; Pilailuk Okada; Ratana Tacharoenmuang; Siripaporn Phuygun; Sittiporn Parnmen; Sunthareeya Waicharoen; Thanutsapa Thanadachakul; Warawan Wongboot; sirikanda wimol
EPI_ISL_708737	Regional medical sciences center 6 chonburi	National Institute of Health, Department of Medical Sciences, Ministry of Public Health, Thailand	Malinee Chittaganpitch; Pilailuk Okada; Siripaporn Phuygun; Sittiporn Parnmen; Sunthareeya Waicharoen; Thanutsapa Thanadachakul; Warawan Wongboot
EPI_ISL_1394032	Regionalne Centrum Krwiodawstwa i Krwiolecznictwa w Białymstoku Pracownia Diagnostyki Molekularnej wirusa SARS-CoV-2	1. National Institute of Public Health - National Institute of Hygiene; 2. Eurofins Genomics Europe Sequencing GmbH	ECDC COVID-19 WGS support team; Eurofins Genomics Europe Sequencing Team; Gierczyński Rafał; Sadkowska-Todys Małgorzata; Wołkowicz Tomasz; Zacharczuk Katarzyna
EPI_ISL_2155223	Research Institute for Tropical Medicine, Inc. (RITM)	Philippine Genome Center	Alethea R. de Guzman; Anna Ong-Lim; Arianne A. Zamora; Asia Louisa U. Chong; Benedict A. Maralit; Carlo Francheska B. Tambaoan; Carlo M. Lapid; Celia Carlos; Devon Ray Pacial; Edsel Maurice Salvaña; El King D. Morado; Eva Maria Cutiongco-de la Paz; Francis A. Tablizo; Irish Coleen A. Asin; Jaime C. Montoya; Jan Michael C. Yap; Jo-Hannah S. Llames; John Q. Wong; Joshua Gregor A. Dizon; Juan Antonio R. Magalang; Karol Sophia Agape R. Padilla; Kenneth M. Kim; Kris P. Punayan; Marc Edsel C. Ayes; Marc Jerrone R. Castro; Maria Rosario Singh-Vergeire and Cynthia P. Saloma; Maria Sofia L. Yangzon; Marissa Alejandria; Razel Nikka M. Hao; Rianna Patricia S. Cruz; Sheila Mae M. Araiza
EPI_ISL_2544702, EPI_ISL_2544703, EPI_ISL_2544707, EPI_ISL_2544708, EPI_ISL_2544712, EPI_ISL_2544713	Research Center for Emerging Viral Infections, Chang Gung University, Taiwan	Research Center for Emerging Viral Infections, Chang Gung University, Taiwan	Carol Wang; Chung-Guei Huang; Hsiao-Chen Tu; Hui-Ying Weng; Hung-Yu Shu; Jason Su; Jora Lin; Kuo-Ming Lee; Po-Wei Huang; Pocky Lai; Shih-Feng Tsai; Shin-Ru Shih; Shu-Li Yang; Tsu-Lan Wu; Yu-Nong Gong; Yung-Feng Lin
EPI_ISL_2748530	Respiratory Virus Unit, Microbiology Services Colindale, Public Health England	COVID-19 Genomics UK (COG-UK) Consortium	PHE Covid Sequencing Team
EPI_ISL_2106001, EPI_ISL_2106013, EPI_ISL_2106069, EPI_ISL_2254398	Respiratory Viruses Branch, Centers for Disease Control and Prevention	Respiratory Viruses Branch, Centers for Disease Control and Prevention	Baker; Bakhash; Batra, D.; Breit, N.; C.R.; Campbell, P.; Caravas, J.; Case, R.; Clark, C.; Cook; Ellis, S.; Ghorpade, V.; Greninger, A.; Gulvick, C.; Hardison, M.; Houdeshell, H.; Howard, D.; Huang; Jerome; K.R.; Kvalvaag, O.; Lin, M.; Livingston; M.-L.; M.S.; MacCannell, D.; Mathias, P.; Morrison, S.; Moser, K.; N.R.; Nall, D.; Nguyen; P.W.; Paden; Paskey, A.; Perez, R.; R.J.; Rambo-Martin, B.; Roychoudhury; S.M.; Sammons, S.; Sanders, E.; Schmerer, M.; Shrestha, L.; T.V.; Unoarumhi, Y.; Vest, A.; Wagner, D.; Westlund, S.; Xie, H.
EPI_ISL_2621615	Respiratory Viruses Branch, US Centers for Disease Control and Prevention	Respiratory Viruses Branch, US Centers for Disease Control and Prevention	Batra, D.; Burroughs, M.; C.R.; Coalter, T.; Cook, P.; Davis, M.; H.J.J.; Kelleher, A.; Lee, J.; Li, Y.; Lynch, B.; Marine, R.; Ng; Nobles, S.; Paden; Padilla, J.; Queen, K.; Retchless, A.; Sheth, M.; Tao, Y.; Tong, S.; Uehara, A.; Wang, H.; Zhang, J.
EPI_ISL_1253986, EPI_ISL_1407397, EPI_ISL_2016594, EPI_ISL_2133866	Rhode Island Department of Health	Infectious Disease Program, Broad Institute of Harvard and MIT	Adams, G.; Azevedo, K.; B.L.; B.W.; Bauer, M.; Birren; Carter, A.; Chaluvadi, S.; D.J.; DeRuff, K.; Gallagher, G.; Gladden-Young, A.; Huard, R.; J.E.; K.J.; King, E.; Lagerborg, K.; Lemieux; Loreth, C.; MacInnis; Miller, A.; Normandin, E.; P.C.; Park; Pearlman, L.; Reilly, S.; Rudy, M.; Sabeti; Siddle; Smole, S.; Tomkins-Tinch, C.; and MacInnis; and Sabeti
EPI_ISL_491298	Rural Health Unit - Calauan, Laguna	Research Institute for Tropical Medicine	Bautista; Brunker, K.; C.S.; C.T.; D.L.; Demetria; E.S.; F.G.M.; I.A.P.; M.A.A.; Manalo; Medado; Mercado; O.J.T.; Onza; Polotan; Tujan
EPI_ISL_1272759	SC Dept of Health and Env. Control-Bureau of Laboratories	Centers for Disease Control and Prevention Division of Viral Diseases, Pathogen Discovery	Anna Montmayeur; Anna Uehara; Ben L. Rambo-Martin; Clinton R. Paden; Dhwani Batra; Haibin Wang; Jasmine Padilla; Jing Zhang; Justin Lee; Katie Dillon; Krista Queen; Kristen Knipe; Kristine Lacek; Lori Rowe; Mark Burroughs; Matthew Schmerer; Mili Sheth; Peter W. Cook; Rachel Marine; Sam Shepard; Sarah Nobles; Shoshona Le; Suxiang Tong; Yan Li; Ying Tao
EPI_ISL_2695238	SESARAM	Instituto Nacional de Saude (INSA)	Borges et al
EPI_ISL_2105766, EPI_ISL_2231022, EPI_ISL_2444479, EPI_ISL_2603801, EPI_ISL_2603805, EPI_ISL_2603812, EPI_ISL_2616785, EPI_ISL_2616845, EPI_ISL_2616851, EPI_ISL_2616859, EPI_ISL_2616864, EPI_ISL_2616874, EPI_ISL_2616879, EPI_ISL_2616937, EPI_ISL_2616953, EPI_ISL_2616966, EPI_ISL_2616986, EPI_ISL_2692733			
see above	Salud Digna	Instituto Nacional de Medicina Genomica	Abraham Campos-Romero; Cedro-Tanda A; Escobar-Arrazola; Gonzalez-Barrera D; Herrera-Montalvo LA.; Hidalgo-Miranda A; Luna-Ruiz Marco; M; Mendoza-Vargas A; Moreno-Camacho José Luis; Munguia-Garza P; Ramirez-Vega O; Rangel-DeLeon D; Reyes-Grajeda JP; Rodriguez-Gallegos Jorge
EPI_ISL_750169, EPI_ISL_750171, EPI_ISL_750172, EPI_ISL_751011	Sanatorio Americano	Institut Pasteur de Montevideo	Ana Carolina Mendonça; Andrés Lizasoain; Camila Simoes; Cecilia Alonso; Cecilia Salazar; Daiana Mir; Fernando López-Tort; Fernando Motta; Gonzalo Bello; Ighor Arantes; Ignacio Ferrés; Jose Sotelo; Leticia Maya; Letícia Garay Martins; Luciana Appolinario; Lucía Spangenberg; Mailen Arleo; Mariana Brandes; Marilda Mendonça Siqueira; Marilda Tereza Mar da Rosa; María José Benitez-Galeano; Martín Graña; Matías Castells; Matías Victoria; Matías Salvo; Natalia Rego; Natalia Reyes; Pablo Smircich; Paola Cristina Resende; Rodney Colina; Tamara Fernandez-Calero; Tania Possi; Tatiana Schäffer Gregianini; Verónica Noya; Yasser Vega
EPI_ISL_738611, EPI_ISL_1529221, EPI_ISL_2341495, EPI_ISL_2341560, EPI_ISL_2341641, EPI_ISL_2500033, EPI_ISL_2500136			
see above	Santa Clara County Public Health Laboratory	Chan-Zuckerberg Biohub	CZB Cliahub Consortium
EPI_ISL_2658987, EPI_ISL_2658993, EPI_ISL_2658999, EPI_ISL_2659011, EPI_ISL_2659023, EPI_ISL_2659027, EPI_ISL_2659045, EPI_ISL_2659046, EPI_ISL_2659048, EPI_ISL_2659079, EPI_ISL_2659090, EPI_ISL_2659099, EPI_ISL_2659101, EPI_ISL_2691231, EPI_ISL_2691232, EPI_ISL_2691239, EPI_ISL_2691249, EPI_ISL_2691268, EPI_ISL_2691273, EPI_ISL_2691282, EPI_ISL_2774577			
see above	Santa Clara Valley Medical Center	Santa Clara County Public Health Laboratory	Santa Clara County Public Health Department
EPI_ISL_661189	Scientific Veterinary Institute Novi Sad	Veterinary Specialized Institute "Kraljevo", Serbia	Afonso, C.; Banovic Djeri, B.; Jankovic, M.; Jovanovic, T.; Knezevic, A.; Petrovic, T.; Sekler, M.; Tesovic, B.; Vidanovic, D.; Volkening, J.
EPI_ISL_1794936	Scripps Medical Laboratory	Andersen lab at Scripps Research	Ellen Stefanski; Ian Mchardy; SEARCH Alliance San Diego with Michael Quigley
EPI_ISL_2011443, EPI_ISL_2011445, EPI_ISL_2489992, EPI_ISL_2490014	Seattle Flu Study	Seattle Flu Study	Amanda Adler; Barry R. Lutz; Benjamin Pelle; Caitlin R. Wolf; Chris D. Frazar; Deborah A. Nickerson; Elisabeth Brandstetter; Erica Ryke; Helen Y. Chu; Janet A. Englund; Jay Shendure; Jeff Duchin; Jover Lee; Kairsten Fay; Karen Cowgill; Kirsten Lacombe; Lea M. Starita; Mark J. Rieder; Matthew Richardson; Matthew Thompson; Melissa Truong; Michael Boeckh; Michael Famulare; Misja Ilcisin; Peter D. Han; Stephanie Schrag; Thomas R. Sibley; Trevor Bedford
EPI_ISL_1393370, EPI_ISL_1745589, EPI_ISL_2133041, EPI_ISL_2516852, EPI_ISL_2516895, EPI_ISL_2516908	Servicio de Microbiología Clínica (Complejo Hospitalario de Navarra, Pamplona)	Centro de Secuenciación NASERTIC	Ana Miqueleiz; Ana Navascués; Carmen Ezpeleta Baquedano
EPI_ISL_1589626	Servicio de Microbiología, Hospital Miguel Servet, Zaragoza	SeqCOVID-SPAIN consortium/IBV(CSIC)	Alexander Tristancho Baró; Ana Milagro; Antonio Rezusta López; Nieves Martínez Cameo and SeqCOVID-SPAIN consortium; Yolanda Gracia Grataloup
EPI_ISL_1786039, EPI_ISL_1786057	Servicio de Microbiología, Hospital Universitario Central de Asturias	SeqCOVID-SPAIN consortium/IBV(CSIC)	Cristián Castelló Abietar; Jose A. Boga; Marta Elena Álvarez-Argüelles; Santiago Melón and SeqCOVID-SPAIN consortium; Susana Rojo-Alba
EPI_ISL_1014721	Servicio de Microbiología. Hospital Ramón y Cajal. (CIBERESP)	SeqCOVID-SPAIN consortium/IBV(CSIC)	Jose Mª González-Alba; Juan C Galán and SeqCOVID-SPAIN consortium; L. Olavarrieta; Val Fernández
EPI_ISL_2491679	Servizo de Microbioloxía. Complexo Hospitalario de Santiago de Compostela	Servizo de Microbioloxía. Complexo Hospitalario de Santiago de Compostela	Amparo Coira; Antonio Aguilera; Carlos García_Riestra; Daniel Navarro; Gema Barbeito; Iria Roca; José Llovo; Laura Millán; Manuela Hernández; María Luisa Pérez_del_Molino; Mercedes Treviño; Rocío trastoy; Teresa Lopez_Valiño; Xosé Costa
EPI_ISL_2231198	Sharp HealthCare Laboratory	Andersen lab at Scripps Research	Art Mendoza; Cathy Woerle; Jacquelyn Berumen; Liam McGinnis; Omid Bakhtar; SEARCH Alliance San Diego with Aaron Harding
EPI_ISL_582662	Sheikh Khalifa Medical City	Molecular/Surveillance lab Sheikh Khalifa Medical City	Amirtharaj Francis; Hala Imambaccus; Hiba Saud; Sahar Almarzooqi; Sajeed Abdul; Stefan Weber
EPI_ISL_583896, EPI_ISL_1969690, EPI_ISL_2566445	Singapore General Hospital	Department of Microbiology	Chayaporn Suphavilai; Chenhao Li; James Sim Heng Chiak; Karrie Ko; Kenneth Xin Long Chan; Kern Rei Chng; Kian Sing Chan; Kun Lee Lim; Lynette Oon; Niranjan Nagarajan; Nurdyana Abdul Rahman; Sui Sin Goh; Suphavilai Chayaporn
EPI_ISL_407987	Singapore General Hospital	Programme in Emerging Infectious Diseases, Duke-NUS Medical School	Danielle E Anderson; Gavin JD Smith; Jayanthi Jayakumar; Jenny GH Low; Kian Sing Chan; Linfa Wang; Lynette LE Oon; Martin Linster; Yan Zhuang; Yvonne CF Su
EPI_ISL_1761811	Sonora Quest Laboratories	TGen North	"Jolene Bowers; Ashlyn Pfeiffer; Chris French; Darrin Lemmer; Dave Engelthaler; Hayley Yaglom; Heather Centner; The Arizona COVID Genomics Union (ACGU)"
EPI_ISL_1253663	South Dakota Public Health Laboratory	South Dakota Public Health Laboratory	Jacob Garfin and Chris Carlson
EPI_ISL_1017040	South Dakota Public Health Laboratory	University of Minnesota Genomics Center	Benjamin Auch; Corbin Dirkx; Daryl M. Gohl; Jaquelyn Kuriger-Laber; John Garbe; and Chris Carlson
EPI_ISL_1307682	South Dakota Public Health Laboratory, South Dakota Department of Health	South Dakota Public Health Laboratory, South Dakota Department of Health	C. and Garfin, J.; Carlson
EPI_ISL_456238	Southern Community Labs Dunedin	Institute of Environmental Science and Research (ESR)	Anja Werno; Antje van der Linden; Arlo Upton; Chris Mansell; David Hammer; Dragana Drinkovic; Erasmus Smit; Gary McAuliffe; Hana Sofia Andersson; James Ussher; Jill Sherwood; Joep de Ligt; Josh Freeman; Julia Howard; Juliet Elvy; Lauren Jelly; Mary DeAlmeida; Matt Blakiston; Matt Storey; Matthew Rogers; Max Bloomfield; Michael Addidle; Michelle Balm; Sally Roberts; Sarah Jefferies; Sharmini Muttaiyah; Susan Morpeth; Susan Taylor; Timothy Blackmore; Vani Sathyendran; Veronica Playle; Virginia Hope; Xiaoyun Ren
EPI_ISL_2230722	Southern Nevada Public Health Laboratory	Southern Nevada Public Health Laboratory	Michael Picker
EPI_ISL_1495113, EPI_ISL_1495132	State Institution «Public Health Center of Ministry of Health of Ukraine»	Robert Koch Institute, ZBS1 Highly Pathogenic Viruses, Berlin, Germany	Andreas Nitsche; Annika Brinkmann; Iryna Demchyshyna; Janine Michel; Liudmyla Chernenko; Roman Rodyna; Steven Uddin

EPI_ISL_752910, EPI_ISL_1771435, EPI_ISL_2007889, EPI_ISL_2007931	State Laboratories Division, Hawaii State Department of Health	State Laboratories Division, Hawaii State Department of Health	Ayana Garnet; Drew Kuwazaki; Edward Desmond; Pamela O'Brien; Razvan Sultana; Sabrina Diemert
EPI_ISL_2377955, EPI_ISL_2377967, EPI_ISL_2378174, EPI_ISL_2378500, EPI_ISL_2473486	State Testing Facility	Altius Institute for Biomedical Research	Alex Isner; Alex Nguyen; Amanda Gale; Audra Johnson; Clem Green; Daniel Bates; Eric Thorland; Jacob Rodriguez; Jean Robinston; Jemma Nelson; Jessica Kunder; John Stamatoyannopoulos; Joshua Richards; Julia Wald; Kneshay Harper; Lauren Mitchell; Mark Frerker; Matt Hartman; Michael Buckley; Muhammad Halimun; Rebecca Bruders; Sadie Patraw; Sofia Olsson; Tobias Ragoczy
EPI_ISL_491038	Suceava County Emergency Hospital	"Stefan cel Mare" University Metagenomics Lab	Antoniadis Panagiotis et al.; Lobiuc Andrei
EPI_ISL_2091020	Sungai Buloh Hospital	Institute for Medical Research, Infectious Disease Research Centre, National Institutes of Health, Ministry of Health Malaysia	Azizan MA; Kamel K; Mohd Zawawi Z; Ramly N; Robert F; Suppiah J; Thayan R
EPI_ISL_1290037, EPI_ISL_1290502, EPI_ISL_1420162, EPI_ISL_1604490, EPI_ISL_1676013, EPI_ISL_1808683, EPI_ISL_1808686, EPI_ISL_1899297, EPI_ISL_2032727, EPI_ISL_2033405, EPI_ISL_2033751, EPI_ISL_2034582, EPI_ISL_2034589, EPI_ISL_2205752, EPI_ISL_2205865, EPI_ISL_2206097, EPI_ISL_2208933, EPI_ISL_2209223, EPI_ISL_2211302, EPI_ISL_2217152, EPI_ISL_2220962, EPI_ISL_2257706, EPI_ISL_2411664, EPI_ISL_2415484, EPI_ISL_2416022, EPI_ISL_2418605, EPI_ISL_2418674, EPI_ISL_2419245, EPI_ISL_2419511, EPI_ISL_2583831, EPI_ISL_2613551, EPI_ISL_2619254, EPI_ISL_2619266, EPI_ISL_2619267, EPI_ISL_2619585, EPI_ISL_2619703, EPI_ISL_2620486, EPI_ISL_2793590, EPI_ISL_2793828, EPI_ISL_2793845			
see above	Swedish national genomic surveillance program of SARS-CoV-2	The Public Health Agency of Sweden	Alma Brolund; Maria Lind Karlberg; Maximilian Riess; Swedish national genomic surveillance program of SARS-CoV-2
EPI_ISL_2695522	TAMBO MEMORIAL	National Institute for Communicable Diseases of the National Health Laboratory Service	Amoako DG; Bhiman JN; Everatt J; Ismail A; Mahlangu B; Mnguni A; Mohale T; Ntuli N; Scheepers C
EPI_ISL_1392534	TGen North	TGen North	"Jolene Bowers; Ashlyn Pfeiffer; Chris French; Darrin Lemmer; Dave Engelthaler; Hayley Yaglom; Heather Centner; The Arizona COVID Genomics Union (ACGU)"
EPI_ISL_2156302	THE LORD'S GRACE MEDICAL AND INDUSTRIAL CLINIC	Philippine Genome Center	Alethea R. de Guzman; Anna Ong-Lim; Arianne A. Zamora; Asia Louisa U. Chong; Benedict A. Maralit; Candice Francheska B. Tambaoan; Carlo M. Lapid; Celia Carlos; Devon Ray Pacial; Edsel Maurice Salvaña; El King D. Morado; Elcid Aaron R. Pangilinan; Eva Maria Cutiongco-de la Paz; Francis A. Tablizo; Irish Coleen A. Asin; Jaime C. Montoya; Jan Michael C. Yap; Jo-Hannah S. Llames; John Q. Wong; Joshua Gregor A. Dizon; Juan Antonio R. Magalang; Karol Sophia Agape R. Padilla; Kenneth M. Kim; Kris P. Punayan; Marc Edsel C. Ayes; Marc Jerrone R. Castro; Maria Rosario Singh-Vergeire and Cynthia P. Saloma; Maria Sofia L. Yangzon; Marissa Alejandria; Razel Nikka M. Hao; Rianna Patricia S. Cruz; Sheila Mae M. Araiza
EPI_ISL_427395	TSGH-CP molecular lab	TSGH-CP molecular lab	Cherng-Lih Perng; Chien-Wen Chen; Chih-Kai Chang; Feng-Yee Chang; Hsing-Yi Chung; Hung-Sheng Shang; Jung-Chung Lin; Kuo-Ming Yeh; Kuo-Sheng Hung; Ming-Jr Jian; Sheng-Kang Chiu; Shih-Hung Tsai; Tien-Yao Chang
EPI_ISL_2141442, EPI_ISL_2177412, EPI_ISL_2443304, EPI_ISL_2443319	TXDSHS	TXDSHS	Anita Pokharel; Bonnie Oh; Chun Wang; Grace Kubin; Jenny Zhang; Karen Bobier; Lorraine Rodriguez; Maliha Rahman; Mayela Pedrueza; Myong Koag; Rachel Lee; Rashmi Tuladhar
EPI_ISL_411926	Taiwan Centers for Disease Control	Taiwan Centers for Disease Control	Ji-Rong Yang; Jung-Jung Mu; Ming-Tsan-Liu; Yu-Chi-Lin
EPI_ISL_2450575	Tambov Hospital	WHO National Influenza Centre Russian Federation	Andrey Komissarov; Artem Fadeev; Daria Danilenko; Dmitry Lioznov; Elena Nabieva; Georgii Bazykin; Kirill Varchenko; Ksenia Safina; Kseniya Komissarova; Maria Pisareva; Maria Timofeeva; Mikhail Bakaev; Natalia Polovitsa; Nikita Yolshin; Oula Mansour; Tamila Musaeva; Veronika Eder
EPI_ISL_2081386	Tampa General Hospital Esoteric Lab	Tampa General Hospital Esoteric Research & Development Lab	Amorce Lima; Deanna Becker; Dominic Uy; Grant Vestal; Suzane Silbert; Vicki Healer
EPI_ISL_1919863	Tawau Hospital	Institute for Medical Research, Infectious Disease Research Centre, National Institutes of Health, Ministry of Health Malaysia	Kamel K; Mohd Zawawi Z; Suppiah J; Thayan R
EPI_ISL_1293118	Tempus	Grubaugh Lab - Yale School of Public Health	Anderson Brito; Annie Watkins; Chaney Kalinich; Chantal Vogels; Christopher E. Mason; Gaurav Khullar; Isabell Ott; Jessica Metti; Jessica Rothman; Joel T. Dudley; Joseph Fauver; Mallery Breban; Mary Petrone; Matthew J. MacKay; Megan Nash; Nathan Grubaugh; Nike Beaubier; Tara Alpert
EPI_ISL_976909	Thai Red Cross Emerging Infectious Diseases Health Science Centre, Chulalongkorn Hospital, Faculty of Medicine, Chulalongkorn University	Thai Red Cross Emerging Infectious Diseases Center and Faculty of Medicine, Chulalongkorn University	Apaporn Rodpan; Gompol Suwanpimolkul; Leilani Paitoonpong; Opass Putcharoen; Pattama Torvorapanit; Sininat Petcharat; Sopon Iamsirithaworn; Supaporn Wacharapluesadee; Thiravat Hemachudha; Watsamon Jantarabenjakul; Weenassarin Ampoot; Yutthana Joyjinda
EPI_ISL_756311, EPI_ISL_1490226, EPI_ISL_1498262, EPI_ISL_2478947, EPI_ISL_2478949, EPI_ISL_2478954	The Caribbean Public Health Agency	Carrington Lab, Department of PreClinical Sciences, Faculty of Medical Sciences, The University of the West Indies	Adesh Ramsubhag; Anushka Ramjag; Arianne Brown-Jordan; Avery Hinds; Chinna Chinnadurai; Christine V. F. Carrington; Christopher Oura; Gabriel Escobar; Jaya Jayaraman; Jerome Foster; Karla Georges; Marsha Ivey; Naresh Nandram; Nikita S. D. Sahadeo; Nuno Faria; Oliver Pybus; Rahul Naidu; Rajini Haraksingh; Risha Singh; Roshan Parasram; Sarah Hill; Sharon Belmar-George; Stanley Giddings; SueMin Nathaniel; Vernie Ramkissoon
EPI_ISL_2565070, EPI_ISL_2621693, EPI_ISL_2621699, EPI_ISL_2621701, EPI_ISL_2621702, EPI_ISL_2621709, EPI_ISL_2621718, EPI_ISL_2626661, EPI_ISL_2626685			
see above	The Caribbean Public Health Agency	Carrington Lab, Department of Preclinical Sciences, Faculty of Medical Sciences, The University of the West Indies	Anushka Ramjag; Arianne Brown-Jordan; Avery Hinds; Christine V. F. Carrington; Christopher Oura; Gabriel Escobar; Karla Georges; Naresh Nandram; Nikita S. D. Sahadeo; Nuno Faria; Oliver Pybus; Risha Singh; Roshan Parasram; Sarah Hill; Sharon Belmar-George; SueMin Nathaniel; Vernie Ramkissoon
EPI_ISL_2617930	The Caribbean Public Health Agency	Carrington Lab, Department of Preclinical Sciences, Faculty of Medical Sciences, The University of the West Indies, St Augustine Campus	Anushka Ramjag; Arianne Brown-Jordan; Avery Hinds; Christine V. F. Carrington; Christopher Oura; Gabriel Escobar; Karla Georges; Naresh Nandram; Nikita S. D. Sahadeo; Nuno Faria; Oliver Pybus; Risha Singh; Roshan Parasram; Sarah Hill; SueMin Nathaniel; Vernie Ramkissoon
EPI_ISL_1625630, EPI_ISL_2562123	The Jackson Laboratory	The Jackson Laboratory	Adams M; Bergeron D; Kelly K; Li L; Long J; Omerza G; Renzette N
EPI_ISL_2521999	The Laboratory of Biosafety with Pathogens Collection The Republican Research and Practical Center for Epidemiology and Microbiology (RRPCEM)	Laboratory for HIV and opportunistic infections diagnosis The Republican Research and Practical Center for Epidemiology and Microbiology (RRPCEM)	Anatoli Krasko; Artur Akhremchuk; Elena Gasich; Ilarion Kupryianav; Iryna Subotsina; Kirill Bulda; Larysa Radzienava; Leonid Valentovich; Olga Klimovich; Olga Saleuskaya; Sergei Semenov
EPI_ISL_2189031	The Medical City	Philippine Genome Center	Alethea R. de Guzman; Anna Ong-Lim; Arianne A. Zamora; Asia Louisa U. Chong; Benedict A. Maralit; Candice Francheska B. Tambaoan; Carlo M. Lapid; Celia Carlos; Devon Ray Pacial; Edsel Maurice Salvaña; El King D. Morado; Elcid Aaron R. Pangilinan; Eva Maria Cutiongco-de la Paz; Francis A. Tablizo; Irish Coleen A. Asin; Jaime C. Montoya; Jan Michael C. Yap; Jo-Hannah S. Llames; John Q. Wong; Joshua Gregor A. Dizon; Juan Antonio R. Magalang; Karol Sophia Agape R. Padilla; Kenneth M. Kim; Kris P. Punayan; Marc Edsel C. Ayes; Maria Rosario Singh-Vergeire and Cynthia P. Saloma; Maria Sofia L. Yangzon; Marissa Alejandria; Razel Nikka M. Hao; Renato Jacinto Q. Mantaring; Rianna Patricia S. Cruz; Sheila Mae M. Araiza
EPI_ISL_891224, EPI_ISL_891227	The Oncology Institute "Prof. Dr. Ion Chiricuta" Cluj Napoca	"Stefan cel Mare" University Metagenomics Lab	Gheorghita Roxana; Lobiuc Andrei
EPI_ISL_2228242, EPI_ISL_2454305	TriCore Reference Laboratories	Center for Global Health, University of New Mexico Health Sciences Center	Cecilia Thompson; Darrell Dinwiddie; Daryl Domman; Karissa Culbreath; Kendra Pesko; Kurt Schwalm; Valerie Morley
EPI_ISL_2598111	TriCore Reference Laboratories	Center for Global Health, University of New Mexico Health Sciences Center	Cecilia Thompson; Darrell Dinwiddie; Daryl Domman; Karissa Culbreath; Kendra Pesko; Kurt Schwalm; Valerie Morley
EPI_ISL_717695, EPI_ISL_717700, EPI_ISL_756362, EPI_ISL_1499115, EPI_ISL_2230680, EPI_ISL_2230681, EPI_ISL_2230682, EPI_ISL_2230686			
see above	Trinidad Public Health Laboratory	Carrington Lab, Department of PreClinical Sciences, Faculty of Medical Sciences, The University of the West Indies	Adesh Ramsubhag; Arianne Brown-Jordan; Avery Hinds; Chinna Chinnadurai; Christine V. F. Carrington; Christopher Oura; Gabriel Escobar; Jaya Jayaraman; Jerome Foster; Karla Georges; Marsha Ivey; Naresh Nandram; Nikita S. D. Sahadeo; Nuno Faria; Oliver Pybus; Rahul Naidu; Rajini Haraksingh; Risha Singh; Roshan Parasram; Sarah Hill; Stanley Giddings; SueMin Nathaniel; Vernie Ramkissoon
EPI_ISL_2545667	Tripler Army Medical Center	Tripler Army Medical Center	Jonathan A. D'Ambrozio; Kendra N. Carter; Lindsey L. O'Neal; Rachael L. Downham; Zakariae Bram; and Catherine F. Uyehara
EPI_ISL_1298482	Tysmenytsia CH	The Institute of Molecular Biology and Genetics of NASU	M.Tukalo et al.
EPI_ISL_1993936, EPI_ISL_2099526	UMC Groningen, Clinical Virology, Department of Medical Microbiology and Infection Prevention	UMC Groningen, Clinical Virology, Department of Medical Microbiology and Infection Prevention	Alexander Friedrich; Coretta Van Leer-Buter; Erley Lizarazo-Forero; Hubert Niesters; Lilli Gard; Marjolein Knoester; Monika Fliss; Sigrid Rosema; Xuewei Zhou
EPI_ISL_2628728, EPI_ISL_2628976, EPI_ISL_2645940, EPI_ISL_2810609, EPI_ISL_2810702, EPI_ISL_2810726	UNILABS	Instituto Nacional de Saude (INSA)	Borges et al
EPI_ISL_2796475	UNILABS	Instituto Nacional de Saude (INSA) and BioSystems & Integrative Sciences Institute (BioISI) Genomics Unit, FCUL	Borges et al
EPI_ISL_2796175	UNILABS	Instituto Nacional de Saude (INSA) and Instituto Gulbenkian de Ciencia (IGC)	Borges et al
EPI_ISL_454410	UPMC Clinical Microbiology Laboratory	Microbial Genome Sequencing Center, Microbial Genomic Epidemiological Laboratory	Chinelo Ezeonwuku; Dan Snyder; Jane W. Marsh; Kady D. Waggle; Lee H. Harrison; Marissa P. Griffith; Mustapha M. Mustapha; Stephanie L. Mitchell; Vatsala R. Srinivasa; Vaughn S. Cooper
EPI_ISL_1091707, EPI_ISL_1260717, EPI_ISL_1711118, EPI_ISL_2383439, EPI_ISL_2383443, EPI_ISL_2450416	US Air Force School of Aerospace Medicine	US Air Force School of Aerospace Medicine	Amanda Javorina; Anthony Fries; Carol Garrett; Clarise Starr; Cole Anderson; Elizabeth Macias; Fritz Castillo; Jennifer Meyer; Sarah Purves; William Buggele; William Gruner
EPI_ISL_436045	US VI Department of Health	Pathogen Discovery, Respiratory Viruses Branch, Division of	Anna Uehara; Bettina Bankamp; Clinton R. Paden; Haibin Wang; Jing Zhang; Krista Queen; Suxiang Tong; Yan Li; Ying Tao; Zachary Weiner

		Viral Diseases, Centers for Disease Control and Prevention	
EPI_ISL_2260379	USC Clinical Lab	Los Angeles County PHL	P. Hemarajata et al.
EPI_ISL_2018388	UT-Unified State Labs: Public Health Utah DOH	Centers for Disease Control and Prevention Division of Viral Diseases, Pathogen Discovery	Alison Laufer Halpin; Ben L. Rambo-Martin; Clinton R. Paden; Dakota Howard; Darlene Wagner; Dave Wentworth; Dhwani Batra; Jasmine Padilla; Justin Lee; Katie Dillon; Krista Queen; Kristen Knipe; Kristine Lacek; Mark Burroughs; Matthew Schmerer; Mili Sheth; Peter Cook; Sam Shepard; Sarah Nobles; Shoshona Le; Suxiang Tong; Vivien Dugan; Yvette Unoarumhi
EPI_ISL_418037, EPI_ISL_1620980, EPI_ISL_1628185, EPI_ISL_1670711, EPI_ISL_1670827, EPI_ISL_1679851, EPI_ISL_1715449, EPI_ISL_1715512, EPI_ISL_1715530, EPI_ISL_1715565, EPI_ISL_1785682, EPI_ISL_1789323, EPI_ISL_1789403, EPI_ISL_1789408, EPI_ISL_1789424, EPI_ISL_1789426, EPI_ISL_1826129, EPI_ISL_1826132, EPI_ISL_1827723, EPI_ISL_1849851, EPI_ISL_1850287, EPI_ISL_2002190, EPI_ISL_2002289, EPI_ISL_2002395, EPI_ISL_2002689, EPI_ISL_2002743, EPI_ISL_2002774, EPI_ISL_2002825, EPI_ISL_2002836, EPI_ISL_2002857, EPI_ISL_2002865, EPI_ISL_2002878, EPI_ISL_2002924, EPI_ISL_2031271, EPI_ISL_2031294, EPI_ISL_2031501, EPI_ISL_2031502, EPI_ISL_2031503, EPI_ISL_2031512, EPI_ISL_2031536, EPI_ISL_2037457, EPI_ISL_2037467, EPI_ISL_2037490, EPI_ISL_2037527, EPI_ISL_2037537, EPI_ISL_2037573, EPI_ISL_2037581, EPI_ISL_2037634, EPI_ISL_2037663, EPI_ISL_2082516, EPI_ISL_2082680, EPI_ISL_2086392, EPI_ISL_2086411, EPI_ISL_2104962, EPI_ISL_2233209, EPI_ISL_2497395, EPI_ISL_2502898, EPI_ISL_2502925, EPI_ISL_2502986, EPI_ISL_2503005, EPI_ISL_2503017, EPI_ISL_2503018, EPI_ISL_2503049, EPI_ISL_2503087, EPI_ISL_2503106, EPI_ISL_2545145, EPI_ISL_2549418, EPI_ISL_2618118, EPI_ISL_2618216, EPI_ISL_2618264, EPI_ISL_2618292, EPI_ISL_2618387, EPI_ISL_2618402, EPI_ISL_2618579, EPI_ISL_2802977			
see above	UW Virology Lab	UW Virology Lab	Alexander Greninger; Hong Xie; Keith Jerome; Keith R Jerome; Lasata Shrestha; Meei-Li Huang; Michelle Lin; Nathan Breit; Noah R. Baker; Patrick Mathias; Pavitra Roychoudhury; Ricardo Perez; Robert J. Livingston; Saraswathi Sathees; Sean Ellis; Shah Mohamed Bakhash; Tien V. Nguyen
EPI_ISL_737951	Uganda Central Public Health Lab and Uganda Virus Research Institute	MRC/UVRI & LSHTM Uganda Research Unit	Dan Lule Bugembe; Matthew Cotten; My V.T. Phan; Pontiano Kaleebu et al.
EPI_ISL_451199	Uganda Virus Research Institute	MRC/UVRI & LSHTM Uganda Research Unit	Beatrice Dhaala; Dan Lule Bugembe; Deogratius Ssemwanga; Henry Kyobe; Henry Mwebesa; Jane Aceng; John Kayiwa; Jonas Lexow; Julius Lutwama; Matthew Cotten; My V.T Phan; Phionah Tushabe; Pontiano Kaleebu; Stephen Balinandi
EPI_ISL_1662223	Unidad de Investigacion Biomedica de Zacatecas (UIBZ)	Unidad de Genomica Avanzada	Alejandro Sanchez-Flores; Alfredo Herrera-Estrella; Alicia Ocana-Mondragon; Angel Gustavo Salas-Lais; Bernardo Martinez-Miguel; Blanca Taboada; Brenda Irasema Maldonado-Meza; Carla Ivon Herrera-Najera; Carlos F. Arias; Celia Boukadida; Celida Duque Molina; Clara Esperanza Santacruz-Tinoco; Concepcion Grajales-Muniz; Consorcio Mexicano de Vigilancia Genomica (CoViGen-Mex). Authors (in alphabetical order): Julio Elias Alvarado-Yaah; Fernando Fontove-Herrera; Francisco Pulido; Gloria Elena Espinosa-Ayala; Gloria Maria Molina-Salinas; Gloria Vazquez; Hector Esteban Paz-Juarez; Hector Montoya-Fuentes; Helen Haydee Fernanda Ramirez-Plascencia; Jose Antonio Enciso-Moreno; Jose Esteban Munoz-Medina; Jose de Jesus Nunez-Contreras; Juan Bautista Chale-Dzul; Luis Alberto Ochoa-Carrera; Margarita Matias-Florentino; Maria Guadalupe Santiago-Mauricio; Maria Guadalupe de Jesus Mireles-Rivera; Nelly Selem-Mojica; Pavel Isa; Ricardo Grande; Santiago Ávila-Rios; Victor Eduardo Garcia-Arias; Victor Hugo Borja-Aburto
EPI_ISL_1351468, EPI_ISL_1351489, EPI_ISL_1351492, EPI_ISL_1351494, EPI_ISL_1351498	Unidad de InvestigaciÃ³n MÃ©dica de YucatÃ¡n (UIMY)	Unidad de Genomica Avanzada	Alejandro Sanchez-Flores; Alfredo Herrera-Estrella; Alicia Ocana-Mondragon; Angel Gustavo Salas-Lais; Bernardo Martinez-Miguel; Blanca Taboada; Brenda Irasema Maldonado-Meza; Carla Ivon Herrera-Najera; Carlos F. Arias; Celia Boukadida; Clara Esperanza Santacruz-Tinoco; Concepcion Grajales-Muniz; Consorcio Mexicano de Vigilancia Genomica (CoViGen-Mex). Authors (in alphabetical order): Julio Elias Alvarado-Yaah; Fernando Fontove-Herrera; Francisco Pulido; Gloria Elena Espinoza-Ayala; Gloria Maria Molina-Salinas; Gloria Vazquez; Hector Esteban Paz-Juarez; Hector Montoya-Fuentes; Helen Haydee Fernanda Ramirez-Plascencia; Jorge Ivan Salinal-Nevarez; Jose Antonio Enciso-Moreno; Jose Esteban Munoz-Medina; Jose de Jesus Nunez-Contreras; Juan Bautista Chale-Dzul; Luis Alberto Ochoa-Carrera; Margarita Matias-Florentino; Maria Guadalupe Santiago-Mauricio; Maria Guadalupe de Jesus Mireles-Rivera; Nelly Selem-Mojica; Pavel Isa; Ricardo Grande; Santiago Avila-Rios; Victor Hugo Borja-Aburto
EPI_ISL_2490502, EPI_ISL_2490510	Unidad de Investigación Biomédica de Zacatecas (UIBZ)	Centro de Investigación en Enfermedades Infecciosas (CIENI), Instituto Nacional de Enfermedades Respiratorias (INER)	; Alejandra Garcia-Gasca; Alejandra Hernández-Terán; Alejandro Sanchez-Flores; Alfredo Herrera-Estrella; Alicia Ocaña-Mondragón; Andreu Comas-García; Angel Gustavo Salas-Lais; Antonio Loza Román; Bernardo Martínez-Miguel; Blanca Taboada; Brenda Irasema Maldonado-Meza; Bruno Gomez-Gil; Carla Ivón Herrera-Najera; Carlos F. Arias; Celia Boukadida; Clara Esperanza Santacruz-Tinoco; Concepción Grajales-Muñiz; Consorcio Mexicano de Vigilancia Genómica (CoViGen-Mex). Authors (in alphabetical order): Julio Elias Alvarado-Yaah; Cristóbal Cháidez-Quiróz; Célida Duque Molina; Célida Martínez- Rodríguez; Daniel Fregoso-Rueda; Daniel Lira Morales; Eduardo Becerril-Vargas; Fernando Fontove-Herrera; Fidencio Mejía-Nepomuceno; Francisco Pulido; Gloria Elena Espinoza-Ayala; Gloria María Molina-Salinas; Gloria Vazquez; Hector Esteban Paz-Juárez; Hector Montoya-Fuentes; Helen Haydee Fernanda Ramírez-Plascencia; Irvin González-López; Jean Pierre González; Joel Armando Vázquez-Pérez.; Jorge Salas-Hernández; José Antonio Enciso-Moreno; José Arturo Martínez-Orozco; José Esteban Muñoz-Medina; José de Jesús Nuñez-Contreras; Juan Bautista Chale-Dzul; Julissa Enciso-Ibarra; Luis Alberto Ochoa-Carrera; Margarita Matías-Florentino; Mario Mújica-Sánchez; Marissa Perez-Garcia; María Guadalupe Santiago-Mauricio; María Guadalupe de Jesús Mireles-Rivera; Nelly Sélem-Mojica; Pavel Isa; Ricardo Ciria Merce; Ricardo Grande; Rosa María Gutierrez Rios; Santiago Ávila-Ríos; Selene Zárate; Susana Lopez; Victor Eduardo Garcia-Arias; Víctor Hugo Borja-Aburto
EPI_ISL_1279365, EPI_ISL_1279374	Unidad de Investigación Biomédica de Zacatecas (UIBZ)	Instituto Nacional de Enfermedades Respiratorias (INER): Centro de Investigación en Enfermedades Infecciosas (CIENI)	Alejandro Sanchez-Flores; Alfredo Herrera-Estrella; Alicia Ocaña-Mondragón; Angel Gustavo Salas-Lais; Bernardo Martínez-Miguel; Blanca Taboada; Brenda Irasema Maldonado-Meza; Carla Ivón Herrera-Najera; Carlos F. Arias; Celia Boukadida; Clara Esperanza Santacruz-Tinoco; Concepción Grajales-Muñiz; Consorcio Mexicano de Vigilancia Genómica (CoViGen-Mex). Authors (in alphabetical order): Julio Elias Alvarado-Yaah; Fernando Fontove-Herrera; Francisco Pulido; Gloria Elena Espinoza-Ayala; Gloria María Molina-Salinas; Gloria Vazquez; Hector Esteban Paz-Juárez; Hector Montoya-Fuentes; Helen Haydee Fernanda Ramírez-Plascencia; Jorge Ivan Salinal-Nevarez; José Antonio Enciso-Moreno; José Esteban Muñoz-Medina; José de Jesús Nuñez-Contreras; Juan Bautista Chale-Dzul; Luis Alberto Ochoa-Carrera; Margarita Matías-Florentino; María Guadalupe Santiago-Mauricio; María Guadalupe de Jesús Mireles-Rivera; Nelly Sélem-Mojica; Pavel Isa; Ricardo Grande; Santiago Ávila-Ríos; Víctor Hugo Borja-Aburto
EPI_ISL_2402096	Unidad de Investigación Biomédica de Zacatecas (UIBZ)	Unidad de Genomica Avanzada	Alejandro Sanchez-Flores; Alfredo Herrera-Estrella; Alicia Ocana-Mondragon; Angel Gustavo Salas-Lais; Bernardo Martinez-Miguel; Blanca Taboada; Brenda Irasema Maldonado-Meza; Carla Ivon Herrera-Najera; Carlos F. Arias; Celia Boukadida; Clara Esperanza Santacruz-Tinoco; Concepcion Grajales-Muniz; Consorcio Mexicano de Vigilancia Genomica (CoViGen-Mex). Authors (in alphabetical order): Julio Elias Alvarado-Yaah; Fernando Fontove-Herrera; Francisco Pulido; Gloria Elena Espinoza-Ayala; Gloria Maria Molina-Salinas; Gloria Vazquez; Hector Esteban Paz-Juarez; Hector Montoya-Fuentes; Helen Haydee Fernanda Ramirez-Plascencia; Jorge Ivan Salinal-Nevarez; Jose Antonio Enciso-Moreno; Jose Esteban Munoz-Medina; Jose de Jesus Nunez-Contreras; Juan Bautista Chale-Dzul; Luis Alberto Ochoa-Carrera; Margarita Matias-Florentino; Maria Guadalupe Santiago-Mauricio; Maria Guadalupe de Jesus Mireles-Rivera; Nelly Selem-Mojica; Pavel Isa; Ricardo Grande; Santiago Avila-Rios; Victor Hugo Borja-Aburto
EPI_ISL_2091244	Unidad de Investigación Médica de Yucatán (UIMY)	Centro de Investigación en Enfermedades Infecciosas (CIENI), Instituto Nacional de Enfermedades Respiratorias (INER)	Alejandro Sanchez-Flores; Alfredo Herrera-Estrella; Alicia Ocaña-Mondragón; Angel Gustavo Salas-Lais; Bernardo Martínez-Miguel; Blanca Taboada; Brenda Irasema Maldonado-Meza; Carla Ivón Herrera-Najera; Carlos F. Arias; Celia Boukadida; Clara Esperanza Santacruz-Tinoco; Concepción Grajales-Muñiz; Consorcio Mexicano de Vigilancia Genómica (CoViGen-Mex). Authors (in alphabetical order): Julio Elias Alvarado-Yaah; Célida Duque Molina; Fernando Fontove-Herrera; Francisco Pulido; Gloria Elena Espinosa-Ayala; Gloria María Molina-Salinas; Gloria Vazquez; Hector Esteban Paz-Juárez; Hector Montoya-Fuentes; Helen Haydee Fernanda Ramírez-Plascencia; José Antonio Enciso-Moreno; José Esteban Muñoz-Medina; José de Jesús Nuñez-Contreras; Juan Bautista Chale-Dzul; Luis Alberto Ochoa-Carrera; Margarita Matías-Florentino; María Guadalupe Santiago-Mauricio; María Guadalupe de Jesús Mireles-Rivera; Nelly Sélem-Mojica; Pavel Isa; Ricardo Grande; Santiago Ávila-Ríos; Victor Eduardo Garcia-Arias; Víctor Hugo Borja-Aburto
EPI_ISL_1585434, EPI_ISL_1585443	Unidad de Investigación Médica de Yucatán (UIMY)	Instituto Nacional de Enfermedades Respiratorias (INER): Centro de Investigación en Enfermedades Infecciosas (CIENI)	Alejandro Sanchez-Flores; Alfredo Herrera-Estrella; Alicia Ocaña-Mondragón; Angel Gustavo Salas-Lais; Bernardo Martínez-Miguel; Blanca Taboada; Brenda Irasema Maldonado-Meza; Carla Ivón Herrera-Najera; Carlos F. Arias; Celia Boukadida; Clara Esperanza Santacruz-Tinoco; Concepción Grajales-Muñiz; Consorcio Mexicano de Vigilancia Genómica (CoViGen-Mex). Authors (in alphabetical order): Julio Elias Alvarado-Yaah; Célida Duque Molina; Fernando Fontove-Herrera; Francisco Pulido; Gloria Elena Espinosa-Ayala; Gloria María Molina-Salinas; Gloria Vazquez; Hector Esteban Paz-Juárez; Hector Montoya-Fuentes; Helen Haydee Fernanda Ramírez-Plascencia; José Antonio Enciso-Moreno; José Esteban Muñoz-Medina; José de Jesús Nuñez-Contreras; Juan Bautista Chale-Dzul; Luis Alberto Ochoa-Carrera; Margarita Matías-Florentino; María Guadalupe Santiago-Mauricio; María Guadalupe de Jesús Mireles-Rivera; Nelly Sélem-Mojica; Pavel Isa; Ricardo Grande; Santiago Ávila-Ríos; Victor Eduardo Garcia-Arias; Víctor Hugo Borja-Aburto
EPI_ISL_2426083	Unilabs Laboratory Medicine	Norwegian Institute of Public Health, Department of Virology	Atiya R Ali; Debech Nadia; Engebretsen Serina Beate; Garcia Llorente Ignacio; Hilde Elshaug; Hilde Vollan; Jon Bråte; Kamilla Heddeland Instefjord; Karoline Bragstad; Kathrine Stene-Johansen; Line Victoria Moen; Marie Paulsen Madsen; Olav Hungnes; Pedersen Benedikte Nevjen; Rasmus Riis Kopperud
EPI_ISL_1260721	United States Air Force Academy	US Air Force School of Aerospace Medicine	Amanda Javorina; Anthony Fries; Clarise Starr; Elizabeth Macias; Jennifer Meyer; Sarah Purves; William Buggele; William Gruner
EPI_ISL_812657, EPI_ISL_831873	United States Air Force School of Aerospace Medicine	United States Air Force School of Aerospace Medicine	Amanda Javorina; Anthony Fries; Clarise Starr; Elizabeth Macias; Jennifer Meyer; Sarah Purves; William Gruner
EPI_ISL_1035988	Universidad de León	SeqCOVID-SPAIN consortium/IBV(CSIC)	Ana Carvajal; Antonio J. Molina and SeqCOVID-SPAIN consortium; Héctor Argüello; Juan M. Fregeneda; Tania Fernández-Villa; Vicente Martín
EPI_ISL_812966	University Clinical Research Center, University of Sciences	University Clinical Research Center, University of Sciences	A.A.; Bane, S.; Dao, S.; Diakite, M.; Diarra, B.; Doumbia, S.; Guindo, I.; Iknane; Kone, A.
EPI_ISL_581913, EPI_ISL_581996, EPI_ISL_1388962	University Hospital Basel, Clinical Virology	University Hospital Basel, Clinical Bacteriology	Adrian Egli; Alexander Gensch; Alfredo Mari; Christian Nickel; Hans Hirsch; Hans Pargger; Helena MB Seth-Smith; Julia Bielicki; Karoline Leuzinger; Kirstine K. Soegaard; Madlen Stange; Manuel Battegay; Martin Siegemund; Michael Osthoff; Michael Schweitzer; Myrta Brunner; Rita Schneider-Sliwa; Roland Bingisser; Sarah Tschudin-Sutter; Simon Fuchs; Stefano Bassetti; Tim Roloff
EPI_ISL_1240790	University Hospitals Translational Laboratory (UHTL), University Hospitals	University Hospitals Translational Laboratory (UHTL), University Hospitals	Alouani, D.; Sadri, N.; Song, X.
EPI_ISL_1040386, EPI_ISL_1533218, EPI_ISL_1811202, EPI_ISL_1963016, EPI_ISL_1963298, EPI_ISL_1963299, EPI_ISL_1963300, EPI_ISL_2104479, EPI_ISL_2104494, EPI_ISL_2213579, EPI_ISL_2213582, EPI_ISL_2343033, EPI_ISL_2343125, EPI_ISL_2601080			
see above	University Hospitals of Geneva, Laboratory of Virology	HUG, Laboratory of Virology and the Health2030 Genome Center	Ana Rita Goncalves; Deborah Penet; Emmanouil Dermitzakis; Henri Pegeot; Ioannis Xenarios; Keith Harshman; Laurent Kaiser; Lorenzo Cerutti; Melyssa Elies; Samuel Cordey
EPI_ISL_2100469, EPI_ISL_2100480, EPI_ISL_2100482	University of Health Sciences	Quadram Institute Bioscience	Alam Khan; Alexander J Trotter; Alison E. Mather; Almina Shafiq; Alp Aydin; Ana P. Tedim; Anastasia Kolyva; Andrew Bell; Andrew J. Page; Claire Stuart; Dave J. Baker; Gemma L. Kay; Javed Akram; John Wain; Justin O'Grady; Leonardo de Oliveira Martins; Lizzie Meadows; Maria Diaz; Mark Webber; Muhammad Bilal Sarwar; Muhammad Roman; Muhammed Yasir; Nabil-Fareed Alikhan; Nadeem Afzal; Ngozi Elumogo; Nicholas M. Thomson; Rachael Stanley; Rachel Gilroy; Reenesh Prakash; Samir Dervisevic; Samuel Bloomfield; Shah Jahan; Sidra-tul-muntaha; Steven Rudder; Thanh Le-Viet
EPI_ISL_976938	University of Massachusetts Medical School	Infectious Disease Program, Broad Institute of Harvard and MIT	Adams, G.; B.L.; B.W.; Bauer, M.; Birren; Carter, A.; Chaluvadi, S.; D.J.; DeRuff, K.; Ellison, R.; Gallagher, G.; Gladden-Young, A.; J.E.; K.J.; Lagerborg, K.; Lemieux; Loreth, C.; MacInnis; Normandin, E.; P.C.; Park; Reilly, S.; Rudy, M.; Siddle; Smole, S.; Tomkins-Tinch, C.; Ward, D.; and Sabeti
EPI_ISL_896374	University of Medicine and Pharmacy of Craiova	"Stefan cel Mare" University Metagenomics Lab	Gheorghita Roxana; Lobiuc Andrei
EPI_ISL_590727, EPI_ISL_1919930, EPI_ISL_2001270, EPI_ISL_2001286, EPI_ISL_2100385, EPI_ISL_2612297	University of Michigan Clinical Microbiology Laboratory	Lauring Lab, University of Michigan, Department of Microbiology and Immunology	Valesano
EPI_ISL_1527249, EPI_ISL_2333917, EPI_ISL_2497893, EPI_ISL_2497894	University of Mississippi Medical Center, Department of Pathology	University of Mississippi Medical Center, Molecular and Genomics Core Facility	Ashley C. Johnson; D. Ashley Robinson; Ithiel J. Frame; Krishna K. Ayyalasomayajula; Michael R. Garrett
EPI_ISL_2382313	University of Pittsburgh	Genomics and Discovery, Respiratory Viruses Branch, Division of Viral Diseases, Centers for Disease Control and Prevention	Adam Retchless; Anna Kelleher; Anna Uehara; Brian Lynch; Clinton R. Paden; Dhwani Batra; Haibin Wang; Han Jia Justin Ng; Jasmine Padilla; Jing Zhang; Justin Lee; Krista Queen; Mark Burroughs; Mili Sheth; Morgan Davis; Peter Cook; Rachel Marine; Sarah Nobles; Suxiang Tong; Tara Coalter; Yan Li; Ying Tao
EPI_ISL_416491, EPI_ISL_1064293, EPI_ISL_1112214, EPI_ISL_1403817, EPI_ISL_1493127	University of Wisconsin-Madison AIDS Vaccine Research Laboratories	University of Wisconsin-Madison AIDS Vaccine Research Laboratories	Gage Moreno; Katarina Braun; et al. AIDS Vaccine Research Laboratories
EPI_ISL_977331, EPI_ISL_977421, EPI_ISL_977463, EPI_ISL_977469, EPI_ISL_2803691	University of Zambia, School of Veterinary Medicine	UNZAVET and PATH	Daniel Bridges; Mulenga Mwenda-Chimfwembe; Ngonda Saasa; ZNPHI and ZGSC
EPI_ISL_856695	University of Zambia, School of Veterinary Medicine, Disease Control	University of Zambia, School of Veterinary Medicine, Disease Control	A.L.; A.N.; Bates, M.; C. and Zumla, A.; Chambaro, H.; Chanda, D.; Changula, K.; Chilufya; Chipimo; Chitanga, S.; Fwoloshi, S.; K.S.; Kapata; Kapata, N.; Kapaya, F.; Kapin'a, M.; Kayeyi, N.; Liwewe; M.M.; Malama, K.; Masahiro, K.; Monze, M.; Morales; Mubemba, B.; Mukonka, V.; Mulenga, L.; Muleya, W.; Mupeta, F.; Musonda, K.; Nalubamba; Ngosa, W.; P.C.; P.J.; Saasa, N.; Sawa, H.; Shibemba; Simulundu, E.; Sinyange, N.; Takada, A.; Tembo, J.; Zulu, P.
EPI_ISL_2382031	Universität Innsbruck, Institut für Mikrobiologie	Bergthaler laboratory, CeMM Research Center for Molecular Medicine of the Austrian Academy of Sciences	Andreas Bergthaler; Anna Schedl; Bekir Erguner; Benedikt Agerer; Christoph Bock; Fabian Amman; Jan Laine; Lukas Endler; Maelle Le Moing; Martin Senekowitsch; Michael Schuster; Petr Triska; Thomas Penz

EPI_ISL_738358, EPI_ISL_2279989, EPI_ISL_2284693, EPI_ISL_2290872, EPI_ISL_2291743, EPI_ISL_2291745, EPI_ISL_2291749, EPI_ISL_2291802, EPI_ISL_2292170, EPI_ISL_2292414, EPI_ISL_2292490, EPI_ISL_2292612, EPI_ISL_2292738, EPI_ISL_2292937, EPI_ISL_2292941, EPI_ISL_2292972, EPI_ISL_2458375, EPI_ISL_2458500, EPI_ISL_2496656, EPI_ISL_2679564			
see above	Utah Public Health Laboratory	Utah Public Health Laboratory	Erin L. Young; Erin Young; Kelly F. Oakeson; Kelly Oakeson; Tara Gallagher
EPI_ISL_2384224, EPI_ISL_2384227, EPI_ISL_2384231, EPI_ISL_2452023, EPI_ISL_2452025	VI-US Virgin Islands Department of Health	Centers for Disease Control and Prevention Division of Viral Diseases, Pathogen Discovery	Alison Laufer Halpin; Ben L. Rambo-Martin; Clinton R. Paden; Dakota Howard; Darlene Wagner; Dave Wentworth; Dhwani Batra; Jasmine Padilla; Justin Lee; Katie Dillon; Krista Queen; Kristen Knipe; Kristine Lacek; Mark Burroughs; Matthew Schmerer; Mili Sheth; Peter Cook; Sam Shepard; Sarah Nobles; Shoshona Le; Suxiang Tong; Vivien Dugan; Yvette Unoarumhi
EPI_ISL_452138	VI-US Virgin Islands Department of Health	Pathogen Discovery, Respiratory Viruses Branch, Division of Viral Diseases, Centers for Disease Control and Prevention	Anna Montmayeur; Anna Uehara; Bettina Bankamp; Clinton R. Paden; Haibin Wang; Jing Zhang; Krista Queen; Rachel Marine; Suxiang Tong; Yan Li; Ying Tao; Zachary Weiner
EPI_ISL_2090613, EPI_ISL_2090614	VIROLOGY, AFRIMS	VIROLOGY, AFRIMS	A.T.; Chinnawirotpisan, P.; Chua; Claire Navarro, F.; Corazon Diones, P.; D. Jr.; Fernandez, S.; H. Jr.; Huang; J.M.; Jones, A.; Joonlasak, K.; Klungthong, C.; M.T.; Manasatienkij, W.; Tabinas; V. II; Valderama; Velasco; Villa
EPI_ISL_1824682, EPI_ISL_1824683, EPI_ISL_1824684	VITHAS Lab	Microbiology Department. Complexo Hospitalario Universitario de Vigo	Camacho AT; Perez S; Regueiro B
EPI_ISL_2404806, EPI_ISL_2404815	VMC	Santa Clara County public Health Laboratory	Santa Clara County Public Health Department
EPI_ISL_903635, EPI_ISL_903657	VT Dept. of Health Laboratory	Genomics and Discovery, Respiratory Viruses Branch, Division of Viral Diseases, Centers for Disease Control and Prevention	Anna Montmayeur; Anna Uehara; Ben L. Rambo-Martin; Clinton R. Paden; Dhwani Batra; Haibin Wang; Jasmine Padilla; Jing Zhang; Justin Lee; Krista Queen; Lori Rowe; Mark Burroughs; Mili Sheth; Peter W. Cook; Rachel Marine; Sarah Nobles; Suxiang Tong; Yan Li; Ying Tao
EPI_ISL_2333568	Vestfold Hospital, Toensberg Department of Microbiology	Norwegian Institute of Public Health, Department of Virology	'Kathrine Stene-Johansen; Atiya R Ali; Debech Nadia; Engebretsen Serina Beate; Garcia Llorente Ignacio; Hilde Elshaug; Hilde Vollan; Jon Bråte; Kamilla Heddeland Instefjord; Karoline Bragstad; Line Victoria Moen; Marie Paulsen Madsen; Olav Hungnes; Pedersen Benedikte Nevjen; Rasmus Riis Kopperud
EPI_ISL_833511	Veterinary Specialized Instute "Nis"	Veterinary Specialized Institute "Kraljevo", Serbia	Afonso, C.; Banovic Djeri, B.; Jankovic, M.; Jovanovic, T.; Knezevic, A.; Manic, M.; Petrovic, M.; Petrovic, T.; Sekler, M.; Tesovic, B.; Vidanovic, D.; Volkening, J.
EPI_ISL_2227287	Veterinary institute in Zvolen, Pod Drahami 918, 960 86 Zvolen Slovakia	Veterinary institute in Zvolen, Pod Drahami 918, 960 86 Zvolen Slovakia	Dirbáková Z.; Mojžiš M.; Mokryšová S.; Sujová S.; Tinák M.
EPI_ISL_1658609, EPI_ISL_1658683, EPI_ISL_1914236, EPI_ISL_1914237, EPI_ISL_1914238, EPI_ISL_1914239	Viollier AG	Department of Biosystems Science and Engineering, ETH Zurich	Andrea Patrignani; Andreia Cabral de Gouvea; Catharine Aquino; Chaoran Chen; Christian Beisel; Christiane Beckmann; Christoph Noppen; David Dreifuss; Deborah Penet; Doris Popovic; Elodie Burcklen; Emmanouil Dermitzakis; Griffin White; Henri Pegeot; Ina Nissen; Ioannis Xenarios; Ivan Topolsky; Jay Tracy; Katharina Jahn; Keith Harshman; Lara Fuhrmann; Laura Neff; Lennart Opitz; Lorenzo Cerutti; Maria Domenica Moccia; Maurice Redondo; Mirjam Feldkamp; Natascha Santacroce; Niko Beerenwinkel; Noemie Santamaria de Souza; Olivier Kobel; Philipp Jablonski; Ralph Schlapbach; Rebecca Denes; Sarah Nadeau; Simon Gruter; Sophie Seidel; Tanja Stadler; Timothy Sykes
EPI_ISL_483654, EPI_ISL_486505, EPI_ISL_728915, EPI_ISL_900047, EPI_ISL_1120044, EPI_ISL_1259693, EPI_ISL_1598642, EPI_ISL_1750690, EPI_ISL_2017208, EPI_ISL_2017223, EPI_ISL_2017224, EPI_ISL_2017225, EPI_ISL_2018995, EPI_ISL_2019465, EPI_ISL_2019535, EPI_ISL_2212778, EPI_ISL_2307500, EPI_ISL_2307730, EPI_ISL_2308061, EPI_ISL_2375070, EPI_ISL_2462613, EPI_ISL_2662435, EPI_ISL_2724396			
see above	Viollier AG	Department of Biosystems Science and Engineering, ETH Zürich	Andrea Patrignani; Andrea Patrizia Salzmann; Andreia Cabral de Gouvea; Catharine Aquino; Chaoran Chen; Christian Beisel; Christiane Beckmann; Christoph Noppen; Daniel Ehrsam; David Dreifuss; Deborah Penet; Doris Popovic; Elodie Burcklen; Emmanouil Dermitzakis; Griffin White; Henri Pegeot; Henriette Kurth; Ina Nissen; Ioannis Xenarios; Isabel Stürmer; Ivan Topolsky; Jay Tracy; Katharina Jahn; Keith Harshman; Kim Philipp Jablonski; Lara Fuhrmann; Laura Neff; Lennart Opitz; Lorenzo Cerutti; Maria Domenica Moccia; Maurice Redondo; Mirjam Feldkamp; Natascha Santacroce; Niko Beerenwinkel; Noemie Santamaria de Souza; Olivier Kobel; Pedro Ferreira; Philipp Jablonski; Ralph Schlapbach; Rebecca Denes; Sarah Nadeau; Simon Grüter; Sophie Seidel; Susana Posada-Céspedes; Tanja Stadler; Timothy Sykes; Tobias Schär
EPI_ISL_2017850, EPI_ISL_2100241, EPI_ISL_2100242	Viral Respiratory Infections Laboratory, Cantacuzino National Military-Medical Institute	Cantacuzino Institute Virology	Carmen Cherciu; Catalina Pascu; Luiza Ustea; Mihaela Lazar; Mihaela Oprea; Nicoleta Paraschiv; Sorin Dinu
EPI_ISL_452815, EPI_ISL_526906	Virginia DCLS	Virginia DCLS	Virginia DCLS
EPI_ISL_1805678, EPI_ISL_2003700, EPI_ISL_2081439, EPI_ISL_2603363	Virginia Division of Consolidated Laboratory Services	Virginia Division of Consolidated Laboratory Services	Virginia DCLS
EPI_ISL_2532755, EPI_ISL_2549198, EPI_ISL_2549199	Virology Department, Central Health Laboratory	The Francis Crick Institute	Bahadoor BS; Crawford M; Daniels RS; Goldstone R; Harvey R; Manraj SS; Nicod J; Patel H; Ramuth M; Sonoo J
EPI_ISL_1974856, EPI_ISL_2125642, EPI_ISL_2125659, EPI_ISL_2125661, EPI_ISL_2125662, EPI_ISL_2125711, EPI_ISL_2125713, EPI_ISL_2125715, EPI_ISL_2125717			
see above	Virology Department, Royal Infirmary of Edinburgh, NHS Lothian / School of Biological Sciences, University of Edinburgh	COVID-19 Genomics UK (COG-UK) Consortium	Colquhoun R; Cotton S; Dewar R; Gallagher A; Hill V; Jackson B; Maloney D; McCrone JT; McHugh M; O'Toole Á; Rambaut A; Rooke S; Scher E; Templeton K; Yu X
EPI_ISL_1827666, EPI_ISL_1827667, EPI_ISL_1827670, EPI_ISL_1827678, EPI_ISL_1827689	Virology Department, Victoria Hospital, Plaine-Wilhems, Mauritius	National Institute for Communicable Diseases of the National Health Laboratory Service	Allam M; Amoako DG; Baboo SB; Bhiman JN; Ismail A; Mahlangu B; Manraj SS; Mohale T; Ntuli N; Ramuth M; Sonoo J
EPI_ISL_2547367	Vredendal North Clinic wc VDN	National Health Laboratory Service/University of Cape Town (NHLS/UCT)	Arash Iranzadeh; Bruna Galvao; Carolyn Williamson; Deelan Doolabh; Diana Hardie; Gert Marais; Innocent Mudau; Lynn Tyers; Marvin Hsiao; Stephen Korsman
EPI_ISL_1789541, EPI_ISL_1789542, EPI_ISL_2385074, EPI_ISL_2385092, EPI_ISL_2385108, EPI_ISL_2385128, EPI_ISL_2385152, EPI_ISL_2450489, EPI_ISL_2523507, EPI_ISL_2523522, EPI_ISL_2523525, EPI_ISL_2523543			
see above	WHO National Influenza Centre Russian Federation	WHO National Influenza Centre Russian Federation	Alexey Masharsky; Andrey Komissarov; Artem Fadeev; Daria Danilenko; Dmitry Lioznov; Elena Nabieva; Georgii Bazykin; Kirill Varchenko; Ksenia Safina; Kseniya Komissarova; Maria Baturova; Maria Pisareva; Maria Timofeeva; Mikhail Bakaev; Nikita Yolshin; Oula Mansour; Oula Masour; Tamila Musaeva; Veronika Eder
EPI_ISL_1273790	WSSE Gdańsk	1. National Institute of Public Health - National Institute of Hygiene; 2. Eurofins Genomics Europe Sequencing GmbH	ECDC COVID-19 WGS support team; Eurofins Genomics Europe Sequencing Team; Gierczyńki Rafał; Sadkowska-Todys Małgorzata; Wołkowicz Tomasz; Zacharczuk Katarzyna
EPI_ISL_1909092, EPI_ISL_1909093, EPI_ISL_1909094, EPI_ISL_1909095	WSSE Katowice	1. Academic Center for Pathomorphological and Genetic-Molecular Diagnostics ltd, Bialystok, Poland 2. National Institute of Public Health - National Institute of Hygiene, Warsaw, Poland	Anetta Sulewska; Jacek Nikliński; Janusz Dzięcioł; Joanna Kiśluk; Katarzyna Zacharczuk; Konrad Raczkowski; Magdalena Nowakowska; Małgorzata Sadkowska-Todys; Piotr Karabowicz; Piotr Majewski; Przemysław Biecek. Joanna Reszeć; Radosław Charkiewicz; Tomasz Wołkowicz
EPI_ISL_2105608	WSSE w Gdansku	1. Academic Center for Pathomorphological and Genetic-Molecular Diagnostics ltd, Bialystok, Poland 2. National Institute of Public Health - National Institute of Hygiene, Warsaw, Poland	Anetta Sulewska; Jacek Nikliński; Janusz Dzięcioł; Joanna Kiśluk; Katarzyna Zacharczuk; Konrad Raczkowski; Magdalena Nowakowska; Małgorzata Sadkowska-Todys; Piotr Karabowicz; Piotr Majewski; Przemysław Biecek. Joanna Reszeć; Radosław Charkiewicz; Tomasz Wołkowicz
EPI_ISL_1908335	WSSE w Krakowie	83. National Institute of Public Health - National Institute of Hygiene, Warsaw, Poland 2. Biobank Lab, University of Lodz 3. Laboratory of Respiratory Viruses, Teaching and Clinical Center of the Medical University of Lodz	Dominik Strapagiel; Izabela Dróżdż; Jakub Lach; Katarzyna Zacharczuk; Klaudyna Królikowska; Maciej Borowiec; Magdalena Nowakowska; Magdalena Traczyk-Borszyńska; Marcin Słomka; Marta Sobalska-Kwapis; Małgorzata Sadkowska-Todys; Tomasz Płoszaj; Tomasz Wołkowicz
EPI_ISL_2090620, EPI_ISL_2090621, EPI_ISL_2090622, EPI_ISL_2090623, EPI_ISL_2094439, EPI_ISL_2094440, EPI_ISL_2094441			
see above	WSSE w Warszawie	National Institute of Public Health - National Institute of Hygiene	Gierczyńki Rafał; Sadkowska-Todys Małgorzata; Wołkowicz Tomasz; Zacharczuk Katarzyna
EPI_ISL_2195442	WVU Rapid Development Lab	WVU and Marshall University Combined Genomics Core Facilities	James Denvir; Peter Perrotta; Peter Stoilov; Ryan Percifield; Wesley Kimble
EPI_ISL_1446420, EPI_ISL_1824013	WY Public Health Laboratory	Centers for Disease Control and Prevention Division of Viral Diseases, Pathogen Discovery	Alison Laufer Halpin; Ben L. Rambo-Martin; Clinton R. Paden; Dakota Howard; Darlene Wagner; Dave Wentworth; Dhwani Batra; Jasmine Padilla; Justin Lee; Katie Dillon; Krista Queen; Kristen Knipe; Kristine Lacek; Mark Burroughs; Matthew Schmerer; Mili Sheth; Peter Cook; Sam Shepard; Sarah Nobles; Shoshona Le; Suxiang Tong; Vivien Dugan; Yvette Unoarumhi
EPI_ISL_734400	Wadsworth Center, New York State Department.of Health	Wadsworth Center, New York State Department.of Health	Alexis Russel; Daryl M. Lamson; Erasmus Schneider; Erica Lasek-Nesselquist; John Kelly; Jonathan Plitnick; Kirsten St. George; Navjot Singh; Sara Griesemer
EPI_ISL_536791, EPI_ISL_649124, EPI_ISL_707794	Waikato Hospital	Institute of Environmental Science and Research (ESR)	Anja Werno; Antje van der Linden; Arlo Upton; Chris Mansell; David Hammer; Dragana Drinkovic; Erasmus Smit; Gary McAuliffe; Hana Sofia Andersson; Hermes Perez; James Ussher; Jill Sherwood; Jing Wang; Joep de Ligt; Josh Freeman; Julia Howard; Juliet Elvy; Lauren Jelly; Mary DeAlmeida; Matt Blakiston; Matt Storey; Matthew Rogers; Max Bloomfield; Michael Addidle; Michelle Balm; Muhammad Faisal; Nikki Freed; Olin Silander; Sally Roberts; Sarah Jefferies; Sharmini Muttaiyah; Susan Morpeth; Susan Taylor; Timothy Blackmore; Vani Sathyendran; Veronica Playle; Virginia Hope; Xiaoyun Ren
EPI_ISL_474054	Wales Specialist Virology Centre Sequencing lab: Pathogen Genomics Unit	COVID-19 Genomics UK (COG-UK) Consortium	Alec Birchley; Alexander Adams; Amy Gaskin; Angela Marchbank; Bree Gatica-Wilcox; Catherine Moore; Jason Coombes; Joanne Watkins; Joel Southgate; Johnathan Evans; Laura Gifford; Lauren Gilbert; Lee Graham; Malorie Perry; Matthew Bull; Nicole Pacchiarini; Sally Corden; Sara Kumziene-Summerhayes; Sara Rey; Sarah Taylor; Simon Cottrell; Sophie Jones; Tom Connor
EPI_ISL_1833202, EPI_ISL_2520676, EPI_ISL_2520706	Wales Specialist Virology Centre Sequencing lab: Pathogen Genomics Unit	Public Health Wales Microbiology Cardiff Wales Specialist Virology Centre	Alec Birchley; Alexander Adams; Amy Gaskin; Angela Marchbank; Bree Gatica-Wilcox; Catherine Moore; Jason Coombes; Joanne Watkins; Joel Southgate; Johnathan Evans; Laura Gifford; Lauren Gilbert; Lee Graham; Malorie Perry; Matthew Bull; Nicole Pacchiarini; Sally Corden; Sara Kumziene-Summerhayes; Sara Rey; Sarah Taylor; Simon Cottrell; Sophie Jones; Tom Connor
EPI_ISL_2096118	Washington State Department of Health Public Health Laboratories	Washington State Department of Health Public Health Laboratories	Avi Singh; Darren Lucas; Denny Russell; Drew MacKellar; Geoff Melly; Hannah Gray; Joenice Gonzalez; JohnAric Peterson; Philip Dykema; Rebecca Cao; Vanessa De Los Santos

EPI_ISL_413455	Washington State Public Health Lab	University of Washington Virology Lab	Alexander Greninger; Arun Nalla; Hong Xie; Keith Jerome; Pavitra Roychoudhury
EPI_ISL_476021, EPI_ISL_493071	Washington University in St. Louis	Washington University in St. Louis	Carey-Ann Burnham; David Wang; Lindsay Droit; Scott Handley; Stephen Tahan
EPI_ISL_2031771	Weill Cornell Medicine	New York Genome Center	Andre Corvelo; Arryn Craney; Chris Mason; Dayna M. Oschwald; Hanna Rennert; Lars F Westblade; Margaret Elizabeth Ross; Melissa Cushing; Michael Zody; Olivier Elemento; Priya Velu; Samantha Fennessey; Tom Maniatis
EPI_ISL_416539	Wellington Hospital	Institute of Environmental Science and Research (ESR)	Craig Thornley; Erasmus Smit; Joep de Ligt; Lauren Jelly; Matt Storey; Maxim Bloomfield; Xiaoyun Ren
EPI_ISL_649125, EPI_ISL_1016878, EPI_ISL_1250695	Wellington SCL (WN)	Institute of Environmental Science and Research (ESR)	Anja Werno; Antje van der Linden; Arlo Upton; Chris Mansell; David Hammer; Dragana Drinkovic; Erasmus Smit; Gary McAuliffe; Hana Sofia Andersson; Hermes Perez; James Ussher; Jill Sherwood; Jing Wang; Joep de Ligt; Josh Freeman; Julia Howard; Juliet Elvy; Lauren Jelly; Mary DeAlmeida; Matt Blakiston; Matt Storey; Matthew Rogers; Max Bloomfield; Michael Addidle; Michelle Balm; Muhammad Faisal; Nikki Freed; Olin Silander; Olivia Stroeven; Rachel Boyle; Sally Roberts; SallyAnn Harbison; Sarah Jefferies; Sharmini Muttaiyah; Susan Morpeth; Susan Taylor; Timothy Blackmore; Vani Sathyendran; Veronica Playle; Virginia Hope; Xiaoyun Ren
EPI_ISL_451366	West China Hospital of Sichuan University	State Key Laboratory of Biotherapy of Sichuan University	Baowen Du; Binwu Ying; Chao Tang; Chuan Chen; Hancheng Wei; Jia Geng; Jing-wen Lin; Lu Chen; Mingxia Yu; Minjin Wang; Weimin Li; Yongzhao Zhou
EPI_ISL_2517255, EPI_ISL_2722640, EPI_ISL_2734310	West of Scotland Specialist Virology Centre, NHSGGC / MRC-University of Glasgow Centre for Virus Research	COVID-19 Genomics UK (COG-UK) Consortium	Alasdair MacLean; Alice Broos; Ana da Silva Filipe; Antonia Ho; Daniel Mair; David L Robertson; Emma Thomson; Guy Mollett; Ioulia Tsatsani; James Shepherd; Jenna Nichols; Joseph Hughes; Kathy Li; Kathy Smollett; Kyriaki Nomikou; Lily Tong; Matthew Holden; Natasha Johnson; Rachel Blacow; Richard Orton; Rory Gunson; Sarah McDonald; Sharif Shaaban; Sreenu Vattipally; Stephen Carmichael
EPI_ISL_1760176, EPI_ISL_1760178, EPI_ISL_1760180, EPI_ISL_1805456, EPI_ISL_1922708, EPI_ISL_1940150, EPI_ISL_2497916, EPI_ISL_2534251, EPI_ISL_2627936			
see above	Wichita State University - Molecular Diagnostics Lab	Kansas Health and Environmental Lab	Ben Olsen; Jonathan Barnell; Katherine Wiggins; Mike Grose; and Phil Adam
EPI_ISL_471201, EPI_ISL_1794262, EPI_ISL_1794299, EPI_ISL_1794552, EPI_ISL_2279192	Wisconsin State Laboratory of Hygiene Communicable Disease Division	Wisconsin State Laboratory of Hygiene Communicable Disease Division	Abigail C. Shockey; Alicia J. Mooney; Erika M. Hanson; Kelsey R. Florek; Richard Griesser; Sara Wagner; Tonya Danz
EPI_ISL_2241381	Wojewodzka Stacja Sanitarno-Epidemiologiczna w Olsztynie, Laboratorium Badan Epidemiologiczno-Klinicznych	Wojewodzka Stacja Sanitarno-Epidemiologiczna w Olsztynie, Laboratorium Badan Epidemiologiczno-Klinicznych	Aleksandra Kobiatko; Barbara Dolinska; Emilia Tarabasz; Ewa Liszewska; Marta Lukian; Monika Czerminska; Patryk Bielecki; Paulina Rozycka; Sylwia Krzetowska; Tomasz Jakubczak
EPI_ISL_2547364	Worcester Hospital wc WOC	National Health Laboratory Service/University of Cape Town (NHLS/UCT)	Arash Iranzadeh; Bruna Galvao; Carolyn Williamson; Deelan Doolabh; Diana Hardie; Gert Marais; Innocent Mudau; Lynn Tyers; Marvin Hsiao; Stephen Korsman
EPI_ISL_455376	Wuhan Chain Medical Labs (CMLabs)	State Key Laboratory of Biotherapy of Sichuan University	Baowen Du; Binwu Ying; Chao Tang; Chuan Chen; Hancheng Wei; Jia Geng; Jing-wen Lin; Lu Chen; Mingxia Yu; Minjin Wang; Weimin Li; Yongzhao Zhou
EPI_ISL_462921, EPI_ISL_614248	Wyoming Public Health Laboratory	Center for Global Health, University of New Mexico Health Sciences Center	Cari Sloma; Darrell Dinwiddie; Daryl Domman; Kurt Schwalm; Noah Hull; Rob Christensen; Wanda Manley
EPI_ISL_539796, EPI_ISL_540921, EPI_ISL_1503290, EPI_ISL_1511254, EPI_ISL_1919582, EPI_ISL_2501965	Wyoming Public Health Laboratory	Wyoming Public Health Laboratory	Ashley Norberg; Brian Dominguez; Cari Sloma; Channing Weber; Chayse Rowley; Elliot Thomasson; Jim Mildenberger; Joel Sevinsky; Lynette Gumbleton; Marley Goetz; Noah Hull; Rob Christensen; Robert Petit; Sam Britz; Taylor Fearing; Wanda Manley; and Rob Christensen; and Wanda Manley
EPI_ISL_1225592	Yale Clinical Virology Lab	Grubaugh Lab - Yale School of Public Health	Anderson Brito; Annie Watkins; Chaney Kalinich; Chantal Vogels; Isabell Ott; Joseph Fauver; Mallery Breban; Marie L. Landry; Mary Petrone; Nathan Grubaugh; Tara Alpert
EPI_ISL_2035068	Yale Clinical Virology Lab	Yale Center for Genomic Analysis	Brooke Sullivan; Curt Scharfe; Irina Tikhonova; Kaya Bilguvar; Shrikant Mane
EPI_ISL_1595852, EPI_ISL_1595853	Yunan Center for Disease Control and Prevention	National Institute for Viral Disease Control and Prevention, China CDC	Jienan Zhou; Meiling Zhang; Senquan Jia; Xiang Zhao; Xiaoqing Fu; Yenan Feng; Yuchao Wu; Zhixiao Chen
EPI_ISL_1911195, EPI_ISL_1911196, EPI_ISL_1911197, EPI_ISL_1911250	Zhejiang Provincial Center for Disease Control and Prevention Zhoushan Center for Disease Prevention and Contol	Zhejiang Province Center of Disease Control and prevention	Bing WU; Hongling Wang; Yanjun Zhang
EPI_ISL_468153	[Romania, Bucharest] National Institute for Infectious Diseases “Prof. Dr. Matei Balș”	[Romania, Bucharest] National Institute for Infectious Diseases “Prof. Dr. Matei Balș”	Corina Casangiu; Leontina Banica; Marius Cotic; Marius Surleac; Simona Paraschiv
EPI_ISL_447912	n/a	National Institute of Health. Department of medical Sciences, Ministry of Public Health, Thailand	Chittaganpitch; Malinee; Okada; Parnmen; Phuygun; Pilailuk; Siripaporn; Sittiporn; Sunthareeya; Thanadachakul; Thanutsapa; Waicharoen; Warawan; Wongboot
EPI_ISL_458286	unknown	Bundeswehr Institute of Microbiology	Antwerpen; Bestehorn-Willmann; Eckstein, S.; Handrick, S.; M.C.; M.H.; M.S.; Naija, H.; R. and Ben Moussa, M.; Rehn, A.; Stoecker, K.; Walter; Woelfel
EPI_ISL_463006	unknown	Clinical virology	Fares, W.; Triki, H.
EPI_ISL_1738871, EPI_ISL_2248966, EPI_ISL_2445733	unknown	Instituto Nacional de Saude (INSA)	Borges et al
EPI_ISL_476559	unknown	Laboratoire Sciences et Technologies de la Santé (STS) Institut Supérieur des Sciences de la Santé Université Hassan 1er, Settat, Morocco	Abderraouf Hilali; Amal Souiri; Hajar Lemriss; Jawad Bouzid; Mustapha Mouallif; Narjis Amar; Sanaâ Lemriss; Saâd EL Kabbaj; Touria Essayagh
EPI_ISL_483060	unknown	Microbiology, Canterbury Health Laboratories	Anderson, T.; Dilcher, M.
EPI_ISL_640073	unknown	NHLS/UCT	Arash Iranzadeh; Bruna Galvao; Carolyn Williamson; Deelan Doolabh; Diana Hardie; Innocent Mudau; Kruger Marais; Lynn Tyers; Marvin Hsiao; Stephen Korsman
