# Supplementary material for: Two Separate Clusters of SARS-CoV-2 Delta Variant Infections in a Group of 41 Students Travelling from India: An Illustration of the Need for Rigorous Testing and Quarantine
Source: Viruses. 2022 May 31;14(6):1198. doi: 10.3390/v14061198 (PMC9229483; doi:10.3390/v14061198)
Supplement: Supplementary file 1 [file viruses-14-01198-s001.zip › Viruses_1730360_OnlinsSupplement_revision.pdf]

Online supplement

Legend of Supplementary Figures

Supplementary Figure 1: All of the mutations/deletions (compared to Wuhan wild type) that characterize each of the clusters, along with their associated phylogeny.

Supplementary Figure 2: Overall SARS-CoV-2 phylogeny, with the sequences from this manuscript highlighted as maroon points.

Supplementary Table 1: RT-PCR and IgG anti-SARS CoV-2 results (Day 1 = 12/4/2021 (Day of arrival in Belgium))

	**PCR**					**Phylogenetic analysis**	**IgG anti-N (S/CO)**	**IgG anti-S (AU/mL)**	**Vaccination**
**House**	**Day 6 -7**	**Day 9-10**	**Day 15**	**Day 18**	**Day 24**		**Day 15**	**Day 15**	
A	Neg	**Strong** Ct 19.9	**Very strong** Ct 14.2	**Moderate** Ct 21.9	**Moderate** Ct 27.9	Cluster 1	Neg	Neg	
A	Neg	**Strong** Ct 17.6	**Moderate** Ct 23.0	*Weak Ct 29.0*	*Weak Ct 29.4*	Cluster 1	Neg	Neg	
A	Neg	**Strong** Ct 18.8	**Strong** Ct 17.0	**Moderate** Ct 24.1	**Moderate** Ct 25.5	Cluster 1	Neg	Neg	
A	Neg	**Strong** Ct 19.4	**Strong** Ct 18.2	**Moderate** Ct 25.9	**Moderate** Ct 27.1	Cluster 1	Neg	Neg	
A	**Strong** Ct 21.2	/	*Weak Ct 29.5*	Neg	*Weak Ct 30.6*	Cluster 1	**Positive (1.54)**	**Positive (423)**	
A	Neg	/	**Strong** Ct 17.2	**Strong** Ct 18.1	**Moderate** Ct 25.5	Cluster 1	Neg	Neg	
A	Neg	/	**Very strong** Ct 14.2	**Moderate** Ct 23.6	*Weak Ct 28.5*	Cluster 1	Neg	Neg	

A	Neg	/	**Very strong** Ct 14.1	**Moderate** Ct 22.9	**Moderate** Ct 26.3	Cluster 1	Neg	Neg	
A	Neg	/	Neg	Neg	*Weak Ct 31.6*	Untypeable	**Positive (2.05)**	**Positive (51769)**	**2x Pfizer**
B	Neg	**Strong** Ct 19.3	**Strong** Ct 19.1	*Weak Ct 29.3*	*Weak Ct 32.2*	Cluster 2	Neg	Neg	
B	**Strong** Ct 20.5	/	**Moderate** Ct 26.7	*Weak Ct 30.3*	*Weak Ct 29.9*	Cluster 2	**Grey zone (0.98)**	**Positive (67716)**	**2x Covishield**
B	Neg	**Strong** Ct 17.5	**Moderate** Ct 23.2	*Weak Ct 28.7*	*Weak Ct 30.1*	Cluster 2	Neg	Neg	
B	Neg	**Strong** Ct 18.7	**Strong** Ct 19.0	**Strong** Ct 21.0	**Moderate** Ct 26.2	Cluster 2	Neg	Neg	
B	*Weak Ct 36.3*	/	**Moderate** Ct 24.9	*Weak Ct 29.6*	**Moderate** Ct 27.0	Cluster 2	Neg	Neg	
B	Neg	/	**Very strong** Ct 13.7	**Strong** Ct 20.8	**Moderate** Ct 27.7	Cluster 2	Neg	**Positive (23055)**	**2x Pfizer**
B	Neg	/	**Moderate** Ct 24.0	*Weak Ct 33.2*	*Weak Ct 30.4*	Cluster 2	Neg	Neg	**1x Pfizer**
B	*Weak* Ct 31.2	/	**Strong** Ct 21.0	*Weak Ct 29.0*	*Weak Ct 33.6*	Cluster 2	**Positive (4.09)**	**Positive (1762)**	
B	Neg	/	*Weak Ct 30.1*	Neg	Neg	Untypeable	Neg	Neg	
G=>C	*Weak Ct 31.6*	/	*Weak Ct 30.4*	*Weak Ct 31.1*	*Weak Ct 31.8*	Untypeable	**Positive (5.78)**	**Positive (5588)**	
C	**Very strong** Ct 9.9	/	**Moderate** Ct 23.6	**Moderate** Ct 25.9	**Moderate** Ct 27.6	Cluster 2	**Positive (1.45)**	Neg	
C	**Very strong** Ct 10.3	/	**Moderate** Ct 23.3	**Moderate** Ct 26.8	*Weak Ct 29.1*	Cluster 2	**Positive (5.06)**	**Positive (231)**	

C	**Very strong** Ct 10.1	/	**Moderate** Ct 25.3	**Moderate** Ct 25.0	*Weak* *Ct 29.4*	Cluster 2	Neg	**Positive (100)**	
C	**Strong** Ct 18.8	/	**Moderate** Ct 26.6	*Weak* *Ct 29.9*	*Weak* *Ct 30.7*	Cluster 2	**Positive (4.67)**	**Positive (30456)**	**2x Covishield**
D	**Moderate** Ct 22.5	/	**Moderate** Ct 21.4	**Strong** Ct 18.5	**Moderate** Ct 26.3	Cluster 2	Neg	Neg	
D	**Very strong** Ct 10.9	/	**Strong** Ct 20.8	**Moderate** Ct 24.7	*Weak* *Ct 29.2*	Cluster 2	Neg	**Positive (179457)**	**1x Pfizer**
D	**Very strong** Ct 9.7	/	**Moderate** Ct 21.5	**Moderate** Ct 25.9	**Moderate** Ct 27.1	Cluster 2	Neg	**Positive (123)**	
D	**Very strong** Ct 10.8	/	**Moderate** Ct 24.6	**Moderate** Ct 25.2	**Moderate** Ct 27.5	Cluster 2	**Positive (1.65)**	**Positive (101)**	
E	Neg	/	Neg	Neg	/		Neg	Neg	
E	Neg	/	Neg	Neg	/		Neg	Neg	
E	Neg	/	Neg	Neg	/		Neg	Neg	
F	Neg	Neg	Neg	Neg	/		Neg	Neg	
F	Neg	Neg	Neg	Neg	/		Neg	**Positive (252)**	**1x Pfizer**
F	Neg	Neg	Neg	Neg	/		Neg	Neg	
F	Neg	Neg	Neg	Neg	/		Neg	Neg	
G	Neg	Neg	Neg	Neg	/		Neg	Neg	
G	Neg	Neg	Neg	Neg	/		Neg	**Positive (1778)**	**2x Covishield**
G	Neg	Neg	Neg	Neg	/		Neg	Neg	
G	Neg	Neg	Neg	Neg	/		Neg	Neg	
G	Neg	Neg	Neg	Neg	/		Neg	Neg	
H	Neg	Neg	Neg	Neg	/		Neg	Neg	

H	Neg	Neg	Neg	Neg	/		Neg	Neg	
H	Neg	Neg	Neg	Neg	/		Neg	Neg	
H	Neg	Neg	Neg	Neg	/		Neg	**Positive (53)**	

Untypeable: no sequencing possible due to low viral laod. Grey italic text: Weak result. Ct: cycle threshold, /: not tested that day.

Supplementary Table 2: GISAID acknowledgments (see separate document)

COG-Belgium consortium or COVID-19 Genomics Belgium consortium

National Reference Center for Respiratory Pathogens UZ/KU Leuven

Emmanuel André (emmanuel.andre@uzleuven.be)

Piet Maes (piet.maes@kuleuven.be)

Guy Baele (guy.baele@kuleuven.be)

Simon Dellicour (simon.dellicour@kuleuven.be)

Lize Cuypers (lize.cuypers@uzleuven.be)

Marc Van Ranst (marc.vanranst@uzleuven.be)

Laboratory of Proteomics and Microbiology, UMONS

François E. Dufrasne (francois.dufrasne2@umons.ac.be)

Guillaume Bayon-Vicente (guillaume.bayon-vicente@umons.ac.be)

Ruddy Wattiez (ruddy.wattiez@umons.ac.be)

AZ Klina

Carl Vael (carl.vael@klina.be)

Lynsey Berckmans (lynsey.berckmans@klina.be)

Virology Unit, Department of Biomedical Sciences, Institute of Tropical Medicine Antwerp & Department of Biomedical Sciences, University of Antwerp

Philippe Selhorst

Kevin K. Ariën

Brightcore group

Université libre de Bruxelles (ULB)

Arnaud MARCHANT

Coralie HENIN

Benoit Haerlingen

Ricardo De Mendonca (additional affiliation: Hôpital Erasme)

Institut de Biologie Clinique

Marie-Luce DELFORGE (additional affiliation: Hôpital Erasme)

UZ Brussel

Sonia Van Dooren - Sonia.VanDooren@uzbrussel.be

Bruno Hinckel - Bruno.Hinckel@uzbrussel.be

Hideo Imamura - Hideo.Imamura@uzbrussel.be

Toon Janssen - Toon.Janssen@uzbrussel.be

Ben Caljon - Ben.Caljon@uzbrussel.be

Oriane Soetens – oriane.soetens@uzbrussel.be

Denis Piérard – denis.pierard@uzbrussel.be

Thomas Demuyser – thomas.demuyser@uzbrussel.be

LHUB-ULB

Charlotte Martin

UCLouvain

IREC – Medical Microbiology unit (MBLG)

Jean Ruelle jean.ruelle@uclouvain.be

Benoit Kabamba Mukadi benoit.kabamba@uclouvain.be (additional affiliation: Cliniques Universitaires Saint-Luc – Department of clinical laboratories)

Center for Applied Molecular Technologies (CTMA), Institut de Recherche Expérimentale et Clinique (IREC), Université Catholique de Louvain

Professor Jean-Luc Gala MD PhD : jean-luc.gala@uclouvain.be

Bertrand Bearzatto PhD : bertrand.bearzatto@uclouvain.be

Jérôme Ambroise PhD : jerome.ambroise@uclouvain.be

GZA/ZNA

Van Lint Philippe Philippe.VanLint@gza.be

Walter Verstrepen, Pharm D, PhD; walter.verstrepen@zna.be

Reinout Naesens, MD: reinout.naesens@zna.be

Sciensano

Michael Peeters

Kate Bakelants

Sarah Denayer

Sofieke Klamer

IPG

Pascale Hilbert, PhD pascale.hilbert@ipg.be

Sylvain Brohée, MD sylvain.brohee@ipg.be

Pierre-Emmanuel Léonard, MSc pierre-emmanuel.leonard@ipg.be

Deniz Karadurmus, PhD deniz.karadurmus@ipg.be

Jeremie Gras, MD jeremie.gras@ipg.be

Damien Féret, MSc damien.feret@ipg.be

Barbara Lambert MD, PhD barbara.lambert@ipg.be

OLVZ Aalst

Anne Vankeerberghen (anne.vankeerberghen@olvz-aalst.be)

Astrid Holderbeke (astrid.holderbeke@olvz-aalst.be)

Hans De Beenhouwer (hans.de.beenhouwer@olvz-aalst.be)

Lien Cattoir, lien.cattoir@olvz-aalst.be

Department of Microbiology, University Hospital Antwerp, Edegem & Laboratory of Medical Microbiology, Vaccine & Infectious Disease Institute (VAXINFECTIO), University of Antwerp, Wilrijk

Christine Lammens, christine.lammens@uantwerpen.be (only university affiliation)

Basil Britto Xavier, basilbritto.xavier@uantwerpen.be (only university affiliation)

Marie Le Mercier, marie.lemercier@uza.be (only hospital affiliation)

Jasmine Coppens, jasmine.coppens@uza.be (only hospital affiliation)

Veerle Matheeussen, veerle.matheeussen@uza.be

Herman Goossens, herman.goossens@uza.be

Department of Laboratory Medicine, AZ Delta General Hospital, Roeselare, Belgium

Vanhee Merijn merijn.vanhee@azdelta.be

Geert A. Martens geert.martens@azdelta.be

Koen Swaerts koen.swaerts@azdelta.be

Frederik Van Hoecke frederik.vanhoecke@azdelta.be

Dieter Desmet dieter.desmet@azdelta.be

Clinical Biology Lab, CHU UCL Namur

BOGAERTS Pierre pierre.bogaerts@uclouvain.be

DEGOSSERIE Jonathan jonathan.degosserie@uclouvain.be

DENIS Olivier olivier.denis@uclouvain.be

HUANG Te-Din te-din.huang@uclouvain.be

Briant network

Imelda

Dagmar Obbels

Hanne Valgaeren

Johan Frans

Annick Smismans

Paul-Emile Claus

AZ St Maarten Mechelen

Truus Goegebuer

Ann Lemmens

AZ Jan Portaels

Bea Van den Poel Bea.vandenpoel@azjanportaels.be

Sonja De Bock sdebock@azjanportaels.be

Lier

Wim Laffut (wim.laffut@heilighartlier.be)

Ellen Van Even (ellen.van.even@heilighartlier.be)

AZ St Lucas Gent

Jos Van Acker (jos.vanacker@azstlucas.be)

Charlotte Verfaillie (charlotte.verfaillie@azstlucas.be)

Elke Vanlaere (elke.vanlaere@azstlucas.be)

Klara De Rauw (klara.derauw@azstlucas.be)

Jessa

Brigitte Maes brigitte.maes@jessazh.be

Guy Froyen guy.froyen@jessazh.be

Bert Cruys bert.cruys@jessazh.be

Ellen Geerdens ellen.geerdens@jessazh.be

Luc Waumans luc.waumans@jessazh.be

Britt Van Meensel britta.vanmeensel@jessazh

Reinoud Cartuyvels reinoud.cartuyvels@jessazh.be

Severine Berden severine.berden@jessazh.be

Marijke Raymaekers marijke.raymaekers@jessazh.be,

UZ and UGent

Bruno.verhasselt@uzgent.be

CHU and U Liège

Cécile Meex

Keith Durkin

Laurent Gillet

Maria Artesi

Marie-Pierre Hayette

Sébastien Bontems

Vincent Bours

AZ St-Jan Brugge

Jorn Hellemans

Patrick Descheemaeker

Marijke Reynders
