# Supplementary figures and images for: Two Separate Clusters of SARS-CoV-2 Delta Variant Infections in a Group of 41 Students Travelling from India: An Illustration of the Need for Rigorous Testing and Quarantine
Source: Viruses. 2022 May 31;14(6):1198. doi: 10.3390/v14061198 (PMC9229483; doi:10.3390/v14061198)

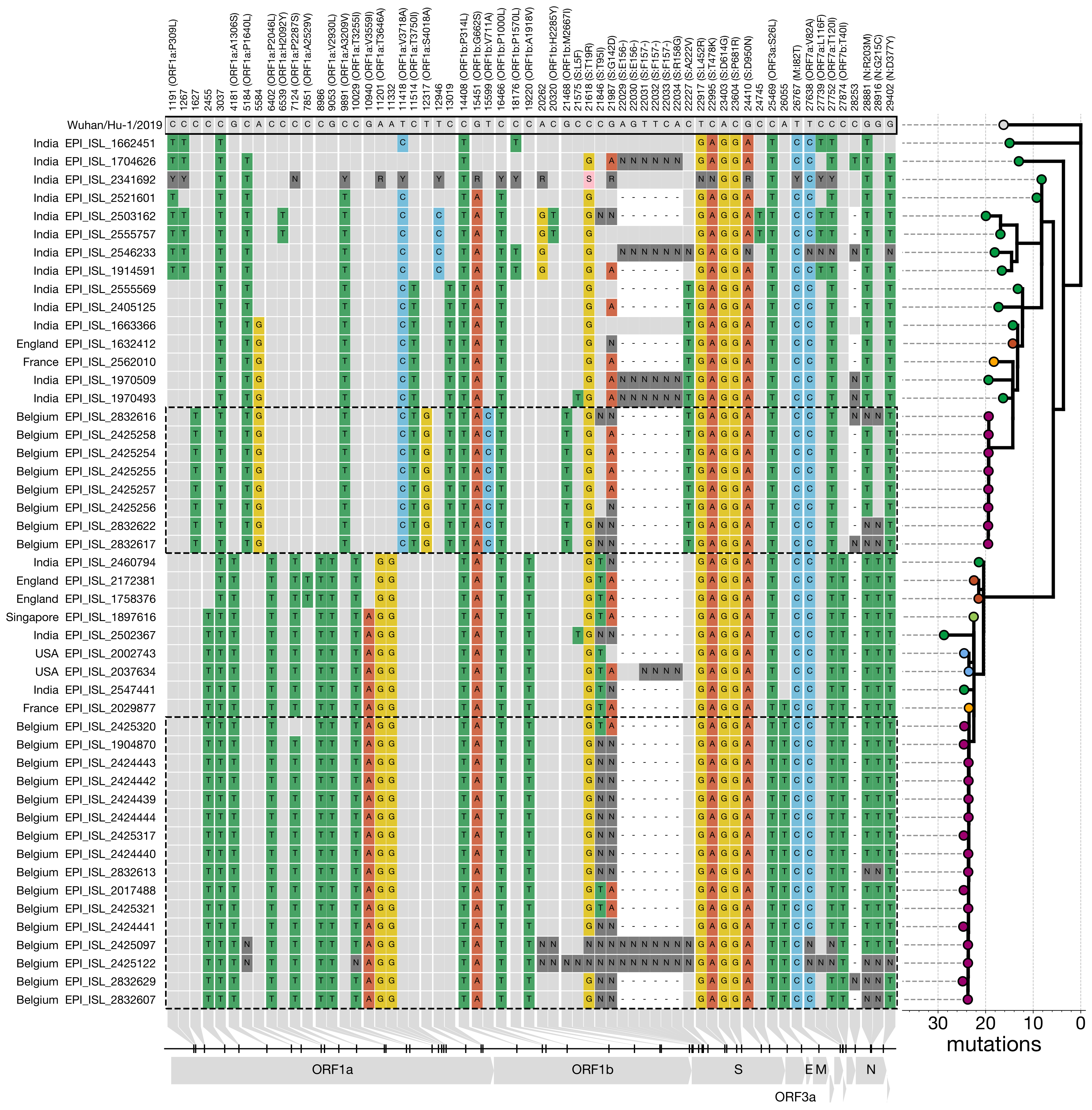

Supplement: Supplementary file 1 [file viruses-14-01198-s001.zip › Supplementary Figure S1.tiff]

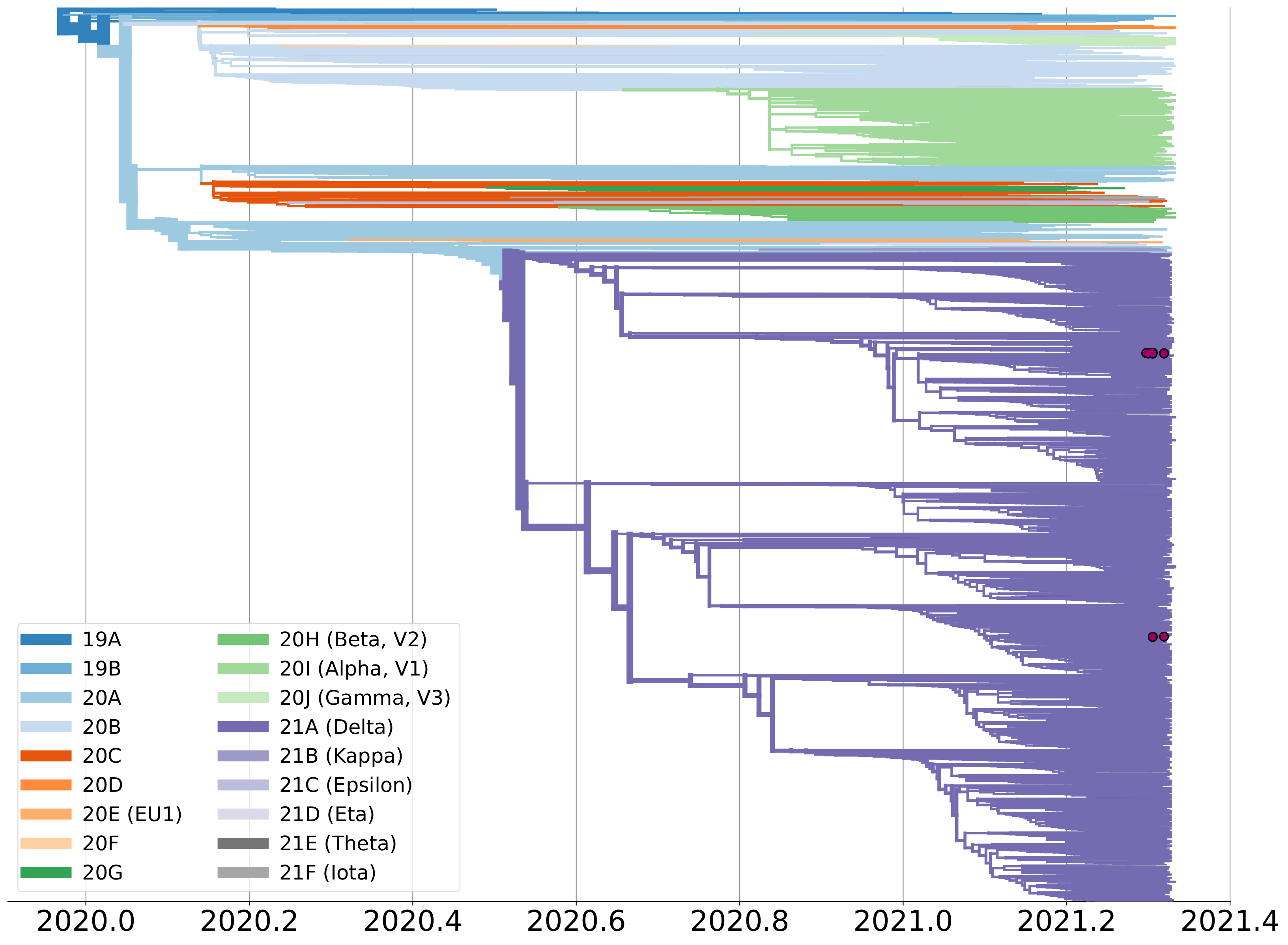

Supplement: Supplementary file 1 [file viruses-14-01198-s001.zip › Supplementary Figure S2.tiff]
